# Supplementary material for: Methodological choices and clinical usefulness for machine learning predictions of outcome in Internet-based cognitive behavioural therapy
Source: Commun Med (Lond). 2024 Oct 10;4:196. doi: 10.1038/s43856-024-00626-4 (PMC11464669; doi:10.1038/s43856-024-00626-4)
Supplement: Supplementary file 2 — Supplementary Information [file 43856_2024_626_MOESM2_ESM.pdf]

Supplementary information

Manuscript

Methodological choices and clinical usefulness for machine learning predictions of outcome in Internet-based cognitive behavioral therapy

Due to the extensive methodological investigation this supplement contains more details for the main paper. Code for prediction procedure and for analysing the results is found on github on the following link: https://github.com/intraverbal/paper_ipsy_outcome_pred [1]

Contents

Supplementary Methods.

Supplementary Results.

Supplementary References.

Supplementary notes 1.

Supplementary notes 2.

Supplementary Methods.

Data type

The homework variables were extensively feature engineered and consisted of constructing a representation of the text answers using the mean representation of a normalized vector created by Word2Vec [2] implemented using genism in python. [3] This was done first by building the vocabulary on all the answers for the specified homework, then a general representation of the answer was created by taking the mean vector of the representation of the entire answer. For the activity schedule filled in by patients in depression treatment, k-means clustering [4] was used on these general vector representations to be able to classify answers to each specific timeslot in the homework. The answers to all the timeslots were then aggregated again using k-means, yielding one classification per patient. For patients in treatment for social anxiety disorder or panic disorder the cosine-similarity of the answers to questions in regards to their anxiety before and after an exposure exercise was used as the aggregated measure.

Software information

We used Python 2.7.1 and scikit-learn [5] for the implementation of the machine learning algorithms, and their evaluation. For visualization we used the ggplot2 [6] package, and for summary of datasets we used DataMaid [7], both implemented in R. [8]

Supplementary Results.

Models always predicted the continuous outcome score and was subsequently transformed into a dichotomised form for the calculation of balanced accuracy to facilitate comparison to other research in the field. However, the dichtomisation of outcome itself gives the following base rates of success in the different treatments: 49% in major depressive disorder, 74% in panic disorder, 28% in social anxiety disorder, and 50% for all patients.

Accuracy over time

There was a general pattern of increasing accuracy of prediction over time see Figure 1. The best performing setup (as measured by balanced accuracy) for timepoint ‘pre’ and imputed data was using all patients, handpicked variables and random forest with evaluative scores (balanced accuracy in cross-validation, balanced accuracy in holdout, r^2 in cross-validation, r^2 in holdout) being (72.59%, 69.15%, 0.37, 0.318). However, for the other treatment groupings other setups were superior: for depression linear PCA variable selection with bayesian ridge regression (65.34%, 61.65%, 0.313, 0.228), for social anxiety benchmark variable selection with random forest (71.86%, 74.3%, 0.536, 0.516), and for panic disorder the benchmark variable selection with adaboost regressor (66.37%, 56.37%, 0.239, 0.06).

Supplementary Figure 1. Accuracy over time

Supplementary Figure 1. Showing progressive increasing accuracy as a function of time in treatment.

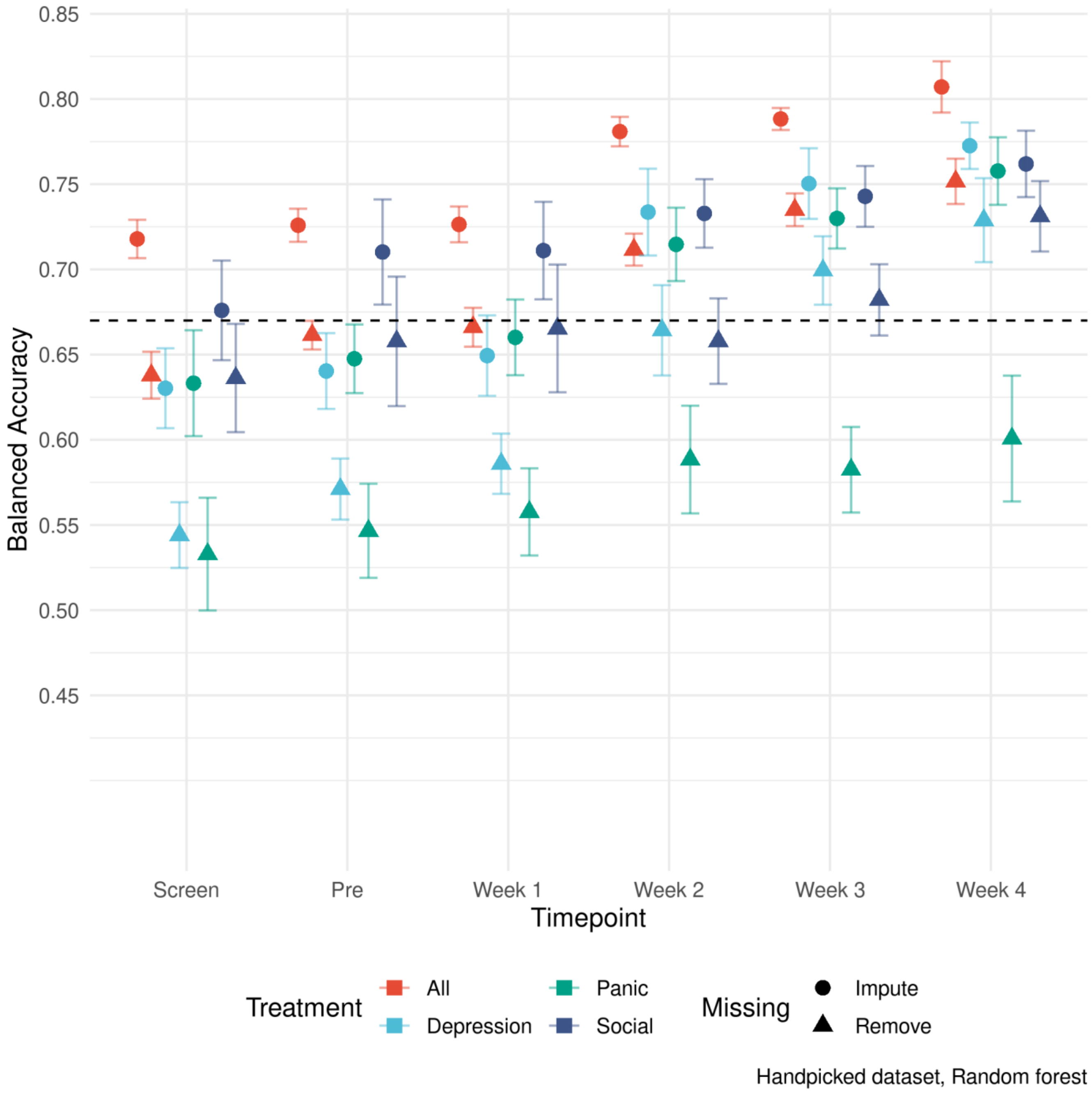

Here Random forest and the handpicked variables were used, but the variation over different methods and variables is limited.

Supplementary Model 1. Benchmark model.

Predictor	*b*	*b* 95% CI [LL, UL]	*sr*^{2}	*sr*^{2} 95% CI [LL, UL]	Fit
(Intercept)	-0.14**	[-0.16, -0.12]			
sex	0.09**	[0.05, 0.12]	.00	[.00, .00]	
age	-0.01	[-0.03, 0.01]	.00	[-.00, .00]	
PDSS.SR _SCREEN	0.06**	[0.04, 0.07]	.00	[.00, .01]	
MADRS _SCREEN	-0.04**	[-0.06, -0.02]	.00	[.00, .00]	
LSAS_SCREEN	0.09**	[0.07, 0.11]	.01	[.00, .01]	
mainsymptom_PRE	-0.10**	[-0.14, -0.07]	.00	[.00, .00]	
mainsymptom_WEEK 01	0.08**	[0.05, 0.12]	.00	[.00, .00]	
mainsymptom_WEEK 02	0.19**	[0.16, 0.23]	.01	[.00, .01]	
mainsymptom_WEEK 03	0.52**	[0.49, 0.55]	.07	[.06, .08]	
					R^2 = .539** 95% CI[.52,.55]

Note. A significant b-weight indicates the beta-weight and semi-partial correlation are also significant. b represents standardized regression weights. sr2 represents the semi-partial correlation squared. LL and UL indicate the lower and upper limits of a confidence interval, respectively.* indicates p < .05. ** indicates p < .01. Produced by apaTables [9] implemented in R version 3.6.0 [8]. All Variables (including outcome) are standardized using z-transformation before training except “sex” where 0 = female and 1 = male. PDSS.SR = Panic Disorder Symptom Scale-Self Report. MADRS = Montgomery-Åsberg Depression Rating Scale-Self report. LSAS = Leibowitz Social Anxiety Scale-Self Report version. Mainsymptom is MADRS for those in depression treatment, PDSS-SR for those in treatment for panic disorder and LSAS-SR for those in treatment for social anxiety disorder. The model reported here have not been evaluated in external samples and because of this the accuracy in novel sample is unknown.

Supplementary References

1. Hentati Isacsson, N. Software for Methodological choices and clinical usefulness for machine learning predictions of outcome in Internet-based cognitive behavioral therapy. (2024).
2. Mikolov, T., Chen, K., Corrado, G. & Dean, J. Efficient Estimation of Word Representations in Vector Space. *arXiv:1301.3781 [cs]* (2013).
3. Řehůřek, R. & Sojka, P. Software framework for topic modelling with large corpora. in *Proceedings of the LREC 2010 workshop on new challenges for NLP frameworks* 45–50 (ELRA, Valletta, Malta, 2010).
4. Hartigan, J. A. & Wong, M. A. Algorithm AS 136 A K-Means Clustering Algorithm. *Applied Statistics* **28**, 100 (1979).
5. Pedregosa, F. *et al.* Scikit-learn: Machine learning in Python. *Journal of Machine Learning Research* **12**, 2825–2830 (2011).
6. Wickham, H. *Ggplot2: Elegant Graphics for Data Analysis*. (Springer-Verlag New York, 2016).
7. Petersen, A. H. & Ekstrøm, C. T. dataMaid: Your assistant for documenting supervised data quality screening in R. *Journal of Statistical Software* **90**, 1–38 (2019).
8. R Core Team. *R: A Language and Environment for Statistical Computing*. (R Foundation for Statistical Computing, Vienna, Austria, 2019).
9. Stanley, D. *apaTables: Create American Psychological Association (APA) Style Tables*. (2018).

Supplementary notes 1

```
hyperparams = [
    (LinearSVR(random_state=0, max_iter=1500), {
    'epsilon': [0, 1],
    'loss': ['epsilon_insensitive','squared_epsilon_insensitive'],
    'C': [1.5, 1, 0.5, 0.2]}),
    (LinearRegression(), {
         'fit_intercept': [True]}),
    (ElasticNet(random_state=0), {
        'alpha': [3, 2, 1, 0.1],
        'l1_ratio': [0.8, 0.5, 0.2]}),
    (Ridge(random_state=0), {
    'solver': ['auto', 'svd','saga']}),
    (Lasso(random_state=0), {
        'alpha': [3, 2, 1, 0.1]}),
    (BayesianRidge(), {
    'tol': [0.01, 0.001, 0.0001],
    'alpha_1': [0.000001],
     'alpha_2': [0.000001],
     'lambda_1': [0.000001],
     'lambda_2':[0.000001]}),
    (KNeighborsRegressor(n_jobs= -1), {
    'n_neighbors': [25, 15, 10, 5],
    'weights': ['uniform', 'distance'],
    'algorithm': ['ball_tree','kd_tree','brute','auto'],
    'leaf_size': [30, 20, 10, 5]}),
    (RandomForestRegressor(random_state=0, n_jobs= -1), {
    'n_estimators': [300],
    'min_samples_split': [100, 50, 20, 10],
    'min_samples_leaf': [50, 25, 10, 5],
    'max_features': ['auto', 'sqrt', 'log2']}),
    (AdaBoostRegressor(random_state=0), {
    'n_estimators': [300],
    'learning_rate': [1],
    'loss': ['linear', 'square', 'exponential'],
    'base_estimator': [RandomForestRegressor(random_state=0,
                                             n_jobs=-1,
                                             min_samples_split=5,
                                             min_samples_leaf=2,
                                             max_features='auto')]}),
    (GradientBoostingRegressor(random_state=0), {
    'n_estimators': [300],
    'learning_rate': [1],
    'loss': ['ls', 'lad', 'huber'],
    'subsample': [1, 0.8, 0.5],
    'min_samples_split': [100, 50, 20, 10],
    'min_samples_leaf': [50, 25, 10, 5],
    'max_depth': [20, 10, 5, 3]})
]
```

Supplementary notes 2

Columns in supplementary data 2

Header	Explanation
Dataset	What dataset was used, treatment_time_imputation
shape	How many patients (x) how many variables (y). (x,y)
outcome	What outcome was predicted
pca	What type if any of PCA
pcaparams	What parameters did this PCA have
classifier	What algorithm was used
bestparams	What was the best parameters for this algorithm
meanscore	What was its mean score in the cross validation procedure
stdscore	What was its standard deviation
filename	What time of data selection was used

Columns in supplementary data 2 detailing the best hyperparameters for each dataset and algorithm.

Columns in supplementary data 3

Header	Explanation
Data_amount	Is data imputed or removed?
Data_shape	How many variables are included
FN	Mean false negatives in cross-validation sample
FN_ho	False negative in holdout sample
FN_std	Standard deviation of mean false negatives in cross-validation sample
FP	Mean false positives in cross-validation sample
FP_ho	False positive in holdout sample
FP_std	Standard deviation of false positives in cross-validation sample
Method	Algorithm / classifier used.
Outcome	What outcome was predicted - symptom outcome
PCA	How was data selected (e.g. handpicked or benchmark)
PCA_params	Was there any PCA parameters (relevant for PCA as data selection)
Params	Parameters used for the algorithm
TN	Mean true negatives in cross-validation sample
TN_ho	True negatives in holdout sample
TN_std	Standard deviation of true negatives in cross-validation sample
TP	Mean true positives in cross-validation sample
TP_ho	True positives in holdout sample
TP_std	Standard deviation of true positives in cross-validation sample
Time	Timepoint for prediction. E.g. week1 uses data up and until week 1.
Treatment	What patients were included (Depression, Panic, Social, All)
b.acc	Mean balanced accuracy in cross-validation sample
b.acc_ho	Balanced accuracy in hold out sample
b.acc_std	Standard deviation of balanced accuracy in cross-validation sample
f1	Mean F1 score in cross-validation sample
f1_ho	F1 score in hold out sample
f1_std	Standard deviation of balanced accuracy in in cross-validation sample
feat.imp_X	When using random forest derived methods, the "feature importance" of X feature.
Data_shape_org	Amount of patients (x) and features (y) (x,y).

Columns in supplementary data 3 detailing all metric results and details on datasets used.
