# Supplementary material for: Methodological choices and clinical usefulness for machine learning predictions of outcome in Internet-based cognitive behavioural therapy
Source: Commun Med (Lond). 2024 Oct 10;4:196. doi: 10.1038/s43856-024-00626-4 (PMC11464669; doi:10.1038/s43856-024-00626-4)
Supplement: Supplementary file 3 — Description of Additional Supplementary Files [file 43856_2024_626_MOESM3_ESM.pdf]

Description of Additional Supplementary Files

File name: Supplementary Data 1
Description: Codebooks for all datasets.

File name: Supplementary Data 2
Description: Best fitting hyperparameters

File name: Supplementary Data 3
Description: Results from all analyses

File name: Supplementary Software 1
Description: Code for prediction procedure, and code for reported results.
