# Supplementary material for: Methodological choices and clinical usefulness for machine learning predictions of outcome in Internet-based cognitive behavioural therapy
Source: Commun Med (Lond). 2024 Oct 10;4:196. doi: 10.1038/s43856-024-00626-4 (PMC11464669; doi:10.1038/s43856-024-00626-4)
Supplement: Supplementary file 4 — Supplementary Data 1 [file 43856_2024_626_MOESM4_ESM.pdf]

Handpicked_All_week04-imputed_benchmark_test

Autogenerated data summary from dataMaid

2023-01-09 13:10:32

Data report overview

The dataset examined has the following dimensions:

Feature	Result
Number of observations	669
Number of variables	10

Codebook summary table

Label	Variable	Class	# unique values	Missing	Description
	**sex**	factor	2	0.00 %	Sex of patient, 0 = Female, 1=Male
	**age**	numeric	56	0.00 %	
	**PDSS-SR-3064_SCREEN_sum**	numeric	79	0.00 %	Anxiety questionnaire, self rated-Timepoint before treatment starts-Sum of the entire measure
	**MADRS-1951_SCREEN_sum**	numeric	70	0.00 %	Depression questionnaire, self rated-Timepoint before treatment starts-Sum of the entire measure
	**LSAS-2241_SCREEN_sum**	numeric	202	0.00 %	Social anxiety questionnaire, self rated-Timepoint before treatment starts-Sum of the entire measure
	**outcome**	numeric	295	0.00 %	
	**mainsymptom_PRE_sum**	numeric	156	0.00 %	PDSS-SR for panic, MADRS for depression, LSAS for social anxiety-Timepoint just before beginning treatment-Sum of the entire measure
	**mainsymptom_WEEK01_sum**	numeric	242	0.00 %	PDSS-SR for panic, MADRS for depression, LSAS for social anxiety-Timepoint after one week in treatment-Sum of the entire measure
	**mainsymptom_WEEK02_sum**	numeric	244	0.00 %	PDSS-SR for panic, MADRS for depression, LSAS for social anxiety-Timepoint after two weeks in treatment-Sum of the entire measure

Label	Variable	Class	# unique values	Missing	Description
	**mainsymptom_WEEK03_sum**	numeric	250	0.00 %	PDSS-SR for panic, MADRS for depression, LSAS for social anxiety-Timepoint after three weeks in treatment-Sum of the entire measure

Variable list

sex

Feature	Result
Variable type	factor
Number of missing obs.	0 (0 %)
Number of unique values	2
Mode	“0”
Reference category	0

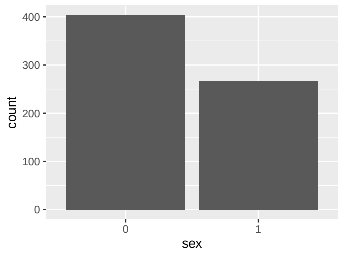

- Observed factor levels: "0", "1".

---

age

Feature	Result
Variable type	numeric
Number of missing obs.	0 (0 %)
Number of unique values	56
Median	-0.29
1st and 3rd quartiles	-0.81; 0.58
Min. and max.	-1.6; 3.73

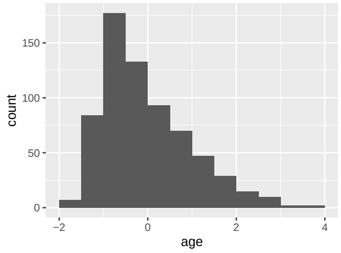

PDSS-SR-3064_SCREEN_sum

Feature	Result
Variable type	numeric
Number of missing obs.	0 (0 %)
Number of unique values	79
Median	-0.03
1st and 3rd quartiles	-0.82; 0.75
Min. and max.	-1.29; 2.96

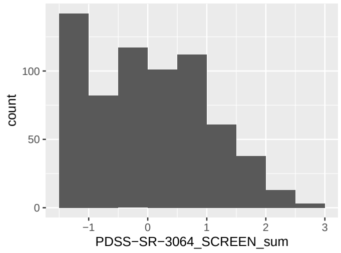

MADRS-1951_SCREEN_sum

Feature	Result
Variable type	numeric
Number of missing obs.	0 (0 %)
Number of unique values	70
Median	0.17
1st and 3rd quartiles	-0.57; 0.79
Min. and max.	-2.68; 3.02

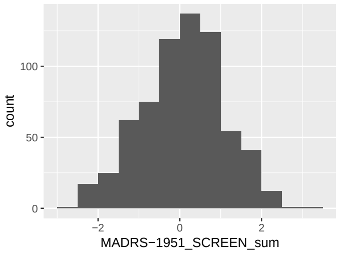

LSAS-2241_SCREEN_sum

Feature	Result
Variable type	numeric
Number of missing obs.	0 (0 %)
Number of unique values	202
Median	-0.06
1st and 3rd quartiles	-0.72; 0.75
Min. and max.	-1.79; 2.99

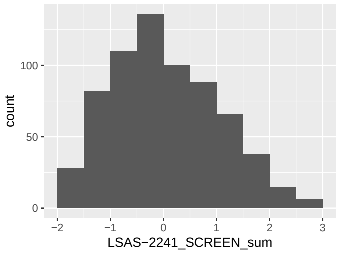

outcome

Feature	Result
Variable type	numeric
Number of missing obs.	0 (0 %)
Number of unique values	295
Median	0.11
1st and 3rd quartiles	-0.59; 0.69
Min. and max.	-2.05; 3.53

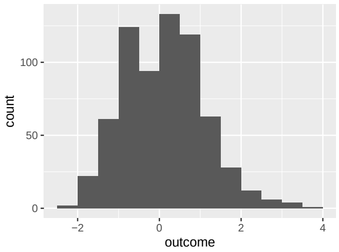

mainsymptom_PRE_sum

Feature	Result
Variable type	numeric
Number of missing obs.	0 (0 %)
Number of unique values	156
Median	0.34
1st and 3rd quartiles	-0.28; 0.89
Min. and max.	-2.62; 2.87

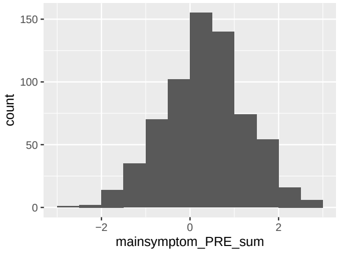

mainsymptom_WEEK01_sum

Feature	Result
Variable type	numeric
Number of missing obs.	0 (0 %)
Number of unique values	242
Median	0.23
1st and 3rd quartiles	-0.3; 0.89
Min. and max.	-2.84; 2.99

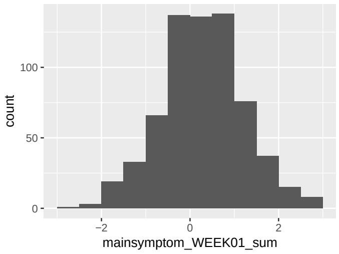

---

mainsymptom_WEEK02_sum

Feature	Result
Variable type	numeric
Number of missing obs.	0 (0 %)
Number of unique values	244
Median	0.18
1st and 3rd quartiles	-0.34; 0.92
Min. and max.	-2.75; 3.13

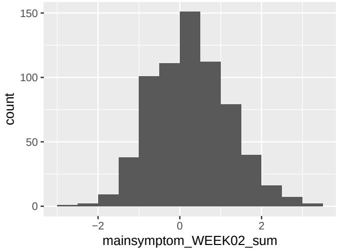

---

mainsymptom_WEEK03_sum

Feature	Result
Variable type	numeric
Number of missing obs.	0 (0 %)
Number of unique values	250
Median	0.19
1st and 3rd quartiles	-0.47; 0.94
Min. and max.	-2.4; 3.45

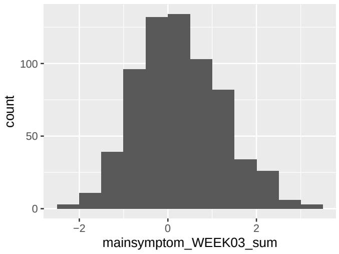

Report generation information:

- Created by: Could not determine from system (username: `nilisa`).
- Report creation time: Mon Jan 09 2023 13:10:32
- Report was run from directory: `/home/nilisa/projects/phd_study1/r`
- dataMaid v1.4.1 [Pkg: 2021-10-08 from CRAN (R 4.2.2)]
- R version 4.2.2 Patched (2022-11-10 r83330).
- Platform: x86_64-pc-linux-gnu (64-bit)(Ubuntu 20.04.5 LTS).
- Function call: `dataMaid::makeDataReport(data = gd, mode = c("summarize", "visualize",  "check"), smartNum = FALSE, file = "~/projects/data/study1multiverse/results/graphs_n_figures/codebooks/codeboo` `replace = TRUE, openResult = FALSE, checks = list(character = "showAllFactorLevels", factor = "showAllFactorLevels", labelled = "showAllFactorLevels",   haven_labelled = "showAllFactorLevels", numeric = NULL,   integer = NULL, logical = NULL, Date = NULL), listChecks = FALSE, maxProbVals = Inf, codebook = TRUE, reportTitle = "Handpicked_All_week04-imputed_`

Handpicked_All_week04-imputed_benchmark_train

Autogenerated data summary from dataMaid

2023-01-09 13:04:05

Data report overview

The dataset examined has the following dimensions:

Feature	Result
Number of observations	6026
Number of variables	10

Codebook summary table

Label	Variable	Class	# unique values	Missing	Description
	**sex**	factor	2	0.00 %	Sex of patient, 0 = Female, 1=Male
	**age**	numeric	72	0.00 %	
	**PDSS-SR-3064_SCREEN_sum**	numeric	410	0.00 %	Anxiety questionnaire, self rated-Timepoint before treatment starts-Sum of the entire measure
	**MADRS-1951_SCREEN_sum**	numeric	206	0.00 %	Depression questionnaire, self rated-Timepoint before treatment starts-Sum of the entire measure
	**LSAS-2241_SCREEN_sum**	numeric	734	0.00 %	Social anxiety questionnaire, self rated-Timepoint before treatment starts-Sum of the entire measure
	**outcome**	numeric	1639	0.00 %	
	**mainsymptom_PRE_sum**	numeric	431	0.00 %	PDSS-SR for panic, MADRS for depression, LSAS for social anxiety-Timepoint just before beginning treatment-Sum of the entire measure
	**mainsymptom_WEEK01_sum**	numeric	1299	0.00 %	PDSS-SR for panic, MADRS for depression, LSAS for social anxiety-Timepoint after one week in treatment-Sum of the entire measure
	**mainsymptom_WEEK02_sum**	numeric	1147	0.00 %	PDSS-SR for panic, MADRS for depression, LSAS for social anxiety-Timepoint after two weeks in treatment-Sum of the entire measure

Label	Variable	Class	# unique values	Missing	Description
	**mainsymptom_WEEK03_sum**	numeric	1247	0.00 %	PDSS-SR for panic, MADRS for depression, LSAS for social anxiety-Timepoint after three weeks in treatment-Sum of the entire measure

Variable list

sex

Feature	Result
Variable type	factor
Number of missing obs.	0 (0 %)
Number of unique values	2
Mode	“0”
Reference category	0

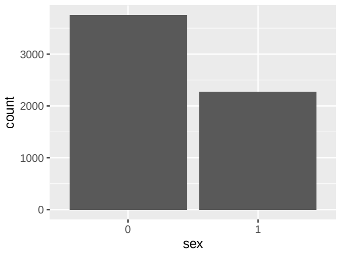

- Observed factor levels: "0", "1".

---

age

Feature	Result
Variable type	numeric
Number of missing obs.	0 (0 %)
Number of unique values	72
Median	-0.2
1st and 3rd quartiles	-0.73; 0.58
Min. and max.	-1.69; 4.25

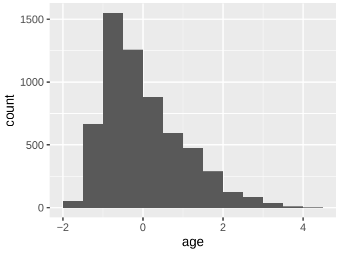

PDSS-SR-3064_SCREEN_sum

Feature	Result
Variable type	numeric
Number of missing obs.	0 (0 %)
Number of unique values	410
Median	-0.03
1st and 3rd quartiles	-0.82; 0.75
Min. and max.	-1.29; 3.12

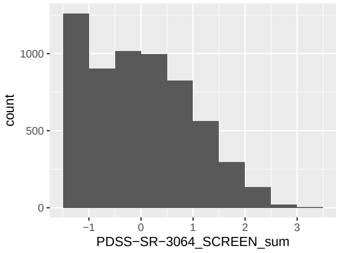

MADRS-1951_SCREEN_sum

Feature	Result
Variable type	numeric
Number of missing obs.	0 (0 %)
Number of unique values	206
Median	0.05
1st and 3rd quartiles	-0.7; 0.67
Min. and max.	-2.68; 3.4

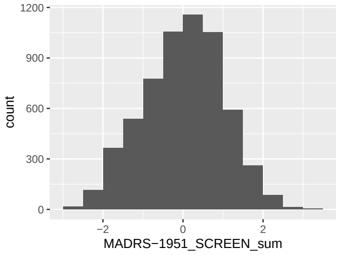

LSAS-2241_SCREEN_sum

Feature	Result
Variable type	numeric
Number of missing obs.	0 (0 %)
Number of unique values	734
Median	-0.09
1st and 3rd quartiles	-0.72; 0.61
Min. and max.	-1.79; 3.09

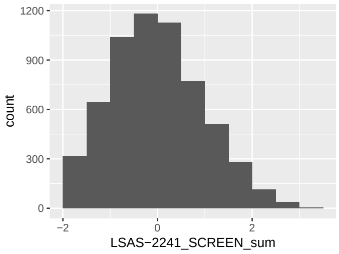

outcome

Feature	Result
Variable type	numeric
Number of missing obs.	0 (0 %)
Number of unique values	1639
Median	-0.09
1st and 3rd quartiles	-0.65; 0.62
Min. and max.	-2.05; 4.66

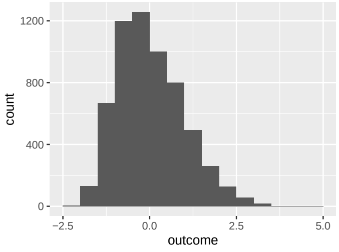

mainsymptom_PRE_sum

Feature	Result
Variable type	numeric
Number of missing obs.	0 (0 %)
Number of unique values	431
Median	0.27
1st and 3rd quartiles	-0.46; 0.89
Min. and max.	-2.7; 3.48

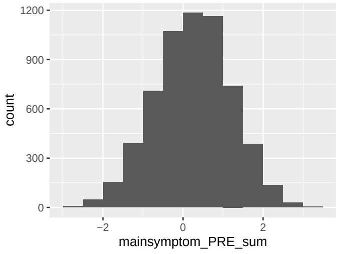

mainsymptom_WEEK01_sum

Feature	Result
Variable type	numeric
Number of missing obs.	0 (0 %)
Number of unique values	1299
Median	0.19
1st and 3rd quartiles	-0.44; 0.85
Min. and max.	-2.84; 3.6

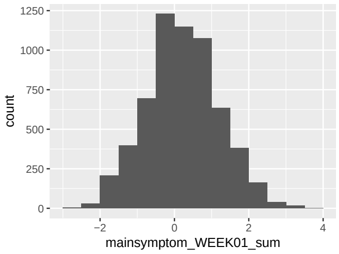

mainsymptom_WEEK02_sum

Feature	Result
Variable type	numeric
Number of missing obs.	0 (0 %)
Number of unique values	1147
Median	0.14
1st and 3rd quartiles	-0.46; 0.86
Min. and max.	-2.75; 4.09

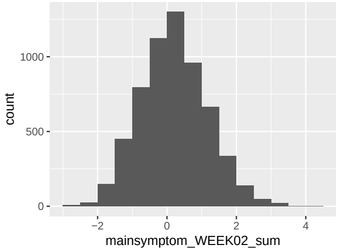

mainsymptom_WEEK03_sum

Feature	Result
Variable type	numeric
Number of missing obs.	0 (0 %)
Number of unique values	1247
Median	0.13
1st and 3rd quartiles	-0.47; 0.83
Min. and max.	-2.64; 4.64

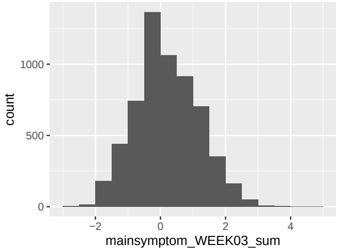

Report generation information:

- Created by: Could not determine from system (username: `nilisa`).
- Report creation time: Mon Jan 09 2023 13:04:05
- Report was run from directory: `/home/nilisa/projects/phd_study1/r`
- dataMaid v1.4.1 [Pkg: 2021-10-08 from CRAN (R 4.2.2)]
- R version 4.2.2 Patched (2022-11-10 r83330).
- Platform: x86_64-pc-linux-gnu (64-bit)(Ubuntu 20.04.5 LTS).
- Function call: `dataMaid::makeDataReport(data = gd, mode = c("summarize", "visualize", "check"), smartNum = FALSE, file = "~/projects/data/study1multiverse/results/graphs_n_figures/codebooks/codebook replace = TRUE, openResult = FALSE, checks = list(character = "showAllFactorLevels", factor = "showAllFactorLevels", labelled = "showAllFactorLevels",   haven_labelled = "showAllFactorLevels", numeric = NULL,   integer = NULL, logical = NULL, Date = NULL), listChecks = FALSE, maxProbVals = Inf, codebook = TRUE, reportTitle = "Handpicked_All_week04-imputed_`

Handpicked_All_week04-imputed_test

Autogenerated data summary from dataMaid

2023-01-09 13:10:40

Data report overview

The dataset examined has the following dimensions:

Feature	Result
Number of observations	669
Number of variables	66

Codebook summary table

Label	Variable	Class	# unique values	Missing	Description
	**sex**	factor	2	0.00 %	Sex of patient, 0 = Female, 1=Male
	**age**	numeric	56	0.00 %	
	**messages_len_7**	numeric	173	0.00 %	-Meta information of messages-Length of messages-up until day-7
	**messages_len_tp_7**	numeric	384	0.00 %	-Meta information of messages-Length of messages-therapist messages-up until day-7
	**messages_7**	numeric	6	0.00 %	-Meta information of messages-up until day-7
	**messages_tp_7**	numeric	7	0.00 %	-Meta information of messages-therapist messages-up until day-7
	**homeworks_7**	numeric	6	0.00 %	-Number of homework messages sent in-up until day-7
	**messages_len_14**	numeric	212	0.00 %	-Meta information of messages-Length of messages-up until day-14
	**messages_len_tp_14**	numeric	426	0.00 %	-Meta information of messages-Length of messages-therapist messages-up until day-14
	**messages_14**	numeric	7	0.00 %	-Meta information of messages-up until day-14
	**messages_tp_14**	numeric	6	0.00 %	-Meta information of messages-therapist messages-up until day-14

Label	Variable	Class	# unique values	Missing	Description
	**homeworks_14**	numeric	6	0.00 %	-Number of homework messages sent in-up until day-14
	**messages_len_21**	numeric	221	0.00 %	-Meta information of messages-Length of messages-up until day-21
	**messages_len_tp_21**	numeric	411	0.00 %	-Meta information of messages-Length of messages-therapist messages-up until day-21
	**messages_21**	numeric	7	0.00 %	-Meta information of messages-up until day-21
	**messages_tp_21**	numeric	5	0.00 %	-Meta information of messages-therapist messages-up until day-21
	**homeworks_21**	numeric	5	0.00 %	-Number of homework messages sent in-up until day-21
	**messages_len_28**	numeric	207	0.00 %	-Meta information of messages-Length of messages-up until day-28
	**messages_len_tp_28**	numeric	403	0.00 %	-Meta information of messages-Length of messages-therapist messages-up until day-28
	**messages_28**	numeric	6	0.00 %	-Meta information of messages-up until day-28
	**messages_tp_28**	numeric	5	0.00 %	-Meta information of messages-therapist messages-up until day-28
	**homeworks_28**	numeric	6	0.00 %	-Number of homework messages sent in-up until day-28
	**PDSS-SR-3064_SCREEN_sum**	numeric	79	0.00 %	Anxiety questionnaire, self rated-Timepoint before treatment starts-Sum of the entire measure
	**MADRS-1951_SCREEN_sum**	numeric	70	0.00 %	Depression questionnaire, self rated-Timepoint before treatment starts-Sum of the entire measure
	**LSAS-2241_SCREEN_sum**	numeric	201	0.00 %	Social anxiety questionnaire, self rated-Timepoint before treatment starts-Sum of the entire measure
	**MADRS-1951_SCREEN_DateCompleted_day**	numeric	20	0.00 %	Depression questionnaire, self rated-Timepoint before treatment starts-Cyclic transformation of what day 0-6 during week it was filled in

Label	Variable	Class	# unique values	Missing	Description
	**MADRS-1951_SCREEN_DateCompleted_time**	numeric	469	0.00 %	Depression questionnaire, self rated-Timepoint before treatment starts-Cyclic transformation of what time during day 0-1440 it was filled in
	**PDSS-SR-3064_SCREEN_DateCompleted_day**	numeric	25	0.00 %	Anxiety questionnaire, self rated-Timepoint before treatment starts-Cyclic transformation of what day 0-6 during week it was filled in
	**PDSS-SR-3064_SCREEN_DateCompleted_time**	numeric	496	0.00 %	Anxiety questionnaire, self rated-Timepoint before treatment starts-Cyclic transformation of what time during day 0-1440 it was filled in
	**LSAS-2241_SCREEN_DateCompleted_day**	numeric	28	0.00 %	Social anxiety questionnaire, self rated-Timepoint before treatment starts-Cyclic transformation of what day 0-6 during week it was filled in
	**LSAS-2241_SCREEN_DateCompleted_time**	numeric	494	0.00 %	Social anxiety questionnaire, self rated-Timepoint before treatment starts-Cyclic transformation of what time during day 0-1440 it was filled in
	**Depression**	numeric	2	0.00 %	
	**Panic**	numeric	2	0.00 %	
	**Social_Anxiety**	numeric	2	0.00 %	
	**outcome**	numeric	295	0.00 %	
	**ncomorbid**	numeric	36	0.00 %	
	**HW-01**	numeric	449	0.00 %	
	**HW-02**	numeric	505	0.00 %	
	**HW-03**	numeric	576	0.00 %	
	**currentwork_proff**	factor	28	0.00 %	Currently in work for trained proffession
	**Marital_1833_gift**	factor	2	0.00 %	Marital status: Married or not
	**Marital_1833_separerad**	factor	2	0.00 %	Marital status: divocered/equivalent
	**Marital_1833_singel**	factor	2	0.00 %	Marital status: single
	**Edu_1843_2**	factor	2	0.00 %	7-9 years education
	**Edu_1843_3**	factor	2	0.00 %	Uncompleted upper secondary school
	**Edu_1843_4**	factor	2	0.00 %	Higher vocational education
	**Edu_1843_5**	factor	2	0.00 %	Completed upper secondary school
	**Edu_1843_6**	factor	2	0.00 %	Uncompleted university degree
	**Edu_1843_7**	factor	2	0.00 %	University degree
	**cscale**	numeric	106	0.00 %	

Label	Variable	Class	# unique values	Missing	Description
	**mainsymptom_PRE_sum**	numeric	156	0.00 %	PDSS-SR for panic, MADRS for depression, LSAS for social anxiety-Timepoint just before beginning treatment-Sum of the entire measure
	**mainsymptom_PRE_duration**	numeric	559	0.00 %	PDSS-SR for panic, MADRS for depression, LSAS for social anxiety-Timepoint just before beginning treatment-Time to fill in measure/questionnaire
	**mainsymptom_PRE_DateCompleted_day**	numeric	30	0.00 %	PDSS-SR for panic, MADRS for depression, LSAS for social anxiety-Timepoint just before beginning treatment-Cyclic transformation of what day 0-6 during week it was filled in
	**mainsymptom_PRE_DateCompleted_time**	numeric	464	0.00 %	PDSS-SR for panic, MADRS for depression, LSAS for social anxiety-Timepoint just before beginning treatment-Cyclic transformation of what time during day 0-1440 it was filled in
	**mainsymptom_WEEK01_sum**	numeric	242	0.00 %	PDSS-SR for panic, MADRS for depression, LSAS for social anxiety-Timepoint after one week in treatment-Sum of the entire measure
	**mainsymptom_WEEK01_duration**	numeric	512	0.00 %	PDSS-SR for panic, MADRS for depression, LSAS for social anxiety-Timepoint after one week in treatment-Time to fill in measure/questionnaire
	**mainsymptom_WEEK01_DateCompleted_day**	numeric	112	0.00 %	PDSS-SR for panic, MADRS for depression, LSAS for social anxiety-Timepoint after one week in treatment-Cyclic transformation of what day 0-6 during week it was filled in
	**mainsymptom_WEEK01_DateCompleted_time**	numeric	506	0.00 %	PDSS-SR for panic, MADRS for depression, LSAS for social anxiety-Timepoint after one week in treatment-Cyclic transformation of what time during day 0-1440 it was filled in
	**mainsymptom_WEEK02_sum**	numeric	244	0.00 %	PDSS-SR for panic, MADRS for depression, LSAS for social anxiety-Timepoint after two weeks in treatment-Sum of the entire measure

Label	Variable	Class	# unique values	Missing	Description
	**mainsymptom_WEEK02_duration**	numeric	524	0.00 %	PDSS-SR for panic, MADRS for depression, LSAS for social anxiety-Timepoint after two weeks in treatment-Time to fill in measure/questionnaire
	**mainsymptom_WEEK02_DateCompleted_day**	numeric	116	0.00 %	PDSS-SR for panic, MADRS for depression, LSAS for social anxiety-Timepoint after two weeks in treatment-Cyclic transformation of what day 0-6 during week it was filled in
	**mainsymptom_WEEK02_DateCompleted_time**	numeric	522	0.00 %	PDSS-SR for panic, MADRS for depression, LSAS for social anxiety-Timepoint after two weeks in treatment-Cyclic transformation of what time during day 0-1440 it was filled in
	**mainsymptom_WEEK03_sum**	numeric	250	0.00 %	PDSS-SR for panic, MADRS for depression, LSAS for social anxiety-Timepoint after three weeks in treatment-Sum of the entire measure
	**mainsymptom_WEEK03_duration**	numeric	508	0.00 %	PDSS-SR for panic, MADRS for depression, LSAS for social anxiety-Timepoint after three weeks in treatment-Time to fill in measure/questionnaire
	**mainsymptom_WEEK03_DateCompleted_day**	numeric	119	0.00 %	PDSS-SR for panic, MADRS for depression, LSAS for social anxiety-Timepoint after three weeks in treatment-Cyclic transformation of what day 0-6 during week it was filled in
	**mainsymptom_WEEK03_DateCompleted_time**	numeric	518	0.00 %	PDSS-SR for panic, MADRS for depression, LSAS for social anxiety-Timepoint after three weeks in treatment-Cyclic transformation of what time during day 0-1440 it was filled in

Variable list

sex

Feature	Result
Variable type	factor
Number of missing obs.	0 (0 %)
Number of unique values	2
Mode	“0”
Reference category	0

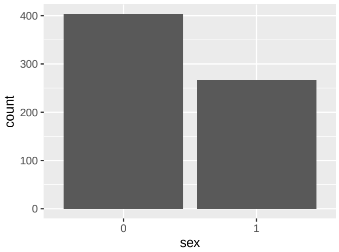

- Observed factor levels: "0", "1".

---

age

Feature	Result
Variable type	numeric
Number of missing obs.	0 (0 %)
Number of unique values	56
Median	-0.29
1st and 3rd quartiles	-0.81; 0.58
Min. and max.	-1.6; 3.73

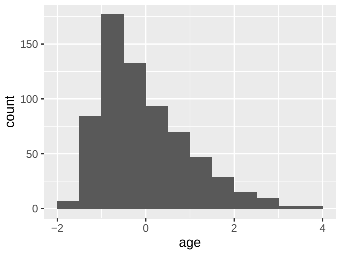

---

messages_len_7

Feature	Result
Variable type	numeric
Number of missing obs.	0 (0 %)
Number of unique values	173
Median	-0.32
1st and 3rd quartiles	-0.32; -0.05
Min. and max.	-0.32; 5.88

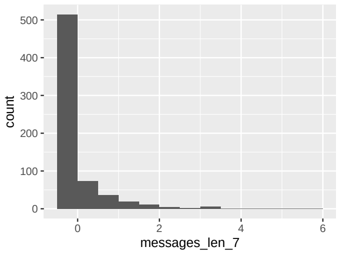

---

messages_len_tp_7

Feature	Result
Variable type	numeric
Number of missing obs.	0 (0 %)
Number of unique values	384
Median	-0.13
1st and 3rd quartiles	-0.95; 0.71
Min. and max.	-1.46; 4.91

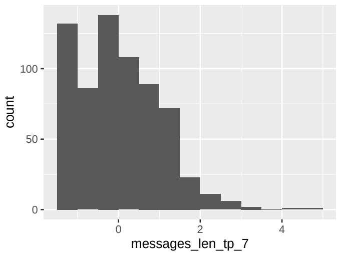

---

messages_7

Feature	Result
Variable type	numeric
Number of missing obs.	0 (0 %)
Number of unique values	6
Median	-0.55
1st and 3rd quartiles	-0.55; 0.58
Min. and max.	-0.55; 5.13

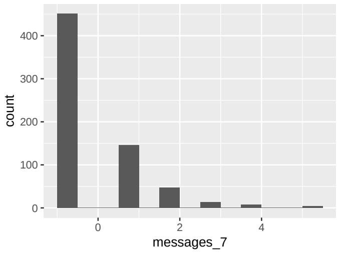

messages_tp_7

Feature	Result
Variable type	numeric
Number of missing obs.	0 (0 %)
Number of unique values	7
Median	0.15
1st and 3rd quartiles	-0.91; 0.15
Min. and max.	-1.97; 5.46

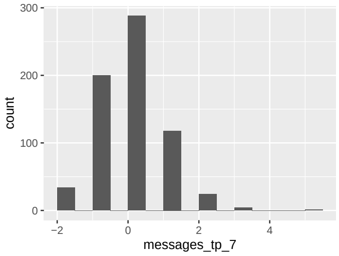

homeworks_7

Feature	Result
Variable type	numeric
Number of missing obs.	0 (0 %)
Number of unique values	6
Median	0.2
1st and 3rd quartiles	-1.03; 0.2
Min. and max.	-1.03; 5.15

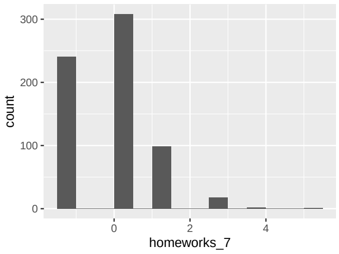

messages_len_14

Feature	Result
Variable type	numeric
Number of missing obs.	0 (0 %)
Number of unique values	212
Median	-0.41
1st and 3rd quartiles	-0.41; 0.07
Min. and max.	-0.41; 12.82

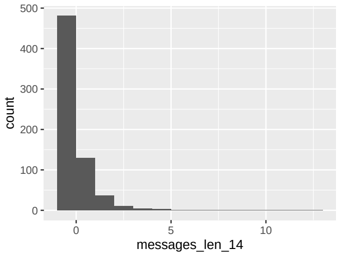

messages_len_tp_14

Feature	Result
Variable type	numeric
Number of missing obs.	0 (0 %)
Number of unique values	426
Median	-0.25
1st and 3rd quartiles	-0.78; 0.46
Min. and max.	-1.11; 4.12

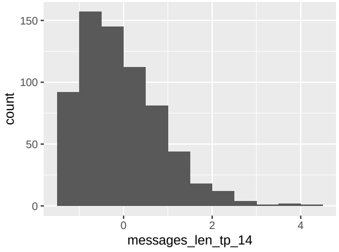

messages_14

Feature	Result
Variable type	numeric
Number of missing obs.	0 (0 %)
Number of unique values	7
Median	-0.7
1st and 3rd quartiles	-0.7; 0.36
Min. and max.	-0.7; 5.66

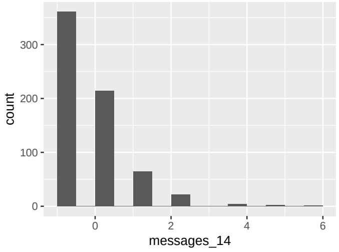

messages_tp_14

Feature	Result
Variable type	numeric
Number of missing obs.	0 (0 %)
Number of unique values	6
Median	-0.35
1st and 3rd quartiles	-0.35; 0.87
Min. and max.	-1.57; 6.97

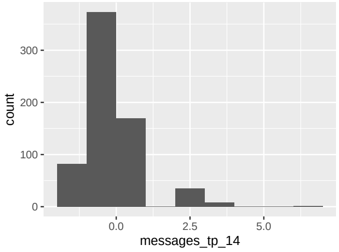

homeworks_14

Feature	Result
Variable type	numeric
Number of missing obs.	0 (0 %)
Number of unique values	6
Median	0.33
1st and 3rd quartiles	-0.98; 0.33
Min. and max.	-0.98; 14.78

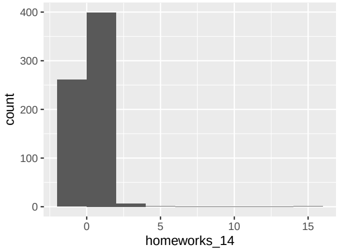

messages_len_21

Feature	Result
Variable type	numeric
Number of missing obs.	0 (0 %)
Number of unique values	221
Median	-0.46
1st and 3rd quartiles	-0.46; 0.14
Min. and max.	-0.46; 10.88

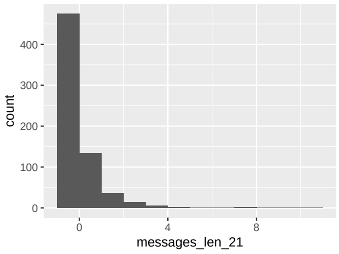

messages_len_tp_21

Feature	Result
Variable type	numeric
Number of missing obs.	0 (0 %)
Number of unique values	411
Median	-0.24
1st and 3rd quartiles	-0.82; 0.5
Min. and max.	-1.01; 4.87

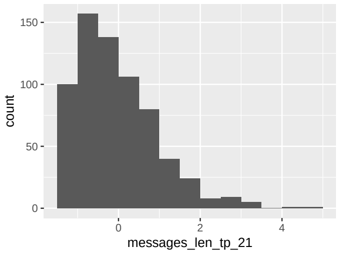

messages_21

Feature	Result
Variable type	numeric
Number of missing obs.	0 (0 %)
Number of unique values	7
Median	-0.74
1st and 3rd quartiles	-0.74; 0.34
Min. and max.	-0.74; 5.75

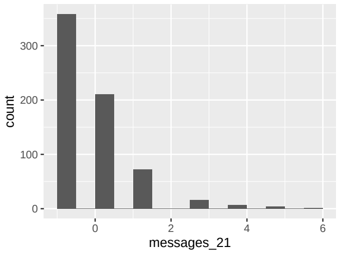

messages_tp_21

Feature	Result
Variable type	numeric
Number of missing obs.	0 (0 %)
Number of unique values	5
Median	-0.32
1st and 3rd quartiles	-0.32; 0.84
Min. and max.	-1.49; 3.17

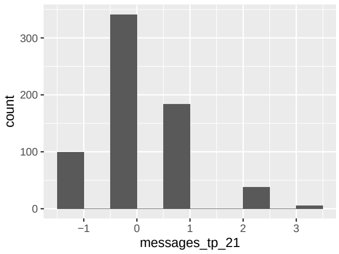

homeworks_21

Feature	Result
Variable type	numeric
Number of missing obs.	0 (0 %)
Number of unique values	5
Median	0.36
1st and 3rd quartiles	-1.01; 0.36
Min. and max.	-1.01; 4.49

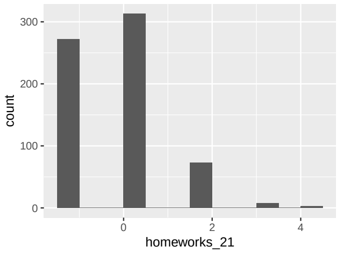

messages_len_28

Feature	Result
Variable type	numeric
Number of missing obs.	0 (0 %)
Number of unique values	207
Median	-0.41
1st and 3rd quartiles	-0.41; 0.06
Min. and max.	-0.41; 7.99

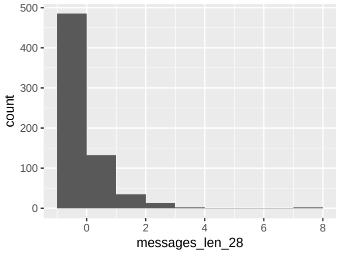

messages_len_tp_28

Feature	Result
Variable type	numeric
Number of missing obs.	0 (0 %)
Number of unique values	403
Median	-0.24
1st and 3rd quartiles	-0.76; 0.5
Min. and max.	-0.94; 6.1

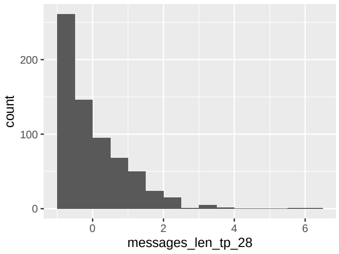

messages_28

Feature	Result
Variable type	numeric
Number of missing obs.	0 (0 %)
Number of unique values	6
Median	-0.72
1st and 3rd quartiles	-0.72; 0.41
Min. and max.	-0.72; 4.94

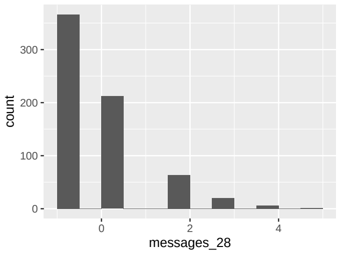

messages_tp_28

Feature	Result
Variable type	numeric
Number of missing obs.	0 (0 %)
Number of unique values	5
Median	-0.24
1st and 3rd quartiles	-0.24; 0.93
Min. and max.	-1.42; 3.28

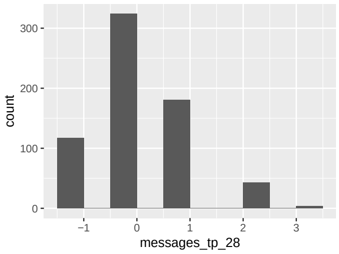

homeworks_28

Feature	Result
Variable type	numeric
Number of missing obs.	0 (0 %)
Number of unique values	6
Median	0.46
1st and 3rd quartiles	-0.91; 0.46
Min. and max.	-0.91; 5.94

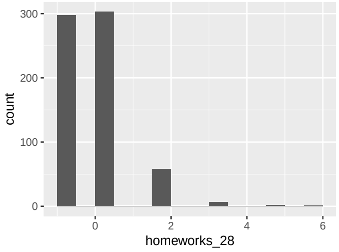

PDSS-SR-3064_SCREEN_sum

Feature	Result
Variable type	numeric
Number of missing obs.	0 (0 %)
Number of unique values	79
Median	-0.03
1st and 3rd quartiles	-0.85; 0.75
Min. and max.	-1.29; 2.96

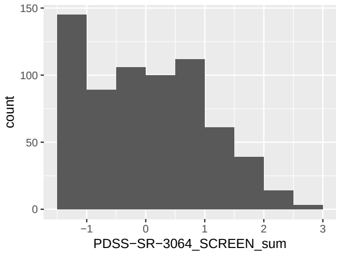

MADRS-1951_SCREEN_sum

Feature	Result
Variable type	numeric
Number of missing obs.	0 (0 %)
Number of unique values	70
Median	0.05
1st and 3rd quartiles	-0.57; 0.67
Min. and max.	-2.68; 3.02

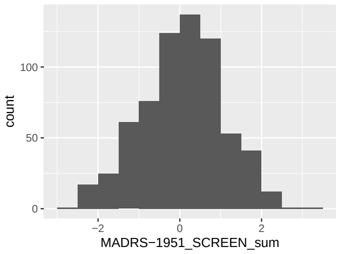

LSAS-2241_SCREEN_sum

Feature	Result
Variable type	numeric
Number of missing obs.	0 (0 %)
Number of unique values	201
Median	-0.13
1st and 3rd quartiles	-0.75; 0.74
Min. and max.	-1.79; 2.99

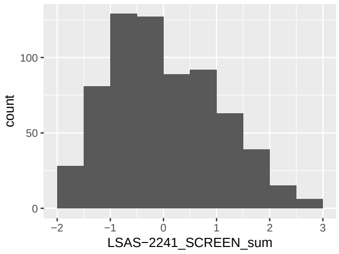

MADRS-1951_SCREEN_DateCompleted_day

Feature	Result
Variable type	numeric
Number of missing obs.	0 (0 %)
Number of unique values	20
Median	0.37
1st and 3rd quartiles	-0.37; 1
Min. and max.	-1.37; 1.37

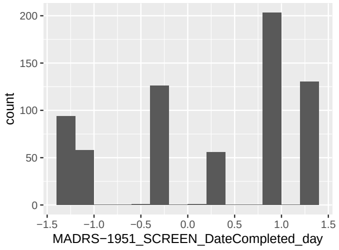

MADRS-1951_SCREEN_DateCompleted_time

Feature	Result
Variable type	numeric
Number of missing obs.	0 (0 %)
Number of unique values	469
Median	-0.78
1st and 3rd quartiles	-1.24; 0.1
Min. and max.	-1.41; 1.41

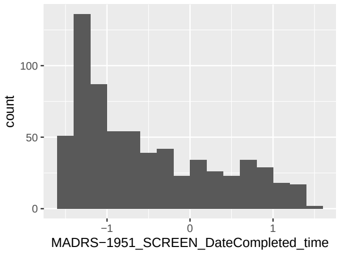

PDSS-SR-3064_SCREEN_DateCompleted_day

Feature	Result
Variable type	numeric
Number of missing obs.	0 (0 %)
Number of unique values	25
Median	0.37
1st and 3rd quartiles	-0.37; 1
Min. and max.	-1.37; 1.37

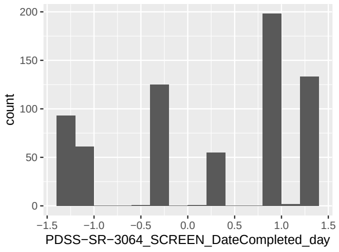

PDSS-SR-3064_SCREEN_DateCompleted_time

Feature	Result
Variable type	numeric
Number of missing obs.	0 (0 %)
Number of unique values	496
Median	-0.76
1st and 3rd quartiles	-1.22; 0.14
Min. and max.	-1.41; 1.41

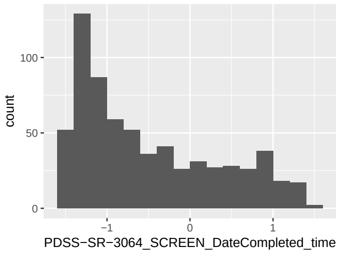

LSAS-2241_SCREEN_DateCompleted_day

Feature	Result
Variable type	numeric
Number of missing obs.	0 (0 %)
Number of unique values	28
Median	0.37
1st and 3rd quartiles	-0.37; 1
Min. and max.	-1.37; 1.37

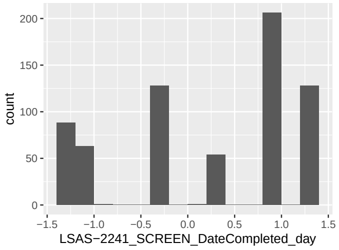

LSAS-2241_SCREEN_DateCompleted_time

Feature	Result
Variable type	numeric
Number of missing obs.	0 (0 %)
Number of unique values	494
Median	-0.78
1st and 3rd quartiles	-1.22; 0.17
Min. and max.	-1.41; 1.41

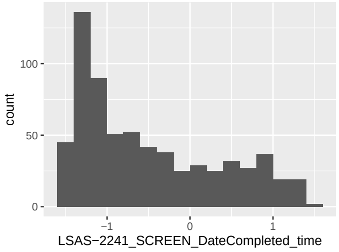

Depression

Feature	Result
Variable type	numeric
Number of missing obs.	0 (0 %)
Number of unique values	2
Median	0
1st and 3rd quartiles	0; 1
Min. and max.	0; 1

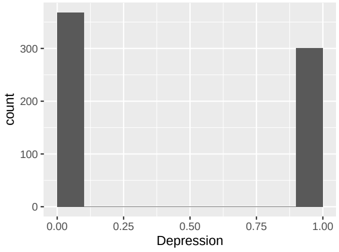

Panic

Feature	Result
Variable type	numeric
Number of missing obs.	0 (0 %)
Number of unique values	2
Median	0
1st and 3rd quartiles	0; 1
Min. and max.	0; 1

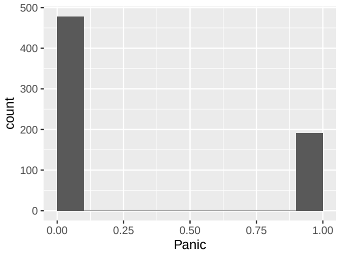

Social_Anxiety

Feature	Result
Variable type	numeric
Number of missing obs.	0 (0 %)
Number of unique values	2
Median	0
1st and 3rd quartiles	0; 1
Min. and max.	0; 1

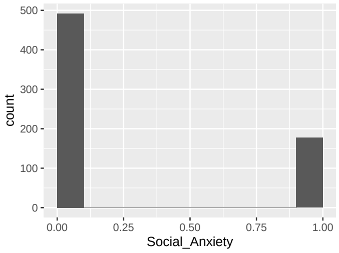

outcome

Feature	Result
Variable type	numeric
Number of missing obs.	0 (0 %)
Number of unique values	295
Median	0.17
1st and 3rd quartiles	-0.56; 0.73
Min. and max.	-2.05; 3.53

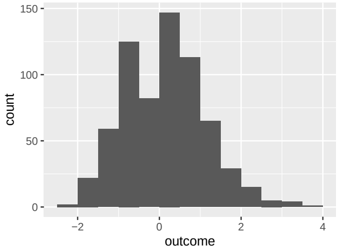

ncomorbid

Feature	Result
Variable type	numeric
Number of missing obs.	0 (0 %)
Number of unique values	36
Median	0
1st and 3rd quartiles	0; 1
Min. and max.	0; 4

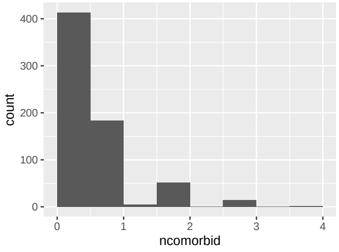

HW-01

Feature	Result
Variable type	numeric
Number of missing obs.	0 (0 %)
Number of unique values	449
Median	-0.08
1st and 3rd quartiles	-0.68; 0.54
Min. and max.	-4.03; 2.35

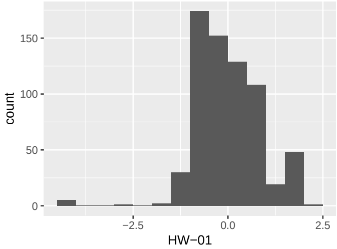

HW-02

Feature	Result
Variable type	numeric
Number of missing obs.	0 (0 %)
Number of unique values	505
Median	-0.31
1st and 3rd quartiles	-0.61; 0
Min. and max.	-1.13; 4.65

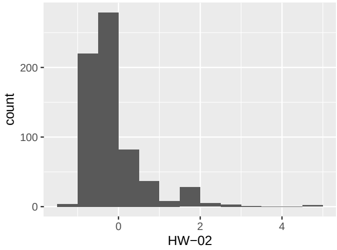

HW-03

Feature	Result
Variable type	numeric
Number of missing obs.	0 (0 %)
Number of unique values	576
Median	-0.11
1st and 3rd quartiles	-0.35; 0.07
Min. and max.	-4.83; 2.04

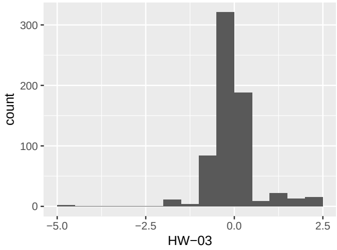

currentwork_proff

Feature	Result
Variable type	factor
Number of missing obs.	0 (0 %)
Number of unique values	28
Mode	"1"
Reference category	0

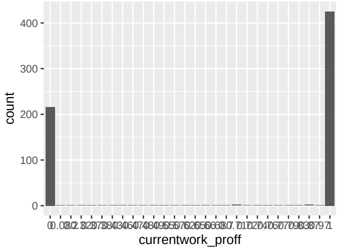

- Observed factor levels: "0", "0.08", "0.2", "0.32", "0.37", "0.38", "0.43", "0.46", "0.47", "0.48", "0.49", "0.55", "0.57", "0.62", "0.65", "0.66", "0.68", "0.7", "0.71", "0.72", "0.74", "0.76", "0.77", "0.79", "0.83", "0.87", "0.97", "1".

Marital_1833_gift

Feature	Result
Variable type	factor
Number of missing obs.	0 (0 %)
Number of unique values	2
Mode	"1"
Reference category	0

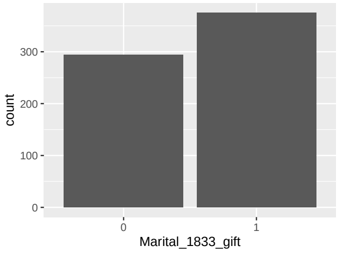

- Observed factor levels: "0", "1".

Marital_1833_separerad

Feature	Result
Variable type	factor
Number of missing obs.	0 (0 %)
Number of unique values	2
Mode	"0"
Reference category	0

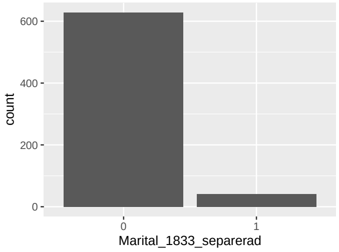

- Observed factor levels: "0", "1".

Marital_1833_singel

Feature	Result
Variable type	factor
Number of missing obs.	0 (0 %)
Number of unique values	2
Mode	"0"
Reference category	0

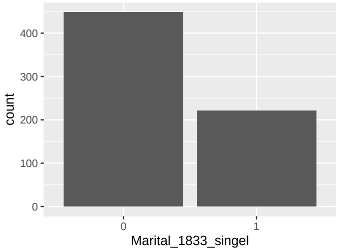

- Observed factor levels: "0", "1".

Edu_1843_2

Feature	Result
Variable type	factor
Number of missing obs.	0 (0 %)
Number of unique values	2
Mode	"0"
Reference category	0

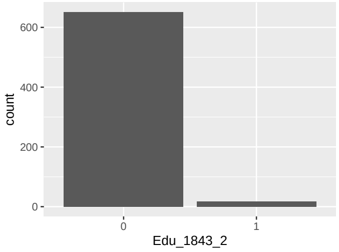

- Observed factor levels: "0", "1".

Edu_1843_3

Feature	Result
Variable type	factor
Number of missing obs.	0 (0 %)
Number of unique values	2
Mode	“0”
Reference category	0

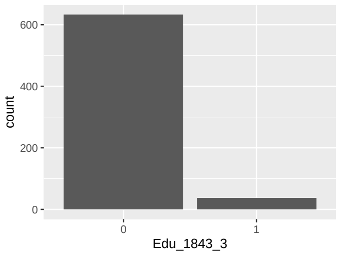

- Observed factor levels: "0", "1".

Edu_1843_4

Feature	Result
Variable type	factor
Number of missing obs.	0 (0 %)
Number of unique values	2
Mode	“0”
Reference category	0

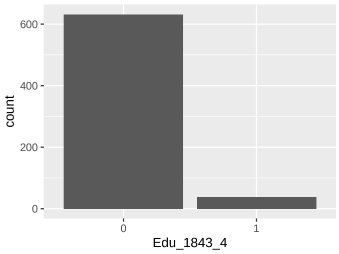

- Observed factor levels: "0", "1".

Edu_1843_5

Feature	Result
Variable type	factor
Number of missing obs.	0 (0 %)
Number of unique values	2
Mode	“0”
Reference category	0

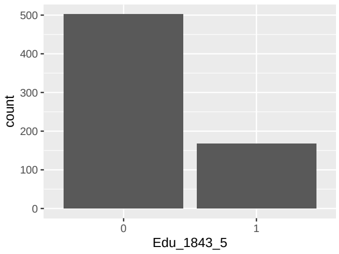

- Observed factor levels: "0", "1".

Edu_1843_6

Feature	Result
Variable type	factor
Number of missing obs.	0 (0 %)
Number of unique values	2
Mode	“0”
Reference category	0

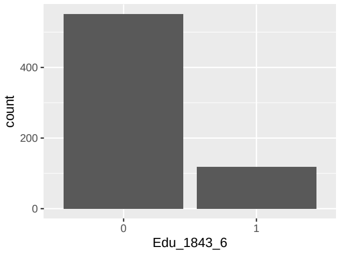

- Observed factor levels: "0", "1".

Edu_1843_7

Feature	Result
Variable type	factor
Number of missing obs.	0 (0 %)
Number of unique values	2
Mode	“0”
Reference category	0

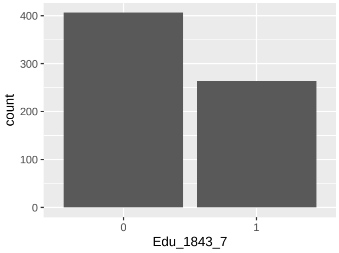

- Observed factor levels: "0", "1".

---

cscale

Feature	Result
Variable type	numeric
Number of missing obs.	0 (0 %)
Number of unique values	106
Median	0.02
1st and 3rd quartiles	-0.58; 0.6
Min. and max.	-4.14; 1.79

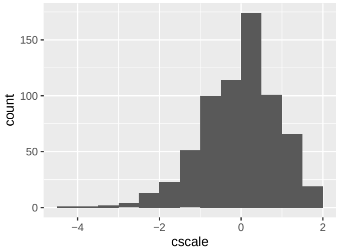

---

mainsymptom_PRE_sum

Feature	Result
Variable type	numeric
Number of missing obs.	0 (0 %)
Number of unique values	156
Median	0.34
1st and 3rd quartiles	-0.28; 0.89
Min. and max.	-2.62; 2.87

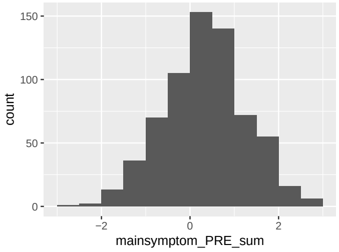

---

mainsymptom_PRE_duration

Feature	Result
Variable type	numeric
Number of missing obs.	0 (0 %)
Number of unique values	559
Median	-0.13
1st and 3rd quartiles	-0.2; -0.01
Min. and max.	-0.37; 7.79

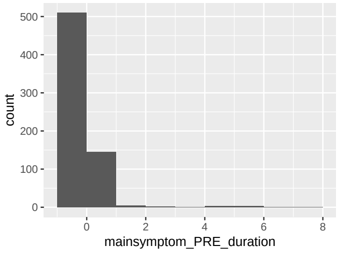

---

mainsymptom_PRE_DateCompleted_day

Feature	Result
Variable type	numeric
Number of missing obs.	0 (0 %)
Number of unique values	30
Median	0.37
1st and 3rd quartiles	-0.37; 1
Min. and max.	-1.37; 1.37

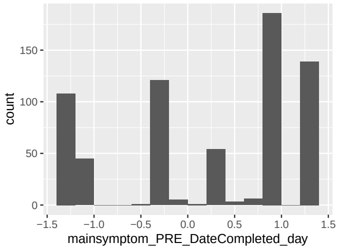

---

mainsymptom_PRE_DateCompleted_time

Feature	Result
Variable type	numeric
Number of missing obs.	0 (0 %)
Number of unique values	464
Median	-0.72
1st and 3rd quartiles	-1.26; 0.02
Min. and max.	-1.41; 1.41

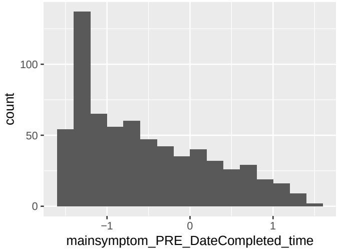

---

mainsymptom_WEEK01_sum

Feature	Result
Variable type	numeric
Number of missing obs.	0 (0 %)
Number of unique values	242
Median	0.28
1st and 3rd quartiles	-0.3; 0.9
Min. and max.	-2.84; 2.99

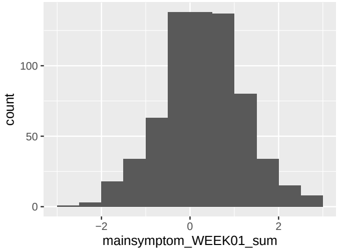

---

mainsymptom_WEEK01_duration

Feature	Result
Variable type	numeric
Number of missing obs.	0 (0 %)
Number of unique values	512
Median	-0.1
1st and 3rd quartiles	-0.17; 0
Min. and max.	-0.62; 7.09

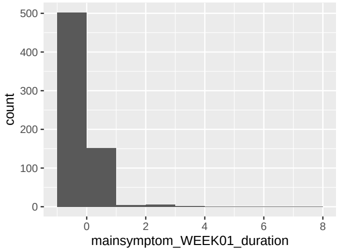

---

mainsymptom_WEEK01_DateCompleted_day

Feature	Result
Variable type	numeric
Number of missing obs.	0 (0 %)
Number of unique values	112
Median	0.4
1st and 3rd quartiles	-0.37; 1
Min. and max.	-1.37; 1.37

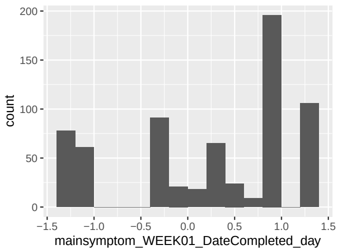

mainsymptom_WEEK01_DateCompleted_time

Feature	Result
Variable type	numeric
Number of missing obs.	0 (0 %)
Number of unique values	506
Median	-0.67
1st and 3rd quartiles	-1.13; 0
Min. and max.	-1.41; 1.41

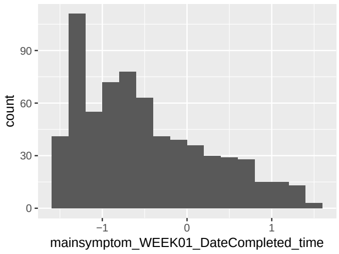

mainsymptom_WEEK02_sum

Feature	Result
Variable type	numeric
Number of missing obs.	0 (0 %)
Number of unique values	244
Median	0.25
1st and 3rd quartiles	-0.34; 0.92
Min. and max.	-2.75; 3.13

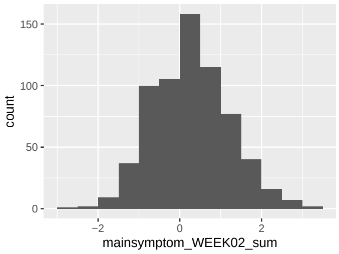

mainsymptom_WEEK02_duration

Feature	Result
Variable type	numeric
Number of missing obs.	0 (0 %)
Number of unique values	524
Median	-0.09
1st and 3rd quartiles	-0.17; 0.01
Min. and max.	-0.34; 4.45

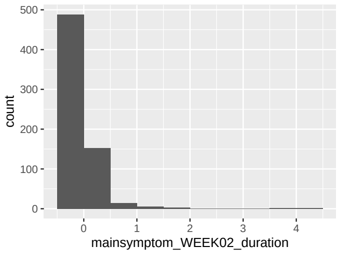

mainsymptom_WEEK02_DateCompleted_day

Feature	Result
Variable type	numeric
Number of missing obs.	0 (0 %)
Number of unique values	116
Median	0.39
1st and 3rd quartiles	-0.37; 1
Min. and max.	-1.37; 1.37

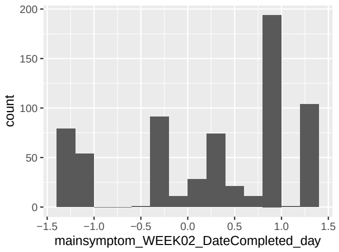

mainsymptom_WEEK02_DateCompleted_time

Feature	Result
Variable type	numeric
Number of missing obs.	0 (0 %)
Number of unique values	522
Median	-0.64
1st and 3rd quartiles	-1.12; -0.01
Min. and max.	-1.41; 1.41

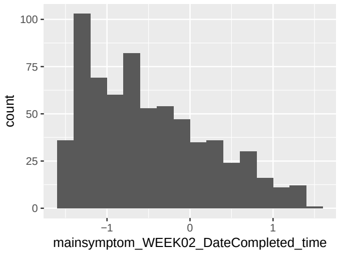

mainsymptom_WEEK03_sum

Feature	Result
Variable type	numeric
Number of missing obs.	0 (0 %)
Number of unique values	250
Median	0.19
1st and 3rd quartiles	-0.47; 0.92
Min. and max.	-2.4; 3.45

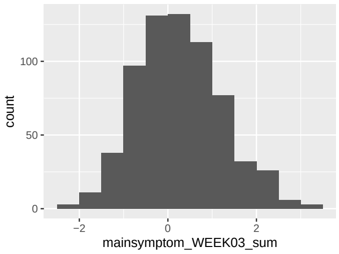

mainsymptom_WEEK03_duration

Feature	Result
Variable type	numeric
Number of missing obs.	0 (0 %)
Number of unique values	508
Median	-0.09
1st and 3rd quartiles	-0.14; -0.03
Min. and max.	-0.25; 4.23

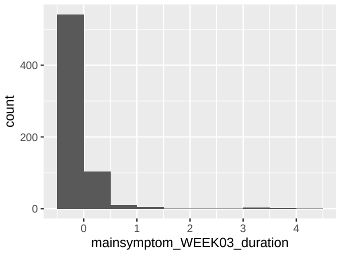

mainsymptom_WEEK03_DateCompleted_day

Feature	Result
Variable type	numeric
Number of missing obs.	0 (0 %)
Number of unique values	119
Median	0.37
1st and 3rd quartiles	-0.37; 1
Min. and max.	-1.37; 1.37

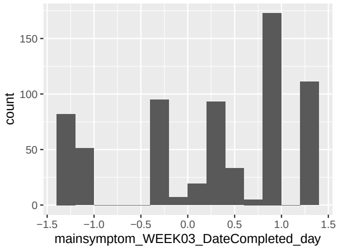

mainsymptom_WEEK03_DateCompleted_time

Feature	Result
Variable type	numeric
Number of missing obs.	0 (0 %)
Number of unique values	518
Median	-0.64
1st and 3rd quartiles	-1.13; 0.01
Min. and max.	-1.41; 1.41

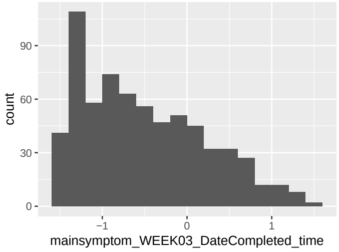

Report generation information:

- Created by: Could not determine from system (username: `nilisa`).
- Report creation time: Mon Jan 09 2023 13:10:41
- Report was run from directory: `/home/nilisa/projects/phd_study1/r`
- dataMaid v1.4.1 [Pkg: 2021-10-08 from CRAN (R 4.2.2)]
- R version 4.2.2 Patched (2022-11-10 r83330).
- Platform: x86_64-pc-linux-gnu (64-bit)(Ubuntu 20.04.5 LTS).
- Function call: `dataMaid::makeDataReport(data = gd, mode = c("summarize", "visualize", "check"), smartNum = FALSE, file = "~/projects/data/study1multiverse/results/graphs_n_figures/codebooks/codebook replace = TRUE, openResult = FALSE, checks = list(character = "showAllFactorLevels", factor = "showAllFactorLevels", labelled = "showAllFactorLevels",   haven_labelled = "showAllFactorLevels", numeric = NULL,   integer = NULL, logical = NULL, Date = NULL), listChecks = FALSE, maxProbVals = Inf, codebook = TRUE, reportTitle = "Handpicked_All_week04-imputed_`

Handpicked_All_week04-imputed_train

Autogenerated data summary from dataMaid

2023-01-09 13:04:16

Data report overview

The dataset examined has the following dimensions:

Feature	Result
Number of observations	6026
Number of variables	66

Codebook summary table

Label	Variable	Class	# unique values	Missing	Description
	**sex**	factor	2	0.00 %	Sex of patient, 0 = Female, 1=Male
	**age**	numeric	72	0.00 %	
	**messages_len_7**	numeric	633	0.00 %	-Meta information of messages-Length of messages-up until day-7
	**messages_len_tp_7**	numeric	1247	0.00 %	-Meta information of messages-Length of messages-therapist messages-up until day-7
	**messages_7**	numeric	11	0.00 %	-Meta information of messages-up until day-7
	**messages_tp_7**	numeric	9	0.00 %	-Meta information of messages-therapist messages-up until day-7
	**homeworks_7**	numeric	7	0.00 %	-Number of homework messages sent in-up until day-7
	**messages_len_14**	numeric	679	0.00 %	-Meta information of messages-Length of messages-up until day-14
	**messages_len_tp_14**	numeric	1143	0.00 %	-Meta information of messages-Length of messages-therapist messages-up until day-14
	**messages_14**	numeric	12	0.00 %	-Meta information of messages-up until day-14
	**messages_tp_14**	numeric	8	0.00 %	-Meta information of messages-therapist messages-up until day-14

Label	Variable	Class	# unique values	Missing	Description
	**homeworks_14**	numeric	7	0.00 %	-Number of homework messages sent in-up until day-14
	**messages_len_21**	numeric	662	0.00 %	-Meta information of messages-Length of messages-up until day-21
	**messages_len_tp_21**	numeric	1124	0.00 %	-Meta information of messages-Length of messages-therapist messages-up until day-21
	**messages_21**	numeric	10	0.00 %	-Meta information of messages-up until day-21
	**messages_tp_21**	numeric	8	0.00 %	-Meta information of messages-therapist messages-up until day-21
	**homeworks_21**	numeric	7	0.00 %	-Number of homework messages sent in-up until day-21
	**messages_len_28**	numeric	623	0.00 %	-Meta information of messages-Length of messages-up until day-28
	**messages_len_tp_28**	numeric	1080	0.00 %	-Meta information of messages-Length of messages-therapist messages-up until day-28
	**messages_28**	numeric	9	0.00 %	-Meta information of messages-up until day-28
	**messages_tp_28**	numeric	8	0.00 %	-Meta information of messages-therapist messages-up until day-28
	**homeworks_28**	numeric	8	0.00 %	-Number of homework messages sent in-up until day-28
	**PDSS-SR-3064_SCREEN_sum**	numeric	423	0.00 %	Anxiety questionnaire, self rated-Timepoint before treatment starts-Sum of the entire measure
	**MADRS-1951_SCREEN_sum**	numeric	207	0.00 %	Depression questionnaire, self rated-Timepoint before treatment starts-Sum of the entire measure
	**LSAS-2241_SCREEN_sum**	numeric	739	0.00 %	Social anxiety questionnaire, self rated-Timepoint before treatment starts-Sum of the entire measure
	**MADRS-1951_SCREEN_DateCompleted_day**	numeric	54	0.00 %	Depression questionnaire, self rated-Timepoint before treatment starts-Cyclic transformation of what day 0-6 during week it was filled in

Label	Variable	Class	# unique values	Missing	Description
	**MADRS-1951_SCREEN_DateCompleted_time**	numeric	1171	0.00 %	Depression questionnaire, self rated-Timepoint before treatment starts-Cyclic transformation of what time during day 0-1440 it was filled in
	**PDSS-SR-3064_SCREEN_DateCompleted_day**	numeric	193	0.00 %	Anxiety questionnaire, self rated-Timepoint before treatment starts-Cyclic transformation of what day 0-6 during week it was filled in
	**PDSS-SR-3064_SCREEN_DateCompleted_time**	numeric	1411	0.00 %	Anxiety questionnaire, self rated-Timepoint before treatment starts-Cyclic transformation of what time during day 0-1440 it was filled in
	**LSAS-2241_SCREEN_DateCompleted_day**	numeric	159	0.00 %	Social anxiety questionnaire, self rated-Timepoint before treatment starts-Cyclic transformation of what day 0-6 during week it was filled in
	**LSAS-2241_SCREEN_DateCompleted_time**	numeric	1607	0.00 %	Social anxiety questionnaire, self rated-Timepoint before treatment starts-Cyclic transformation of what time during day 0-1440 it was filled in
	**Depression**	numeric	2	0.00 %	
	**Panic**	numeric	2	0.00 %	
	**Social_Anxiety**	numeric	2	0.00 %	
	**outcome**	numeric	1640	0.00 %	
	**ncomorbid**	numeric	118	0.00 %	
	**HW-01**	numeric	3997	0.00 %	
	**HW-02**	numeric	4230	0.00 %	
	**HW-03**	numeric	5152	0.00 %	
	**currentwork_proff**	factor	63	0.00 %	Currently in work for trained proffession
	**Marital_1833_gift**	factor	2	0.00 %	Marital status: Married or not
	**Marital_1833_separerad**	factor	2	0.00 %	Marital status: divocered/equivalent
	**Marital_1833_singel**	factor	2	0.00 %	Marital status: single
	**Edu_1843_2**	factor	2	0.00 %	7-9 years education
	**Edu_1843_3**	factor	2	0.00 %	Uncompleted upper secondary school
	**Edu_1843_4**	factor	2	0.00 %	Higher vocational education
	**Edu_1843_5**	factor	2	0.00 %	Completed upper secondary school
	**Edu_1843_6**	factor	2	0.00 %	Uncompleted university degree
	**Edu_1843_7**	factor	2	0.00 %	University degree
	**cscale**	numeric	642	0.00 %	

Label	Variable	Class	# unique values	Missing	Description
	**mainsymptom_PRE_sum**	numeric	432	0.00 %	PDSS-SR for panic, MADRS for depression, LSAS for social anxiety-Timepoint just before beginning treatment-Sum of the entire measure
	**mainsymptom_PRE_duration**	numeric	2483	0.00 %	PDSS-SR for panic, MADRS for depression, LSAS for social anxiety-Timepoint just before beginning treatment-Time to fill in measure/questionnaire
	**mainsymptom_PRE_DateCompleted_day**	numeric	238	0.00 %	PDSS-SR for panic, MADRS for depression, LSAS for social anxiety-Timepoint just before beginning treatment-Cyclic transformation of what day 0-6 during week it was filled in
	**mainsymptom_PRE_DateCompleted_time**	numeric	1215	0.00 %	PDSS-SR for panic, MADRS for depression, LSAS for social anxiety-Timepoint just before beginning treatment-Cyclic transformation of what time during day 0-1440 it was filled in
	**mainsymptom_WEEK01_sum**	numeric	1300	0.00 %	PDSS-SR for panic, MADRS for depression, LSAS for social anxiety-Timepoint after one week in treatment-Sum of the entire measure
	**mainsymptom_WEEK01_duration**	numeric	2498	0.00 %	PDSS-SR for panic, MADRS for depression, LSAS for social anxiety-Timepoint after one week in treatment-Time to fill in measure/questionnaire
	**mainsymptom_WEEK01_DateCompleted_day**	numeric	1046	0.00 %	PDSS-SR for panic, MADRS for depression, LSAS for social anxiety-Timepoint after one week in treatment-Cyclic transformation of what day 0-6 during week it was filled in
	**mainsymptom_WEEK01_DateCompleted_time**	numeric	2109	0.00 %	PDSS-SR for panic, MADRS for depression, LSAS for social anxiety-Timepoint after one week in treatment-Cyclic transformation of what time during day 0-1440 it was filled in
	**mainsymptom_WEEK02_sum**	numeric	1148	0.00 %	PDSS-SR for panic, MADRS for depression, LSAS for social anxiety-Timepoint after two weeks in treatment-Sum of the entire measure

Label	Variable	Class	# unique values	Missing	Description
	**mainsymptom_WEEK02_duration**	numeric	2291	0.00 %	PDSS-SR for panic, MADRS for depression, LSAS for social anxiety-Timepoint after two weeks in treatment-Time to fill in measure/questionnaire
	**mainsymptom_WEEK02_DateCompleted_day**	numeric	926	0.00 %	PDSS-SR for panic, MADRS for depression, LSAS for social anxiety-Timepoint after two weeks in treatment-Cyclic transformation of what day 0-6 during week it was filled in
	**mainsymptom_WEEK02_DateCompleted_time**	numeric	1969	0.00 %	PDSS-SR for panic, MADRS for depression, LSAS for social anxiety-Timepoint after two weeks in treatment-Cyclic transformation of what time during day 0-1440 it was filled in
	**mainsymptom_WEEK03_sum**	numeric	1248	0.00 %	PDSS-SR for panic, MADRS for depression, LSAS for social anxiety-Timepoint after three weeks in treatment-Sum of the entire measure
	**mainsymptom_WEEK03_duration**	numeric	2351	0.00 %	PDSS-SR for panic, MADRS for depression, LSAS for social anxiety-Timepoint after three weeks in treatment-Time to fill in measure/questionnaire
	**mainsymptom_WEEK03_DateCompleted_day**	numeric	1028	0.00 %	PDSS-SR for panic, MADRS for depression, LSAS for social anxiety-Timepoint after three weeks in treatment-Cyclic transformation of what day 0-6 during week it was filled in
	**mainsymptom_WEEK03_DateCompleted_time**	numeric	2071	0.00 %	PDSS-SR for panic, MADRS for depression, LSAS for social anxiety-Timepoint after three weeks in treatment-Cyclic transformation of what time during day 0-1440 it was filled in

Variable list

sex

Feature	Result
Variable type	factor
Number of missing obs.	0 (0 %)
Number of unique values	2
Mode	“0”
Reference category	0

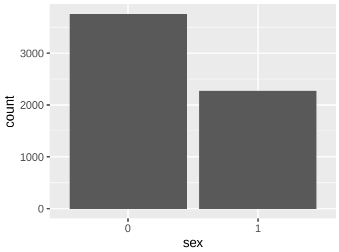

- Observed factor levels: "0", "1".

---

age

Feature	Result
Variable type	numeric
Number of missing obs.	0 (0 %)
Number of unique values	72
Median	-0.2
1st and 3rd quartiles	-0.73; 0.58
Min. and max.	-1.69; 4.25

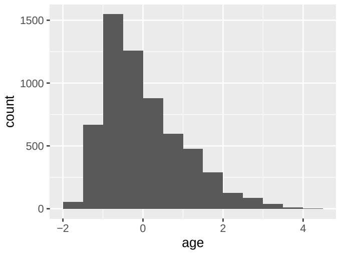

---

messages_len_7

Feature	Result
Variable type	numeric
Number of missing obs.	0 (0 %)
Number of unique values	633
Median	-0.32
1st and 3rd quartiles	-0.32; -0.08
Min. and max.	-0.32; 35.05

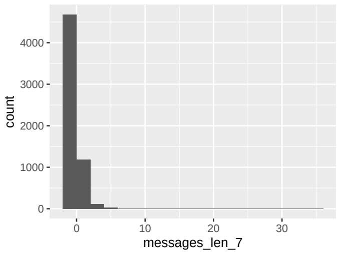

---

messages_len_tp_7

Feature	Result
Variable type	numeric
Number of missing obs.	0 (0 %)
Number of unique values	1247
Median	-0.16
1st and 3rd quartiles	-0.95; 0.67
Min. and max.	-1.46; 9.81

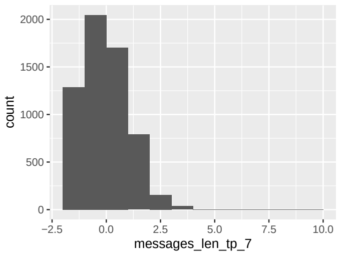

---

messages_7

Feature	Result
Variable type	numeric
Number of missing obs.	0 (0 %)
Number of unique values	11
Median	-0.55
1st and 3rd quartiles	-0.55; 0.58
Min. and max.	-0.55; 16.49

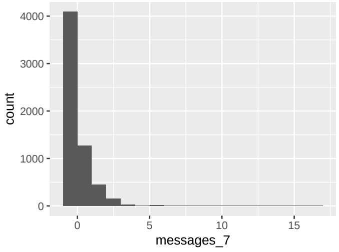

messages_tp_7

Feature	Result
Variable type	numeric
Number of missing obs.	0 (0 %)
Number of unique values	9
Median	0.15
1st and 3rd quartiles	-0.91; 0.15
Min. and max.	-1.97; 6.52

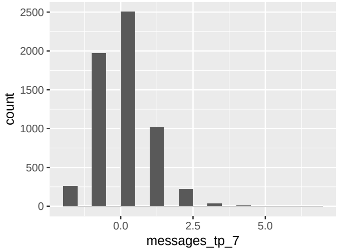

homeworks_7

Feature	Result
Variable type	numeric
Number of missing obs.	0 (0 %)
Number of unique values	7
Median	0.2
1st and 3rd quartiles	-1.03; 0.2
Min. and max.	-1.03; 6.39

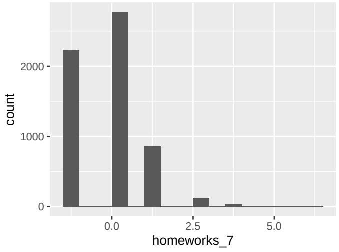

messages_len_14

Feature	Result
Variable type	numeric
Number of missing obs.	0 (0 %)
Number of unique values	679
Median	-0.41
1st and 3rd quartiles	-0.41; 0.03
Min. and max.	-0.41; 34.19

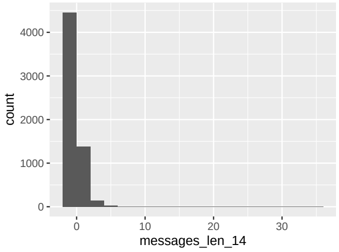

messages_len_tp_14

Feature	Result
Variable type	numeric
Number of missing obs.	0 (0 %)
Number of unique values	1143
Median	-0.16
1st and 3rd quartiles	-0.77; 0.52
Min. and max.	-1.11; 14.98

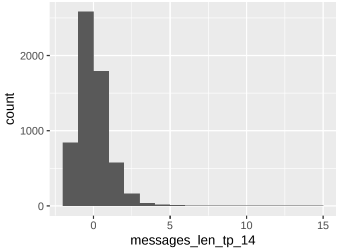

messages_14

Feature	Result
Variable type	numeric
Number of missing obs.	0 (0 %)
Number of unique values	12
Median	-0.7
1st and 3rd quartiles	-0.7; 0.36
Min. and max.	-0.7; 12.02

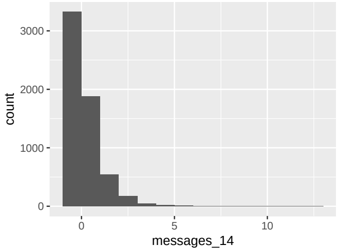

messages_tp_14

Feature	Result
Variable type	numeric
Number of missing obs.	0 (0 %)
Number of unique values	8
Median	-0.35
1st and 3rd quartiles	-0.35; 0.87
Min. and max.	-1.57; 8.19

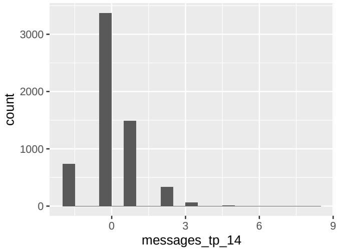

homeworks_14

Feature	Result
Variable type	numeric
Number of missing obs.	0 (0 %)
Number of unique values	7
Median	0.33
1st and 3rd quartiles	-0.98; 0.33
Min. and max.	-0.98; 8.21

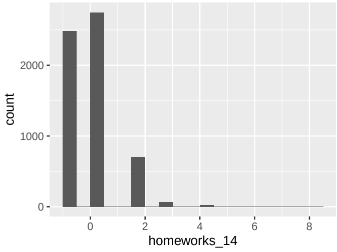

messages_len_21

Feature	Result
Variable type	numeric
Number of missing obs.	0 (0 %)
Number of unique values	662
Median	-0.46
1st and 3rd quartiles	-0.46; 0.07
Min. and max.	-0.46; 18.45

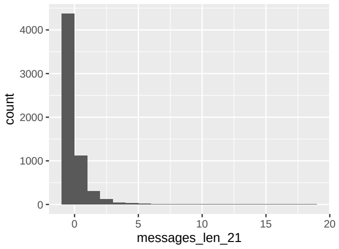

messages_len_tp_21

Feature	Result
Variable type	numeric
Number of missing obs.	0 (0 %)
Number of unique values	1124
Median	-0.22
1st and 3rd quartiles	-0.79; 0.49
Min. and max.	-1.01; 8.85

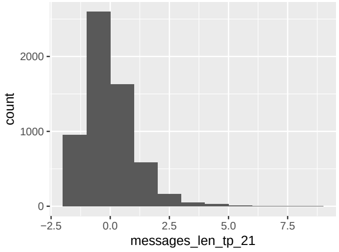

messages_21

Feature	Result
Variable type	numeric
Number of missing obs.	0 (0 %)
Number of unique values	10
Median	-0.74
1st and 3rd quartiles	-0.74; 0.34
Min. and max.	-0.74; 8.99

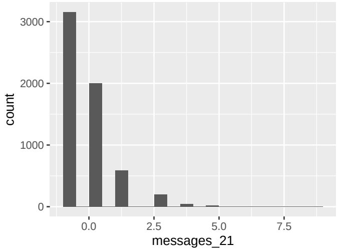

messages_tp_21

Feature	Result
Variable type	numeric
Number of missing obs.	0 (0 %)
Number of unique values	8
Median	-0.32
1st and 3rd quartiles	-0.32; 0.84
Min. and max.	-1.49; 11.31

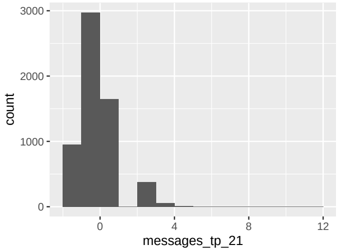

homeworks_21

Feature	Result
Variable type	numeric
Number of missing obs.	0 (0 %)
Number of unique values	7
Median	0.36
1st and 3rd quartiles	-1.01; 0.36
Min. and max.	-1.01; 7.24

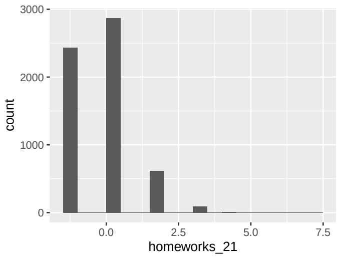

messages_len_28

Feature	Result
Variable type	numeric
Number of missing obs.	0 (0 %)
Number of unique values	623
Median	-0.41
1st and 3rd quartiles	-0.41; 0.05
Min. and max.	-0.41; 39.96

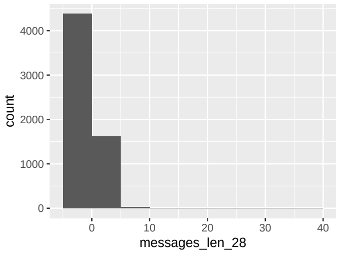

messages_len_tp_28

Feature	Result
Variable type	numeric
Number of missing obs.	0 (0 %)
Number of unique values	1080
Median	-0.25
1st and 3rd quartiles	-0.77; 0.45
Min. and max.	-0.94; 10.31

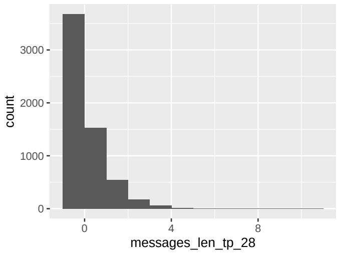

messages_28

Feature	Result
Variable type	numeric
Number of missing obs.	0 (0 %)
Number of unique values	9
Median	-0.72
1st and 3rd quartiles	-0.72; 0.41
Min. and max.	-0.72; 10.59

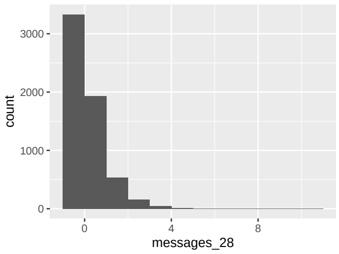

messages_tp_28

Feature	Result
Variable type	numeric
Number of missing obs.	0 (0 %)
Number of unique values	8
Median	-0.24
1st and 3rd quartiles	-0.24; 0.93
Min. and max.	-1.42; 7.97

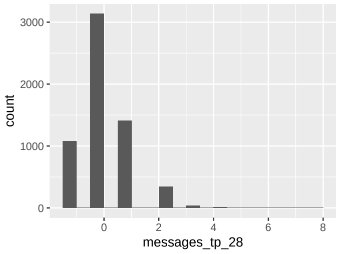

homeworks_28

Feature	Result
Variable type	numeric
Number of missing obs.	0 (0 %)
Number of unique values	8
Median	0.46
1st and 3rd quartiles	-0.91; 0.46
Min. and max.	-0.91; 10.05

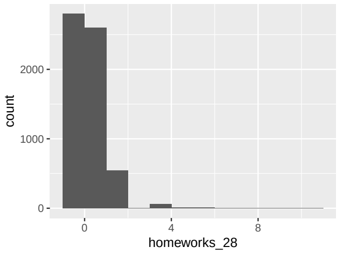

PDSS-SR-3064_SCREEN_sum

Feature	Result
Variable type	numeric
Number of missing obs.	0 (0 %)
Number of unique values	423
Median	-0.03
1st and 3rd quartiles	-0.82; 0.75
Min. and max.	-1.29; 3.12

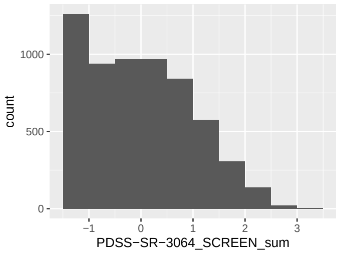

MADRS-1951_SCREEN_sum

Feature	Result
Variable type	numeric
Number of missing obs.	0 (0 %)
Number of unique values	207
Median	0.05
1st and 3rd quartiles	-0.7; 0.67
Min. and max.	-2.68; 3.4

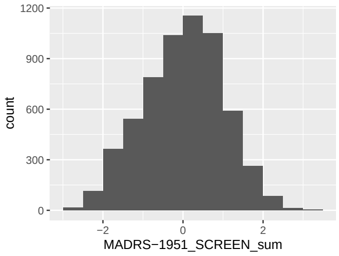

LSAS-2241_SCREEN_sum

Feature	Result
Variable type	numeric
Number of missing obs.	0 (0 %)
Number of unique values	739
Median	-0.12
1st and 3rd quartiles	-0.75; 0.64
Min. and max.	-1.79; 3.09

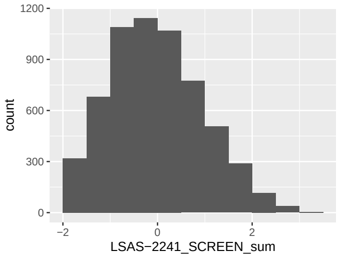

MADRS-1951_SCREEN_DateCompleted_day

Feature	Result
Variable type	numeric
Number of missing obs.	0 (0 %)
Number of unique values	54
Median	0.37
1st and 3rd quartiles	-1; 1
Min. and max.	-1.37; 1.37

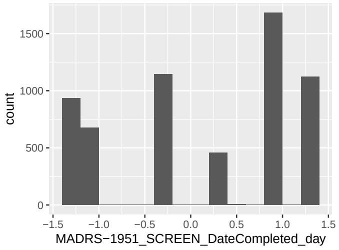

MADRS-1951_SCREEN_DateCompleted_time

Feature	Result
Variable type	numeric
Number of missing obs.	0 (0 %)
Number of unique values	1171
Median	-0.71
1st and 3rd quartiles	-1.24; 0.22
Min. and max.	-1.41; 1.41

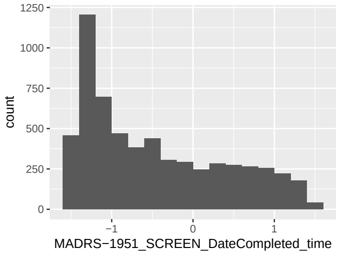

PDSS-SR-3064_SCREEN_DateCompleted_day

Feature	Result
Variable type	numeric
Number of missing obs.	0 (0 %)
Number of unique values	193
Median	0.37
1st and 3rd quartiles	-1; 1
Min. and max.	-1.37; 1.37

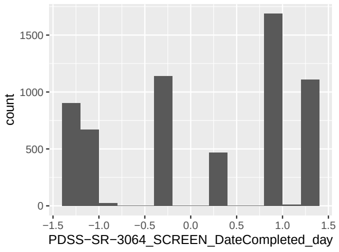

PDSS-SR-3064_SCREEN_DateCompleted_time

Feature	Result
Variable type	numeric
Number of missing obs.	0 (0 %)
Number of unique values	1411
Median	-0.72
1st and 3rd quartiles	-1.24; 0.22
Min. and max.	-1.41; 1.41

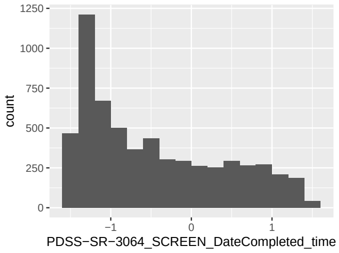

LSAS-2241_SCREEN_DateCompleted_day

Feature	Result
Variable type	numeric
Number of missing obs.	0 (0 %)
Number of unique values	159
Median	0.37
1st and 3rd quartiles	-1; 1
Min. and max.	-1.37; 1.37

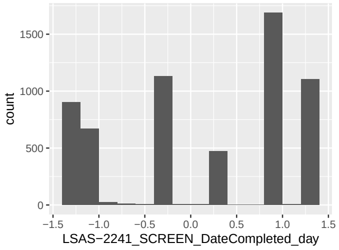

LSAS-2241_SCREEN_DateCompleted_time

Feature	Result
Variable type	numeric
Number of missing obs.	0 (0 %)
Number of unique values	1607
Median	-0.7
1st and 3rd quartiles	-1.24; 0.28
Min. and max.	-1.41; 1.41

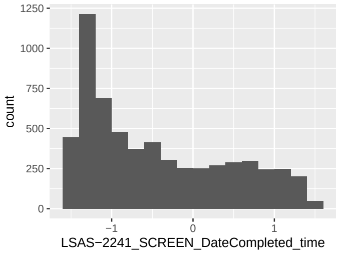

Depression

Feature	Result
Variable type	numeric
Number of missing obs.	0 (0 %)
Number of unique values	2
Median	0
1st and 3rd quartiles	0; 1
Min. and max.	0; 1

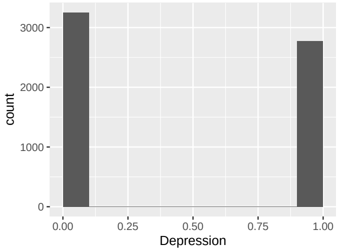

Panic

Feature	Result
Variable type	numeric
Number of missing obs.	0 (0 %)
Number of unique values	2
Median	0
1st and 3rd quartiles	0; 1
Min. and max.	0; 1

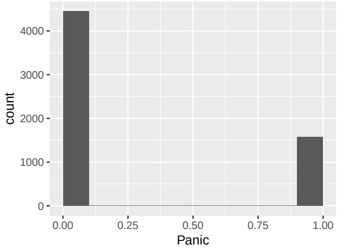

Social_Anxiety

Feature	Result
Variable type	numeric
Number of missing obs.	0 (0 %)
Number of unique values	2
Median	0
1st and 3rd quartiles	0; 1
Min. and max.	0; 1

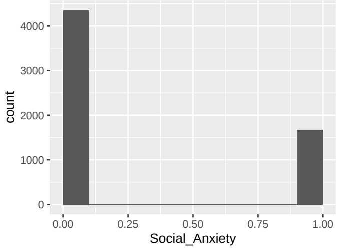

outcome

Feature	Result
Variable type	numeric
Number of missing obs.	0 (0 %)
Number of unique values	1640
Median	-0.06
1st and 3rd quartiles	-0.65; 0.65
Min. and max.	-2.05; 4.66

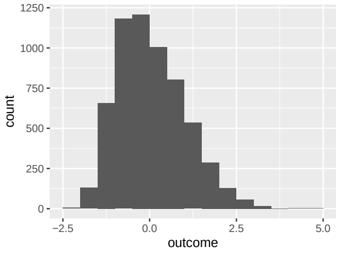

ncomorbid

Feature	Result
Variable type	numeric
Number of missing obs.	0 (0 %)
Number of unique values	118
Median	0
1st and 3rd quartiles	0; 1
Min. and max.	0; 5

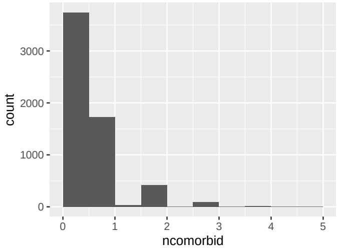

HW-01

Feature	Result
Variable type	numeric
Number of missing obs.	0 (0 %)
Number of unique values	3997
Median	-0.08
1st and 3rd quartiles	-0.67; 0.44
Min. and max.	-4.65; 3.37

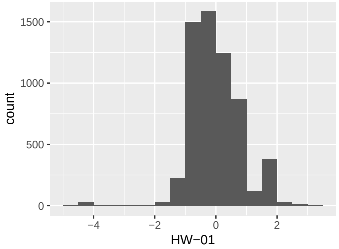

HW-02

Feature	Result
Variable type	numeric
Number of missing obs.	0 (0 %)
Number of unique values	4230
Median	-0.34
1st and 3rd quartiles	-0.54; 0.02
Min. and max.	-1.63; 10.79

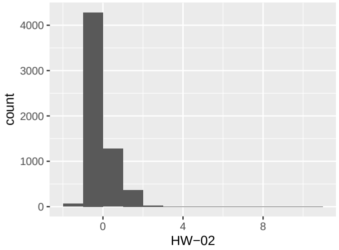

HW-03

Feature	Result
Variable type	numeric
Number of missing obs.	0 (0 %)
Number of unique values	5152
Median	-0.18
1st and 3rd quartiles	-0.39; 0.07
Min. and max.	-4.83; 2.04

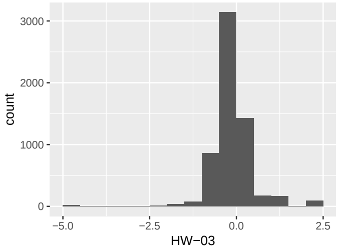

currentwork_proff

Feature	Result
Variable type	factor
Number of missing obs.	0 (0 %)
Number of unique values	63
Mode	“1”
Reference category	0

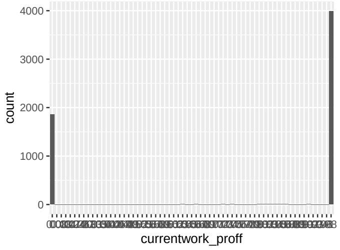

- Observed factor levels: "0", "0.08", "0.13", "0.14", "0.17", "0.24", "0.26", "0.27", "0.3", "0.31", "0.35", "0.36", "0.41", "0.42", "0.46", "0.47", "0.48", "0.49", "0.5", "0.52", "0.55", "0.56", "0.57", "0.58", "0.59", "0.6", "0.61", "0.62", "0.63", "0.64", "0.65", "0.66", "0.67", "0.68", "0.69", "0.7", "0.71", "0.72", "0.73", "0.74", "0.75", "0.76", "0.77", "0.78", "0.79", "0.8", "0.81", "0.82", "0.83", "0.84", "0.85", "0.86", "0.87", "0.88", "0.89", "0.9", "0.91", "0.92", "0.93", "0.94", "0.96", "0.98", "1".

Marital_1833_gift

Feature	Result
Variable type	factor
Number of missing obs.	0 (0 %)
Number of unique values	2
Mode	“1”
Reference category	0

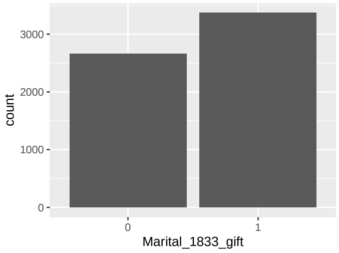

- Observed factor levels: "0", "1".

Marital_1833_separerad

Feature	Result
Variable type	factor
Number of missing obs.	0 (0 %)
Number of unique values	2
Mode	“0”
Reference category	0

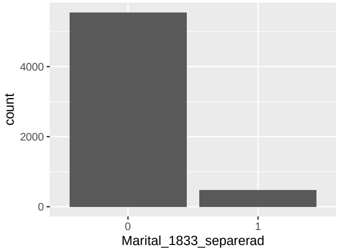

- Observed factor levels: "0", "1".

Marital_1833_singel

Feature	Result
Variable type	factor
Number of missing obs.	0 (0 %)
Number of unique values	2
Mode	“0”
Reference category	0

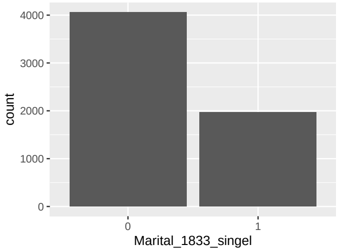

- Observed factor levels: "0", "1".

Edu_1843_2

Feature	Result
Variable type	factor
Number of missing obs.	0 (0 %)
Number of unique values	2
Mode	“0”
Reference category	0

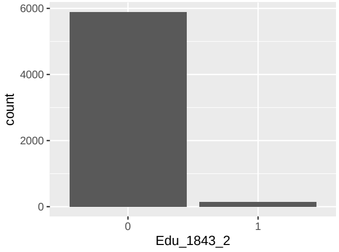

- Observed factor levels: "0", "1".

Edu_1843_3

Feature	Result
Variable type	factor
Number of missing obs.	0 (0 %)
Number of unique values	2
Mode	“0”
Reference category	0

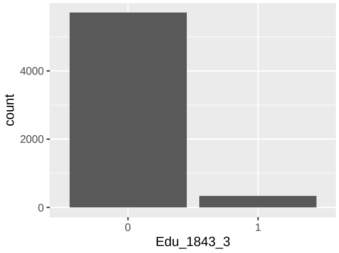

- Observed factor levels: "0", "1".

Edu_1843_4

Feature	Result
Variable type	factor
Number of missing obs.	0 (0 %)
Number of unique values	2
Mode	“0”
Reference category	0

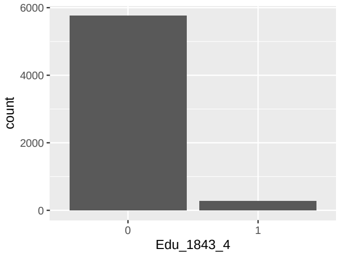

- Observed factor levels: "0", "1".

Edu_1843_5

Feature	Result
Variable type	factor
Number of missing obs.	0 (0 %)
Number of unique values	2
Mode	“0”
Reference category	0

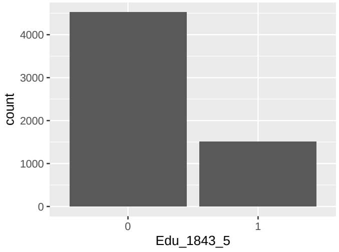

- Observed factor levels: "0", "1".

Edu_1843_6

Feature	Result
Variable type	factor
Number of missing obs.	0 (0 %)
Number of unique values	2
Mode	“0”
Reference category	0

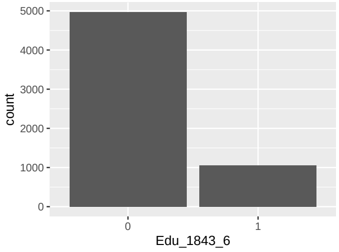

- Observed factor levels: "0", "1".

Edu_1843_7

Feature	Result
Variable type	factor
Number of missing obs.	0 (0 %)
Number of unique values	2
Mode	"0"
Reference category	0

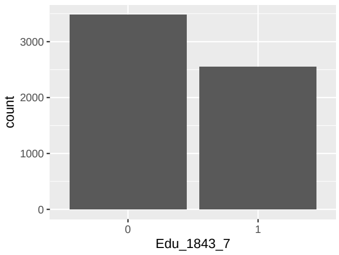

- Observed factor levels: "0", "1".

---

cscale

Feature	Result
Variable type	numeric
Number of missing obs.	0 (0 %)
Number of unique values	642
Median	0.04
1st and 3rd quartiles	-0.58; 0.66
Min. and max.	-4.14; 1.79

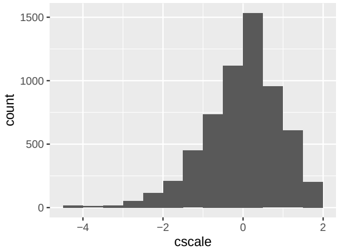

---

mainsymptom_PRE_sum

Feature	Result
Variable type	numeric
Number of missing obs.	0 (0 %)
Number of unique values	432
Median	0.27
1st and 3rd quartiles	-0.46; 0.89
Min. and max.	-2.7; 3.48

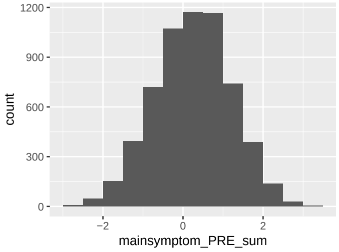

---

mainsymptom_PRE_duration

Feature	Result
Variable type	numeric
Number of missing obs.	0 (0 %)
Number of unique values	2483
Median	-0.12
1st and 3rd quartiles	-0.2; 0
Min. and max.	-0.44; 34.1

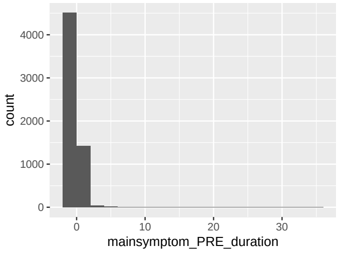

---

mainsymptom_PRE_DateCompleted_day

Feature	Result
Variable type	numeric
Number of missing obs.	0 (0 %)
Number of unique values	238
Median	0.37
1st and 3rd quartiles	-0.37; 1
Min. and max.	-1.37; 1.37

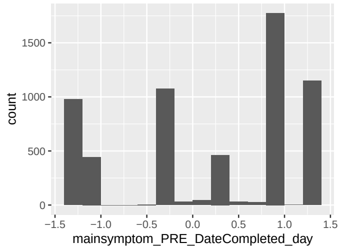

mainsymptom_PRE_DateCompleted_time

Feature	Result
Variable type	numeric
Number of missing obs.	0 (0 %)
Number of unique values	1215
Median	-0.67
1st and 3rd quartiles	-1.22; 0.08
Min. and max.	-1.41; 1.41

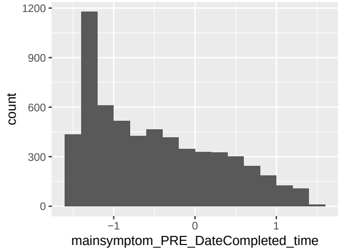

mainsymptom_WEEK01_sum

Feature	Result
Variable type	numeric
Number of missing obs.	0 (0 %)
Number of unique values	1300
Median	0.19
1st and 3rd quartiles	-0.42; 0.9
Min. and max.	-2.84; 3.6

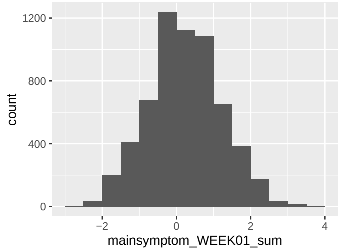

mainsymptom_WEEK01_duration

Feature	Result
Variable type	numeric
Number of missing obs.	0 (0 %)
Number of unique values	2498
Median	-0.09
1st and 3rd quartiles	-0.17; 0.03
Min. and max.	-0.62; 35.17

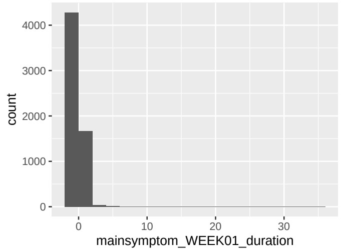

mainsymptom_WEEK01_DateCompleted_day

Feature	Result
Variable type	numeric
Number of missing obs.	0 (0 %)
Number of unique values	1046
Median	0.37
1st and 3rd quartiles	-0.37; 1
Min. and max.	-1.37; 1.37

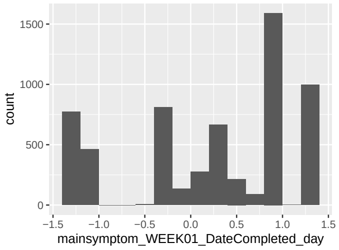

mainsymptom_WEEK01_DateCompleted_time

Feature	Result
Variable type	numeric
Number of missing obs.	0 (0 %)
Number of unique values	2109
Median	-0.63
1st and 3rd quartiles	-1.1; 0
Min. and max.	-1.41; 1.41

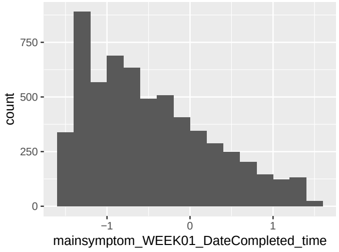

mainsymptom_WEEK02_sum

Feature	Result
Variable type	numeric
Number of missing obs.	0 (0 %)
Number of unique values	1148
Median	0.14
1st and 3rd quartiles	-0.46; 0.86
Min. and max.	-2.75; 4.09

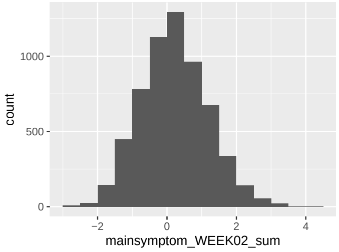

mainsymptom_WEEK02_duration

Feature	Result
Variable type	numeric
Number of missing obs.	0 (0 %)
Number of unique values	2291
Median	-0.09
1st and 3rd quartiles	-0.18; 0.01
Min. and max.	-0.46; 63.88

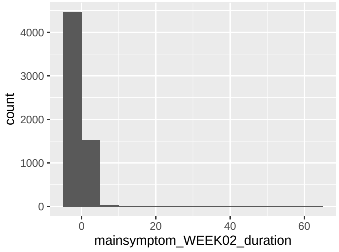

mainsymptom_WEEK02_DateCompleted_day

Feature	Result
Variable type	numeric
Number of missing obs.	0 (0 %)
Number of unique values	926
Median	0.38
1st and 3rd quartiles	-0.37; 1
Min. and max.	-1.37; 1.37

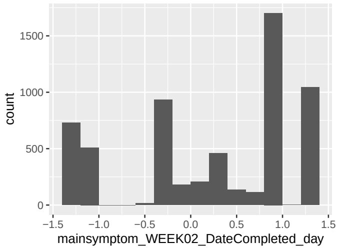

mainsymptom_WEEK02_DateCompleted_time

Feature	Result
Variable type	numeric
Number of missing obs.	0 (0 %)
Number of unique values	1969
Median	-0.64
1st and 3rd quartiles	-1.12; -0.01
Min. and max.	-1.41; 1.41

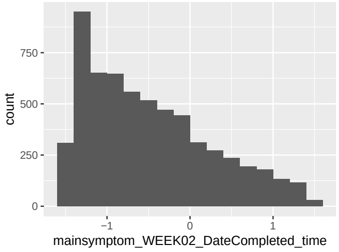

mainsymptom_WEEK03_sum

Feature	Result
Variable type	numeric
Number of missing obs.	0 (0 %)
Number of unique values	1248
Median	0.17
1st and 3rd quartiles	-0.45; 0.83
Min. and max.	-2.64; 4.64

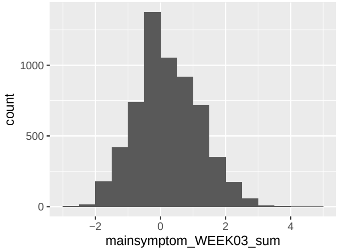

mainsymptom_WEEK03_duration

Feature	Result
Variable type	numeric
Number of missing obs.	0 (0 %)
Number of unique values	2351
Median	-0.08
1st and 3rd quartiles	-0.14; 0
Min. and max.	-0.28; 45.7

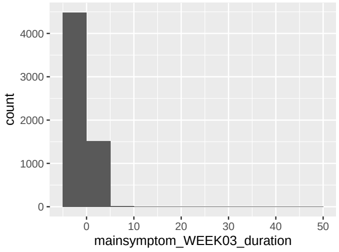

mainsymptom_WEEK03_DateCompleted_day

Feature	Result
Variable type	numeric
Number of missing obs.	0 (0 %)
Number of unique values	1028
Median	0.37
1st and 3rd quartiles	-0.37; 1
Min. and max.	-1.37; 1.37

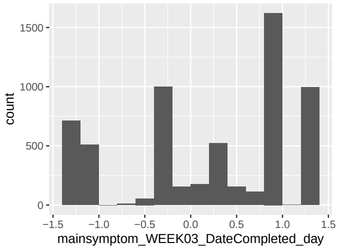

---

mainsymptom_WEEK03_DateCompleted_time

Feature	Result
Variable type	numeric
Number of missing obs.	0 (0 %)
Number of unique values	2071
Median	-0.6
1st and 3rd quartiles	-1.1; 0.03
Min. and max.	-1.41; 1.41

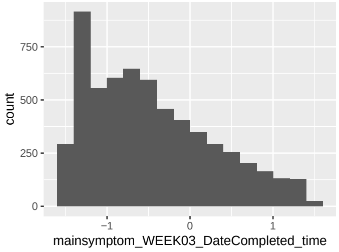

Report generation information:

- Created by: Could not determine from system (username: `nilisa`).
- Report creation time: Mon Jan 09 2023 13:04:17
- Report was run from directory: `/home/nilisa/projects/phd_study1/r`
- dataMaid v1.4.1 [Pkg: 2021-10-08 from CRAN (R 4.2.2)]
- R version 4.2.2 Patched (2022-11-10 r83330).
- Platform: x86_64-pc-linux-gnu (64-bit)(Ubuntu 20.04.5 LTS).
- Function call: `dataMaid::makeDataReport(data = gd, mode = c("summarize", "visualize", "check"), smartNum = FALSE, file = "~/projects/data/study1multiverse/results/graphs_n_figures/codebooks/codebook replace = TRUE, openResult = FALSE, checks = list(character = "showAllFactorLevels", factor = "showAllFactorLevels", labelled = "showAllFactorLevels",   haven_labelled = "showAllFactorLevels", numeric = NULL,   integer = NULL, logical = NULL, Date = NULL), listChecks = FALSE, maxProbVals = Inf, codebook = TRUE, reportTitle = "Handpicked_All_week04-imputed_`

Handpicked_All_week04-naremove_benchmark_test

Autogenerated data summary from dataMaid

2023-01-09 13:11:24

Data report overview

The dataset examined has the following dimensions:

Feature	Result
Number of observations	353
Number of variables	10

Codebook summary table

Label	Variable	Class	# unique values	Missing	Description
	**sex**	factor	2	0.00 %	Sex of patient, 0 = Female, 1=Male
	**age**	numeric	53	0.00 %	
	**PDSS-SR-3064_SCREEN_sum**	numeric	25	0.00 %	Anxiety questionnaire, self rated-Timepoint before treatment starts-Sum of the entire measure
	**MADRS-1951_SCREEN_sum**	numeric	40	0.00 %	Depression questionnaire, self rated-Timepoint before treatment starts-Sum of the entire measure
	**LSAS-2241_SCREEN_sum**	numeric	109	0.00 %	Social anxiety questionnaire, self rated-Timepoint before treatment starts-Sum of the entire measure
	**outcome**	numeric	110	0.00 %	
	**mainsymptom_PRE_sum**	numeric	109	0.00 %	PDSS-SR for panic, MADRS for depression, LSAS for social anxiety-Timepoint just before beginning treatment-Sum of the entire measure
	**mainsymptom_WEEK01_sum**	numeric	109	0.00 %	PDSS-SR for panic, MADRS for depression, LSAS for social anxiety-Timepoint after one week in treatment-Sum of the entire measure
	**mainsymptom_WEEK02_sum**	numeric	109	0.00 %	PDSS-SR for panic, MADRS for depression, LSAS for social anxiety-Timepoint after two weeks in treatment-Sum of the entire measure

Label	Variable	Class	# unique values	Missing	Description
	**mainsymptom_WEEK03_sum**	numeric	109	0.00 %	PDSS-SR for panic, MADRS for depression, LSAS for social anxiety-Timepoint after three weeks in treatment-Sum of the entire measure

Variable list

sex

Feature	Result
Variable type	factor
Number of missing obs.	0 (0 %)
Number of unique values	2
Mode	“0”
Reference category	0

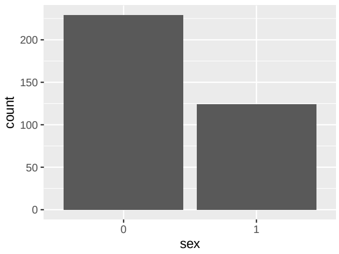

- Observed factor levels: "0", "1".

age

Feature	Result
Variable type	numeric
Number of missing obs.	0 (0 %)
Number of unique values	53
Median	-0.2
1st and 3rd quartiles	-0.81; 0.67
Min. and max.	-1.51; 3.73

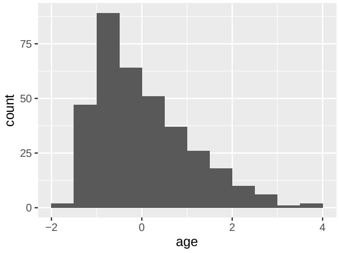

PDSS-SR-3064_SCREEN_sum

Feature	Result
Variable type	numeric
Number of missing obs.	0 (0 %)
Number of unique values	25
Median	0.12
1st and 3rd quartiles	-0.82; 0.75
Min. and max.	-1.29; 2.49

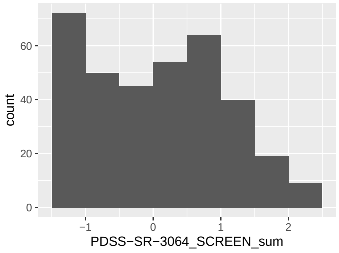

MADRS-1951_SCREEN_sum

Feature	Result
Variable type	numeric
Number of missing obs.	0 (0 %)
Number of unique values	40
Median	0.05
1st and 3rd quartiles	-0.7; 0.67
Min. and max.	-2.43; 3.02

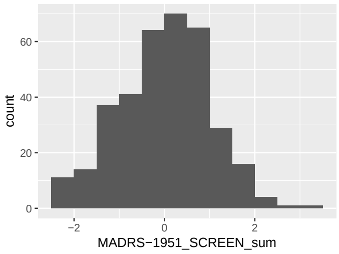

LSAS-2241_SCREEN_sum

Feature	Result
Variable type	numeric
Number of missing obs.	0 (0 %)
Number of unique values	109
Median	-0.13
1st and 3rd quartiles	-0.78; 0.81
Min. and max.	-1.79; 2.99

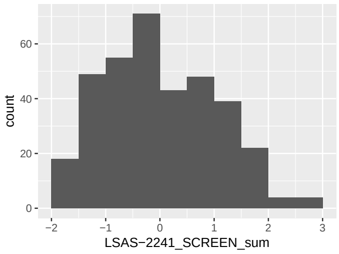

outcome

Feature	Result
Variable type	numeric
Number of missing obs.	0 (0 %)
Number of unique values	110
Median	-0.09
1st and 3rd quartiles	-0.71; 0.53
Min. and max.	-1.78; 3.53

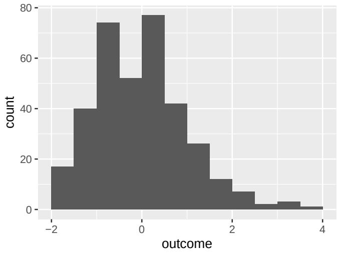

mainsymptom_PRE_sum

Feature	Result
Variable type	numeric
Number of missing obs.	0 (0 %)
Number of unique values	109
Median	0.27
1st and 3rd quartiles	-0.28; 0.89
Min. and max.	-2.37; 2.87

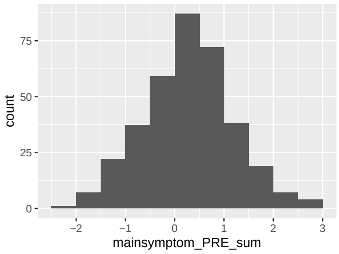

mainsymptom_WEEK01_sum

Feature	Result
Variable type	numeric
Number of missing obs.	0 (0 %)
Number of unique values	109
Median	0.19
1st and 3rd quartiles	-0.3; 0.78
Min. and max.	-2.22; 2.87

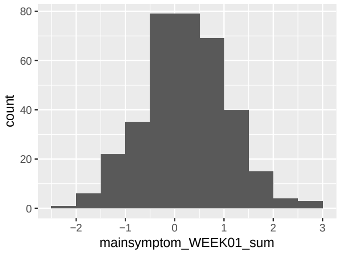

---

mainsymptom_WEEK02_sum

Feature	Result
Variable type	numeric
Number of missing obs.	0 (0 %)
Number of unique values	109
Median	0.08
1st and 3rd quartiles	-0.52; 0.71
Min. and max.	-2.18; 2.93

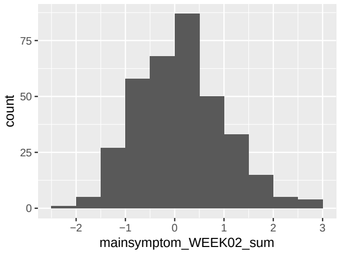

---

mainsymptom_WEEK03_sum

Feature	Result
Variable type	numeric
Number of missing obs.	0 (0 %)
Number of unique values	109
Median	-0.01
1st and 3rd quartiles	-0.6; 0.71
Min. and max.	-2.08; 3.07

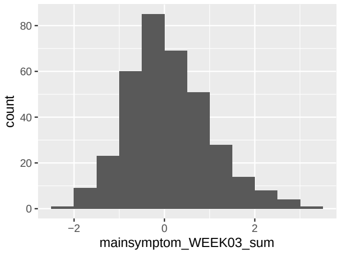

Report generation information:

- Created by: Could not determine from system (username: `nilisa`).
- Report creation time: Mon Jan 09 2023 13:11:25
- Report was run from directory: `/home/nilisa/projects/phd_study1/r`
- dataMaid v1.4.1 [Pkg: 2021-10-08 from CRAN (R 4.2.2)]
- R version 4.2.2 Patched (2022-11-10 r83330).
- Platform: x86_64-pc-linux-gnu (64-bit)(Ubuntu 20.04.5 LTS).
- Function call: `dataMaid::makeDataReport(data = gd, mode = c("summarize", "visualize", "check"), smartNum = FALSE, file = "~/projects/data/study1multiverse/results/graphs_n_figures/codebooks/codebook replace = TRUE, openResult = FALSE, checks = list(character = "showAllFactorLevels", factor = "showAllFactorLevels", labelled = "showAllFactorLevels",   haven_labelled = "showAllFactorLevels", numeric = NULL,   integer = NULL, logical = NULL, Date = NULL), listChecks = FALSE, maxProbVals = Inf, codebook = TRUE, reportTitle = "Handpicked_All_week04-naremove`

Handpicked_All_week04-naremove_benchmark_train

Autogenerated data summary from dataMaid

2023-01-09 13:04:58

Data report overview

The dataset examined has the following dimensions:

Feature	Result
Number of observations	3190
Number of variables	10

Codebook summary table

Label	Variable	Class	# unique values	Missing	Description
	**sex**	factor	2	0.00 %	Sex of patient, 0 = Female, 1=Male
	**age**	numeric	65	0.00 %	
	**PDSS-SR-3064_SCREEN_sum**	numeric	28	0.00 %	Anxiety questionnaire, self rated-Timepoint before treatment starts-Sum of the entire measure
	**MADRS-1951_SCREEN_sum**	numeric	44	0.00 %	Depression questionnaire, self rated-Timepoint before treatment starts-Sum of the entire measure
	**LSAS-2241_SCREEN_sum**	numeric	134	0.00 %	Social anxiety questionnaire, self rated-Timepoint before treatment starts-Sum of the entire measure
	**outcome**	numeric	185	0.00 %	
	**mainsymptom_PRE_sum**	numeric	180	0.00 %	PDSS-SR for panic, MADRS for depression, LSAS for social anxiety-Timepoint just before beginning treatment-Sum of the entire measure
	**mainsymptom_WEEK01_sum**	numeric	183	0.00 %	PDSS-SR for panic, MADRS for depression, LSAS for social anxiety-Timepoint after one week in treatment-Sum of the entire measure
	**mainsymptom_WEEK02_sum**	numeric	186	0.00 %	PDSS-SR for panic, MADRS for depression, LSAS for social anxiety-Timepoint after two weeks in treatment-Sum of the entire measure

Label	Variable	Class	# unique values	Missing	Description
	**mainsymptom_WEEK03_sum**	numeric	184	0.00 %	PDSS-SR for panic, MADRS for depression, LSAS for social anxiety-Timepoint after three weeks in treatment-Sum of the entire measure

Variable list

sex

Feature	Result
Variable type	factor
Number of missing obs.	0 (0 %)
Number of unique values	2
Mode	"0"
Reference category	0

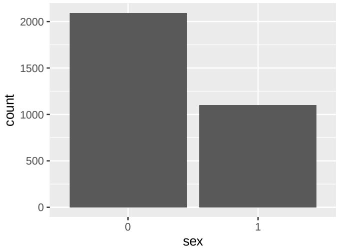

- Observed factor levels: "0", "1".

---

age

Feature	Result
Variable type	numeric
Number of missing obs.	0 (0 %)
Number of unique values	65
Median	-0.2
1st and 3rd quartiles	-0.73; 0.67
Min. and max.	-1.69; 4.25

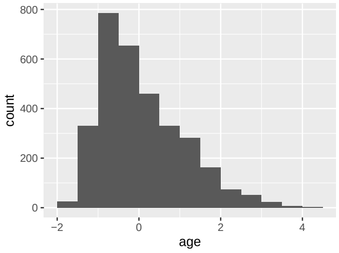

PDSS-SR-3064_SCREEN_sum

Feature	Result
Variable type	numeric
Number of missing obs.	0 (0 %)
Number of unique values	28
Median	-0.03
1st and 3rd quartiles	-0.98; 0.75
Min. and max.	-1.29; 3.12

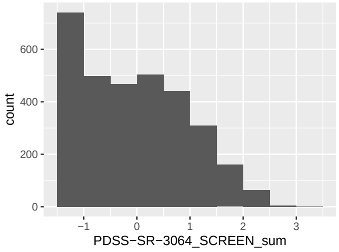

MADRS-1951_SCREEN_sum

Feature	Result
Variable type	numeric
Number of missing obs.	0 (0 %)
Number of unique values	44
Median	0.05
1st and 3rd quartiles	-0.7; 0.67
Min. and max.	-2.68; 2.65

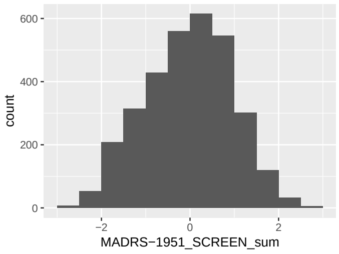

LSAS-2241_SCREEN_sum

Feature	Result
Variable type	numeric
Number of missing obs.	0 (0 %)
Number of unique values	134
Median	-0.09
1st and 3rd quartiles	-0.78; 0.67
Min. and max.	-1.79; 3.02

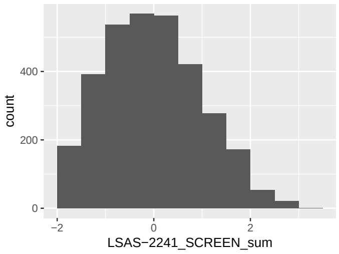

outcome

Feature	Result
Variable type	numeric
Number of missing obs.	0 (0 %)
Number of unique values	185
Median	-0.18
1st and 3rd quartiles	-0.77; 0.53
Min. and max.	-2.05; 4.04

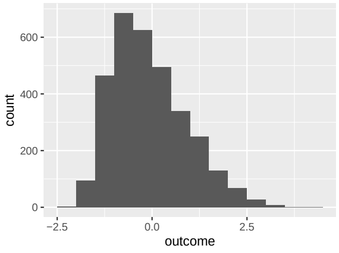

mainsymptom_PRE_sum

Feature	Result
Variable type	numeric
Number of missing obs.	0 (0 %)
Number of unique values	180
Median	0.27
1st and 3rd quartiles	-0.37; 0.89
Min. and max.	-2.49; 3

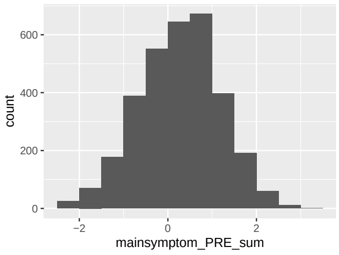

mainsymptom_WEEK01_sum

Feature	Result
Variable type	numeric
Number of missing obs.	0 (0 %)
Number of unique values	183
Median	0.19
1st and 3rd quartiles	-0.42; 0.82
Min. and max.	-2.84; 3.6

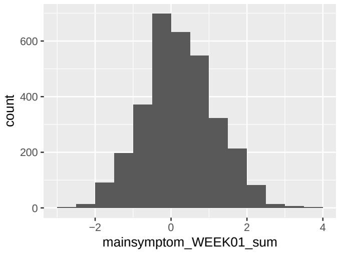

mainsymptom_WEEK02_sum

Feature	Result
Variable type	numeric
Number of missing obs.	0 (0 %)
Number of unique values	186
Median	0.09
1st and 3rd quartiles	-0.52; 0.74
Min. and max.	-2.75; 3.44

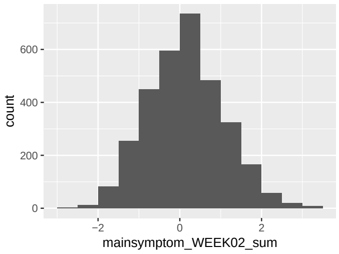

mainsymptom_WEEK03_sum

Feature	Result
Variable type	numeric
Number of missing obs.	0 (0 %)
Number of unique values	184
Median	0.09
1st and 3rd quartiles	-0.48; 0.82
Min. and max.	-2.64; 3.99

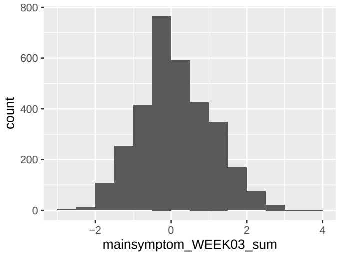

Report generation information:

- Created by: Could not determine from system (username: `nilisa`).
- Report creation time: Mon Jan 09 2023 13:04:58
- Report was run from directory: `/home/nilisa/projects/phd_study1/r`
- dataMaid v1.4.1 [Pkg: 2021-10-08 from CRAN (R 4.2.2)]
- R version 4.2.2 Patched (2022-11-10 r83330).
- Platform: x86_64-pc-linux-gnu (64-bit)(Ubuntu 20.04.5 LTS).
- Function call: `dataMaid::makeDataReport(data = gd, mode = c("summarize", "visualize", "check"), smartNum = FALSE, file = "~/projects/data/study1multiverse/results/graphs_n_figures/codebooks/codebook replace = TRUE, openResult = FALSE, checks = list(character = "showAllFactorLevels", factor = "showAllFactorLevels", labelled = "showAllFactorLevels",   haven_labelled = "showAllFactorLevels", numeric = NULL,   integer = NULL, logical = NULL, Date = NULL), listChecks = FALSE, maxProbVals = Inf, codebook = TRUE, reportTitle = "Handpicked_All_week04-naremove`

Handpicked_All_week04-naremove_test

Autogenerated data summary from dataMaid

2023-01-09 13:11:33

Data report overview

The dataset examined has the following dimensions:

Feature	Result
Number of observations	341
Number of variables	63

Codebook summary table

Label	Variable	Class	# unique values	Missing	Description
	**sex**	factor	2	0.00 %	Sex of patient, 0 = Female, 1=Male
	**age**	numeric	53	0.00 %	
	**messages_len_7**	numeric	118	0.00 %	-Meta information of messages-Length of messages-up until day-7
	**messages_len_tp_7**	numeric	242	0.00 %	-Meta information of messages-Length of messages-therapist messages-up until day-7
	**messages_7**	numeric	6	0.00 %	-Meta information of messages-up until day-7
	**messages_tp_7**	numeric	6	0.00 %	-Meta information of messages-therapist messages-up until day-7
	**homeworks_7**	numeric	6	0.00 %	-Number of homework messages sent in-up until day-7
	**messages_len_14**	numeric	146	0.00 %	-Meta information of messages-Length of messages-up until day-14
	**messages_len_tp_14**	numeric	266	0.00 %	-Meta information of messages-Length of messages-therapist messages-up until day-14
	**messages_14**	numeric	6	0.00 %	-Meta information of messages-up until day-14
	**messages_tp_14**	numeric	5	0.00 %	-Meta information of messages-therapist messages-up until day-14

Label	Variable	Class	# unique values	Missing	Description
	**homeworks_14**	numeric	6	0.00 %	-Number of homework messages sent in-up until day-14
	**messages_len_21**	numeric	150	0.00 %	-Meta information of messages-Length of messages-up until day-21
	**messages_len_tp_21**	numeric	273	0.00 %	-Meta information of messages-Length of messages-therapist messages-up until day-21
	**messages_21**	numeric	7	0.00 %	-Meta information of messages-up until day-21
	**messages_tp_21**	numeric	5	0.00 %	-Meta information of messages-therapist messages-up until day-21
	**homeworks_21**	numeric	5	0.00 %	-Number of homework messages sent in-up until day-21
	**messages_len_28**	numeric	155	0.00 %	-Meta information of messages-Length of messages-up until day-28
	**messages_len_tp_28**	numeric	262	0.00 %	-Meta information of messages-Length of messages-therapist messages-up until day-28
	**messages_28**	numeric	5	0.00 %	-Meta information of messages-up until day-28
	**messages_tp_28**	numeric	5	0.00 %	-Meta information of messages-therapist messages-up until day-28
	**homeworks_28**	numeric	5	0.00 %	-Number of homework messages sent in-up until day-28
	**PDSS-SR-3064_SCREEN_sum**	numeric	25	0.00 %	Anxiety questionnaire, self rated-Timepoint before treatment starts-Sum of the entire measure
	**MADRS-1951_SCREEN_sum**	numeric	40	0.00 %	Depression questionnaire, self rated-Timepoint before treatment starts-Sum of the entire measure
	**LSAS-2241_SCREEN_sum**	numeric	108	0.00 %	Social anxiety questionnaire, self rated-Timepoint before treatment starts-Sum of the entire measure
	**MADRS-1951_SCREEN_DateCompleted_day**	numeric	7	0.00 %	Depression questionnaire, self rated-Timepoint before treatment starts-Cyclic transformation of what day 0-6 during week it was filled in

Label	Variable	Class	# unique values	Missing	Description
	**MADRS-1951_SCREEN_DateCompleted_time**	numeric	269	0.00 %	Depression questionnaire, self rated-Timepoint before treatment starts-Cyclic transformation of what time during day 0-1440 it was filled in
	**PDSS-SR-3064_SCREEN_DateCompleted_day**	numeric	7	0.00 %	Anxiety questionnaire, self rated-Timepoint before treatment starts-Cyclic transformation of what day 0-6 during week it was filled in
	**PDSS-SR-3064_SCREEN_DateCompleted_time**	numeric	283	0.00 %	Anxiety questionnaire, self rated-Timepoint before treatment starts-Cyclic transformation of what time during day 0-1440 it was filled in
	**LSAS-2241_SCREEN_DateCompleted_day**	numeric	7	0.00 %	Social anxiety questionnaire, self rated-Timepoint before treatment starts-Cyclic transformation of what day 0-6 during week it was filled in
	**LSAS-2241_SCREEN_DateCompleted_time**	numeric	271	0.00 %	Social anxiety questionnaire, self rated-Timepoint before treatment starts-Cyclic transformation of what time during day 0-1440 it was filled in
	**Depression**	numeric	2	0.00 %	
	**Panic**	numeric	2	0.00 %	
	**Social_Anxiety**	numeric	2	0.00 %	
	**outcome**	numeric	108	0.00 %	
	**ncomorbid**	numeric	5	0.00 %	
	**currentwork_proff**	factor	2	0.00 %	Currently in work for trained proffession
	**Marital_1833_gift**	factor	2	0.00 %	Marital status: Married or not
	**Marital_1833_separerad**	factor	2	0.00 %	Marital status: divocered/equivalent
	**Marital_1833_singel**	factor	2	0.00 %	Marital status: single
	**Edu_1843_2**	factor	2	0.00 %	7-9 years education
	**Edu_1843_3**	factor	2	0.00 %	Uncompleted upper secondary school
	**Edu_1843_4**	factor	2	0.00 %	Higher vocational education
	**Edu_1843_5**	factor	2	0.00 %	Completed upper secondary school
	**Edu_1843_6**	factor	2	0.00 %	Uncompleted university degree
	**Edu_1843_7**	factor	2	0.00 %	University degree
	**cscale**	numeric	38	0.00 %	
	**mainsymptom_PRE_sum**	numeric	108	0.00 %	PDSS-SR for panic, MADRS for depression, LSAS for social anxiety-Timepoint just before beginning treatment-Sum of the entire measure

Label	Variable	Class	# unique values	Missing	Description
	**mainsymptom_PRE_duration**	numeric	310	0.00 %	PDSS-SR for panic, MADRS for depression, LSAS for social anxiety-Timepoint just before beginning treatment-Time to fill in measure/questionnaire
	**mainsymptom_PRE_DateCompleted_day**	numeric	7	0.00 %	PDSS-SR for panic, MADRS for depression, LSAS for social anxiety-Timepoint just before beginning treatment-Cyclic transformation of what day 0-6 during week it was filled in
	**mainsymptom_PRE_DateCompleted_time**	numeric	276	0.00 %	PDSS-SR for panic, MADRS for depression, LSAS for social anxiety-Timepoint just before beginning treatment-Cyclic transformation of what time during day 0-1440 it was filled in
	**mainsymptom_WEEK01_sum**	numeric	107	0.00 %	PDSS-SR for panic, MADRS for depression, LSAS for social anxiety-Timepoint after one week in treatment-Sum of the entire measure
	**mainsymptom_WEEK01_duration**	numeric	271	0.00 %	PDSS-SR for panic, MADRS for depression, LSAS for social anxiety-Timepoint after one week in treatment-Time to fill in measure/questionnaire
	**mainsymptom_WEEK01_DateCompleted_day**	numeric	7	0.00 %	PDSS-SR for panic, MADRS for depression, LSAS for social anxiety-Timepoint after one week in treatment-Cyclic transformation of what day 0-6 during week it was filled in
	**mainsymptom_WEEK01_DateCompleted_time**	numeric	279	0.00 %	PDSS-SR for panic, MADRS for depression, LSAS for social anxiety-Timepoint after one week in treatment-Cyclic transformation of what time during day 0-1440 it was filled in
	**mainsymptom_WEEK02_sum**	numeric	108	0.00 %	PDSS-SR for panic, MADRS for depression, LSAS for social anxiety-Timepoint after two weeks in treatment-Sum of the entire measure
	**mainsymptom_WEEK02_duration**	numeric	282	0.00 %	PDSS-SR for panic, MADRS for depression, LSAS for social anxiety-Timepoint after two weeks in treatment-Time to fill in measure/questionnaire

Label	Variable	Class	# unique values	Missing	Description
	**mainsymptom_WEEK02_DateCompleted_day**	numeric	7	0.00 %	PDSS-SR for panic, MADRS for depression, LSAS for social anxiety-Timepoint after two weeks in treatment-Cyclic transformation of what day 0-6 during week it was filled in
	**mainsymptom_WEEK02_DateCompleted_time**	numeric	272	0.00 %	PDSS-SR for panic, MADRS for depression, LSAS for social anxiety-Timepoint after two weeks in treatment-Cyclic transformation of what time during day 0-1440 it was filled in
	**mainsymptom_WEEK03_sum**	numeric	109	0.00 %	PDSS-SR for panic, MADRS for depression, LSAS for social anxiety-Timepoint after three weeks in treatment-Sum of the entire measure
	**mainsymptom_WEEK03_duration**	numeric	269	0.00 %	PDSS-SR for panic, MADRS for depression, LSAS for social anxiety-Timepoint after three weeks in treatment-Time to fill in measure/questionnaire
	**mainsymptom_WEEK03_DateCompleted_day**	numeric	7	0.00 %	PDSS-SR for panic, MADRS for depression, LSAS for social anxiety-Timepoint after three weeks in treatment-Cyclic transformation of what day 0-6 during week it was filled in
	**mainsymptom_WEEK03_DateCompleted_time**	numeric	282	0.00 %	PDSS-SR for panic, MADRS for depression, LSAS for social anxiety-Timepoint after three weeks in treatment-Cyclic transformation of what time during day 0-1440 it was filled in

Variable list

sex

Feature	Result
Variable type	factor
Number of missing obs.	0 (0 %)
Number of unique values	2
Mode	“0”
Reference category	0

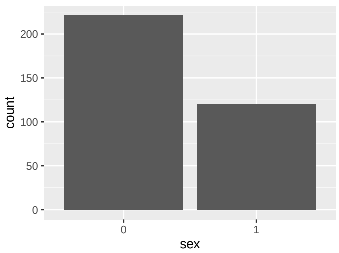

- Observed factor levels: "0", "1".

age

Feature	Result
Variable type	numeric
Number of missing obs.	0 (0 %)
Number of unique values	53
Median	-0.2
1st and 3rd quartiles	-0.81; 0.67
Min. and max.	-1.51; 3.73

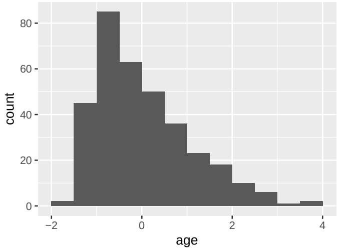

messages_len_7

Feature	Result
Variable type	numeric
Number of missing obs.	0 (0 %)
Number of unique values	118
Median	-0.32
1st and 3rd quartiles	-0.32; 0.13
Min. and max.	-0.32; 5.35

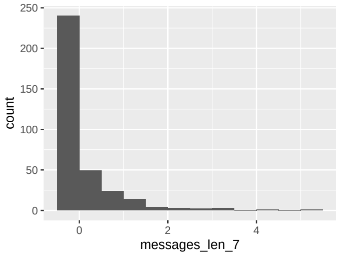

messages_len_tp_7

Feature	Result
Variable type	numeric
Number of missing obs.	0 (0 %)
Number of unique values	242
Median	0.21
1st and 3rd quartiles	-0.36; 0.85
Min. and max.	-1.46; 4.91

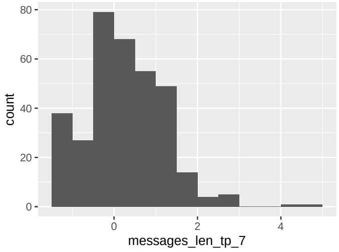

messages_7

Feature	Result
Variable type	numeric
Number of missing obs.	0 (0 %)
Number of unique values	6
Median	-0.55
1st and 3rd quartiles	-0.55; 0.58
Min. and max.	-0.55; 5.13

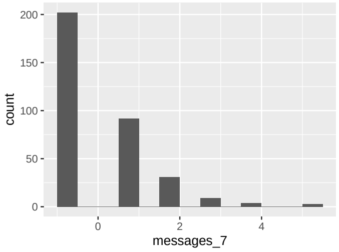

messages_tp_7

Feature	Result
Variable type	numeric
Number of missing obs.	0 (0 %)
Number of unique values	6
Median	0.15
1st and 3rd quartiles	0.15; 1.21
Min. and max.	-1.97; 3.34

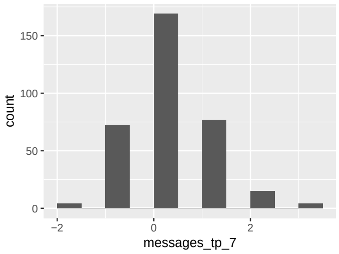

homeworks_7

Feature	Result
Variable type	numeric
Number of missing obs.	0 (0 %)
Number of unique values	6
Median	0.2
1st and 3rd quartiles	0.2; 0.2
Min. and max.	-1.03; 5.15

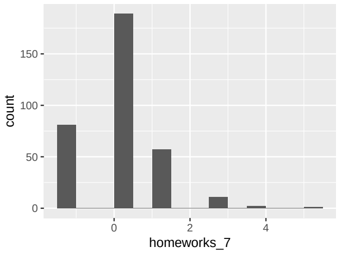

messages_len_14

Feature	Result
Variable type	numeric
Number of missing obs.	0 (0 %)
Number of unique values	146
Median	-0.31
1st and 3rd quartiles	-0.41; 0.27
Min. and max.	-0.41; 12.82

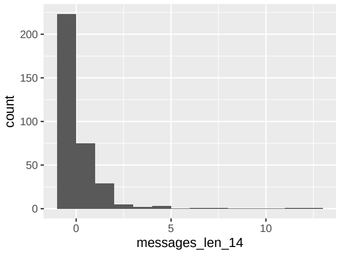

messages_len_tp_14

Feature	Result
Variable type	numeric
Number of missing obs.	0 (0 %)
Number of unique values	266
Median	0.03
1st and 3rd quartiles	-0.58; 0.63
Min. and max.	-1.11; 4.12

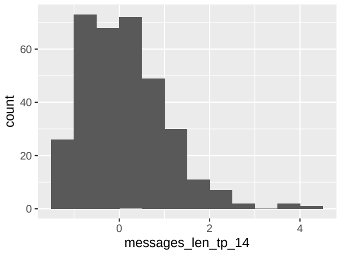

messages_14

Feature	Result
Variable type	numeric
Number of missing obs.	0 (0 %)
Number of unique values	6
Median	0.36
1st and 3rd quartiles	-0.7; 0.36
Min. and max.	-0.7; 4.6

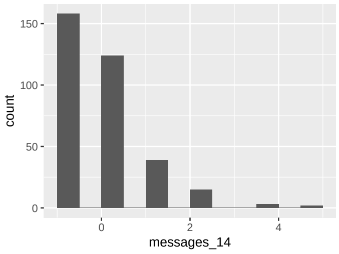

messages_tp_14

Feature	Result
Variable type	numeric
Number of missing obs.	0 (0 %)
Number of unique values	5
Median	-0.35
1st and 3rd quartiles	-0.35; 0.87
Min. and max.	-1.57; 3.31

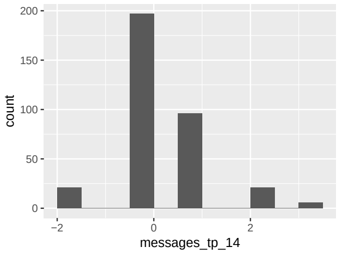

homeworks_14

Feature	Result
Variable type	numeric
Number of missing obs.	0 (0 %)
Number of unique values	6
Median	0.33
1st and 3rd quartiles	-0.98; 0.33
Min. and max.	-0.98; 14.78

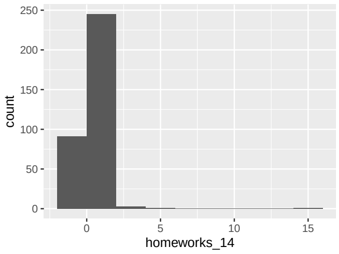

messages_len_21

Feature	Result
Variable type	numeric
Number of missing obs.	0 (0 %)
Number of unique values	150
Median	-0.34
1st and 3rd quartiles	-0.46; 0.3
Min. and max.	-0.46; 7.17

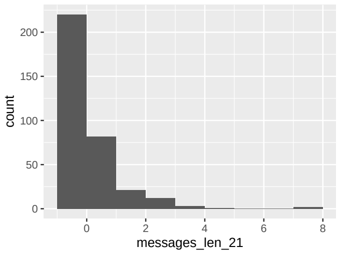

messages_len_tp_21

Feature	Result
Variable type	numeric
Number of missing obs.	0 (0 %)
Number of unique values	273
Median	0.08
1st and 3rd quartiles	-0.48; 0.67
Min. and max.	-1.01; 4.87

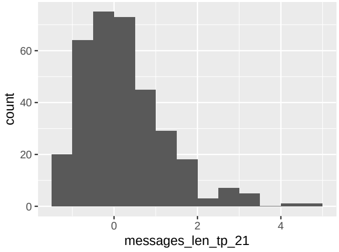

messages_21

Feature	Result
Variable type	numeric
Number of missing obs.	0 (0 %)
Number of unique values	7
Median	0.34
1st and 3rd quartiles	-0.74; 0.34
Min. and max.	-0.74; 5.75

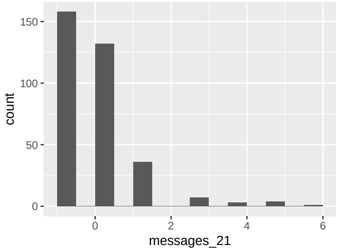

messages_tp_21

Feature	Result
Variable type	numeric
Number of missing obs.	0 (0 %)
Number of unique values	5
Median	-0.32
1st and 3rd quartiles	-0.32; 0.84
Min. and max.	-1.49; 3.17

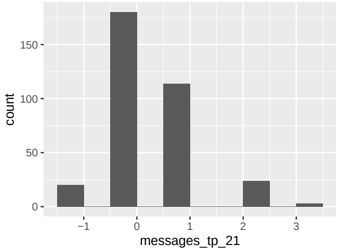

homeworks_21

Feature	Result
Variable type	numeric
Number of missing obs.	0 (0 %)
Number of unique values	5
Median	0.36
1st and 3rd quartiles	-1.01; 0.36
Min. and max.	-1.01; 4.49

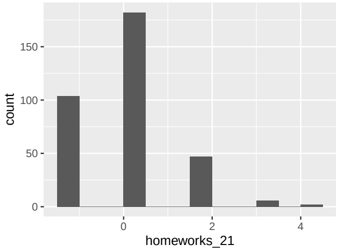

messages_len_28

Feature	Result
Variable type	numeric
Number of missing obs.	0 (0 %)
Number of unique values	155
Median	-0.28
1st and 3rd quartiles	-0.41; 0.37
Min. and max.	-0.41; 7.29

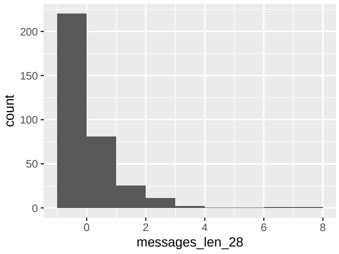

messages_len_tp_28

Feature	Result
Variable type	numeric
Number of missing obs.	0 (0 %)
Number of unique values	262
Median	0.04
1st and 3rd quartiles	-0.51; 0.74
Min. and max.	-0.94; 6.1

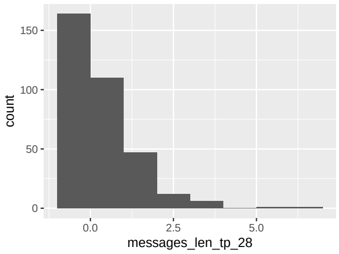

messages_28

Feature	Result
Variable type	numeric
Number of missing obs.	0 (0 %)
Number of unique values	5
Median	0.41
1st and 3rd quartiles	-0.72; 0.41
Min. and max.	-0.72; 3.8

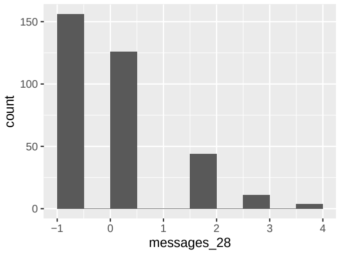

messages_tp_28

Feature	Result
Variable type	numeric
Number of missing obs.	0 (0 %)
Number of unique values	5
Median	-0.24
1st and 3rd quartiles	-0.24; 0.93
Min. and max.	-1.42; 3.28

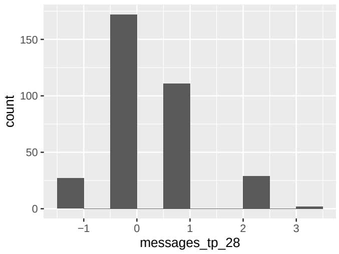

homeworks_28

Feature	Result
Variable type	numeric
Number of missing obs.	0 (0 %)
Number of unique values	5
Median	0.46
1st and 3rd quartiles	-0.91; 0.46
Min. and max.	-0.91; 5.94

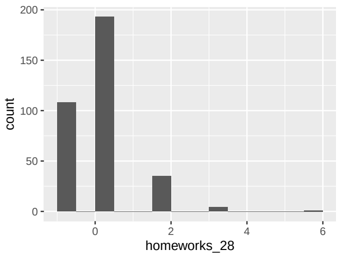

PDSS-SR-3064_SCREEN_sum

Feature	Result
Variable type	numeric
Number of missing obs.	0 (0 %)
Number of unique values	25
Median	0.12
1st and 3rd quartiles	-0.82; 0.75
Min. and max.	-1.29; 2.49

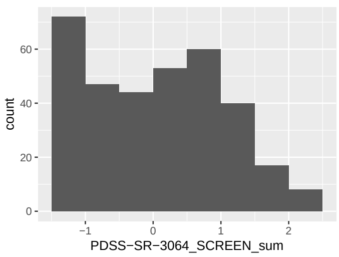

MADRS-1951_SCREEN_sum

Feature	Result
Variable type	numeric
Number of missing obs.	0 (0 %)
Number of unique values	40
Median	0.05
1st and 3rd quartiles	-0.7; 0.67
Min. and max.	-2.43; 3.02

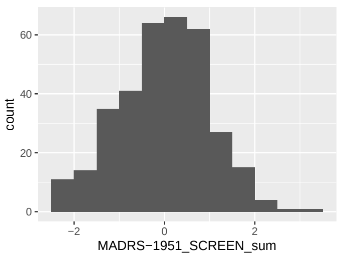

LSAS-2241_SCREEN_sum

Feature	Result
Variable type	numeric
Number of missing obs.	0 (0 %)
Number of unique values	108
Median	-0.13
1st and 3rd quartiles	-0.82; 0.81
Min. and max.	-1.79; 2.99

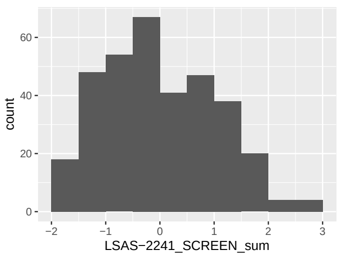

MADRS-1951_SCREEN_DateCompleted_day

Feature	Result
Variable type	numeric
Number of missing obs.	0 (0 %)
Number of unique values	7
Median	0.37
1st and 3rd quartiles	-1; 1
Min. and max.	-1.37; 1.37

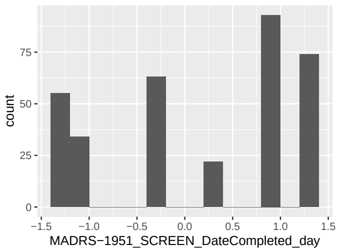

MADRS-1951_SCREEN_DateCompleted_time

Feature	Result
Variable type	numeric
Number of missing obs.	0 (0 %)
Number of unique values	269
Median	-0.8
1st and 3rd quartiles	-1.24; 0.12
Min. and max.	-1.41; 1.35

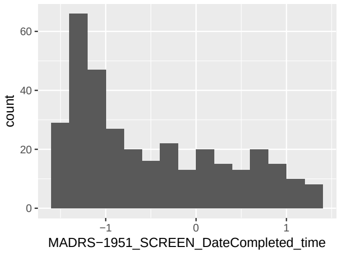

PDSS-SR-3064_SCREEN_DateCompleted_day

Feature	Result
Variable type	numeric
Number of missing obs.	0 (0 %)
Number of unique values	7
Median	0.37
1st and 3rd quartiles	-1; 1
Min. and max.	-1.37; 1.37

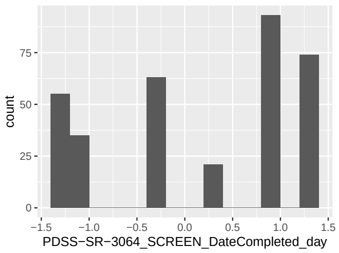

PDSS-SR-3064_SCREEN_DateCompleted_time

Feature	Result
Variable type	numeric
Number of missing obs.	0 (0 %)
Number of unique values	283
Median	-0.81
1st and 3rd quartiles	-1.22; 0.15
Min. and max.	-1.41; 1.36

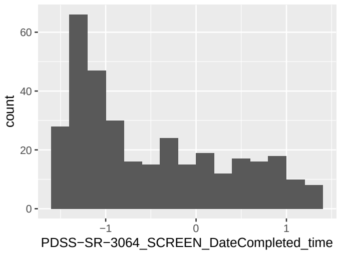

LSAS-2241_SCREEN_DateCompleted_day

Feature	Result
Variable type	numeric
Number of missing obs.	0 (0 %)
Number of unique values	7
Median	0.37
1st and 3rd quartiles	-1; 1
Min. and max.	-1.37; 1.37

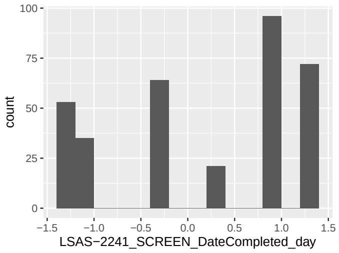

LSAS-2241_SCREEN_DateCompleted_time

Feature	Result
Variable type	numeric
Number of missing obs.	0 (0 %)
Number of unique values	271
Median	-0.78
1st and 3rd quartiles	-1.21; 0.22
Min. and max.	-1.41; 1.37

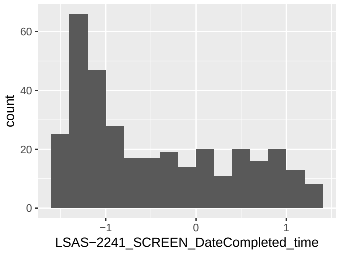

Depression

Feature	Result
Variable type	numeric
Number of missing obs.	0 (0 %)
Number of unique values	2
Median	0
1st and 3rd quartiles	0; 1
Min. and max.	0; 1

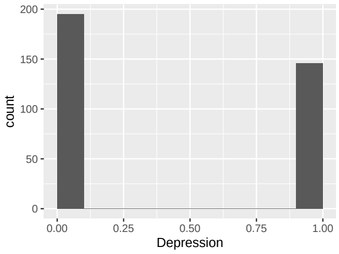

Panic

Feature	Result
Variable type	numeric
Number of missing obs.	0 (0 %)
Number of unique values	2
Median	0
1st and 3rd quartiles	0; 1
Min. and max.	0; 1

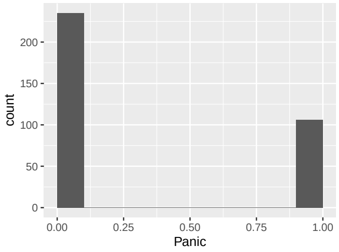

Social_Anxiety

Feature	Result
Variable type	numeric
Number of missing obs.	0 (0 %)
Number of unique values	2
Median	0
1st and 3rd quartiles	0; 1
Min. and max.	0; 1

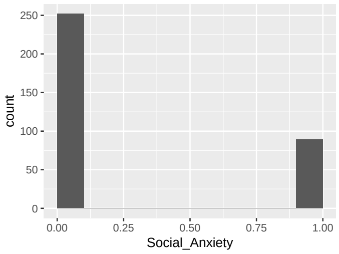

---

outcome

Feature	Result
Variable type	numeric
Number of missing obs.	0 (0 %)
Number of unique values	108
Median	-0.09
1st and 3rd quartiles	-0.71; 0.53
Min. and max.	-1.78; 3.53

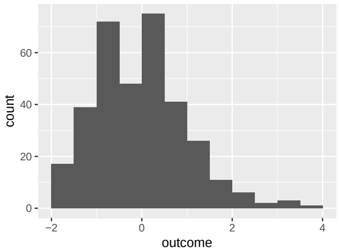

---

ncomorbid

Feature	Result
Variable type	numeric
Number of missing obs.	0 (0 %)
Number of unique values	5
Median	0
1st and 3rd quartiles	0; 1
Min. and max.	0; 4

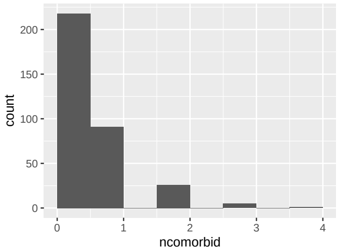

---

currentwork_proff

Feature	Result
Variable type	factor
Number of missing obs.	0 (0 %)
Number of unique values	2
Mode	“1”
Reference category	0

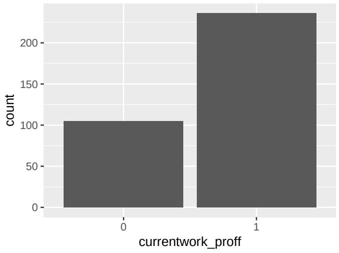

- Observed factor levels: "0", "1".

Marital_1833_gift

Feature	Result
Variable type	factor
Number of missing obs.	0 (0 %)
Number of unique values	2
Mode	“1”
Reference category	0

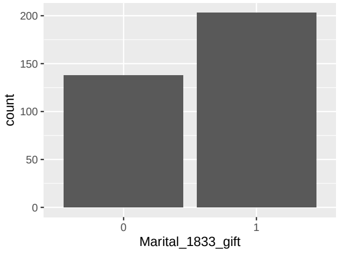

- Observed factor levels: "0", "1".

Marital_1833_separerad

Feature	Result
Variable type	factor
Number of missing obs.	0 (0 %)
Number of unique values	2
Mode	“0”
Reference category	0

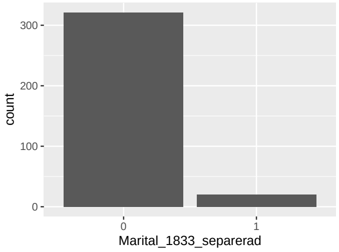

- Observed factor levels: "0", "1".

Marital_1833_singel

Feature	Result
Variable type	factor
Number of missing obs.	0 (0 %)
Number of unique values	2
Mode	“0”
Reference category	0

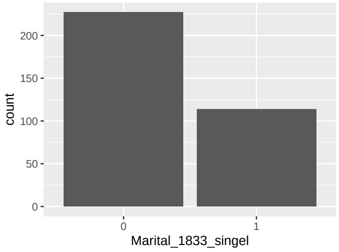

- Observed factor levels: "0", "1".

Edu_1843_2

Feature	Result
Variable type	factor
Number of missing obs.	0 (0 %)
Number of unique values	2
Mode	“0”
Reference category	0

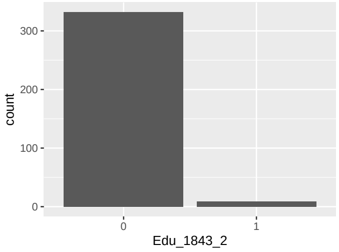

- Observed factor levels: "0", "1".

Edu_1843_3

Feature	Result
Variable type	factor
Number of missing obs.	0 (0 %)
Number of unique values	2
Mode	“0”
Reference category	0

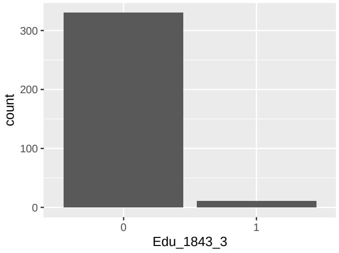

- Observed factor levels: "0", "1".

Edu_1843_4

Feature	Result
Variable type	factor
Number of missing obs.	0 (0 %)
Number of unique values	2
Mode	“0”
Reference category	0

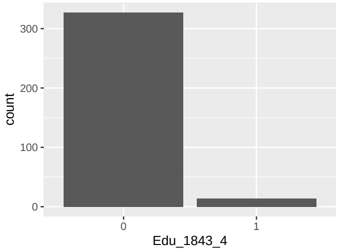

- Observed factor levels: "0", "1".

Edu_1843_5

Feature	Result
Variable type	factor
Number of missing obs.	0 (0 %)
Number of unique values	2
Mode	“0”
Reference category	0

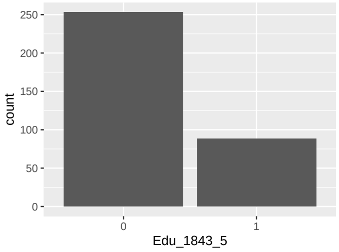

- Observed factor levels: "0", "1".

Edu_1843_6

Feature	Result
Variable type	factor
Number of missing obs.	0 (0 %)
Number of unique values	2
Mode	“0”
Reference category	0

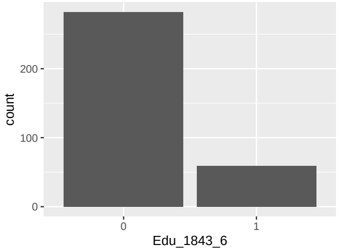

- Observed factor levels: "0", "1".

Edu_1843_7

Feature	Result
Variable type	factor
Number of missing obs.	0 (0 %)
Number of unique values	2
Mode	“0”
Reference category	0

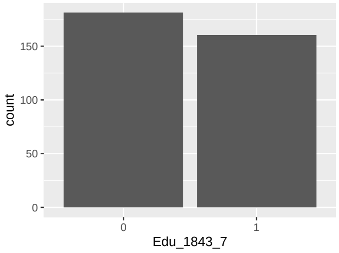

- Observed factor levels: "0", "1".

---

cscale

Feature	Result
Variable type	numeric
Number of missing obs.	0 (0 %)
Number of unique values	38
Median	0.25
1st and 3rd quartiles	-0.46; 0.72
Min. and max.	-2.6; 1.79

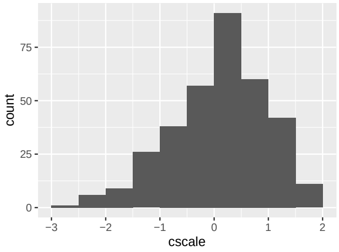

---

mainsymptom_PRE_sum

Feature	Result
Variable type	numeric
Number of missing obs.	0 (0 %)
Number of unique values	108
Median	0.27
1st and 3rd quartiles	-0.28; 0.89
Min. and max.	-2.37; 2.87

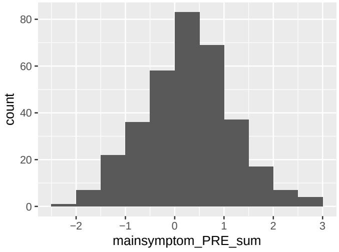

---

mainsymptom_PRE_duration

Feature	Result
Variable type	numeric
Number of missing obs.	0 (0 %)
Number of unique values	310
Median	-0.11
1st and 3rd quartiles	-0.2; 0.02
Min. and max.	-0.36; 7.79

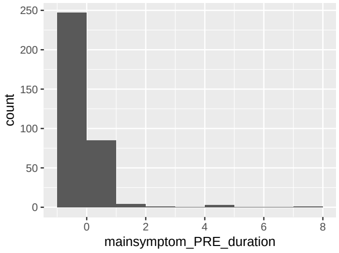

---

mainsymptom_PRE_DateCompleted_day

Feature	Result
Variable type	numeric
Number of missing obs.	0 (0 %)
Number of unique values	7
Median	0.37
1st and 3rd quartiles	-0.37; 1
Min. and max.	-1.37; 1.37

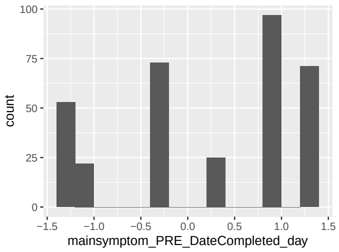

mainsymptom_PRE_DateCompleted_time

Feature	Result
Variable type	numeric
Number of missing obs.	0 (0 %)
Number of unique values	276
Median	-0.64
1st and 3rd quartiles	-1.22; 0.15
Min. and max.	-1.41; 1.35

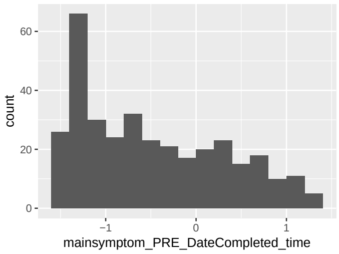

mainsymptom_WEEK01_sum

Feature	Result
Variable type	numeric
Number of missing obs.	0 (0 %)
Number of unique values	107
Median	0.19
1st and 3rd quartiles	-0.3; 0.78
Min. and max.	-2.22; 2.87

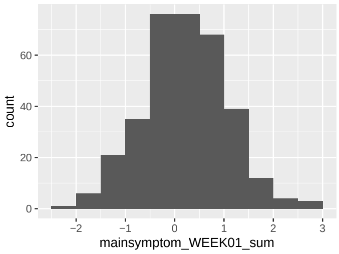

mainsymptom_WEEK01_duration

Feature	Result
Variable type	numeric
Number of missing obs.	0 (0 %)
Number of unique values	271
Median	-0.11
1st and 3rd quartiles	-0.17; 0.02
Min. and max.	-0.51; 4.27

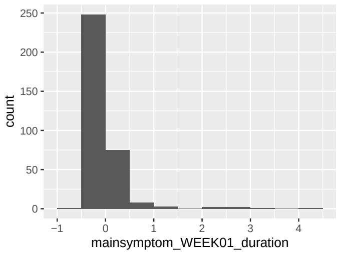

mainsymptom_WEEK01_DateCompleted_day

Feature	Result
Variable type	numeric
Number of missing obs.	0 (0 %)
Number of unique values	7
Median	1
1st and 3rd quartiles	-0.37; 1
Min. and max.	-1.37; 1.37

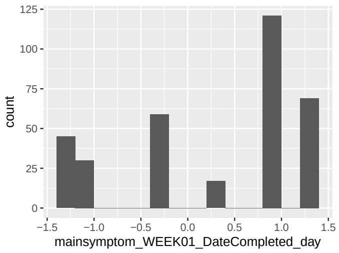

---

mainsymptom_WEEK01_DateCompleted_time

Feature	Result
Variable type	numeric
Number of missing obs.	0 (0 %)
Number of unique values	279
Median	-0.64
1st and 3rd quartiles	-1.21; 0.2
Min. and max.	-1.41; 1.41

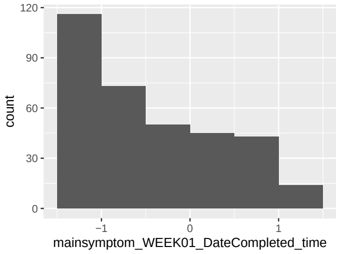

---

mainsymptom_WEEK02_sum

Feature	Result
Variable type	numeric
Number of missing obs.	0 (0 %)
Number of unique values	108
Median	0.08
1st and 3rd quartiles	-0.52; 0.71
Min. and max.	-2.18; 2.93

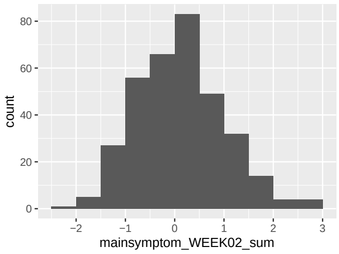

---

mainsymptom_WEEK02_duration

Feature	Result
Variable type	numeric
Number of missing obs.	0 (0 %)
Number of unique values	282
Median	-0.1
1st and 3rd quartiles	-0.18; -0.01
Min. and max.	-0.34; 3.7

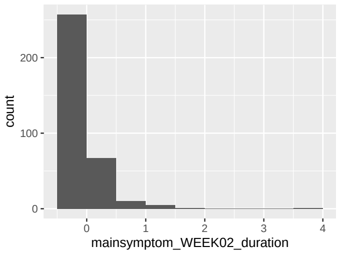

---

mainsymptom_WEEK02_DateCompleted_day

Feature	Result
Variable type	numeric
Number of missing obs.	0 (0 %)
Number of unique values	7
Median	1
1st and 3rd quartiles	-0.37; 1
Min. and max.	-1.37; 1.37

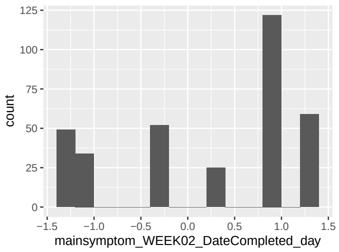

mainsymptom_WEEK02_DateCompleted_time

Feature	Result
Variable type	numeric
Number of missing obs.	0 (0 %)
Number of unique values	272
Median	-0.71
1st and 3rd quartiles	-1.21; 0.07
Min. and max.	-1.41; 1.39

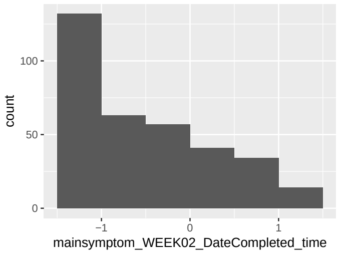

mainsymptom_WEEK03_sum

Feature	Result
Variable type	numeric
Number of missing obs.	0 (0 %)
Number of unique values	109
Median	-0.01
1st and 3rd quartiles	-0.6; 0.71
Min. and max.	-2.08; 3.07

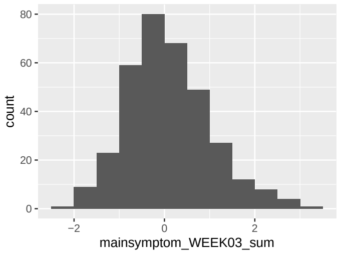

mainsymptom_WEEK03_duration

Feature	Result
Variable type	numeric
Number of missing obs.	0 (0 %)
Number of unique values	269
Median	-0.1
1st and 3rd quartiles	-0.15; -0.04
Min. and max.	-0.25; 3.76

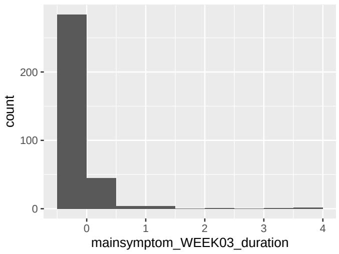

mainsymptom_WEEK03_DateCompleted_day

Feature	Result
Variable type	numeric
Number of missing obs.	0 (0 %)
Number of unique values	7
Median	1
1st and 3rd quartiles	-0.37; 1
Min. and max.	-1.37; 1.37

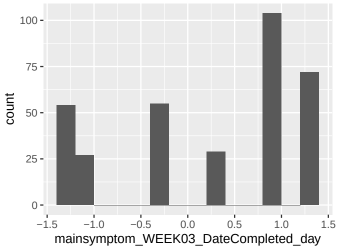

---

mainsymptom_WEEK03_DateCompleted_time

Feature	Result
Variable type	numeric
Number of missing obs.	0 (0 %)
Number of unique values	282
Median	-0.74
1st and 3rd quartiles	-1.24; 0.06
Min. and max.	-1.41; 1.41

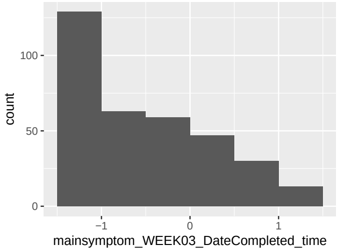

Report generation information:

- Created by: Could not determine from system (username: `nilisa`).
- Report creation time: Mon Jan 09 2023 13:11:33
- Report was run from directory: `/home/nilisa/projects/phd_study1/r`
- dataMaid v1.4.1 [Pkg: 2021-10-08 from CRAN (R 4.2.2)]
- R version 4.2.2 Patched (2022-11-10 r83330).
- Platform: x86_64-pc-linux-gnu (64-bit)(Ubuntu 20.04.5 LTS).
- Function call: `dataMaid::makeDataReport(data = gd, mode = c("summarize", "visualize", "check"), smartNum = FALSE, file = "~/projects/data/study1multiverse/results/graphs_n_figures/codebooks/codebook replace = TRUE, openResult = FALSE, checks = list(character = "showAllFactorLevels", factor = "showAllFactorLevels", labelled = "showAllFactorLevels",   haven_labelled = "showAllFactorLevels", numeric = NULL,   integer = NULL, logical = NULL, Date = NULL), listChecks = FALSE, maxProbVals = Inf, codebook = TRUE, reportTitle = "Handpicked_All_week04-naremove`

Handpicked_All_week04-naremove_train

Autogenerated data summary from dataMaid

2023-01-09 13:05:06

Data report overview

The dataset examined has the following dimensions:

Feature	Result
Number of observations	3094
Number of variables	63

Codebook summary table

Label	Variable	Class	# unique values	Missing	Description
	**sex**	factor	2	0.00 %	Sex of patient, 0 = Female, 1=Male
	**age**	numeric	65	0.00 %	
	**messages_len_7**	numeric	540	0.00 %	-Meta information of messages-Length of messages-up until day-7
	**messages_len_tp_7**	numeric	998	0.00 %	-Meta information of messages-Length of messages-therapist messages-up until day-7
	**messages_7**	numeric	10	0.00 %	-Meta information of messages-up until day-7
	**messages_tp_7**	numeric	8	0.00 %	-Meta information of messages-therapist messages-up until day-7
	**homeworks_7**	numeric	7	0.00 %	-Number of homework messages sent in-up until day-7
	**messages_len_14**	numeric	576	0.00 %	-Meta information of messages-Length of messages-up until day-14
	**messages_len_tp_14**	numeric	1016	0.00 %	-Meta information of messages-Length of messages-therapist messages-up until day-14
	**messages_14**	numeric	10	0.00 %	-Meta information of messages-up until day-14
	**messages_tp_14**	numeric	7	0.00 %	-Meta information of messages-therapist messages-up until day-14

Label	Variable	Class	# unique values	Missing	Description
	**homeworks_14**	numeric	6	0.00 %	-Number of homework messages sent in-up until day-14
	**messages_len_21**	numeric	561	0.00 %	-Meta information of messages-Length of messages-up until day-21
	**messages_len_tp_21**	numeric	1023	0.00 %	-Meta information of messages-Length of messages-therapist messages-up until day-21
	**messages_21**	numeric	10	0.00 %	-Meta information of messages-up until day-21
	**messages_tp_21**	numeric	7	0.00 %	-Meta information of messages-therapist messages-up until day-21
	**homeworks_21**	numeric	6	0.00 %	-Number of homework messages sent in-up until day-21
	**messages_len_28**	numeric	518	0.00 %	-Meta information of messages-Length of messages-up until day-28
	**messages_len_tp_28**	numeric	966	0.00 %	-Meta information of messages-Length of messages-therapist messages-up until day-28
	**messages_28**	numeric	9	0.00 %	-Meta information of messages-up until day-28
	**messages_tp_28**	numeric	7	0.00 %	-Meta information of messages-therapist messages-up until day-28
	**homeworks_28**	numeric	7	0.00 %	-Number of homework messages sent in-up until day-28
	**PDSS-SR-3064_SCREEN_sum**	numeric	28	0.00 %	Anxiety questionnaire, self rated-Timepoint before treatment starts-Sum of the entire measure
	**MADRS-1951_SCREEN_sum**	numeric	44	0.00 %	Depression questionnaire, self rated-Timepoint before treatment starts-Sum of the entire measure
	**LSAS-2241_SCREEN_sum**	numeric	133	0.00 %	Social anxiety questionnaire, self rated-Timepoint before treatment starts-Sum of the entire measure
	**MADRS-1951_SCREEN_DateCompleted_day**	numeric	7	0.00 %	Depression questionnaire, self rated-Timepoint before treatment starts-Cyclic transformation of what day 0-6 during week it was filled in

Label	Variable	Class	# unique values	Missing	Description
	**MADRS-1951_SCREEN_DateCompleted_time**	numeric	897	0.00 %	Depression questionnaire, self rated-Timepoint before treatment starts-Cyclic transformation of what time during day 0-1440 it was filled in
	**PDSS-SR-3064_SCREEN_DateCompleted_day**	numeric	7	0.00 %	Anxiety questionnaire, self rated-Timepoint before treatment starts-Cyclic transformation of what day 0-6 during week it was filled in
	**PDSS-SR-3064_SCREEN_DateCompleted_time**	numeric	894	0.00 %	Anxiety questionnaire, self rated-Timepoint before treatment starts-Cyclic transformation of what time during day 0-1440 it was filled in
	**LSAS-2241_SCREEN_DateCompleted_day**	numeric	7	0.00 %	Social anxiety questionnaire, self rated-Timepoint before treatment starts-Cyclic transformation of what day 0-6 during week it was filled in
	**LSAS-2241_SCREEN_DateCompleted_time**	numeric	904	0.00 %	Social anxiety questionnaire, self rated-Timepoint before treatment starts-Cyclic transformation of what time during day 0-1440 it was filled in
	**Depression**	numeric	2	0.00 %	
	**Panic**	numeric	2	0.00 %	
	**Social_Anxiety**	numeric	2	0.00 %	
	**outcome**	numeric	182	0.00 %	
	**ncomorbid**	numeric	5	0.00 %	
	**currentwork_proff**	factor	2	0.00 %	Currently in work for trained proffession
	**Marital_1833_gift**	factor	2	0.00 %	Marital status: Married or not
	**Marital_1833_separerad**	factor	2	0.00 %	Marital status: divocered/equivalent
	**Marital_1833_singel**	factor	2	0.00 %	Marital status: single
	**Edu_1843_2**	factor	2	0.00 %	7-9 years education
	**Edu_1843_3**	factor	2	0.00 %	Uncompleted upper secondary school
	**Edu_1843_4**	factor	2	0.00 %	Higher vocational education
	**Edu_1843_5**	factor	2	0.00 %	Completed upper secondary school
	**Edu_1843_6**	factor	2	0.00 %	Uncompleted university degree
	**Edu_1843_7**	factor	2	0.00 %	University degree
	**cscale**	numeric	47	0.00 %	
	**mainsymptom_PRE_sum**	numeric	179	0.00 %	PDSS-SR for panic, MADRS for depression, LSAS for social anxiety-Timepoint just before beginning treatment-Sum of the entire measure

Label	Variable	Class	# unique values	Missing	Description
	**mainsymptom_PRE_duration**	numeric	1679	0.00 %	PDSS-SR for panic, MADRS for depression, LSAS for social anxiety-Timepoint just before beginning treatment-Time to fill in measure/questionnaire
	**mainsymptom_PRE_DateCompleted_day**	numeric	7	0.00 %	PDSS-SR for panic, MADRS for depression, LSAS for social anxiety-Timepoint just before beginning treatment-Cyclic transformation of what day 0-6 during week it was filled in
	**mainsymptom_PRE_DateCompleted_time**	numeric	855	0.00 %	PDSS-SR for panic, MADRS for depression, LSAS for social anxiety-Timepoint just before beginning treatment-Cyclic transformation of what time during day 0-1440 it was filled in
	**mainsymptom_WEEK01_sum**	numeric	182	0.00 %	PDSS-SR for panic, MADRS for depression, LSAS for social anxiety-Timepoint after one week in treatment-Sum of the entire measure
	**mainsymptom_WEEK01_duration**	numeric	1162	0.00 %	PDSS-SR for panic, MADRS for depression, LSAS for social anxiety-Timepoint after one week in treatment-Time to fill in measure/questionnaire
	**mainsymptom_WEEK01_DateCompleted_day**	numeric	7	0.00 %	PDSS-SR for panic, MADRS for depression, LSAS for social anxiety-Timepoint after one week in treatment-Cyclic transformation of what day 0-6 during week it was filled in
	**mainsymptom_WEEK01_DateCompleted_time**	numeric	928	0.00 %	PDSS-SR for panic, MADRS for depression, LSAS for social anxiety-Timepoint after one week in treatment-Cyclic transformation of what time during day 0-1440 it was filled in
	**mainsymptom_WEEK02_sum**	numeric	185	0.00 %	PDSS-SR for panic, MADRS for depression, LSAS for social anxiety-Timepoint after two weeks in treatment-Sum of the entire measure
	**mainsymptom_WEEK02_duration**	numeric	1103	0.00 %	PDSS-SR for panic, MADRS for depression, LSAS for social anxiety-Timepoint after two weeks in treatment-Time to fill in measure/questionnaire

Label	Variable	Class	# unique values	Missing	Description
	**mainsymptom_WEEK02_DateCompleted_day**	numeric	7	0.00 %	PDSS-SR for panic, MADRS for depression, LSAS for social anxiety-Timepoint after two weeks in treatment-Cyclic transformation of what day 0-6 during week it was filled in
	**mainsymptom_WEEK02_DateCompleted_time**	numeric	934	0.00 %	PDSS-SR for panic, MADRS for depression, LSAS for social anxiety-Timepoint after two weeks in treatment-Cyclic transformation of what time during day 0-1440 it was filled in
	**mainsymptom_WEEK03_sum**	numeric	183	0.00 %	PDSS-SR for panic, MADRS for depression, LSAS for social anxiety-Timepoint after three weeks in treatment-Sum of the entire measure
	**mainsymptom_WEEK03_duration**	numeric	1058	0.00 %	PDSS-SR for panic, MADRS for depression, LSAS for social anxiety-Timepoint after three weeks in treatment-Time to fill in measure/questionnaire
	**mainsymptom_WEEK03_DateCompleted_day**	numeric	7	0.00 %	PDSS-SR for panic, MADRS for depression, LSAS for social anxiety-Timepoint after three weeks in treatment-Cyclic transformation of what day 0-6 during week it was filled in
	**mainsymptom_WEEK03_DateCompleted_time**	numeric	956	0.00 %	PDSS-SR for panic, MADRS for depression, LSAS for social anxiety-Timepoint after three weeks in treatment-Cyclic transformation of what time during day 0-1440 it was filled in

Variable list

sex

Feature	Result
Variable type	factor
Number of missing obs.	0 (0 %)
Number of unique values	2
Mode	“0”
Reference category	0

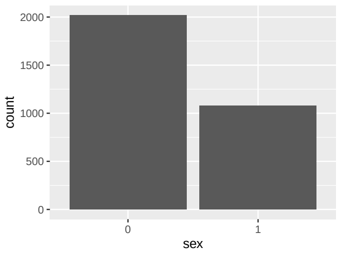

- Observed factor levels: "0", "1".

---

age

Feature	Result
Variable type	numeric
Number of missing obs.	0 (0 %)
Number of unique values	65
Median	-0.2
1st and 3rd quartiles	-0.73; 0.67
Min. and max.	-1.69; 4.25

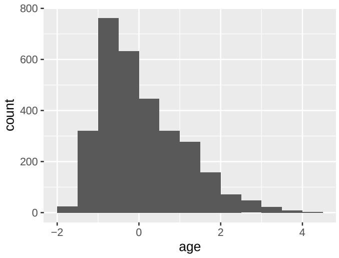

---

messages_len_7

Feature	Result
Variable type	numeric
Number of missing obs.	0 (0 %)
Number of unique values	540
Median	-0.32
1st and 3rd quartiles	-0.32; 0.13
Min. and max.	-0.32; 35.05

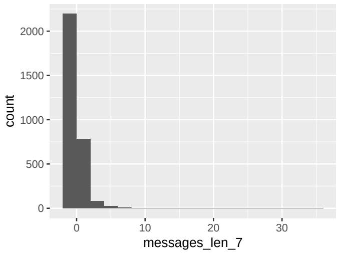

---

messages_len_tp_7

Feature	Result
Variable type	numeric
Number of missing obs.	0 (0 %)
Number of unique values	998
Median	0.2
1st and 3rd quartiles	-0.45; 0.89
Min. and max.	-1.46; 9.81

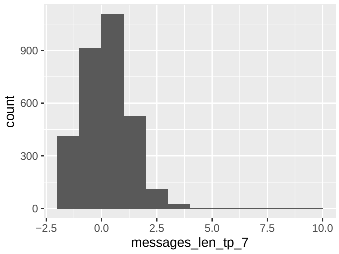

---

messages_7

Feature	Result
Variable type	numeric
Number of missing obs.	0 (0 %)
Number of unique values	10
Median	-0.55
1st and 3rd quartiles	-0.55; 0.58
Min. and max.	-0.55; 16.49

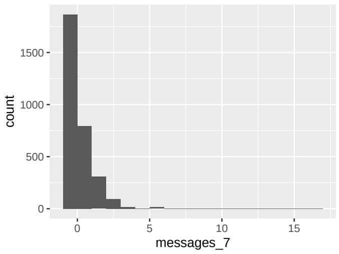

---

messages_tp_7

Feature	Result
Variable type	numeric
Number of missing obs.	0 (0 %)
Number of unique values	8
Median	0.15
1st and 3rd quartiles	0.15; 1.21
Min. and max.	-1.97; 6.52

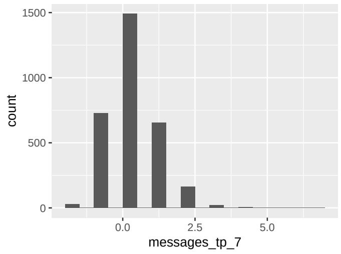

homeworks_7

Feature	Result
Variable type	numeric
Number of missing obs.	0 (0 %)
Number of unique values	7
Median	0.2
1st and 3rd quartiles	0.2; 0.2
Min. and max.	-1.03; 6.39

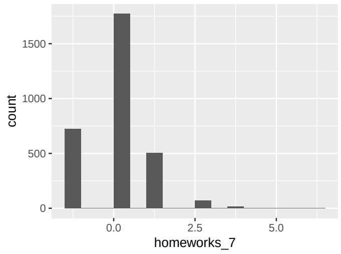

messages_len_14

Feature	Result
Variable type	numeric
Number of missing obs.	0 (0 %)
Number of unique values	576
Median	-0.3
1st and 3rd quartiles	-0.41; 0.21
Min. and max.	-0.41; 10.98

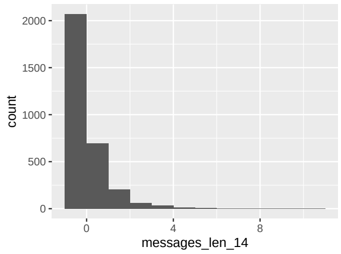

messages_len_tp_14

Feature	Result
Variable type	numeric
Number of missing obs.	0 (0 %)
Number of unique values	1016
Median	0.09
1st and 3rd quartiles	-0.51; 0.72
Min. and max.	-1.11; 14.98

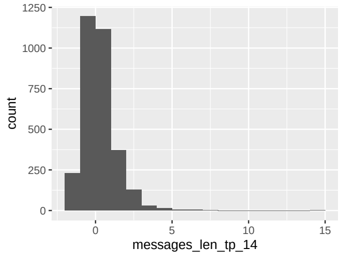

messages_14

Feature	Result
Variable type	numeric
Number of missing obs.	0 (0 %)
Number of unique values	10
Median	0.36
1st and 3rd quartiles	-0.7; 0.36
Min. and max.	-0.7; 9.9

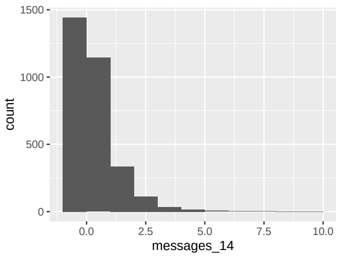

messages_tp_14

Feature	Result
Variable type	numeric
Number of missing obs.	0 (0 %)
Number of unique values	7
Median	-0.35
1st and 3rd quartiles	-0.35; 0.87
Min. and max.	-1.57; 5.75

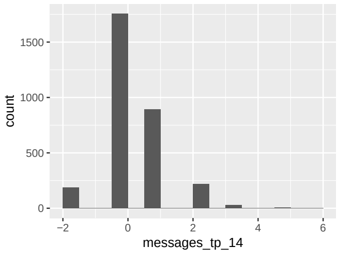

homeworks_14

Feature	Result
Variable type	numeric
Number of missing obs.	0 (0 %)
Number of unique values	6
Median	0.33
1st and 3rd quartiles	-0.98; 0.33
Min. and max.	-0.98; 5.58

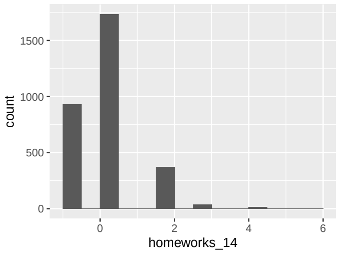

messages_len_21

Feature	Result
Variable type	numeric
Number of missing obs.	0 (0 %)
Number of unique values	561
Median	-0.28
1st and 3rd quartiles	-0.46; 0.24
Min. and max.	-0.46; 13.29

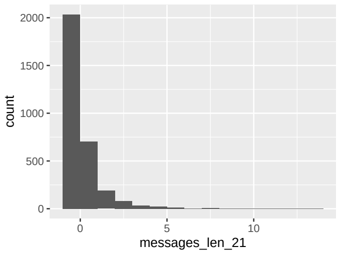

messages_len_tp_21

Feature	Result
Variable type	numeric
Number of missing obs.	0 (0 %)
Number of unique values	1023
Median	0.06
1st and 3rd quartiles	-0.5; 0.77
Min. and max.	-1.01; 8.85

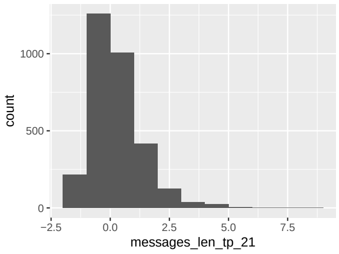

messages_21

Feature	Result
Variable type	numeric
Number of missing obs.	0 (0 %)
Number of unique values	10
Median	0.34
1st and 3rd quartiles	-0.74; 0.34
Min. and max.	-0.74; 8.99

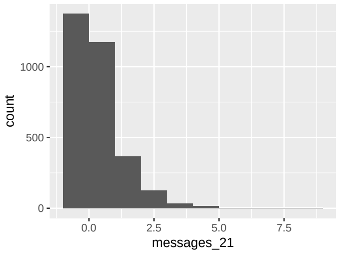

messages_tp_21

Feature	Result
Variable type	numeric
Number of missing obs.	0 (0 %)
Number of unique values	7
Median	-0.32
1st and 3rd quartiles	-0.32; 0.84
Min. and max.	-1.49; 5.5

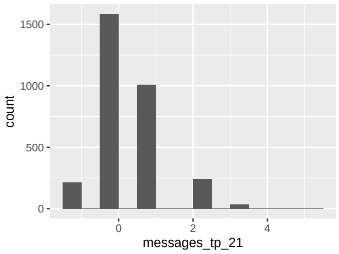

homeworks_21

Feature	Result
Variable type	numeric
Number of missing obs.	0 (0 %)
Number of unique values	6
Median	0.36
1st and 3rd quartiles	-1.01; 0.36
Min. and max.	-1.01; 5.86

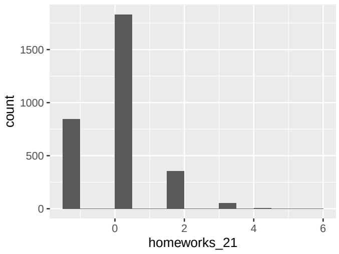

messages_len_28

Feature	Result
Variable type	numeric
Number of missing obs.	0 (0 %)
Number of unique values	518
Median	-0.3
1st and 3rd quartiles	-0.41; 0.19
Min. and max.	-0.41; 13.67

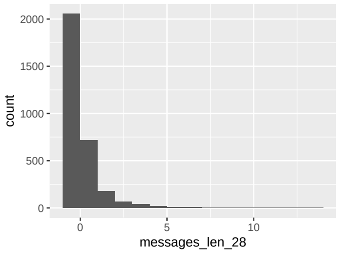

messages_len_tp_28

Feature	Result
Variable type	numeric
Number of missing obs.	0 (0 %)
Number of unique values	966
Median	0.04
1st and 3rd quartiles	-0.49; 0.75
Min. and max.	-0.94; 10.31

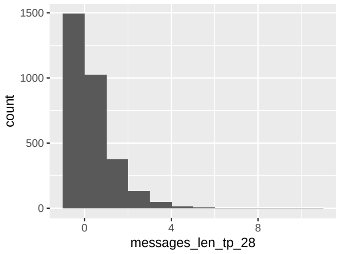

messages_28

Feature	Result
Variable type	numeric
Number of missing obs.	0 (0 %)
Number of unique values	9
Median	0.41
1st and 3rd quartiles	-0.72; 0.41
Min. and max.	-0.72; 10.59

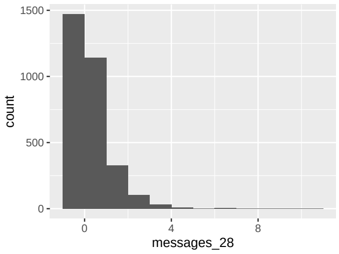

messages_tp_28

Feature	Result
Variable type	numeric
Number of missing obs.	0 (0 %)
Number of unique values	7
Median	-0.24
1st and 3rd quartiles	-0.24; 0.93
Min. and max.	-1.42; 5.63

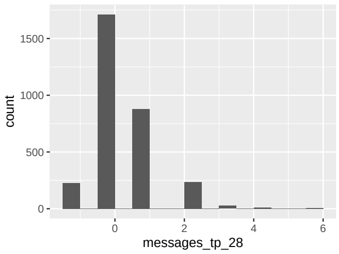

homeworks_28

Feature	Result
Variable type	numeric
Number of missing obs.	0 (0 %)
Number of unique values	7
Median	0.46
1st and 3rd quartiles	-0.91; 0.46
Min. and max.	-0.91; 7.31

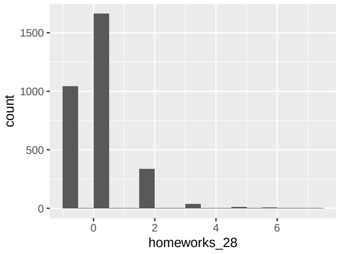

PDSS-SR-3064_SCREEN_sum

Feature	Result
Variable type	numeric
Number of missing obs.	0 (0 %)
Number of unique values	28
Median	-0.03
1st and 3rd quartiles	-0.98; 0.75
Min. and max.	-1.29; 3.12

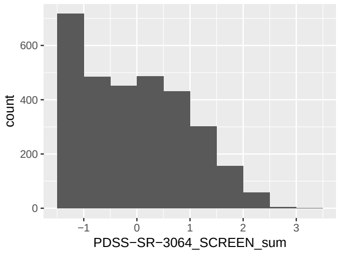

MADRS-1951_SCREEN_sum

Feature	Result
Variable type	numeric
Number of missing obs.	0 (0 %)
Number of unique values	44
Median	0.05
1st and 3rd quartiles	-0.7; 0.67
Min. and max.	-2.68; 2.65

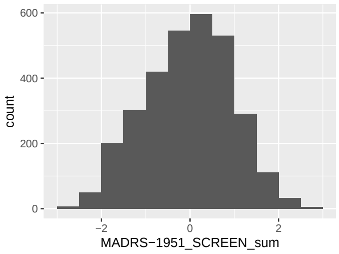

LSAS-2241_SCREEN_sum

Feature	Result
Variable type	numeric
Number of missing obs.	0 (0 %)
Number of unique values	133
Median	-0.09
1st and 3rd quartiles	-0.82; 0.67
Min. and max.	-1.79; 3.02

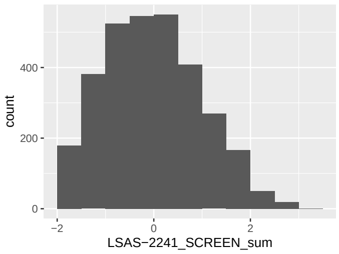

MADRS-1951_SCREEN_DateCompleted_day

Feature	Result
Variable type	numeric
Number of missing obs.	0 (0 %)
Number of unique values	7
Median	0.37
1st and 3rd quartiles	-1; 1
Min. and max.	-1.37; 1.37

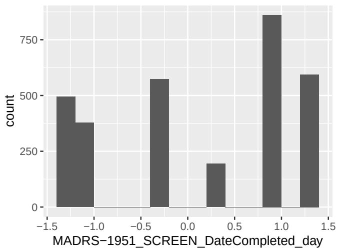

MADRS-1951_SCREEN_DateCompleted_time

Feature	Result
Variable type	numeric
Number of missing obs.	0 (0 %)
Number of unique values	897
Median	-0.74
1st and 3rd quartiles	-1.24; 0.25
Min. and max.	-1.41; 1.41

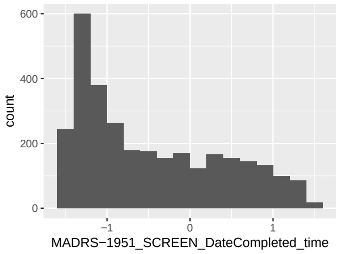

PDSS-SR-3064_SCREEN_DateCompleted_day

Feature	Result
Variable type	numeric
Number of missing obs.	0 (0 %)
Number of unique values	7
Median	0.37
1st and 3rd quartiles	-1; 1
Min. and max.	-1.37; 1.37

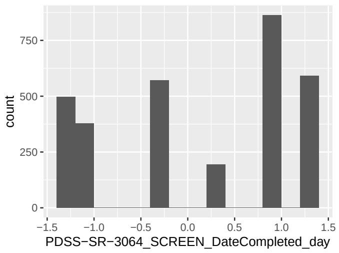

PDSS-SR-3064_SCREEN_DateCompleted_time

Feature	Result
Variable type	numeric
Number of missing obs.	0 (0 %)
Number of unique values	894
Median	-0.73
1st and 3rd quartiles	-1.24; 0.27
Min. and max.	-1.41; 1.41

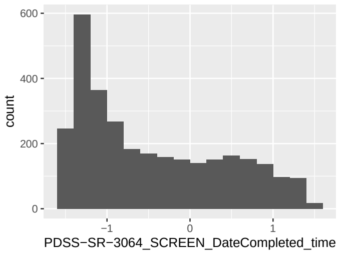

LSAS-2241_SCREEN_DateCompleted_day

Feature	Result
Variable type	numeric
Number of missing obs.	0 (0 %)
Number of unique values	7
Median	0.37
1st and 3rd quartiles	-1; 1
Min. and max.	-1.37; 1.37

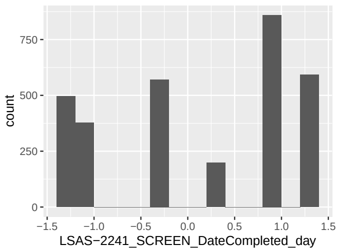

LSAS-2241_SCREEN_DateCompleted_time

Feature	Result
Variable type	numeric
Number of missing obs.	0 (0 %)
Number of unique values	904
Median	-0.73
1st and 3rd quartiles	-1.23; 0.31
Min. and max.	-1.41; 1.41

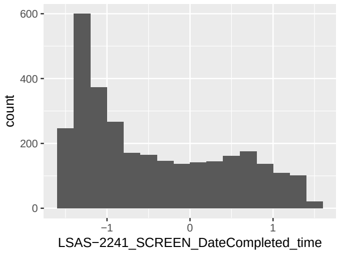

Depression

Feature	Result
Variable type	numeric
Number of missing obs.	0 (0 %)
Number of unique values	2
Median	0
1st and 3rd quartiles	0; 1
Min. and max.	0; 1

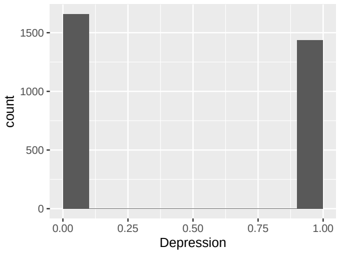

Panic

Feature	Result
Variable type	numeric
Number of missing obs.	0 (0 %)
Number of unique values	2
Median	0
1st and 3rd quartiles	0; 0
Min. and max.	0; 1

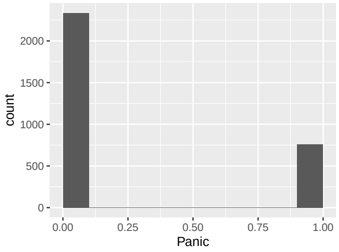

Social_Anxiety

Feature	Result
Variable type	numeric
Number of missing obs.	0 (0 %)
Number of unique values	2
Median	0
1st and 3rd quartiles	0; 1
Min. and max.	0; 1

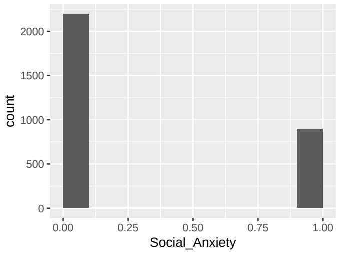

---

outcome

Feature	Result
Variable type	numeric
Number of missing obs.	0 (0 %)
Number of unique values	182
Median	-0.18
1st and 3rd quartiles	-0.77; 0.53
Min. and max.	-2.05; 4.04

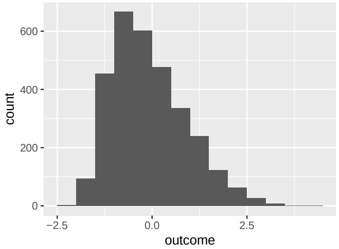

---

ncomorbid

Feature	Result
Variable type	numeric
Number of missing obs.	0 (0 %)
Number of unique values	5
Median	0
1st and 3rd quartiles	0; 1
Min. and max.	0; 4

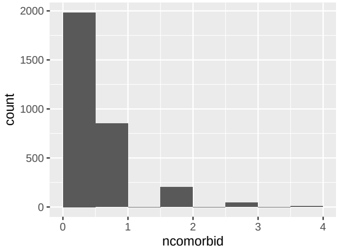

---

currentwork_proff

Feature	Result
Variable type	factor
Number of missing obs.	0 (0 %)
Number of unique values	2
Mode	"1"
Reference category	0

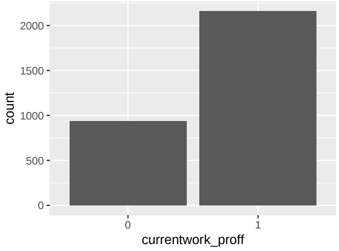

- Observed factor levels: "0", "1".

Marital_1833_gift

Feature	Result
Variable type	factor
Number of missing obs.	0 (0 %)
Number of unique values	2
Mode	“1”
Reference category	0

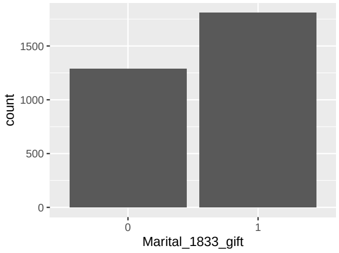

- Observed factor levels: "0", "1".

Marital_1833_separerad

Feature	Result
Variable type	factor
Number of missing obs.	0 (0 %)
Number of unique values	2
Mode	“0”
Reference category	0

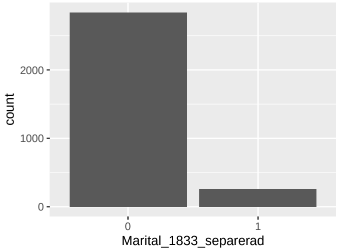

- Observed factor levels: "0", "1".

Marital_1833_singel

Feature	Result
Variable type	factor
Number of missing obs.	0 (0 %)
Number of unique values	2
Mode	“0”
Reference category	0

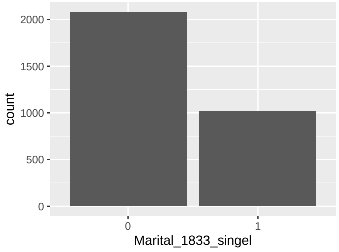

- Observed factor levels: "0", "1".

Edu_1843_2

Feature	Result
Variable type	factor
Number of missing obs.	0 (0 %)
Number of unique values	2
Mode	“0”
Reference category	0

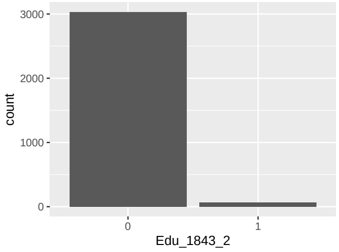

- Observed factor levels: "0", "1".

Edu_1843_3

Feature	Result
Variable type	factor
Number of missing obs.	0 (0 %)
Number of unique values	2
Mode	“0”
Reference category	0

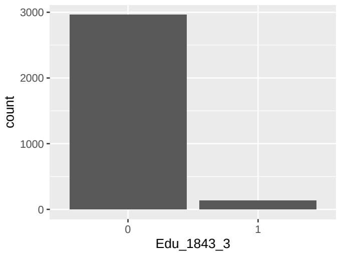

- Observed factor levels: "0", "1".

Edu_1843_4

Feature	Result
Variable type	factor
Number of missing obs.	0 (0 %)
Number of unique values	2
Mode	“0”
Reference category	0

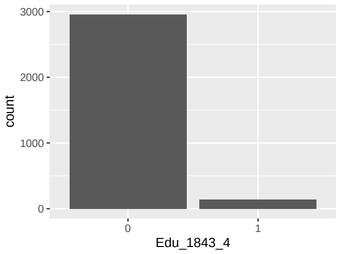

- Observed factor levels: "0", "1".

Edu_1843_5

Feature	Result
Variable type	factor
Number of missing obs.	0 (0 %)
Number of unique values	2
Mode	“0”
Reference category	0

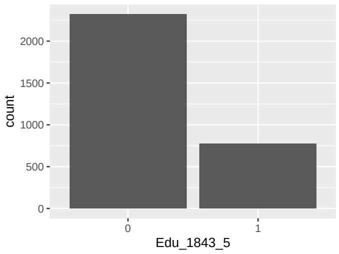

- Observed factor levels: "0", "1".

Edu_1843_6

Feature	Result
Variable type	factor
Number of missing obs.	0 (0 %)
Number of unique values	2
Mode	“0”
Reference category	0

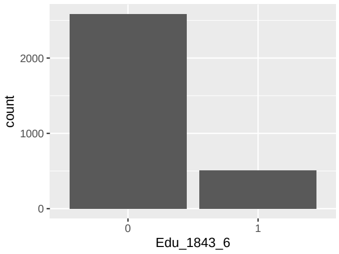

- Observed factor levels: "0", "1".

Edu_1843_7

Feature	Result
Variable type	factor
Number of missing obs.	0 (0 %)
Number of unique values	2
Mode	"0"
Reference category	0

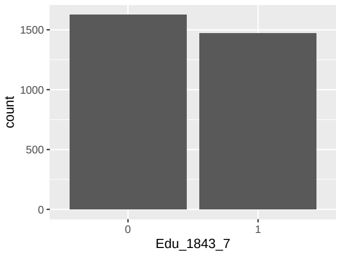

- Observed factor levels: "0", "1".

---

cscale

Feature	Result
Variable type	numeric
Number of missing obs.	0 (0 %)
Number of unique values	47
Median	0.25
1st and 3rd quartiles	-0.46; 0.84
Min. and max.	-4.14; 1.79

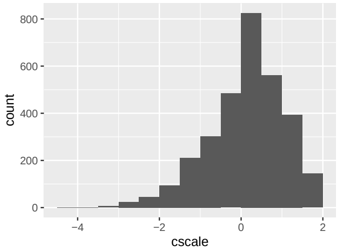

---

mainsymptom_PRE_sum

Feature	Result
Variable type	numeric
Number of missing obs.	0 (0 %)
Number of unique values	179
Median	0.27
1st and 3rd quartiles	-0.38; 0.89
Min. and max.	-2.49; 3

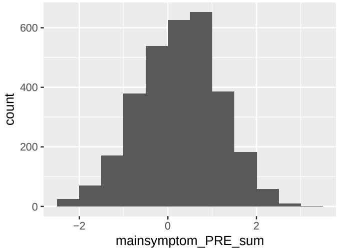

---

mainsymptom_PRE_duration

Feature	Result
Variable type	numeric
Number of missing obs.	0 (0 %)
Number of unique values	1679
Median	-0.11
1st and 3rd quartiles	-0.19; 0.01
Min. and max.	-0.44; 24.6

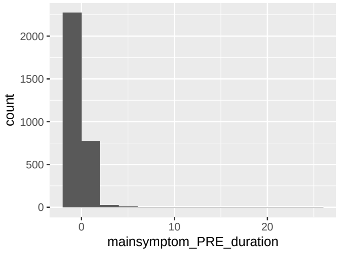

---

mainsymptom_PRE_DateCompleted_day

Feature	Result
Variable type	numeric
Number of missing obs.	0 (0 %)
Number of unique values	7
Median	1
1st and 3rd quartiles	-0.37; 1
Min. and max.	-1.37; 1.37

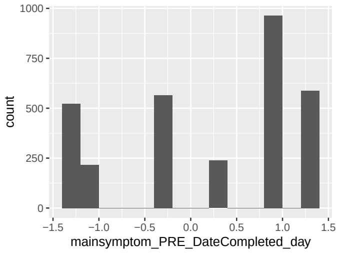

mainsymptom_PRE_DateCompleted_time

Feature	Result
Variable type	numeric
Number of missing obs.	0 (0 %)
Number of unique values	855
Median	-0.7
1st and 3rd quartiles	-1.23; 0.07
Min. and max.	-1.41; 1.41

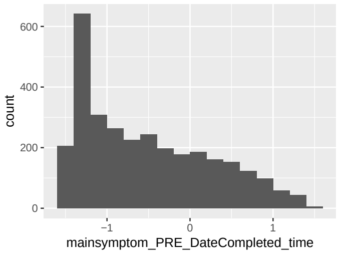

mainsymptom_WEEK01_sum

Feature	Result
Variable type	numeric
Number of missing obs.	0 (0 %)
Number of unique values	182
Median	0.19
1st and 3rd quartiles	-0.42; 0.82
Min. and max.	-2.84; 3.6

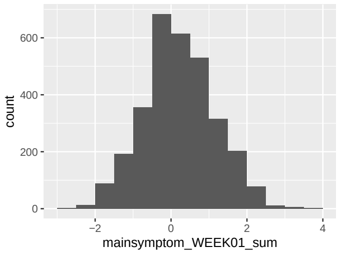

mainsymptom_WEEK01_duration

Feature	Result
Variable type	numeric
Number of missing obs.	0 (0 %)
Number of unique values	1162
Median	-0.1
1st and 3rd quartiles	-0.17; 0.01
Min. and max.	-0.46; 35.17

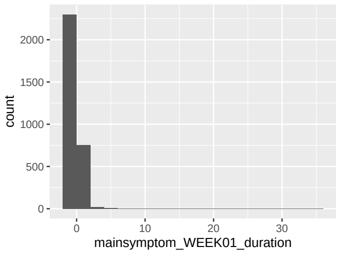

mainsymptom_WEEK01_DateCompleted_day

Feature	Result
Variable type	numeric
Number of missing obs.	0 (0 %)
Number of unique values	7
Median	1
1st and 3rd quartiles	-1; 1
Min. and max.	-1.37; 1.37

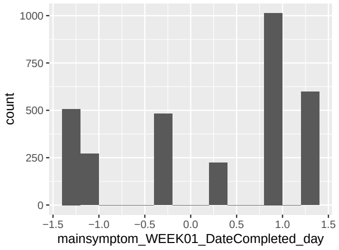

mainsymptom_WEEK01_DateCompleted_time

Feature	Result
Variable type	numeric
Number of missing obs.	0 (0 %)
Number of unique values	928
Median	-0.67
1st and 3rd quartiles	-1.21; 0.09
Min. and max.	-1.41; 1.41

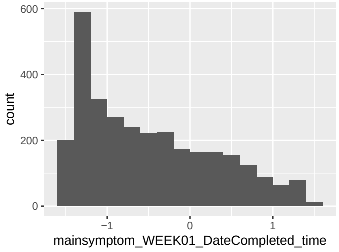

mainsymptom_WEEK02_sum

Feature	Result
Variable type	numeric
Number of missing obs.	0 (0 %)
Number of unique values	185
Median	0.09
1st and 3rd quartiles	-0.52; 0.74
Min. and max.	-2.75; 3.44

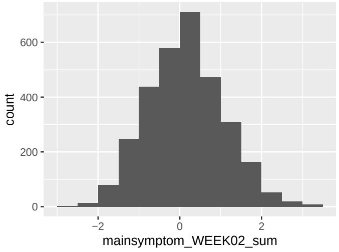

mainsymptom_WEEK02_duration

Feature	Result
Variable type	numeric
Number of missing obs.	0 (0 %)
Number of unique values	1103
Median	-0.1
1st and 3rd quartiles	-0.19; -0.01
Min. and max.	-0.46; 23.95

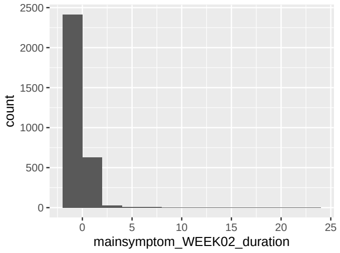

mainsymptom_WEEK02_DateCompleted_day

Feature	Result
Variable type	numeric
Number of missing obs.	0 (0 %)
Number of unique values	7
Median	1
1st and 3rd quartiles	-0.37; 1
Min. and max.	-1.37; 1.37

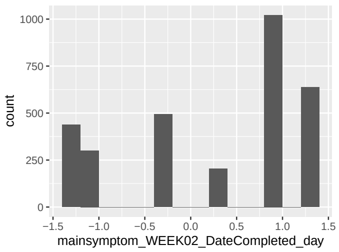

mainsymptom_WEEK02_DateCompleted_time

Feature	Result
Variable type	numeric
Number of missing obs.	0 (0 %)
Number of unique values	934
Median	-0.7
1st and 3rd quartiles	-1.2; 0.05
Min. and max.	-1.41; 1.41

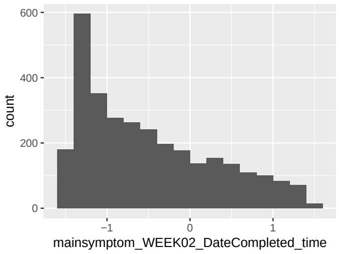

mainsymptom_WEEK03_sum

Feature	Result
Variable type	numeric
Number of missing obs.	0 (0 %)
Number of unique values	183
Median	0.09
1st and 3rd quartiles	-0.48; 0.82
Min. and max.	-2.64; 3.99

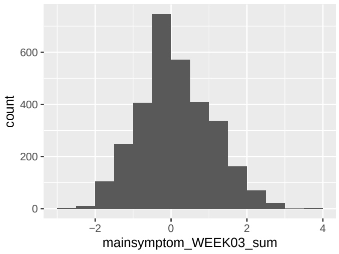

mainsymptom_WEEK03_duration

Feature	Result
Variable type	numeric
Number of missing obs.	0 (0 %)
Number of unique values	1058
Median	-0.09
1st and 3rd quartiles	-0.14; -0.02
Min. and max.	-0.27; 41.39

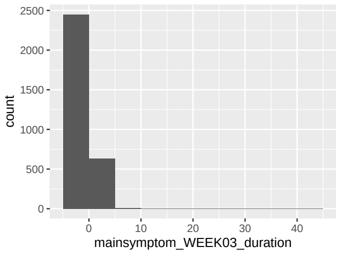

mainsymptom_WEEK03_DateCompleted_day

Feature	Result
Variable type	numeric
Number of missing obs.	0 (0 %)
Number of unique values	7
Median	1
1st and 3rd quartiles	-0.37; 1
Min. and max.	-1.37; 1.37

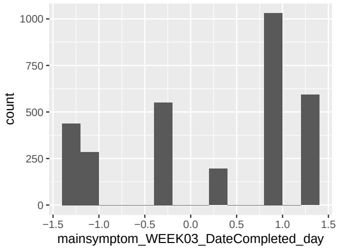

mainsymptom_WEEK03_DateCompleted_time

Feature	Result
Variable type	numeric
Number of missing obs.	0 (0 %)
Number of unique values	956
Median	-0.64
1st and 3rd quartiles	-1.18; 0.12
Min. and max.	-1.41; 1.41

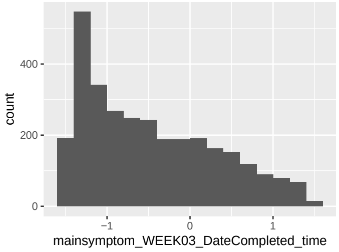

Report generation information:

- Created by: Could not determine from system (username: `nilisa`).
- Report creation time: Mon Jan 09 2023 13:05:07
- Report was run from directory: `/home/nilisa/projects/phd_study1/r`
- dataMaid v1.4.1 [Pkg: 2021-10-08 from CRAN (R 4.2.2)]
- R version 4.2.2 Patched (2022-11-10 r83330).
- Platform: x86_64-pc-linux-gnu (64-bit)(Ubuntu 20.04.5 LTS).
- Function call:

```
dataMaid::makeDataReport(data = gd, mode = c("summarize", "visualize",  "check"),
smartNum = FALSE, file = "~/projects/data/study1multiverse/results/graphs_n_figures/codebooks/codebook
replace = TRUE, openResult = FALSE, checks = list(character = "showAllFactorLevels",
factor = "showAllFactorLevels", labelled = "showAllFactorLevels",      haven_labelled =
"showAllFactorLevels", numeric = NULL,      integer = NULL, logical = NULL, Date = NULL),
listChecks = FALSE,  maxProbVals = Inf, codebook = TRUE, reportTitle = "Handpicked_All_week04-naremove
```

Handpicked_Depression_week04-imputed_benchmark_test

Autogenerated data summary from dataMaid

2023-01-09 13:12:14

Data report overview

The dataset examined has the following dimensions:

Feature	Result
Number of observations	308
Number of variables	10

Codebook summary table

Label	Variable	Class	# unique values	Missing	Description
	**sex**	factor	2	0.00 %	Sex of patient, 0 = Female, 1=Male
	**age**	numeric	54	0.00 %	
	**PDSS-SR-3064_SCREEN_sum**	numeric	47	0.00 %	Anxiety questionnaire, self rated-Timepoint before treatment starts-Sum of the entire measure
	**MADRS-1951_SCREEN_sum**	numeric	45	0.00 %	Depression questionnaire, self rated-Timepoint before treatment starts-Sum of the entire measure
	**LSAS-2241_SCREEN_sum**	numeric	139	0.00 %	Social anxiety questionnaire, self rated-Timepoint before treatment starts-Sum of the entire measure
	**outcome**	numeric	125	0.00 %	
	**mainsymptom_PRE_sum**	numeric	47	0.00 %	PDSS-SR for panic, MADRS for depression, LSAS for social anxiety-Timepoint just before beginning treatment-Sum of the entire measure
	**mainsymptom_WEEK01_sum**	numeric	101	0.00 %	PDSS-SR for panic, MADRS for depression, LSAS for social anxiety-Timepoint after one week in treatment-Sum of the entire measure
	**mainsymptom_WEEK02_sum**	numeric	96	0.00 %	PDSS-SR for panic, MADRS for depression, LSAS for social anxiety-Timepoint after two weeks in treatment-Sum of the entire measure

Label	Variable	Class	# unique values	Missing	Description
	**mainsymptom_WEEK03_sum**	numeric	107	0.00 %	PDSS-SR for panic, MADRS for depression, LSAS for social anxiety-Timepoint after three weeks in treatment-Sum of the entire measure

Variable list

sex

Feature	Result
Variable type	factor
Number of missing obs.	0 (0 %)
Number of unique values	2
Mode	"0"
Reference category	0

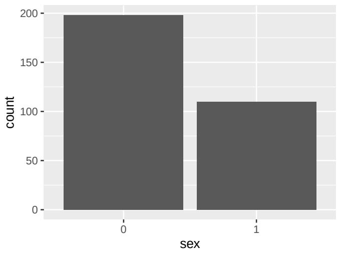

- Observed factor levels: "0", "1".

---

age

Feature	Result
Variable type	numeric
Number of missing obs.	0 (0 %)
Number of unique values	54
Median	0.1
1st and 3rd quartiles	-0.55; 0.85
Min. and max.	-1.6; 4.08

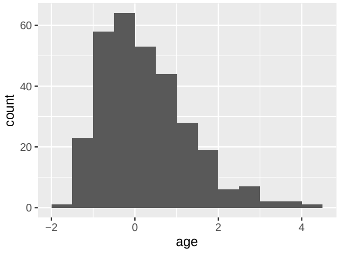

PDSS-SR-3064_SCREEN_sum

Feature	Result
Variable type	numeric
Number of missing obs.	0 (0 %)
Number of unique values	47
Median	-0.35
1st and 3rd quartiles	-1.29; 0.28
Min. and max.	-1.29; 2.17

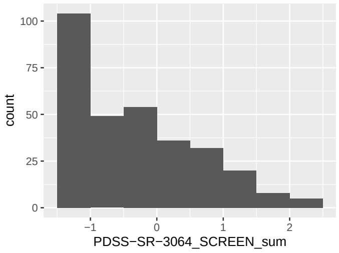

MADRS-1951_SCREEN_sum

Feature	Result
Variable type	numeric
Number of missing obs.	0 (0 %)
Number of unique values	45
Median	0.54
1st and 3rd quartiles	0.05; 1.04
Min. and max.	-2.68; 2.53

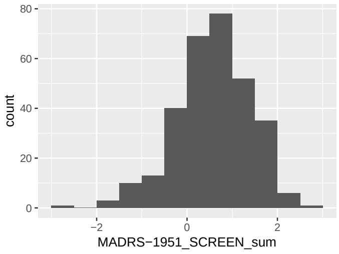

LSAS-2241_SCREEN_sum

Feature	Result
Variable type	numeric
Number of missing obs.	0 (0 %)
Number of unique values	139
Median	-0.23
1st and 3rd quartiles	-0.89; 0.36
Min. and max.	-1.79; 2.99

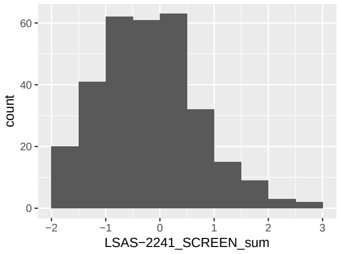

outcome

Feature	Result
Variable type	numeric
Number of missing obs.	0 (0 %)
Number of unique values	125
Median	-0.06
1st and 3rd quartiles	-0.77; 1.08
Min. and max.	-1.59; 3.36

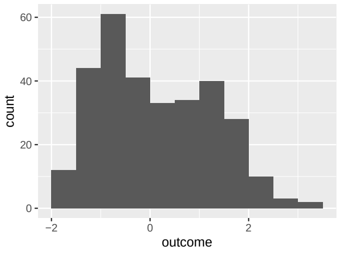

mainsymptom_PRE_sum

Feature	Result
Variable type	numeric
Number of missing obs.	0 (0 %)
Number of unique values	47
Median	0.52
1st and 3rd quartiles	0.03; 1.14
Min. and max.	-2.32; 2.5

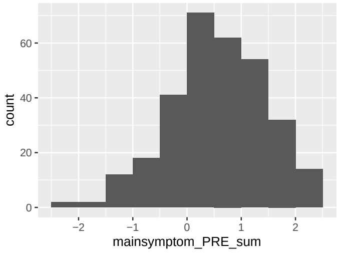

mainsymptom_WEEK01_sum

Feature	Result
Variable type	numeric
Number of missing obs.	0 (0 %)
Number of unique values	101
Median	0.55
1st and 3rd quartiles	-0.18; 1.16
Min. and max.	-2.01; 2.87

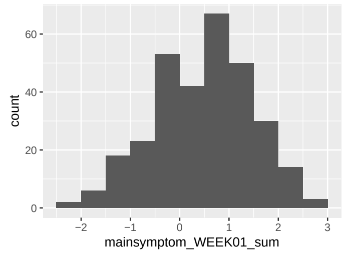

---

mainsymptom_WEEK02_sum

Feature	Result
Variable type	numeric
Number of missing obs.	0 (0 %)
Number of unique values	96
Median	0.5
1st and 3rd quartiles	-0.34; 1.17
Min. and max.	-1.9; 3.01

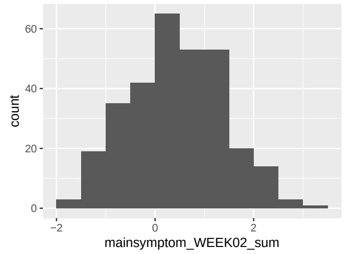

---

mainsymptom_WEEK03_sum

Feature	Result
Variable type	numeric
Number of missing obs.	0 (0 %)
Number of unique values	107
Median	0.47
1st and 3rd quartiles	-0.17; 1.23
Min. and max.	-1.79; 4.04

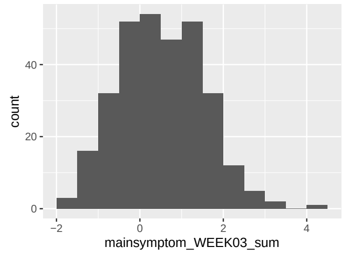

Report generation information:

- Created by: Could not determine from system (username: `nilisa`).
- Report creation time: Mon Jan 09 2023 13:12:14
- Report was run from directory: `/home/nilisa/projects/phd_study1/r`
- dataMaid v1.4.1 [Pkg: 2021-10-08 from CRAN (R 4.2.2)]
- R version 4.2.2 Patched (2022-11-10 r83330).
- Platform: x86_64-pc-linux-gnu (64-bit)(Ubuntu 20.04.5 LTS).
- Function call: `dataMaid::makeDataReport(data = gd, mode = c("summarize", "visualize", "check"), smartNum = FALSE, file = "~/projects/data/study1multiverse/results/graphs_n_figures/codebooks/codebook replace = TRUE, openResult = FALSE, checks = list(character = "showAllFactorLevels", factor = "showAllFactorLevels", labelled = "showAllFactorLevels",   haven_labelled = "showAllFactorLevels", numeric = NULL,   integer = NULL, logical = NULL, Date = NULL), listChecks = FALSE, maxProbVals = Inf, codebook = TRUE, reportTitle = "Handpicked_Depression_week04-`

Handpicked_Depression_week04-imputed_benchmark_train

Autogenerated data summary from dataMaid

2023-01-09 13:05:46

Data report overview

The dataset examined has the following dimensions:

Feature	Result
Number of observations	2768
Number of variables	10

Codebook summary table

Label	Variable	Class	# unique values	Missing	Description
	**sex**	factor	2	0.00 %	Sex of patient, 0 = Female, 1=Male
	**age**	numeric	69	0.00 %	
	**PDSS-SR-3064_SCREEN_sum**	numeric	204	0.00 %	Anxiety questionnaire, self rated-Timepoint before treatment starts-Sum of the entire measure
	**MADRS-1951_SCREEN_sum**	numeric	99	0.00 %	Depression questionnaire, self rated-Timepoint before treatment starts-Sum of the entire measure
	**LSAS-2241_SCREEN_sum**	numeric	436	0.00 %	Social anxiety questionnaire, self rated-Timepoint before treatment starts-Sum of the entire measure
	**outcome**	numeric	646	0.00 %	
	**mainsymptom_PRE_sum**	numeric	95	0.00 %	PDSS-SR for panic, MADRS for depression, LSAS for social anxiety-Timepoint just before beginning treatment-Sum of the entire measure
	**mainsymptom_WEEK01_sum**	numeric	512	0.00 %	PDSS-SR for panic, MADRS for depression, LSAS for social anxiety-Timepoint after one week in treatment-Sum of the entire measure
	**mainsymptom_WEEK02_sum**	numeric	455	0.00 %	PDSS-SR for panic, MADRS for depression, LSAS for social anxiety-Timepoint after two weeks in treatment-Sum of the entire measure

Label	Variable	Class	# unique values	Missing	Description
	**mainsymptom_WEEK03_sum**	numeric	528	0.00 %	PDSS-SR for panic, MADRS for depression, LSAS for social anxiety-Timepoint after three weeks in treatment-Sum of the entire measure

Variable list

sex

Feature	Result
Variable type	factor
Number of missing obs.	0 (0 %)
Number of unique values	2
Mode	“0”
Reference category	0

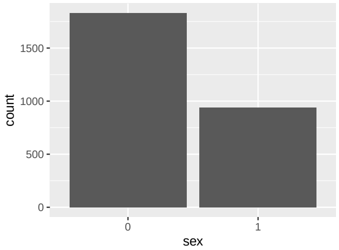

- Observed factor levels: "0", "1".

---

age

Feature	Result
Variable type	numeric
Number of missing obs.	0 (0 %)
Number of unique values	69
Median	0.02
1st and 3rd quartiles	-0.64; 0.85
Min. and max.	-1.69; 4.16

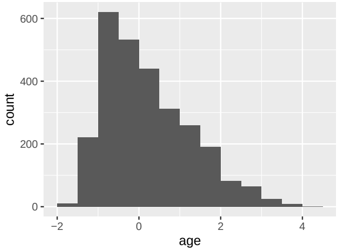

PDSS-SR-3064_SCREEN_sum

Feature	Result
Variable type	numeric
Number of missing obs.	0 (0 %)
Number of unique values	204
Median	-0.51
1st and 3rd quartiles	-1.29; 0.12
Min. and max.	-1.29; 2.96

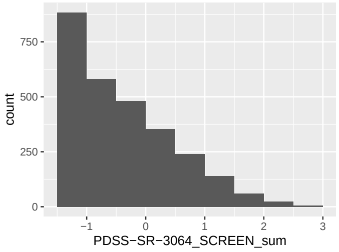

MADRS-1951_SCREEN_sum

Feature	Result
Variable type	numeric
Number of missing obs.	0 (0 %)
Number of unique values	99
Median	0.54
1st and 3rd quartiles	0.05; 1.04
Min. and max.	-2.18; 3.15

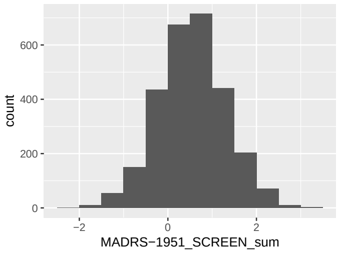

LSAS-2241_SCREEN_sum

Feature	Result
Variable type	numeric
Number of missing obs.	0 (0 %)
Number of unique values	436
Median	-0.3
1st and 3rd quartiles	-0.85; 0.29
Min. and max.	-1.79; 3.09

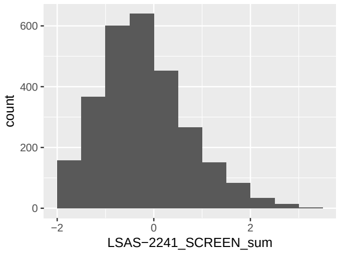

outcome

Feature	Result
Variable type	numeric
Number of missing obs.	0 (0 %)
Number of unique values	646
Median	-0.06
1st and 3rd quartiles	-0.65; 0.65
Min. and max.	-1.59; 4.66

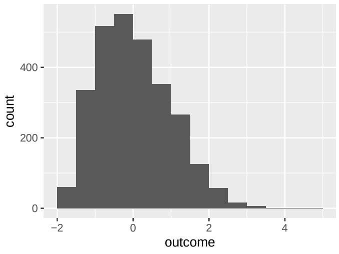

mainsymptom_PRE_sum

Feature	Result
Variable type	numeric
Number of missing obs.	0 (0 %)
Number of unique values	95
Median	0.52
1st and 3rd quartiles	0.03; 1.01
Min. and max.	-2.32; 3.48

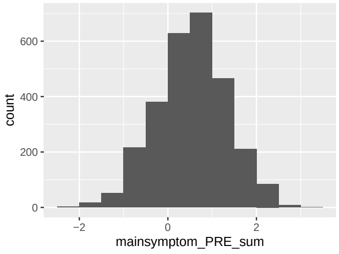

mainsymptom_WEEK01_sum

Feature	Result
Variable type	numeric
Number of missing obs.	0 (0 %)
Number of unique values	512
Median	0.47
1st and 3rd quartiles	-0.05; 1.04
Min. and max.	-2.01; 3.6

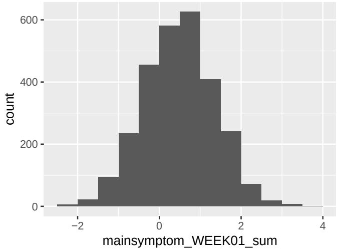

---

mainsymptom_WEEK02_sum

Feature	Result
Variable type	numeric
Number of missing obs.	0 (0 %)
Number of unique values	455
Median	0.5
1st and 3rd quartiles	-0.14; 0.98
Min. and max.	-1.9; 4.09

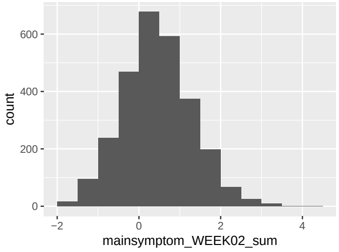

---

mainsymptom_WEEK03_sum

Feature	Result
Variable type	numeric
Number of missing obs.	0 (0 %)
Number of unique values	528
Median	0.36
1st and 3rd quartiles	-0.24; 1.06
Min. and max.	-1.79; 4.64

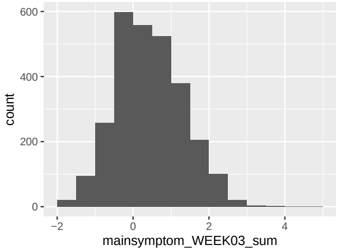

Report generation information:

- Created by: Could not determine from system (username: `nilisa`).
- Report creation time: Mon Jan 09 2023 13:05:46
- Report was run from directory: `/home/nilisa/projects/phd_study1/r`
- dataMaid v1.4.1 [Pkg: 2021-10-08 from CRAN (R 4.2.2)]
- R version 4.2.2 Patched (2022-11-10 r83330).
- Platform: x86_64-pc-linux-gnu (64-bit)(Ubuntu 20.04.5 LTS).
- Function call: `dataMaid::makeDataReport(data = gd, mode = c("summarize", "visualize", "check"), smartNum = FALSE, file = "~/projects/data/study1multiverse/results/graphs_n_figures/codebooks/codebook replace = TRUE, openResult = FALSE, checks = list(character = "showAllFactorLevels", factor = "showAllFactorLevels", labelled = "showAllFactorLevels",   haven_labelled = "showAllFactorLevels", numeric = NULL,   integer = NULL, logical = NULL, Date = NULL), listChecks = FALSE, maxProbVals = Inf, codebook = TRUE, reportTitle = "Handpicked_Depression_week04-`

Handpicked_Depression_week04-imputed_test

Autogenerated data summary from dataMaid

2023-01-09 13:12:22

Data report overview

The dataset examined has the following dimensions:

Feature	Result
Number of observations	308
Number of variables	63

Codebook summary table

Label	Variable	Class	# unique values	Missing	Description
	**sex**	factor	2	0.00 %	Sex of patient, 0 = Female, 1=Male
	**age**	numeric	54	0.00 %	
	**messages_len_7**	numeric	94	0.00 %	-Meta information of messages-Length of messages-up until day-7
	**messages_len_tp_7**	numeric	201	0.00 %	-Meta information of messages-Length of messages-therapist messages-up until day-7
	**messages_7**	numeric	6	0.00 %	-Meta information of messages-up until day-7
	**messages_tp_7**	numeric	7	0.00 %	-Meta information of messages-therapist messages-up until day-7
	**homeworks_7**	numeric	5	0.00 %	-Number of homework messages sent in-up until day-7
	**messages_len_14**	numeric	113	0.00 %	-Meta information of messages-Length of messages-up until day-14
	**messages_len_tp_14**	numeric	226	0.00 %	-Meta information of messages-Length of messages-therapist messages-up until day-14
	**messages_14**	numeric	7	0.00 %	-Meta information of messages-up until day-14
	**messages_tp_14**	numeric	6	0.00 %	-Meta information of messages-therapist messages-up until day-14

Label	Variable	Class	# unique values	Missing	Description
	**homeworks_14**	numeric	7	0.00 %	-Number of homework messages sent in-up until day-14
	**messages_len_21**	numeric	129	0.00 %	-Meta information of messages-Length of messages-up until day-21
	**messages_len_tp_21**	numeric	218	0.00 %	-Meta information of messages-Length of messages-therapist messages-up until day-21
	**messages_21**	numeric	7	0.00 %	-Meta information of messages-up until day-21
	**messages_tp_21**	numeric	6	0.00 %	-Meta information of messages-therapist messages-up until day-21
	**homeworks_21**	numeric	5	0.00 %	-Number of homework messages sent in-up until day-21
	**messages_len_28**	numeric	113	0.00 %	-Meta information of messages-Length of messages-up until day-28
	**messages_len_tp_28**	numeric	210	0.00 %	-Meta information of messages-Length of messages-therapist messages-up until day-28
	**messages_28**	numeric	7	0.00 %	-Meta information of messages-up until day-28
	**messages_tp_28**	numeric	7	0.00 %	-Meta information of messages-therapist messages-up until day-28
	**homeworks_28**	numeric	5	0.00 %	-Number of homework messages sent in-up until day-28
	**PDSS-SR-3064_SCREEN_sum**	numeric	48	0.00 %	Anxiety questionnaire, self rated-Timepoint before treatment starts-Sum of the entire measure
	**MADRS-1951_SCREEN_sum**	numeric	45	0.00 %	Depression questionnaire, self rated-Timepoint before treatment starts-Sum of the entire measure
	**LSAS-2241_SCREEN_sum**	numeric	139	0.00 %	Social anxiety questionnaire, self rated-Timepoint before treatment starts-Sum of the entire measure
	**MADRS-1951_SCREEN_DateCompleted_day**	numeric	12	0.00 %	Depression questionnaire, self rated-Timepoint before treatment starts-Cyclic transformation of what day 0-6 during week it was filled in

Label	Variable	Class	# unique values	Missing	Description
	**MADRS-1951_SCREEN_DateCompleted_time**	numeric	265	0.00 %	Depression questionnaire, self rated-Timepoint before treatment starts-Cyclic transformation of what time during day 0-1440 it was filled in
	**PDSS-SR-3064_SCREEN_DateCompleted_day**	numeric	21	0.00 %	Anxiety questionnaire, self rated-Timepoint before treatment starts-Cyclic transformation of what day 0-6 during week it was filled in
	**PDSS-SR-3064_SCREEN_DateCompleted_time**	numeric	276	0.00 %	Anxiety questionnaire, self rated-Timepoint before treatment starts-Cyclic transformation of what time during day 0-1440 it was filled in
	**LSAS-2241_SCREEN_DateCompleted_day**	numeric	26	0.00 %	Social anxiety questionnaire, self rated-Timepoint before treatment starts-Cyclic transformation of what day 0-6 during week it was filled in
	**LSAS-2241_SCREEN_DateCompleted_time**	numeric	273	0.00 %	Social anxiety questionnaire, self rated-Timepoint before treatment starts-Cyclic transformation of what time during day 0-1440 it was filled in
	**outcome**	numeric	126	0.00 %	
	**ncomorbid**	numeric	26	0.00 %	
	**HW-01**	numeric	98	0.00 %	
	**HW-02**	numeric	136	0.00 %	
	**HW-03**	numeric	134	0.00 %	
	**currentwork_proff**	factor	16	0.00 %	Currently in work for trained proffession
	**Marital_1833_gift**	factor	2	0.00 %	Marital status: Married or not
	**Marital_1833_separerad**	factor	2	0.00 %	Marital status: divocered/equivalent
	**Marital_1833_singel**	factor	2	0.00 %	Marital status: single
	**Edu_1843_2**	factor	2	0.00 %	7-9 years education
	**Edu_1843_3**	factor	2	0.00 %	Uncompleted upper secondary school
	**Edu_1843_4**	factor	2	0.00 %	Higher vocational education
	**Edu_1843_5**	factor	2	0.00 %	Completed upper secondary school
	**Edu_1843_6**	factor	2	0.00 %	Uncompleted university degree
	**Edu_1843_7**	factor	2	0.00 %	University degree
	**cscale**	numeric	80	0.00 %	
	**mainsymptom_PRE_sum**	numeric	47	0.00 %	PDSS-SR for panic, MADRS for depression, LSAS for social anxiety-Timepoint just before beginning treatment-Sum of the entire measure

Label	Variable	Class	# unique values	Missing	Description
	**mainsymptom_PRE_duration**	numeric	251	0.00 %	PDSS-SR for panic, MADRS for depression, LSAS for social anxiety-Timepoint just before beginning treatment-Time to fill in measure/questionnaire
	**mainsymptom_PRE_DateCompleted_day**	numeric	18	0.00 %	PDSS-SR for panic, MADRS for depression, LSAS for social anxiety-Timepoint just before beginning treatment-Cyclic transformation of what day 0-6 during week it was filled in
	**mainsymptom_PRE_DateCompleted_time**	numeric	254	0.00 %	PDSS-SR for panic, MADRS for depression, LSAS for social anxiety-Timepoint just before beginning treatment-Cyclic transformation of what time during day 0-1440 it was filled in
	**mainsymptom_WEEK01_sum**	numeric	100	0.00 %	PDSS-SR for panic, MADRS for depression, LSAS for social anxiety-Timepoint after one week in treatment-Sum of the entire measure
	**mainsymptom_WEEK01_duration**	numeric	221	0.00 %	PDSS-SR for panic, MADRS for depression, LSAS for social anxiety-Timepoint after one week in treatment-Time to fill in measure/questionnaire
	**mainsymptom_WEEK01_DateCompleted_day**	numeric	68	0.00 %	PDSS-SR for panic, MADRS for depression, LSAS for social anxiety-Timepoint after one week in treatment-Cyclic transformation of what day 0-6 during week it was filled in
	**mainsymptom_WEEK01_DateCompleted_time**	numeric	274	0.00 %	PDSS-SR for panic, MADRS for depression, LSAS for social anxiety-Timepoint after one week in treatment-Cyclic transformation of what time during day 0-1440 it was filled in
	**mainsymptom_WEEK02_sum**	numeric	95	0.00 %	PDSS-SR for panic, MADRS for depression, LSAS for social anxiety-Timepoint after two weeks in treatment-Sum of the entire measure
	**mainsymptom_WEEK02_duration**	numeric	210	0.00 %	PDSS-SR for panic, MADRS for depression, LSAS for social anxiety-Timepoint after two weeks in treatment-Time to fill in measure/questionnaire

Label	Variable	Class	# unique values	Missing	Description
	**mainsymptom_WEEK02_DateCompleted_day**	numeric	63	0.00 %	PDSS-SR for panic, MADRS for depression, LSAS for social anxiety-Timepoint after two weeks in treatment-Cyclic transformation of what day 0-6 during week it was filled in
	**mainsymptom_WEEK02_DateCompleted_time**	numeric	277	0.00 %	PDSS-SR for panic, MADRS for depression, LSAS for social anxiety-Timepoint after two weeks in treatment-Cyclic transformation of what time during day 0-1440 it was filled in
	**mainsymptom_WEEK03_sum**	numeric	107	0.00 %	PDSS-SR for panic, MADRS for depression, LSAS for social anxiety-Timepoint after three weeks in treatment-Sum of the entire measure
	**mainsymptom_WEEK03_duration**	numeric	216	0.00 %	PDSS-SR for panic, MADRS for depression, LSAS for social anxiety-Timepoint after three weeks in treatment-Time to fill in measure/questionnaire
	**mainsymptom_WEEK03_DateCompleted_day**	numeric	71	0.00 %	PDSS-SR for panic, MADRS for depression, LSAS for social anxiety-Timepoint after three weeks in treatment-Cyclic transformation of what day 0-6 during week it was filled in
	**mainsymptom_WEEK03_DateCompleted_time**	numeric	278	0.00 %	PDSS-SR for panic, MADRS for depression, LSAS for social anxiety-Timepoint after three weeks in treatment-Cyclic transformation of what time during day 0-1440 it was filled in

Variable list

sex

Feature	Result
Variable type	factor
Number of missing obs.	0 (0 %)
Number of unique values	2
Mode	“0”
Reference category	0

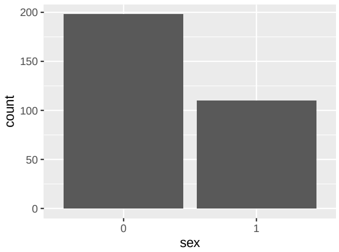

- Observed factor levels: "0", "1".

---

age

Feature	Result
Variable type	numeric
Number of missing obs.	0 (0 %)
Number of unique values	54
Median	0.1
1st and 3rd quartiles	-0.55; 0.85
Min. and max.	-1.6; 4.08

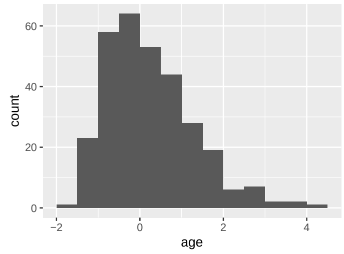

messages_len_7

Feature	Result
Variable type	numeric
Number of missing obs.	0 (0 %)
Number of unique values	94
Median	-0.32
1st and 3rd quartiles	-0.32; -0.08
Min. and max.	-0.32; 5.15

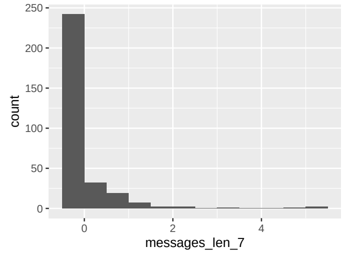

messages_len_tp_7

Feature	Result
Variable type	numeric
Number of missing obs.	0 (0 %)
Number of unique values	201
Median	-0.2
1st and 3rd quartiles	-0.95; 0.56
Min. and max.	-1.46; 4.25

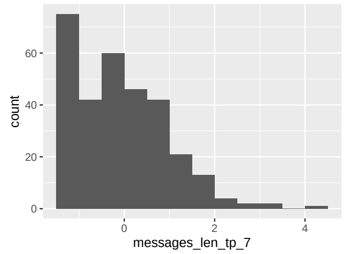

messages_7

Feature	Result
Variable type	numeric
Number of missing obs.	0 (0 %)
Number of unique values	6
Median	-0.55
1st and 3rd quartiles	-0.55; 0.58
Min. and max.	-0.55; 5.13

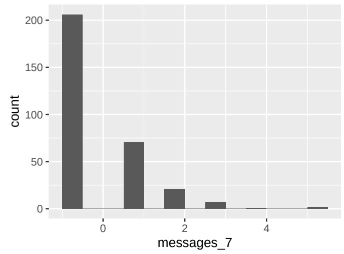

messages_tp_7

Feature	Result
Variable type	numeric
Number of missing obs.	0 (0 %)
Number of unique values	7
Median	0.15
1st and 3rd quartiles	-0.91; 0.15
Min. and max.	-1.97; 4.4

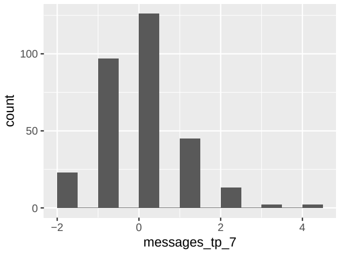

homeworks_7

Feature	Result
Variable type	numeric
Number of missing obs.	0 (0 %)
Number of unique values	5
Median	0.2
1st and 3rd quartiles	-1.03; 0.2
Min. and max.	-1.03; 3.91

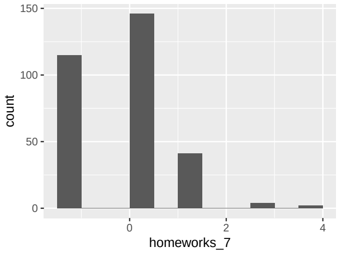

messages_len_14

Feature	Result
Variable type	numeric
Number of missing obs.	0 (0 %)
Number of unique values	113
Median	-0.41
1st and 3rd quartiles	-0.41; 0.05
Min. and max.	-0.41; 12.94

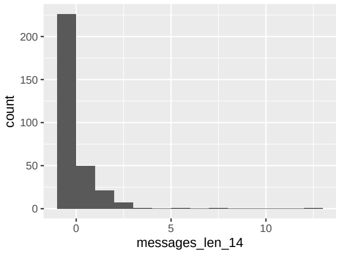

messages_len_tp_14

Feature	Result
Variable type	numeric
Number of missing obs.	0 (0 %)
Number of unique values	226
Median	-0.14
1st and 3rd quartiles	-0.84; 0.61
Min. and max.	-1.11; 14.98

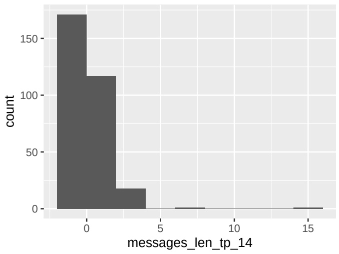

messages_14

Feature	Result
Variable type	numeric
Number of missing obs.	0 (0 %)
Number of unique values	7
Median	-0.7
1st and 3rd quartiles	-0.7; 0.36
Min. and max.	-0.7; 12.02

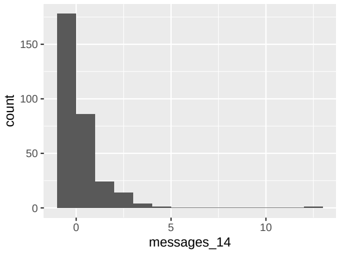

messages_tp_14

Feature	Result
Variable type	numeric
Number of missing obs.	0 (0 %)
Number of unique values	6
Median	-0.35
1st and 3rd quartiles	-0.35; 0.87
Min. and max.	-1.57; 8.19

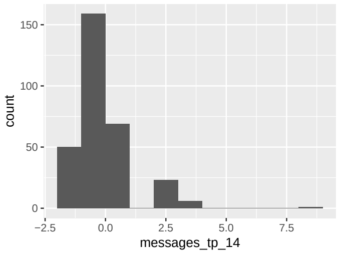

homeworks_14

Feature	Result
Variable type	numeric
Number of missing obs.	0 (0 %)
Number of unique values	7
Median	0.33
1st and 3rd quartiles	-0.98; 0.33
Min. and max.	-0.98; 14.78

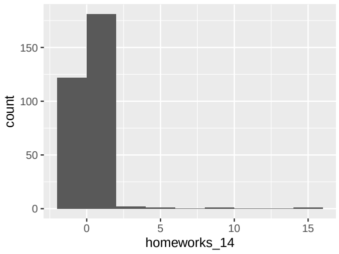

messages_len_21

Feature	Result
Variable type	numeric
Number of missing obs.	0 (0 %)
Number of unique values	129
Median	-0.46
1st and 3rd quartiles	-0.46; 0.15
Min. and max.	-0.46; 11.54

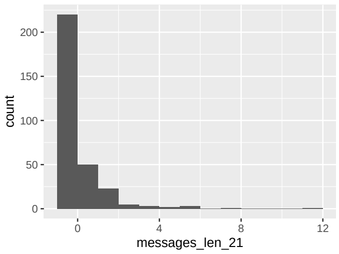

messages_len_tp_21

Feature	Result
Variable type	numeric
Number of missing obs.	0 (0 %)
Number of unique values	218
Median	-0.29
1st and 3rd quartiles	-0.86; 0.63
Min. and max.	-1.01; 8.85

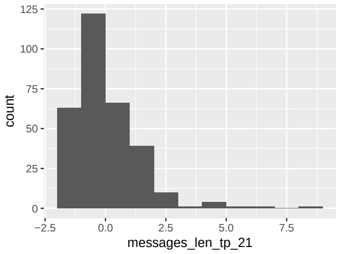

messages_21

Feature	Result
Variable type	numeric
Number of missing obs.	0 (0 %)
Number of unique values	7
Median	-0.74
1st and 3rd quartiles	-0.74; 0.34
Min. and max.	-0.74; 5.75

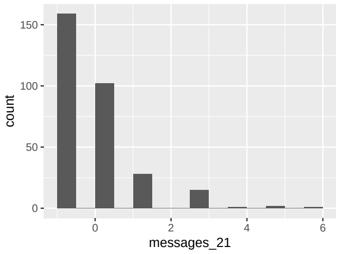

messages_tp_21

Feature	Result
Variable type	numeric
Number of missing obs.	0 (0 %)
Number of unique values	6
Median	-0.32
1st and 3rd quartiles	-0.32; 0.84
Min. and max.	-1.49; 5.5

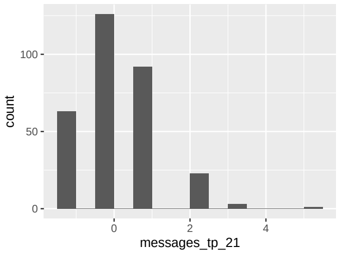

homeworks_21

Feature	Result
Variable type	numeric
Number of missing obs.	0 (0 %)
Number of unique values	5
Median	0.36
1st and 3rd quartiles	-1.01; 0.36
Min. and max.	-1.01; 4.49

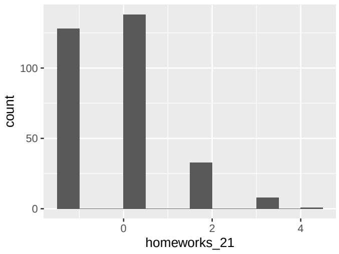

messages_len_28

Feature	Result
Variable type	numeric
Number of missing obs.	0 (0 %)
Number of unique values	113
Median	-0.41
1st and 3rd quartiles	-0.41; 0.04
Min. and max.	-0.41; 7.28

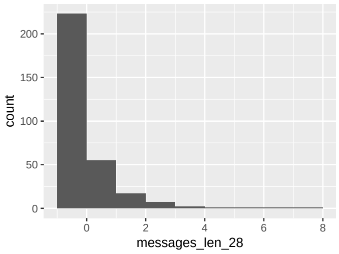

messages_len_tp_28

Feature	Result
Variable type	numeric
Number of missing obs.	0 (0 %)
Number of unique values	210
Median	-0.16
1st and 3rd quartiles	-0.76; 0.53
Min. and max.	-0.94; 6.05

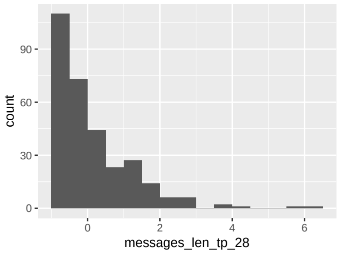

messages_28

Feature	Result
Variable type	numeric
Number of missing obs.	0 (0 %)
Number of unique values	7
Median	-0.72
1st and 3rd quartiles	-0.72; 0.41
Min. and max.	-0.72; 10.59

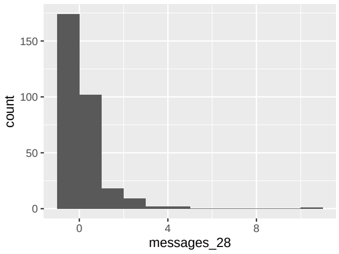

messages_tp_28

Feature	Result
Variable type	numeric
Number of missing obs.	0 (0 %)
Number of unique values	7
Median	-0.24
1st and 3rd quartiles	-0.24; 0.93
Min. and max.	-1.42; 7.97

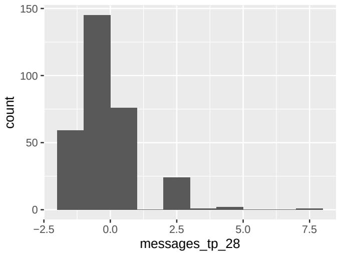

homeworks_28

Feature	Result
Variable type	numeric
Number of missing obs.	0 (0 %)
Number of unique values	5
Median	0.46
1st and 3rd quartiles	-0.91; 0.46
Min. and max.	-0.91; 4.57

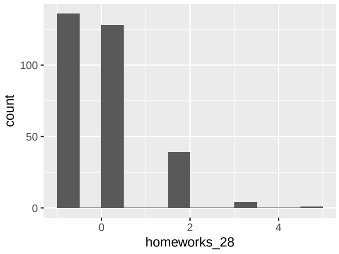

PDSS-SR-3064_SCREEN_sum

Feature	Result
Variable type	numeric
Number of missing obs.	0 (0 %)
Number of unique values	48
Median	-0.37
1st and 3rd quartiles	-1.29; 0.28
Min. and max.	-1.29; 2.17

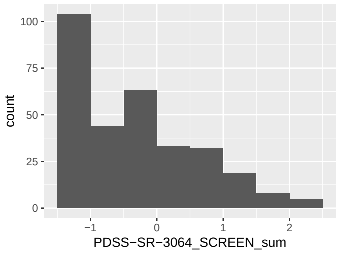

MADRS-1951_SCREEN_sum

Feature	Result
Variable type	numeric
Number of missing obs.	0 (0 %)
Number of unique values	45
Median	0.54
1st and 3rd quartiles	0.05; 1.05
Min. and max.	-2.68; 2.53

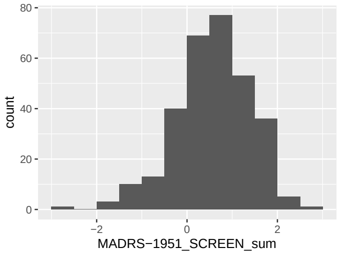

LSAS-2241_SCREEN_sum

Feature	Result
Variable type	numeric
Number of missing obs.	0 (0 %)
Number of unique values	139
Median	-0.27
1st and 3rd quartiles	-0.85; 0.36
Min. and max.	-1.79; 2.99

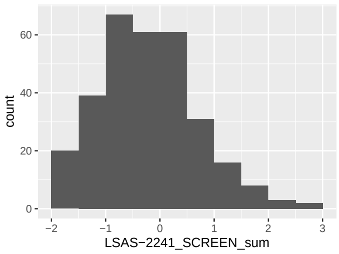

MADRS-1951_SCREEN_DateCompleted_day

Feature	Result
Variable type	numeric
Number of missing obs.	0 (0 %)
Number of unique values	12
Median	0.37
1st and 3rd quartiles	-1; 1
Min. and max.	-1.37; 1.37

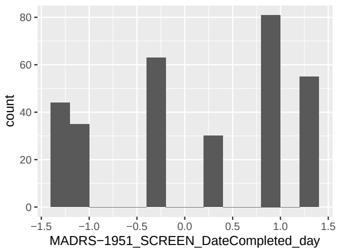

MADRS-1951_SCREEN_DateCompleted_time

Feature	Result
Variable type	numeric
Number of missing obs.	0 (0 %)
Number of unique values	265
Median	-0.77
1st and 3rd quartiles	-1.2; 0.27
Min. and max.	-1.41; 1.41

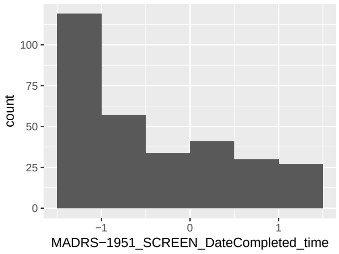

PDSS-SR-3064_SCREEN_DateCompleted_day

Feature	Result
Variable type	numeric
Number of missing obs.	0 (0 %)
Number of unique values	21
Median	0.37
1st and 3rd quartiles	-0.37; 1
Min. and max.	-1.37; 1.37

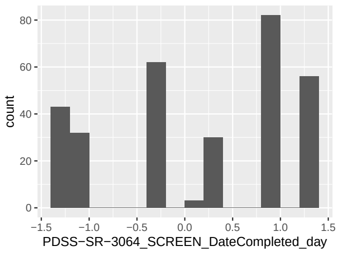

PDSS-SR-3064_SCREEN_DateCompleted_time

Feature	Result
Variable type	numeric
Number of missing obs.	0 (0 %)
Number of unique values	276
Median	-0.75
1st and 3rd quartiles	-1.24; 0.26
Min. and max.	-1.41; 1.41

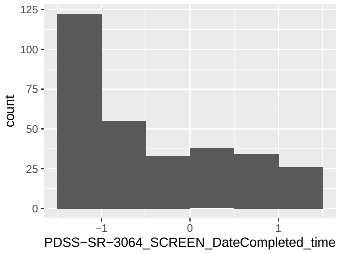

LSAS-2241_SCREEN_DateCompleted_day

Feature	Result
Variable type	numeric
Number of missing obs.	0 (0 %)
Number of unique values	26
Median	0.37
1st and 3rd quartiles	-0.37; 1
Min. and max.	-1.37; 1.37

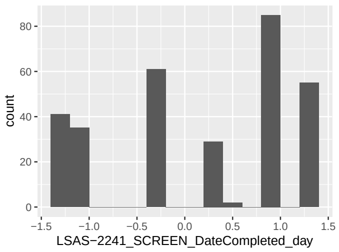

LSAS-2241_SCREEN_DateCompleted_time

Feature	Result
Variable type	numeric
Number of missing obs.	0 (0 %)
Number of unique values	273
Median	-0.77
1st and 3rd quartiles	-1.23; 0.29
Min. and max.	-1.41; 1.41

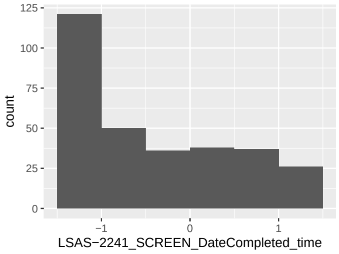

outcome

Feature	Result
Variable type	numeric
Number of missing obs.	0 (0 %)
Number of unique values	126
Median	-0.04
1st and 3rd quartiles	-0.74; 1.12
Min. and max.	-1.59; 3.36

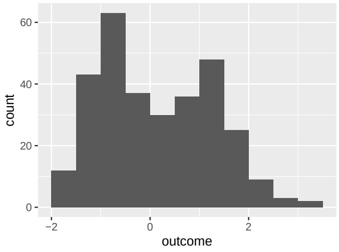

ncomorbid

Feature	Result
Variable type	numeric
Number of missing obs.	0 (0 %)
Number of unique values	26
Median	0
1st and 3rd quartiles	0; 1
Min. and max.	0; 3

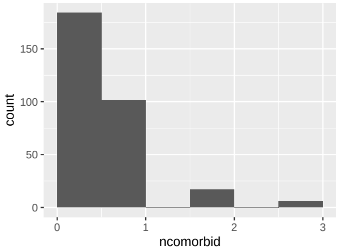

HW-01

Feature	Result
Variable type	numeric
Number of missing obs.	0 (0 %)
Number of unique values	98
Median	-0.08
1st and 3rd quartiles	-0.98; 0.26
Min. and max.	-0.98; 1.71

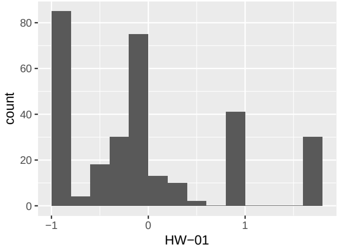

HW-02

Feature	Result
Variable type	numeric
Number of missing obs.	0 (0 %)
Number of unique values	136
Median	-0.29
1st and 3rd quartiles	-0.63; 0.02
Min. and max.	-0.92; 1.89

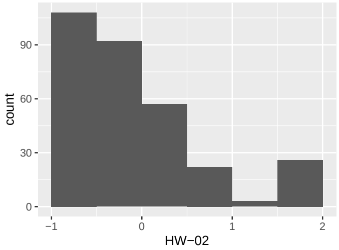

HW-03

Feature	Result
Variable type	numeric
Number of missing obs.	0 (0 %)
Number of unique values	134
Median	-0.24
1st and 3rd quartiles	-0.38; 0.07
Min. and max.	-0.92; 2.04

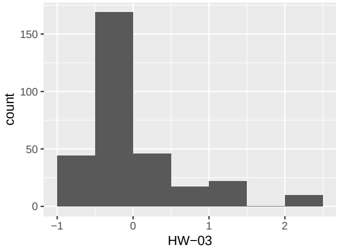

currentwork_proff

Feature	Result
Variable type	factor
Number of missing obs.	0 (0 %)
Number of unique values	16
Mode	“1”
Reference category	0

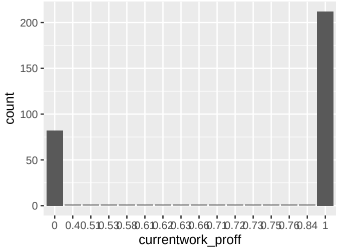

- Observed factor levels: "0", "0.4", "0.51", "0.53", "0.58", "0.61", "0.62", "0.63", "0.66", "0.71", "0.72", "0.73", "0.75", "0.76", "0.84", "1".

Marital_1833_gift

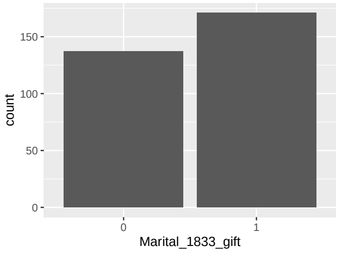

Feature	Result
Variable type	factor
Number of missing obs.	0 (0 %)
Number of unique values	2
Mode	“1”
Reference category	0

- Observed factor levels: "0", "1".

Marital_1833_separerad

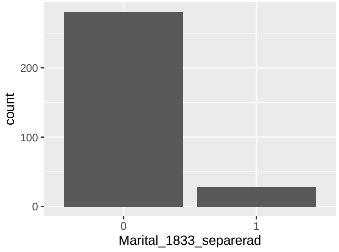

Feature	Result
Variable type	factor
Number of missing obs.	0 (0 %)
Number of unique values	2
Mode	“0”
Reference category	0

- Observed factor levels: "0", "1".

Marital_1833_singel

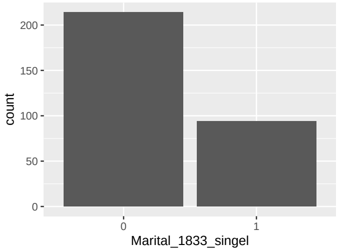

Feature	Result
Variable type	factor
Number of missing obs.	0 (0 %)
Number of unique values	2
Mode	“0”
Reference category	0

- Observed factor levels: "0", "1".

Edu_1843_2

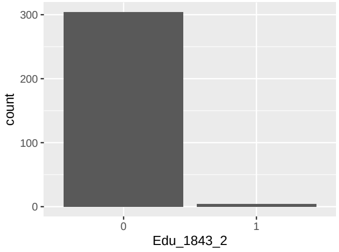

Feature	Result
Variable type	factor
Number of missing obs.	0 (0 %)
Number of unique values	2
Mode	“0”
Reference category	0

- Observed factor levels: "0", "1".

Edu_1843_3

Feature	Result
Variable type	factor
Number of missing obs.	0 (0 %)
Number of unique values	2
Mode	“0”
Reference category	0

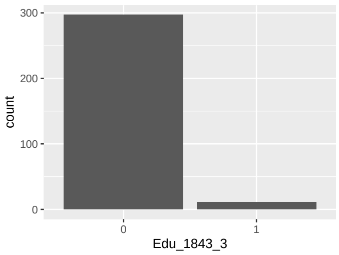

- Observed factor levels: "0", "1".

Edu_1843_4

Feature	Result
Variable type	factor
Number of missing obs.	0 (0 %)
Number of unique values	2
Mode	“0”
Reference category	0

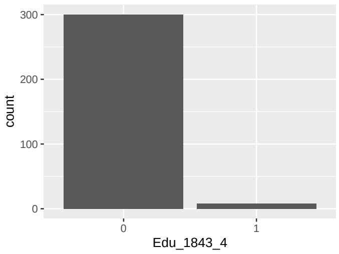

- Observed factor levels: "0", "1".

Edu_1843_5

Feature	Result
Variable type	factor
Number of missing obs.	0 (0 %)
Number of unique values	2
Mode	“0”
Reference category	0

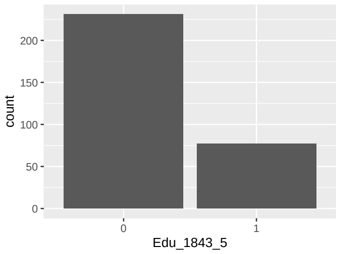

- Observed factor levels: "0", "1".

Edu_1843_6

Feature	Result
Variable type	factor
Number of missing obs.	0 (0 %)
Number of unique values	2
Mode	“0”
Reference category	0

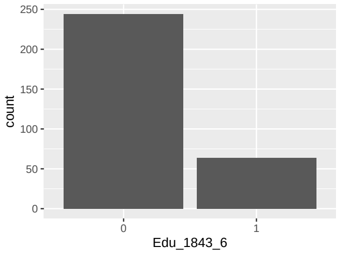

- Observed factor levels: "0", "1".

Edu_1843_7

Feature	Result
Variable type	factor
Number of missing obs.	0 (0 %)
Number of unique values	2
Mode	“0”
Reference category	0

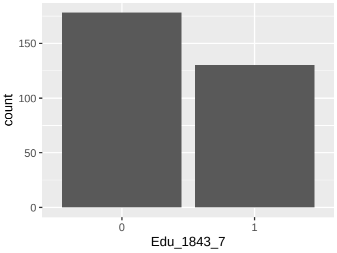

- Observed factor levels: "0", "1".

---

cscale

Feature	Result
Variable type	numeric
Number of missing obs.	0 (0 %)
Number of unique values	80
Median	-0.23
1st and 3rd quartiles	-1.06; 0.49
Min. and max.	-3.55; 1.79

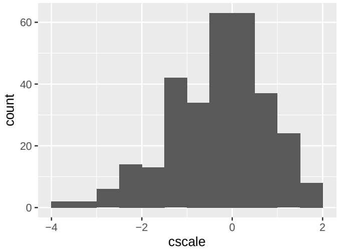

---

mainsymptom_PRE_sum

Feature	Result
Variable type	numeric
Number of missing obs.	0 (0 %)
Number of unique values	47
Median	0.52
1st and 3rd quartiles	0.03; 1.14
Min. and max.	-2.32; 2.5

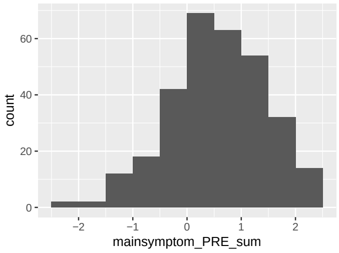

---

mainsymptom_PRE_duration

Feature	Result
Variable type	numeric
Number of missing obs.	0 (0 %)
Number of unique values	251
Median	-0.09
1st and 3rd quartiles	-0.17; 0.04
Min. and max.	-0.25; 2.84

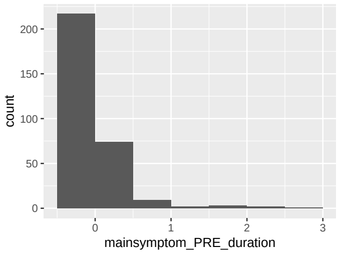

---

mainsymptom_PRE_DateCompleted_day

Feature	Result
Variable type	numeric
Number of missing obs.	0 (0 %)
Number of unique values	18
Median	1
1st and 3rd quartiles	-1; 1
Min. and max.	-1.37; 1.37

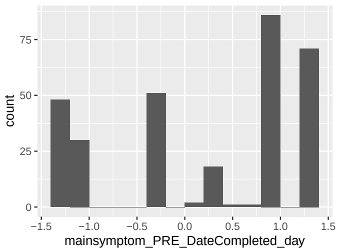

mainsymptom_PRE_DateCompleted_time

Feature	Result
Variable type	numeric
Number of missing obs.	0 (0 %)
Number of unique values	254
Median	-0.62
1st and 3rd quartiles	-1.22; 0.13
Min. and max.	-1.41; 1.41

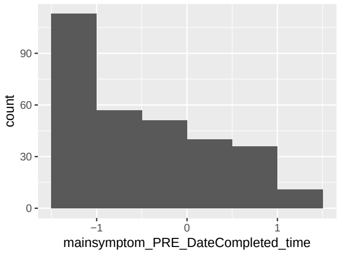

mainsymptom_WEEK01_sum

Feature	Result
Variable type	numeric
Number of missing obs.	0 (0 %)
Number of unique values	100
Median	0.55
1st and 3rd quartiles	-0.18; 1.16
Min. and max.	-2.01; 2.87

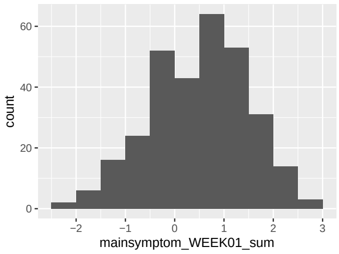

mainsymptom_WEEK01_duration

Feature	Result
Variable type	numeric
Number of missing obs.	0 (0 %)
Number of unique values	221
Median	-0.07
1st and 3rd quartiles	-0.12; 0
Min. and max.	-0.23; 15.74

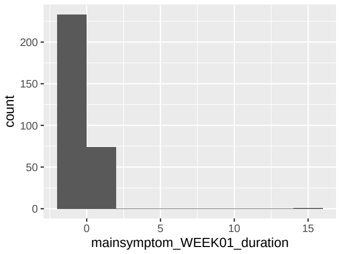

mainsymptom_WEEK01_DateCompleted_day

Feature	Result
Variable type	numeric
Number of missing obs.	0 (0 %)
Number of unique values	68
Median	0.52
1st and 3rd quartiles	-0.37; 1
Min. and max.	-1.37; 1.37

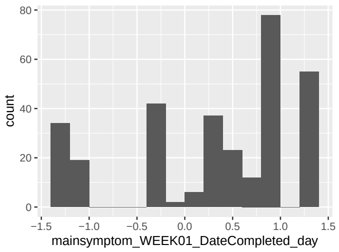

mainsymptom_WEEK01_DateCompleted_time

Feature	Result
Variable type	numeric
Number of missing obs.	0 (0 %)
Number of unique values	274
Median	-0.6
1st and 3rd quartiles	-1.06; -0.05
Min. and max.	-1.41; 1.41

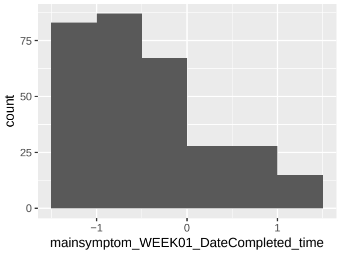

mainsymptom_WEEK02_sum

Feature	Result
Variable type	numeric
Number of missing obs.	0 (0 %)
Number of unique values	95
Median	0.5
1st and 3rd quartiles	-0.34; 1.19
Min. and max.	-1.9; 3.01

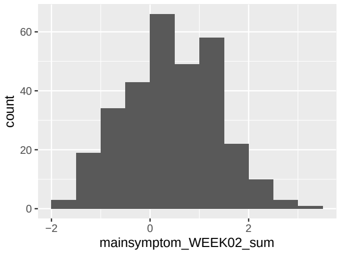

mainsymptom_WEEK02_duration

Feature	Result
Variable type	numeric
Number of missing obs.	0 (0 %)
Number of unique values	210
Median	-0.06
1st and 3rd quartiles	-0.1; 0.02
Min. and max.	-0.17; 12.97

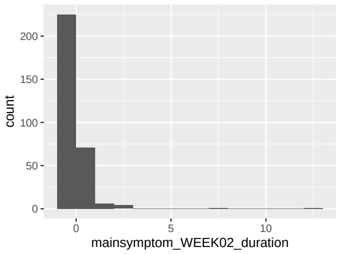

mainsymptom_WEEK02_DateCompleted_day

Feature	Result
Variable type	numeric
Number of missing obs.	0 (0 %)
Number of unique values	63
Median	0.47
1st and 3rd quartiles	-0.37; 1
Min. and max.	-1.37; 1.37

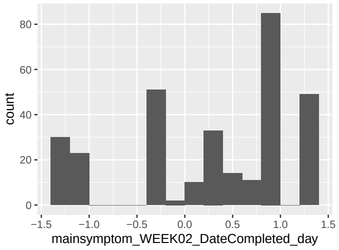

mainsymptom_WEEK02_DateCompleted_time

Feature	Result
Variable type	numeric
Number of missing obs.	0 (0 %)
Number of unique values	277
Median	-0.57
1st and 3rd quartiles	-0.97; 0.08
Min. and max.	-1.41; 1.41

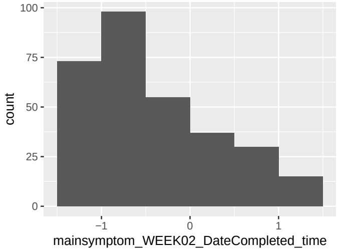

mainsymptom_WEEK03_sum

Feature	Result
Variable type	numeric
Number of missing obs.	0 (0 %)
Number of unique values	107
Median	0.47
1st and 3rd quartiles	-0.21; 1.28
Min. and max.	-1.79; 4.04

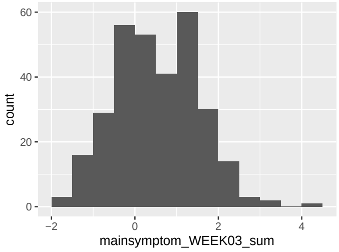

mainsymptom_WEEK03_duration

Feature	Result
Variable type	numeric
Number of missing obs.	0 (0 %)
Number of unique values	216
Median	-0.06
1st and 3rd quartiles	-0.09; 0
Min. and max.	-0.15; 5.21

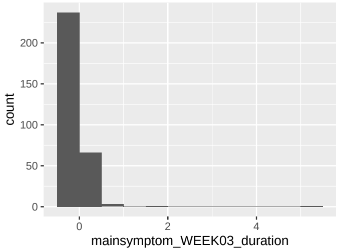

mainsymptom_WEEK03_DateCompleted_day

Feature	Result
Variable type	numeric
Number of missing obs.	0 (0 %)
Number of unique values	71
Median	0.59
1st and 3rd quartiles	-0.37; 1
Min. and max.	-1.37; 1.37

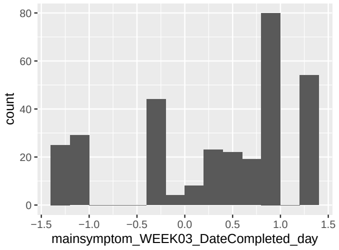

---

mainsymptom_WEEK03_DateCompleted_time

Feature	Result
Variable type	numeric
Number of missing obs.	0 (0 %)
Number of unique values	278
Median	-0.62
1st and 3rd quartiles	-1.05; -0.1
Min. and max.	-1.41; 1.41

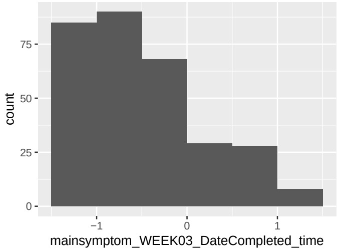

Report generation information:

- Created by: Could not determine from system (username: `nilisa`).
- Report creation time: Mon Jan 09 2023 13:12:22
- Report was run from directory: `/home/nilisa/projects/phd_study1/r`
- dataMaid v1.4.1 [Pkg: 2021-10-08 from CRAN (R 4.2.2)]
- R version 4.2.2 Patched (2022-11-10 r83330).
- Platform: x86_64-pc-linux-gnu (64-bit)(Ubuntu 20.04.5 LTS).
- Function call:

```
dataMaid::makeDataReport(data = gd, mode = c("summarize", "visualize",  "check"),
smartNum = FALSE, file = "~/projects/data/study1multiverse/results/graphs_n_figures/codebooks/codebook
replace = TRUE, openResult = FALSE, checks = list(character = "showAllFactorLevels",
factor = "showAllFactorLevels", labelled = "showAllFactorLevels",      haven_labelled =
"showAllFactorLevels", numeric = NULL,      integer = NULL, logical = NULL, Date = NULL),
listChecks = FALSE,  maxProbVals = Inf, codebook = TRUE, reportTitle = "Handpicked_Depression_week04-
```

Handpicked_Depression_week04-imputed_train

Autogenerated data summary from dataMaid

2023-01-09 13:05:55

Data report overview

The dataset examined has the following dimensions:

Feature	Result
Number of observations	2768
Number of variables	63

Codebook summary table

Label	Variable	Class	# unique values	Missing	Description
	**sex**	factor	2	0.00 %	Sex of patient, 0 = Female, 1=Male
	**age**	numeric	69	0.00 %	
	**messages_len_7**	numeric	452	0.00 %	-Meta information of messages-Length of messages-up until day-7
	**messages_len_tp_7**	numeric	920	0.00 %	-Meta information of messages-Length of messages-therapist messages-up until day-7
	**messages_7**	numeric	9	0.00 %	-Meta information of messages-up until day-7
	**messages_tp_7**	numeric	9	0.00 %	-Meta information of messages-therapist messages-up until day-7
	**homeworks_7**	numeric	5	0.00 %	-Number of homework messages sent in-up until day-7
	**messages_len_14**	numeric	536	0.00 %	-Meta information of messages-Length of messages-up until day-14
	**messages_len_tp_14**	numeric	950	0.00 %	-Meta information of messages-Length of messages-therapist messages-up until day-14
	**messages_14**	numeric	9	0.00 %	-Meta information of messages-up until day-14
	**messages_tp_14**	numeric	8	0.00 %	-Meta information of messages-therapist messages-up until day-14

Label	Variable	Class	# unique values	Missing	Description
	**homeworks_14**	numeric	6	0.00 %	-Number of homework messages sent in-up until day-14
	**messages_len_21**	numeric	516	0.00 %	-Meta information of messages-Length of messages-up until day-21
	**messages_len_tp_21**	numeric	944	0.00 %	-Meta information of messages-Length of messages-therapist messages-up until day-21
	**messages_21**	numeric	9	0.00 %	-Meta information of messages-up until day-21
	**messages_tp_21**	numeric	7	0.00 %	-Meta information of messages-therapist messages-up until day-21
	**homeworks_21**	numeric	7	0.00 %	-Number of homework messages sent in-up until day-21
	**messages_len_28**	numeric	458	0.00 %	-Meta information of messages-Length of messages-up until day-28
	**messages_len_tp_28**	numeric	902	0.00 %	-Meta information of messages-Length of messages-therapist messages-up until day-28
	**messages_28**	numeric	7	0.00 %	-Meta information of messages-up until day-28
	**messages_tp_28**	numeric	7	0.00 %	-Meta information of messages-therapist messages-up until day-28
	**homeworks_28**	numeric	6	0.00 %	-Number of homework messages sent in-up until day-28
	**PDSS-SR-3064_SCREEN_sum**	numeric	198	0.00 %	Anxiety questionnaire, self rated-Timepoint before treatment starts-Sum of the entire measure
	**MADRS-1951_SCREEN_sum**	numeric	98	0.00 %	Depression questionnaire, self rated-Timepoint before treatment starts-Sum of the entire measure
	**LSAS-2241_SCREEN_sum**	numeric	438	0.00 %	Social anxiety questionnaire, self rated-Timepoint before treatment starts-Sum of the entire measure
	**MADRS-1951_SCREEN_DateCompleted_day**	numeric	18	0.00 %	Depression questionnaire, self rated-Timepoint before treatment starts-Cyclic transformation of what day 0-6 during week it was filled in

Label	Variable	Class	# unique values	Missing	Description
	**MADRS-1951_SCREEN_DateCompleted_time**	numeric	931	0.00 %	Depression questionnaire, self rated-Timepoint before treatment starts-Cyclic transformation of what time during day 0-1440 it was filled in
	**PDSS-SR-3064_SCREEN_DateCompleted_day**	numeric	37	0.00 %	Anxiety questionnaire, self rated-Timepoint before treatment starts-Cyclic transformation of what day 0-6 during week it was filled in
	**PDSS-SR-3064_SCREEN_DateCompleted_time**	numeric	1054	0.00 %	Anxiety questionnaire, self rated-Timepoint before treatment starts-Cyclic transformation of what time during day 0-1440 it was filled in
	**LSAS-2241_SCREEN_DateCompleted_day**	numeric	77	0.00 %	Social anxiety questionnaire, self rated-Timepoint before treatment starts-Cyclic transformation of what day 0-6 during week it was filled in
	**LSAS-2241_SCREEN_DateCompleted_time**	numeric	1181	0.00 %	Social anxiety questionnaire, self rated-Timepoint before treatment starts-Cyclic transformation of what time during day 0-1440 it was filled in
	**outcome**	numeric	648	0.00 %	
	**ncomorbid**	numeric	85	0.00 %	
	**HW-01**	numeric	497	0.00 %	
	**HW-02**	numeric	473	0.00 %	
	**HW-03**	numeric	542	0.00 %	
	**currentwork_proff**	factor	37	0.00 %	Currently in work for trained proffession
	**Marital_1833_gift**	factor	2	0.00 %	Marital status: Married or not
	**Marital_1833_separerad**	factor	2	0.00 %	Marital status: divocered/equivalent
	**Marital_1833_singel**	factor	2	0.00 %	Marital status: single
	**Edu_1843_2**	factor	2	0.00 %	7-9 years education
	**Edu_1843_3**	factor	2	0.00 %	Uncompleted upper secondary school
	**Edu_1843_4**	factor	2	0.00 %	Higher vocational education
	**Edu_1843_5**	factor	2	0.00 %	Completed upper secondary school
	**Edu_1843_6**	factor	2	0.00 %	Uncompleted university degree
	**Edu_1843_7**	factor	2	0.00 %	University degree
	**cscale**	numeric	329	0.00 %	
	**mainsymptom_PRE_sum**	numeric	95	0.00 %	PDSS-SR for panic, MADRS for depression, LSAS for social anxiety-Timepoint just before beginning treatment-Sum of the entire measure

Label	Variable	Class	# unique values	Missing	Description
	**mainsymptom_PRE_duration**	numeric	916	0.00 %	PDSS-SR for panic, MADRS for depression, LSAS for social anxiety-Timepoint just before beginning treatment-Time to fill in measure/questionnaire
	**mainsymptom_PRE_DateCompleted_day**	numeric	59	0.00 %	PDSS-SR for panic, MADRS for depression, LSAS for social anxiety-Timepoint just before beginning treatment-Cyclic transformation of what day 0-6 during week it was filled in
	**mainsymptom_PRE_DateCompleted_time**	numeric	912	0.00 %	PDSS-SR for panic, MADRS for depression, LSAS for social anxiety-Timepoint just before beginning treatment-Cyclic transformation of what time during day 0-1440 it was filled in
	**mainsymptom_WEEK01_sum**	numeric	519	0.00 %	PDSS-SR for panic, MADRS for depression, LSAS for social anxiety-Timepoint after one week in treatment-Sum of the entire measure
	**mainsymptom_WEEK01_duration**	numeric	978	0.00 %	PDSS-SR for panic, MADRS for depression, LSAS for social anxiety-Timepoint after one week in treatment-Time to fill in measure/questionnaire
	**mainsymptom_WEEK01_DateCompleted_day**	numeric	484	0.00 %	PDSS-SR for panic, MADRS for depression, LSAS for social anxiety-Timepoint after one week in treatment-Cyclic transformation of what day 0-6 during week it was filled in
	**mainsymptom_WEEK01_DateCompleted_time**	numeric	1343	0.00 %	PDSS-SR for panic, MADRS for depression, LSAS for social anxiety-Timepoint after one week in treatment-Cyclic transformation of what time during day 0-1440 it was filled in
	**mainsymptom_WEEK02_sum**	numeric	448	0.00 %	PDSS-SR for panic, MADRS for depression, LSAS for social anxiety-Timepoint after two weeks in treatment-Sum of the entire measure
	**mainsymptom_WEEK02_duration**	numeric	881	0.00 %	PDSS-SR for panic, MADRS for depression, LSAS for social anxiety-Timepoint after two weeks in treatment-Time to fill in measure/questionnaire

Label	Variable	Class	# unique values	Missing	Description
	**mainsymptom_WEEK02_DateCompleted_day**	numeric	413	0.00 %	PDSS-SR for panic, MADRS for depression, LSAS for social anxiety-Timepoint after two weeks in treatment-Cyclic transformation of what day 0-6 during week it was filled in
	**mainsymptom_WEEK02_DateCompleted_time**	numeric	1295	0.00 %	PDSS-SR for panic, MADRS for depression, LSAS for social anxiety-Timepoint after two weeks in treatment-Cyclic transformation of what time during day 0-1440 it was filled in
	**mainsymptom_WEEK03_sum**	numeric	532	0.00 %	PDSS-SR for panic, MADRS for depression, LSAS for social anxiety-Timepoint after three weeks in treatment-Sum of the entire measure
	**mainsymptom_WEEK03_duration**	numeric	947	0.00 %	PDSS-SR for panic, MADRS for depression, LSAS for social anxiety-Timepoint after three weeks in treatment-Time to fill in measure/questionnaire
	**mainsymptom_WEEK03_DateCompleted_day**	numeric	491	0.00 %	PDSS-SR for panic, MADRS for depression, LSAS for social anxiety-Timepoint after three weeks in treatment-Cyclic transformation of what day 0-6 during week it was filled in
	**mainsymptom_WEEK03_DateCompleted_time**	numeric	1359	0.00 %	PDSS-SR for panic, MADRS for depression, LSAS for social anxiety-Timepoint after three weeks in treatment-Cyclic transformation of what time during day 0-1440 it was filled in

Variable list

sex

Feature	Result
Variable type	factor
Number of missing obs.	0 (0 %)
Number of unique values	2
Mode	“0”
Reference category	0

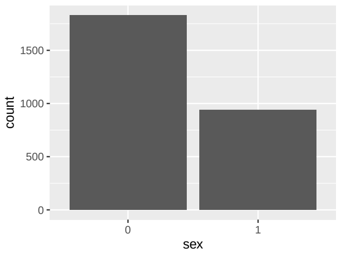

- Observed factor levels: "0", "1".

---

age

Feature	Result
Variable type	numeric
Number of missing obs.	0 (0 %)
Number of unique values	69
Median	-0.03
1st and 3rd quartiles	-0.64; 0.85
Min. and max.	-1.69; 4.16

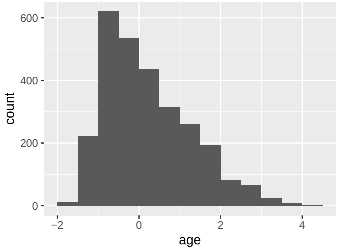

---

messages_len_7

Feature	Result
Variable type	numeric
Number of missing obs.	0 (0 %)
Number of unique values	452
Median	-0.32
1st and 3rd quartiles	-0.32; -0.08
Min. and max.	-0.32; 35.05

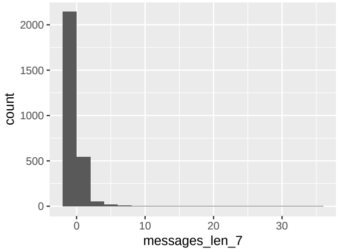

---

messages_len_tp_7

Feature	Result
Variable type	numeric
Number of missing obs.	0 (0 %)
Number of unique values	920
Median	-0.16
1st and 3rd quartiles	-0.95; 0.74
Min. and max.	-1.46; 9.81

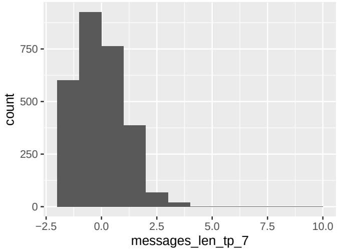

---

messages_7

Feature	Result
Variable type	numeric
Number of missing obs.	0 (0 %)
Number of unique values	9
Median	-0.55
1st and 3rd quartiles	-0.55; 0.58
Min. and max.	-0.55; 10.81

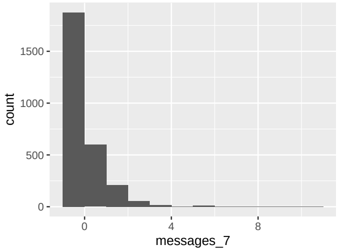

---

messages_tp_7

Feature	Result
Variable type	numeric
Number of missing obs.	0 (0 %)
Number of unique values	9
Median	0.15
1st and 3rd quartiles	-0.91; 0.15
Min. and max.	-1.97; 6.52

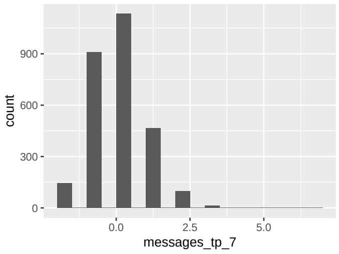

homeworks_7

Feature	Result
Variable type	numeric
Number of missing obs.	0 (0 %)
Number of unique values	5
Median	0.2
1st and 3rd quartiles	-1.03; 0.2
Min. and max.	-1.03; 3.91

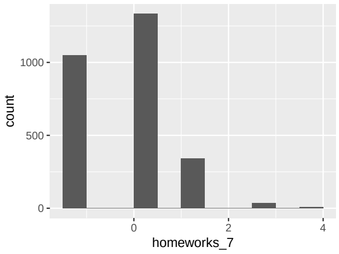

messages_len_14

Feature	Result
Variable type	numeric
Number of missing obs.	0 (0 %)
Number of unique values	536
Median	-0.41
1st and 3rd quartiles	-0.41; 0.07
Min. and max.	-0.41; 34.19

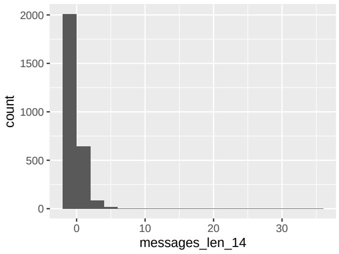

messages_len_tp_14

Feature	Result
Variable type	numeric
Number of missing obs.	0 (0 %)
Number of unique values	950
Median	-0.1
1st and 3rd quartiles	-0.8; 0.59
Min. and max.	-1.11; 7.22

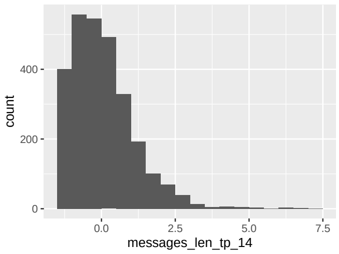

messages_14

Feature	Result
Variable type	numeric
Number of missing obs.	0 (0 %)
Number of unique values	9
Median	-0.7
1st and 3rd quartiles	-0.7; 0.36
Min. and max.	-0.7; 7.78

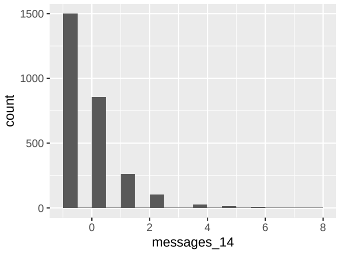

messages_tp_14

Feature	Result
Variable type	numeric
Number of missing obs.	0 (0 %)
Number of unique values	8
Median	-0.35
1st and 3rd quartiles	-0.35; 0.87
Min. and max.	-1.57; 6.97

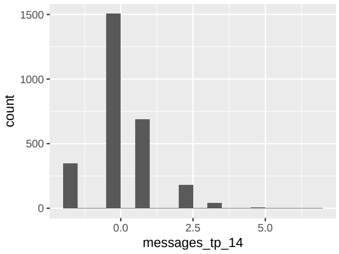

homeworks_14

Feature	Result
Variable type	numeric
Number of missing obs.	0 (0 %)
Number of unique values	6
Median	0.33
1st and 3rd quartiles	-0.98; 0.33
Min. and max.	-0.98; 5.58

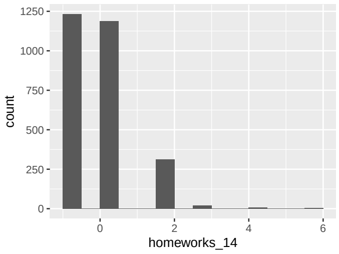

messages_len_21

Feature	Result
Variable type	numeric
Number of missing obs.	0 (0 %)
Number of unique values	516
Median	-0.46
1st and 3rd quartiles	-0.46; 0.14
Min. and max.	-0.46; 18.45

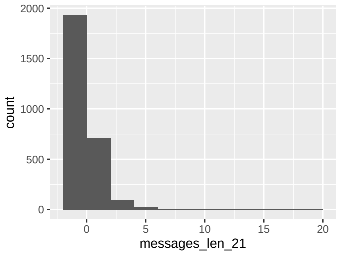

messages_len_tp_21

Feature	Result
Variable type	numeric
Number of missing obs.	0 (0 %)
Number of unique values	944
Median	-0.14
1st and 3rd quartiles	-0.81; 0.61
Min. and max.	-1.01; 6.14

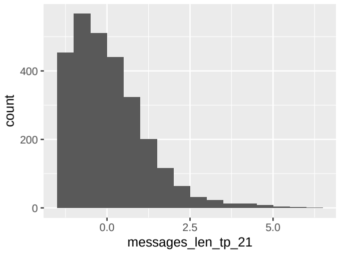

messages_21

Feature	Result
Variable type	numeric
Number of missing obs.	0 (0 %)
Number of unique values	9
Median	-0.74
1st and 3rd quartiles	-0.74; 0.34
Min. and max.	-0.74; 8.99

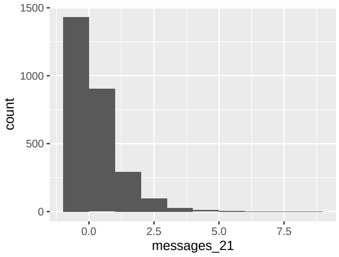

messages_tp_21

Feature	Result
Variable type	numeric
Number of missing obs.	0 (0 %)
Number of unique values	7
Median	-0.32
1st and 3rd quartiles	-0.32; 0.84
Min. and max.	-1.49; 5.5

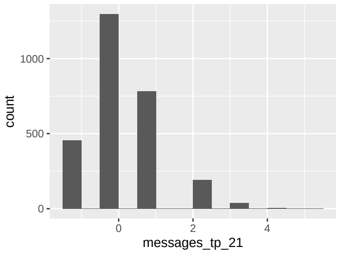

homeworks_21

Feature	Result
Variable type	numeric
Number of missing obs.	0 (0 %)
Number of unique values	7
Median	0.36
1st and 3rd quartiles	-1.01; 0.36
Min. and max.	-1.01; 7.24

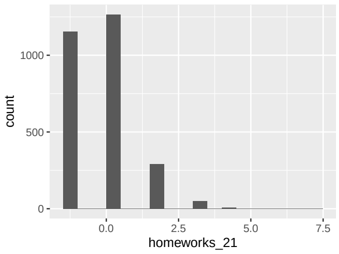

messages_len_28

Feature	Result
Variable type	numeric
Number of missing obs.	0 (0 %)
Number of unique values	458
Median	-0.41
1st and 3rd quartiles	-0.41; 0.07
Min. and max.	-0.41; 39.96

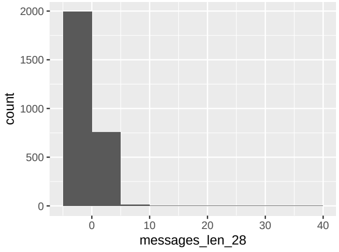

messages_len_tp_28

Feature	Result
Variable type	numeric
Number of missing obs.	0 (0 %)
Number of unique values	902
Median	-0.18
1st and 3rd quartiles	-0.77; 0.59
Min. and max.	-0.94; 10.31

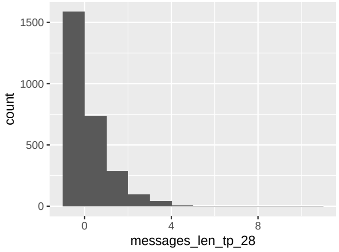

messages_28

Feature	Result
Variable type	numeric
Number of missing obs.	0 (0 %)
Number of unique values	7
Median	-0.72
1st and 3rd quartiles	-0.72; 0.41
Min. and max.	-0.72; 6.07

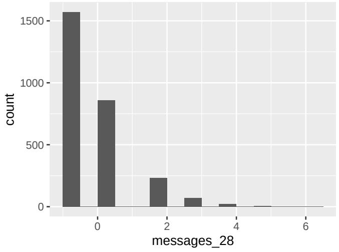

messages_tp_28

Feature	Result
Variable type	numeric
Number of missing obs.	0 (0 %)
Number of unique values	7
Median	-0.24
1st and 3rd quartiles	-0.24; 0.93
Min. and max.	-1.42; 5.63

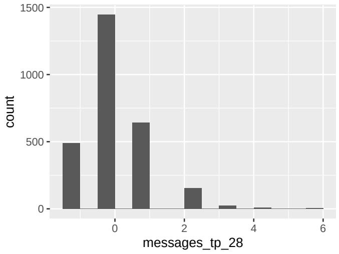

homeworks_28

Feature	Result
Variable type	numeric
Number of missing obs.	0 (0 %)
Number of unique values	6
Median	0.46
1st and 3rd quartiles	-0.91; 0.46
Min. and max.	-0.91; 5.94

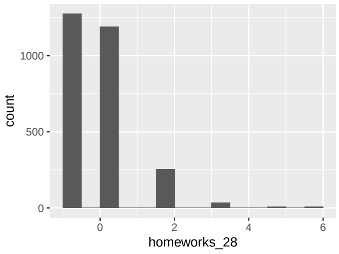

PDSS-SR-3064_SCREEN_sum

Feature	Result
Variable type	numeric
Number of missing obs.	0 (0 %)
Number of unique values	198
Median	-0.51
1st and 3rd quartiles	-1.29; 0.12
Min. and max.	-1.29; 2.96

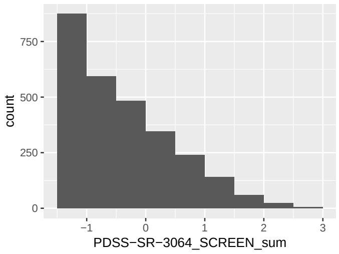

MADRS-1951_SCREEN_sum

Feature	Result
Variable type	numeric
Number of missing obs.	0 (0 %)
Number of unique values	98
Median	0.54
1st and 3rd quartiles	0.05; 1.04
Min. and max.	-2.18; 3.15

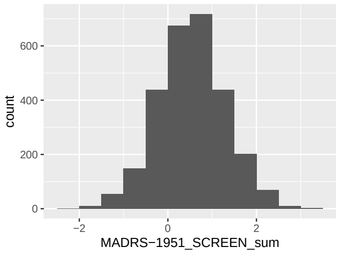

LSAS-2241_SCREEN_sum

Feature	Result
Variable type	numeric
Number of missing obs.	0 (0 %)
Number of unique values	438
Median	-0.3
1st and 3rd quartiles	-0.85; 0.32
Min. and max.	-1.79; 3.09

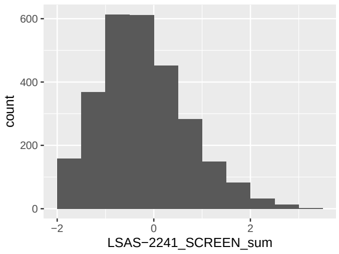

MADRS-1951_SCREEN_DateCompleted_day

Feature	Result
Variable type	numeric
Number of missing obs.	0 (0 %)
Number of unique values	18
Median	0.37
1st and 3rd quartiles	-1; 1
Min. and max.	-1.37; 1.37

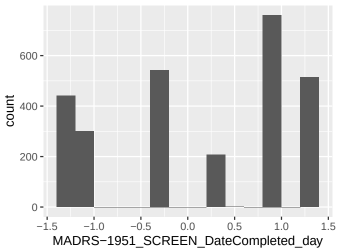

MADRS-1951_SCREEN_DateCompleted_time

Feature	Result
Variable type	numeric
Number of missing obs.	0 (0 %)
Number of unique values	931
Median	-0.76
1st and 3rd quartiles	-1.26; 0.19
Min. and max.	-1.41; 1.41

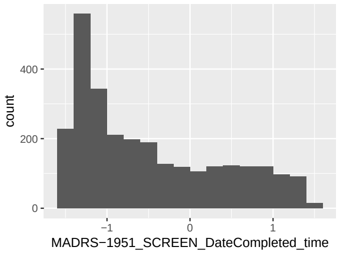

PDSS-SR-3064_SCREEN_DateCompleted_day

Feature	Result
Variable type	numeric
Number of missing obs.	0 (0 %)
Number of unique values	37
Median	0.37
1st and 3rd quartiles	-1; 1
Min. and max.	-1.37; 1.37

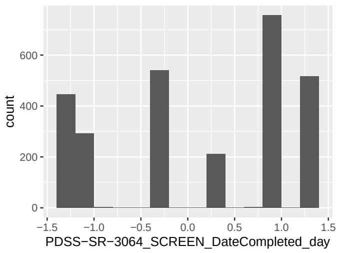

PDSS-SR-3064_SCREEN_DateCompleted_time

Feature	Result
Variable type	numeric
Number of missing obs.	0 (0 %)
Number of unique values	1054
Median	-0.76
1st and 3rd quartiles	-1.25; 0.17
Min. and max.	-1.41; 1.41

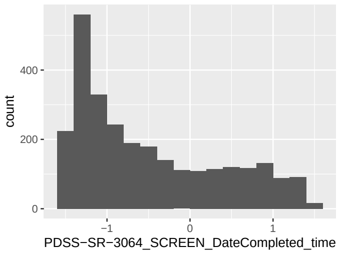

LSAS-2241_SCREEN_DateCompleted_day

Feature	Result
Variable type	numeric
Number of missing obs.	0 (0 %)
Number of unique values	77
Median	0.37
1st and 3rd quartiles	-1; 1
Min. and max.	-1.37; 1.37

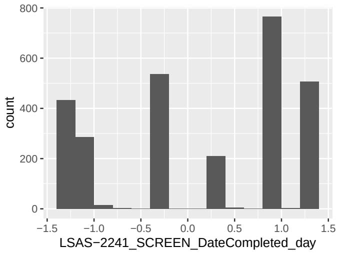

LSAS-2241_SCREEN_DateCompleted_time

Feature	Result
Variable type	numeric
Number of missing obs.	0 (0 %)
Number of unique values	1181
Median	-0.75
1st and 3rd quartiles	-1.25; 0.26
Min. and max.	-1.41; 1.41

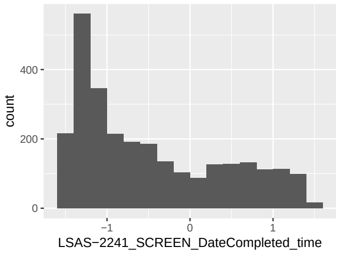

outcome

Feature	Result
Variable type	numeric
Number of missing obs.	0 (0 %)
Number of unique values	648
Median	-0.06
1st and 3rd quartiles	-0.65; 0.65
Min. and max.	-1.59; 4.66

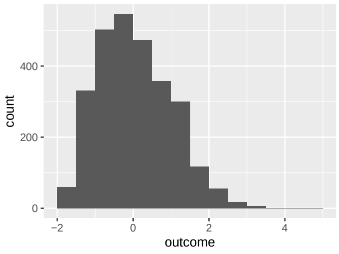

ncomorbid

Feature	Result
Variable type	numeric
Number of missing obs.	0 (0 %)
Number of unique values	85
Median	0
1st and 3rd quartiles	0; 1
Min. and max.	0; 4

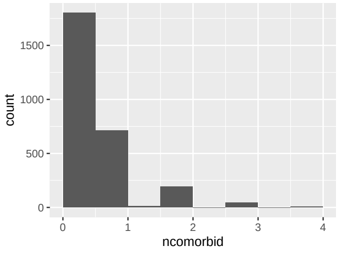

HW-01

Feature	Result
Variable type	numeric
Number of missing obs.	0 (0 %)
Number of unique values	497
Median	-0.22
1st and 3rd quartiles	-0.98; 0.81
Min. and max.	-0.98; 1.71

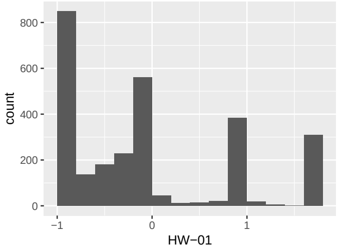

HW-02

Feature	Result
Variable type	numeric
Number of missing obs.	0 (0 %)
Number of unique values	473
Median	-0.36
1st and 3rd quartiles	-0.73; 0.02
Min. and max.	-0.92; 1.89

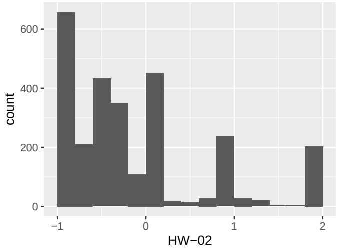

HW-03

Feature	Result
Variable type	numeric
Number of missing obs.	0 (0 %)
Number of unique values	542
Median	-0.26
1st and 3rd quartiles	-0.51; 0.07
Min. and max.	-0.92; 2.04

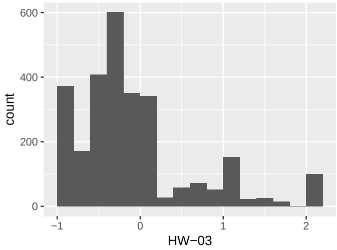

---

currentwork_proff

Feature	Result
Variable type	factor
Number of missing obs.	0 (0 %)
Number of unique values	37
Mode	“1”
Reference category	0

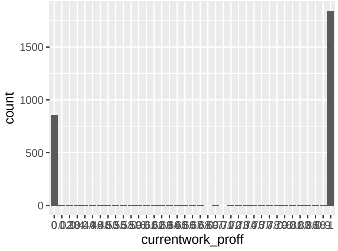

- Observed factor levels: "0", "0.02", "0.33", "0.4", "0.44", "0.46", "0.48", "0.53", "0.55", "0.58", "0.59", "0.6", "0.61", "0.62", "0.63", "0.64", "0.65", "0.66", "0.67", "0.68", "0.69", "0.7", "0.71", "0.72", "0.73", "0.74", "0.75", "0.77", "0.78", "0.79", "0.8", "0.81", "0.83", "0.86", "0.88", "0.9", "1".

Marital_1833_gift

Feature	Result
Variable type	factor
Number of missing obs.	0 (0 %)
Number of unique values	2
Mode	“1”
Reference category	0

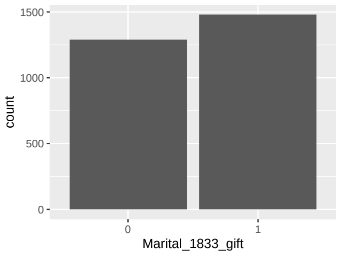

- Observed factor levels: "0", "1".

Marital_1833_separerad

Feature	Result
Variable type	factor
Number of missing obs.	0 (0 %)
Number of unique values	2
Mode	“0”
Reference category	0

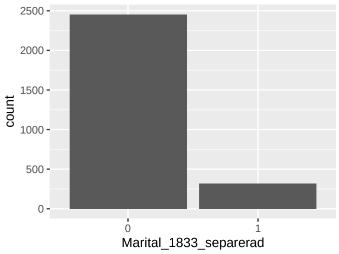

- Observed factor levels: "0", "1".

Marital_1833_singel

Feature	Result
Variable type	factor
Number of missing obs.	0 (0 %)
Number of unique values	2
Mode	“0”
Reference category	0

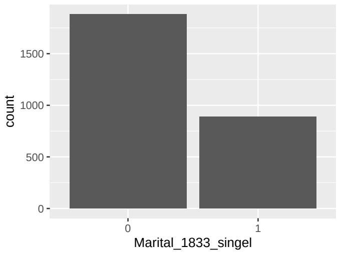

- Observed factor levels: "0", "1".

Edu_1843_2

Feature	Result
Variable type	factor
Number of missing obs.	0 (0 %)
Number of unique values	2
Mode	“0”
Reference category	0

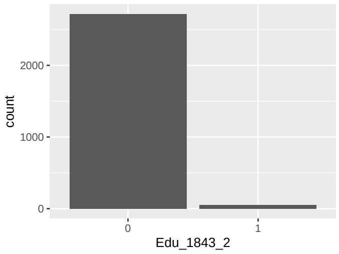

- Observed factor levels: "0", "1".

Edu_1843_3

Feature	Result
Variable type	factor
Number of missing obs.	0 (0 %)
Number of unique values	2
Mode	“0”
Reference category	0

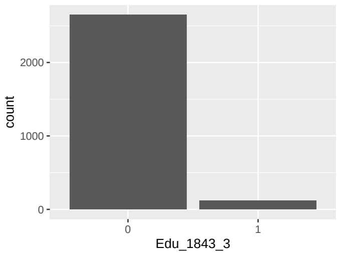

- Observed factor levels: "0", "1".

Edu_1843_4

Feature	Result
Variable type	factor
Number of missing obs.	0 (0 %)
Number of unique values	2
Mode	“0”
Reference category	0

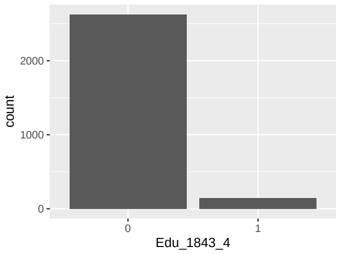

- Observed factor levels: "0", "1".

Edu_1843_5

Feature	Result
Variable type	factor
Number of missing obs.	0 (0 %)
Number of unique values	2
Mode	“0”
Reference category	0

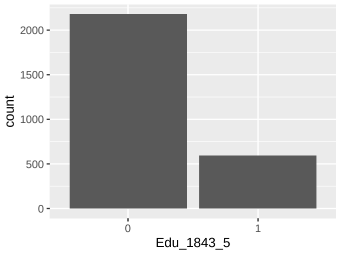

- Observed factor levels: "0", "1".

Edu_1843_6

Feature	Result
Variable type	factor
Number of missing obs.	0 (0 %)
Number of unique values	2
Mode	“0”
Reference category	0

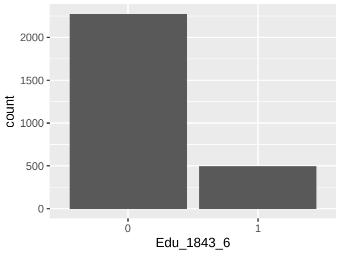

- Observed factor levels: "0", "1".

Edu_1843_7

Feature	Result
Variable type	factor
Number of missing obs.	0 (0 %)
Number of unique values	2
Mode	"0"
Reference category	0

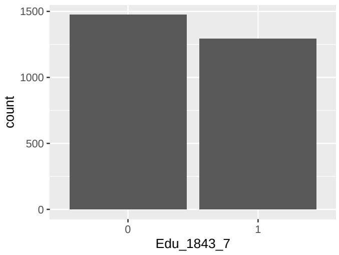

- Observed factor levels: "0", "1".

---

cscale

Feature	Result
Variable type	numeric
Number of missing obs.	0 (0 %)
Number of unique values	329
Median	-0.11
1st and 3rd quartiles	-0.7; 0.49
Min. and max.	-4.14; 1.79

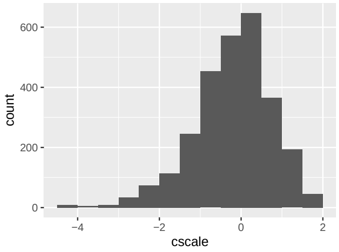

---

mainsymptom_PRE_sum

Feature	Result
Variable type	numeric
Number of missing obs.	0 (0 %)
Number of unique values	95
Median	0.52
1st and 3rd quartiles	0.03; 1.01
Min. and max.	-2.32; 3.48

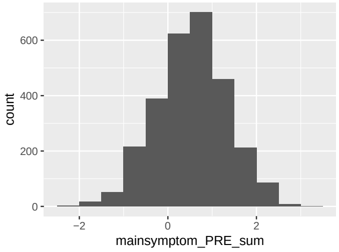

---

mainsymptom_PRE_duration

Feature	Result
Variable type	numeric
Number of missing obs.	0 (0 %)
Number of unique values	916
Median	-0.11
1st and 3rd quartiles	-0.18; 0.02
Min. and max.	-0.28; 34.1

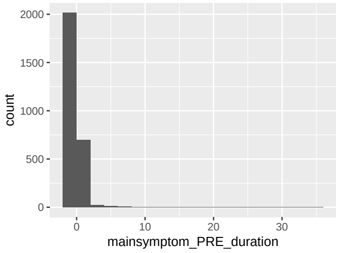

---

mainsymptom_PRE_DateCompleted_day

Feature	Result
Variable type	numeric
Number of missing obs.	0 (0 %)
Number of unique values	59
Median	0.37
1st and 3rd quartiles	-0.37; 1
Min. and max.	-1.37; 1.37

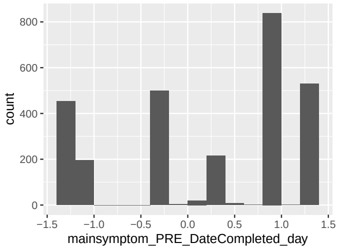

mainsymptom_PRE_DateCompleted_time

Feature	Result
Variable type	numeric
Number of missing obs.	0 (0 %)
Number of unique values	912
Median	-0.71
1st and 3rd quartiles	-1.25; 0.07
Min. and max.	-1.41; 1.41

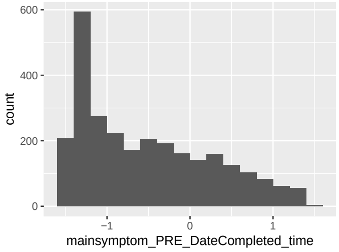

mainsymptom_WEEK01_sum

Feature	Result
Variable type	numeric
Number of missing obs.	0 (0 %)
Number of unique values	519
Median	0.48
1st and 3rd quartiles	-0.05; 1.04
Min. and max.	-2.01; 3.6

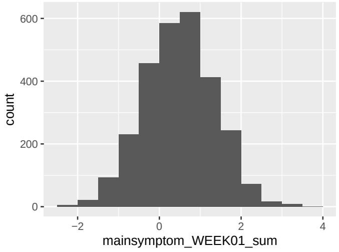

mainsymptom_WEEK01_duration

Feature	Result
Variable type	numeric
Number of missing obs.	0 (0 %)
Number of unique values	978
Median	-0.07
1st and 3rd quartiles	-0.12; 0.02
Min. and max.	-0.23; 35.17

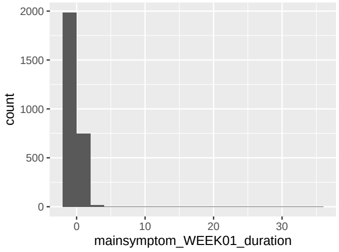

mainsymptom_WEEK01_DateCompleted_day

Feature	Result
Variable type	numeric
Number of missing obs.	0 (0 %)
Number of unique values	484
Median	0.37
1st and 3rd quartiles	-0.37; 1
Min. and max.	-1.37; 1.37

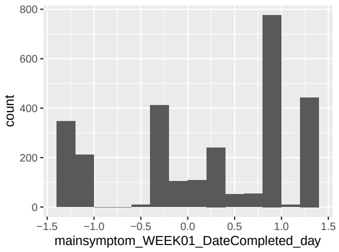

mainsymptom_WEEK01_DateCompleted_time

Feature	Result
Variable type	numeric
Number of missing obs.	0 (0 %)
Number of unique values	1343
Median	-0.64
1st and 3rd quartiles	-1.1; 0.01
Min. and max.	-1.41; 1.41

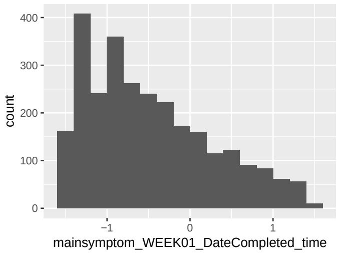

mainsymptom_WEEK02_sum

Feature	Result
Variable type	numeric
Number of missing obs.	0 (0 %)
Number of unique values	448
Median	0.5
1st and 3rd quartiles	-0.1; 0.98
Min. and max.	-1.9; 4.09

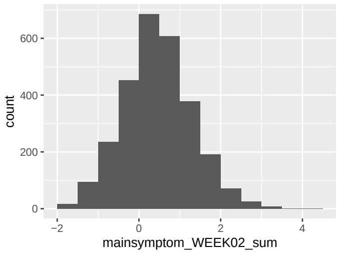

mainsymptom_WEEK02_duration

Feature	Result
Variable type	numeric
Number of missing obs.	0 (0 %)
Number of unique values	881
Median	-0.06
1st and 3rd quartiles	-0.1; 0.02
Min. and max.	-0.18; 63.88

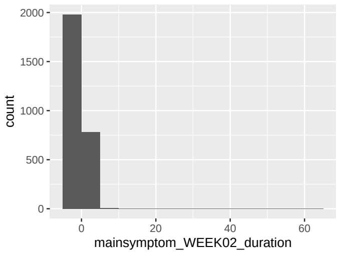

mainsymptom_WEEK02_DateCompleted_day

Feature	Result
Variable type	numeric
Number of missing obs.	0 (0 %)
Number of unique values	413
Median	0.37
1st and 3rd quartiles	-0.37; 1
Min. and max.	-1.37; 1.37

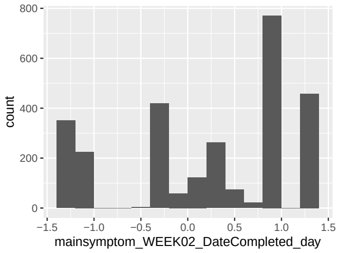

mainsymptom_WEEK02_DateCompleted_time

Feature	Result
Variable type	numeric
Number of missing obs.	0 (0 %)
Number of unique values	1295
Median	-0.66
1st and 3rd quartiles	-1.15; -0.05
Min. and max.	-1.41; 1.41

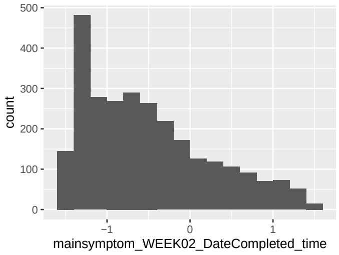

mainsymptom_WEEK03_sum

Feature	Result
Variable type	numeric
Number of missing obs.	0 (0 %)
Number of unique values	532
Median	0.35
1st and 3rd quartiles	-0.24; 1.06
Min. and max.	-1.79; 4.64

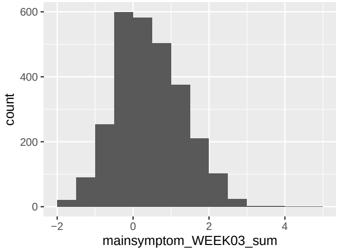

mainsymptom_WEEK03_duration

Feature	Result
Variable type	numeric
Number of missing obs.	0 (0 %)
Number of unique values	947
Median	-0.06
1st and 3rd quartiles	-0.1; 0.02
Min. and max.	-0.18; 45.7

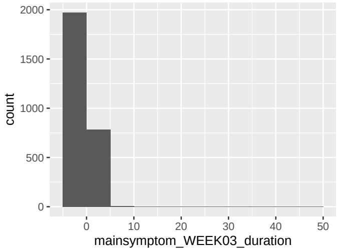

mainsymptom_WEEK03_DateCompleted_day

Feature	Result
Variable type	numeric
Number of missing obs.	0 (0 %)
Number of unique values	491
Median	0.37
1st and 3rd quartiles	-0.37; 1
Min. and max.	-1.37; 1.37

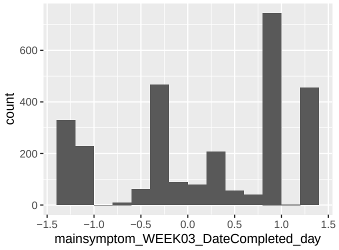

mainsymptom_WEEK03_DateCompleted_time

Feature	Result
Variable type	numeric
Number of missing obs.	0 (0 %)
Number of unique values	1359
Median	-0.59
1st and 3rd quartiles	-1.1; 0.02
Min. and max.	-1.41; 1.41

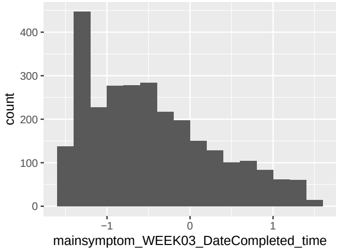

Report generation information:

- Created by: Could not determine from system (username: `nilisa`).
- Report creation time: Mon Jan 09 2023 13:05:55
- Report was run from directory: `/home/nilisa/projects/phd_study1/r`
- dataMaid v1.4.1 [Pkg: 2021-10-08 from CRAN (R 4.2.2)]
- R version 4.2.2 Patched (2022-11-10 r83330).
- Platform: x86_64-pc-linux-gnu (64-bit)(Ubuntu 20.04.5 LTS).
- Function call: `dataMaid::makeDataReport(data = gd, mode = c("summarize", "visualize", "check"), smartNum = FALSE, file = "~/projects/data/study1multiverse/results/graphs_n_figures/codebooks/codebook replace = TRUE, openResult = FALSE, checks = list(character = "showAllFactorLevels", factor = "showAllFactorLevels", labelled = "showAllFactorLevels",   haven_labelled = "showAllFactorLevels", numeric = NULL,   integer = NULL, logical = NULL, Date = NULL), listChecks = FALSE, maxProbVals = Inf, codebook = TRUE, reportTitle = "Handpicked_Depression_week04-`

Handpicked_Depression_week04-naremove_benchmark_test

Autogenerated data summary from dataMaid

2023-01-09 13:13:01

Data report overview

The dataset examined has the following dimensions:

Feature	Result
Number of observations	152
Number of variables	10

Codebook summary table

Label	Variable	Class	# unique values	Missing	Description
	**sex**	factor	2	0.00 %	Sex of patient, 0 = Female, 1=Male
	**age**	numeric	47	0.00 %	
	**PDSS-SR-3064_SCREEN_sum**	numeric	22	0.00 %	Anxiety questionnaire, self rated-Timepoint before treatment starts-Sum of the entire measure
	**MADRS-1951_SCREEN_sum**	numeric	30	0.00 %	Depression questionnaire, self rated-Timepoint before treatment starts-Sum of the entire measure
	**LSAS-2241_SCREEN_sum**	numeric	79	0.00 %	Social anxiety questionnaire, self rated-Timepoint before treatment starts-Sum of the entire measure
	**outcome**	numeric	32	0.00 %	
	**mainsymptom_PRE_sum**	numeric	29	0.00 %	PDSS-SR for panic, MADRS for depression, LSAS for social anxiety-Timepoint just before beginning treatment-Sum of the entire measure
	**mainsymptom_WEEK01_sum**	numeric	34	0.00 %	PDSS-SR for panic, MADRS for depression, LSAS for social anxiety-Timepoint after one week in treatment-Sum of the entire measure
	**mainsymptom_WEEK02_sum**	numeric	30	0.00 %	PDSS-SR for panic, MADRS for depression, LSAS for social anxiety-Timepoint after two weeks in treatment-Sum of the entire measure

Label	Variable	Class	# unique values	Missing	Description
	**mainsymptom_WEEK03_sum**	numeric	32	0.00 %	PDSS-SR for panic, MADRS for depression, LSAS for social anxiety-Timepoint after three weeks in treatment-Sum of the entire measure

Variable list

sex

Feature	Result
Variable type	factor
Number of missing obs.	0 (0 %)
Number of unique values	2
Mode	"0"
Reference category	0

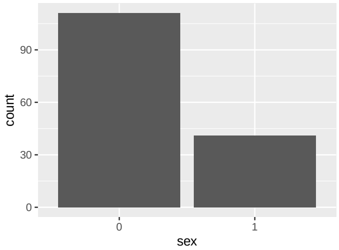

- Observed factor levels: "0", "1".

---

age

Feature	Result
Variable type	numeric
Number of missing obs.	0 (0 %)
Number of unique values	47
Median	0.15
1st and 3rd quartiles	-0.55; 0.93
Min. and max.	-1.42; 4.08

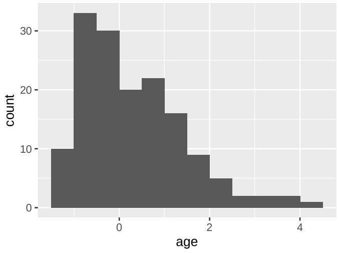

PDSS-SR-3064_SCREEN_sum

Feature	Result
Variable type	numeric
Number of missing obs.	0 (0 %)
Number of unique values	22
Median	-0.66
1st and 3rd quartiles	-1.29; 0.28
Min. and max.	-1.29; 2.17

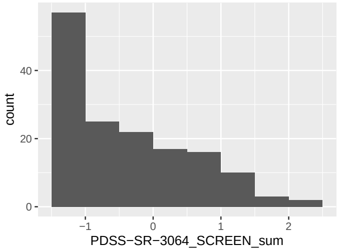

MADRS-1951_SCREEN_sum

Feature	Result
Variable type	numeric
Number of missing obs.	0 (0 %)
Number of unique values	30
Median	0.42
1st and 3rd quartiles	-0.08; 1.04
Min. and max.	-1.56; 2.28

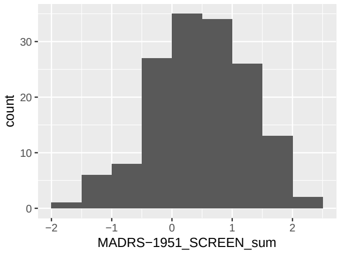

LSAS-2241_SCREEN_sum

Feature	Result
Variable type	numeric
Number of missing obs.	0 (0 %)
Number of unique values	79
Median	-0.28
1st and 3rd quartiles	-0.92; 0.4
Min. and max.	-1.79; 2.26

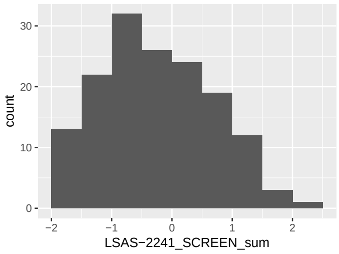

outcome

Feature	Result
Variable type	numeric
Number of missing obs.	0 (0 %)
Number of unique values	32
Median	-0.18
1st and 3rd quartiles	-0.92; 0.53
Min. and max.	-1.59; 2.65

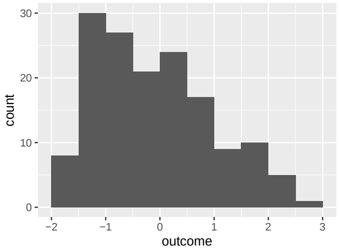

mainsymptom_PRE_sum

Feature	Result
Variable type	numeric
Number of missing obs.	0 (0 %)
Number of unique values	29
Median	0.46
1st and 3rd quartiles	-0.01; 1.04
Min. and max.	-1.33; 2.25

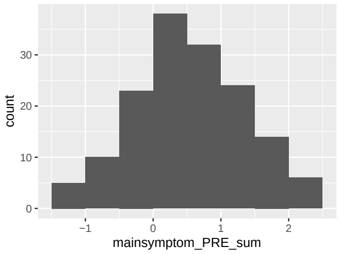

mainsymptom_WEEK01_sum

Feature	Result
Variable type	numeric
Number of missing obs.	0 (0 %)
Number of unique values	34
Median	0.43
1st and 3rd quartiles	-0.18; 1.04
Min. and max.	-1.64; 2.63

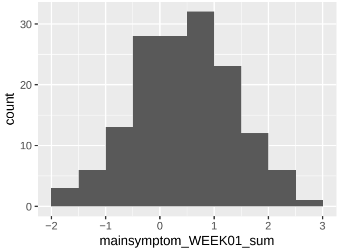

---

mainsymptom_WEEK02_sum

Feature	Result
Variable type	numeric
Number of missing obs.	0 (0 %)
Number of unique values	30
Median	0.26
1st and 3rd quartiles	-0.37; 0.86
Min. and max.	-1.42; 2.65

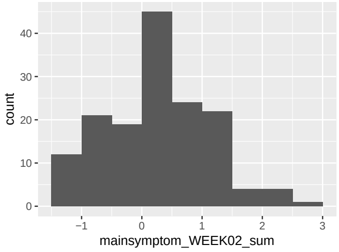

---

mainsymptom_WEEK03_sum

Feature	Result
Variable type	numeric
Number of missing obs.	0 (0 %)
Number of unique values	32
Median	0.35
1st and 3rd quartiles	-0.24; 0.83
Min. and max.	-1.44; 2.37

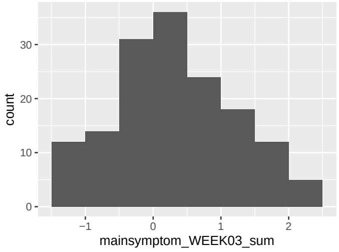

Report generation information:

- Created by: Could not determine from system (username: `nilisa`).
- Report creation time: Mon Jan 09 2023 13:13:02
- Report was run from directory: `/home/nilisa/projects/phd_study1/r`
- dataMaid v1.4.1 [Pkg: 2021-10-08 from CRAN (R 4.2.2)]
- R version 4.2.2 Patched (2022-11-10 r83330).
- Platform: x86_64-pc-linux-gnu (64-bit)(Ubuntu 20.04.5 LTS).
- Function call: `dataMaid::makeDataReport(data = gd, mode = c("summarize", "visualize", "check"), smartNum = FALSE, file = "~/projects/data/study1multiverse/results/graphs_n_figures/codebooks/codebook replace = TRUE, openResult = FALSE, checks = list(character = "showAllFactorLevels", factor = "showAllFactorLevels", labelled = "showAllFactorLevels",   haven_labelled = "showAllFactorLevels", numeric = NULL,   integer = NULL, logical = NULL, Date = NULL), listChecks = FALSE, maxProbVals = Inf, codebook = TRUE, reportTitle = "Handpicked_Depression_week04-r`

Handpicked_Depression_week04-naremove_benchmark_train

Autogenerated data summary from dataMaid

2023-01-09 13:06:37

Data report overview

The dataset examined has the following dimensions:

Feature	Result
Number of observations	1481
Number of variables	10

Codebook summary table

Label	Variable	Class	# unique values	Missing	Description
	**sex**	factor	2	0.00 %	Sex of patient, 0 = Female, 1=Male
	**age**	numeric	61	0.00 %	
	**PDSS-SR-3064_SCREEN_sum**	numeric	25	0.00 %	Anxiety questionnaire, self rated-Timepoint before treatment starts-Sum of the entire measure
	**MADRS-1951_SCREEN_sum**	numeric	39	0.00 %	Depression questionnaire, self rated-Timepoint before treatment starts-Sum of the entire measure
	**LSAS-2241_SCREEN_sum**	numeric	125	0.00 %	Social anxiety questionnaire, self rated-Timepoint before treatment starts-Sum of the entire measure
	**outcome**	numeric	41	0.00 %	
	**mainsymptom_PRE_sum**	numeric	41	0.00 %	PDSS-SR for panic, MADRS for depression, LSAS for social anxiety-Timepoint just before beginning treatment-Sum of the entire measure
	**mainsymptom_WEEK01_sum**	numeric	42	0.00 %	PDSS-SR for panic, MADRS for depression, LSAS for social anxiety-Timepoint after one week in treatment-Sum of the entire measure
	**mainsymptom_WEEK02_sum**	numeric	41	0.00 %	PDSS-SR for panic, MADRS for depression, LSAS for social anxiety-Timepoint after two weeks in treatment-Sum of the entire measure

Label	Variable	Class	# unique values	Missing	Description
	**mainsymptom_WEEK03_sum**	numeric	41	0.00 %	PDSS-SR for panic, MADRS for depression, LSAS for social anxiety-Timepoint after three weeks in treatment-Sum of the entire measure

Variable list

sex

Feature	Result
Variable type	factor
Number of missing obs.	0 (0 %)
Number of unique values	2
Mode	“0”
Reference category	0

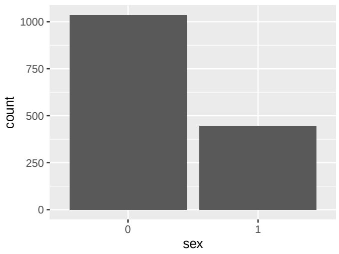

- Observed factor levels: "0", "1".

---

age

Feature	Result
Variable type	numeric
Number of missing obs.	0 (0 %)
Number of unique values	61
Median	0.06
1st and 3rd quartiles	-0.55; 1.02
Min. and max.	-1.51; 4.16

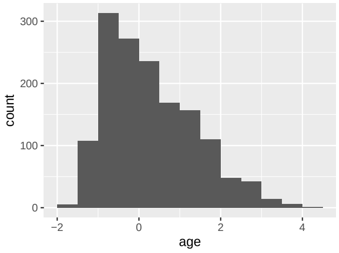

PDSS-SR-3064_SCREEN_sum

Feature	Result
Variable type	numeric
Number of missing obs.	0 (0 %)
Number of unique values	25
Median	-0.51
1st and 3rd quartiles	-1.29; 0.12
Min. and max.	-1.29; 2.65

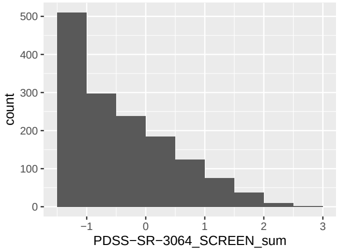

MADRS-1951_SCREEN_sum

Feature	Result
Variable type	numeric
Number of missing obs.	0 (0 %)
Number of unique values	39
Median	0.54
1st and 3rd quartiles	0.05; 0.92
Min. and max.	-1.94; 3.02

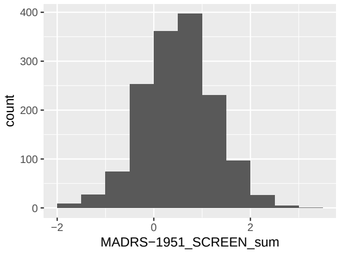

LSAS-2241_SCREEN_sum

Feature	Result
Variable type	numeric
Number of missing obs.	0 (0 %)
Number of unique values	125
Median	-0.27
1st and 3rd quartiles	-0.92; 0.36
Min. and max.	-1.79; 2.92

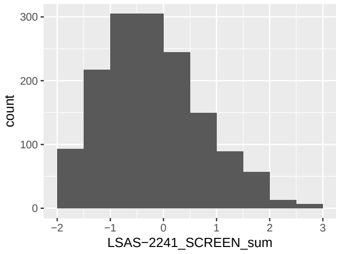

outcome

Feature	Result
Variable type	numeric
Number of missing obs.	0 (0 %)
Number of unique values	41
Median	-0.18
1st and 3rd quartiles	-0.77; 0.53
Min. and max.	-1.59; 3.12

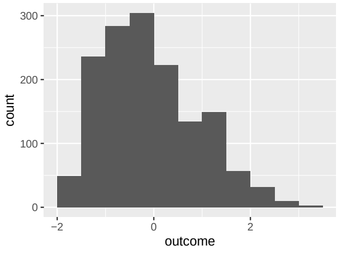

mainsymptom_PRE_sum

Feature	Result
Variable type	numeric
Number of missing obs.	0 (0 %)
Number of unique values	41
Median	0.52
1st and 3rd quartiles	0.03; 1.01
Min. and max.	-2.32; 2.99

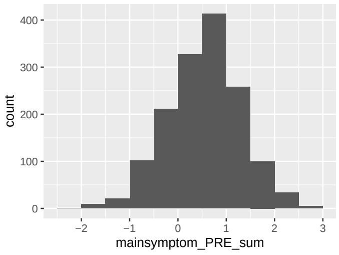

mainsymptom_WEEK01_sum

Feature	Result
Variable type	numeric
Number of missing obs.	0 (0 %)
Number of unique values	42
Median	0.43
1st and 3rd quartiles	-0.05; 1.04
Min. and max.	-2.01; 3.6

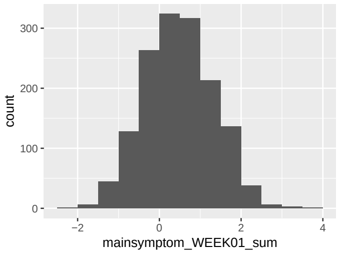

mainsymptom_WEEK02_sum

Feature	Result
Variable type	numeric
Number of missing obs.	0 (0 %)
Number of unique values	41
Median	0.38
1st and 3rd quartiles	-0.22; 0.98
Min. and max.	-1.9; 3.13

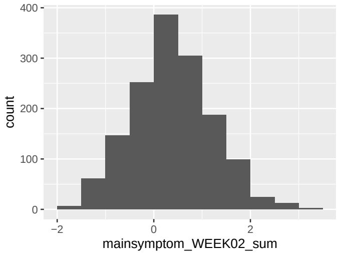

mainsymptom_WEEK03_sum

Feature	Result
Variable type	numeric
Number of missing obs.	0 (0 %)
Number of unique values	41
Median	0.35
1st and 3rd quartiles	-0.24; 0.95
Min. and max.	-1.79; 2.97

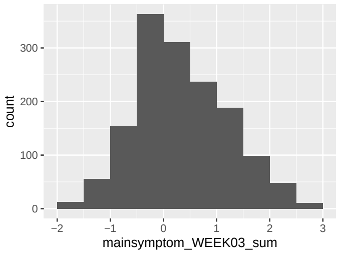

Report generation information:

- Created by: Could not determine from system (username: `nilisa`).
- Report creation time: Mon Jan 09 2023 13:06:37
- Report was run from directory: `/home/nilisa/projects/phd_study1/r`
- dataMaid v1.4.1 [Pkg: 2021-10-08 from CRAN (R 4.2.2)]
- R version 4.2.2 Patched (2022-11-10 r83330).
- Platform: x86_64-pc-linux-gnu (64-bit)(Ubuntu 20.04.5 LTS).
- Function call: `dataMaid::makeDataReport(data = gd, mode = c("summarize", "visualize", "check"), smartNum = FALSE, file = "~/projects/data/study1multiverse/results/graphs_n_figures/codebooks/codebook replace = TRUE, openResult = FALSE, checks = list(character = "showAllFactorLevels", factor = "showAllFactorLevels", labelled = "showAllFactorLevels",   haven_labelled = "showAllFactorLevels", numeric = NULL,   integer = NULL, logical = NULL, Date = NULL), listChecks = FALSE, maxProbVals = Inf, codebook = TRUE, reportTitle = "Handpicked_Depression_week04-r`

Handpicked_Depression_week04-naremove_test

Autogenerated data summary from dataMaid

2023-01-09 13:13:10

Data report overview

The dataset examined has the following dimensions:

Feature	Result
Number of observations	145
Number of variables	60

Codebook summary table

Label	Variable	Class	# unique values	Missing	Description
	**sex**	factor	2	0.00 %	Sex of patient, 0 = Female, 1=Male
	**age**	numeric	47	0.00 %	
	**messages_len_7**	numeric	54	0.00 %	-Meta information of messages-Length of messages-up until day-7
	**messages_len_tp_7**	numeric	115	0.00 %	-Meta information of messages-Length of messages-therapist messages-up until day-7
	**messages_7**	numeric	6	0.00 %	-Meta information of messages-up until day-7
	**messages_tp_7**	numeric	6	0.00 %	-Meta information of messages-therapist messages-up until day-7
	**homeworks_7**	numeric	4	0.00 %	-Number of homework messages sent in-up until day-7
	**messages_len_14**	numeric	66	0.00 %	-Meta information of messages-Length of messages-up until day-14
	**messages_len_tp_14**	numeric	126	0.00 %	-Meta information of messages-Length of messages-therapist messages-up until day-14
	**messages_14**	numeric	5	0.00 %	-Meta information of messages-up until day-14
	**messages_tp_14**	numeric	5	0.00 %	-Meta information of messages-therapist messages-up until day-14

Label	Variable	Class	# unique values	Missing	Description
	**homeworks_14**	numeric	5	0.00 %	-Number of homework messages sent in-up until day-14
	**messages_len_21**	numeric	68	0.00 %	-Meta information of messages-Length of messages-up until day-21
	**messages_len_tp_21**	numeric	127	0.00 %	-Meta information of messages-Length of messages-therapist messages-up until day-21
	**messages_21**	numeric	7	0.00 %	-Meta information of messages-up until day-21
	**messages_tp_21**	numeric	5	0.00 %	-Meta information of messages-therapist messages-up until day-21
	**homeworks_21**	numeric	5	0.00 %	-Number of homework messages sent in-up until day-21
	**messages_len_28**	numeric	73	0.00 %	-Meta information of messages-Length of messages-up until day-28
	**messages_len_tp_28**	numeric	127	0.00 %	-Meta information of messages-Length of messages-therapist messages-up until day-28
	**messages_28**	numeric	6	0.00 %	-Meta information of messages-up until day-28
	**messages_tp_28**	numeric	6	0.00 %	-Meta information of messages-therapist messages-up until day-28
	**homeworks_28**	numeric	4	0.00 %	-Number of homework messages sent in-up until day-28
	**PDSS-SR-3064_SCREEN_sum**	numeric	22	0.00 %	Anxiety questionnaire, self rated-Timepoint before treatment starts-Sum of the entire measure
	**MADRS-1951_SCREEN_sum**	numeric	30	0.00 %	Depression questionnaire, self rated-Timepoint before treatment starts-Sum of the entire measure
	**LSAS-2241_SCREEN_sum**	numeric	78	0.00 %	Social anxiety questionnaire, self rated-Timepoint before treatment starts-Sum of the entire measure
	**MADRS-1951_SCREEN_DateCompleted_day**	numeric	7	0.00 %	Depression questionnaire, self rated-Timepoint before treatment starts-Cyclic transformation of what day 0-6 during week it was filled in

Label	Variable	Class	# unique values	Missing	Description
	**MADRS-1951_SCREEN_DateCompleted_time**	numeric	132	0.00 %	Depression questionnaire, self rated-Timepoint before treatment starts-Cyclic transformation of what time during day 0-1440 it was filled in
	**PDSS-SR-3064_SCREEN_DateCompleted_day**	numeric	7	0.00 %	Anxiety questionnaire, self rated-Timepoint before treatment starts-Cyclic transformation of what day 0-6 during week it was filled in
	**PDSS-SR-3064_SCREEN_DateCompleted_time**	numeric	136	0.00 %	Anxiety questionnaire, self rated-Timepoint before treatment starts-Cyclic transformation of what time during day 0-1440 it was filled in
	**LSAS-2241_SCREEN_DateCompleted_day**	numeric	7	0.00 %	Social anxiety questionnaire, self rated-Timepoint before treatment starts-Cyclic transformation of what day 0-6 during week it was filled in
	**LSAS-2241_SCREEN_DateCompleted_time**	numeric	130	0.00 %	Social anxiety questionnaire, self rated-Timepoint before treatment starts-Cyclic transformation of what time during day 0-1440 it was filled in
	**outcome**	numeric	32	0.00 %	
	**ncomorbid**	numeric	4	0.00 %	
	**currentwork_proff**	factor	2	0.00 %	Currently in work for trained proffession
	**Marital_1833_gift**	factor	2	0.00 %	Marital status: Married or not
	**Marital_1833_separerad**	factor	2	0.00 %	Marital status: divocered/equivalent
	**Marital_1833_singel**	factor	2	0.00 %	Marital status: single
	**Edu_1843_2**	factor	2	0.00 %	7-9 years education
	**Edu_1843_3**	factor	2	0.00 %	Uncompleted upper secondary school
	**Edu_1843_4**	factor	2	0.00 %	Higher vocational education
	**Edu_1843_5**	factor	2	0.00 %	Completed upper secondary school
	**Edu_1843_6**	factor	2	0.00 %	Uncompleted university degree
	**Edu_1843_7**	factor	2	0.00 %	University degree
	**cscale**	numeric	36	0.00 %	
	**mainsymptom_PRE_sum**	numeric	29	0.00 %	PDSS-SR for panic, MADRS for depression, LSAS for social anxiety-Timepoint just before beginning treatment-Sum of the entire measure

Label	Variable	Class	# unique values	Missing	Description
	**mainsymptom_PRE_duration**	numeric	134	0.00 %	PDSS-SR for panic, MADRS for depression, LSAS for social anxiety-Timepoint just before beginning treatment-Time to fill in measure/questionnaire
	**mainsymptom_PRE_DateCompleted_day**	numeric	7	0.00 %	PDSS-SR for panic, MADRS for depression, LSAS for social anxiety-Timepoint just before beginning treatment-Cyclic transformation of what day 0-6 during week it was filled in
	**mainsymptom_PRE_DateCompleted_time**	numeric	131	0.00 %	PDSS-SR for panic, MADRS for depression, LSAS for social anxiety-Timepoint just before beginning treatment-Cyclic transformation of what time during day 0-1440 it was filled in
	**mainsymptom_WEEK01_sum**	numeric	34	0.00 %	PDSS-SR for panic, MADRS for depression, LSAS for social anxiety-Timepoint after one week in treatment-Sum of the entire measure
	**mainsymptom_WEEK01_duration**	numeric	110	0.00 %	PDSS-SR for panic, MADRS for depression, LSAS for social anxiety-Timepoint after one week in treatment-Time to fill in measure/questionnaire
	**mainsymptom_WEEK01_DateCompleted_day**	numeric	7	0.00 %	PDSS-SR for panic, MADRS for depression, LSAS for social anxiety-Timepoint after one week in treatment-Cyclic transformation of what day 0-6 during week it was filled in
	**mainsymptom_WEEK01_DateCompleted_time**	numeric	136	0.00 %	PDSS-SR for panic, MADRS for depression, LSAS for social anxiety-Timepoint after one week in treatment-Cyclic transformation of what time during day 0-1440 it was filled in
	**mainsymptom_WEEK02_sum**	numeric	30	0.00 %	PDSS-SR for panic, MADRS for depression, LSAS for social anxiety-Timepoint after two weeks in treatment-Sum of the entire measure
	**mainsymptom_WEEK02_duration**	numeric	103	0.00 %	PDSS-SR for panic, MADRS for depression, LSAS for social anxiety-Timepoint after two weeks in treatment-Time to fill in measure/questionnaire

Label	Variable	Class	# unique values	Missing	Description
	**mainsymptom_WEEK02_DateCompleted_day**	numeric	7	0.00 %	PDSS-SR for panic, MADRS for depression, LSAS for social anxiety-Timepoint after two weeks in treatment-Cyclic transformation of what day 0-6 during week it was filled in
	**mainsymptom_WEEK02_DateCompleted_time**	numeric	136	0.00 %	PDSS-SR for panic, MADRS for depression, LSAS for social anxiety-Timepoint after two weeks in treatment-Cyclic transformation of what time during day 0-1440 it was filled in
	**mainsymptom_WEEK03_sum**	numeric	32	0.00 %	PDSS-SR for panic, MADRS for depression, LSAS for social anxiety-Timepoint after three weeks in treatment-Sum of the entire measure
	**mainsymptom_WEEK03_duration**	numeric	105	0.00 %	PDSS-SR for panic, MADRS for depression, LSAS for social anxiety-Timepoint after three weeks in treatment-Time to fill in measure/questionnaire
	**mainsymptom_WEEK03_DateCompleted_day**	numeric	7	0.00 %	PDSS-SR for panic, MADRS for depression, LSAS for social anxiety-Timepoint after three weeks in treatment-Cyclic transformation of what day 0-6 during week it was filled in
	**mainsymptom_WEEK03_DateCompleted_time**	numeric	139	0.00 %	PDSS-SR for panic, MADRS for depression, LSAS for social anxiety-Timepoint after three weeks in treatment-Cyclic transformation of what time during day 0-1440 it was filled in

Variable list

sex

Feature	Result
Variable type	factor
Number of missing obs.	0 (0 %)
Number of unique values	2
Mode	"0"
Reference category	0

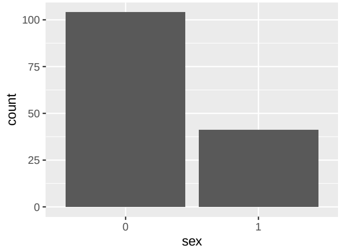

- Observed factor levels: "0", "1".

age

Feature	Result
Variable type	numeric
Number of missing obs.	0 (0 %)
Number of unique values	47
Median	0.24
1st and 3rd quartiles	-0.55; 1.02
Min. and max.	-1.42; 4.08

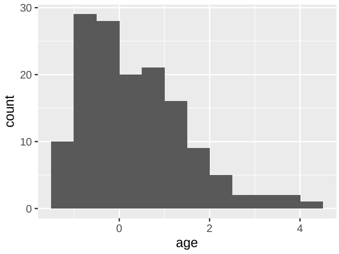

messages_len_7

Feature	Result
Variable type	numeric
Number of missing obs.	0 (0 %)
Number of unique values	54
Median	-0.32
1st and 3rd quartiles	-0.32; 0.03
Min. and max.	-0.32; 5.15

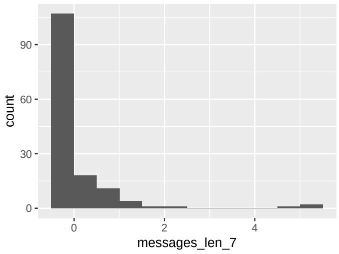

messages_len_tp_7

Feature	Result
Variable type	numeric
Number of missing obs.	0 (0 %)
Number of unique values	115
Median	0
1st and 3rd quartiles	-0.59; 0.66
Min. and max.	-1.46; 3.23

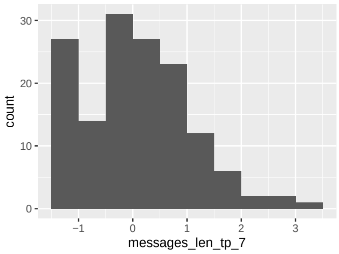

messages_7

Feature	Result
Variable type	numeric
Number of missing obs.	0 (0 %)
Number of unique values	6
Median	-0.55
1st and 3rd quartiles	-0.55; 0.58
Min. and max.	-0.55; 5.13

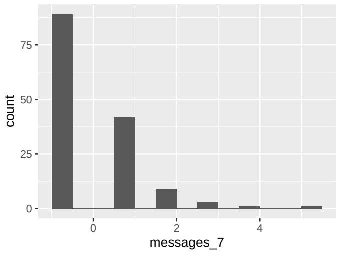

messages_tp_7

Feature	Result
Variable type	numeric
Number of missing obs.	0 (0 %)
Number of unique values	6
Median	0.15
1st and 3rd quartiles	-0.91; 0.15
Min. and max.	-1.97; 4.4

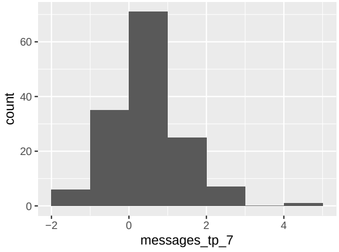

homeworks_7

Feature	Result
Variable type	numeric
Number of missing obs.	0 (0 %)
Number of unique values	4
Median	0.2
1st and 3rd quartiles	-1.03; 0.2
Min. and max.	-1.03; 2.68

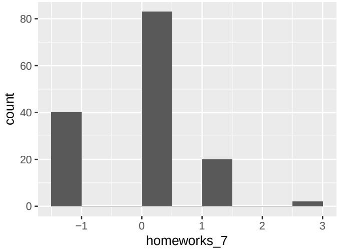

messages_len_14

Feature	Result
Variable type	numeric
Number of missing obs.	0 (0 %)
Number of unique values	66
Median	-0.41
1st and 3rd quartiles	-0.41; 0.28
Min. and max.	-0.41; 7.53

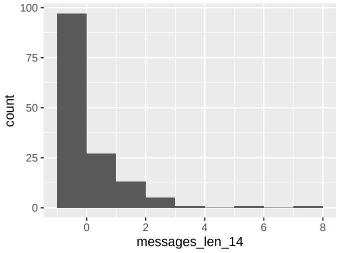

messages_len_tp_14

Feature	Result
Variable type	numeric
Number of missing obs.	0 (0 %)
Number of unique values	126
Median	0.04
1st and 3rd quartiles	-0.6; 0.89
Min. and max.	-1.11; 14.98

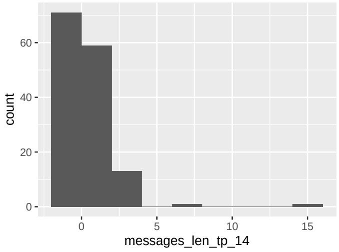

messages_14

Feature	Result
Variable type	numeric
Number of missing obs.	0 (0 %)
Number of unique values	5
Median	-0.7
1st and 3rd quartiles	-0.7; 0.36
Min. and max.	-0.7; 3.54

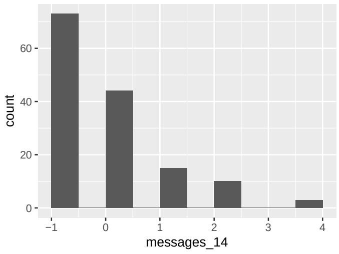

messages_tp_14

Feature	Result
Variable type	numeric
Number of missing obs.	0 (0 %)
Number of unique values	5
Median	-0.35
1st and 3rd quartiles	-0.35; 0.87
Min. and max.	-1.57; 3.31

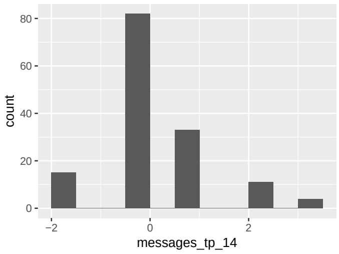

homeworks_14

Feature	Result
Variable type	numeric
Number of missing obs.	0 (0 %)
Number of unique values	5
Median	0.33
1st and 3rd quartiles	-0.98; 0.33
Min. and max.	-0.98; 14.78

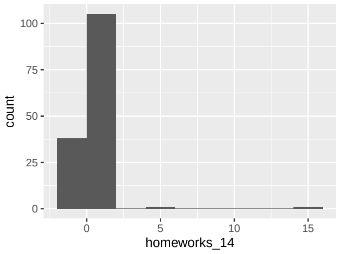

messages_len_21

Feature	Result
Variable type	numeric
Number of missing obs.	0 (0 %)
Number of unique values	68
Median	-0.4
1st and 3rd quartiles	-0.46; 0.51
Min. and max.	-0.46; 11.54

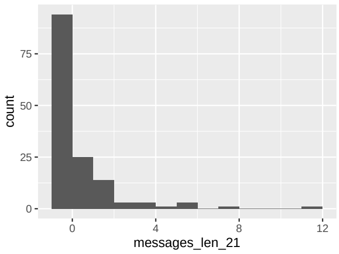

messages_len_tp_21

Feature	Result
Variable type	numeric
Number of missing obs.	0 (0 %)
Number of unique values	127
Median	0.14
1st and 3rd quartiles	-0.49; 1.28
Min. and max.	-1.01; 8.85

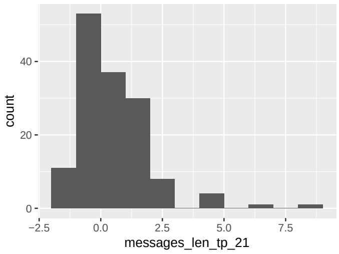

messages_21

Feature	Result
Variable type	numeric
Number of missing obs.	0 (0 %)
Number of unique values	7
Median	0.34
1st and 3rd quartiles	-0.74; 0.34
Min. and max.	-0.74; 5.75

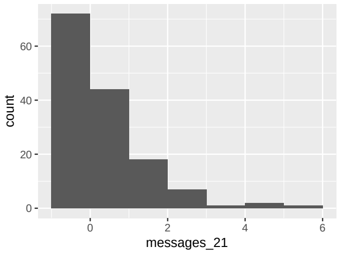

messages_tp_21

Feature	Result
Variable type	numeric
Number of missing obs.	0 (0 %)
Number of unique values	5
Median	-0.32
1st and 3rd quartiles	-0.32; 0.84
Min. and max.	-1.49; 5.5

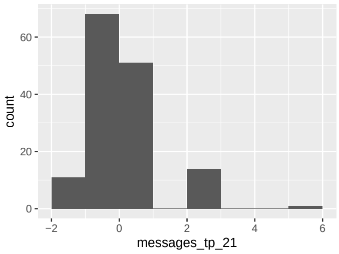

homeworks_21

Feature	Result
Variable type	numeric
Number of missing obs.	0 (0 %)
Number of unique values	5
Median	0.36
1st and 3rd quartiles	-1.01; 0.36
Min. and max.	-1.01; 4.49

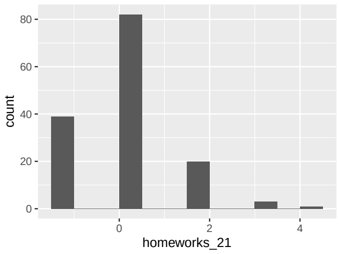

messages_len_28

Feature	Result
Variable type	numeric
Number of missing obs.	0 (0 %)
Number of unique values	73
Median	-0.25
1st and 3rd quartiles	-0.41; 0.46
Min. and max.	-0.41; 7.28

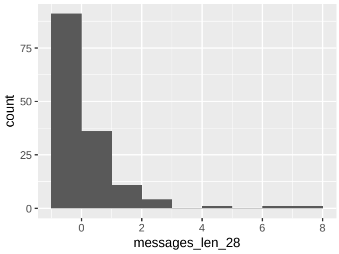

messages_len_tp_28

Feature	Result
Variable type	numeric
Number of missing obs.	0 (0 %)
Number of unique values	127
Median	0.16
1st and 3rd quartiles	-0.38; 1.03
Min. and max.	-0.94; 5.97

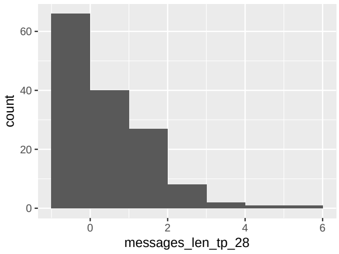

messages_28

Feature	Result
Variable type	numeric
Number of missing obs.	0 (0 %)
Number of unique values	6
Median	0.41
1st and 3rd quartiles	-0.72; 0.41
Min. and max.	-0.72; 4.94

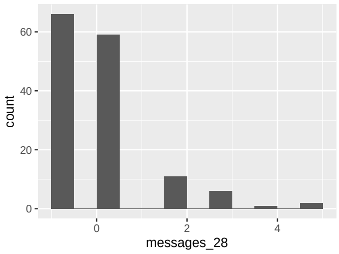

messages_tp_28

Feature	Result
Variable type	numeric
Number of missing obs.	0 (0 %)
Number of unique values	6
Median	-0.24
1st and 3rd quartiles	-0.24; 0.93
Min. and max.	-1.42; 4.45

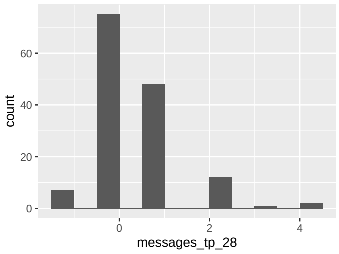

homeworks_28

Feature	Result
Variable type	numeric
Number of missing obs.	0 (0 %)
Number of unique values	4
Median	0.46
1st and 3rd quartiles	-0.91; 0.46
Min. and max.	-0.91; 4.57

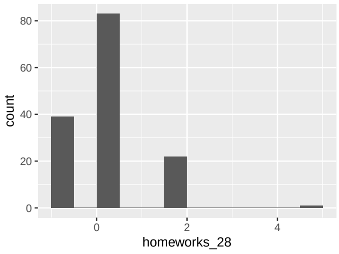

PDSS-SR-3064_SCREEN_sum

Feature	Result
Variable type	numeric
Number of missing obs.	0 (0 %)
Number of unique values	22
Median	-0.66
1st and 3rd quartiles	-1.29; 0.28
Min. and max.	-1.29; 2.17

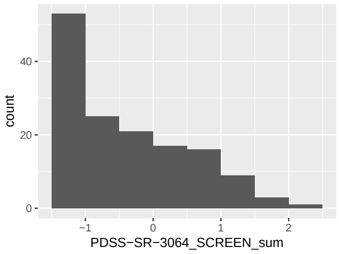

MADRS-1951_SCREEN_sum

Feature	Result
Variable type	numeric
Number of missing obs.	0 (0 %)
Number of unique values	30
Median	0.54
1st and 3rd quartiles	-0.08; 1.04
Min. and max.	-1.56; 2.28

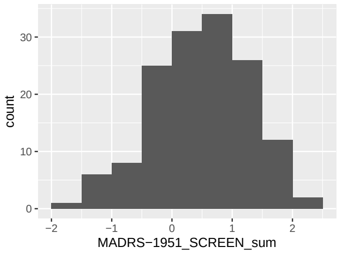

LSAS-2241_SCREEN_sum

Feature	Result
Variable type	numeric
Number of missing obs.	0 (0 %)
Number of unique values	78
Median	-0.3
1st and 3rd quartiles	-0.92; 0.39
Min. and max.	-1.79; 1.71

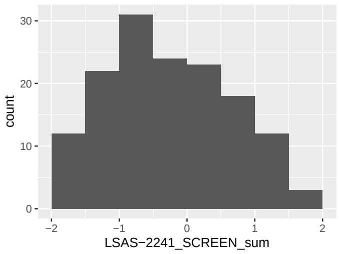

MADRS-1951_SCREEN_DateCompleted_day

Feature	Result
Variable type	numeric
Number of missing obs.	0 (0 %)
Number of unique values	7
Median	0.37
1st and 3rd quartiles	-1; 1
Min. and max.	-1.37; 1.37

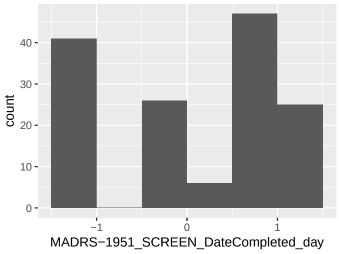

MADRS-1951_SCREEN_DateCompleted_time

Feature	Result
Variable type	numeric
Number of missing obs.	0 (0 %)
Number of unique values	132
Median	-0.59
1st and 3rd quartiles	-1.12; 0.14
Min. and max.	-1.41; 1.41

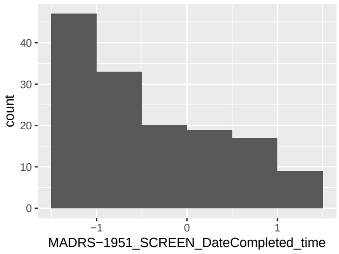

PDSS-SR-3064_SCREEN_DateCompleted_day

Feature	Result
Variable type	numeric
Number of missing obs.	0 (0 %)
Number of unique values	7
Median	1
1st and 3rd quartiles	-1; 1
Min. and max.	-1.37; 1.37

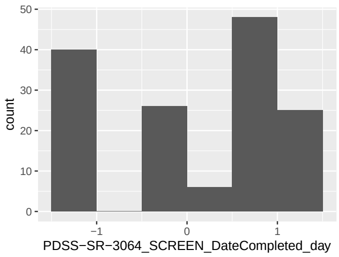

PDSS-SR-3064_SCREEN_DateCompleted_time

Feature	Result
Variable type	numeric
Number of missing obs.	0 (0 %)
Number of unique values	136
Median	-0.69
1st and 3rd quartiles	-1.17; 0.18
Min. and max.	-1.41; 1.38

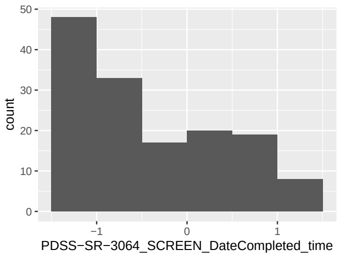

LSAS-2241_SCREEN_DateCompleted_day

Feature	Result
Variable type	numeric
Number of missing obs.	0 (0 %)
Number of unique values	7
Median	1
1st and 3rd quartiles	-1; 1
Min. and max.	-1.37; 1.37

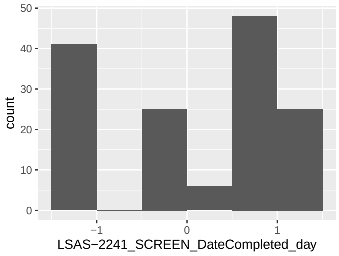

LSAS-2241_SCREEN_DateCompleted_time

Feature	Result
Variable type	numeric
Number of missing obs.	0 (0 %)
Number of unique values	130
Median	-0.57
1st and 3rd quartiles	-1.1; 0.27
Min. and max.	-1.41; 1.41

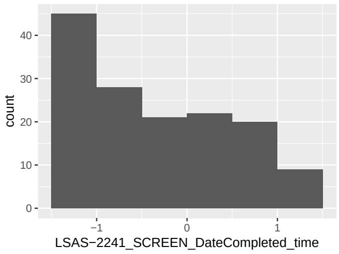

outcome

Feature	Result
Variable type	numeric
Number of missing obs.	0 (0 %)
Number of unique values	32
Median	-0.18
1st and 3rd quartiles	-0.89; 0.53
Min. and max.	-1.59; 2.65

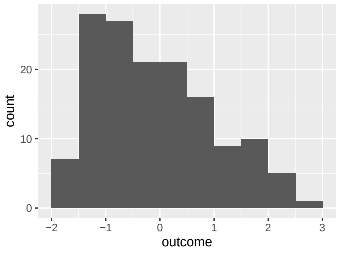

ncomorbid

Feature	Result
Variable type	numeric
Number of missing obs.	0 (0 %)
Number of unique values	4
Median	0
1st and 3rd quartiles	0; 1
Min. and max.	0; 3

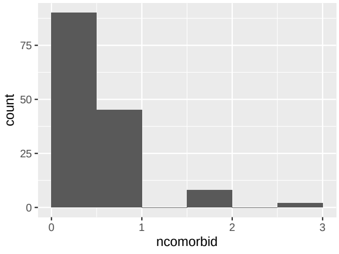

currentwork_proff

Feature	Result
Variable type	factor
Number of missing obs.	0 (0 %)
Number of unique values	2
Mode	"1"
Reference category	0

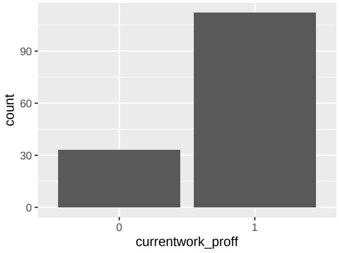

- Observed factor levels: "0", "1".

Marital_1833_gift

Feature	Result
Variable type	factor
Number of missing obs.	0 (0 %)
Number of unique values	2
Mode	"1"
Reference category	0

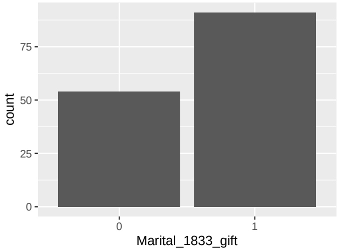

- Observed factor levels: "0", "1".

Marital_1833_separerad

Feature	Result
Variable type	factor
Number of missing obs.	0 (0 %)
Number of unique values	2
Mode	"0"
Reference category	0

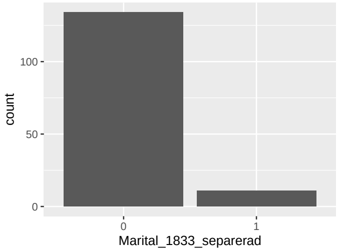

- Observed factor levels: "0", "1".

Marital_1833_singel

Feature	Result
Variable type	factor
Number of missing obs.	0 (0 %)
Number of unique values	2
Mode	"0"
Reference category	0

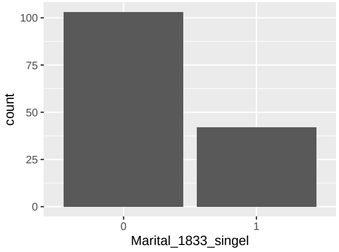

- Observed factor levels: "0", "1".

Edu_1843_2

Feature	Result
Variable type	factor
Number of missing obs.	0 (0 %)
Number of unique values	2
Mode	“0”
Reference category	0

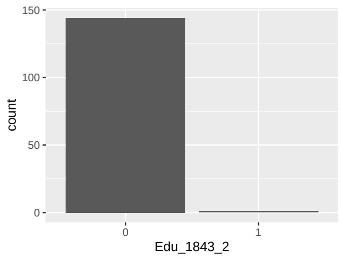

- Observed factor levels: "0", "1".

Edu_1843_3

Feature	Result
Variable type	factor
Number of missing obs.	0 (0 %)
Number of unique values	2
Mode	“0”
Reference category	0

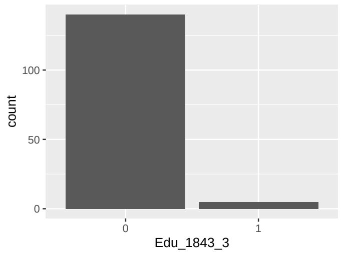

- Observed factor levels: "0", "1".

Edu_1843_4

Feature	Result
Variable type	factor
Number of missing obs.	0 (0 %)
Number of unique values	2
Mode	“0”
Reference category	0

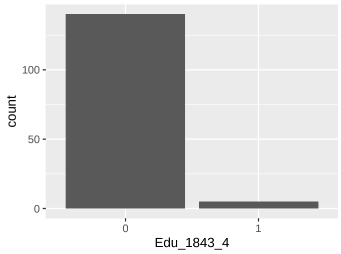

- Observed factor levels: "0", "1".

Edu_1843_5

Feature	Result
Variable type	factor
Number of missing obs.	0 (0 %)
Number of unique values	2
Mode	“0”
Reference category	0

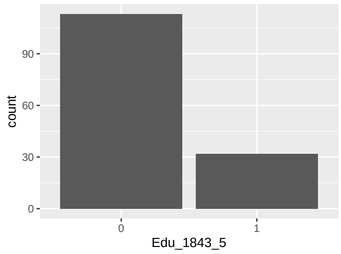

- Observed factor levels: "0", "1".

Edu_1843_6

Feature	Result
Variable type	factor
Number of missing obs.	0 (0 %)
Number of unique values	2
Mode	"0"
Reference category	0

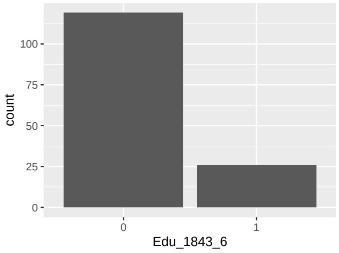

- Observed factor levels: "0", "1".

Edu_1843_7

Feature	Result
Variable type	factor
Number of missing obs.	0 (0 %)
Number of unique values	2
Mode	"1"
Reference category	0

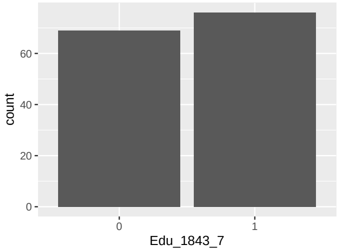

- Observed factor levels: "0", "1".

---

cscale

Feature	Result
Variable type	numeric
Number of missing obs.	0 (0 %)
Number of unique values	36
Median	0.01
1st and 3rd quartiles	-0.7; 0.6
Min. and max.	-3.55; 1.67

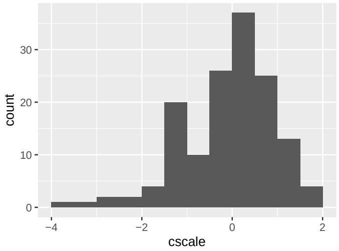

---

mainsymptom_PRE_sum

Feature	Result
Variable type	numeric
Number of missing obs.	0 (0 %)
Number of unique values	29
Median	0.52
1st and 3rd quartiles	-0.1; 1.14
Min. and max.	-1.33; 2.25

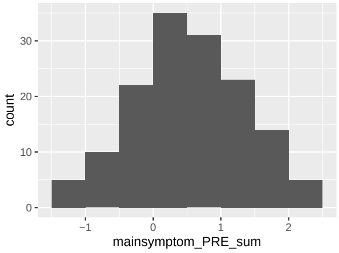

---

mainsymptom_PRE_duration

Feature	Result
Variable type	numeric
Number of missing obs.	0 (0 %)
Number of unique values	134
Median	-0.08
1st and 3rd quartiles	-0.14; 0.08
Min. and max.	-0.25; 2.84

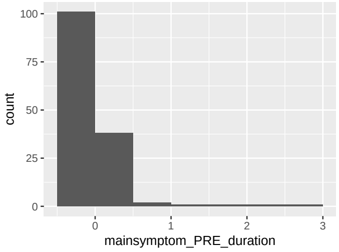

mainsymptom_PRE_DateCompleted_day

Feature	Result
Variable type	numeric
Number of missing obs.	0 (0 %)
Number of unique values	7
Median	1
1st and 3rd quartiles	-1; 1
Min. and max.	-1.37; 1.37

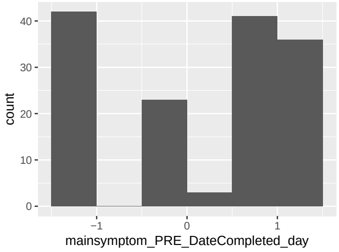

mainsymptom_PRE_DateCompleted_time

Feature	Result
Variable type	numeric
Number of missing obs.	0 (0 %)
Number of unique values	131
Median	-0.67
1st and 3rd quartiles	-1.23; 0.14
Min. and max.	-1.41; 1.12

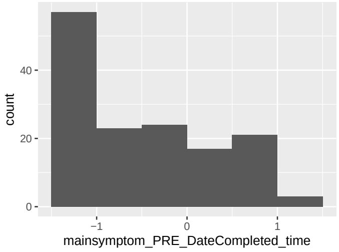

mainsymptom_WEEK01_sum

Feature	Result
Variable type	numeric
Number of missing obs.	0 (0 %)
Number of unique values	34
Median	0.43
1st and 3rd quartiles	-0.18; 1.04
Min. and max.	-1.64; 2.63

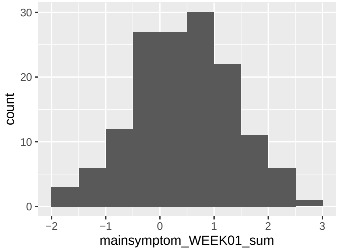

mainsymptom_WEEK01_duration

Feature	Result
Variable type	numeric
Number of missing obs.	0 (0 %)
Number of unique values	110
Median	-0.07
1st and 3rd quartiles	-0.12; -0.01
Min. and max.	-0.17; 15.74

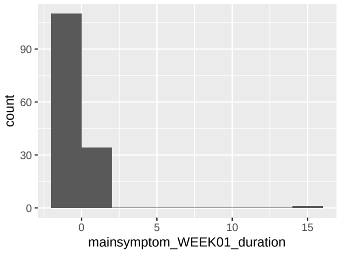

mainsymptom_WEEK01_DateCompleted_day

Feature	Result
Variable type	numeric
Number of missing obs.	0 (0 %)
Number of unique values	7
Median	0.37
1st and 3rd quartiles	-0.37; 1
Min. and max.	-1.37; 1.37

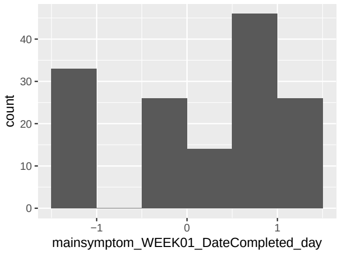

mainsymptom_WEEK01_DateCompleted_time

Feature	Result
Variable type	numeric
Number of missing obs.	0 (0 %)
Number of unique values	136
Median	-0.69
1st and 3rd quartiles	-1.12; 0.2
Min. and max.	-1.41; 1.41

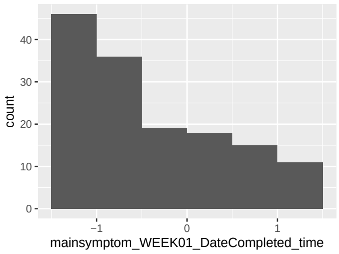

mainsymptom_WEEK02_sum

Feature	Result
Variable type	numeric
Number of missing obs.	0 (0 %)
Number of unique values	30
Median	0.26
1st and 3rd quartiles	-0.34; 0.98
Min. and max.	-1.42; 2.65

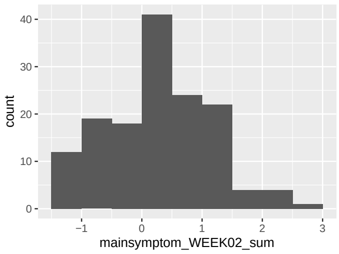

mainsymptom_WEEK02_duration

Feature	Result
Variable type	numeric
Number of missing obs.	0 (0 %)
Number of unique values	103
Median	-0.08
1st and 3rd quartiles	-0.1; -0.01
Min. and max.	-0.16; 12.97

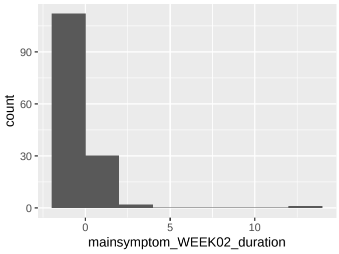

mainsymptom_WEEK02_DateCompleted_day

Feature	Result
Variable type	numeric
Number of missing obs.	0 (0 %)
Number of unique values	7
Median	0.37
1st and 3rd quartiles	-0.37; 1
Min. and max.	-1.37; 1.37

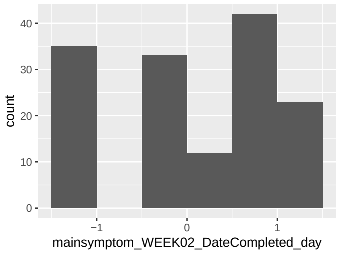

mainsymptom_WEEK02_DateCompleted_time

Feature	Result
Variable type	numeric
Number of missing obs.	0 (0 %)
Number of unique values	136
Median	-0.44
1st and 3rd quartiles	-0.96; 0.34
Min. and max.	-1.41; 1.41

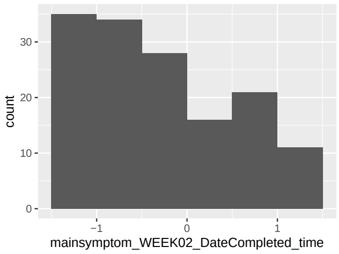

mainsymptom_WEEK03_sum

Feature	Result
Variable type	numeric
Number of missing obs.	0 (0 %)
Number of unique values	32
Median	0.35
1st and 3rd quartiles	-0.24; 0.83
Min. and max.	-1.44; 2.37

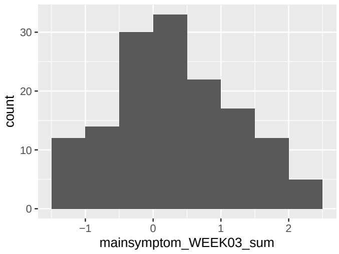

mainsymptom_WEEK03_duration

Feature	Result
Variable type	numeric
Number of missing obs.	0 (0 %)
Number of unique values	105
Median	-0.07
1st and 3rd quartiles	-0.1; -0.01
Min. and max.	-0.15; 5.21

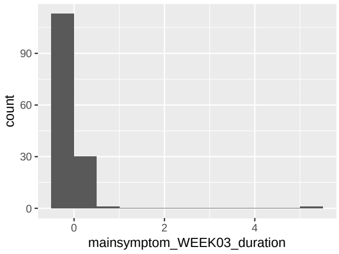

---

mainsymptom_WEEK03_DateCompleted_day

Feature	Result
Variable type	numeric
Number of missing obs.	0 (0 %)
Number of unique values	7
Median	1
1st and 3rd quartiles	-0.37; 1
Min. and max.	-1.37; 1.37

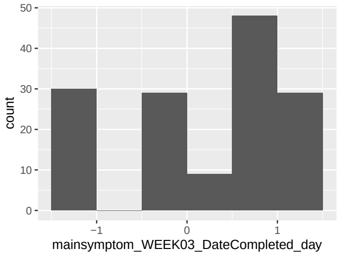

---

mainsymptom_WEEK03_DateCompleted_time

Feature	Result
Variable type	numeric
Number of missing obs.	0 (0 %)
Number of unique values	139
Median	-0.65
1st and 3rd quartiles	-1.18; -0.06
Min. and max.	-1.41; 1.41

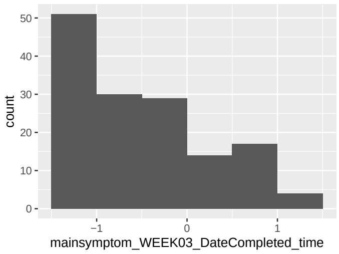

Report generation information:

- Created by: Could not determine from system (username: `nilisa`).
- Report creation time: Mon Jan 09 2023 13:13:10
- Report was run from directory: `/home/nilisa/projects/phd_study1/r`
- dataMaid v1.4.1 [Pkg: 2021-10-08 from CRAN (R 4.2.2)]
- R version 4.2.2 Patched (2022-11-10 r83330).
- Platform: x86_64-pc-linux-gnu (64-bit)(Ubuntu 20.04.5 LTS).
- Function call: `dataMaid::makeDataReport(data = gd, mode = c("summarize", "visualize", "check"), smartNum = FALSE, file = "~/projects/data/study1multiverse/results/graphs_n_figures/codebooks/codebook replace = TRUE, openResult = FALSE, checks = list(character = "showAllFactorLevels", factor = "showAllFactorLevels", labelled = "showAllFactorLevels",   haven_labelled = "showAllFactorLevels", numeric = NULL,   integer = NULL, logical = NULL, Date = NULL), listChecks = FALSE, maxProbVals = Inf, codebook = TRUE, reportTitle = "Handpicked_Depression_week04-r`

Handpicked_Depression_week04-naremove_train

Autogenerated data summary from dataMaid

2023-01-09 13:06:45

Data report overview

The dataset examined has the following dimensions:

Feature	Result
Number of observations	1437
Number of variables	60

Codebook summary table

Label	Variable	Class	# unique values	Missing	Description
	**sex**	factor	2	0.00 %	Sex of patient, 0 = Female, 1=Male
	**age**	numeric	61	0.00 %	
	**messages_len_7**	numeric	363	0.00 %	-Meta information of messages-Length of messages-up until day-7
	**messages_len_tp_7**	numeric	691	0.00 %	-Meta information of messages-Length of messages-therapist messages-up until day-7
	**messages_7**	numeric	8	0.00 %	-Meta information of messages-up until day-7
	**messages_tp_7**	numeric	8	0.00 %	-Meta information of messages-therapist messages-up until day-7
	**homeworks_7**	numeric	5	0.00 %	-Number of homework messages sent in-up until day-7
	**messages_len_14**	numeric	422	0.00 %	-Meta information of messages-Length of messages-up until day-14
	**messages_len_tp_14**	numeric	760	0.00 %	-Meta information of messages-Length of messages-therapist messages-up until day-14
	**messages_14**	numeric	9	0.00 %	-Meta information of messages-up until day-14
	**messages_tp_14**	numeric	6	0.00 %	-Meta information of messages-therapist messages-up until day-14

Label	Variable	Class	# unique values	Missing	Description
	**homeworks_14**	numeric	6	0.00 %	-Number of homework messages sent in-up until day-14
	**messages_len_21**	numeric	424	0.00 %	-Meta information of messages-Length of messages-up until day-21
	**messages_len_tp_21**	numeric	765	0.00 %	-Meta information of messages-Length of messages-therapist messages-up until day-21
	**messages_21**	numeric	9	0.00 %	-Meta information of messages-up until day-21
	**messages_tp_21**	numeric	7	0.00 %	-Meta information of messages-therapist messages-up until day-21
	**homeworks_21**	numeric	6	0.00 %	-Number of homework messages sent in-up until day-21
	**messages_len_28**	numeric	365	0.00 %	-Meta information of messages-Length of messages-up until day-28
	**messages_len_tp_28**	numeric	756	0.00 %	-Meta information of messages-Length of messages-therapist messages-up until day-28
	**messages_28**	numeric	7	0.00 %	-Meta information of messages-up until day-28
	**messages_tp_28**	numeric	7	0.00 %	-Meta information of messages-therapist messages-up until day-28
	**homeworks_28**	numeric	6	0.00 %	-Number of homework messages sent in-up until day-28
	**PDSS-SR-3064_SCREEN_sum**	numeric	25	0.00 %	Anxiety questionnaire, self rated-Timepoint before treatment starts-Sum of the entire measure
	**MADRS-1951_SCREEN_sum**	numeric	39	0.00 %	Depression questionnaire, self rated-Timepoint before treatment starts-Sum of the entire measure
	**LSAS-2241_SCREEN_sum**	numeric	123	0.00 %	Social anxiety questionnaire, self rated-Timepoint before treatment starts-Sum of the entire measure
	**MADRS-1951_SCREEN_DateCompleted_day**	numeric	7	0.00 %	Depression questionnaire, self rated-Timepoint before treatment starts-Cyclic transformation of what day 0-6 during week it was filled in

Label	Variable	Class	# unique values	Missing	Description
	**MADRS-1951_SCREEN_DateCompleted_time**	numeric	702	0.00 %	Depression questionnaire, self rated-Timepoint before treatment starts-Cyclic transformation of what time during day 0-1440 it was filled in
	**PDSS-SR-3064_SCREEN_DateCompleted_day**	numeric	7	0.00 %	Anxiety questionnaire, self rated-Timepoint before treatment starts-Cyclic transformation of what day 0-6 during week it was filled in
	**PDSS-SR-3064_SCREEN_DateCompleted_time**	numeric	711	0.00 %	Anxiety questionnaire, self rated-Timepoint before treatment starts-Cyclic transformation of what time during day 0-1440 it was filled in
	**LSAS-2241_SCREEN_DateCompleted_day**	numeric	7	0.00 %	Social anxiety questionnaire, self rated-Timepoint before treatment starts-Cyclic transformation of what day 0-6 during week it was filled in
	**LSAS-2241_SCREEN_DateCompleted_time**	numeric	714	0.00 %	Social anxiety questionnaire, self rated-Timepoint before treatment starts-Cyclic transformation of what time during day 0-1440 it was filled in
	**outcome**	numeric	41	0.00 %	
	**ncomorbid**	numeric	5	0.00 %	
	**currentwork_proff**	factor	2	0.00 %	Currently in work for trained proffession
	**Marital_1833_gift**	factor	2	0.00 %	Marital status: Married or not
	**Marital_1833_separerad**	factor	2	0.00 %	Marital status: divocered/equivalent
	**Marital_1833_singel**	factor	2	0.00 %	Marital status: single
	**Edu_1843_2**	factor	2	0.00 %	7-9 years education
	**Edu_1843_3**	factor	2	0.00 %	Uncompleted upper secondary school
	**Edu_1843_4**	factor	2	0.00 %	Higher vocational education
	**Edu_1843_5**	factor	2	0.00 %	Completed upper secondary school
	**Edu_1843_6**	factor	2	0.00 %	Uncompleted university degree
	**Edu_1843_7**	factor	2	0.00 %	University degree
	**cscale**	numeric	44	0.00 %	
	**mainsymptom_PRE_sum**	numeric	41	0.00 %	PDSS-SR for panic, MADRS for depression, LSAS for social anxiety-Timepoint just before beginning treatment-Sum of the entire measure

Label	Variable	Class	# unique values	Missing	Description
	**mainsymptom_PRE_duration**	numeric	676	0.00 %	PDSS-SR for panic, MADRS for depression, LSAS for social anxiety-Timepoint just before beginning treatment-Time to fill in measure/questionnaire
	**mainsymptom_PRE_DateCompleted_day**	numeric	7	0.00 %	PDSS-SR for panic, MADRS for depression, LSAS for social anxiety-Timepoint just before beginning treatment-Cyclic transformation of what day 0-6 during week it was filled in
	**mainsymptom_PRE_DateCompleted_time**	numeric	681	0.00 %	PDSS-SR for panic, MADRS for depression, LSAS for social anxiety-Timepoint just before beginning treatment-Cyclic transformation of what time during day 0-1440 it was filled in
	**mainsymptom_WEEK01_sum**	numeric	42	0.00 %	PDSS-SR for panic, MADRS for depression, LSAS for social anxiety-Timepoint after one week in treatment-Sum of the entire measure
	**mainsymptom_WEEK01_duration**	numeric	425	0.00 %	PDSS-SR for panic, MADRS for depression, LSAS for social anxiety-Timepoint after one week in treatment-Time to fill in measure/questionnaire
	**mainsymptom_WEEK01_DateCompleted_day**	numeric	7	0.00 %	PDSS-SR for panic, MADRS for depression, LSAS for social anxiety-Timepoint after one week in treatment-Cyclic transformation of what day 0-6 during week it was filled in
	**mainsymptom_WEEK01_DateCompleted_time**	numeric	725	0.00 %	PDSS-SR for panic, MADRS for depression, LSAS for social anxiety-Timepoint after one week in treatment-Cyclic transformation of what time during day 0-1440 it was filled in
	**mainsymptom_WEEK02_sum**	numeric	41	0.00 %	PDSS-SR for panic, MADRS for depression, LSAS for social anxiety-Timepoint after two weeks in treatment-Sum of the entire measure
	**mainsymptom_WEEK02_duration**	numeric	378	0.00 %	PDSS-SR for panic, MADRS for depression, LSAS for social anxiety-Timepoint after two weeks in treatment-Time to fill in measure/questionnaire

Label	Variable	Class	# unique values	Missing	Description
	**mainsymptom_WEEK02_DateCompleted_day**	numeric	7	0.00 %	PDSS-SR for panic, MADRS for depression, LSAS for social anxiety-Timepoint after two weeks in treatment-Cyclic transformation of what day 0-6 during week it was filled in
	**mainsymptom_WEEK02_DateCompleted_time**	numeric	730	0.00 %	PDSS-SR for panic, MADRS for depression, LSAS for social anxiety-Timepoint after two weeks in treatment-Cyclic transformation of what time during day 0-1440 it was filled in
	**mainsymptom_WEEK03_sum**	numeric	41	0.00 %	PDSS-SR for panic, MADRS for depression, LSAS for social anxiety-Timepoint after three weeks in treatment-Sum of the entire measure
	**mainsymptom_WEEK03_duration**	numeric	369	0.00 %	PDSS-SR for panic, MADRS for depression, LSAS for social anxiety-Timepoint after three weeks in treatment-Time to fill in measure/questionnaire
	**mainsymptom_WEEK03_DateCompleted_day**	numeric	7	0.00 %	PDSS-SR for panic, MADRS for depression, LSAS for social anxiety-Timepoint after three weeks in treatment-Cyclic transformation of what day 0-6 during week it was filled in
	**mainsymptom_WEEK03_DateCompleted_time**	numeric	727	0.00 %	PDSS-SR for panic, MADRS for depression, LSAS for social anxiety-Timepoint after three weeks in treatment-Cyclic transformation of what time during day 0-1440 it was filled in

Variable list

sex

Feature	Result
Variable type	factor
Number of missing obs.	0 (0 %)
Number of unique values	2
Mode	“0”
Reference category	0

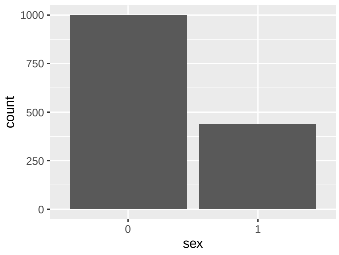

- Observed factor levels: "0", "1".

---

age

Feature	Result
Variable type	numeric
Number of missing obs.	0 (0 %)
Number of unique values	61
Median	0.06
1st and 3rd quartiles	-0.55; 1.02
Min. and max.	-1.51; 4.16

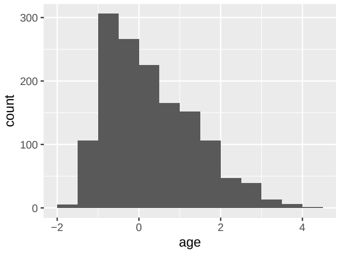

messages_len_7

Feature	Result
Variable type	numeric
Number of missing obs.	0 (0 %)
Number of unique values	363
Median	-0.32
1st and 3rd quartiles	-0.32; 0.14
Min. and max.	-0.32; 35.05

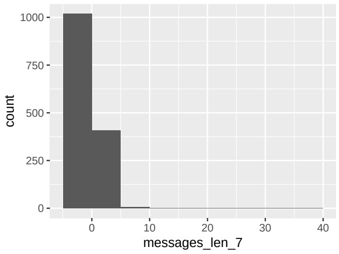

messages_len_tp_7

Feature	Result
Variable type	numeric
Number of missing obs.	0 (0 %)
Number of unique values	691
Median	0.26
1st and 3rd quartiles	-0.44; 0.93
Min. and max.	-1.46; 9.81

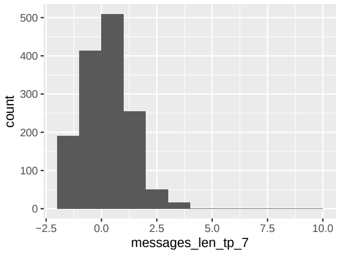

messages_7

Feature	Result
Variable type	numeric
Number of missing obs.	0 (0 %)
Number of unique values	8
Median	-0.55
1st and 3rd quartiles	-0.55; 0.58
Min. and max.	-0.55; 10.81

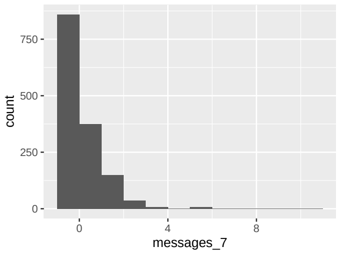

messages_tp_7

Feature	Result
Variable type	numeric
Number of missing obs.	0 (0 %)
Number of unique values	8
Median	0.15
1st and 3rd quartiles	0.15; 1.21
Min. and max.	-1.97; 6.52

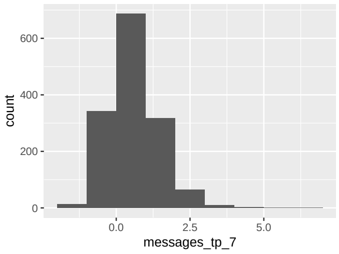

homeworks_7

Feature	Result
Variable type	numeric
Number of missing obs.	0 (0 %)
Number of unique values	5
Median	0.2
1st and 3rd quartiles	0.2; 0.2
Min. and max.	-1.03; 3.91

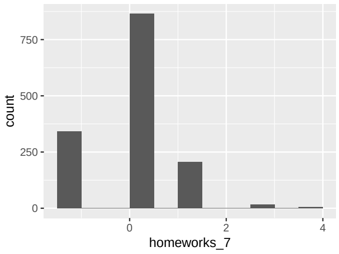

messages_len_14

Feature	Result
Variable type	numeric
Number of missing obs.	0 (0 %)
Number of unique values	422
Median	-0.29
1st and 3rd quartiles	-0.41; 0.27
Min. and max.	-0.41; 11.44

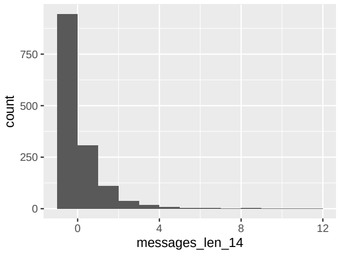

messages_len_tp_14

Feature	Result
Variable type	numeric
Number of missing obs.	0 (0 %)
Number of unique values	760
Median	0.16
1st and 3rd quartiles	-0.52; 0.82
Min. and max.	-1.11; 7.22

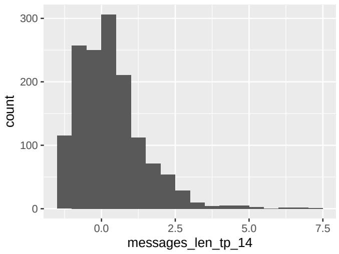

messages_14

Feature	Result
Variable type	numeric
Number of missing obs.	0 (0 %)
Number of unique values	9
Median	0.36
1st and 3rd quartiles	-0.7; 0.36
Min. and max.	-0.7; 7.78

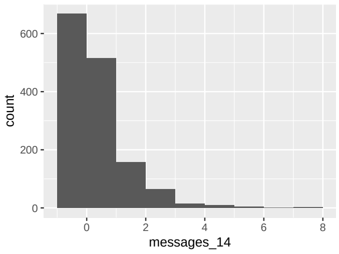

---

messages_tp_14

Feature	Result
Variable type	numeric
Number of missing obs.	0 (0 %)
Number of unique values	6
Median	-0.35
1st and 3rd quartiles	-0.35; 0.87
Min. and max.	-1.57; 4.53

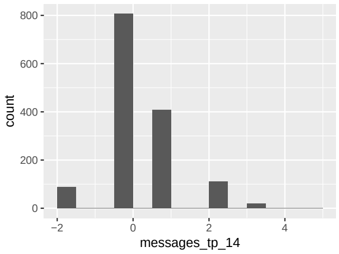

---

homeworks_14

Feature	Result
Variable type	numeric
Number of missing obs.	0 (0 %)
Number of unique values	6
Median	0.33
1st and 3rd quartiles	-0.98; 0.33
Min. and max.	-0.98; 5.58

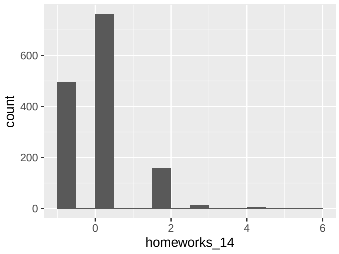

---

messages_len_21

Feature	Result
Variable type	numeric
Number of missing obs.	0 (0 %)
Number of unique values	424
Median	-0.26
1st and 3rd quartiles	-0.46; 0.31
Min. and max.	-0.46; 9.83

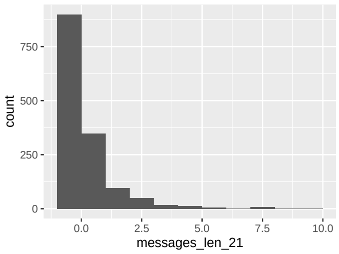

---

messages_len_tp_21

Feature	Result
Variable type	numeric
Number of missing obs.	0 (0 %)
Number of unique values	765
Median	0.18
1st and 3rd quartiles	-0.45; 0.94
Min. and max.	-1.01; 6.14

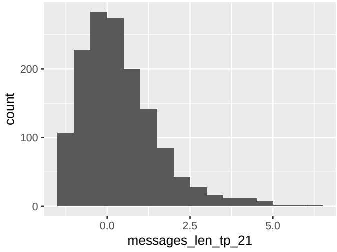

messages_21

Feature	Result
Variable type	numeric
Number of missing obs.	0 (0 %)
Number of unique values	9
Median	0.34
1st and 3rd quartiles	-0.74; 0.34
Min. and max.	-0.74; 8.99

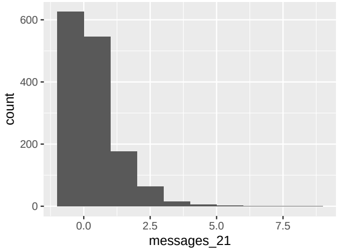

messages_tp_21

Feature	Result
Variable type	numeric
Number of missing obs.	0 (0 %)
Number of unique values	7
Median	-0.32
1st and 3rd quartiles	-0.32; 0.84
Min. and max.	-1.49; 5.5

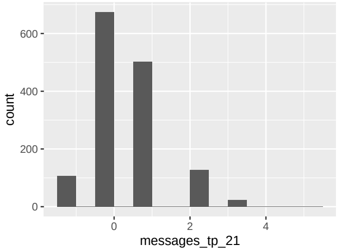

homeworks_21

Feature	Result
Variable type	numeric
Number of missing obs.	0 (0 %)
Number of unique values	6
Median	0.36
1st and 3rd quartiles	-1.01; 0.36
Min. and max.	-1.01; 5.86

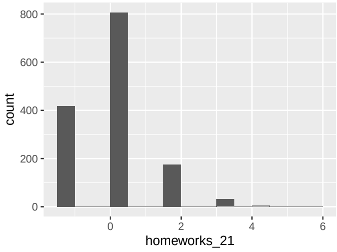

messages_len_28

Feature	Result
Variable type	numeric
Number of missing obs.	0 (0 %)
Number of unique values	365
Median	-0.36
1st and 3rd quartiles	-0.41; 0.21
Min. and max.	-0.41; 13.67

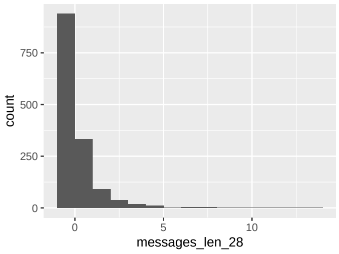

messages_len_tp_28

Feature	Result
Variable type	numeric
Number of missing obs.	0 (0 %)
Number of unique values	756
Median	0.17
1st and 3rd quartiles	-0.43; 0.91
Min. and max.	-0.94; 10.31

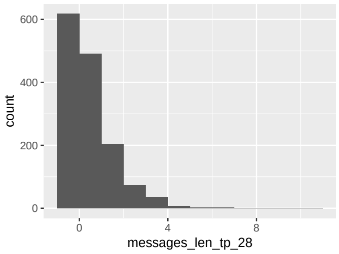

messages_28

Feature	Result
Variable type	numeric
Number of missing obs.	0 (0 %)
Number of unique values	7
Median	0.41
1st and 3rd quartiles	-0.72; 0.41
Min. and max.	-0.72; 6.07

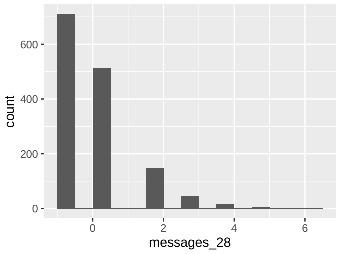

messages_tp_28

Feature	Result
Variable type	numeric
Number of missing obs.	0 (0 %)
Number of unique values	7
Median	-0.24
1st and 3rd quartiles	-0.24; 0.93
Min. and max.	-1.42; 5.63

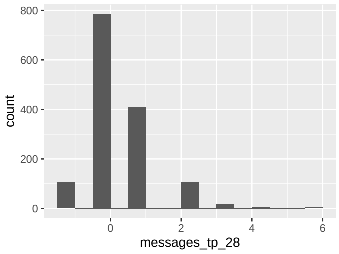

homeworks_28

Feature	Result
Variable type	numeric
Number of missing obs.	0 (0 %)
Number of unique values	6
Median	0.46
1st and 3rd quartiles	-0.91; 0.46
Min. and max.	-0.91; 5.94

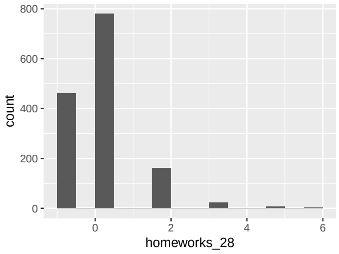

PDSS-SR-3064_SCREEN_sum

Feature	Result
Variable type	numeric
Number of missing obs.	0 (0 %)
Number of unique values	25
Median	-0.51
1st and 3rd quartiles	-1.29; 0.28
Min. and max.	-1.29; 2.65

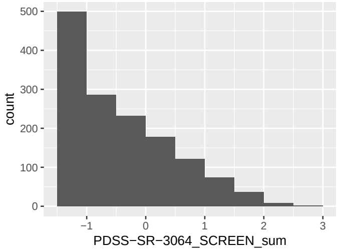

MADRS-1951_SCREEN_sum

Feature	Result
Variable type	numeric
Number of missing obs.	0 (0 %)
Number of unique values	39
Median	0.54
1st and 3rd quartiles	0.05; 0.92
Min. and max.	-1.94; 3.02

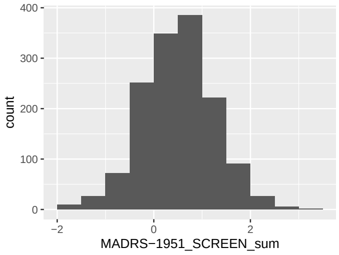

LSAS-2241_SCREEN_sum

Feature	Result
Variable type	numeric
Number of missing obs.	0 (0 %)
Number of unique values	123
Median	-0.3
1st and 3rd quartiles	-0.92; 0.36
Min. and max.	-1.79; 2.85

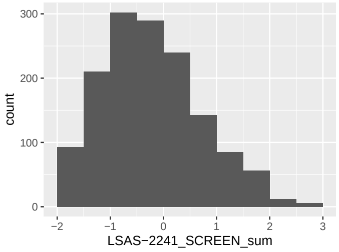

MADRS-1951_SCREEN_DateCompleted_day

Feature	Result
Variable type	numeric
Number of missing obs.	0 (0 %)
Number of unique values	7
Median	0.37
1st and 3rd quartiles	-1; 1
Min. and max.	-1.37; 1.37

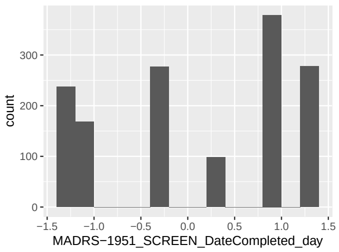

MADRS-1951_SCREEN_DateCompleted_time

Feature	Result
Variable type	numeric
Number of missing obs.	0 (0 %)
Number of unique values	702
Median	-0.77
1st and 3rd quartiles	-1.25; 0.21
Min. and max.	-1.41; 1.41

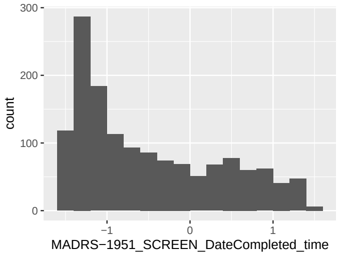

PDSS-SR-3064_SCREEN_DateCompleted_day

Feature	Result
Variable type	numeric
Number of missing obs.	0 (0 %)
Number of unique values	7
Median	0.37
1st and 3rd quartiles	-1; 1
Min. and max.	-1.37; 1.37

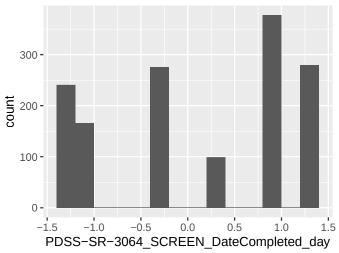

PDSS-SR-3064_SCREEN_DateCompleted_time

Feature	Result
Variable type	numeric
Number of missing obs.	0 (0 %)
Number of unique values	711
Median	-0.76
1st and 3rd quartiles	-1.25; 0.23
Min. and max.	-1.41; 1.41

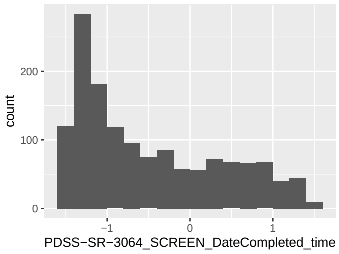

LSAS-2241_SCREEN_DateCompleted_day

Feature	Result
Variable type	numeric
Number of missing obs.	0 (0 %)
Number of unique values	7
Median	0.37
1st and 3rd quartiles	-1; 1
Min. and max.	-1.37; 1.37

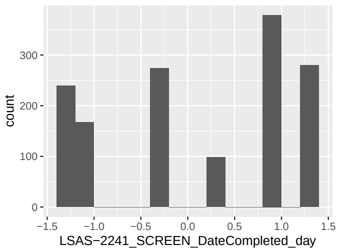

LSAS-2241_SCREEN_DateCompleted_time

Feature	Result
Variable type	numeric
Number of missing obs.	0 (0 %)
Number of unique values	714
Median	-0.77
1st and 3rd quartiles	-1.24; 0.29
Min. and max.	-1.41; 1.41

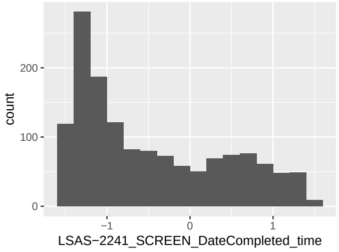

outcome

Feature	Result
Variable type	numeric
Number of missing obs.	0 (0 %)
Number of unique values	41
Median	-0.18
1st and 3rd quartiles	-0.77; 0.53
Min. and max.	-1.59; 3.12

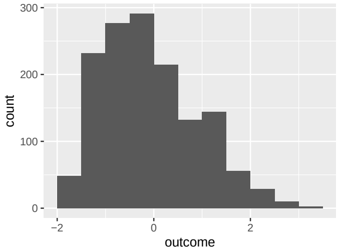

ncomorbid

Feature	Result
Variable type	numeric
Number of missing obs.	0 (0 %)
Number of unique values	5
Median	0
1st and 3rd quartiles	0; 1
Min. and max.	0; 4

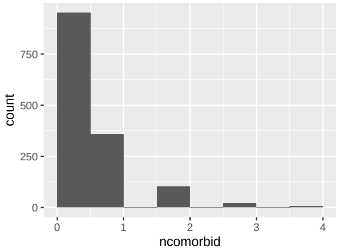

currentwork_proff

Feature	Result
Variable type	factor
Number of missing obs.	0 (0 %)
Number of unique values	2
Mode	"1"
Reference category	0

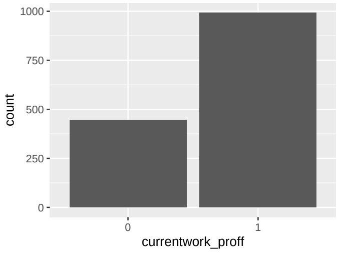

- Observed factor levels: "0", "1".

Marital_1833_gift

Feature	Result
Variable type	factor
Number of missing obs.	0 (0 %)
Number of unique values	2
Mode	"1"
Reference category	0

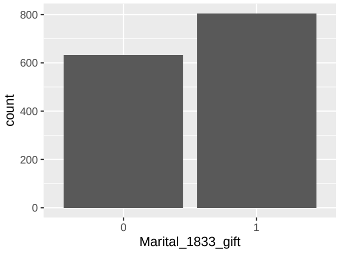

- Observed factor levels: "0", "1".

Marital_1833_separerad

Feature	Result
Variable type	factor
Number of missing obs.	0 (0 %)
Number of unique values	2
Mode	"0"
Reference category	0

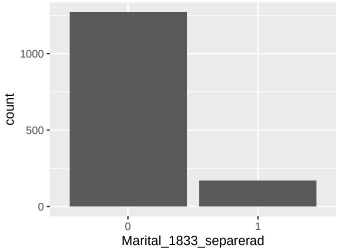

- Observed factor levels: "0", "1".

Marital_1833_singel

Feature	Result
Variable type	factor
Number of missing obs.	0 (0 %)
Number of unique values	2
Mode	"0"
Reference category	0

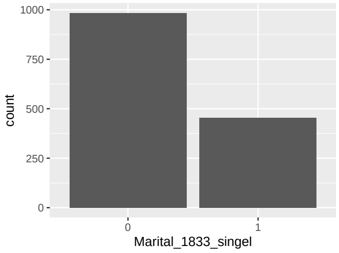

- Observed factor levels: "0", "1".

Edu_1843_2

Feature	Result
Variable type	factor
Number of missing obs.	0 (0 %)
Number of unique values	2
Mode	“0”
Reference category	0

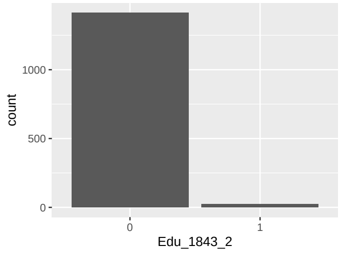

- Observed factor levels: "0", "1".

Edu_1843_3

Feature	Result
Variable type	factor
Number of missing obs.	0 (0 %)
Number of unique values	2
Mode	“0”
Reference category	0

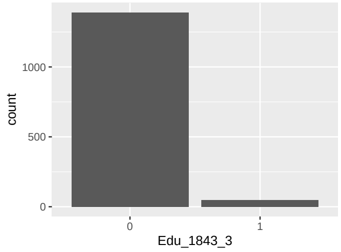

- Observed factor levels: "0", "1".

Edu_1843_4

Feature	Result
Variable type	factor
Number of missing obs.	0 (0 %)
Number of unique values	2
Mode	“0”
Reference category	0

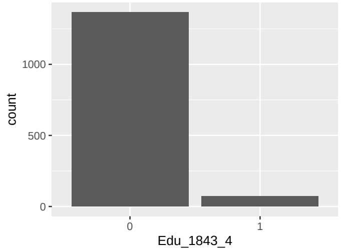

- Observed factor levels: "0", "1".

Edu_1843_5

Feature	Result
Variable type	factor
Number of missing obs.	0 (0 %)
Number of unique values	2
Mode	“0”
Reference category	0

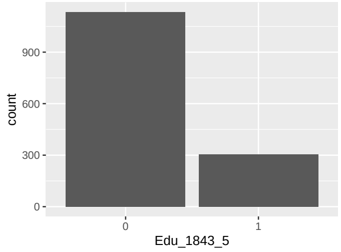

- Observed factor levels: "0", "1".

Edu_1843_6

Feature	Result
Variable type	factor
Number of missing obs.	0 (0 %)
Number of unique values	2
Mode	“0”
Reference category	0

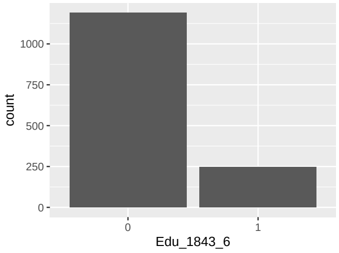

- Observed factor levels: "0", "1".

Edu_1843_7

Feature	Result
Variable type	factor
Number of missing obs.	0 (0 %)
Number of unique values	2
Mode	“1”
Reference category	0

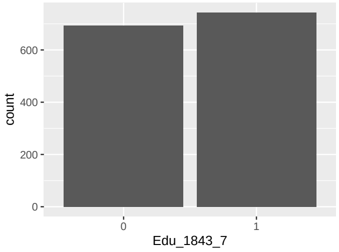

- Observed factor levels: "0", "1".

---

cscale

Feature	Result
Variable type	numeric
Number of missing obs.	0 (0 %)
Number of unique values	44
Median	0.01
1st and 3rd quartiles	-0.7; 0.6
Min. and max.	-3.43; 1.79

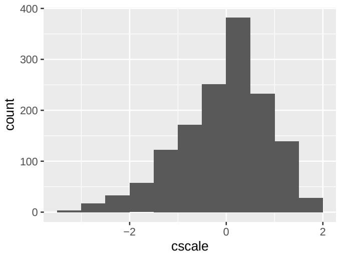

---

mainsymptom_PRE_sum

Feature	Result
Variable type	numeric
Number of missing obs.	0 (0 %)
Number of unique values	41
Median	0.52
1st and 3rd quartiles	0.03; 1.01
Min. and max.	-2.32; 2.99

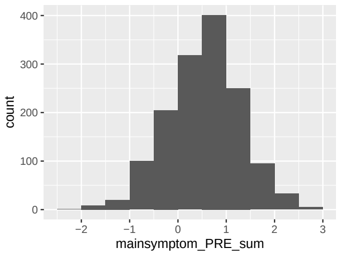

---

mainsymptom_PRE_duration

Feature	Result
Variable type	numeric
Number of missing obs.	0 (0 %)
Number of unique values	676
Median	-0.09
1st and 3rd quartiles	-0.17; 0.03
Min. and max.	-0.28; 24.6

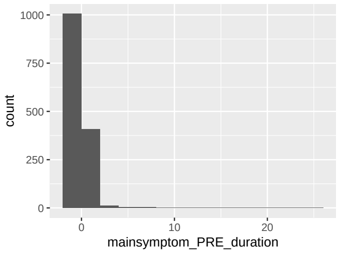

mainsymptom_PRE_DateCompleted_day

Feature	Result
Variable type	numeric
Number of missing obs.	0 (0 %)
Number of unique values	7
Median	1
1st and 3rd quartiles	-0.37; 1
Min. and max.	-1.37; 1.37

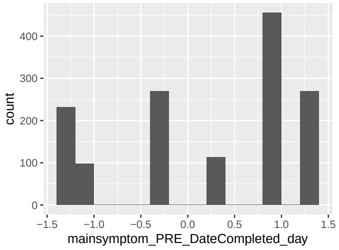

mainsymptom_PRE_DateCompleted_time

Feature	Result
Variable type	numeric
Number of missing obs.	0 (0 %)
Number of unique values	681
Median	-0.69
1st and 3rd quartiles	-1.24; 0.07
Min. and max.	-1.41; 1.4

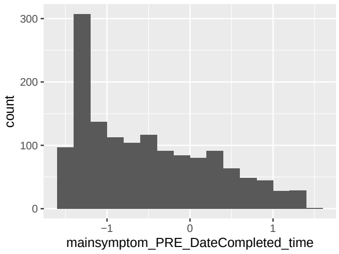

mainsymptom_WEEK01_sum

Feature	Result
Variable type	numeric
Number of missing obs.	0 (0 %)
Number of unique values	42
Median	0.43
1st and 3rd quartiles	-0.05; 1.04
Min. and max.	-2.01; 3.6

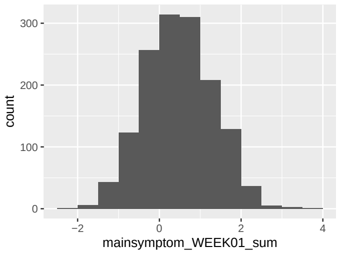

mainsymptom_WEEK01_duration

Feature	Result
Variable type	numeric
Number of missing obs.	0 (0 %)
Number of unique values	425
Median	-0.07
1st and 3rd quartiles	-0.12; 0.01
Min. and max.	-0.2; 35.17

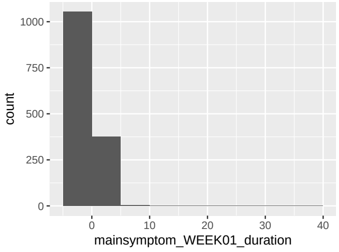

mainsymptom_WEEK01_DateCompleted_day

Feature	Result
Variable type	numeric
Number of missing obs.	0 (0 %)
Number of unique values	7
Median	1
1st and 3rd quartiles	-0.37; 1
Min. and max.	-1.37; 1.37

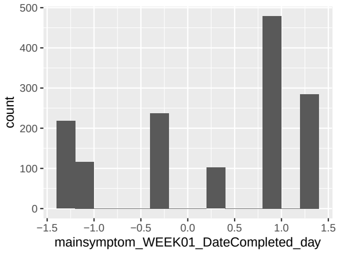

mainsymptom_WEEK01_DateCompleted_time

Feature	Result
Variable type	numeric
Number of missing obs.	0 (0 %)
Number of unique values	725
Median	-0.67
1st and 3rd quartiles	-1.21; 0.12
Min. and max.	-1.41; 1.41

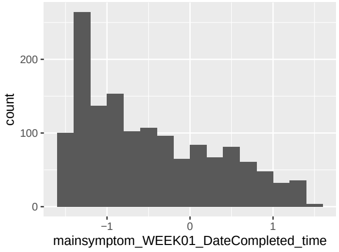

mainsymptom_WEEK02_sum

Feature	Result
Variable type	numeric
Number of missing obs.	0 (0 %)
Number of unique values	41
Median	0.38
1st and 3rd quartiles	-0.22; 0.98
Min. and max.	-1.9; 3.13

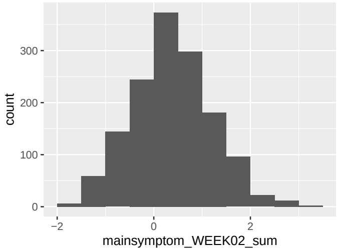

mainsymptom_WEEK02_duration

Feature	Result
Variable type	numeric
Number of missing obs.	0 (0 %)
Number of unique values	378
Median	-0.07
1st and 3rd quartiles	-0.11; 0
Min. and max.	-0.17; 23.95

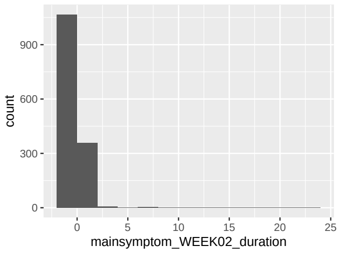

mainsymptom_WEEK02_DateCompleted_day

Feature	Result
Variable type	numeric
Number of missing obs.	0 (0 %)
Number of unique values	7
Median	1
1st and 3rd quartiles	-0.37; 1
Min. and max.	-1.37; 1.37

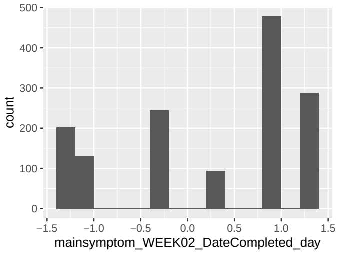

mainsymptom_WEEK02_DateCompleted_time

Feature	Result
Variable type	numeric
Number of missing obs.	0 (0 %)
Number of unique values	730
Median	-0.74
1st and 3rd quartiles	-1.22; 0.05
Min. and max.	-1.41; 1.41

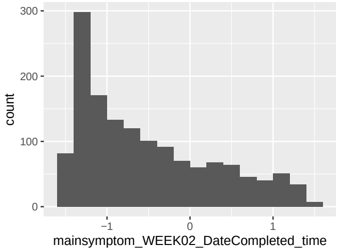

mainsymptom_WEEK03_sum

Feature	Result
Variable type	numeric
Number of missing obs.	0 (0 %)
Number of unique values	41
Median	0.35
1st and 3rd quartiles	-0.24; 0.95
Min. and max.	-1.79; 2.97

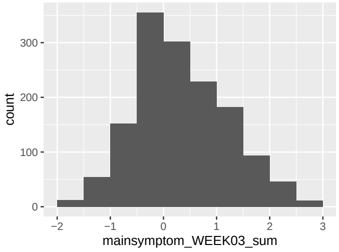

mainsymptom_WEEK03_duration

Feature	Result
Variable type	numeric
Number of missing obs.	0 (0 %)
Number of unique values	369
Median	-0.07
1st and 3rd quartiles	-0.1; 0
Min. and max.	-0.18; 41.39

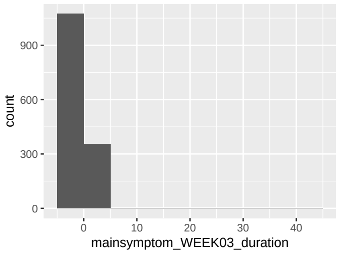

mainsymptom_WEEK03_DateCompleted_day

Feature	Result
Variable type	numeric
Number of missing obs.	0 (0 %)
Number of unique values	7
Median	1
1st and 3rd quartiles	-0.37; 1
Min. and max.	-1.37; 1.37

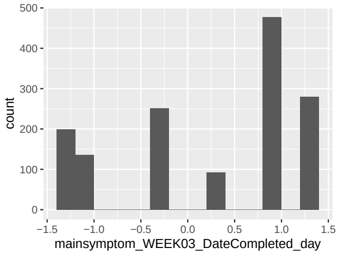

mainsymptom_WEEK03_DateCompleted_time

Feature	Result
Variable type	numeric
Number of missing obs.	0 (0 %)
Number of unique values	727
Median	-0.64
1st and 3rd quartiles	-1.21; 0.15
Min. and max.	-1.41; 1.41

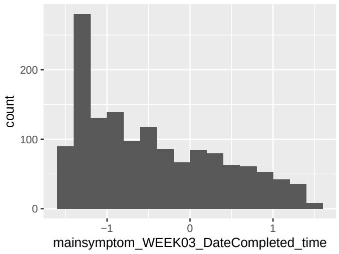

Report generation information:

- Created by: Could not determine from system (username: `nilisa`).
- Report creation time: Mon Jan 09 2023 13:06:46
- Report was run from directory: `/home/nilisa/projects/phd_study1/r`
- dataMaid v1.4.1 [Pkg: 2021-10-08 from CRAN (R 4.2.2)]
- R version 4.2.2 Patched (2022-11-10 r83330).
- Platform: x86_64-pc-linux-gnu (64-bit)(Ubuntu 20.04.5 LTS).
- Function call: `dataMaid::makeDataReport(data = gd, mode = c("summarize", "visualize",  "check"), smartNum = FALSE, file = "~/projects/data/study1multiverse/results/graphs_n_figures/codebooks/codebook` `replace = TRUE, openResult = FALSE, checks = list(character = "showAllFactorLevels", factor = "showAllFactorLevels", labelled = "showAllFactorLevels",   haven_labelled = "showAllFactorLevels", numeric = NULL,   integer = NULL, logical = NULL, Date = NULL), listChecks = FALSE, maxProbVals = Inf, codebook = TRUE, reportTitle = "Handpicked_Depression_week04-r`

Handpicked_Panic_week04-imputed_benchmark_test

Autogenerated data summary from dataMaid

2023-01-09 13:13:48

Data report overview

The dataset examined has the following dimensions:

Feature	Result
Number of observations	177
Number of variables	10

Codebook summary table

Label	Variable	Class	# unique values	Missing	Description
	**sex**	factor	2	0.00 %	Sex of patient, 0 = Female, 1=Male
	**age**	numeric	45	0.00 %	
	**PDSS-SR-3064_SCREEN_sum**	numeric	38	0.00 %	Anxiety questionnaire, self rated-Timepoint before treatment starts-Sum of the entire measure
	**MADRS-1951_SCREEN_sum**	numeric	38	0.00 %	Depression questionnaire, self rated-Timepoint before treatment starts-Sum of the entire measure
	**LSAS-2241_SCREEN_sum**	numeric	94	0.00 %	Social anxiety questionnaire, self rated-Timepoint before treatment starts-Sum of the entire measure
	**outcome**	numeric	61	0.00 %	
	**mainsymptom_PRE_sum**	numeric	33	0.00 %	PDSS-SR for panic, MADRS for depression, LSAS for social anxiety-Timepoint just before beginning treatment-Sum of the entire measure
	**mainsymptom_WEEK01_sum**	numeric	62	0.00 %	PDSS-SR for panic, MADRS for depression, LSAS for social anxiety-Timepoint after one week in treatment-Sum of the entire measure
	**mainsymptom_WEEK02_sum**	numeric	56	0.00 %	PDSS-SR for panic, MADRS for depression, LSAS for social anxiety-Timepoint after two weeks in treatment-Sum of the entire measure

Label	Variable	Class	# unique values	Missing	Description
	**mainsymptom_WEEK03_sum**	numeric	60	0.00 %	PDSS-SR for panic, MADRS for depression, LSAS for social anxiety-Timepoint after three weeks in treatment-Sum of the entire measure

Variable list

sex

Feature	Result
Variable type	factor
Number of missing obs.	0 (0 %)
Number of unique values	2
Mode	“0”
Reference category	0

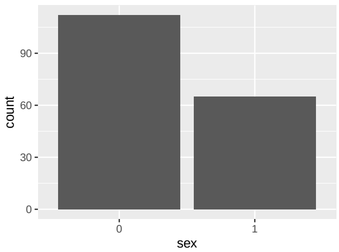

- Observed factor levels: "0", "1".

---

age

Feature	Result
Variable type	numeric
Number of missing obs.	0 (0 %)
Number of unique values	45
Median	-0.46
1st and 3rd quartiles	-0.9; 0.32
Min. and max.	-1.6; 2.77

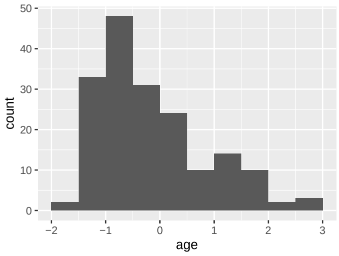

PDSS-SR-3064_SCREEN_sum

Feature	Result
Variable type	numeric
Number of missing obs.	0 (0 %)
Number of unique values	38
Median	0.75
1st and 3rd quartiles	0.17; 1.23
Min. and max.	-1.14; 2.65

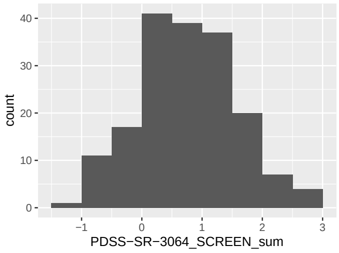

MADRS-1951_SCREEN_sum

Feature	Result
Variable type	numeric
Number of missing obs.	0 (0 %)
Number of unique values	38
Median	-0.45
1st and 3rd quartiles	-1.07; 0.05
Min. and max.	-2.68; 2.28

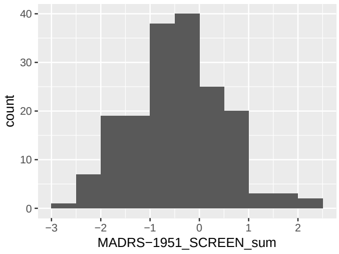

LSAS-2241_SCREEN_sum

Feature	Result
Variable type	numeric
Number of missing obs.	0 (0 %)
Number of unique values	94
Median	-0.51
1st and 3rd quartiles	-0.99; 0.15
Min. and max.	-1.79; 1.74

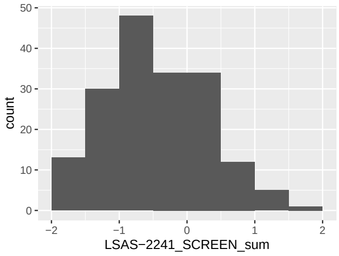

outcome

Feature	Result
Variable type	numeric
Number of missing obs.	0 (0 %)
Number of unique values	61
Median	-0.06
1st and 3rd quartiles	-0.71; 0.53
Min. and max.	-1.13; 2.8

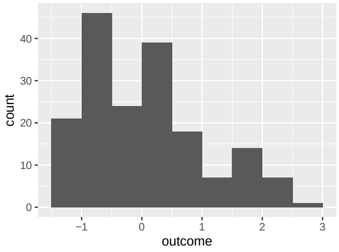

mainsymptom_PRE_sum

Feature	Result
Variable type	numeric
Number of missing obs.	0 (0 %)
Number of unique values	33
Median	-0.28
1st and 3rd quartiles	-0.68; 0.74
Min. and max.	-2.31; 2.78

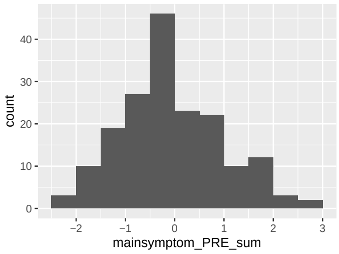

mainsymptom_WEEK01_sum

Feature	Result
Variable type	numeric
Number of missing obs.	0 (0 %)
Number of unique values	62
Median	-0.14
1st and 3rd quartiles	-0.69; 0.57
Min. and max.	-1.95; 3.51

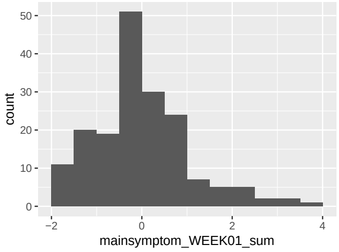

---

mainsymptom_WEEK02_sum

Feature	Result
Variable type	numeric
Number of missing obs.	0 (0 %)
Number of unique values	56
Median	-0.32
1st and 3rd quartiles	-0.77; 0.5
Min. and max.	-1.82; 3.02

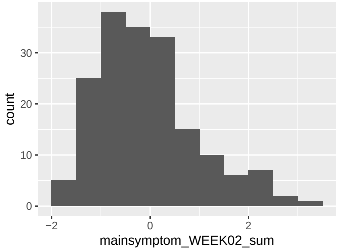

---

mainsymptom_WEEK03_sum

Feature	Result
Variable type	numeric
Number of missing obs.	0 (0 %)
Number of unique values	60
Median	-0.05
1st and 3rd quartiles	-0.87; 0.61
Min. and max.	-1.72; 2.51

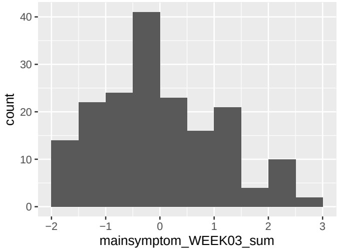

Report generation information:

- Created by: Could not determine from system (username: `nilisa`).
- Report creation time: Mon Jan 09 2023 13:13:49
- Report was run from directory: `/home/nilisa/projects/phd_study1/r`
- dataMaid v1.4.1 [Pkg: 2021-10-08 from CRAN (R 4.2.2)]
- R version 4.2.2 Patched (2022-11-10 r83330).
- Platform: x86_64-pc-linux-gnu (64-bit)(Ubuntu 20.04.5 LTS).
- Function call: `dataMaid::makeDataReport(data = gd, mode = c("summarize", "visualize", "check"), smartNum = FALSE, file = "~/projects/data/study1multiverse/results/graphs_n_figures/codebooks/codebook replace = TRUE, openResult = FALSE, checks = list(character = "showAllFactorLevels", factor = "showAllFactorLevels", labelled = "showAllFactorLevels",   haven_labelled = "showAllFactorLevels", numeric = NULL,   integer = NULL, logical = NULL, Date = NULL), listChecks = FALSE, maxProbVals = Inf, codebook = TRUE, reportTitle = "Handpicked_Panic_week04-impute`

Handpicked_Panic_week04-imputed_benchmark_train

Autogenerated data summary from dataMaid

2023-01-09 13:07:24

Data report overview

The dataset examined has the following dimensions:

Feature	Result
Number of observations	1590
Number of variables	10

Codebook summary table

Label	Variable	Class	# unique values	Missing	Description
	**sex**	factor	2	0.00 %	Sex of patient, 0 = Female, 1=Male
	**age**	numeric	59	0.00 %	
	**PDSS-SR-3064_SCREEN_sum**	numeric	173	0.00 %	Anxiety questionnaire, self rated-Timepoint before treatment starts-Sum of the entire measure
	**MADRS-1951_SCREEN_sum**	numeric	76	0.00 %	Depression questionnaire, self rated-Timepoint before treatment starts-Sum of the entire measure
	**LSAS-2241_SCREEN_sum**	numeric	342	0.00 %	Social anxiety questionnaire, self rated-Timepoint before treatment starts-Sum of the entire measure
	**outcome**	numeric	423	0.00 %	
	**mainsymptom_PRE_sum**	numeric	70	0.00 %	PDSS-SR for panic, MADRS for depression, LSAS for social anxiety-Timepoint just before beginning treatment-Sum of the entire measure
	**mainsymptom_WEEK01_sum**	numeric	337	0.00 %	PDSS-SR for panic, MADRS for depression, LSAS for social anxiety-Timepoint after one week in treatment-Sum of the entire measure
	**mainsymptom_WEEK02_sum**	numeric	329	0.00 %	PDSS-SR for panic, MADRS for depression, LSAS for social anxiety-Timepoint after two weeks in treatment-Sum of the entire measure

Label	Variable	Class	# unique values	Missing	Description
	**mainsymptom_WEEK03_sum**	numeric	322	0.00 %	PDSS-SR for panic, MADRS for depression, LSAS for social anxiety-Timepoint after three weeks in treatment-Sum of the entire measure

Variable list

sex

Feature	Result
Variable type	factor
Number of missing obs.	0 (0 %)
Number of unique values	2
Mode	"0"
Reference category	0

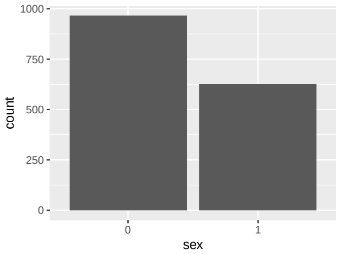

- Observed factor levels: "0", "1".

---

age

Feature	Result
Variable type	numeric
Number of missing obs.	0 (0 %)
Number of unique values	59
Median	-0.29
1st and 3rd quartiles	-0.81; 0.41
Min. and max.	-1.69; 3.55

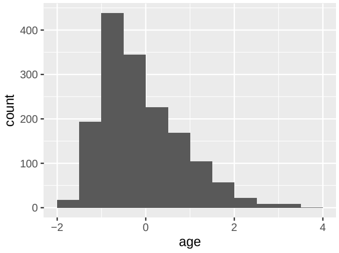

PDSS-SR-3064_SCREEN_sum

Feature	Result
Variable type	numeric
Number of missing obs.	0 (0 %)
Number of unique values	173
Median	0.75
1st and 3rd quartiles	0.28; 1.38
Min. and max.	-1.29; 3.12

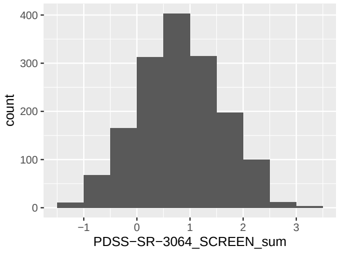

MADRS-1951_SCREEN_sum

Feature	Result
Variable type	numeric
Number of missing obs.	0 (0 %)
Number of unique values	76
Median	-0.45
1st and 3rd quartiles	-1.19; 0.17
Min. and max.	-2.68; 3.4

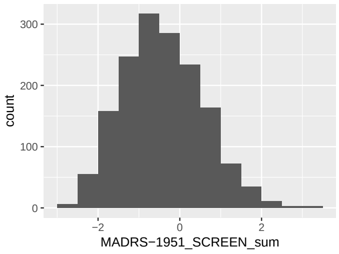

LSAS-2241_SCREEN_sum

Feature	Result
Variable type	numeric
Number of missing obs.	0 (0 %)
Number of unique values	342
Median	-0.56
1st and 3rd quartiles	-1.1; 0.08
Min. and max.	-1.79; 3.02

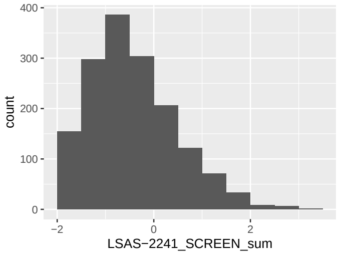

outcome

Feature	Result
Variable type	numeric
Number of missing obs.	0 (0 %)
Number of unique values	423
Median	-0.09
1st and 3rd quartiles	-0.71; 0.53
Min. and max.	-1.13; 4.66

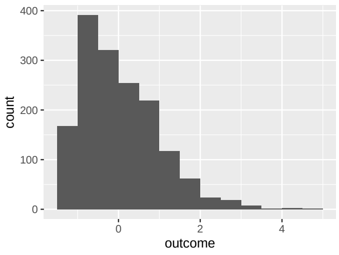

mainsymptom_PRE_sum

Feature	Result
Variable type	numeric
Number of missing obs.	0 (0 %)
Number of unique values	70
Median	-0.07
1st and 3rd quartiles	-0.68; 0.74
Min. and max.	-2.31; 3.39

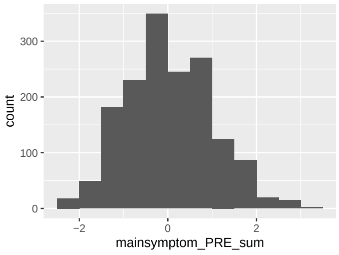

mainsymptom_WEEK01_sum

Feature	Result
Variable type	numeric
Number of missing obs.	0 (0 %)
Number of unique values	337
Median	-0.06
1st and 3rd quartiles	-0.69; 0.57
Min. and max.	-1.95; 3.51

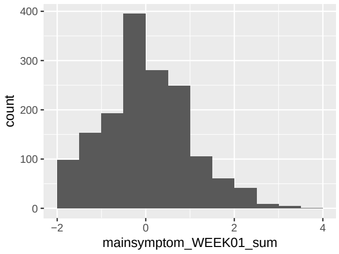

---

mainsymptom_WEEK02_sum

Feature	Result
Variable type	numeric
Number of missing obs.	0 (0 %)
Number of unique values	329
Median	-0.13
1st and 3rd quartiles	-0.69; 0.71
Min. and max.	-1.82; 3.86

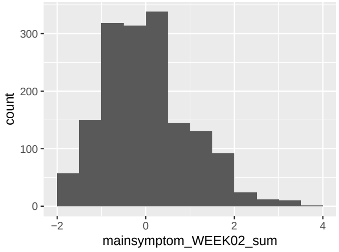

---

mainsymptom_WEEK03_sum

Feature	Result
Variable type	numeric
Number of missing obs.	0 (0 %)
Number of unique values	322
Median	-0.02
1st and 3rd quartiles	-0.66; 0.61
Min. and max.	-1.72; 3.99

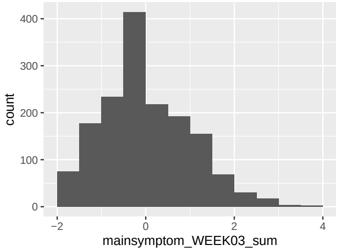

Report generation information:

- Created by: Could not determine from system (username: `nilisa`).
- Report creation time: Mon Jan 09 2023 13:07:24
- Report was run from directory: `/home/nilisa/projects/phd_study1/r`
- dataMaid v1.4.1 [Pkg: 2021-10-08 from CRAN (R 4.2.2)]
- R version 4.2.2 Patched (2022-11-10 r83330).
- Platform: x86_64-pc-linux-gnu (64-bit)(Ubuntu 20.04.5 LTS).
- Function call: `dataMaid::makeDataReport(data = gd, mode = c("summarize", "visualize", "check"), smartNum = FALSE, file = "~/projects/data/study1multiverse/results/graphs_n_figures/codebooks/codebook replace = TRUE, openResult = FALSE, checks = list(character = "showAllFactorLevels", factor = "showAllFactorLevels", labelled = "showAllFactorLevels",   haven_labelled = "showAllFactorLevels", numeric = NULL,   integer = NULL, logical = NULL, Date = NULL), listChecks = FALSE, maxProbVals = Inf, codebook = TRUE, reportTitle = "Handpicked_Panic_week04-impute`

Handpicked_Panic_week04-imputed_test

Autogenerated data summary from dataMaid

2023-01-09 13:13:56

Data report overview

The dataset examined has the following dimensions:

Feature	Result
Number of observations	177
Number of variables	61

Codebook summary table

Label	Variable	Class	# unique values	Missing	Description
	**sex**	factor	2	0.00 %	Sex of patient, 0 = Female, 1=Male
	**age**	numeric	45	0.00 %	
	**messages_len_7**	numeric	62	0.00 %	-Meta information of messages-Length of messages-up until day-7
	**messages_len_tp_7**	numeric	128	0.00 %	-Meta information of messages-Length of messages-therapist messages-up until day-7
	**messages_7**	numeric	6	0.00 %	-Meta information of messages-up until day-7
	**messages_tp_7**	numeric	6	0.00 %	-Meta information of messages-therapist messages-up until day-7
	**homeworks_7**	numeric	5	0.00 %	-Number of homework messages sent in-up until day-7
	**messages_len_14**	numeric	72	0.00 %	-Meta information of messages-Length of messages-up until day-14
	**messages_len_tp_14**	numeric	140	0.00 %	-Meta information of messages-Length of messages-therapist messages-up until day-14
	**messages_14**	numeric	5	0.00 %	-Meta information of messages-up until day-14
	**messages_tp_14**	numeric	5	0.00 %	-Meta information of messages-therapist messages-up until day-14

Label	Variable	Class	# unique values	Missing	Description
	**homeworks_14**	numeric	6	0.00 %	-Number of homework messages sent in-up until day-14
	**messages_len_21**	numeric	76	0.00 %	-Meta information of messages-Length of messages-up until day-21
	**messages_len_tp_21**	numeric	132	0.00 %	-Meta information of messages-Length of messages-therapist messages-up until day-21
	**messages_21**	numeric	7	0.00 %	-Meta information of messages-up until day-21
	**messages_tp_21**	numeric	5	0.00 %	-Meta information of messages-therapist messages-up until day-21
	**homeworks_21**	numeric	5	0.00 %	-Number of homework messages sent in-up until day-21
	**messages_len_28**	numeric	70	0.00 %	-Meta information of messages-Length of messages-up until day-28
	**messages_len_tp_28**	numeric	121	0.00 %	-Meta information of messages-Length of messages-therapist messages-up until day-28
	**messages_28**	numeric	5	0.00 %	-Meta information of messages-up until day-28
	**messages_tp_28**	numeric	5	0.00 %	-Meta information of messages-therapist messages-up until day-28
	**homeworks_28**	numeric	4	0.00 %	-Number of homework messages sent in-up until day-28
	**PDSS-SR-3064_SCREEN_sum**	numeric	38	0.00 %	Anxiety questionnaire, self rated-Timepoint before treatment starts-Sum of the entire measure
	**MADRS-1951_SCREEN_sum**	numeric	37	0.00 %	Depression questionnaire, self rated-Timepoint before treatment starts-Sum of the entire measure
	**LSAS-2241_SCREEN_sum**	numeric	94	0.00 %	Social anxiety questionnaire, self rated-Timepoint before treatment starts-Sum of the entire measure
	**MADRS-1951_SCREEN_DateCompleted_day**	numeric	10	0.00 %	Depression questionnaire, self rated-Timepoint before treatment starts-Cyclic transformation of what day 0-6 during week it was filled in

Label	Variable	Class	# unique values	Missing	Description
	**MADRS-1951_SCREEN_DateCompleted_time**	numeric	157	0.00 %	Depression questionnaire, self rated-Timepoint before treatment starts-Cyclic transformation of what time during day 0-1440 it was filled in
	**PDSS-SR-3064_SCREEN_DateCompleted_day**	numeric	12	0.00 %	Anxiety questionnaire, self rated-Timepoint before treatment starts-Cyclic transformation of what day 0-6 during week it was filled in
	**PDSS-SR-3064_SCREEN_DateCompleted_time**	numeric	162	0.00 %	Anxiety questionnaire, self rated-Timepoint before treatment starts-Cyclic transformation of what time during day 0-1440 it was filled in
	**LSAS-2241_SCREEN_DateCompleted_day**	numeric	15	0.00 %	Social anxiety questionnaire, self rated-Timepoint before treatment starts-Cyclic transformation of what day 0-6 during week it was filled in
	**LSAS-2241_SCREEN_DateCompleted_time**	numeric	166	0.00 %	Social anxiety questionnaire, self rated-Timepoint before treatment starts-Cyclic transformation of what time during day 0-1440 it was filled in
	**outcome**	numeric	61	0.00 %	
	**ncomorbid**	numeric	18	0.00 %	
	**HW-01**	numeric	173	0.00 %	
	**currentwork_proff**	factor	7	0.00 %	Currently in work for trained proffession
	**Marital_1833_gift**	factor	2	0.00 %	Marital status: Married or not
	**Marital_1833_separerad**	factor	2	0.00 %	Marital status: divocered/equivalent
	**Marital_1833_singel**	factor	2	0.00 %	Marital status: single
	**Edu_1843_2**	factor	2	0.00 %	7-9 years education
	**Edu_1843_3**	factor	2	0.00 %	Uncompleted upper secondary school
	**Edu_1843_4**	factor	2	0.00 %	Higher vocational education
	**Edu_1843_5**	factor	2	0.00 %	Completed upper secondary school
	**Edu_1843_6**	factor	2	0.00 %	Uncompleted university degree
	**Edu_1843_7**	factor	2	0.00 %	University degree
	**cscale**	numeric	51	0.00 %	
	**mainsymptom_PRE_sum**	numeric	33	0.00 %	PDSS-SR for panic, MADRS for depression, LSAS for social anxiety-Timepoint just before beginning treatment-Sum of the entire measure

Label	Variable	Class	# unique values	Missing	Description
	**mainsymptom_PRE_duration**	numeric	150	0.00 %	PDSS-SR for panic, MADRS for depression, LSAS for social anxiety-Timepoint just before beginning treatment-Time to fill in measure/questionnaire
	**mainsymptom_PRE_DateCompleted_day**	numeric	15	0.00 %	PDSS-SR for panic, MADRS for depression, LSAS for social anxiety-Timepoint just before beginning treatment-Cyclic transformation of what day 0-6 during week it was filled in
	**mainsymptom_PRE_DateCompleted_time**	numeric	153	0.00 %	PDSS-SR for panic, MADRS for depression, LSAS for social anxiety-Timepoint just before beginning treatment-Cyclic transformation of what time during day 0-1440 it was filled in
	**mainsymptom_WEEK01_sum**	numeric	61	0.00 %	PDSS-SR for panic, MADRS for depression, LSAS for social anxiety-Timepoint after one week in treatment-Sum of the entire measure
	**mainsymptom_WEEK01_duration**	numeric	148	0.00 %	PDSS-SR for panic, MADRS for depression, LSAS for social anxiety-Timepoint after one week in treatment-Time to fill in measure/questionnaire
	**mainsymptom_WEEK01_DateCompleted_day**	numeric	44	0.00 %	PDSS-SR for panic, MADRS for depression, LSAS for social anxiety-Timepoint after one week in treatment-Cyclic transformation of what day 0-6 during week it was filled in
	**mainsymptom_WEEK01_DateCompleted_time**	numeric	164	0.00 %	PDSS-SR for panic, MADRS for depression, LSAS for social anxiety-Timepoint after one week in treatment-Cyclic transformation of what time during day 0-1440 it was filled in
	**mainsymptom_WEEK02_sum**	numeric	56	0.00 %	PDSS-SR for panic, MADRS for depression, LSAS for social anxiety-Timepoint after two weeks in treatment-Sum of the entire measure
	**mainsymptom_WEEK02_duration**	numeric	144	0.00 %	PDSS-SR for panic, MADRS for depression, LSAS for social anxiety-Timepoint after two weeks in treatment-Time to fill in measure/questionnaire

Label	Variable	Class	# unique values	Missing	Description
	**mainsymptom_WEEK02_DateCompleted_day**	numeric	40	0.00 %	PDSS-SR for panic, MADRS for depression, LSAS for social anxiety-Timepoint after two weeks in treatment-Cyclic transformation of what day 0-6 during week it was filled in
	**mainsymptom_WEEK02_DateCompleted_time**	numeric	165	0.00 %	PDSS-SR for panic, MADRS for depression, LSAS for social anxiety-Timepoint after two weeks in treatment-Cyclic transformation of what time during day 0-1440 it was filled in
	**mainsymptom_WEEK03_sum**	numeric	60	0.00 %	PDSS-SR for panic, MADRS for depression, LSAS for social anxiety-Timepoint after three weeks in treatment-Sum of the entire measure
	**mainsymptom_WEEK03_duration**	numeric	144	0.00 %	PDSS-SR for panic, MADRS for depression, LSAS for social anxiety-Timepoint after three weeks in treatment-Time to fill in measure/questionnaire
	**mainsymptom_WEEK03_DateCompleted_day**	numeric	47	0.00 %	PDSS-SR for panic, MADRS for depression, LSAS for social anxiety-Timepoint after three weeks in treatment-Cyclic transformation of what day 0-6 during week it was filled in
	**mainsymptom_WEEK03_DateCompleted_time**	numeric	167	0.00 %	PDSS-SR for panic, MADRS for depression, LSAS for social anxiety-Timepoint after three weeks in treatment-Cyclic transformation of what time during day 0-1440 it was filled in

Variable list

sex

Feature	Result
Variable type	factor
Number of missing obs.	0 (0 %)
Number of unique values	2
Mode	“0”
Reference category	0

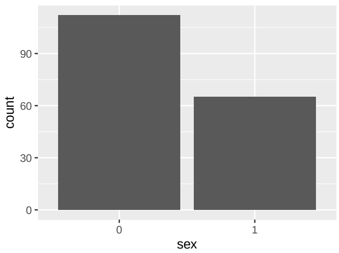

- Observed factor levels: "0", "1".

---

age

Feature	Result
Variable type	numeric
Number of missing obs.	0 (0 %)
Number of unique values	45
Median	-0.46
1st and 3rd quartiles	-0.9; 0.32
Min. and max.	-1.6; 2.77

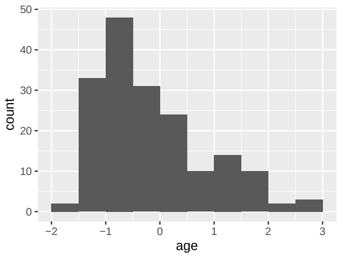

messages_len_7

Feature	Result
Variable type	numeric
Number of missing obs.	0 (0 %)
Number of unique values	62
Median	-0.32
1st and 3rd quartiles	-0.32; 0.06
Min. and max.	-0.32; 9.21

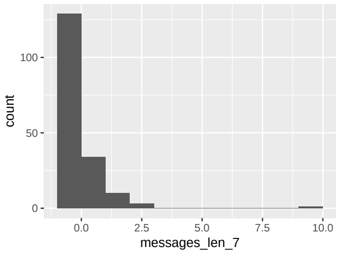

messages_len_tp_7

Feature	Result
Variable type	numeric
Number of missing obs.	0 (0 %)
Number of unique values	128
Median	-0.18
1st and 3rd quartiles	-0.94; 0.46
Min. and max.	-1.46; 4.25

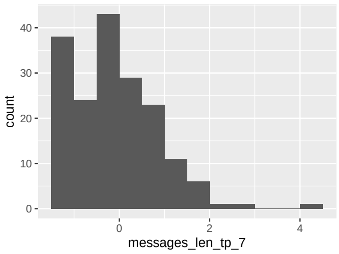

messages_7

Feature	Result
Variable type	numeric
Number of missing obs.	0 (0 %)
Number of unique values	6
Median	-0.55
1st and 3rd quartiles	-0.55; 0.58
Min. and max.	-0.55; 5.13

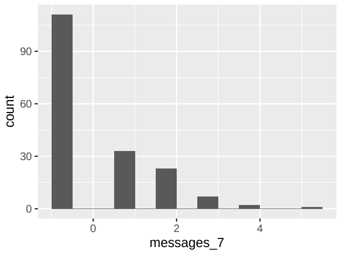

messages_tp_7

Feature	Result
Variable type	numeric
Number of missing obs.	0 (0 %)
Number of unique values	6
Median	0.15
1st and 3rd quartiles	-0.91; 1.21
Min. and max.	-1.97; 3.34

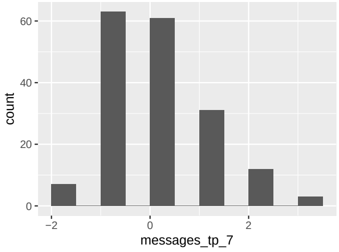

homeworks_7

Feature	Result
Variable type	numeric
Number of missing obs.	0 (0 %)
Number of unique values	5
Median	0.2
1st and 3rd quartiles	-1.03; 0.2
Min. and max.	-1.03; 3.91

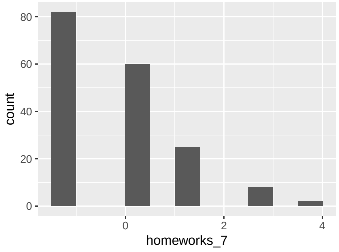

messages_len_14

Feature	Result
Variable type	numeric
Number of missing obs.	0 (0 %)
Number of unique values	72
Median	-0.41
1st and 3rd quartiles	-0.41; -0.02
Min. and max.	-0.41; 4.63

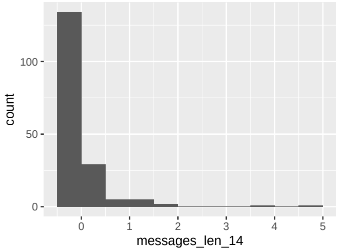

messages_len_tp_14

Feature	Result
Variable type	numeric
Number of missing obs.	0 (0 %)
Number of unique values	140
Median	-0.31
1st and 3rd quartiles	-0.76; 0.14
Min. and max.	-1.11; 4.65

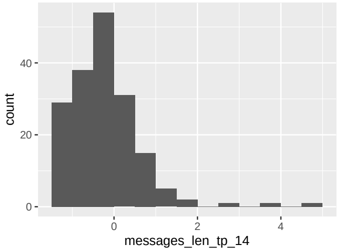

messages_14

Feature	Result
Variable type	numeric
Number of missing obs.	0 (0 %)
Number of unique values	5
Median	-0.7
1st and 3rd quartiles	-0.7; 0.36
Min. and max.	-0.7; 3.54

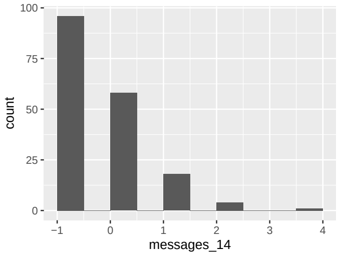

messages_tp_14

Feature	Result
Variable type	numeric
Number of missing obs.	0 (0 %)
Number of unique values	5
Median	-0.35
1st and 3rd quartiles	-0.35; 0.87
Min. and max.	-1.57; 3.31

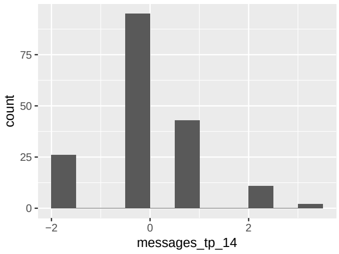

homeworks_14

Feature	Result
Variable type	numeric
Number of missing obs.	0 (0 %)
Number of unique values	6
Median	0.33
1st and 3rd quartiles	-0.98; 0.33
Min. and max.	-0.98; 5.58

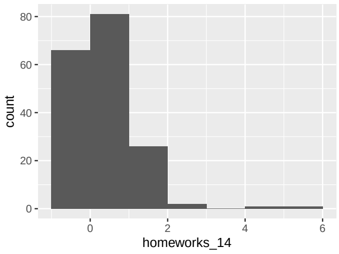

messages_len_21

Feature	Result
Variable type	numeric
Number of missing obs.	0 (0 %)
Number of unique values	76
Median	-0.46
1st and 3rd quartiles	-0.46; 0
Min. and max.	-0.46; 5.5

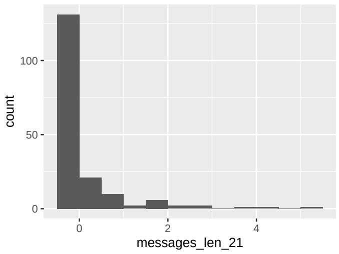

messages_len_tp_21

Feature	Result
Variable type	numeric
Number of missing obs.	0 (0 %)
Number of unique values	132
Median	-0.42
1st and 3rd quartiles	-0.85; 0.08
Min. and max.	-1.01; 5.8

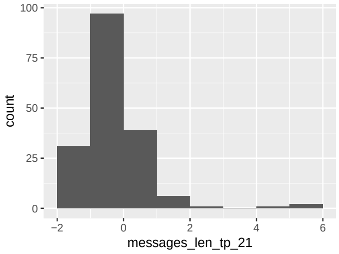

messages_21

Feature	Result
Variable type	numeric
Number of missing obs.	0 (0 %)
Number of unique values	7
Median	-0.74
1st and 3rd quartiles	-0.74; 0.34
Min. and max.	-0.74; 5.75

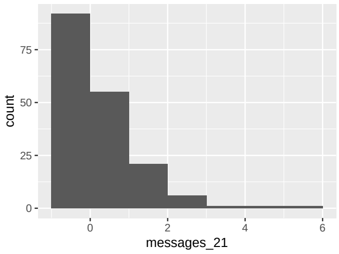

messages_tp_21

Feature	Result
Variable type	numeric
Number of missing obs.	0 (0 %)
Number of unique values	5
Median	-0.32
1st and 3rd quartiles	-0.32; 0.84
Min. and max.	-1.49; 3.17

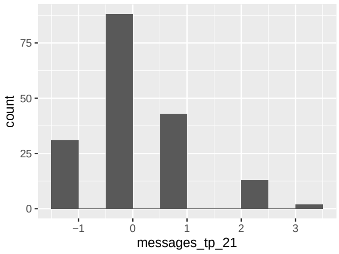

homeworks_21

Feature	Result
Variable type	numeric
Number of missing obs.	0 (0 %)
Number of unique values	5
Median	0.36
1st and 3rd quartiles	-1.01; 0.36
Min. and max.	-1.01; 5.86

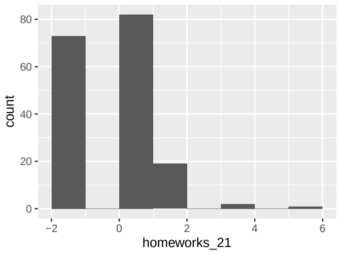

messages_len_28

Feature	Result
Variable type	numeric
Number of missing obs.	0 (0 %)
Number of unique values	70
Median	-0.41
1st and 3rd quartiles	-0.41; 0.04
Min. and max.	-0.41; 3.53

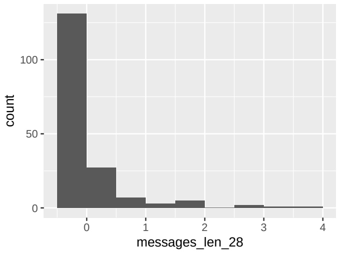

messages_len_tp_28

Feature	Result
Variable type	numeric
Number of missing obs.	0 (0 %)
Number of unique values	121
Median	-0.44
1st and 3rd quartiles	-0.85; 0.05
Min. and max.	-0.94; 3.35

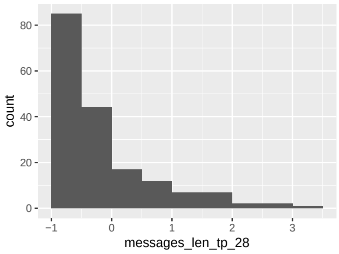

messages_28

Feature	Result
Variable type	numeric
Number of missing obs.	0 (0 %)
Number of unique values	5
Median	-0.72
1st and 3rd quartiles	-0.72; 0.41
Min. and max.	-0.72; 4.94

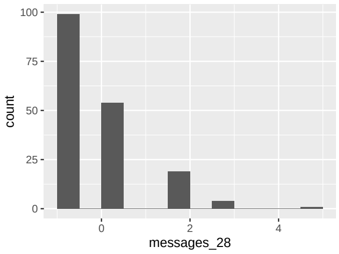

messages_tp_28

Feature	Result
Variable type	numeric
Number of missing obs.	0 (0 %)
Number of unique values	5
Median	-0.24
1st and 3rd quartiles	-0.24; 0.93
Min. and max.	-1.42; 3.28

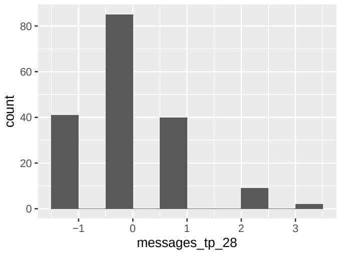

homeworks_28

Feature	Result
Variable type	numeric
Number of missing obs.	0 (0 %)
Number of unique values	4
Median	-0.91
1st and 3rd quartiles	-0.91; 0.46
Min. and max.	-0.91; 3.2

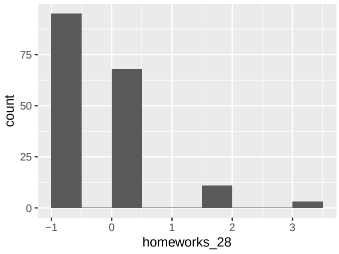

PDSS-SR-3064_SCREEN_sum

Feature	Result
Variable type	numeric
Number of missing obs.	0 (0 %)
Number of unique values	38
Median	0.75
1st and 3rd quartiles	0.25; 1.23
Min. and max.	-1.14; 2.65

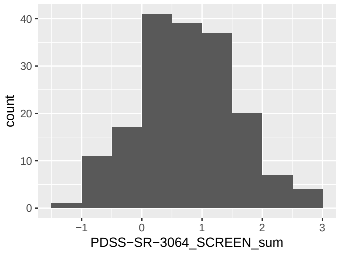

MADRS-1951_SCREEN_sum

Feature	Result
Variable type	numeric
Number of missing obs.	0 (0 %)
Number of unique values	37
Median	-0.45
1st and 3rd quartiles	-1.07; 0.05
Min. and max.	-2.68; 2.28

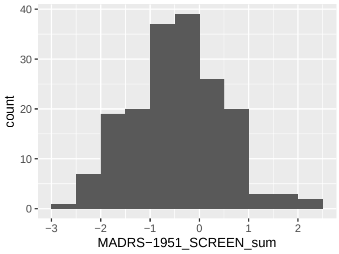

LSAS-2241_SCREEN_sum

Feature	Result
Variable type	numeric
Number of missing obs.	0 (0 %)
Number of unique values	94
Median	-0.51
1st and 3rd quartiles	-0.96; 0.01
Min. and max.	-1.79; 1.74

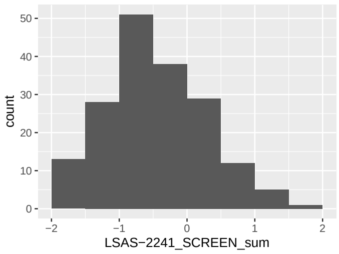

MADRS-1951_SCREEN_DateCompleted_day

Feature	Result
Variable type	numeric
Number of missing obs.	0 (0 %)
Number of unique values	10
Median	1
1st and 3rd quartiles	-0.37; 1
Min. and max.	-1.37; 1.37

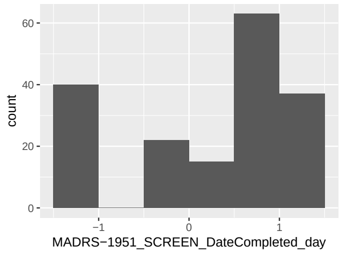

MADRS-1951_SCREEN_DateCompleted_time

Feature	Result
Variable type	numeric
Number of missing obs.	0 (0 %)
Number of unique values	157
Median	-0.8
1st and 3rd quartiles	-1.24; -0.01
Min. and max.	-1.41; 1.41

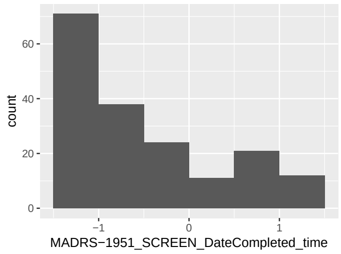

PDSS-SR-3064_SCREEN_DateCompleted_day

Feature	Result
Variable type	numeric
Number of missing obs.	0 (0 %)
Number of unique values	12
Median	1
1st and 3rd quartiles	-0.37; 1
Min. and max.	-1.37; 1.37

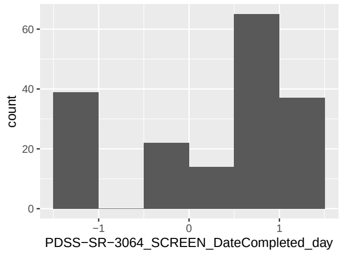

PDSS-SR-3064_SCREEN_DateCompleted_time

Feature	Result
Variable type	numeric
Number of missing obs.	0 (0 %)
Number of unique values	162
Median	-0.81
1st and 3rd quartiles	-1.23; -0.01
Min. and max.	-1.41; 1.41

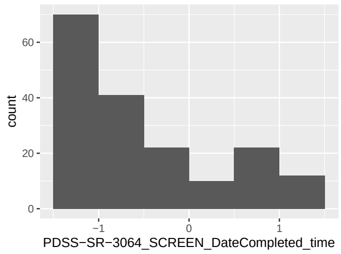

LSAS-2241_SCREEN_DateCompleted_day

Feature	Result
Variable type	numeric
Number of missing obs.	0 (0 %)
Number of unique values	15
Median	1
1st and 3rd quartiles	-0.37; 1
Min. and max.	-1.37; 1.37

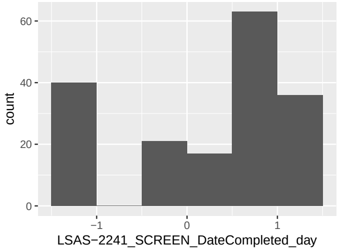

LSAS-2241_SCREEN_DateCompleted_time

Feature	Result
Variable type	numeric
Number of missing obs.	0 (0 %)
Number of unique values	166
Median	-0.8
1st and 3rd quartiles	-1.23; 0.05
Min. and max.	-1.41; 1.41

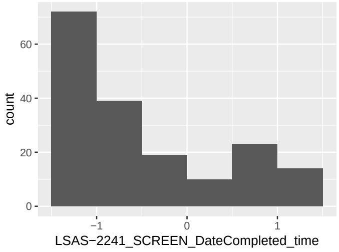

outcome

Feature	Result
Variable type	numeric
Number of missing obs.	0 (0 %)
Number of unique values	61
Median	-0.05
1st and 3rd quartiles	-0.71; 0.46
Min. and max.	-1.13; 2.8

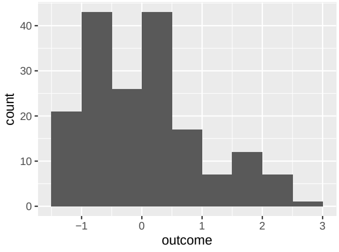

ncomorbid

Feature	Result
Variable type	numeric
Number of missing obs.	0 (0 %)
Number of unique values	18
Median	0
1st and 3rd quartiles	0; 1
Min. and max.	0; 3

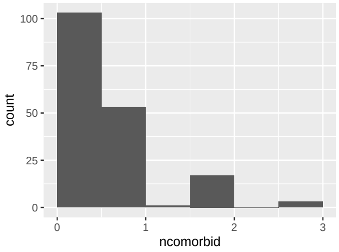

HW-01

Feature	Result
Variable type	numeric
Number of missing obs.	0 (0 %)
Number of unique values	173
Median	-0.11
1st and 3rd quartiles	-0.53; 0.23
Min. and max.	-4.03; 1.05

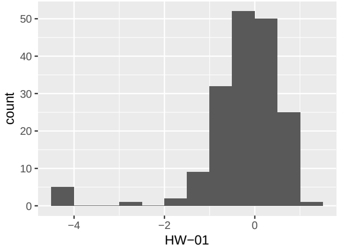

---

currentwork_proff

Feature	Result
Variable type	factor
Number of missing obs.	0 (0 %)
Number of unique values	7
Mode	“1”
Reference category	0

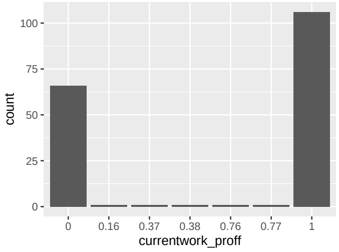

- Observed factor levels: "0", "0.16", "0.37", "0.38", "0.76", "0.77", "1".

Marital_1833_gift

Feature	Result
Variable type	factor
Number of missing obs.	0 (0 %)
Number of unique values	2
Mode	“1”
Reference category	0

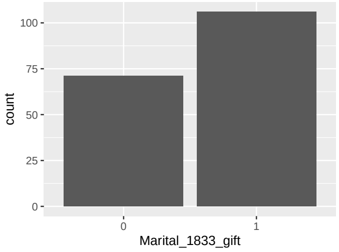

- Observed factor levels: "0", "1".

Marital_1833_separerad

Feature	Result
Variable type	factor
Number of missing obs.	0 (0 %)
Number of unique values	2
Mode	“0”
Reference category	0

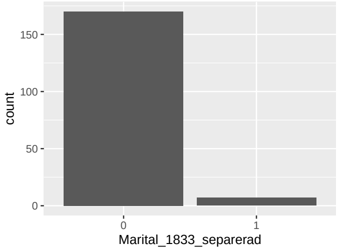

- Observed factor levels: "0", "1".

Marital_1833_singel

Feature	Result
Variable type	factor
Number of missing obs.	0 (0 %)
Number of unique values	2
Mode	"0"
Reference category	0

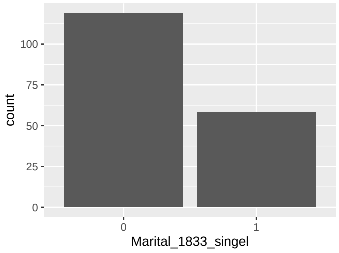

- Observed factor levels: "0", "1".

Edu_1843_2

Feature	Result
Variable type	factor
Number of missing obs.	0 (0 %)
Number of unique values	2
Mode	"0"
Reference category	0

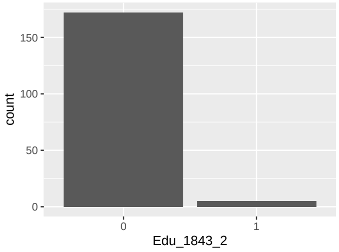

- Observed factor levels: "0", "1".

Edu_1843_3

Feature	Result
Variable type	factor
Number of missing obs.	0 (0 %)
Number of unique values	2
Mode	"0"
Reference category	0

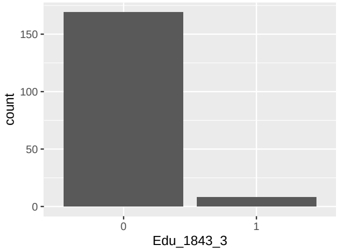

- Observed factor levels: "0", "1".

Edu_1843_4

Feature	Result
Variable type	factor
Number of missing obs.	0 (0 %)
Number of unique values	2
Mode	"0"
Reference category	0

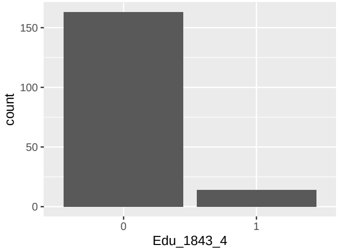

- Observed factor levels: "0", "1".

Edu_1843_5

Feature	Result
Variable type	factor
Number of missing obs.	0 (0 %)
Number of unique values	2
Mode	“0”
Reference category	0

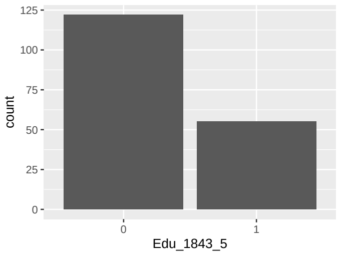

- Observed factor levels: "0", "1".

Edu_1843_6

Feature	Result
Variable type	factor
Number of missing obs.	0 (0 %)
Number of unique values	2
Mode	“0”
Reference category	0

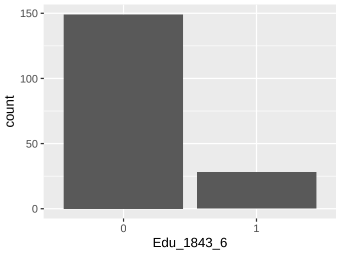

- Observed factor levels: "0", "1".

Edu_1843_7

Feature	Result
Variable type	factor
Number of missing obs.	0 (0 %)
Number of unique values	2
Mode	“0”
Reference category	0

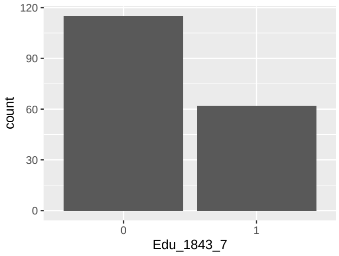

- Observed factor levels: "0", "1".

---

cscale

Feature	Result
Variable type	numeric
Number of missing obs.	0 (0 %)
Number of unique values	51
Median	0.37
1st and 3rd quartiles	-0.11; 0.96
Min. and max.	-2.6; 1.79

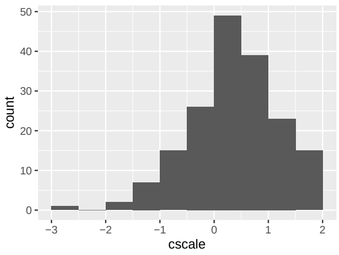

---

mainsymptom_PRE_sum

Feature	Result
Variable type	numeric
Number of missing obs.	0 (0 %)
Number of unique values	33
Median	-0.28
1st and 3rd quartiles	-0.89; 0.74
Min. and max.	-2.31; 2.78

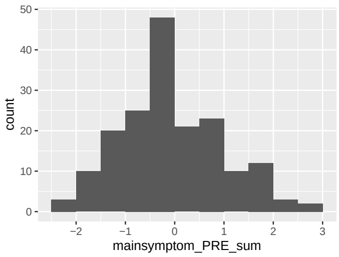

mainsymptom_PRE_duration

Feature	Result
Variable type	numeric
Number of missing obs.	0 (0 %)
Number of unique values	150
Median	-0.15
1st and 3rd quartiles	-0.19; -0.05
Min. and max.	-0.26; 20.63

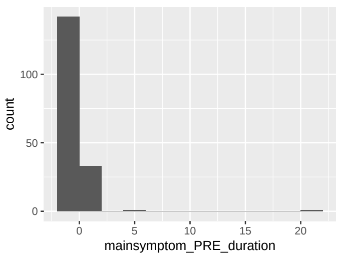

mainsymptom_PRE_DateCompleted_day

Feature	Result
Variable type	numeric
Number of missing obs.	0 (0 %)
Number of unique values	15
Median	0.21
1st and 3rd quartiles	-1; 1
Min. and max.	-1.37; 1.37

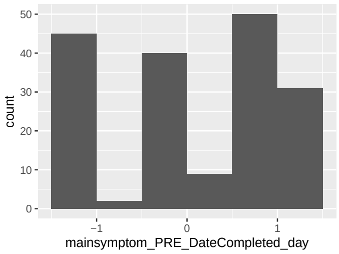

mainsymptom_PRE_DateCompleted_time

Feature	Result
Variable type	numeric
Number of missing obs.	0 (0 %)
Number of unique values	153
Median	-0.72
1st and 3rd quartiles	-1.19; 0.1
Min. and max.	-1.41; 1.41

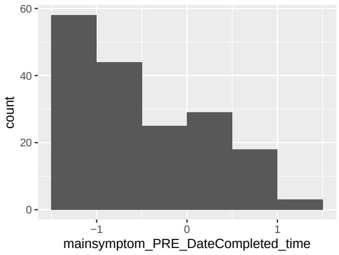

mainsymptom_WEEK01_sum

Feature	Result
Variable type	numeric
Number of missing obs.	0 (0 %)
Number of unique values	61
Median	-0.12
1st and 3rd quartiles	-0.69; 0.57
Min. and max.	-1.95; 3.51

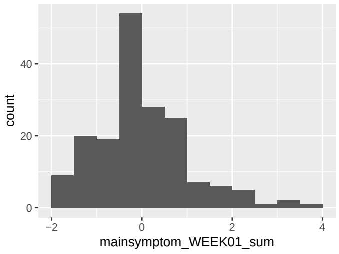

mainsymptom_WEEK01_duration

Feature	Result
Variable type	numeric
Number of missing obs.	0 (0 %)
Number of unique values	148
Median	-0.13
1st and 3rd quartiles	-0.21; -0.02
Min. and max.	-0.36; 7.81

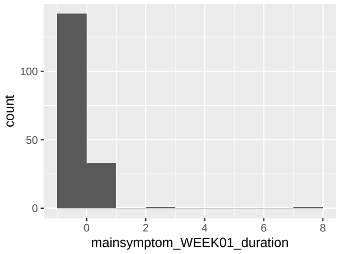

mainsymptom_WEEK01_DateCompleted_day

Feature	Result
Variable type	numeric
Number of missing obs.	0 (0 %)
Number of unique values	44
Median	0.44
1st and 3rd quartiles	-0.37; 1
Min. and max.	-1.37; 1.37

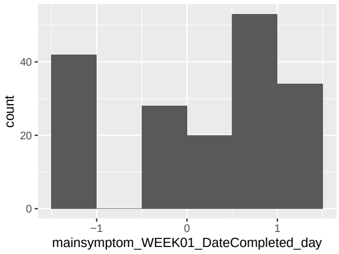

mainsymptom_WEEK01_DateCompleted_time

Feature	Result
Variable type	numeric
Number of missing obs.	0 (0 %)
Number of unique values	164
Median	-0.64
1st and 3rd quartiles	-1.1; -0.06
Min. and max.	-1.41; 1.39

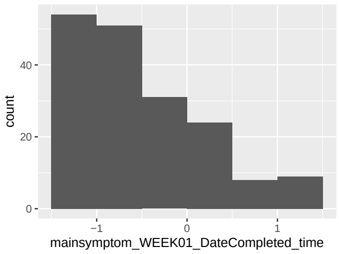

mainsymptom_WEEK02_sum

Feature	Result
Variable type	numeric
Number of missing obs.	0 (0 %)
Number of unique values	56
Median	-0.3
1st and 3rd quartiles	-0.77; 0.5
Min. and max.	-1.82; 3.02

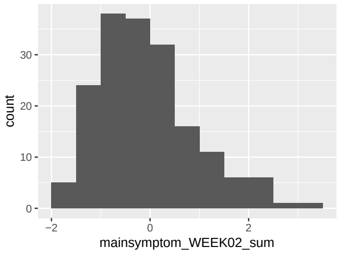

mainsymptom_WEEK02_duration

Feature	Result
Variable type	numeric
Number of missing obs.	0 (0 %)
Number of unique values	144
Median	-0.18
1st and 3rd quartiles	-0.24; -0.06
Min. and max.	-0.35; 14.21

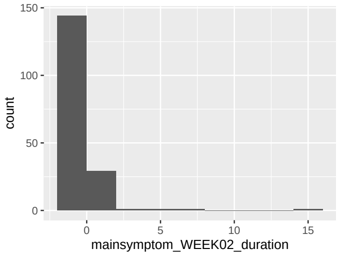

mainsymptom_WEEK02_DateCompleted_day

Feature	Result
Variable type	numeric
Number of missing obs.	0 (0 %)
Number of unique values	40
Median	0.74
1st and 3rd quartiles	-0.37; 1
Min. and max.	-1.37; 1.37

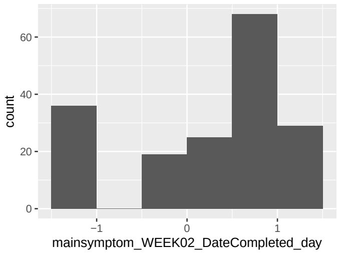

mainsymptom_WEEK02_DateCompleted_time

Feature	Result
Variable type	numeric
Number of missing obs.	0 (0 %)
Number of unique values	165
Median	-0.8
1st and 3rd quartiles	-1.23; -0.28
Min. and max.	-1.41; 1.41

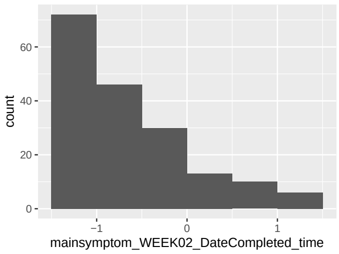

mainsymptom_WEEK03_sum

Feature	Result
Variable type	numeric
Number of missing obs.	0 (0 %)
Number of unique values	60
Median	-0.02
1st and 3rd quartiles	-0.87; 0.61
Min. and max.	-1.72; 2.51

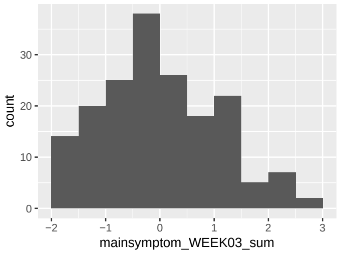

---

mainsymptom_WEEK03_duration

Feature	Result
Variable type	numeric
Number of missing obs.	0 (0 %)
Number of unique values	144
Median	-0.11
1st and 3rd quartiles	-0.17; -0.04
Min. and max.	-0.27; 5.07

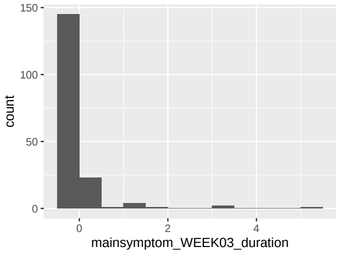

---

mainsymptom_WEEK03_DateCompleted_day

Feature	Result
Variable type	numeric
Number of missing obs.	0 (0 %)
Number of unique values	47
Median	0.27
1st and 3rd quartiles	-0.37; 1
Min. and max.	-1.37; 1.37

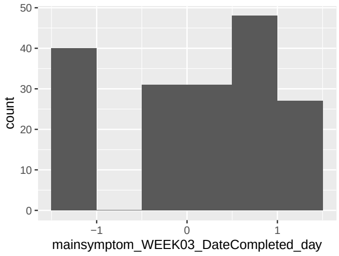

---

mainsymptom_WEEK03_DateCompleted_time

Feature	Result
Variable type	numeric
Number of missing obs.	0 (0 %)
Number of unique values	167
Median	-0.71
1st and 3rd quartiles	-1.14; -0.13
Min. and max.	-1.41; 1.38

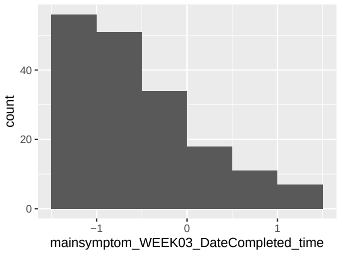

Report generation information:

- Created by: Could not determine from system (username: `nilisa`).
- Report creation time: Mon Jan 09 2023 13:13:57
- Report was run from directory: `/home/nilisa/projects/phd_study1/r`

- dataMaid v1.4.1 [Pkg: 2021-10-08 from CRAN (R 4.2.2)]
- R version 4.2.2 Patched (2022-11-10 r83330).
- Platform: x86_64-pc-linux-gnu (64-bit)(Ubuntu 20.04.5 LTS).
- Function call: `dataMaid::makeDataReport(data = gd, mode = c("summarize", "visualize", "check"), smartNum = FALSE, file = "~/projects/data/study1multiverse/results/graphs_n_figures/codebooks/codebook replace = TRUE, openResult = FALSE, checks = list(character = "showAllFactorLevels", factor = "showAllFactorLevels", labelled = "showAllFactorLevels",   haven_labelled = "showAllFactorLevels", numeric = NULL,   integer = NULL, logical = NULL, Date = NULL), listChecks = FALSE, maxProbVals = Inf, codebook = TRUE, reportTitle = "Handpicked_Panic_week04-impute`

Handpicked_Panic_week04-imputed_train

Autogenerated data summary from dataMaid

2023-01-09 13:07:32

Data report overview

The dataset examined has the following dimensions:

Feature	Result
Number of observations	1590
Number of variables	61

Codebook summary table

Label	Variable	Class	# unique values	Missing	Description
	**sex**	factor	2	0.00 %	Sex of patient, 0 = Female, 1=Male
	**age**	numeric	59	0.00 %	
	**messages_len_7**	numeric	350	0.00 %	-Meta information of messages-Length of messages-up until day-7
	**messages_len_tp_7**	numeric	657	0.00 %	-Meta information of messages-Length of messages-therapist messages-up until day-7
	**messages_7**	numeric	9	0.00 %	-Meta information of messages-up until day-7
	**messages_tp_7**	numeric	9	0.00 %	-Meta information of messages-therapist messages-up until day-7
	**homeworks_7**	numeric	7	0.00 %	-Number of homework messages sent in-up until day-7
	**messages_len_14**	numeric	371	0.00 %	-Meta information of messages-Length of messages-up until day-14
	**messages_len_tp_14**	numeric	665	0.00 %	-Meta information of messages-Length of messages-therapist messages-up until day-14
	**messages_14**	numeric	8	0.00 %	-Meta information of messages-up until day-14
	**messages_tp_14**	numeric	6	0.00 %	-Meta information of messages-therapist messages-up until day-14

Label	Variable	Class	# unique values	Missing	Description
	**homeworks_14**	numeric	5	0.00 %	-Number of homework messages sent in-up until day-14
	**messages_len_21**	numeric	353	0.00 %	-Meta information of messages-Length of messages-up until day-21
	**messages_len_tp_21**	numeric	632	0.00 %	-Meta information of messages-Length of messages-therapist messages-up until day-21
	**messages_21**	numeric	9	0.00 %	-Meta information of messages-up until day-21
	**messages_tp_21**	numeric	8	0.00 %	-Meta information of messages-therapist messages-up until day-21
	**homeworks_21**	numeric	6	0.00 %	-Number of homework messages sent in-up until day-21
	**messages_len_28**	numeric	339	0.00 %	-Meta information of messages-Length of messages-up until day-28
	**messages_len_tp_28**	numeric	583	0.00 %	-Meta information of messages-Length of messages-therapist messages-up until day-28
	**messages_28**	numeric	9	0.00 %	-Meta information of messages-up until day-28
	**messages_tp_28**	numeric	7	0.00 %	-Meta information of messages-therapist messages-up until day-28
	**homeworks_28**	numeric	6	0.00 %	-Number of homework messages sent in-up until day-28
	**PDSS-SR-3064_SCREEN_sum**	numeric	177	0.00 %	Anxiety questionnaire, self rated-Timepoint before treatment starts-Sum of the entire measure
	**MADRS-1951_SCREEN_sum**	numeric	76	0.00 %	Depression questionnaire, self rated-Timepoint before treatment starts-Sum of the entire measure
	**LSAS-2241_SCREEN_sum**	numeric	340	0.00 %	Social anxiety questionnaire, self rated-Timepoint before treatment starts-Sum of the entire measure
	**MADRS-1951_SCREEN_DateCompleted_day**	numeric	20	0.00 %	Depression questionnaire, self rated-Timepoint before treatment starts-Cyclic transformation of what day 0-6 during week it was filled in

Label	Variable	Class	# unique values	Missing	Description
	**MADRS-1951_SCREEN_DateCompleted_time**	numeric	738	0.00 %	Depression questionnaire, self rated-Timepoint before treatment starts-Cyclic transformation of what time during day 0-1440 it was filled in
	**PDSS-SR-3064_SCREEN_DateCompleted_day**	numeric	39	0.00 %	Anxiety questionnaire, self rated-Timepoint before treatment starts-Cyclic transformation of what day 0-6 during week it was filled in
	**PDSS-SR-3064_SCREEN_DateCompleted_time**	numeric	853	0.00 %	Anxiety questionnaire, self rated-Timepoint before treatment starts-Cyclic transformation of what time during day 0-1440 it was filled in
	**LSAS-2241_SCREEN_DateCompleted_day**	numeric	65	0.00 %	Social anxiety questionnaire, self rated-Timepoint before treatment starts-Cyclic transformation of what day 0-6 during week it was filled in
	**LSAS-2241_SCREEN_DateCompleted_time**	numeric	902	0.00 %	Social anxiety questionnaire, self rated-Timepoint before treatment starts-Cyclic transformation of what time during day 0-1440 it was filled in
	**outcome**	numeric	420	0.00 %	
	**ncomorbid**	numeric	73	0.00 %	
	**HW-01**	numeric	1562	0.00 %	
	**currentwork_proff**	factor	25	0.00 %	Currently in work for trained proffession
	**Marital_1833_gift**	factor	2	0.00 %	Marital status: Married or not
	**Marital_1833_separerad**	factor	2	0.00 %	Marital status: divocered/equivalent
	**Marital_1833_singel**	factor	2	0.00 %	Marital status: single
	**Edu_1843_2**	factor	2	0.00 %	7-9 years education
	**Edu_1843_3**	factor	2	0.00 %	Uncompleted upper secondary school
	**Edu_1843_4**	factor	2	0.00 %	Higher vocational education
	**Edu_1843_5**	factor	2	0.00 %	Completed upper secondary school
	**Edu_1843_6**	factor	2	0.00 %	Uncompleted university degree
	**Edu_1843_7**	factor	2	0.00 %	University degree
	**cscale**	numeric	216	0.00 %	
	**mainsymptom_PRE_sum**	numeric	70	0.00 %	PDSS-SR for panic, MADRS for depression, LSAS for social anxiety-Timepoint just before beginning treatment-Sum of the entire measure

Label	Variable	Class	# unique values	Missing	Description
	**mainsymptom_PRE_duration**	numeric	714	0.00 %	PDSS-SR for panic, MADRS for depression, LSAS for social anxiety-Timepoint just before beginning treatment-Time to fill in measure/questionnaire
	**mainsymptom_PRE_DateCompleted_day**	numeric	47	0.00 %	PDSS-SR for panic, MADRS for depression, LSAS for social anxiety-Timepoint just before beginning treatment-Cyclic transformation of what day 0-6 during week it was filled in
	**mainsymptom_PRE_DateCompleted_time**	numeric	752	0.00 %	PDSS-SR for panic, MADRS for depression, LSAS for social anxiety-Timepoint just before beginning treatment-Cyclic transformation of what time during day 0-1440 it was filled in
	**mainsymptom_WEEK01_sum**	numeric	337	0.00 %	PDSS-SR for panic, MADRS for depression, LSAS for social anxiety-Timepoint after one week in treatment-Sum of the entire measure
	**mainsymptom_WEEK01_duration**	numeric	730	0.00 %	PDSS-SR for panic, MADRS for depression, LSAS for social anxiety-Timepoint after one week in treatment-Time to fill in measure/questionnaire
	**mainsymptom_WEEK01_DateCompleted_day**	numeric	323	0.00 %	PDSS-SR for panic, MADRS for depression, LSAS for social anxiety-Timepoint after one week in treatment-Cyclic transformation of what day 0-6 during week it was filled in
	**mainsymptom_WEEK01_DateCompleted_time**	numeric	988	0.00 %	PDSS-SR for panic, MADRS for depression, LSAS for social anxiety-Timepoint after one week in treatment-Cyclic transformation of what time during day 0-1440 it was filled in
	**mainsymptom_WEEK02_sum**	numeric	324	0.00 %	PDSS-SR for panic, MADRS for depression, LSAS for social anxiety-Timepoint after two weeks in treatment-Sum of the entire measure
	**mainsymptom_WEEK02_duration**	numeric	711	0.00 %	PDSS-SR for panic, MADRS for depression, LSAS for social anxiety-Timepoint after two weeks in treatment-Time to fill in measure/questionnaire

Label	Variable	Class	# unique values	Missing	Description
	**mainsymptom_WEEK02_DateCompleted_day**	numeric	313	0.00 %	PDSS-SR for panic, MADRS for depression, LSAS for social anxiety-Timepoint after two weeks in treatment-Cyclic transformation of what day 0-6 during week it was filled in
	**mainsymptom_WEEK02_DateCompleted_time**	numeric	973	0.00 %	PDSS-SR for panic, MADRS for depression, LSAS for social anxiety-Timepoint after two weeks in treatment-Cyclic transformation of what time during day 0-1440 it was filled in
	**mainsymptom_WEEK03_sum**	numeric	320	0.00 %	PDSS-SR for panic, MADRS for depression, LSAS for social anxiety-Timepoint after three weeks in treatment-Sum of the entire measure
	**mainsymptom_WEEK03_duration**	numeric	667	0.00 %	PDSS-SR for panic, MADRS for depression, LSAS for social anxiety-Timepoint after three weeks in treatment-Time to fill in measure/questionnaire
	**mainsymptom_WEEK03_DateCompleted_day**	numeric	303	0.00 %	PDSS-SR for panic, MADRS for depression, LSAS for social anxiety-Timepoint after three weeks in treatment-Cyclic transformation of what day 0-6 during week it was filled in
	**mainsymptom_WEEK03_DateCompleted_time**	numeric	993	0.00 %	PDSS-SR for panic, MADRS for depression, LSAS for social anxiety-Timepoint after three weeks in treatment-Cyclic transformation of what time during day 0-1440 it was filled in

Variable list

sex

Feature	Result
Variable type	factor
Number of missing obs.	0 (0 %)
Number of unique values	2
Mode	"0"
Reference category	0

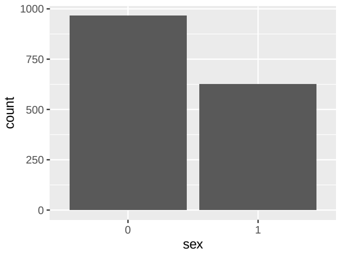

- Observed factor levels: "0", "1".

---

age

Feature	Result
Variable type	numeric
Number of missing obs.	0 (0 %)
Number of unique values	59
Median	-0.29
1st and 3rd quartiles	-0.81; 0.41
Min. and max.	-1.69; 3.55

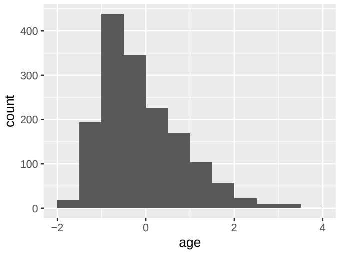

messages_len_7

Feature	Result
Variable type	numeric
Number of missing obs.	0 (0 %)
Number of unique values	350
Median	-0.32
1st and 3rd quartiles	-0.32; -0.01
Min. and max.	-0.32; 12.42

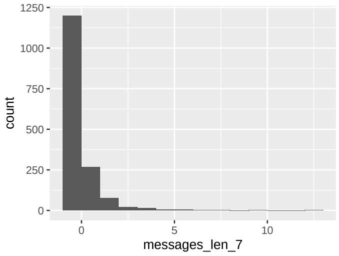

messages_len_tp_7

Feature	Result
Variable type	numeric
Number of missing obs.	0 (0 %)
Number of unique values	657
Median	-0.28
1st and 3rd quartiles	-1; 0.43
Min. and max.	-1.46; 4.08

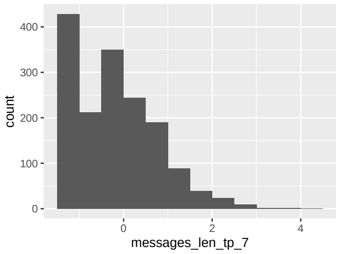

messages_7

Feature	Result
Variable type	numeric
Number of missing obs.	0 (0 %)
Number of unique values	9
Median	-0.55
1st and 3rd quartiles	-0.55; 0.58
Min. and max.	-0.55; 16.49

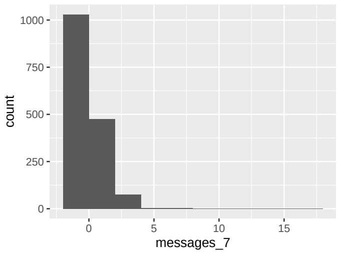

messages_tp_7

Feature	Result
Variable type	numeric
Number of missing obs.	0 (0 %)
Number of unique values	9
Median	0.15
1st and 3rd quartiles	-0.91; 0.15
Min. and max.	-1.97; 6.52

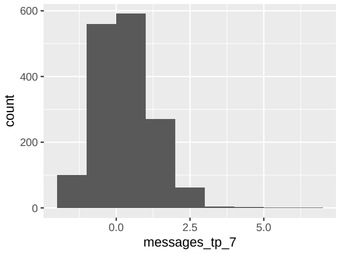

homeworks_7

Feature	Result
Variable type	numeric
Number of missing obs.	0 (0 %)
Number of unique values	7
Median	0.2
1st and 3rd quartiles	-1.03; 0.2
Min. and max.	-1.03; 6.39

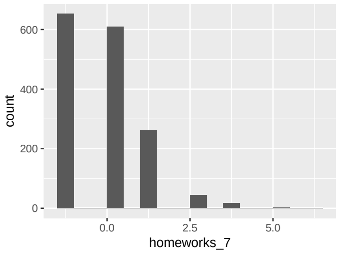

messages_len_14

Feature	Result
Variable type	numeric
Number of missing obs.	0 (0 %)
Number of unique values	371
Median	-0.41
1st and 3rd quartiles	-0.41; 0.08
Min. and max.	-0.41; 17.74

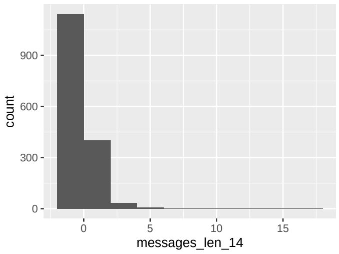

messages_len_tp_14

Feature	Result
Variable type	numeric
Number of missing obs.	0 (0 %)
Number of unique values	665
Median	-0.3
1st and 3rd quartiles	-0.78; 0.31
Min. and max.	-1.11; 7.55

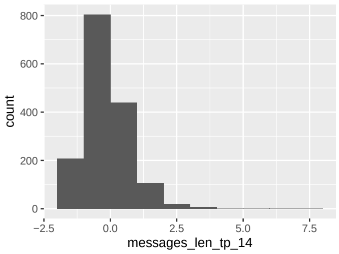

messages_14

Feature	Result
Variable type	numeric
Number of missing obs.	0 (0 %)
Number of unique values	8
Median	-0.7
1st and 3rd quartiles	-0.7; 0.36
Min. and max.	-0.7; 10.96

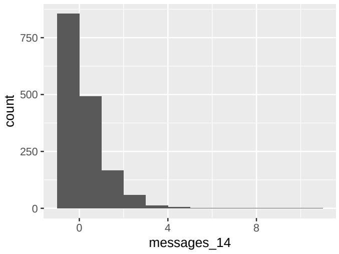

messages_tp_14

Feature	Result
Variable type	numeric
Number of missing obs.	0 (0 %)
Number of unique values	6
Median	-0.35
1st and 3rd quartiles	-0.35; 0.87
Min. and max.	-1.57; 4.53

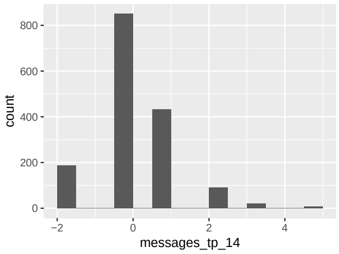

homeworks_14

Feature	Result
Variable type	numeric
Number of missing obs.	0 (0 %)
Number of unique values	5
Median	0.33
1st and 3rd quartiles	-0.98; 0.33
Min. and max.	-0.98; 4.27

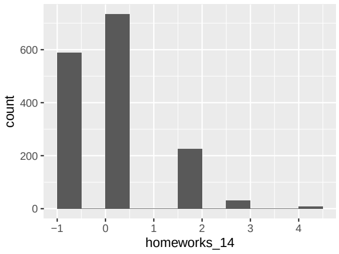

messages_len_21

Feature	Result
Variable type	numeric
Number of missing obs.	0 (0 %)
Number of unique values	353
Median	-0.46
1st and 3rd quartiles	-0.46; 0.06
Min. and max.	-0.46; 7.06

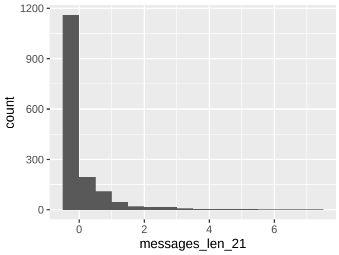

messages_len_tp_21

Feature	Result
Variable type	numeric
Number of missing obs.	0 (0 %)
Number of unique values	632
Median	-0.32
1st and 3rd quartiles	-0.8; 0.2
Min. and max.	-1.01; 8.13

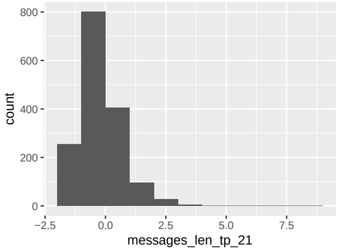

messages_21

Feature	Result
Variable type	numeric
Number of missing obs.	0 (0 %)
Number of unique values	9
Median	-0.74
1st and 3rd quartiles	-0.74; 0.34
Min. and max.	-0.74; 8.99

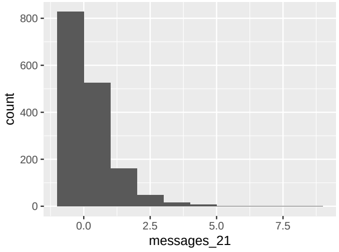

messages_tp_21

Feature	Result
Variable type	numeric
Number of missing obs.	0 (0 %)
Number of unique values	8
Median	-0.32
1st and 3rd quartiles	-0.32; 0.84
Min. and max.	-1.49; 11.31

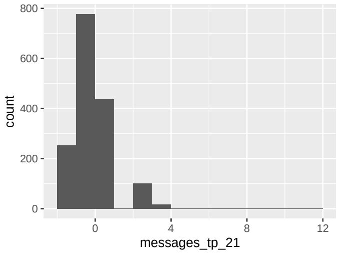

homeworks_21

Feature	Result
Variable type	numeric
Number of missing obs.	0 (0 %)
Number of unique values	6
Median	0.36
1st and 3rd quartiles	-1.01; 0.36
Min. and max.	-1.01; 5.86

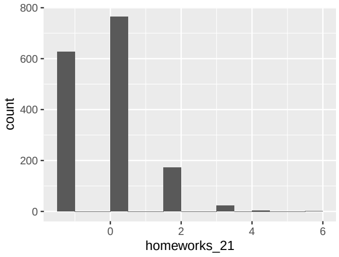

messages_len_28

Feature	Result
Variable type	numeric
Number of missing obs.	0 (0 %)
Number of unique values	339
Median	-0.41
1st and 3rd quartiles	-0.41; 0.07
Min. and max.	-0.41; 7.2

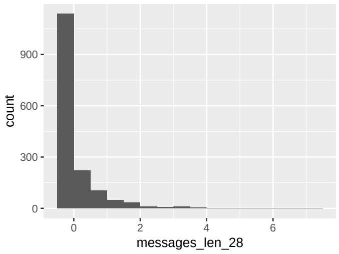

messages_len_tp_28

Feature	Result
Variable type	numeric
Number of missing obs.	0 (0 %)
Number of unique values	583
Median	-0.4
1st and 3rd quartiles	-0.81; 0.14
Min. and max.	-0.94; 6.01

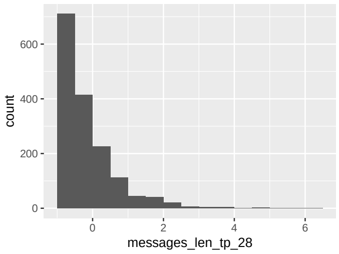

messages_28

Feature	Result
Variable type	numeric
Number of missing obs.	0 (0 %)
Number of unique values	9
Median	-0.72
1st and 3rd quartiles	-0.72; 0.41
Min. and max.	-0.72; 10.59

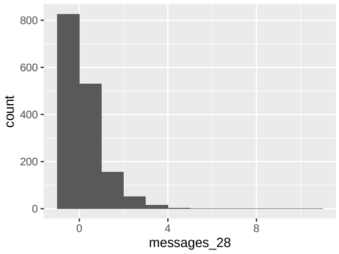

messages_tp_28

Feature	Result
Variable type	numeric
Number of missing obs.	0 (0 %)
Number of unique values	7
Median	-0.24
1st and 3rd quartiles	-0.24; 0.93
Min. and max.	-1.42; 5.63

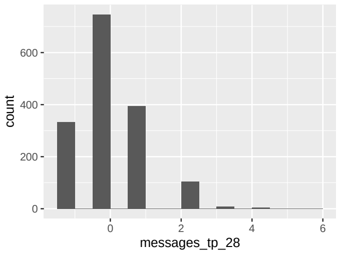

homeworks_28

Feature	Result
Variable type	numeric
Number of missing obs.	0 (0 %)
Number of unique values	6
Median	0.46
1st and 3rd quartiles	-0.91; 0.46
Min. and max.	-0.91; 5.94

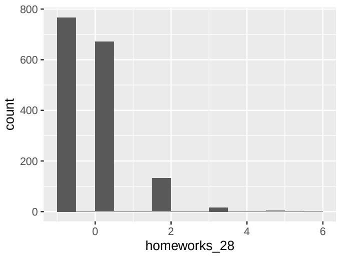

PDSS-SR-3064_SCREEN_sum

Feature	Result
Variable type	numeric
Number of missing obs.	0 (0 %)
Number of unique values	177
Median	0.75
1st and 3rd quartiles	0.28; 1.38
Min. and max.	-1.29; 3.12

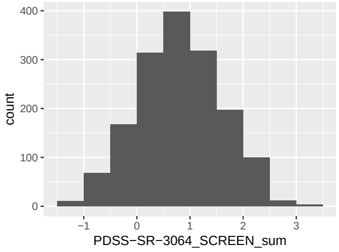

MADRS-1951_SCREEN_sum

Feature	Result
Variable type	numeric
Number of missing obs.	0 (0 %)
Number of unique values	76
Median	-0.45
1st and 3rd quartiles	-1.19; 0.17
Min. and max.	-2.68; 3.4

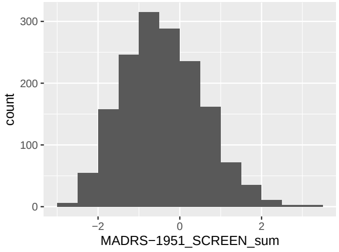

LSAS-2241_SCREEN_sum

Feature	Result
Variable type	numeric
Number of missing obs.	0 (0 %)
Number of unique values	340
Median	-0.58
1st and 3rd quartiles	-1.1; 0.08
Min. and max.	-1.79; 3.02

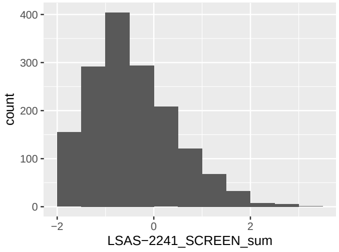

MADRS-1951_SCREEN_DateCompleted_day

Feature	Result
Variable type	numeric
Number of missing obs.	0 (0 %)
Number of unique values	20
Median	1
1st and 3rd quartiles	-0.37; 1
Min. and max.	-1.37; 1.37

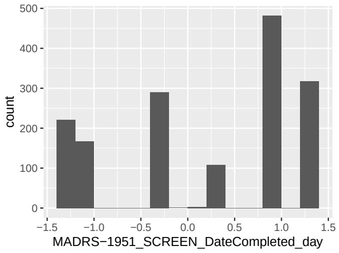

MADRS-1951_SCREEN_DateCompleted_time

Feature	Result
Variable type	numeric
Number of missing obs.	0 (0 %)
Number of unique values	738
Median	-0.77
1st and 3rd quartiles	-1.26; 0.1
Min. and max.	-1.41; 1.41

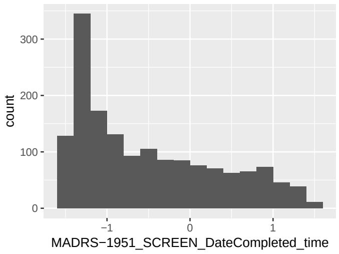

PDSS-SR-3064_SCREEN_DateCompleted_day

Feature	Result
Variable type	numeric
Number of missing obs.	0 (0 %)
Number of unique values	39
Median	1
1st and 3rd quartiles	-0.37; 1
Min. and max.	-1.37; 1.37

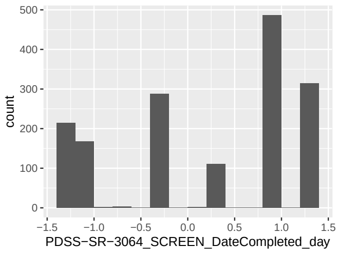

PDSS-SR-3064_SCREEN_DateCompleted_time

Feature	Result
Variable type	numeric
Number of missing obs.	0 (0 %)
Number of unique values	853
Median	-0.78
1st and 3rd quartiles	-1.26; 0.14
Min. and max.	-1.41; 1.41

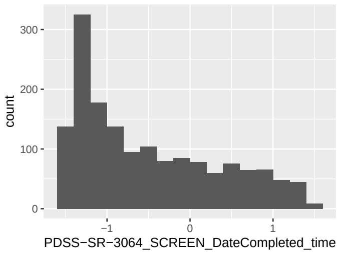

LSAS-2241_SCREEN_DateCompleted_day

Feature	Result
Variable type	numeric
Number of missing obs.	0 (0 %)
Number of unique values	65
Median	0.99
1st and 3rd quartiles	-0.38; 1
Min. and max.	-1.37; 1.37

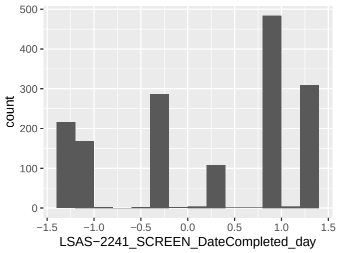

LSAS-2241_SCREEN_DateCompleted_time

Feature	Result
Variable type	numeric
Number of missing obs.	0 (0 %)
Number of unique values	902
Median	-0.77
1st and 3rd quartiles	-1.25; 0.18
Min. and max.	-1.41; 1.41

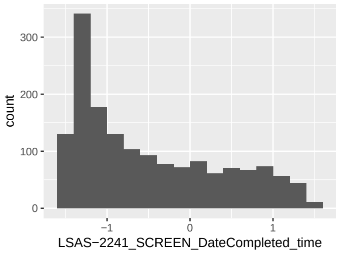

outcome

Feature	Result
Variable type	numeric
Number of missing obs.	0 (0 %)
Number of unique values	420
Median	-0.09
1st and 3rd quartiles	-0.71; 0.65
Min. and max.	-1.13; 4.66

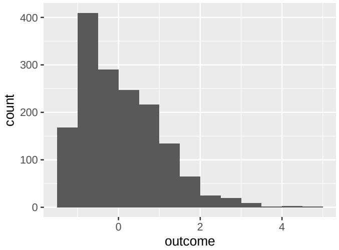

ncomorbid

Feature	Result
Variable type	numeric
Number of missing obs.	0 (0 %)
Number of unique values	73
Median	0
1st and 3rd quartiles	0; 1
Min. and max.	0; 5

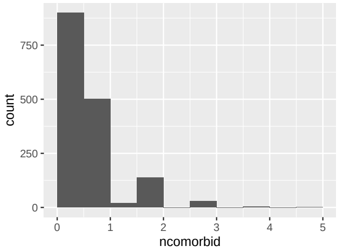

HW-01

Feature	Result
Variable type	numeric
Number of missing obs.	0 (0 %)
Number of unique values	1562
Median	0.04
1st and 3rd quartiles	-0.3; 0.38
Min. and max.	-4.65; 1.08

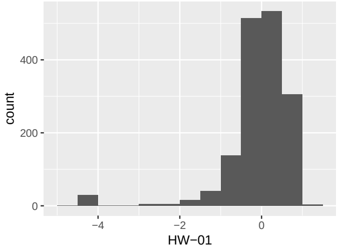

---

currentwork_proff

Feature	Result
Variable type	factor
Number of missing obs.	0 (0 %)
Number of unique values	25
Mode	"1"
Reference category	0

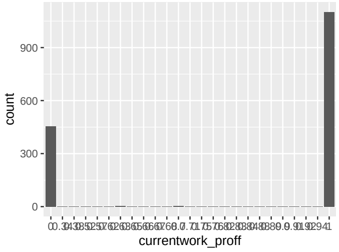

- Observed factor levels: "0", "0.34", "0.38", "0.52", "0.57", "0.62", "0.63", "0.65", "0.66", "0.67", "0.68", "0.7", "0.71", "0.75", "0.76", "0.82", "0.83", "0.84", "0.88", "0.89", "0.9", "0.91", "0.92", "0.94", "1".

Marital_1833_gift

Feature	Result
Variable type	factor
Number of missing obs.	0 (0 %)
Number of unique values	2
Mode	"1"
Reference category	0

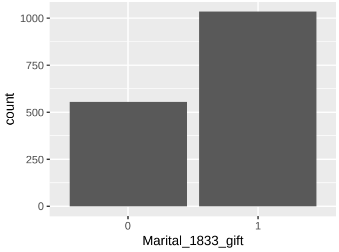

- Observed factor levels: "0", "1".

Marital_1833_separerad

Feature	Result
Variable type	factor
Number of missing obs.	0 (0 %)
Number of unique values	2
Mode	"0"
Reference category	0

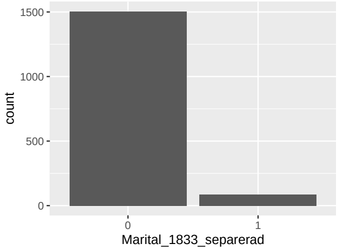

- Observed factor levels: "0", "1".

Marital_1833_singel

Feature	Result
Variable type	factor
Number of missing obs.	0 (0 %)
Number of unique values	2
Mode	“0”
Reference category	0

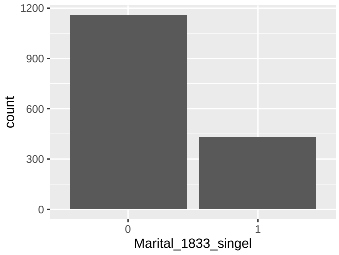

- Observed factor levels: "0", "1".

Edu_1843_2

Feature	Result
Variable type	factor
Number of missing obs.	0 (0 %)
Number of unique values	2
Mode	“0”
Reference category	0

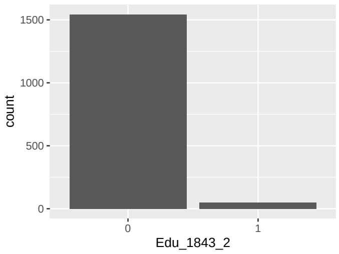

- Observed factor levels: "0", "1".

Edu_1843_3

Feature	Result
Variable type	factor
Number of missing obs.	0 (0 %)
Number of unique values	2
Mode	“0”
Reference category	0

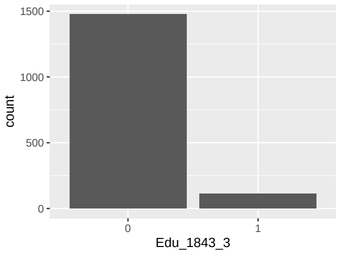

- Observed factor levels: "0", "1".

Edu_1843_4

Feature	Result
Variable type	factor
Number of missing obs.	0 (0 %)
Number of unique values	2
Mode	“0”
Reference category	0

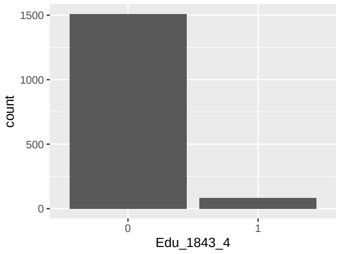

- Observed factor levels: "0", "1".

Edu_1843_5

Feature	Result
Variable type	factor
Number of missing obs.	0 (0 %)
Number of unique values	2
Mode	“0”
Reference category	0

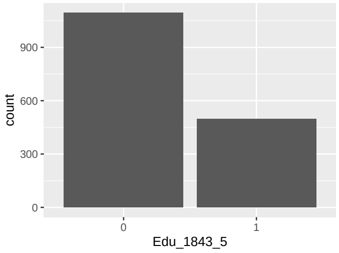

- Observed factor levels: "0", "1".

Edu_1843_6

Feature	Result
Variable type	factor
Number of missing obs.	0 (0 %)
Number of unique values	2
Mode	“0”
Reference category	0

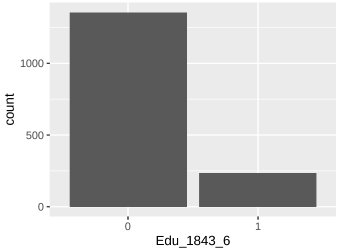

- Observed factor levels: "0", "1".

Edu_1843_7

Feature	Result
Variable type	factor
Number of missing obs.	0 (0 %)
Number of unique values	2
Mode	“0”
Reference category	0

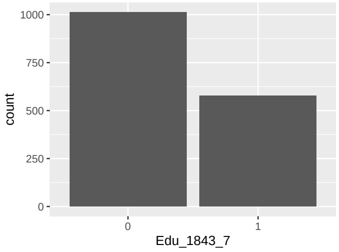

- Observed factor levels: "0", "1".

---

cscale

Feature	Result
Variable type	numeric
Number of missing obs.	0 (0 %)
Number of unique values	216
Median	0.47
1st and 3rd quartiles	-0.23; 0.96
Min. and max.	-4.14; 1.79

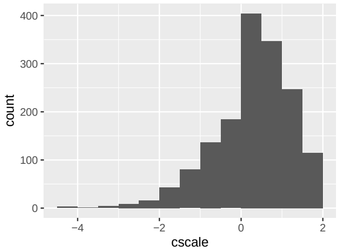

---

mainsymptom_PRE_sum

Feature	Result
Variable type	numeric
Number of missing obs.	0 (0 %)
Number of unique values	70
Median	-0.07
1st and 3rd quartiles	-0.68; 0.74
Min. and max.	-2.31; 3.39

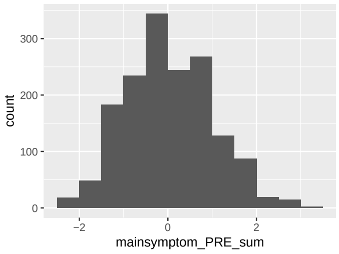

mainsymptom_PRE_duration

Feature	Result
Variable type	numeric
Number of missing obs.	0 (0 %)
Number of unique values	714
Median	-0.14
1st and 3rd quartiles	-0.2; -0.05
Min. and max.	-0.28; 19.88

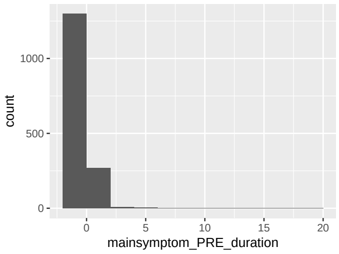

mainsymptom_PRE_DateCompleted_day

Feature	Result
Variable type	numeric
Number of missing obs.	0 (0 %)
Number of unique values	47
Median	0.37
1st and 3rd quartiles	-0.37; 1
Min. and max.	-1.37; 1.37

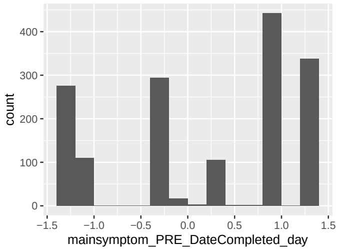

mainsymptom_PRE_DateCompleted_time

Feature	Result
Variable type	numeric
Number of missing obs.	0 (0 %)
Number of unique values	752
Median	-0.75
1st and 3rd quartiles	-1.25; 0.02
Min. and max.	-1.41; 1.41

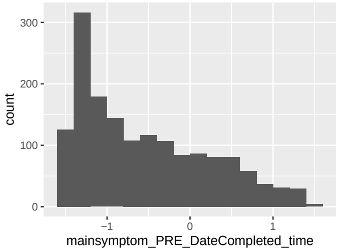

mainsymptom_WEEK01_sum

Feature	Result
Variable type	numeric
Number of missing obs.	0 (0 %)
Number of unique values	337
Median	-0.06
1st and 3rd quartiles	-0.69; 0.57
Min. and max.	-1.95; 3.51

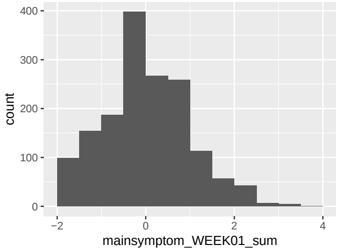

mainsymptom_WEEK01_duration

Feature	Result
Variable type	numeric
Number of missing obs.	0 (0 %)
Number of unique values	730
Median	-0.13
1st and 3rd quartiles	-0.2; 0
Min. and max.	-0.36; 27.35

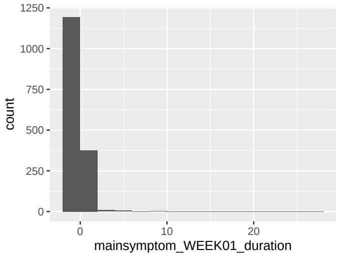

mainsymptom_WEEK01_DateCompleted_day

Feature	Result
Variable type	numeric
Number of missing obs.	0 (0 %)
Number of unique values	323
Median	0.44
1st and 3rd quartiles	-0.37; 1
Min. and max.	-1.37; 1.37

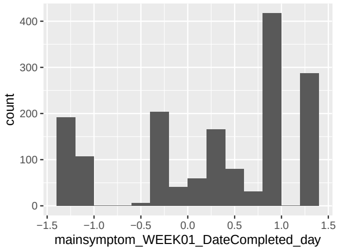

mainsymptom_WEEK01_DateCompleted_time

Feature	Result
Variable type	numeric
Number of missing obs.	0 (0 %)
Number of unique values	988
Median	-0.68
1st and 3rd quartiles	-1.13; -0.15
Min. and max.	-1.41; 1.41

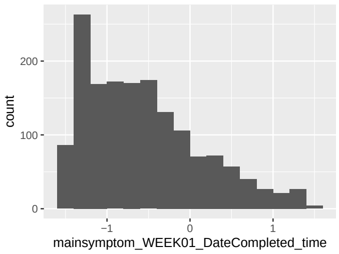

mainsymptom_WEEK02_sum

Feature	Result
Variable type	numeric
Number of missing obs.	0 (0 %)
Number of unique values	324
Median	-0.13
1st and 3rd quartiles	-0.56; 0.71
Min. and max.	-1.82; 3.86

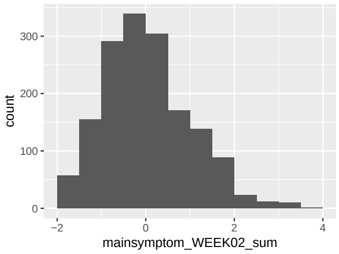

mainsymptom_WEEK02_duration

Feature	Result
Variable type	numeric
Number of missing obs.	0 (0 %)
Number of unique values	711
Median	-0.15
1st and 3rd quartiles	-0.24; 0.02
Min. and max.	-0.35; 19.53

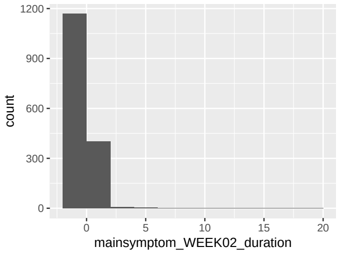

mainsymptom_WEEK02_DateCompleted_day

Feature	Result
Variable type	numeric
Number of missing obs.	0 (0 %)
Number of unique values	313
Median	0.37
1st and 3rd quartiles	-0.37; 1
Min. and max.	-1.37; 1.37

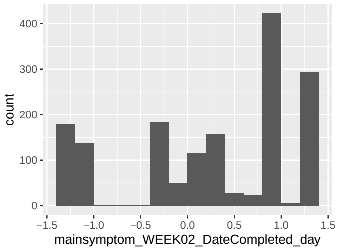

mainsymptom_WEEK02_DateCompleted_time

Feature	Result
Variable type	numeric
Number of missing obs.	0 (0 %)
Number of unique values	973
Median	-0.63
1st and 3rd quartiles	-1.07; -0.09
Min. and max.	-1.41; 1.41

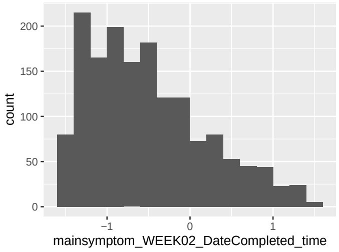

mainsymptom_WEEK03_sum

Feature	Result
Variable type	numeric
Number of missing obs.	0 (0 %)
Number of unique values	320
Median	-0.02
1st and 3rd quartiles	-0.66; 0.71
Min. and max.	-1.72; 3.99

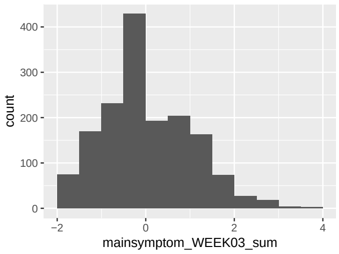

mainsymptom_WEEK03_duration

Feature	Result
Variable type	numeric
Number of missing obs.	0 (0 %)
Number of unique values	667
Median	-0.11
1st and 3rd quartiles	-0.17; -0.01
Min. and max.	-0.26; 31.82

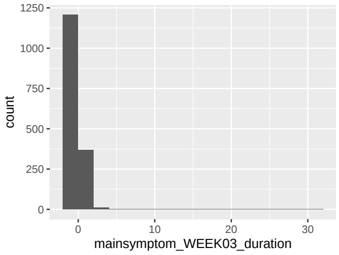

mainsymptom_WEEK03_DateCompleted_day

Feature	Result
Variable type	numeric
Number of missing obs.	0 (0 %)
Number of unique values	303
Median	0.37
1st and 3rd quartiles	-0.37; 1
Min. and max.	-1.37; 1.37

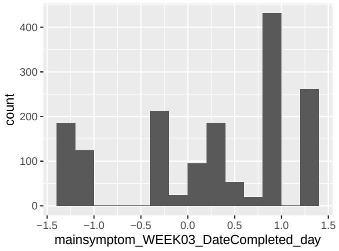

mainsymptom_WEEK03_DateCompleted_time

Feature	Result
Variable type	numeric
Number of missing obs.	0 (0 %)
Number of unique values	993
Median	-0.66
1st and 3rd quartiles	-1.1; -0.05
Min. and max.	-1.41; 1.41

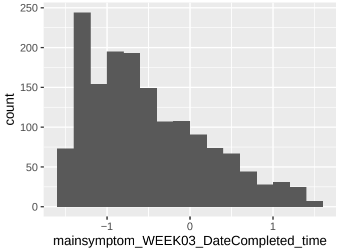

Report generation information:

- Created by: Could not determine from system (username: `nilisa`).
- Report creation time: Mon Jan 09 2023 13:07:33
- Report was run from directory: `/home/nilisa/projects/phd_study1/r`

- dataMaid v1.4.1 [Pkg: 2021-10-08 from CRAN (R 4.2.2)]
- R version 4.2.2 Patched (2022-11-10 r83330).
- Platform: x86_64-pc-linux-gnu (64-bit)(Ubuntu 20.04.5 LTS).
- Function call: `dataMaid::makeDataReport(data = gd, mode = c("summarize", "visualize",  "check"), smartNum = FALSE, file = "~/projects/data/study1multiverse/results/graphs_n_figures/codebooks/codebook` `replace = TRUE, openResult = FALSE, checks = list(character = "showAllFactorLevels", factor = "showAllFactorLevels", labelled = "showAllFactorLevels",   haven_labelled = "showAllFactorLevels", numeric = NULL,   integer = NULL, logical = NULL, Date = NULL), listChecks = FALSE, maxProbVals = Inf, codebook = TRUE, reportTitle = "Handpicked_Panic_week04-impute`

Handpicked_Panic_week04-naremove_benchmark_test

Autogenerated data summary from dataMaid

2023-01-09 13:14:36

Data report overview

The dataset examined has the following dimensions:

Feature	Result
Number of observations	88
Number of variables	10

Codebook summary table

Label	Variable	Class	# unique values	Missing	Description
	**sex**	factor	2	0.00 %	Sex of patient, 0 = Female, 1=Male
	**age**	numeric	34	0.00 %	
	**PDSS-SR-3064_SCREEN_sum**	numeric	21	0.00 %	Anxiety questionnaire, self rated-Timepoint before treatment starts-Sum of the entire measure
	**MADRS-1951_SCREEN_sum**	numeric	25	0.00 %	Depression questionnaire, self rated-Timepoint before treatment starts-Sum of the entire measure
	**LSAS-2241_SCREEN_sum**	numeric	53	0.00 %	Social anxiety questionnaire, self rated-Timepoint before treatment starts-Sum of the entire measure
	**outcome**	numeric	16	0.00 %	
	**mainsymptom_PRE_sum**	numeric	23	0.00 %	PDSS-SR for panic, MADRS for depression, LSAS for social anxiety-Timepoint just before beginning treatment-Sum of the entire measure
	**mainsymptom_WEEK01_sum**	numeric	20	0.00 %	PDSS-SR for panic, MADRS for depression, LSAS for social anxiety-Timepoint after one week in treatment-Sum of the entire measure
	**mainsymptom_WEEK02_sum**	numeric	20	0.00 %	PDSS-SR for panic, MADRS for depression, LSAS for social anxiety-Timepoint after two weeks in treatment-Sum of the entire measure

Label	Variable	Class	# unique values	Missing	Description
	**mainsymptom_WEEK03_sum**	numeric	18	0.00 %	PDSS-SR for panic, MADRS for depression, LSAS for social anxiety-Timepoint after three weeks in treatment-Sum of the entire measure

Variable list

sex

Feature	Result
Variable type	factor
Number of missing obs.	0 (0 %)
Number of unique values	2
Mode	"0"
Reference category	0

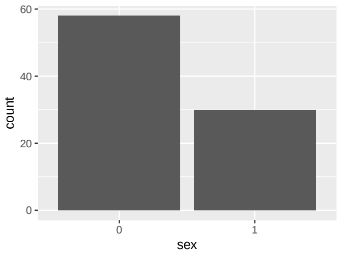

- Observed factor levels: "0", "1".

---

age

Feature	Result
Variable type	numeric
Number of missing obs.	0 (0 %)
Number of unique values	34
Median	-0.46
1st and 3rd quartiles	-1.01; 0.15
Min. and max.	-1.6; 2.51

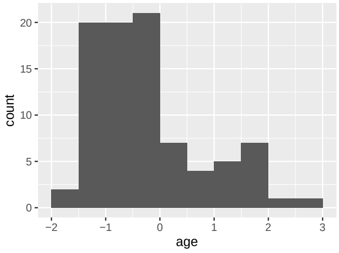

PDSS-SR-3064_SCREEN_sum

Feature	Result
Variable type	numeric
Number of missing obs.	0 (0 %)
Number of unique values	21
Median	0.91
1st and 3rd quartiles	0.44; 1.27
Min. and max.	-1.14; 2.65

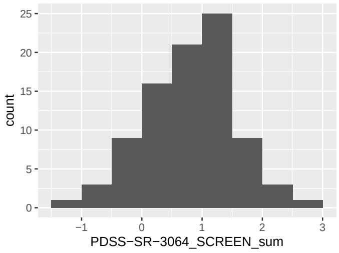

MADRS-1951_SCREEN_sum

Feature	Result
Variable type	numeric
Number of missing obs.	0 (0 %)
Number of unique values	25
Median	-0.45
1st and 3rd quartiles	-1.19; 0.05
Min. and max.	-2.43; 1.66

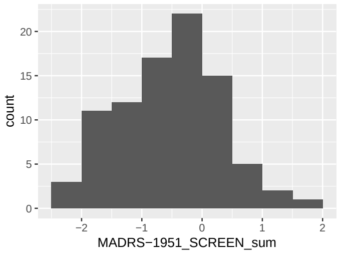

LSAS-2241_SCREEN_sum

Feature	Result
Variable type	numeric
Number of missing obs.	0 (0 %)
Number of unique values	53
Median	-0.51
1st and 3rd quartiles	-1.17; 0.23
Min. and max.	-1.79; 1.09

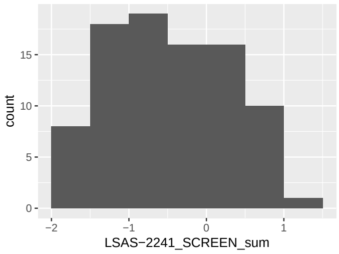

outcome

Feature	Result
Variable type	numeric
Number of missing obs.	0 (0 %)
Number of unique values	16
Median	-0.3
1st and 3rd quartiles	-0.92; 0.32
Min. and max.	-1.13; 2.8

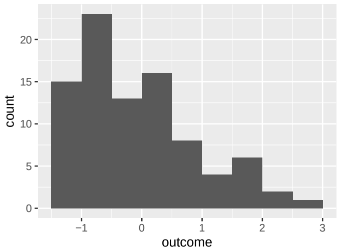

mainsymptom_PRE_sum

Feature	Result
Variable type	numeric
Number of missing obs.	0 (0 %)
Number of unique values	23
Median	-0.07
1st and 3rd quartiles	-0.68; 0.74
Min. and max.	-2.11; 2.58

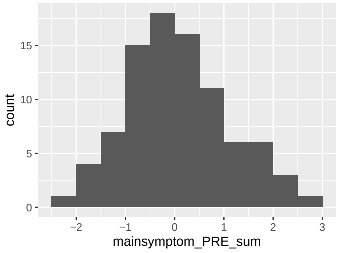

mainsymptom_WEEK01_sum

Feature	Result
Variable type	numeric
Number of missing obs.	0 (0 %)
Number of unique values	20
Median	-0.06
1st and 3rd quartiles	-0.69; 0.41
Min. and max.	-1.95; 3.3

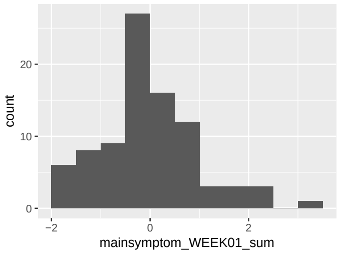

---

mainsymptom_WEEK02_sum

Feature	Result
Variable type	numeric
Number of missing obs.	0 (0 %)
Number of unique values	20
Median	-0.34
1st and 3rd quartiles	-0.77; 0.5
Min. and max.	-1.82; 3.02

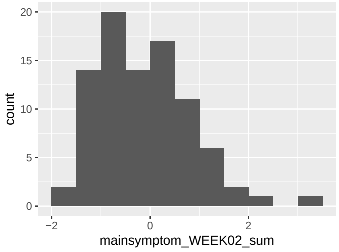

---

mainsymptom_WEEK03_sum

Feature	Result
Variable type	numeric
Number of missing obs.	0 (0 %)
Number of unique values	18
Median	-0.24
1st and 3rd quartiles	-0.87; 0.61
Min. and max.	-1.72; 2.51

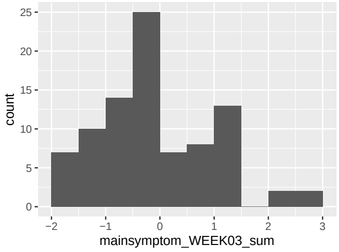

Report generation information:

- Created by: Could not determine from system (username: `nilisa`).
- Report creation time: Mon Jan 09 2023 13:14:36
- Report was run from directory: `/home/nilisa/projects/phd_study1/r`
- dataMaid v1.4.1 [Pkg: 2021-10-08 from CRAN (R 4.2.2)]
- R version 4.2.2 Patched (2022-11-10 r83330).
- Platform: x86_64-pc-linux-gnu (64-bit)(Ubuntu 20.04.5 LTS).
- Function call: `dataMaid::makeDataReport(data = gd, mode = c("summarize", "visualize", "check"), smartNum = FALSE, file = "~/projects/data/study1multiverse/results/graphs_n_figures/codebooks/codebook replace = TRUE, openResult = FALSE, checks = list(character = "showAllFactorLevels", factor = "showAllFactorLevels", labelled = "showAllFactorLevels",   haven_labelled = "showAllFactorLevels", numeric = NULL,   integer = NULL, logical = NULL, Date = NULL), listChecks = FALSE, maxProbVals = Inf, codebook = TRUE, reportTitle = "Handpicked_Panic_week04-naremo`

Handpicked_Panic_week04-naremove_benchmark_train

Autogenerated data summary from dataMaid

2023-01-09 13:08:11

Data report overview

The dataset examined has the following dimensions:

Feature	Result
Number of observations	808
Number of variables	10

Codebook summary table

Label	Variable	Class	# unique values	Missing	Description
	**sex**	factor	2	0.00 %	Sex of patient, 0 = Female, 1=Male
	**age**	numeric	54	0.00 %	
	**PDSS-SR-3064_SCREEN_sum**	numeric	26	0.00 %	Anxiety questionnaire, self rated-Timepoint before treatment starts-Sum of the entire measure
	**MADRS-1951_SCREEN_sum**	numeric	40	0.00 %	Depression questionnaire, self rated-Timepoint before treatment starts-Sum of the entire measure
	**LSAS-2241_SCREEN_sum**	numeric	113	0.00 %	Social anxiety questionnaire, self rated-Timepoint before treatment starts-Sum of the entire measure
	**outcome**	numeric	25	0.00 %	
	**mainsymptom_PRE_sum**	numeric	26	0.00 %	PDSS-SR for panic, MADRS for depression, LSAS for social anxiety-Timepoint just before beginning treatment-Sum of the entire measure
	**mainsymptom_WEEK01_sum**	numeric	26	0.00 %	PDSS-SR for panic, MADRS for depression, LSAS for social anxiety-Timepoint after one week in treatment-Sum of the entire measure
	**mainsymptom_WEEK02_sum**	numeric	25	0.00 %	PDSS-SR for panic, MADRS for depression, LSAS for social anxiety-Timepoint after two weeks in treatment-Sum of the entire measure

Label	Variable	Class	# unique values	Missing	Description
	**mainsymptom_WEEK03_sum**	numeric	25	0.00 %	PDSS-SR for panic, MADRS for depression, LSAS for social anxiety-Timepoint after three weeks in treatment-Sum of the entire measure

Variable list

sex

Feature	Result
Variable type	factor
Number of missing obs.	0 (0 %)
Number of unique values	2
Mode	"0"
Reference category	0

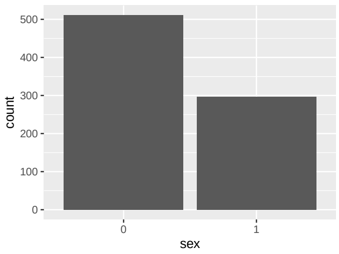

- Observed factor levels: "0", "1".

---

age

Feature	Result
Variable type	numeric
Number of missing obs.	0 (0 %)
Number of unique values	54
Median	-0.2
1st and 3rd quartiles	-0.73; 0.58
Min. and max.	-1.69; 3.55

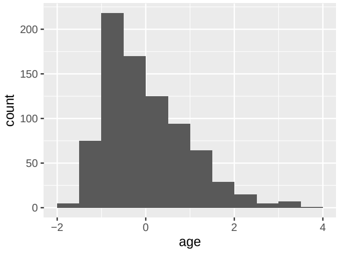

PDSS-SR-3064_SCREEN_sum

Feature	Result
Variable type	numeric
Number of missing obs.	0 (0 %)
Number of unique values	26
Median	0.75
1st and 3rd quartiles	0.28; 1.23
Min. and max.	-1.29; 3.12

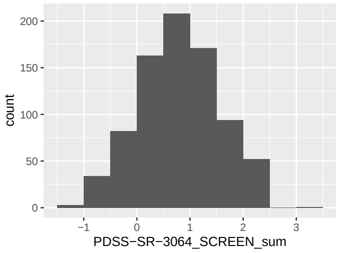

MADRS-1951_SCREEN_sum

Feature	Result
Variable type	numeric
Number of missing obs.	0 (0 %)
Number of unique values	40
Median	-0.57
1st and 3rd quartiles	-1.19; 0.17
Min. and max.	-2.68; 2.65

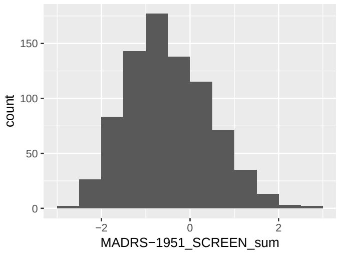

LSAS-2241_SCREEN_sum

Feature	Result
Variable type	numeric
Number of missing obs.	0 (0 %)
Number of unique values	113
Median	-0.58
1st and 3rd quartiles	-1.2; 0.12
Min. and max.	-1.79; 3.02

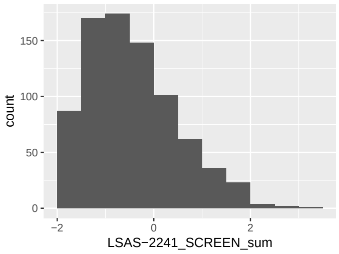

outcome

Feature	Result
Variable type	numeric
Number of missing obs.	0 (0 %)
Number of unique values	25
Median	-0.3
1st and 3rd quartiles	-0.92; 0.32
Min. and max.	-1.13; 4.04

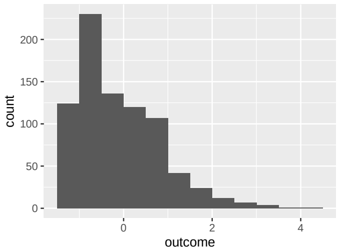

mainsymptom_PRE_sum

Feature	Result
Variable type	numeric
Number of missing obs.	0 (0 %)
Number of unique values	26
Median	-0.07
1st and 3rd quartiles	-0.68; 0.74
Min. and max.	-2.31; 2.78

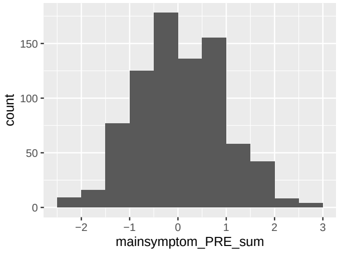

mainsymptom_WEEK01_sum

Feature	Result
Variable type	numeric
Number of missing obs.	0 (0 %)
Number of unique values	26
Median	-0.06
1st and 3rd quartiles	-0.69; 0.57
Min. and max.	-1.95; 3.51

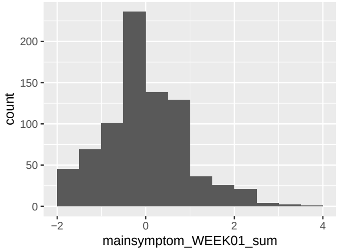

---

mainsymptom_WEEK02_sum

Feature	Result
Variable type	numeric
Number of missing obs.	0 (0 %)
Number of unique values	25
Median	-0.13
1st and 3rd quartiles	-0.77; 0.5
Min. and max.	-1.82; 3.44

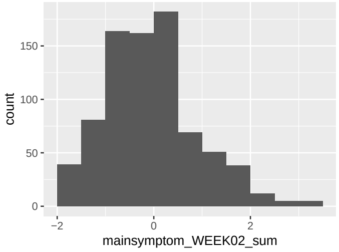

---

mainsymptom_WEEK03_sum

Feature	Result
Variable type	numeric
Number of missing obs.	0 (0 %)
Number of unique values	25
Median	-0.24
1st and 3rd quartiles	-0.87; 0.61
Min. and max.	-1.72; 3.99

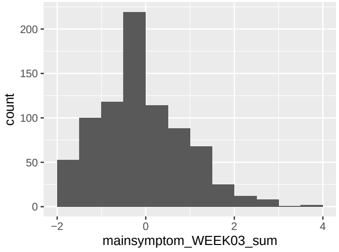

Report generation information:

- Created by: Could not determine from system (username: `nilisa`).
- Report creation time: Mon Jan 09 2023 13:08:11
- Report was run from directory: `/home/nilisa/projects/phd_study1/r`
- dataMaid v1.4.1 [Pkg: 2021-10-08 from CRAN (R 4.2.2)]
- R version 4.2.2 Patched (2022-11-10 r83330).
- Platform: x86_64-pc-linux-gnu (64-bit)(Ubuntu 20.04.5 LTS).
- Function call: `dataMaid::makeDataReport(data = gd, mode = c("summarize", "visualize", "check"), smartNum = FALSE, file = "~/projects/data/study1multiverse/results/graphs_n_figures/codebooks/codeboo` `replace = TRUE, openResult = FALSE, checks = list(character = "showAllFactorLevels", factor = "showAllFactorLevels", labelled = "showAllFactorLevels", haven_labelled = "showAllFactorLevels", numeric = NULL, integer = NULL, logical = NULL, Date = NULL), listChecks = FALSE, maxProbVals = Inf, codebook = TRUE, reportTitle = "Handpicked_Panic_week04-narem`

Handpicked_Panic_week04-naremove_test

Autogenerated data summary from dataMaid

2023-01-09 13:14:44

Data report overview

The dataset examined has the following dimensions:

Feature	Result
Number of observations	84
Number of variables	60

Codebook summary table

Label	Variable	Class	# unique values	Missing	Description
	**sex**	factor	2	0.00 %	Sex of patient, 0 = Female, 1=Male
	**age**	numeric	33	0.00 %	
	**messages_len_7**	numeric	39	0.00 %	-Meta information of messages-Length of messages-up until day-7
	**messages_len_tp_7**	numeric	76	0.00 %	-Meta information of messages-Length of messages-therapist messages-up until day-7
	**messages_7**	numeric	6	0.00 %	-Meta information of messages-up until day-7
	**messages_tp_7**	numeric	5	0.00 %	-Meta information of messages-therapist messages-up until day-7
	**homeworks_7**	numeric	4	0.00 %	-Number of homework messages sent in-up until day-7
	**messages_len_14**	numeric	45	0.00 %	-Meta information of messages-Length of messages-up until day-14
	**messages_len_tp_14**	numeric	79	0.00 %	-Meta information of messages-Length of messages-therapist messages-up until day-14
	**messages_14**	numeric	4	0.00 %	-Meta information of messages-up until day-14
	**messages_tp_14**	numeric	4	0.00 %	-Meta information of messages-therapist messages-up until day-14

Label	Variable	Class	# unique values	Missing	Description
	**homeworks_14**	numeric	5	0.00 %	-Number of homework messages sent in-up until day-14
	**messages_len_21**	numeric	42	0.00 %	-Meta information of messages-Length of messages-up until day-21
	**messages_len_tp_21**	numeric	74	0.00 %	-Meta information of messages-Length of messages-therapist messages-up until day-21
	**messages_21**	numeric	5	0.00 %	-Meta information of messages-up until day-21
	**messages_tp_21**	numeric	5	0.00 %	-Meta information of messages-therapist messages-up until day-21
	**homeworks_21**	numeric	4	0.00 %	-Number of homework messages sent in-up until day-21
	**messages_len_28**	numeric	45	0.00 %	-Meta information of messages-Length of messages-up until day-28
	**messages_len_tp_28**	numeric	75	0.00 %	-Meta information of messages-Length of messages-therapist messages-up until day-28
	**messages_28**	numeric	4	0.00 %	-Meta information of messages-up until day-28
	**messages_tp_28**	numeric	4	0.00 %	-Meta information of messages-therapist messages-up until day-28
	**homeworks_28**	numeric	4	0.00 %	-Number of homework messages sent in-up until day-28
	**PDSS-SR-3064_SCREEN_sum**	numeric	20	0.00 %	Anxiety questionnaire, self rated-Timepoint before treatment starts-Sum of the entire measure
	**MADRS-1951_SCREEN_sum**	numeric	24	0.00 %	Depression questionnaire, self rated-Timepoint before treatment starts-Sum of the entire measure
	**LSAS-2241_SCREEN_sum**	numeric	52	0.00 %	Social anxiety questionnaire, self rated-Timepoint before treatment starts-Sum of the entire measure
	**MADRS-1951_SCREEN_DateCompleted_day**	numeric	7	0.00 %	Depression questionnaire, self rated-Timepoint before treatment starts-Cyclic transformation of what day 0-6 during week it was filled in

Label	Variable	Class	# unique values	Missing	Description
	**MADRS-1951_SCREEN_DateCompleted_time**	numeric	75	0.00 %	Depression questionnaire, self rated-Timepoint before treatment starts-Cyclic transformation of what time during day 0-1440 it was filled in
	**PDSS-SR-3064_SCREEN_DateCompleted_day**	numeric	7	0.00 %	Anxiety questionnaire, self rated-Timepoint before treatment starts-Cyclic transformation of what day 0-6 during week it was filled in
	**PDSS-SR-3064_SCREEN_DateCompleted_time**	numeric	81	0.00 %	Anxiety questionnaire, self rated-Timepoint before treatment starts-Cyclic transformation of what time during day 0-1440 it was filled in
	**LSAS-2241_SCREEN_DateCompleted_day**	numeric	7	0.00 %	Social anxiety questionnaire, self rated-Timepoint before treatment starts-Cyclic transformation of what day 0-6 during week it was filled in
	**LSAS-2241_SCREEN_DateCompleted_time**	numeric	83	0.00 %	Social anxiety questionnaire, self rated-Timepoint before treatment starts-Cyclic transformation of what time during day 0-1440 it was filled in
	**outcome**	numeric	16	0.00 %	
	**ncomorbid**	numeric	4	0.00 %	
	**currentwork_proff**	factor	2	0.00 %	Currently in work for trained proffession
	**Marital_1833_gift**	factor	2	0.00 %	Marital status: Married or not
	**Marital_1833_separerad**	factor	2	0.00 %	Marital status: divocered/equivalent
	**Marital_1833_singel**	factor	2	0.00 %	Marital status: single
	**Edu_1843_2**	factor	2	0.00 %	7-9 years education
	**Edu_1843_3**	factor	2	0.00 %	Uncompleted upper secondary school
	**Edu_1843_4**	factor	2	0.00 %	Higher vocational education
	**Edu_1843_5**	factor	2	0.00 %	Completed upper secondary school
	**Edu_1843_6**	factor	2	0.00 %	Uncompleted university degree
	**Edu_1843_7**	factor	2	0.00 %	University degree
	**cscale**	numeric	27	0.00 %	
	**mainsymptom_PRE_sum**	numeric	22	0.00 %	PDSS-SR for panic, MADRS for depression, LSAS for social anxiety-Timepoint just before beginning treatment-Sum of the entire measure

Label	Variable	Class	# unique values	Missing	Description
	**mainsymptom_PRE_duration**	numeric	80	0.00 %	PDSS-SR for panic, MADRS for depression, LSAS for social anxiety-Timepoint just before beginning treatment-Time to fill in measure/questionnaire
	**mainsymptom_PRE_DateCompleted_day**	numeric	7	0.00 %	PDSS-SR for panic, MADRS for depression, LSAS for social anxiety-Timepoint just before beginning treatment-Cyclic transformation of what day 0-6 during week it was filled in
	**mainsymptom_PRE_DateCompleted_time**	numeric	76	0.00 %	PDSS-SR for panic, MADRS for depression, LSAS for social anxiety-Timepoint just before beginning treatment-Cyclic transformation of what time during day 0-1440 it was filled in
	**mainsymptom_WEEK01_sum**	numeric	20	0.00 %	PDSS-SR for panic, MADRS for depression, LSAS for social anxiety-Timepoint after one week in treatment-Sum of the entire measure
	**mainsymptom_WEEK01_duration**	numeric	74	0.00 %	PDSS-SR for panic, MADRS for depression, LSAS for social anxiety-Timepoint after one week in treatment-Time to fill in measure/questionnaire
	**mainsymptom_WEEK01_DateCompleted_day**	numeric	7	0.00 %	PDSS-SR for panic, MADRS for depression, LSAS for social anxiety-Timepoint after one week in treatment-Cyclic transformation of what day 0-6 during week it was filled in
	**mainsymptom_WEEK01_DateCompleted_time**	numeric	81	0.00 %	PDSS-SR for panic, MADRS for depression, LSAS for social anxiety-Timepoint after one week in treatment-Cyclic transformation of what time during day 0-1440 it was filled in
	**mainsymptom_WEEK02_sum**	numeric	19	0.00 %	PDSS-SR for panic, MADRS for depression, LSAS for social anxiety-Timepoint after two weeks in treatment-Sum of the entire measure
	**mainsymptom_WEEK02_duration**	numeric	73	0.00 %	PDSS-SR for panic, MADRS for depression, LSAS for social anxiety-Timepoint after two weeks in treatment-Time to fill in measure/questionnaire

Label	Variable	Class	# unique values	Missing	Description
	**mainsymptom_WEEK02_DateCompleted_day**	numeric	7	0.00 %	PDSS-SR for panic, MADRS for depression, LSAS for social anxiety-Timepoint after two weeks in treatment-Cyclic transformation of what day 0-6 during week it was filled in
	**mainsymptom_WEEK02_DateCompleted_time**	numeric	81	0.00 %	PDSS-SR for panic, MADRS for depression, LSAS for social anxiety-Timepoint after two weeks in treatment-Cyclic transformation of what time during day 0-1440 it was filled in
	**mainsymptom_WEEK03_sum**	numeric	18	0.00 %	PDSS-SR for panic, MADRS for depression, LSAS for social anxiety-Timepoint after three weeks in treatment-Sum of the entire measure
	**mainsymptom_WEEK03_duration**	numeric	68	0.00 %	PDSS-SR for panic, MADRS for depression, LSAS for social anxiety-Timepoint after three weeks in treatment-Time to fill in measure/questionnaire
	**mainsymptom_WEEK03_DateCompleted_day**	numeric	7	0.00 %	PDSS-SR for panic, MADRS for depression, LSAS for social anxiety-Timepoint after three weeks in treatment-Cyclic transformation of what day 0-6 during week it was filled in
	**mainsymptom_WEEK03_DateCompleted_time**	numeric	82	0.00 %	PDSS-SR for panic, MADRS for depression, LSAS for social anxiety-Timepoint after three weeks in treatment-Cyclic transformation of what time during day 0-1440 it was filled in

Variable list

sex

Feature	Result
Variable type	factor
Number of missing obs.	0 (0 %)
Number of unique values	2
Mode	"0"
Reference category	0

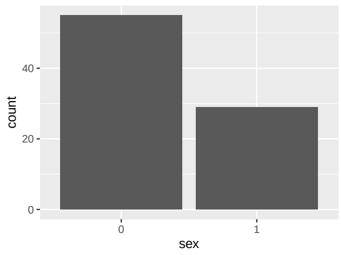

- Observed factor levels: "0", "1".

age

Feature	Result
Variable type	numeric
Number of missing obs.	0 (0 %)
Number of unique values	33
Median	-0.46
1st and 3rd quartiles	-1.01; 0.15
Min. and max.	-1.6; 2.42

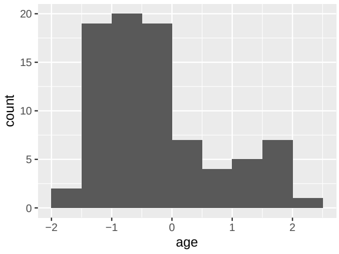

messages_len_7

Feature	Result
Variable type	numeric
Number of missing obs.	0 (0 %)
Number of unique values	39
Median	-0.32
1st and 3rd quartiles	-0.32; 0.43
Min. and max.	-0.32; 2.66

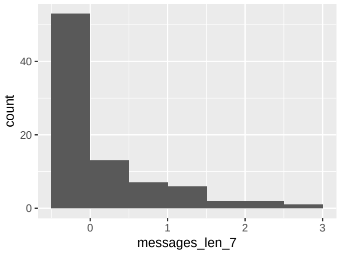

messages_len_tp_7

Feature	Result
Variable type	numeric
Number of missing obs.	0 (0 %)
Number of unique values	76
Median	0.16
1st and 3rd quartiles	-0.4; 0.7
Min. and max.	-1.14; 4.25

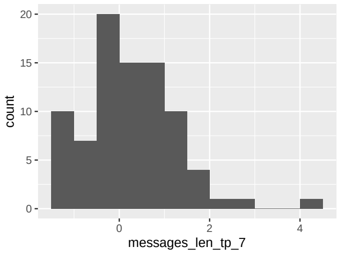

messages_7

Feature	Result
Variable type	numeric
Number of missing obs.	0 (0 %)
Number of unique values	6
Median	-0.55
1st and 3rd quartiles	-0.55; 0.58
Min. and max.	-0.55; 5.13

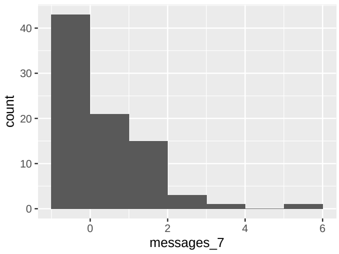

messages_tp_7

Feature	Result
Variable type	numeric
Number of missing obs.	0 (0 %)
Number of unique values	5
Median	0.15
1st and 3rd quartiles	0.15; 1.21
Min. and max.	-0.91; 3.34

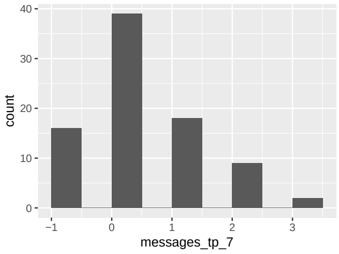

homeworks_7

Feature	Result
Variable type	numeric
Number of missing obs.	0 (0 %)
Number of unique values	4
Median	0.2
1st and 3rd quartiles	-1.03; 0.2
Min. and max.	-1.03; 2.68

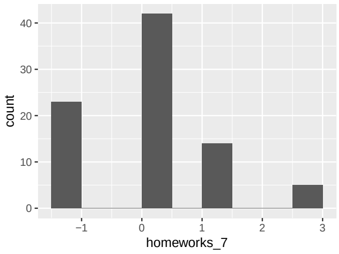

messages_len_14

Feature	Result
Variable type	numeric
Number of missing obs.	0 (0 %)
Number of unique values	45
Median	-0.25
1st and 3rd quartiles	-0.41; 0.13
Min. and max.	-0.41; 4.63

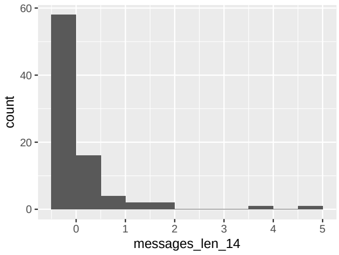

messages_len_tp_14

Feature	Result
Variable type	numeric
Number of missing obs.	0 (0 %)
Number of unique values	79
Median	-0.16
1st and 3rd quartiles	-0.5; 0.34
Min. and max.	-1.11; 4.65

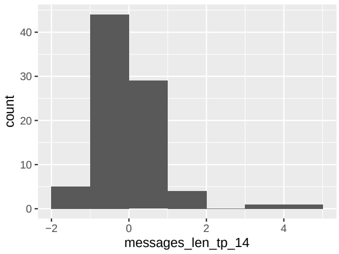

messages_14

Feature	Result
Variable type	numeric
Number of missing obs.	0 (0 %)
Number of unique values	4
Median	0.36
1st and 3rd quartiles	-0.7; 0.36
Min. and max.	-0.7; 2.48

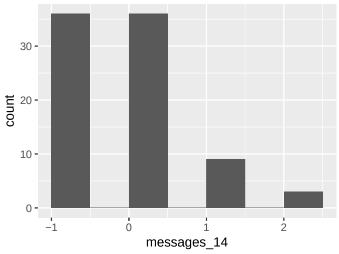

messages_tp_14

Feature	Result
Variable type	numeric
Number of missing obs.	0 (0 %)
Number of unique values	4
Median	-0.35
1st and 3rd quartiles	-0.35; 0.87
Min. and max.	-1.57; 2.09

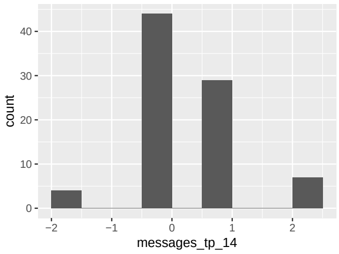

homeworks_14

Feature	Result
Variable type	numeric
Number of missing obs.	0 (0 %)
Number of unique values	5
Median	0.33
1st and 3rd quartiles	0.33; 0.33
Min. and max.	-0.98; 4.27

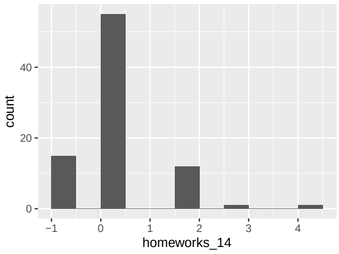

messages_len_21

Feature	Result
Variable type	numeric
Number of missing obs.	0 (0 %)
Number of unique values	42
Median	-0.45
1st and 3rd quartiles	-0.46; 0.01
Min. and max.	-0.46; 5.5

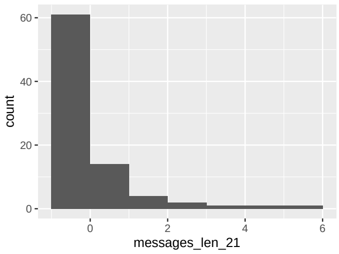

messages_len_tp_21

Feature	Result
Variable type	numeric
Number of missing obs.	0 (0 %)
Number of unique values	74
Median	-0.33
1st and 3rd quartiles	-0.75; 0.25
Min. and max.	-1.01; 5.8

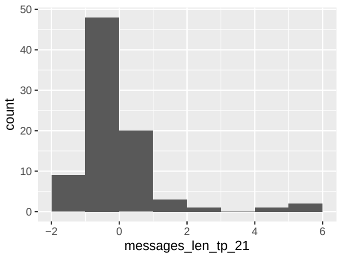

messages_21

Feature	Result
Variable type	numeric
Number of missing obs.	0 (0 %)
Number of unique values	5
Median	-0.2
1st and 3rd quartiles	-0.74; 0.34
Min. and max.	-0.74; 4.66

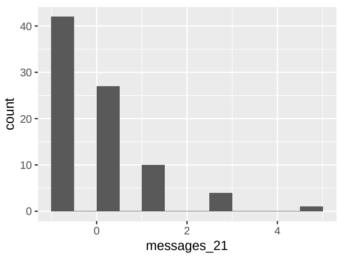

messages_tp_21

Feature	Result
Variable type	numeric
Number of missing obs.	0 (0 %)
Number of unique values	5
Median	-0.32
1st and 3rd quartiles	-0.32; 0.84
Min. and max.	-1.49; 3.17

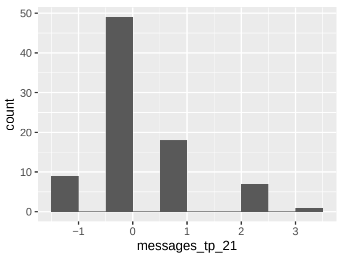

homeworks_21

Feature	Result
Variable type	numeric
Number of missing obs.	0 (0 %)
Number of unique values	4
Median	0.36
1st and 3rd quartiles	0.02; 0.36
Min. and max.	-1.01; 3.11

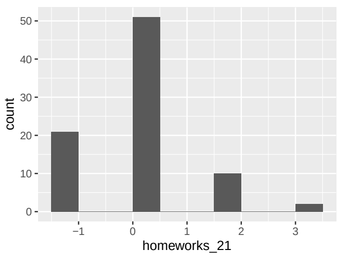

messages_len_28

Feature	Result
Variable type	numeric
Number of missing obs.	0 (0 %)
Number of unique values	45
Median	-0.24
1st and 3rd quartiles	-0.41; 0.08
Min. and max.	-0.41; 3.53

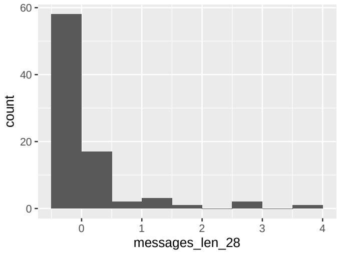

messages_len_tp_28

Feature	Result
Variable type	numeric
Number of missing obs.	0 (0 %)
Number of unique values	75
Median	-0.19
1st and 3rd quartiles	-0.71; 0.37
Min. and max.	-0.94; 3.35

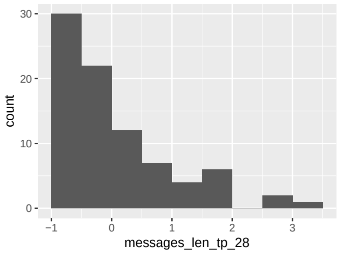

messages_28

Feature	Result
Variable type	numeric
Number of missing obs.	0 (0 %)
Number of unique values	4
Median	0.41
1st and 3rd quartiles	-0.72; 0.41
Min. and max.	-0.72; 2.67

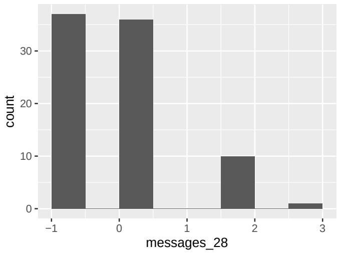

messages_tp_28

Feature	Result
Variable type	numeric
Number of missing obs.	0 (0 %)
Number of unique values	4
Median	-0.24
1st and 3rd quartiles	-0.24; 0.93
Min. and max.	-1.42; 2.1

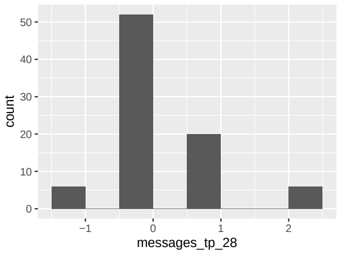

homeworks_28

Feature	Result
Variable type	numeric
Number of missing obs.	0 (0 %)
Number of unique values	4
Median	0.46
1st and 3rd quartiles	-0.91; 0.46
Min. and max.	-0.91; 3.2

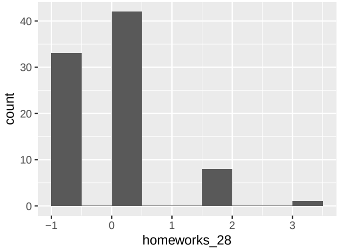

PDSS-SR-3064_SCREEN_sum

Feature	Result
Variable type	numeric
Number of missing obs.	0 (0 %)
Number of unique values	20
Median	0.91
1st and 3rd quartiles	0.44; 1.27
Min. and max.	-0.66; 2.65

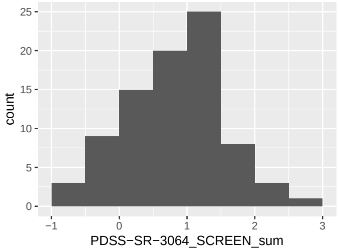

MADRS-1951_SCREEN_sum

Feature	Result
Variable type	numeric
Number of missing obs.	0 (0 %)
Number of unique values	24
Median	-0.39
1st and 3rd quartiles	-1.1; 0.05
Min. and max.	-2.18; 1.66

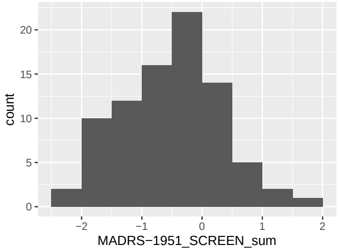

LSAS-2241_SCREEN_sum

Feature	Result
Variable type	numeric
Number of missing obs.	0 (0 %)
Number of unique values	52
Median	-0.51
1st and 3rd quartiles	-1.17; 0.26
Min. and max.	-1.79; 1.09

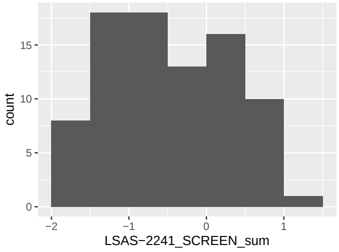

MADRS-1951_SCREEN_DateCompleted_day

Feature	Result
Variable type	numeric
Number of missing obs.	0 (0 %)
Number of unique values	7
Median	1
1st and 3rd quartiles	-0.37; 1
Min. and max.	-1.37; 1.37

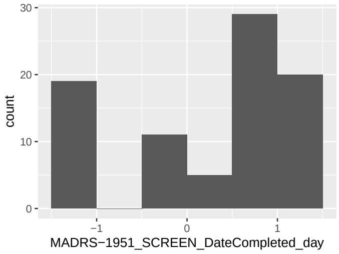

MADRS-1951_SCREEN_DateCompleted_time

Feature	Result
Variable type	numeric
Number of missing obs.	0 (0 %)
Number of unique values	75
Median	-0.82
1st and 3rd quartiles	-1.22; -0.07
Min. and max.	-1.41; 1.41

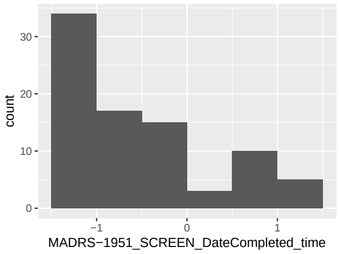

PDSS-SR-3064_SCREEN_DateCompleted_day

Feature	Result
Variable type	numeric
Number of missing obs.	0 (0 %)
Number of unique values	7
Median	1
1st and 3rd quartiles	-0.37; 1
Min. and max.	-1.37; 1.37

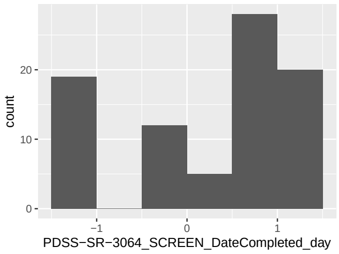

PDSS-SR-3064_SCREEN_DateCompleted_time

Feature	Result
Variable type	numeric
Number of missing obs.	0 (0 %)
Number of unique values	81
Median	-0.82
1st and 3rd quartiles	-1.22; -0.1
Min. and max.	-1.41; 1.41

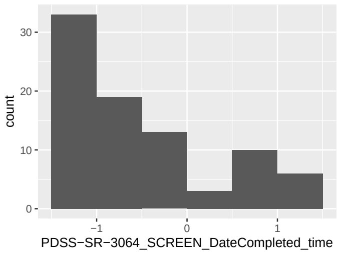

LSAS-2241_SCREEN_DateCompleted_day

Feature	Result
Variable type	numeric
Number of missing obs.	0 (0 %)
Number of unique values	7
Median	1
1st and 3rd quartiles	-0.37; 1
Min. and max.	-1.37; 1.37

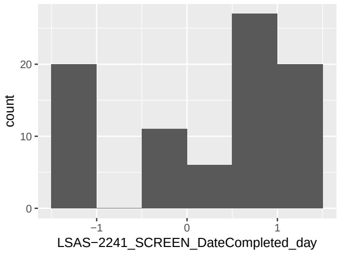

LSAS-2241_SCREEN_DateCompleted_time

Feature	Result
Variable type	numeric
Number of missing obs.	0 (0 %)
Number of unique values	83
Median	-0.81
1st and 3rd quartiles	-1.24; -0.03
Min. and max.	-1.41; 1.41

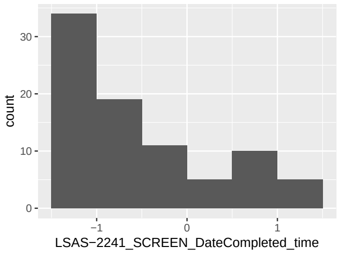

outcome

Feature	Result
Variable type	numeric
Number of missing obs.	0 (0 %)
Number of unique values	16
Median	-0.2
1st and 3rd quartiles	-0.92; 0.37
Min. and max.	-1.13; 2.8

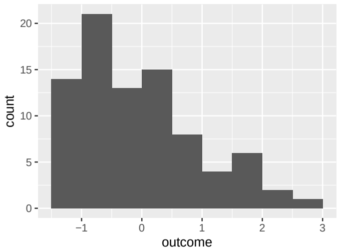

ncomorbid

Feature	Result
Variable type	numeric
Number of missing obs.	0 (0 %)
Number of unique values	4
Median	0
1st and 3rd quartiles	0; 1
Min. and max.	0; 3

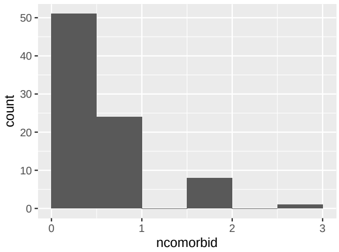

currentwork_proff

Feature	Result
Variable type	factor
Number of missing obs.	0 (0 %)
Number of unique values	2
Mode	"1"
Reference category	0

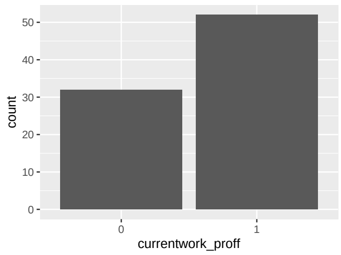

- Observed factor levels: "0", "1".

Marital_1833_gift

Feature	Result
Variable type	factor
Number of missing obs.	0 (0 %)
Number of unique values	2
Mode	"1"
Reference category	0

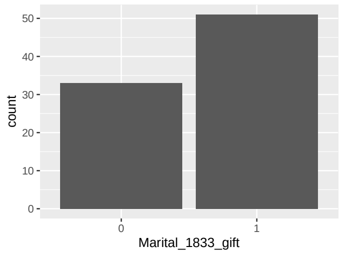

- Observed factor levels: "0", "1".

Marital_1833_separerad

Feature	Result
Variable type	factor
Number of missing obs.	0 (0 %)
Number of unique values	2
Mode	"0"
Reference category	0

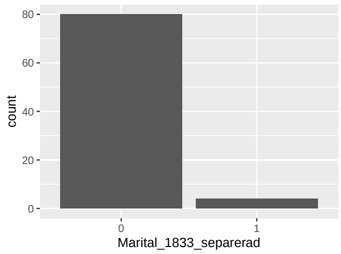

- Observed factor levels: "0", "1".

Marital_1833_singel

Feature	Result
Variable type	factor
Number of missing obs.	0 (0 %)
Number of unique values	2
Mode	"0"
Reference category	0

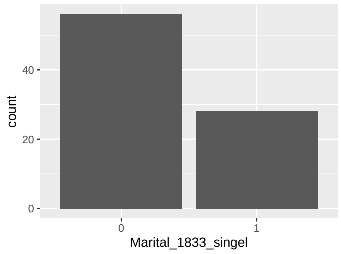

- Observed factor levels: "0", "1".

Edu_1843_2

Feature	Result
Variable type	factor
Number of missing obs.	0 (0 %)
Number of unique values	2
Mode	“0”
Reference category	0

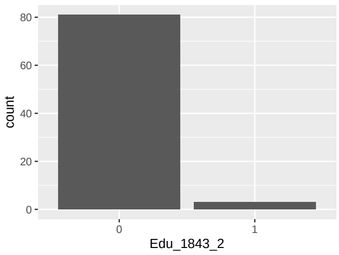

- Observed factor levels: "0", "1".

Edu_1843_3

Feature	Result
Variable type	factor
Number of missing obs.	0 (0 %)
Number of unique values	2
Mode	“0”
Reference category	0

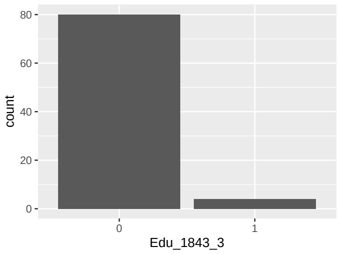

- Observed factor levels: "0", "1".

Edu_1843_4

Feature	Result
Variable type	factor
Number of missing obs.	0 (0 %)
Number of unique values	2
Mode	“0”
Reference category	0

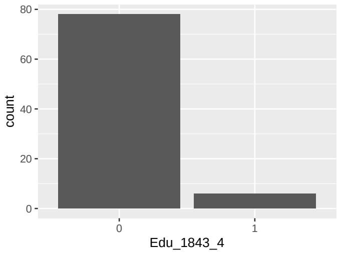

- Observed factor levels: "0", "1".

Edu_1843_5

Feature	Result
Variable type	factor
Number of missing obs.	0 (0 %)
Number of unique values	2
Mode	“0”
Reference category	0

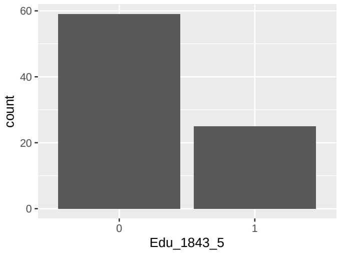

- Observed factor levels: "0", "1".

Edu_1843_6

Feature	Result
Variable type	factor
Number of missing obs.	0 (0 %)
Number of unique values	2
Mode	"0"
Reference category	0

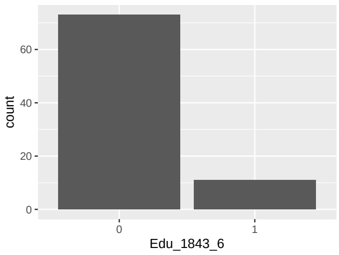

- Observed factor levels: "0", "1".

Edu_1843_7

Feature	Result
Variable type	factor
Number of missing obs.	0 (0 %)
Number of unique values	2
Mode	"0"
Reference category	0

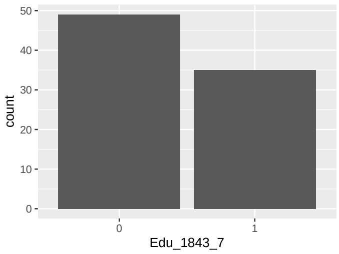

- Observed factor levels: "0", "1".

---

cscale

Feature	Result
Variable type	numeric
Number of missing obs.	0 (0 %)
Number of unique values	27
Median	0.6
1st and 3rd quartiles	-0.11; 1.11
Min. and max.	-1.65; 1.79

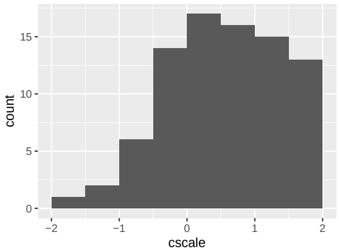

---

mainsymptom_PRE_sum

Feature	Result
Variable type	numeric
Number of missing obs.	0 (0 %)
Number of unique values	22
Median	-0.07
1st and 3rd quartiles	-0.68; 0.79
Min. and max.	-2.11; 2.58

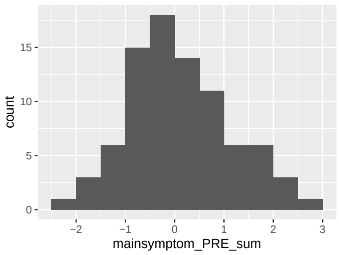

---

mainsymptom_PRE_duration

Feature	Result
Variable type	numeric
Number of missing obs.	0 (0 %)
Number of unique values	80
Median	-0.15
1st and 3rd quartiles	-0.19; -0.02
Min. and max.	-0.26; 4.74

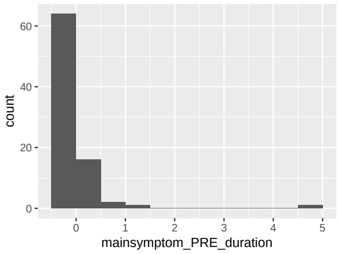

mainsymptom_PRE_DateCompleted_day

Feature	Result
Variable type	numeric
Number of missing obs.	0 (0 %)
Number of unique values	7
Median	1
1st and 3rd quartiles	-0.52; 1
Min. and max.	-1.37; 1.37

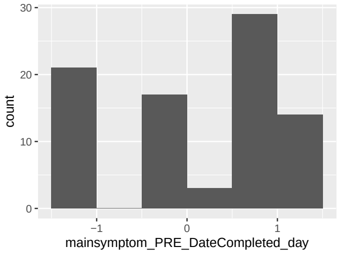

mainsymptom_PRE_DateCompleted_time

Feature	Result
Variable type	numeric
Number of missing obs.	0 (0 %)
Number of unique values	76
Median	-0.56
1st and 3rd quartiles	-1.15; 0.1
Min. and max.	-1.41; 1.41

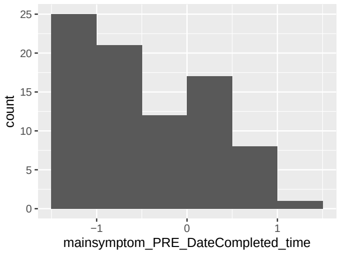

mainsymptom_WEEK01_sum

Feature	Result
Variable type	numeric
Number of missing obs.	0 (0 %)
Number of unique values	20
Median	-0.06
1st and 3rd quartiles	-0.53; 0.57
Min. and max.	-1.95; 3.3

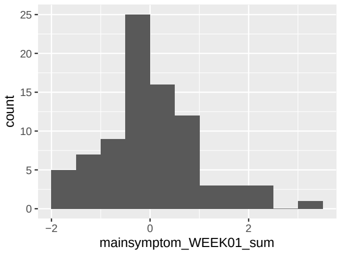

mainsymptom_WEEK01_duration

Feature	Result
Variable type	numeric
Number of missing obs.	0 (0 %)
Number of unique values	74
Median	-0.12
1st and 3rd quartiles	-0.22; -0.01
Min. and max.	-0.3; 7.81

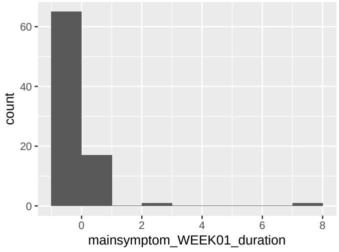

mainsymptom_WEEK01_DateCompleted_day

Feature	Result
Variable type	numeric
Number of missing obs.	0 (0 %)
Number of unique values	7
Median	0.68
1st and 3rd quartiles	-1; 1
Min. and max.	-1.37; 1.37

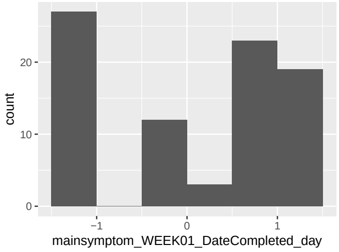

mainsymptom_WEEK01_DateCompleted_time

Feature	Result
Variable type	numeric
Number of missing obs.	0 (0 %)
Number of unique values	81
Median	-0.7
1st and 3rd quartiles	-1.3; 0.06
Min. and max.	-1.41; 1.39

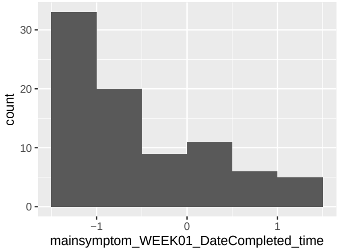

mainsymptom_WEEK02_sum

Feature	Result
Variable type	numeric
Number of missing obs.	0 (0 %)
Number of unique values	19
Median	-0.24
1st and 3rd quartiles	-0.77; 0.55
Min. and max.	-1.61; 3.02

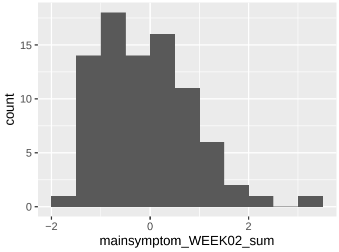

mainsymptom_WEEK02_duration

Feature	Result
Variable type	numeric
Number of missing obs.	0 (0 %)
Number of unique values	73
Median	-0.19
1st and 3rd quartiles	-0.24; -0.06
Min. and max.	-0.35; 3.7

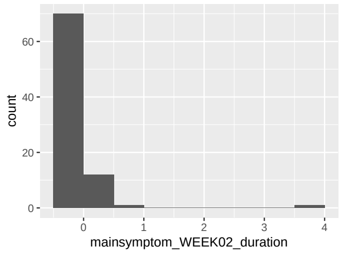

mainsymptom_WEEK02_DateCompleted_day

Feature	Result
Variable type	numeric
Number of missing obs.	0 (0 %)
Number of unique values	7
Median	1
1st and 3rd quartiles	-1; 1
Min. and max.	-1.37; 1.37

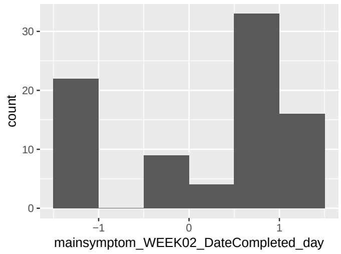

mainsymptom_WEEK02_DateCompleted_time

Feature	Result
Variable type	numeric
Number of missing obs.	0 (0 %)
Number of unique values	81
Median	-0.9
1st and 3rd quartiles	-1.32; -0.27
Min. and max.	-1.41; 1.4

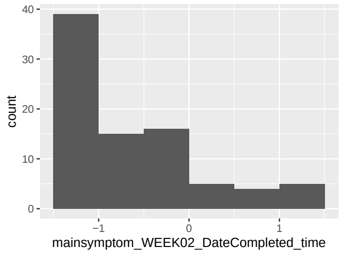

mainsymptom_WEEK03_sum

Feature	Result
Variable type	numeric
Number of missing obs.	0 (0 %)
Number of unique values	18
Median	-0.24
1st and 3rd quartiles	-0.71; 0.61
Min. and max.	-1.72; 2.51

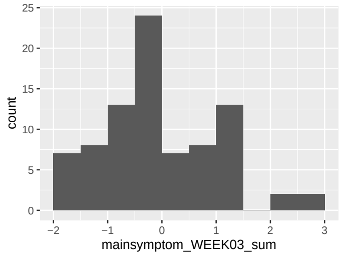

mainsymptom_WEEK03_duration

Feature	Result
Variable type	numeric
Number of missing obs.	0 (0 %)
Number of unique values	68
Median	-0.14
1st and 3rd quartiles	-0.18; -0.05
Min. and max.	-0.27; 1.09

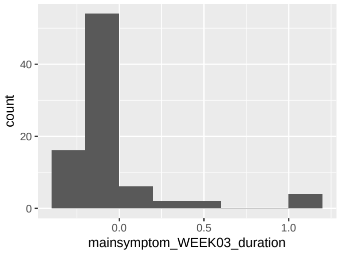

mainsymptom_WEEK03_DateCompleted_day

Feature	Result
Variable type	numeric
Number of missing obs.	0 (0 %)
Number of unique values	7
Median	1
1st and 3rd quartiles	-1; 1
Min. and max.	-1.37; 1.37

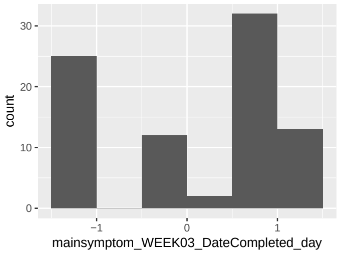

mainsymptom_WEEK03_DateCompleted_time

Feature	Result
Variable type	numeric
Number of missing obs.	0 (0 %)
Number of unique values	82
Median	-0.88
1st and 3rd quartiles	-1.25; -0.16
Min. and max.	-1.41; 1.28

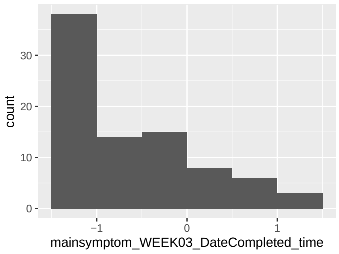

Report generation information:

- Created by: Could not determine from system (username: `nilisa`).
- Report creation time: Mon Jan 09 2023 13:14:45
- Report was run from directory: `/home/nilisa/projects/phd_study1/r`
- dataMaid v1.4.1 [Pkg: 2021-10-08 from CRAN (R 4.2.2)]
- R version 4.2.2 Patched (2022-11-10 r83330).
- Platform: x86_64-pc-linux-gnu (64-bit)(Ubuntu 20.04.5 LTS).
- Function call: `dataMaid::makeDataReport(data = gd, mode = c("summarize", "visualize",  "check"), smartNum = FALSE, file = "~/projects/data/study1multiverse/results/graphs_n_figures/codebooks/codeboo` `replace = TRUE, openResult = FALSE, checks = list(character = "showAllFactorLevels", factor = "showAllFactorLevels", labelled = "showAllFactorLevels",   haven_labelled = "showAllFactorLevels", numeric = NULL,   integer = NULL, logical = NULL, Date = NULL), listChecks = FALSE, maxProbVals = Inf, codebook = TRUE, reportTitle = "Handpicked_Panic_week04-narem`

Handpicked_Panic_week04-naremove_train

Autogenerated data summary from dataMaid

2023-01-09 13:08:18

Data report overview

The dataset examined has the following dimensions:

Feature	Result
Number of observations	782
Number of variables	60

Codebook summary table

Label	Variable	Class	# unique values	Missing	Description
	**sex**	factor	2	0.00 %	Sex of patient, 0 = Female, 1=Male
	**age**	numeric	54	0.00 %	
	**messages_len_7**	numeric	272	0.00 %	-Meta information of messages-Length of messages-up until day-7
	**messages_len_tp_7**	numeric	444	0.00 %	-Meta information of messages-Length of messages-therapist messages-up until day-7
	**messages_7**	numeric	8	0.00 %	-Meta information of messages-up until day-7
	**messages_tp_7**	numeric	8	0.00 %	-Meta information of messages-therapist messages-up until day-7
	**homeworks_7**	numeric	7	0.00 %	-Number of homework messages sent in-up until day-7
	**messages_len_14**	numeric	286	0.00 %	-Meta information of messages-Length of messages-up until day-14
	**messages_len_tp_14**	numeric	479	0.00 %	-Meta information of messages-Length of messages-therapist messages-up until day-14
	**messages_14**	numeric	6	0.00 %	-Meta information of messages-up until day-14
	**messages_tp_14**	numeric	6	0.00 %	-Meta information of messages-therapist messages-up until day-14

Label	Variable	Class	# unique values	Missing	Description
	**homeworks_14**	numeric	5	0.00 %	-Number of homework messages sent in-up until day-14
	**messages_len_21**	numeric	268	0.00 %	-Meta information of messages-Length of messages-up until day-21
	**messages_len_tp_21**	numeric	475	0.00 %	-Meta information of messages-Length of messages-therapist messages-up until day-21
	**messages_21**	numeric	8	0.00 %	-Meta information of messages-up until day-21
	**messages_tp_21**	numeric	6	0.00 %	-Meta information of messages-therapist messages-up until day-21
	**homeworks_21**	numeric	5	0.00 %	-Number of homework messages sent in-up until day-21
	**messages_len_28**	numeric	271	0.00 %	-Meta information of messages-Length of messages-up until day-28
	**messages_len_tp_28**	numeric	458	0.00 %	-Meta information of messages-Length of messages-therapist messages-up until day-28
	**messages_28**	numeric	9	0.00 %	-Meta information of messages-up until day-28
	**messages_tp_28**	numeric	7	0.00 %	-Meta information of messages-therapist messages-up until day-28
	**homeworks_28**	numeric	5	0.00 %	-Number of homework messages sent in-up until day-28
	**PDSS-SR-3064_SCREEN_sum**	numeric	26	0.00 %	Anxiety questionnaire, self rated-Timepoint before treatment starts-Sum of the entire measure
	**MADRS-1951_SCREEN_sum**	numeric	40	0.00 %	Depression questionnaire, self rated-Timepoint before treatment starts-Sum of the entire measure
	**LSAS-2241_SCREEN_sum**	numeric	113	0.00 %	Social anxiety questionnaire, self rated-Timepoint before treatment starts-Sum of the entire measure
	**MADRS-1951_SCREEN_DateCompleted_day**	numeric	7	0.00 %	Depression questionnaire, self rated-Timepoint before treatment starts-Cyclic transformation of what day 0-6 during week it was filled in

Label	Variable	Class	# unique values	Missing	Description
	**MADRS-1951_SCREEN_DateCompleted_time**	numeric	492	0.00 %	Depression questionnaire, self rated-Timepoint before treatment starts-Cyclic transformation of what time during day 0-1440 it was filled in
	**PDSS-SR-3064_SCREEN_DateCompleted_day**	numeric	7	0.00 %	Anxiety questionnaire, self rated-Timepoint before treatment starts-Cyclic transformation of what day 0-6 during week it was filled in
	**PDSS-SR-3064_SCREEN_DateCompleted_time**	numeric	497	0.00 %	Anxiety questionnaire, self rated-Timepoint before treatment starts-Cyclic transformation of what time during day 0-1440 it was filled in
	**LSAS-2241_SCREEN_DateCompleted_day**	numeric	7	0.00 %	Social anxiety questionnaire, self rated-Timepoint before treatment starts-Cyclic transformation of what day 0-6 during week it was filled in
	**LSAS-2241_SCREEN_DateCompleted_time**	numeric	489	0.00 %	Social anxiety questionnaire, self rated-Timepoint before treatment starts-Cyclic transformation of what time during day 0-1440 it was filled in
	**outcome**	numeric	25	0.00 %	
	**ncomorbid**	numeric	5	0.00 %	
	**currentwork_proff**	factor	2	0.00 %	Currently in work for trained proffession
	**Marital_1833_gift**	factor	2	0.00 %	Marital status: Married or not
	**Marital_1833_separerad**	factor	2	0.00 %	Marital status: divocered/equivalent
	**Marital_1833_singel**	factor	2	0.00 %	Marital status: single
	**Edu_1843_2**	factor	2	0.00 %	7-9 years education
	**Edu_1843_3**	factor	2	0.00 %	Uncompleted upper secondary school
	**Edu_1843_4**	factor	2	0.00 %	Higher vocational education
	**Edu_1843_5**	factor	2	0.00 %	Completed upper secondary school
	**Edu_1843_6**	factor	2	0.00 %	Uncompleted university degree
	**Edu_1843_7**	factor	2	0.00 %	University degree
	**cscale**	numeric	34	0.00 %	
	**mainsymptom_PRE_sum**	numeric	26	0.00 %	PDSS-SR for panic, MADRS for depression, LSAS for social anxiety-Timepoint just before beginning treatment-Sum of the entire measure

Label	Variable	Class	# unique values	Missing	Description
	**mainsymptom_PRE_duration**	numeric	481	0.00 %	PDSS-SR for panic, MADRS for depression, LSAS for social anxiety-Timepoint just before beginning treatment-Time to fill in measure/questionnaire
	**mainsymptom_PRE_DateCompleted_day**	numeric	7	0.00 %	PDSS-SR for panic, MADRS for depression, LSAS for social anxiety-Timepoint just before beginning treatment-Cyclic transformation of what day 0-6 during week it was filled in
	**mainsymptom_PRE_DateCompleted_time**	numeric	499	0.00 %	PDSS-SR for panic, MADRS for depression, LSAS for social anxiety-Timepoint just before beginning treatment-Cyclic transformation of what time during day 0-1440 it was filled in
	**mainsymptom_WEEK01_sum**	numeric	26	0.00 %	PDSS-SR for panic, MADRS for depression, LSAS for social anxiety-Timepoint after one week in treatment-Sum of the entire measure
	**mainsymptom_WEEK01_duration**	numeric	339	0.00 %	PDSS-SR for panic, MADRS for depression, LSAS for social anxiety-Timepoint after one week in treatment-Time to fill in measure/questionnaire
	**mainsymptom_WEEK01_DateCompleted_day**	numeric	7	0.00 %	PDSS-SR for panic, MADRS for depression, LSAS for social anxiety-Timepoint after one week in treatment-Cyclic transformation of what day 0-6 during week it was filled in
	**mainsymptom_WEEK01_DateCompleted_time**	numeric	490	0.00 %	PDSS-SR for panic, MADRS for depression, LSAS for social anxiety-Timepoint after one week in treatment-Cyclic transformation of what time during day 0-1440 it was filled in
	**mainsymptom_WEEK02_sum**	numeric	25	0.00 %	PDSS-SR for panic, MADRS for depression, LSAS for social anxiety-Timepoint after two weeks in treatment-Sum of the entire measure
	**mainsymptom_WEEK02_duration**	numeric	318	0.00 %	PDSS-SR for panic, MADRS for depression, LSAS for social anxiety-Timepoint after two weeks in treatment-Time to fill in measure/questionnaire

Label	Variable	Class	# unique values	Missing	Description
	**mainsymptom_WEEK02_DateCompleted_day**	numeric	7	0.00 %	PDSS-SR for panic, MADRS for depression, LSAS for social anxiety-Timepoint after two weeks in treatment-Cyclic transformation of what day 0-6 during week it was filled in
	**mainsymptom_WEEK02_DateCompleted_time**	numeric	493	0.00 %	PDSS-SR for panic, MADRS for depression, LSAS for social anxiety-Timepoint after two weeks in treatment-Cyclic transformation of what time during day 0-1440 it was filled in
	**mainsymptom_WEEK03_sum**	numeric	25	0.00 %	PDSS-SR for panic, MADRS for depression, LSAS for social anxiety-Timepoint after three weeks in treatment-Sum of the entire measure
	**mainsymptom_WEEK03_duration**	numeric	298	0.00 %	PDSS-SR for panic, MADRS for depression, LSAS for social anxiety-Timepoint after three weeks in treatment-Time to fill in measure/questionnaire
	**mainsymptom_WEEK03_DateCompleted_day**	numeric	7	0.00 %	PDSS-SR for panic, MADRS for depression, LSAS for social anxiety-Timepoint after three weeks in treatment-Cyclic transformation of what day 0-6 during week it was filled in
	**mainsymptom_WEEK03_DateCompleted_time**	numeric	516	0.00 %	PDSS-SR for panic, MADRS for depression, LSAS for social anxiety-Timepoint after three weeks in treatment-Cyclic transformation of what time during day 0-1440 it was filled in

Variable list

sex

Feature	Result
Variable type	factor
Number of missing obs.	0 (0 %)
Number of unique values	2
Mode	“0”
Reference category	0

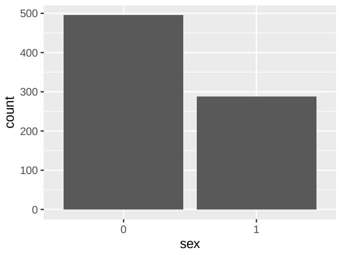

- Observed factor levels: "0", "1".

---

age

Feature	Result
Variable type	numeric
Number of missing obs.	0 (0 %)
Number of unique values	54
Median	-0.2
1st and 3rd quartiles	-0.73; 0.58
Min. and max.	-1.69; 3.55

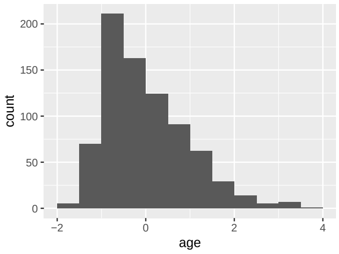

messages_len_7

Feature	Result
Variable type	numeric
Number of missing obs.	0 (0 %)
Number of unique values	272
Median	-0.32
1st and 3rd quartiles	-0.32; 0.25
Min. and max.	-0.32; 6.46

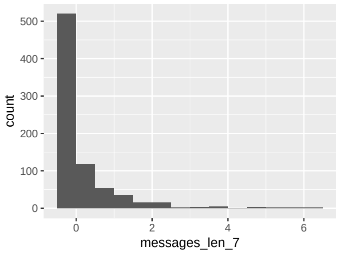

messages_len_tp_7

Feature	Result
Variable type	numeric
Number of missing obs.	0 (0 %)
Number of unique values	444
Median	0.05
1st and 3rd quartiles	-0.52; 0.69
Min. and max.	-1.46; 4.08

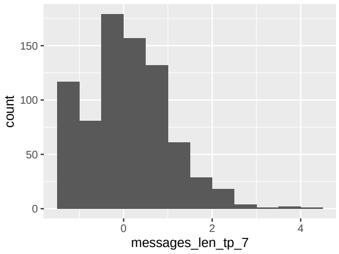

messages_7

Feature	Result
Variable type	numeric
Number of missing obs.	0 (0 %)
Number of unique values	8
Median	-0.55
1st and 3rd quartiles	-0.55; 0.58
Min. and max.	-0.55; 16.49

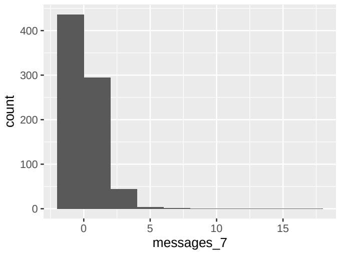

messages_tp_7

Feature	Result
Variable type	numeric
Number of missing obs.	0 (0 %)
Number of unique values	8
Median	0.15
1st and 3rd quartiles	-0.91; 1.21
Min. and max.	-1.97; 6.52

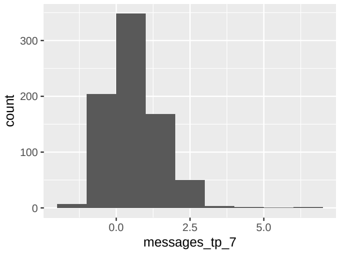

homeworks_7

Feature	Result
Variable type	numeric
Number of missing obs.	0 (0 %)
Number of unique values	7
Median	0.2
1st and 3rd quartiles	-1.03; 0.2
Min. and max.	-1.03; 6.39

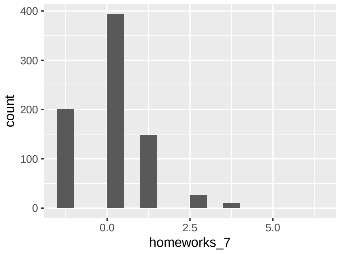

messages_len_14

Feature	Result
Variable type	numeric
Number of missing obs.	0 (0 %)
Number of unique values	286
Median	-0.27
1st and 3rd quartiles	-0.41; 0.24
Min. and max.	-0.41; 7.54

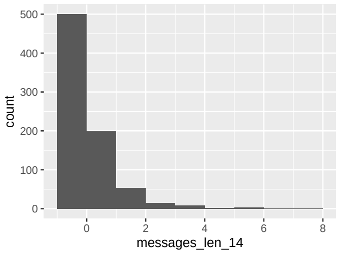

messages_len_tp_14

Feature	Result
Variable type	numeric
Number of missing obs.	0 (0 %)
Number of unique values	479
Median	-0.03
1st and 3rd quartiles	-0.53; 0.53
Min. and max.	-1.11; 7.55

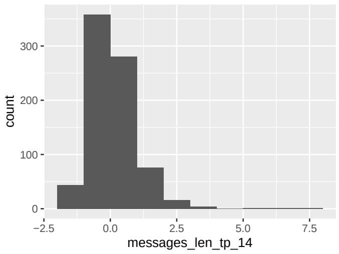

messages_14

Feature	Result
Variable type	numeric
Number of missing obs.	0 (0 %)
Number of unique values	6
Median	0.36
1st and 3rd quartiles	-0.7; 0.36
Min. and max.	-0.7; 4.6

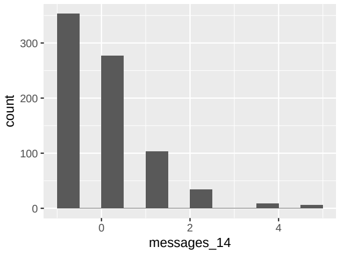

messages_tp_14

Feature	Result
Variable type	numeric
Number of missing obs.	0 (0 %)
Number of unique values	6
Median	-0.35
1st and 3rd quartiles	-0.35; 0.87
Min. and max.	-1.57; 4.53

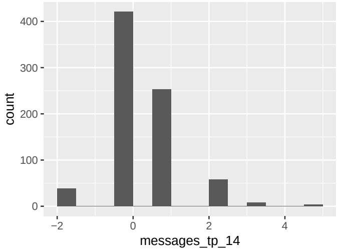

homeworks_14

Feature	Result
Variable type	numeric
Number of missing obs.	0 (0 %)
Number of unique values	5
Median	0.33
1st and 3rd quartiles	0.33; 0.33
Min. and max.	-0.98; 4.27

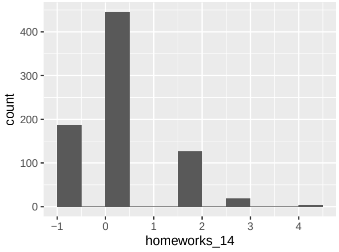

messages_len_21

Feature	Result
Variable type	numeric
Number of missing obs.	0 (0 %)
Number of unique values	268
Median	-0.24
1st and 3rd quartiles	-0.46; 0.31
Min. and max.	-0.46; 7.06

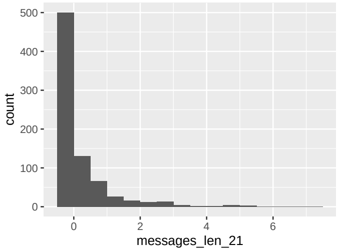

messages_len_tp_21

Feature	Result
Variable type	numeric
Number of missing obs.	0 (0 %)
Number of unique values	475
Median	-0.08
1st and 3rd quartiles	-0.47; 0.4
Min. and max.	-1.01; 8.13

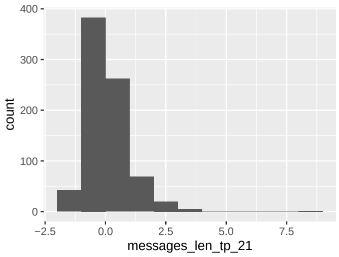

messages_21

Feature	Result
Variable type	numeric
Number of missing obs.	0 (0 %)
Number of unique values	8
Median	0.34
1st and 3rd quartiles	-0.74; 0.34
Min. and max.	-0.74; 6.83

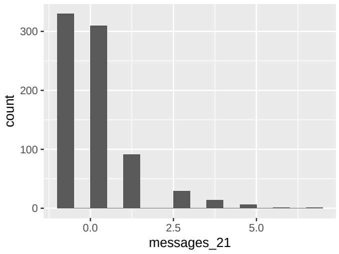

messages_tp_21

Feature	Result
Variable type	numeric
Number of missing obs.	0 (0 %)
Number of unique values	6
Median	-0.32
1st and 3rd quartiles	-0.32; 0.84
Min. and max.	-1.49; 5.5

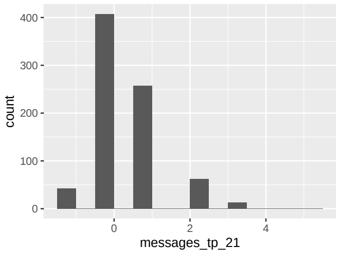

homeworks_21

Feature	Result
Variable type	numeric
Number of missing obs.	0 (0 %)
Number of unique values	5
Median	0.36
1st and 3rd quartiles	-1.01; 0.36
Min. and max.	-1.01; 4.49

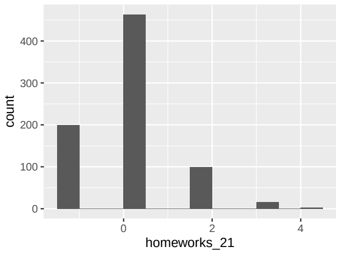

messages_len_28

Feature	Result
Variable type	numeric
Number of missing obs.	0 (0 %)
Number of unique values	271
Median	-0.24
1st and 3rd quartiles	-0.41; 0.28
Min. and max.	-0.41; 7.2

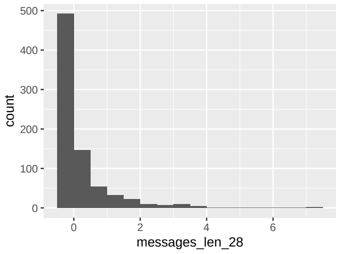

messages_len_tp_28

Feature	Result
Variable type	numeric
Number of missing obs.	0 (0 %)
Number of unique values	458
Median	-0.17
1st and 3rd quartiles	-0.57; 0.38
Min. and max.	-0.94; 4.76

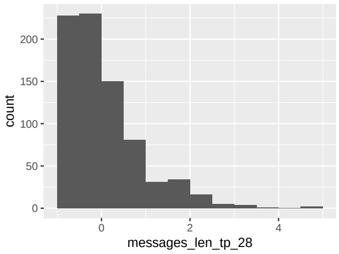

messages_28

Feature	Result
Variable type	numeric
Number of missing obs.	0 (0 %)
Number of unique values	9
Median	0.41
1st and 3rd quartiles	-0.72; 0.41
Min. and max.	-0.72; 10.59

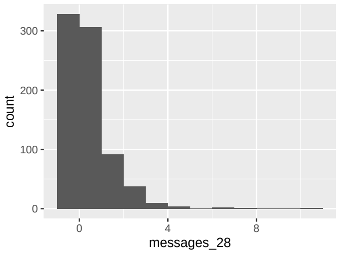

messages_tp_28

Feature	Result
Variable type	numeric
Number of missing obs.	0 (0 %)
Number of unique values	7
Median	-0.24
1st and 3rd quartiles	-0.24; 0.93
Min. and max.	-1.42; 5.63

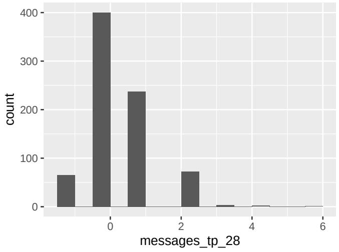

homeworks_28

Feature	Result
Variable type	numeric
Number of missing obs.	0 (0 %)
Number of unique values	5
Median	0.46
1st and 3rd quartiles	-0.91; 0.46
Min. and max.	-0.91; 4.57

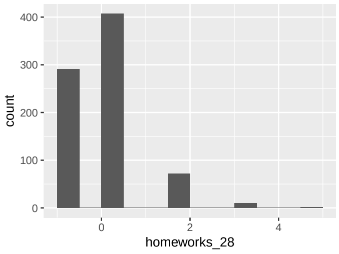

PDSS-SR-3064_SCREEN_sum

Feature	Result
Variable type	numeric
Number of missing obs.	0 (0 %)
Number of unique values	26
Median	0.75
1st and 3rd quartiles	0.28; 1.23
Min. and max.	-1.29; 3.12

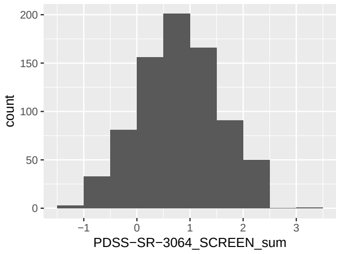

MADRS-1951_SCREEN_sum

Feature	Result
Variable type	numeric
Number of missing obs.	0 (0 %)
Number of unique values	40
Median	-0.57
1st and 3rd quartiles	-1.19; 0.17
Min. and max.	-2.68; 2.65

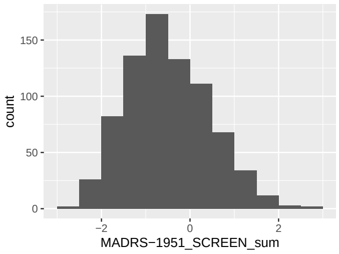

LSAS-2241_SCREEN_sum

Feature	Result
Variable type	numeric
Number of missing obs.	0 (0 %)
Number of unique values	113
Median	-0.58
1st and 3rd quartiles	-1.2; 0.12
Min. and max.	-1.79; 3.02

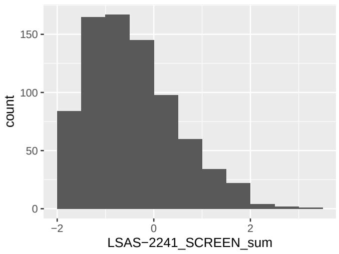

MADRS-1951_SCREEN_DateCompleted_day

Feature	Result
Variable type	numeric
Number of missing obs.	0 (0 %)
Number of unique values	7
Median	1
1st and 3rd quartiles	-1; 1
Min. and max.	-1.37; 1.37

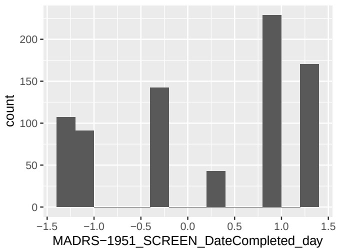

MADRS-1951_SCREEN_DateCompleted_time

Feature	Result
Variable type	numeric
Number of missing obs.	0 (0 %)
Number of unique values	492
Median	-0.82
1st and 3rd quartiles	-1.26; 0.09
Min. and max.	-1.41; 1.41

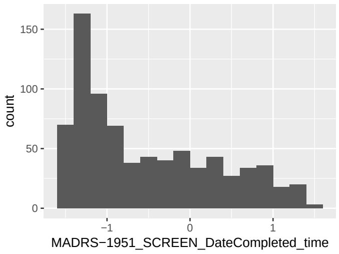

PDSS-SR-3064_SCREEN_DateCompleted_day

Feature	Result
Variable type	numeric
Number of missing obs.	0 (0 %)
Number of unique values	7
Median	1
1st and 3rd quartiles	-1; 1
Min. and max.	-1.37; 1.37

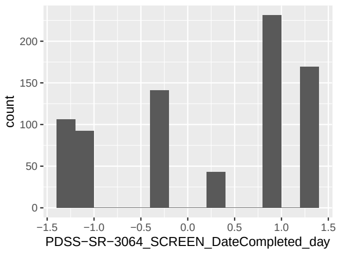

PDSS-SR-3064_SCREEN_DateCompleted_time

Feature	Result
Variable type	numeric
Number of missing obs.	0 (0 %)
Number of unique values	497
Median	-0.83
1st and 3rd quartiles	-1.26; 0.12
Min. and max.	-1.41; 1.41

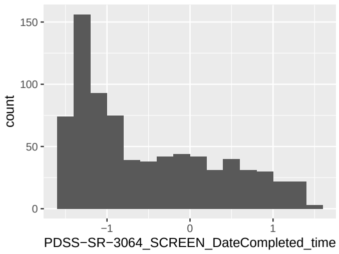

LSAS-2241_SCREEN_DateCompleted_day

Feature	Result
Variable type	numeric
Number of missing obs.	0 (0 %)
Number of unique values	7
Median	1
1st and 3rd quartiles	-0.84; 1
Min. and max.	-1.37; 1.37

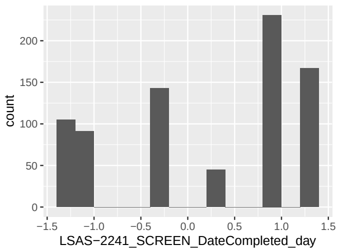

LSAS-2241_SCREEN_DateCompleted_time

Feature	Result
Variable type	numeric
Number of missing obs.	0 (0 %)
Number of unique values	489
Median	-0.82
1st and 3rd quartiles	-1.26; 0.16
Min. and max.	-1.41; 1.41

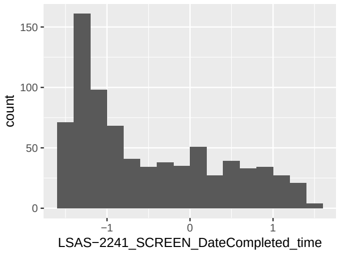

outcome

Feature	Result
Variable type	numeric
Number of missing obs.	0 (0 %)
Number of unique values	25
Median	-0.3
1st and 3rd quartiles	-0.92; 0.32
Min. and max.	-1.13; 4.04

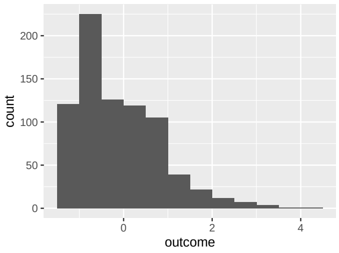

ncomorbid

Feature	Result
Variable type	numeric
Number of missing obs.	0 (0 %)
Number of unique values	5
Median	0
1st and 3rd quartiles	0; 1
Min. and max.	0; 4

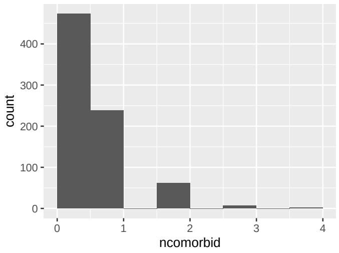

currentwork_proff

Feature	Result
Variable type	factor
Number of missing obs.	0 (0 %)
Number of unique values	2
Mode	“1”
Reference category	0

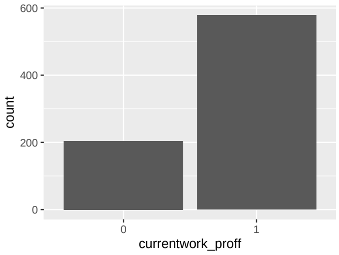

- Observed factor levels: "0", "1".

Marital_1833_gift

Feature	Result
Variable type	factor
Number of missing obs.	0 (0 %)
Number of unique values	2
Mode	“1”
Reference category	0

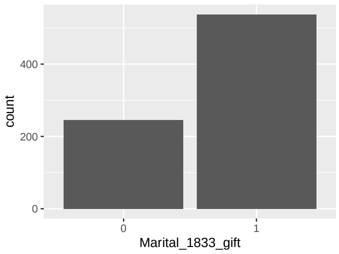

- Observed factor levels: "0", "1".

Marital_1833_separerad

Feature	Result
Variable type	factor
Number of missing obs.	0 (0 %)
Number of unique values	2
Mode	“0”
Reference category	0

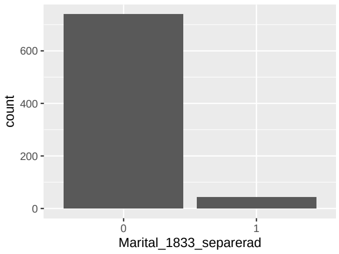

- Observed factor levels: "0", "1".

Marital_1833_singel

Feature	Result
Variable type	factor
Number of missing obs.	0 (0 %)
Number of unique values	2
Mode	“0”
Reference category	0

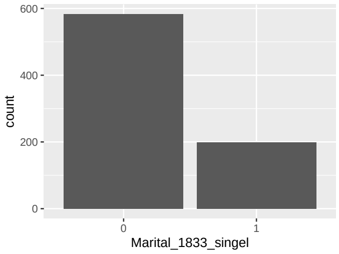

- Observed factor levels: "0", "1".

Edu_1843_2

Feature	Result
Variable type	factor
Number of missing obs.	0 (0 %)
Number of unique values	2
Mode	"0"
Reference category	0

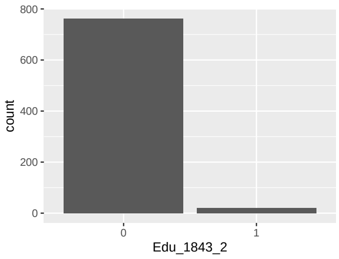

- Observed factor levels: "0", "1".

Edu_1843_3

Feature	Result
Variable type	factor
Number of missing obs.	0 (0 %)
Number of unique values	2
Mode	"0"
Reference category	0

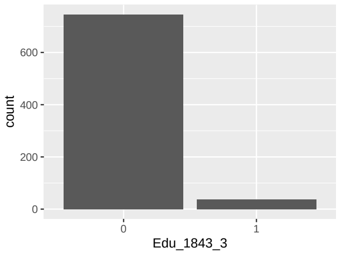

- Observed factor levels: "0", "1".

Edu_1843_4

Feature	Result
Variable type	factor
Number of missing obs.	0 (0 %)
Number of unique values	2
Mode	"0"
Reference category	0

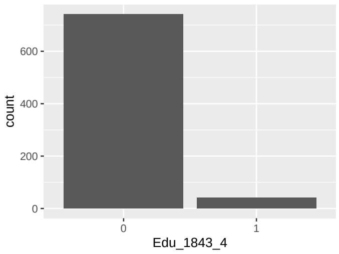

- Observed factor levels: "0", "1".

Edu_1843_5

Feature	Result
Variable type	factor
Number of missing obs.	0 (0 %)
Number of unique values	2
Mode	"0"
Reference category	0

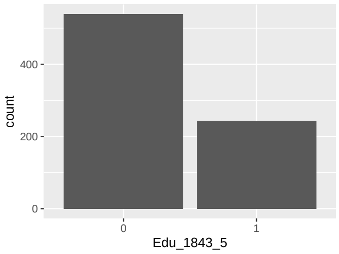

- Observed factor levels: "0", "1".

Edu_1843_6

Feature	Result
Variable type	factor
Number of missing obs.	0 (0 %)
Number of unique values	2
Mode	“0”
Reference category	0

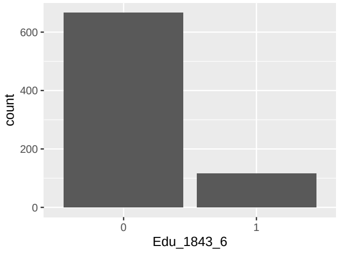

- Observed factor levels: "0", "1".

Edu_1843_7

Feature	Result
Variable type	factor
Number of missing obs.	0 (0 %)
Number of unique values	2
Mode	“0”
Reference category	0

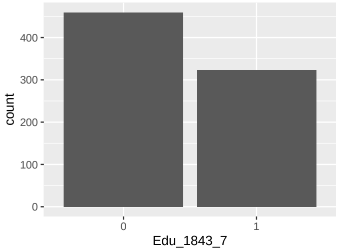

- Observed factor levels: "0", "1".

---

cscale

Feature	Result
Variable type	numeric
Number of missing obs.	0 (0 %)
Number of unique values	34
Median	0.6
1st and 3rd quartiles	0.01; 1.2
Min. and max.	-2.36; 1.79

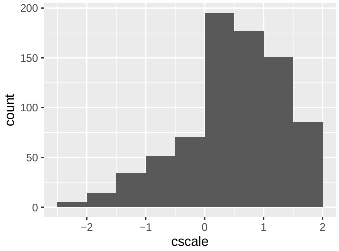

---

mainsymptom_PRE_sum

Feature	Result
Variable type	numeric
Number of missing obs.	0 (0 %)
Number of unique values	26
Median	-0.07
1st and 3rd quartiles	-0.68; 0.74
Min. and max.	-2.31; 2.78

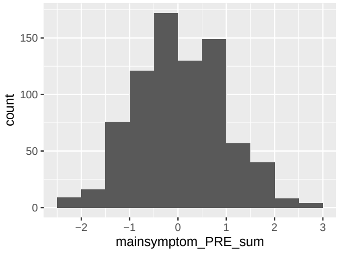

---

mainsymptom_PRE_duration

Feature	Result
Variable type	numeric
Number of missing obs.	0 (0 %)
Number of unique values	481
Median	-0.13
1st and 3rd quartiles	-0.19; -0.03
Min. and max.	-0.27; 11.15

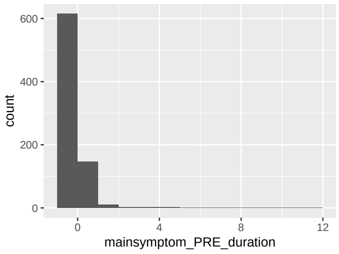

mainsymptom_PRE_DateCompleted_day

Feature	Result
Variable type	numeric
Number of missing obs.	0 (0 %)
Number of unique values	7
Median	1
1st and 3rd quartiles	-0.37; 1
Min. and max.	-1.37; 1.37

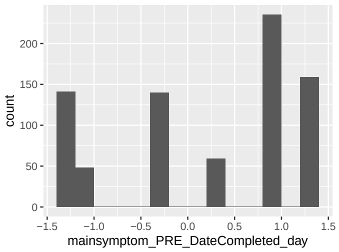

mainsymptom_PRE_DateCompleted_time

Feature	Result
Variable type	numeric
Number of missing obs.	0 (0 %)
Number of unique values	499
Median	-0.79
1st and 3rd quartiles	-1.25; -0.06
Min. and max.	-1.41; 1.41

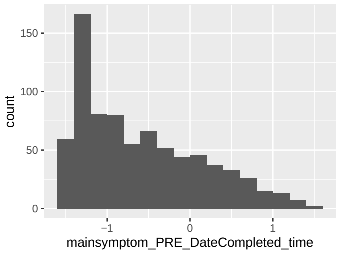

mainsymptom_WEEK01_sum

Feature	Result
Variable type	numeric
Number of missing obs.	0 (0 %)
Number of unique values	26
Median	-0.06
1st and 3rd quartiles	-0.69; 0.57
Min. and max.	-1.95; 3.51

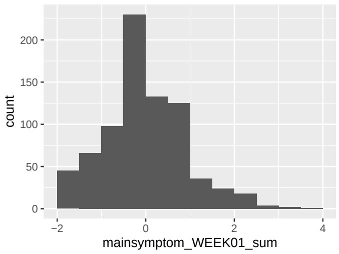

mainsymptom_WEEK01_duration

Feature	Result
Variable type	numeric
Number of missing obs.	0 (0 %)
Number of unique values	339
Median	-0.13
1st and 3rd quartiles	-0.2; 0
Min. and max.	-0.3; 10.79

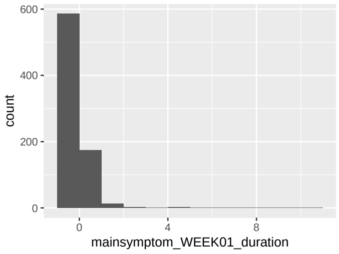

mainsymptom_WEEK01_DateCompleted_day

Feature	Result
Variable type	numeric
Number of missing obs.	0 (0 %)
Number of unique values	7
Median	1
1st and 3rd quartiles	-0.37; 1
Min. and max.	-1.37; 1.37

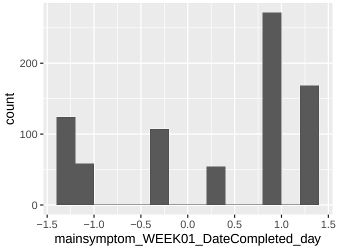

mainsymptom_WEEK01_DateCompleted_time

Feature	Result
Variable type	numeric
Number of missing obs.	0 (0 %)
Number of unique values	490
Median	-0.74
1st and 3rd quartiles	-1.24; -0.15
Min. and max.	-1.41; 1.41

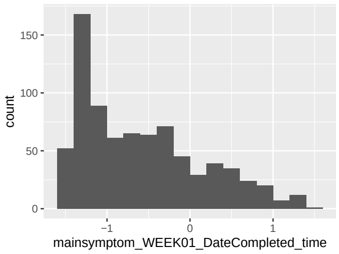

mainsymptom_WEEK02_sum

Feature	Result
Variable type	numeric
Number of missing obs.	0 (0 %)
Number of unique values	25
Median	-0.13
1st and 3rd quartiles	-0.77; 0.5
Min. and max.	-1.82; 3.44

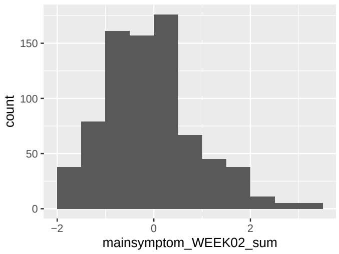

mainsymptom_WEEK02_duration

Feature	Result
Variable type	numeric
Number of missing obs.	0 (0 %)
Number of unique values	318
Median	-0.17
1st and 3rd quartiles	-0.24; -0.05
Min. and max.	-0.35; 10.58

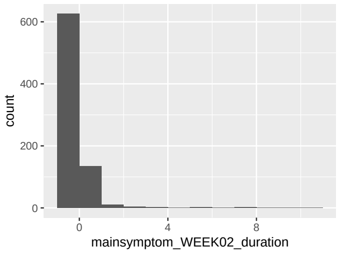

mainsymptom_WEEK02_DateCompleted_day

Feature	Result
Variable type	numeric
Number of missing obs.	0 (0 %)
Number of unique values	7
Median	1
1st and 3rd quartiles	-0.37; 1
Min. and max.	-1.37; 1.37

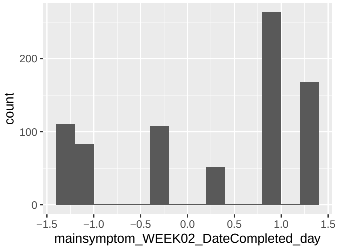

mainsymptom_WEEK02_DateCompleted_time

Feature	Result
Variable type	numeric
Number of missing obs.	0 (0 %)
Number of unique values	493
Median	-0.71
1st and 3rd quartiles	-1.18; -0.01
Min. and max.	-1.41; 1.41

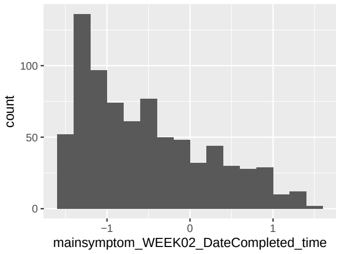

mainsymptom_WEEK03_sum

Feature	Result
Variable type	numeric
Number of missing obs.	0 (0 %)
Number of unique values	25
Median	-0.24
1st and 3rd quartiles	-0.87; 0.4
Min. and max.	-1.72; 3.99

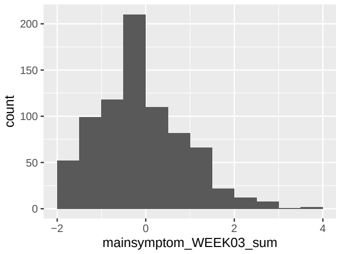

mainsymptom_WEEK03_duration

Feature	Result
Variable type	numeric
Number of missing obs.	0 (0 %)
Number of unique values	298
Median	-0.13
1st and 3rd quartiles	-0.18; -0.05
Min. and max.	-0.26; 31.82

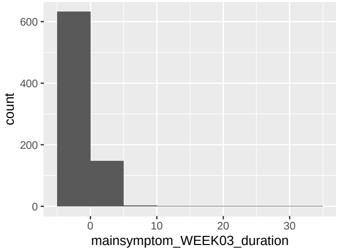

---

mainsymptom_WEEK03_DateCompleted_day

Feature	Result
Variable type	numeric
Number of missing obs.	0 (0 %)
Number of unique values	7
Median	1
1st and 3rd quartiles	-0.37; 1
Min. and max.	-1.37; 1.37

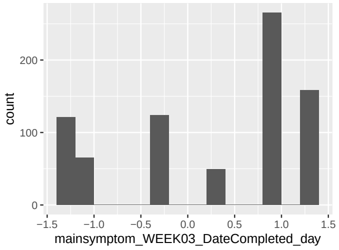

---

mainsymptom_WEEK03_DateCompleted_time

Feature	Result
Variable type	numeric
Number of missing obs.	0 (0 %)
Number of unique values	516
Median	-0.7
1st and 3rd quartiles	-1.18; 0.02
Min. and max.	-1.41; 1.41

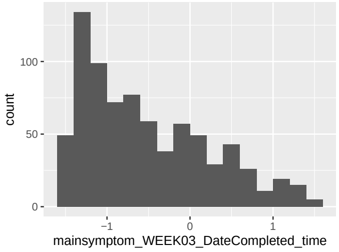

Report generation information:

- Created by: Could not determine from system (username: `nilisa`).
- Report creation time: Mon Jan 09 2023 13:08:19
- Report was run from directory: `/home/nilisa/projects/phd_study1/r`
- dataMaid v1.4.1 [Pkg: 2021-10-08 from CRAN (R 4.2.2)]
- R version 4.2.2 Patched (2022-11-10 r83330).
- Platform: x86_64-pc-linux-gnu (64-bit)(Ubuntu 20.04.5 LTS).
- Function call: `dataMaid::makeDataReport(data = gd, mode = c("summarize", "visualize", "check"), smartNum = FALSE, file = "~/projects/data/study1multiverse/results/graphs_n_figures/codebooks/codeboo replace = TRUE, openResult = FALSE, checks = list(character = "showAllFactorLevels", factor = "showAllFactorLevels", labelled = "showAllFactorLevels",   haven_labelled = "showAllFactorLevels", numeric = NULL,   integer = NULL, logical = NULL, Date = NULL), listChecks = FALSE, maxProbVals = Inf, codebook = TRUE, reportTitle = "Handpicked_Panic_week04-narem`

Handpicked_Social_week04-imputed_benchmark_test

Autogenerated data summary from dataMaid

2023-01-09 13:15:24

Data report overview

The dataset examined has the following dimensions:

Feature	Result
Number of observations	185
Number of variables	10

Codebook summary table

Label	Variable	Class	# unique values	Missing	Description
	**sex**	factor	2	0.00 %	Sex of patient, 0 = Female, 1=Male
	**age**	numeric	38	0.00 %	
	**PDSS-SR-3064_SCREEN_sum**	numeric	29	0.00 %	Anxiety questionnaire, self rated-Timepoint before treatment starts-Sum of the entire measure
	**MADRS-1951_SCREEN_sum**	numeric	43	0.00 %	Depression questionnaire, self rated-Timepoint before treatment starts-Sum of the entire measure
	**LSAS-2241_SCREEN_sum**	numeric	89	0.00 %	Social anxiety questionnaire, self rated-Timepoint before treatment starts-Sum of the entire measure
	**outcome**	numeric	110	0.00 %	
	**mainsymptom_PRE_sum**	numeric	96	0.00 %	PDSS-SR for panic, MADRS for depression, LSAS for social anxiety-Timepoint just before beginning treatment-Sum of the entire measure
	**mainsymptom_WEEK01_sum**	numeric	109	0.00 %	PDSS-SR for panic, MADRS for depression, LSAS for social anxiety-Timepoint after one week in treatment-Sum of the entire measure
	**mainsymptom_WEEK02_sum**	numeric	99	0.00 %	PDSS-SR for panic, MADRS for depression, LSAS for social anxiety-Timepoint after two weeks in treatment-Sum of the entire measure

Label	Variable	Class	# unique values	Missing	Description
	**mainsymptom_WEEK03_sum**	numeric	98	0.00 %	PDSS-SR for panic, MADRS for depression, LSAS for social anxiety-Timepoint after three weeks in treatment-Sum of the entire measure

Variable list

sex

Feature	Result
Variable type	factor
Number of missing obs.	0 (0 %)
Number of unique values	2
Mode	"0"
Reference category	0

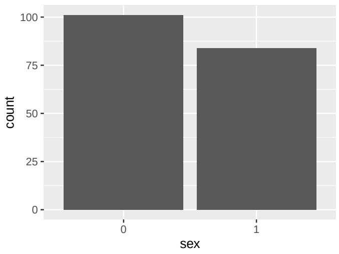

- Observed factor levels: "0", "1".

---

age

Feature	Result
Variable type	numeric
Number of missing obs.	0 (0 %)
Number of unique values	38
Median	-0.46
1st and 3rd quartiles	-0.9; 0.06
Min. and max.	-1.42; 2.51

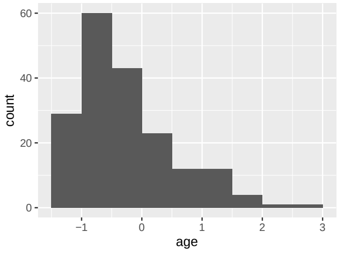

PDSS-SR-3064_SCREEN_sum

Feature	Result
Variable type	numeric
Number of missing obs.	0 (0 %)
Number of unique values	29
Median	-0.35
1st and 3rd quartiles	-0.82; 0.28
Min. and max.	-1.29; 2.49

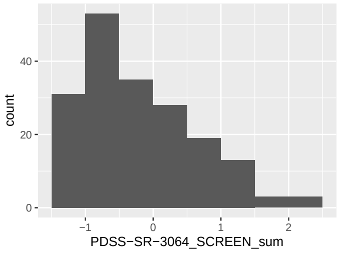

MADRS-1951_SCREEN_sum

Feature	Result
Variable type	numeric
Number of missing obs.	0 (0 %)
Number of unique values	43
Median	-0.57
1st and 3rd quartiles	-1.19; 0.3
Min. and max.	-2.68; 2.03

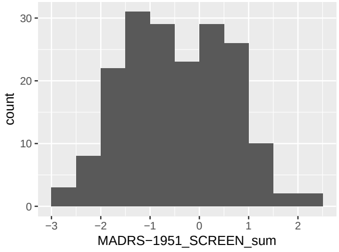

LSAS-2241_SCREEN_sum

Feature	Result
Variable type	numeric
Number of missing obs.	0 (0 %)
Number of unique values	89
Median	0.6
1st and 3rd quartiles	0.08; 1.26
Min. and max.	-1.23; 2.64

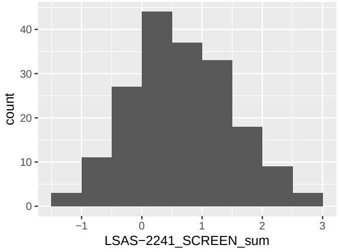

outcome

Feature	Result
Variable type	numeric
Number of missing obs.	0 (0 %)
Number of unique values	110
Median	-0.09
1st and 3rd quartiles	-0.6; 0.62
Min. and max.	-2.01; 2.79

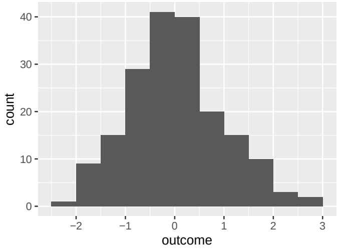

mainsymptom_PRE_sum

Feature	Result
Variable type	numeric
Number of missing obs.	0 (0 %)
Number of unique values	96
Median	-0.08
1st and 3rd quartiles	-0.72; 0.8
Min. and max.	-2.62; 2.41

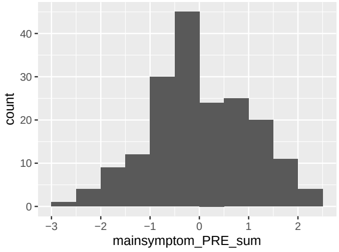

mainsymptom_WEEK01_sum

Feature	Result
Variable type	numeric
Number of missing obs.	0 (0 %)
Number of unique values	109
Median	-0.09
1st and 3rd quartiles	-0.58; 0.71
Min. and max.	-2.84; 2.46

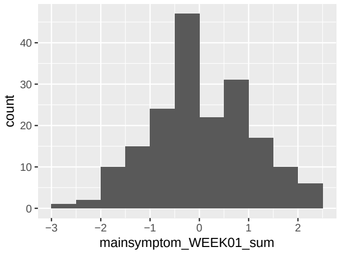

---

mainsymptom_WEEK02_sum

Feature	Result
Variable type	numeric
Number of missing obs.	0 (0 %)
Number of unique values	99
Median	-0.03
1st and 3rd quartiles	-0.72; 0.74
Min. and max.	-2.75; 2.44

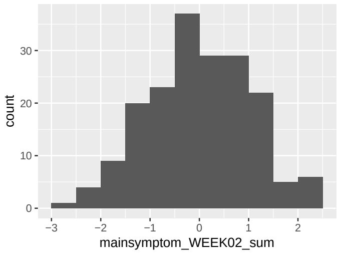

---

mainsymptom_WEEK03_sum

Feature	Result
Variable type	numeric
Number of missing obs.	0 (0 %)
Number of unique values	98
Median	-0.03
1st and 3rd quartiles	-0.63; 0.78
Min. and max.	-2.64; 2.26

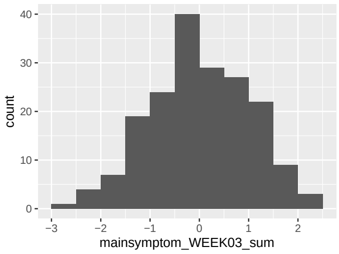

Report generation information:

- Created by: Could not determine from system (username: `nilisa`).
- Report creation time: Mon Jan 09 2023 13:15:25
- Report was run from directory: `/home/nilisa/projects/phd_study1/r`
- dataMaid v1.4.1 [Pkg: 2021-10-08 from CRAN (R 4.2.2)]
- R version 4.2.2 Patched (2022-11-10 r83330).
- Platform: x86_64-pc-linux-gnu (64-bit)(Ubuntu 20.04.5 LTS).
- Function call: `dataMaid::makeDataReport(data = gd, mode = c("summarize", "visualize", "check"), smartNum = FALSE, file = "~/projects/data/study1multiverse/results/graphs_n_figures/codebooks/codebook replace = TRUE, openResult = FALSE, checks = list(character = "showAllFactorLevels", factor = "showAllFactorLevels", labelled = "showAllFactorLevels",   haven_labelled = "showAllFactorLevels", numeric = NULL,   integer = NULL, logical = NULL, Date = NULL), listChecks = FALSE, maxProbVals = Inf, codebook = TRUE, reportTitle = "Handpicked_Social_week04-imput`

Handpicked_Social_week04-imputed_benchmark_train

Autogenerated data summary from dataMaid

2023-01-09 13:08:57

Data report overview

The dataset examined has the following dimensions:

Feature	Result
Number of observations	1667
Number of variables	10

Codebook summary table

Label	Variable	Class	# unique values	Missing	Description
	**sex**	factor	2	0.00 %	Sex of patient, 0 = Female, 1=Male
	**age**	numeric	58	0.00 %	
	**PDSS-SR-3064_SCREEN_sum**	numeric	109	0.00 %	Anxiety questionnaire, self rated-Timepoint before treatment starts-Sum of the entire measure
	**MADRS-1951_SCREEN_sum**	numeric	120	0.00 %	Depression questionnaire, self rated-Timepoint before treatment starts-Sum of the entire measure
	**LSAS-2241_SCREEN_sum**	numeric	203	0.00 %	Social anxiety questionnaire, self rated-Timepoint before treatment starts-Sum of the entire measure
	**outcome**	numeric	533	0.00 %	
	**mainsymptom_PRE_sum**	numeric	258	0.00 %	PDSS-SR for panic, MADRS for depression, LSAS for social anxiety-Timepoint just before beginning treatment-Sum of the entire measure
	**mainsymptom_WEEK01_sum**	numeric	410	0.00 %	PDSS-SR for panic, MADRS for depression, LSAS for social anxiety-Timepoint after one week in treatment-Sum of the entire measure
	**mainsymptom_WEEK02_sum**	numeric	355	0.00 %	PDSS-SR for panic, MADRS for depression, LSAS for social anxiety-Timepoint after two weeks in treatment-Sum of the entire measure

Label	Variable	Class	# unique values	Missing	Description
	**mainsymptom_WEEK03_sum**	numeric	371	0.00 %	PDSS-SR for panic, MADRS for depression, LSAS for social anxiety-Timepoint after three weeks in treatment-Sum of the entire measure

Variable list

sex

Feature	Result
Variable type	factor
Number of missing obs.	0 (0 %)
Number of unique values	2
Mode	"0"
Reference category	0

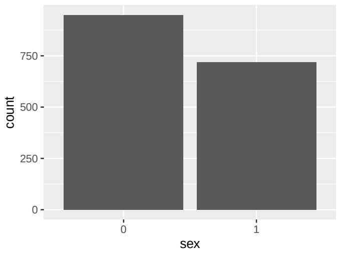

- Observed factor levels: "0", "1".

---

age

Feature	Result
Variable type	numeric
Number of missing obs.	0 (0 %)
Number of unique values	58
Median	-0.46
1st and 3rd quartiles	-0.9; 0.24
Min. and max.	-1.69; 4.25

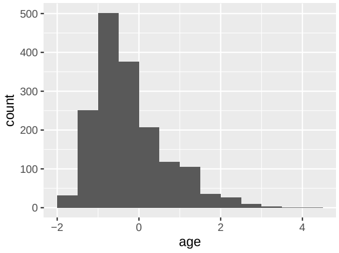

PDSS-SR-3064_SCREEN_sum

Feature	Result
Variable type	numeric
Number of missing obs.	0 (0 %)
Number of unique values	109
Median	-0.19
1st and 3rd quartiles	-0.98; 0.52
Min. and max.	-1.29; 2.8

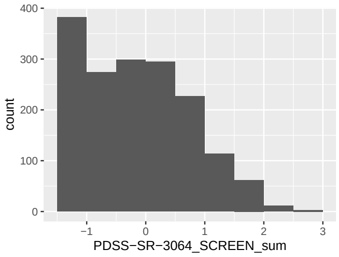

MADRS-1951_SCREEN_sum

Feature	Result
Variable type	numeric
Number of missing obs.	0 (0 %)
Number of unique values	120
Median	-0.45
1st and 3rd quartiles	-1.19; 0.17
Min. and max.	-2.68; 2.28

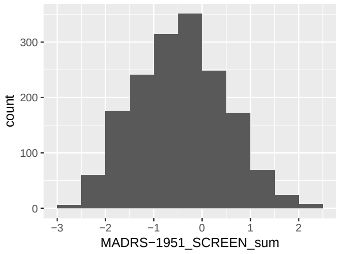

LSAS-2241_SCREEN_sum

Feature	Result
Variable type	numeric
Number of missing obs.	0 (0 %)
Number of unique values	203
Median	0.67
1st and 3rd quartiles	0.12; 1.25
Min. and max.	-1.44; 3.09

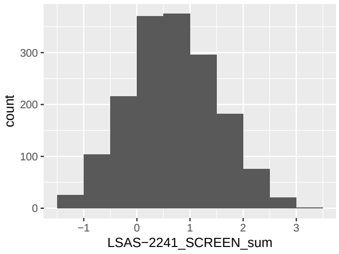

outcome

Feature	Result
Variable type	numeric
Number of missing obs.	0 (0 %)
Number of unique values	533
Median	-0.05
1st and 3rd quartiles	-0.64; 0.62
Min. and max.	-2.05; 3.53

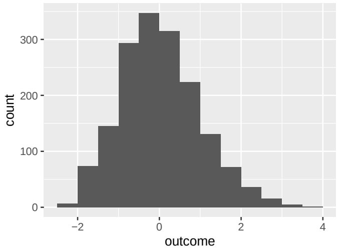

mainsymptom_PRE_sum

Feature	Result
Variable type	numeric
Number of missing obs.	0 (0 %)
Number of unique values	258
Median	0
1st and 3rd quartiles	-0.68; 0.63
Min. and max.	-2.7; 3

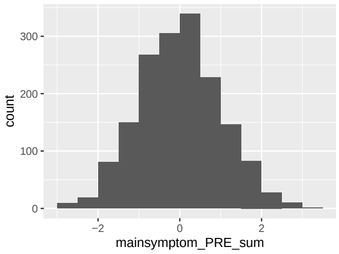

mainsymptom_WEEK01_sum

Feature	Result
Variable type	numeric
Number of missing obs.	0 (0 %)
Number of unique values	410
Median	-0.05
1st and 3rd quartiles	-0.7; 0.61
Min. and max.	-2.84; 3.08

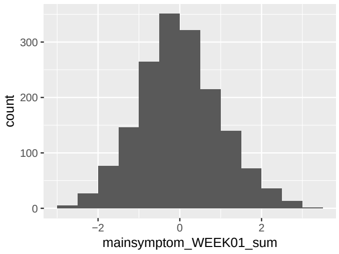

mainsymptom_WEEK02_sum

Feature	Result
Variable type	numeric
Number of missing obs.	0 (0 %)
Number of unique values	355
Median	-0.03
1st and 3rd quartiles	-0.68; 0.65
Min. and max.	-2.75; 3.01

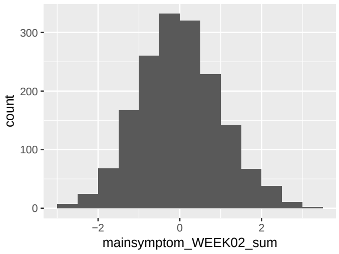

mainsymptom_WEEK03_sum

Feature	Result
Variable type	numeric
Number of missing obs.	0 (0 %)
Number of unique values	371
Median	-0.03
1st and 3rd quartiles	-0.67; 0.69
Min. and max.	-2.64; 3.07

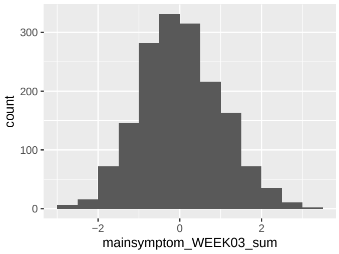

Report generation information:

- Created by: Could not determine from system (username: `nilisa`).
- Report creation time: Mon Jan 09 2023 13:08:57
- Report was run from directory: `/home/nilisa/projects/phd_study1/r`
- dataMaid v1.4.1 [Pkg: 2021-10-08 from CRAN (R 4.2.2)]
- R version 4.2.2 Patched (2022-11-10 r83330).
- Platform: x86_64-pc-linux-gnu (64-bit)(Ubuntu 20.04.5 LTS).
- Function call: `dataMaid::makeDataReport(data = gd, mode = c("summarize", "visualize", "check"), smartNum = FALSE, file = "~/projects/data/study1multiverse/results/graphs_n_figures/codebooks/codebook replace = TRUE, openResult = FALSE, checks = list(character = "showAllFactorLevels", factor = "showAllFactorLevels", labelled = "showAllFactorLevels",   haven_labelled = "showAllFactorLevels", numeric = NULL,   integer = NULL, logical = NULL, Date = NULL), listChecks = FALSE, maxProbVals = Inf, codebook = TRUE, reportTitle = "Handpicked_Social_week04-imput`

Handpicked_Social_week04-imputed_test

Autogenerated data summary from dataMaid

2023-01-09 13:15:33

Data report overview

The dataset examined has the following dimensions:

Feature	Result
Number of observations	185
Number of variables	63

Codebook summary table

Label	Variable	Class	# unique values	Missing	Description
	**sex**	factor	2	0.00 %	Sex of patient, 0 = Female, 1=Male
	**age**	numeric	38	0.00 %	
	**messages_len_7**	numeric	52	0.00 %	-Meta information of messages-Length of messages-up until day-7
	**messages_len_tp_7**	numeric	135	0.00 %	-Meta information of messages-Length of messages-therapist messages-up until day-7
	**messages_7**	numeric	5	0.00 %	-Meta information of messages-up until day-7
	**messages_tp_7**	numeric	5	0.00 %	-Meta information of messages-therapist messages-up until day-7
	**homeworks_7**	numeric	4	0.00 %	-Number of homework messages sent in-up until day-7
	**messages_len_14**	numeric	69	0.00 %	-Meta information of messages-Length of messages-up until day-14
	**messages_len_tp_14**	numeric	155	0.00 %	-Meta information of messages-Length of messages-therapist messages-up until day-14
	**messages_14**	numeric	5	0.00 %	-Meta information of messages-up until day-14
	**messages_tp_14**	numeric	4	0.00 %	-Meta information of messages-therapist messages-up until day-14

Label	Variable	Class	# unique values	Missing	Description
	**homeworks_14**	numeric	4	0.00 %	-Number of homework messages sent in-up until day-14
	**messages_len_21**	numeric	73	0.00 %	-Meta information of messages-Length of messages-up until day-21
	**messages_len_tp_21**	numeric	144	0.00 %	-Meta information of messages-Length of messages-therapist messages-up until day-21
	**messages_21**	numeric	5	0.00 %	-Meta information of messages-up until day-21
	**messages_tp_21**	numeric	5	0.00 %	-Meta information of messages-therapist messages-up until day-21
	**homeworks_21**	numeric	4	0.00 %	-Number of homework messages sent in-up until day-21
	**messages_len_28**	numeric	75	0.00 %	-Meta information of messages-Length of messages-up until day-28
	**messages_len_tp_28**	numeric	148	0.00 %	-Meta information of messages-Length of messages-therapist messages-up until day-28
	**messages_28**	numeric	5	0.00 %	-Meta information of messages-up until day-28
	**messages_tp_28**	numeric	5	0.00 %	-Meta information of messages-therapist messages-up until day-28
	**homeworks_28**	numeric	6	0.00 %	-Number of homework messages sent in-up until day-28
	**PDSS-SR-3064_SCREEN_sum**	numeric	29	0.00 %	Anxiety questionnaire, self rated-Timepoint before treatment starts-Sum of the entire measure
	**MADRS-1951_SCREEN_sum**	numeric	43	0.00 %	Depression questionnaire, self rated-Timepoint before treatment starts-Sum of the entire measure
	**LSAS-2241_SCREEN_sum**	numeric	89	0.00 %	Social anxiety questionnaire, self rated-Timepoint before treatment starts-Sum of the entire measure
	**MADRS-1951_SCREEN_DateCompleted_day**	numeric	14	0.00 %	Depression questionnaire, self rated-Timepoint before treatment starts-Cyclic transformation of what day 0-6 during week it was filled in

Label	Variable	Class	# unique values	Missing	Description
	**MADRS-1951_SCREEN_DateCompleted_time**	numeric	165	0.00 %	Depression questionnaire, self rated-Timepoint before treatment starts-Cyclic transformation of what time during day 0-1440 it was filled in
	**PDSS-SR-3064_SCREEN_DateCompleted_day**	numeric	13	0.00 %	Anxiety questionnaire, self rated-Timepoint before treatment starts-Cyclic transformation of what day 0-6 during week it was filled in
	**PDSS-SR-3064_SCREEN_DateCompleted_time**	numeric	167	0.00 %	Anxiety questionnaire, self rated-Timepoint before treatment starts-Cyclic transformation of what time during day 0-1440 it was filled in
	**LSAS-2241_SCREEN_DateCompleted_day**	numeric	9	0.00 %	Social anxiety questionnaire, self rated-Timepoint before treatment starts-Cyclic transformation of what day 0-6 during week it was filled in
	**LSAS-2241_SCREEN_DateCompleted_time**	numeric	168	0.00 %	Social anxiety questionnaire, self rated-Timepoint before treatment starts-Cyclic transformation of what time during day 0-1440 it was filled in
	**outcome**	numeric	110	0.00 %	
	**ncomorbid**	numeric	20	0.00 %	
	**HW-01**	numeric	185	0.00 %	
	**HW-02**	numeric	180	0.00 %	
	**HW-03**	numeric	185	0.00 %	
	**currentwork_proff**	factor	8	0.00 %	Currently in work for trained proffession
	**Marital_1833_gift**	factor	2	0.00 %	Marital status: Married or not
	**Marital_1833_separerad**	factor	2	0.00 %	Marital status: divocered/equivalent
	**Marital_1833_singel**	factor	2	0.00 %	Marital status: single
	**Edu_1843_2**	factor	2	0.00 %	7-9 years education
	**Edu_1843_3**	factor	2	0.00 %	Uncompleted upper secondary school
	**Edu_1843_4**	factor	2	0.00 %	Higher vocational education
	**Edu_1843_5**	factor	2	0.00 %	Completed upper secondary school
	**Edu_1843_6**	factor	2	0.00 %	Uncompleted university degree
	**Edu_1843_7**	factor	2	0.00 %	University degree
	**cscale**	numeric	47	0.00 %	
	**mainsymptom_PRE_sum**	numeric	96	0.00 %	PDSS-SR for panic, MADRS for depression, LSAS for social anxiety-Timepoint just before beginning treatment-Sum of the entire measure

Label	Variable	Class	# unique values	Missing	Description
	**mainsymptom_PRE_duration**	numeric	164	0.00 %	PDSS-SR for panic, MADRS for depression, LSAS for social anxiety-Timepoint just before beginning treatment-Time to fill in measure/questionnaire
	**mainsymptom_PRE_DateCompleted_day**	numeric	20	0.00 %	PDSS-SR for panic, MADRS for depression, LSAS for social anxiety-Timepoint just before beginning treatment-Cyclic transformation of what day 0-6 during week it was filled in
	**mainsymptom_PRE_DateCompleted_time**	numeric	167	0.00 %	PDSS-SR for panic, MADRS for depression, LSAS for social anxiety-Timepoint just before beginning treatment-Cyclic transformation of what time during day 0-1440 it was filled in
	**mainsymptom_WEEK01_sum**	numeric	109	0.00 %	PDSS-SR for panic, MADRS for depression, LSAS for social anxiety-Timepoint after one week in treatment-Sum of the entire measure
	**mainsymptom_WEEK01_duration**	numeric	163	0.00 %	PDSS-SR for panic, MADRS for depression, LSAS for social anxiety-Timepoint after one week in treatment-Time to fill in measure/questionnaire
	**mainsymptom_WEEK01_DateCompleted_day**	numeric	32	0.00 %	PDSS-SR for panic, MADRS for depression, LSAS for social anxiety-Timepoint after one week in treatment-Cyclic transformation of what day 0-6 during week it was filled in
	**mainsymptom_WEEK01_DateCompleted_time**	numeric	169	0.00 %	PDSS-SR for panic, MADRS for depression, LSAS for social anxiety-Timepoint after one week in treatment-Cyclic transformation of what time during day 0-1440 it was filled in
	**mainsymptom_WEEK02_sum**	numeric	99	0.00 %	PDSS-SR for panic, MADRS for depression, LSAS for social anxiety-Timepoint after two weeks in treatment-Sum of the entire measure
	**mainsymptom_WEEK02_duration**	numeric	147	0.00 %	PDSS-SR for panic, MADRS for depression, LSAS for social anxiety-Timepoint after two weeks in treatment-Time to fill in measure/questionnaire

Label	Variable	Class	# unique values	Missing	Description
	**mainsymptom_WEEK02_DateCompleted_day**	numeric	24	0.00 %	PDSS-SR for panic, MADRS for depression, LSAS for social anxiety-Timepoint after two weeks in treatment-Cyclic transformation of what day 0-6 during week it was filled in
	**mainsymptom_WEEK02_DateCompleted_time**	numeric	172	0.00 %	PDSS-SR for panic, MADRS for depression, LSAS for social anxiety-Timepoint after two weeks in treatment-Cyclic transformation of what time during day 0-1440 it was filled in
	**mainsymptom_WEEK03_sum**	numeric	98	0.00 %	PDSS-SR for panic, MADRS for depression, LSAS for social anxiety-Timepoint after three weeks in treatment-Sum of the entire measure
	**mainsymptom_WEEK03_duration**	numeric	155	0.00 %	PDSS-SR for panic, MADRS for depression, LSAS for social anxiety-Timepoint after three weeks in treatment-Time to fill in measure/questionnaire
	**mainsymptom_WEEK03_DateCompleted_day**	numeric	25	0.00 %	PDSS-SR for panic, MADRS for depression, LSAS for social anxiety-Timepoint after three weeks in treatment-Cyclic transformation of what day 0-6 during week it was filled in
	**mainsymptom_WEEK03_DateCompleted_time**	numeric	173	0.00 %	PDSS-SR for panic, MADRS for depression, LSAS for social anxiety-Timepoint after three weeks in treatment-Cyclic transformation of what time during day 0-1440 it was filled in

Variable list

sex

Feature	Result
Variable type	factor
Number of missing obs.	0 (0 %)
Number of unique values	2
Mode	“0”
Reference category	0

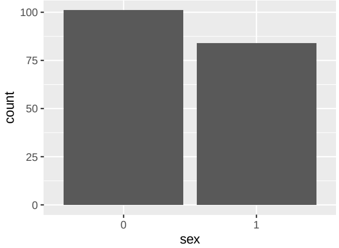

- Observed factor levels: "0", "1".

---

age

Feature	Result
Variable type	numeric
Number of missing obs.	0 (0 %)
Number of unique values	38
Median	-0.46
1st and 3rd quartiles	-0.9; 0.06
Min. and max.	-1.42; 2.51

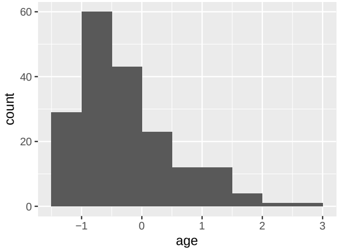

messages_len_7

Feature	Result
Variable type	numeric
Number of missing obs.	0 (0 %)
Number of unique values	52
Median	-0.32
1st and 3rd quartiles	-0.32; -0.14
Min. and max.	-0.32; 5.33

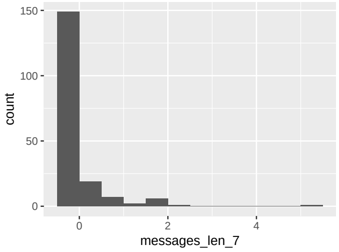

messages_len_tp_7

Feature	Result
Variable type	numeric
Number of missing obs.	0 (0 %)
Number of unique values	135
Median	0.12
1st and 3rd quartiles	-0.62; 0.79
Min. and max.	-1.1; 2.77

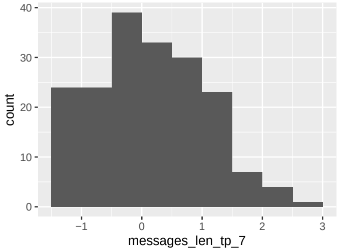

messages_7

Feature	Result
Variable type	numeric
Number of missing obs.	0 (0 %)
Number of unique values	5
Median	-0.55
1st and 3rd quartiles	-0.55; 0.58
Min. and max.	-0.55; 5.13

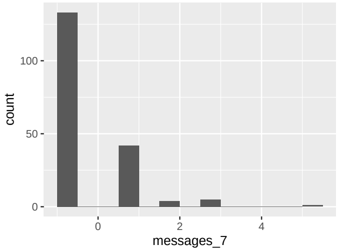

messages_tp_7

Feature	Result
Variable type	numeric
Number of missing obs.	0 (0 %)
Number of unique values	5
Median	0.15
1st and 3rd quartiles	-0.91; 0.15
Min. and max.	-0.91; 3.34

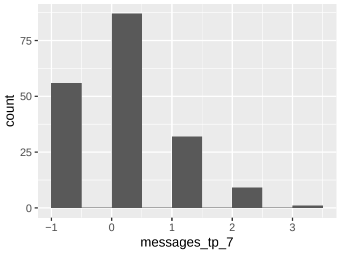

homeworks_7

Feature	Result
Variable type	numeric
Number of missing obs.	0 (0 %)
Number of unique values	4
Median	0.2
1st and 3rd quartiles	-1.03; 0.2
Min. and max.	-1.03; 2.68

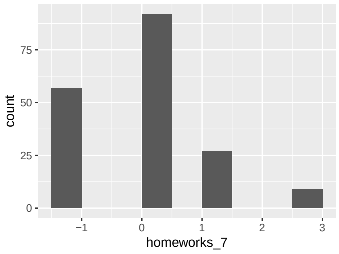

messages_len_14

Feature	Result
Variable type	numeric
Number of missing obs.	0 (0 %)
Number of unique values	69
Median	-0.41
1st and 3rd quartiles	-0.41; -0.08
Min. and max.	-0.41; 7.91

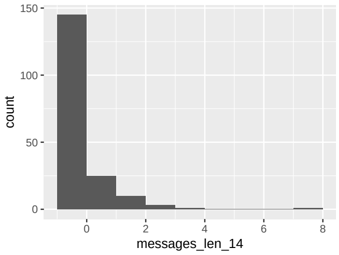

messages_len_tp_14

Feature	Result
Variable type	numeric
Number of missing obs.	0 (0 %)
Number of unique values	155
Median	-0.06
1st and 3rd quartiles	-0.59; 0.53
Min. and max.	-1.11; 4.23

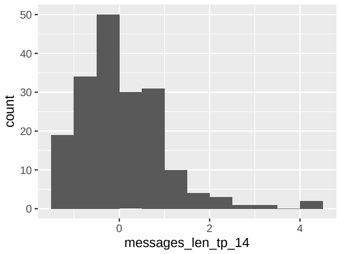

messages_14

Feature	Result
Variable type	numeric
Number of missing obs.	0 (0 %)
Number of unique values	5
Median	-0.7
1st and 3rd quartiles	-0.7; 0.36
Min. and max.	-0.7; 3.54

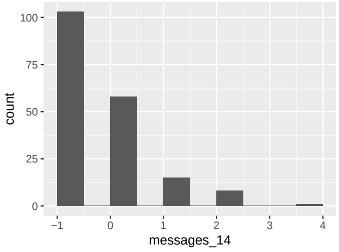

messages_tp_14

Feature	Result
Variable type	numeric
Number of missing obs.	0 (0 %)
Number of unique values	4
Median	-0.35
1st and 3rd quartiles	-0.35; 0.87
Min. and max.	-1.57; 2.09

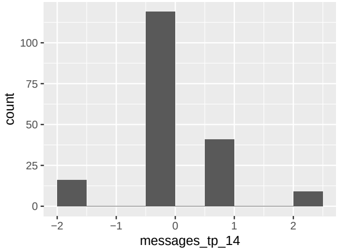

homeworks_14

Feature	Result
Variable type	numeric
Number of missing obs.	0 (0 %)
Number of unique values	4
Median	0.33
1st and 3rd quartiles	-0.98; 0.33
Min. and max.	-0.98; 2.96

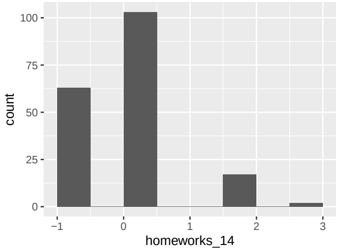

messages_len_21

Feature	Result
Variable type	numeric
Number of missing obs.	0 (0 %)
Number of unique values	73
Median	-0.46
1st and 3rd quartiles	-0.46; -0.09
Min. and max.	-0.46; 5.36

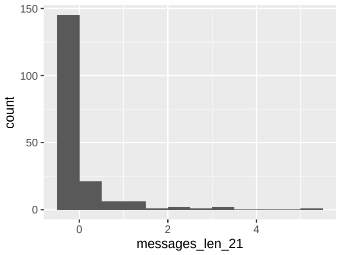

messages_len_tp_21

Feature	Result
Variable type	numeric
Number of missing obs.	0 (0 %)
Number of unique values	144
Median	-0.2
1st and 3rd quartiles	-0.74; 0.45
Min. and max.	-1.01; 7.08

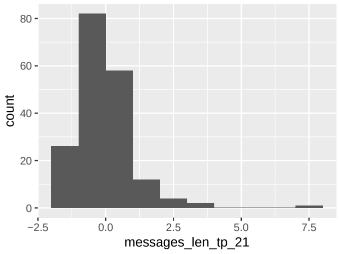

messages_21

Feature	Result
Variable type	numeric
Number of missing obs.	0 (0 %)
Number of unique values	5
Median	-0.74
1st and 3rd quartiles	-0.74; 0.34
Min. and max.	-0.74; 4.66

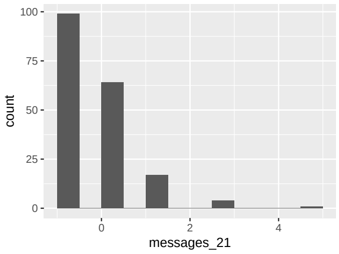

messages_tp_21

Feature	Result
Variable type	numeric
Number of missing obs.	0 (0 %)
Number of unique values	5
Median	-0.32
1st and 3rd quartiles	-0.32; 0.84
Min. and max.	-1.49; 4.33

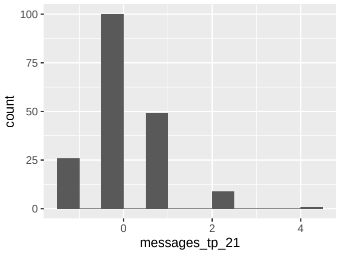

homeworks_21

Feature	Result
Variable type	numeric
Number of missing obs.	0 (0 %)
Number of unique values	4
Median	0.36
1st and 3rd quartiles	-1.01; 0.36
Min. and max.	-1.01; 3.11

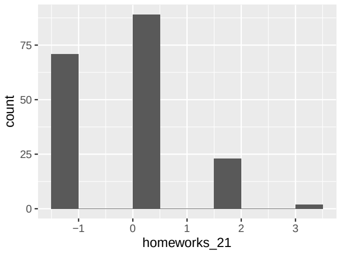

messages_len_28

Feature	Result
Variable type	numeric
Number of missing obs.	0 (0 %)
Number of unique values	75
Median	-0.41
1st and 3rd quartiles	-0.41; -0.01
Min. and max.	-0.41; 3.39

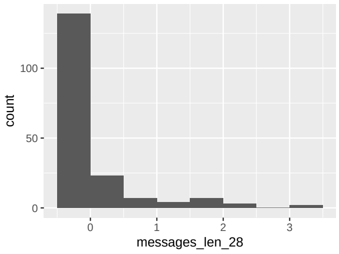

messages_len_tp_28

Feature	Result
Variable type	numeric
Number of missing obs.	0 (0 %)
Number of unique values	148
Median	-0.1
1st and 3rd quartiles	-0.71; 0.57
Min. and max.	-0.94; 3.6

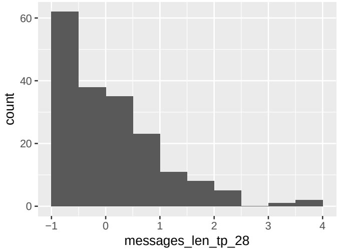

messages_28

Feature	Result
Variable type	numeric
Number of missing obs.	0 (0 %)
Number of unique values	5
Median	-0.72
1st and 3rd quartiles	-0.72; 0.41
Min. and max.	-0.72; 3.8

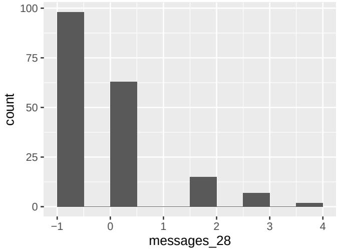

messages_tp_28

Feature	Result
Variable type	numeric
Number of missing obs.	0 (0 %)
Number of unique values	5
Median	-0.24
1st and 3rd quartiles	-0.24; 0.93
Min. and max.	-1.42; 3.28

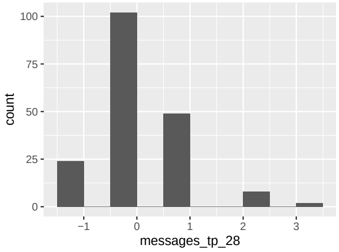

homeworks_28

Feature	Result
Variable type	numeric
Number of missing obs.	0 (0 %)
Number of unique values	6
Median	0.46
1st and 3rd quartiles	-0.91; 0.46
Min. and max.	-0.91; 10.05

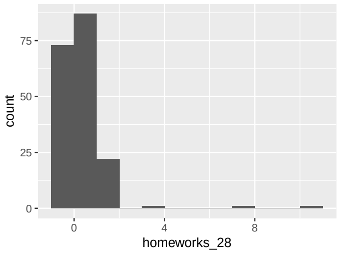

PDSS-SR-3064_SCREEN_sum

Feature	Result
Variable type	numeric
Number of missing obs.	0 (0 %)
Number of unique values	29
Median	-0.35
1st and 3rd quartiles	-0.82; 0.28
Min. and max.	-1.29; 2.49

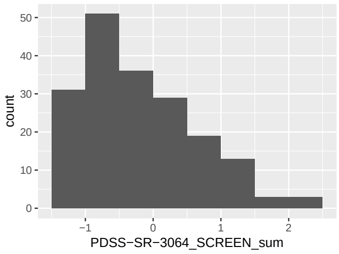

MADRS-1951_SCREEN_sum

Feature	Result
Variable type	numeric
Number of missing obs.	0 (0 %)
Number of unique values	43
Median	-0.45
1st and 3rd quartiles	-1.19; 0.3
Min. and max.	-2.68; 2.03

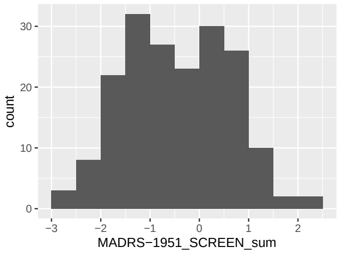

LSAS-2241_SCREEN_sum

Feature	Result
Variable type	numeric
Number of missing obs.	0 (0 %)
Number of unique values	89
Median	0.6
1st and 3rd quartiles	0.08; 1.26
Min. and max.	-1.23; 2.64

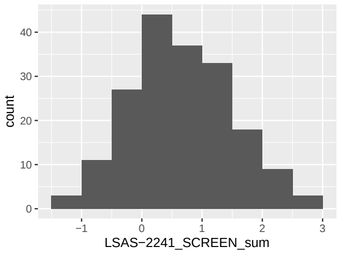

MADRS-1951_SCREEN_DateCompleted_day

Feature	Result
Variable type	numeric
Number of missing obs.	0 (0 %)
Number of unique values	14
Median	0.38
1st and 3rd quartiles	-0.37; 1
Min. and max.	-1.37; 1.37

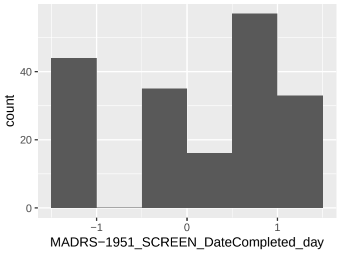

MADRS-1951_SCREEN_DateCompleted_time

Feature	Result
Variable type	numeric
Number of missing obs.	0 (0 %)
Number of unique values	165
Median	-0.44
1st and 3rd quartiles	-1.14; 0.16
Min. and max.	-1.41; 1.41

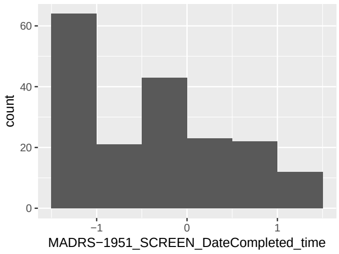

PDSS-SR-3064_SCREEN_DateCompleted_day

Feature	Result
Variable type	numeric
Number of missing obs.	0 (0 %)
Number of unique values	13
Median	0.38
1st and 3rd quartiles	-1; 1
Min. and max.	-1.37; 1.37

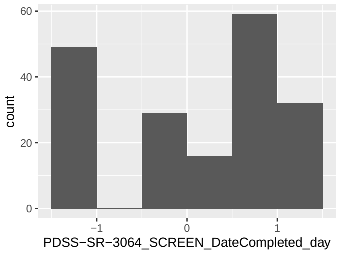

PDSS-SR-3064_SCREEN_DateCompleted_time

Feature	Result
Variable type	numeric
Number of missing obs.	0 (0 %)
Number of unique values	167
Median	-0.45
1st and 3rd quartiles	-1.17; 0.26
Min. and max.	-1.41; 1.41

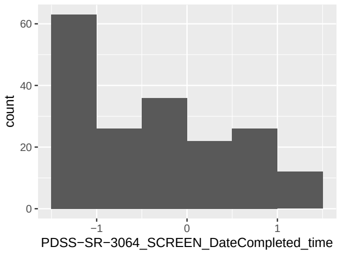

LSAS-2241_SCREEN_DateCompleted_day

Feature	Result
Variable type	numeric
Number of missing obs.	0 (0 %)
Number of unique values	9
Median	1
1st and 3rd quartiles	-0.37; 1
Min. and max.	-1.37; 1.37

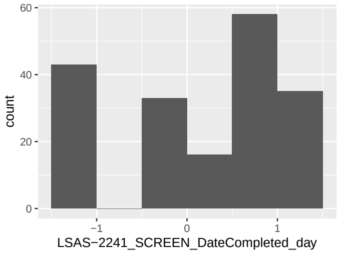

LSAS-2241_SCREEN_DateCompleted_time

Feature	Result
Variable type	numeric
Number of missing obs.	0 (0 %)
Number of unique values	168
Median	-0.43
1st and 3rd quartiles	-1.14; 0.18
Min. and max.	-1.41; 1.41

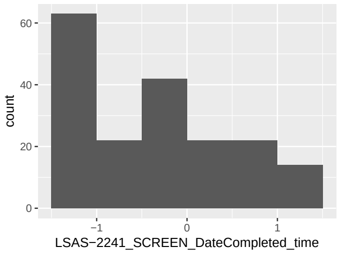

outcome

Feature	Result
Variable type	numeric
Number of missing obs.	0 (0 %)
Number of unique values	110
Median	-0.01
1st and 3rd quartiles	-0.6; 0.62
Min. and max.	-2.01; 2.79

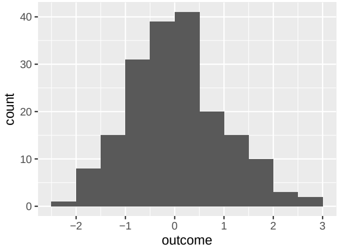

ncomorbid

Feature	Result
Variable type	numeric
Number of missing obs.	0 (0 %)
Number of unique values	20
Median	0
1st and 3rd quartiles	0; 1
Min. and max.	0; 4

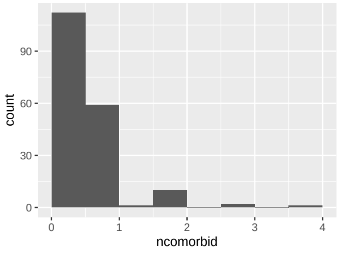

HW-01

Feature	Result
Variable type	numeric
Number of missing obs.	0 (0 %)
Number of unique values	185
Median	-0.14
1st and 3rd quartiles	-0.69; 0.52
Min. and max.	-1.44; 2.54

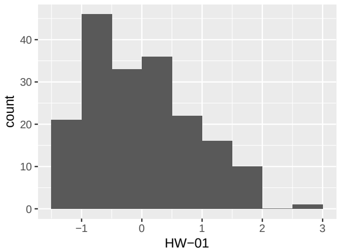

HW-02

Feature	Result
Variable type	numeric
Number of missing obs.	0 (0 %)
Number of unique values	180
Median	-0.16
1st and 3rd quartiles	-0.42; 0.16
Min. and max.	-1.21; 6.32

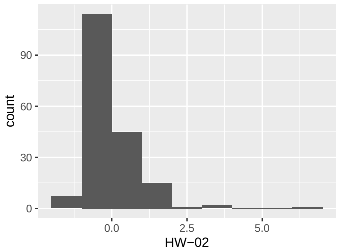

HW-03

Feature	Result
Variable type	numeric
Number of missing obs.	0 (0 %)
Number of unique values	185
Median	0.11
1st and 3rd quartiles	-0.06; 0.23
Min. and max.	-4.83; 0.58

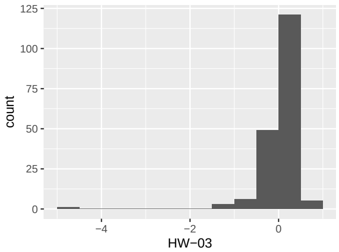

---

currentwork_proff

Feature	Result
Variable type	factor
Number of missing obs.	0 (0 %)
Number of unique values	8
Mode	“1”
Reference category	0

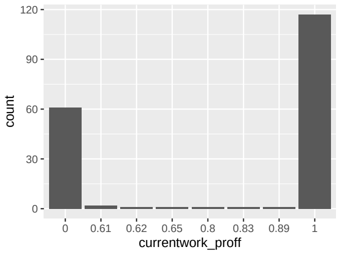

- Observed factor levels: "0", "0.61", "0.62", "0.65", "0.8", "0.83", "0.89", "1".

Marital_1833_gift

Feature	Result
Variable type	factor
Number of missing obs.	0 (0 %)
Number of unique values	2
Mode	"1"
Reference category	0

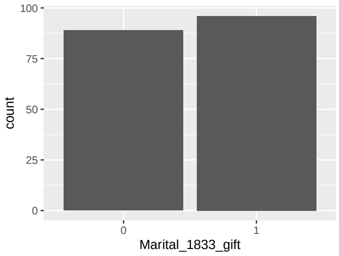

- Observed factor levels: "0", "1".

Marital_1833_separerad

Feature	Result
Variable type	factor
Number of missing obs.	0 (0 %)
Number of unique values	2
Mode	"0"
Reference category	0

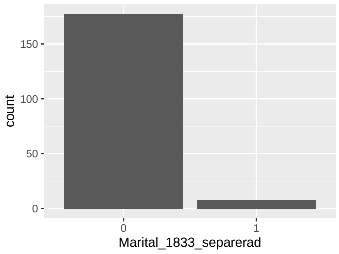

- Observed factor levels: "0", "1".

Marital_1833_singel

Feature	Result
Variable type	factor
Number of missing obs.	0 (0 %)
Number of unique values	2
Mode	"0"
Reference category	0

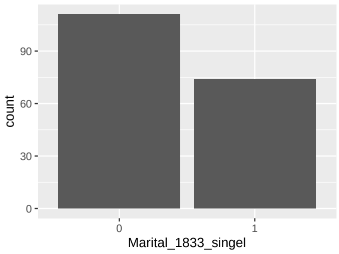

- Observed factor levels: "0", "1".

Edu_1843_2

Feature	Result
Variable type	factor
Number of missing obs.	0 (0 %)
Number of unique values	2
Mode	"0"
Reference category	0

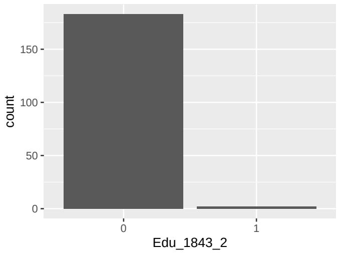

- Observed factor levels: "0", "1".

Edu_1843_3

Feature	Result
Variable type	factor
Number of missing obs.	0 (0 %)
Number of unique values	2
Mode	“0”
Reference category	0

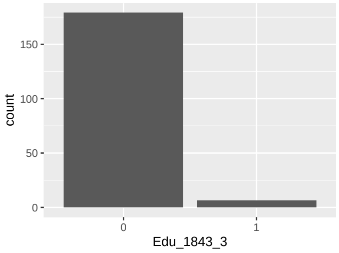

- Observed factor levels: "0", "1".

Edu_1843_4

Feature	Result
Variable type	factor
Number of missing obs.	0 (0 %)
Number of unique values	2
Mode	“0”
Reference category	0

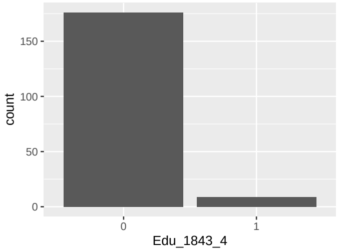

- Observed factor levels: "0", "1".

Edu_1843_5

Feature	Result
Variable type	factor
Number of missing obs.	0 (0 %)
Number of unique values	2
Mode	“0”
Reference category	0

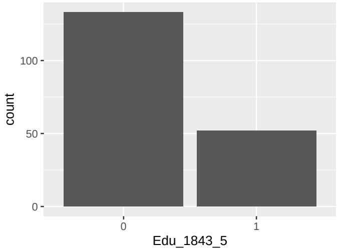

- Observed factor levels: "0", "1".

Edu_1843_6

Feature	Result
Variable type	factor
Number of missing obs.	0 (0 %)
Number of unique values	2
Mode	“0”
Reference category	0

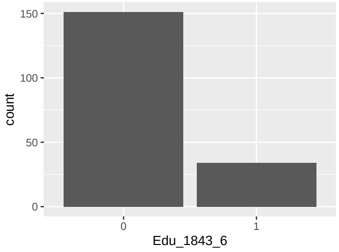

- Observed factor levels: "0", "1".

Edu_1843_7

Feature	Result
Variable type	factor
Number of missing obs.	0 (0 %)
Number of unique values	2
Mode	“0”
Reference category	0

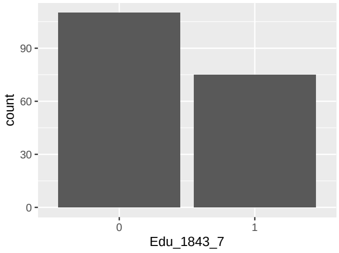

- Observed factor levels: "0", "1".

---

cscale

Feature	Result
Variable type	numeric
Number of missing obs.	0 (0 %)
Number of unique values	47
Median	0.14
1st and 3rd quartiles	-0.23; 0.72
Min. and max.	-4.14; 1.79

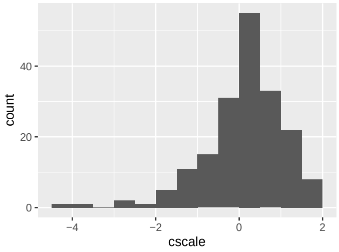

---

mainsymptom_PRE_sum

Feature	Result
Variable type	numeric
Number of missing obs.	0 (0 %)
Number of unique values	96
Median	-0.08
1st and 3rd quartiles	-0.72; 0.8
Min. and max.	-2.62; 2.41

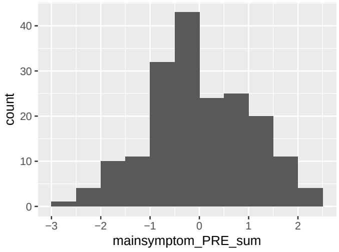

---

mainsymptom_PRE_duration

Feature	Result
Variable type	numeric
Number of missing obs.	0 (0 %)
Number of unique values	164
Median	-0.12
1st and 3rd quartiles	-0.24; -0.01
Min. and max.	-0.39; 4.65

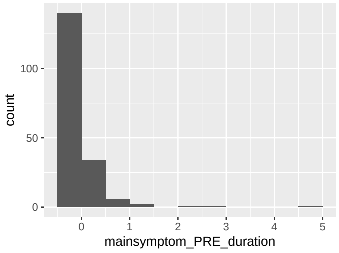

---

mainsymptom_PRE_DateCompleted_day

Feature	Result
Variable type	numeric
Number of missing obs.	0 (0 %)
Number of unique values	20
Median	0.74
1st and 3rd quartiles	-0.37; 1
Min. and max.	-1.37; 1.37

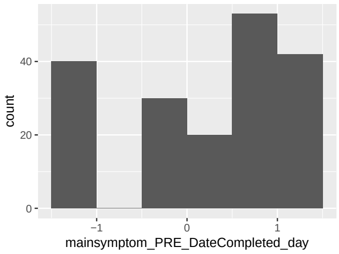

mainsymptom_PRE_DateCompleted_time

Feature	Result
Variable type	numeric
Number of missing obs.	0 (0 %)
Number of unique values	167
Median	-0.66
1st and 3rd quartiles	-1.29; 0.04
Min. and max.	-1.41; 1.41

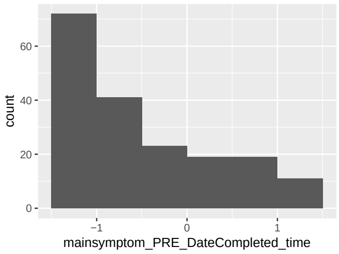

mainsymptom_WEEK01_sum

Feature	Result
Variable type	numeric
Number of missing obs.	0 (0 %)
Number of unique values	109
Median	-0.09
1st and 3rd quartiles	-0.58; 0.69
Min. and max.	-2.84; 2.46

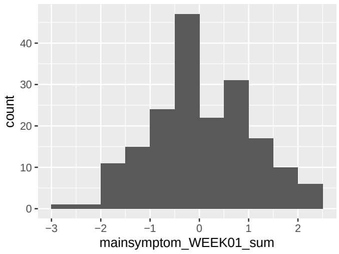

mainsymptom_WEEK01_duration

Feature	Result
Variable type	numeric
Number of missing obs.	0 (0 %)
Number of unique values	163
Median	-0.14
1st and 3rd quartiles	-0.28; 0.05
Min. and max.	-0.62; 3.08

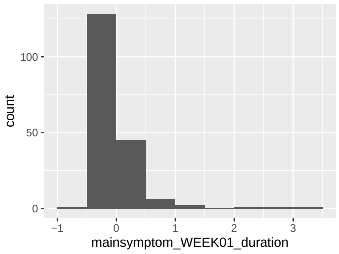

mainsymptom_WEEK01_DateCompleted_day

Feature	Result
Variable type	numeric
Number of missing obs.	0 (0 %)
Number of unique values	32
Median	0.12
1st and 3rd quartiles	-0.4; 1
Min. and max.	-1.37; 1.37

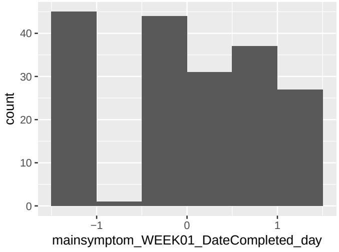

mainsymptom_WEEK01_DateCompleted_time

Feature	Result
Variable type	numeric
Number of missing obs.	0 (0 %)
Number of unique values	169
Median	-0.51
1st and 3rd quartiles	-1.08; 0.25
Min. and max.	-1.41; 1.41

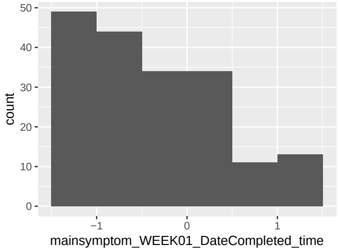

mainsymptom_WEEK02_sum

Feature	Result
Variable type	numeric
Number of missing obs.	0 (0 %)
Number of unique values	99
Median	-0.03
1st and 3rd quartiles	-0.72; 0.74
Min. and max.	-2.75; 2.44

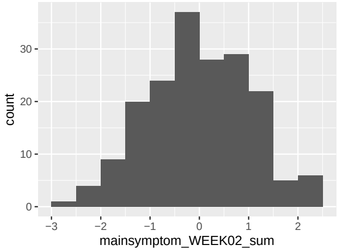

mainsymptom_WEEK02_duration

Feature	Result
Variable type	numeric
Number of missing obs.	0 (0 %)
Number of unique values	147
Median	-0.2
1st and 3rd quartiles	-0.26; -0.1
Min. and max.	-0.4; 20.27

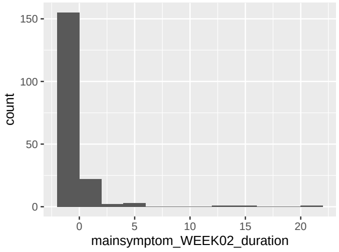

mainsymptom_WEEK02_DateCompleted_day

Feature	Result
Variable type	numeric
Number of missing obs.	0 (0 %)
Number of unique values	24
Median	0.48
1st and 3rd quartiles	-0.37; 1
Min. and max.	-1.37; 1.37

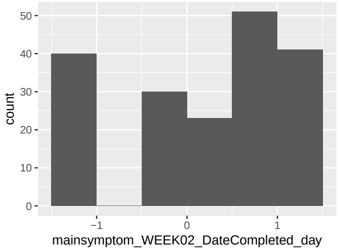

mainsymptom_WEEK02_DateCompleted_time

Feature	Result
Variable type	numeric
Number of missing obs.	0 (0 %)
Number of unique values	172
Median	-0.64
1st and 3rd quartiles	-1.09; 0.02
Min. and max.	-1.41; 1.4

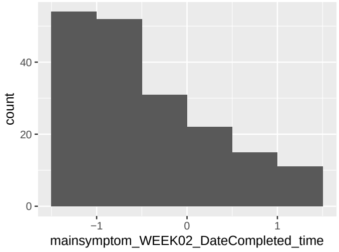

mainsymptom_WEEK03_sum

Feature	Result
Variable type	numeric
Number of missing obs.	0 (0 %)
Number of unique values	98
Median	-0.03
1st and 3rd quartiles	-0.63; 0.74
Min. and max.	-2.64; 2.26

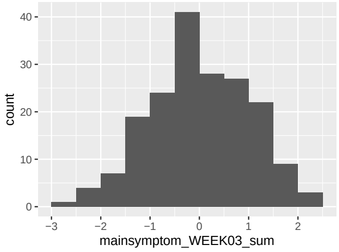

mainsymptom_WEEK03_duration

Feature	Result
Variable type	numeric
Number of missing obs.	0 (0 %)
Number of unique values	155
Median	-0.13
1st and 3rd quartiles	-0.17; -0.03
Min. and max.	-0.23; 6.32

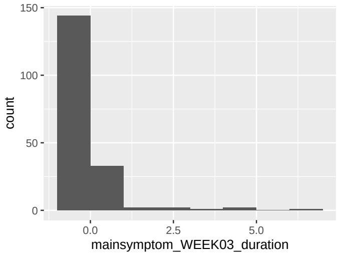

mainsymptom_WEEK03_DateCompleted_day

Feature	Result
Variable type	numeric
Number of missing obs.	0 (0 %)
Number of unique values	25
Median	0.37
1st and 3rd quartiles	-0.37; 1
Min. and max.	-1.37; 1.37

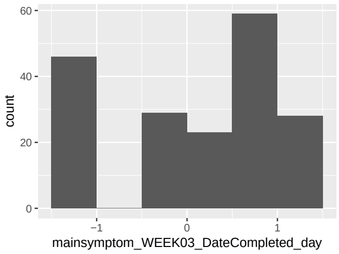

---

mainsymptom_WEEK03_DateCompleted_time

Feature	Result
Variable type	numeric
Number of missing obs.	0 (0 %)
Number of unique values	173
Median	-0.71
1st and 3rd quartiles	-1.14; -0.09
Min. and max.	-1.41; 1.38

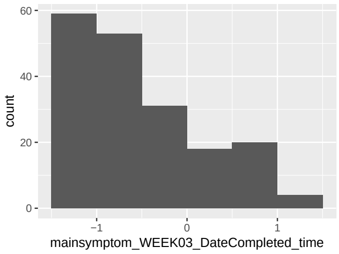

Report generation information:

- Created by: Could not determine from system (username: `nilisa`).
- Report creation time: Mon Jan 09 2023 13:15:33
- Report was run from directory: `/home/nilisa/projects/phd_study1/r`
- dataMaid v1.4.1 [Pkg: 2021-10-08 from CRAN (R 4.2.2)]
- R version 4.2.2 Patched (2022-11-10 r83330).
- Platform: x86_64-pc-linux-gnu (64-bit)(Ubuntu 20.04.5 LTS).
- Function call: `dataMaid::makeDataReport(data = gd, mode = c("summarize", "visualize", "check"), smartNum = FALSE, file = "~/projects/data/study1multiverse/results/graphs_n_figures/codebooks/codebook replace = TRUE, openResult = FALSE, checks = list(character = "showAllFactorLevels", factor = "showAllFactorLevels", labelled = "showAllFactorLevels",   haven_labelled = "showAllFactorLevels", numeric = NULL,   integer = NULL, logical = NULL, Date = NULL), listChecks = FALSE, maxProbVals = Inf, codebook = TRUE, reportTitle = "Handpicked_Social_week04-imput`

Handpicked_Social_week04-imputed_train

Autogenerated data summary from dataMaid

2023-01-09 13:09:05

Data report overview

The dataset examined has the following dimensions:

Feature	Result
Number of observations	1667
Number of variables	63

Codebook summary table

Label	Variable	Class	# unique values	Missing	Description
	**sex**	factor	2	0.00 %	Sex of patient, 0 = Female, 1=Male
	**age**	numeric	58	0.00 %	
	**messages_len_7**	numeric	303	0.00 %	-Meta information of messages-Length of messages-up until day-7
	**messages_len_tp_7**	numeric	714	0.00 %	-Meta information of messages-Length of messages-therapist messages-up until day-7
	**messages_7**	numeric	8	0.00 %	-Meta information of messages-up until day-7
	**messages_tp_7**	numeric	7	0.00 %	-Meta information of messages-therapist messages-up until day-7
	**homeworks_7**	numeric	7	0.00 %	-Number of homework messages sent in-up until day-7
	**messages_len_14**	numeric	339	0.00 %	-Meta information of messages-Length of messages-up until day-14
	**messages_len_tp_14**	numeric	751	0.00 %	-Meta information of messages-Length of messages-therapist messages-up until day-14
	**messages_14**	numeric	9	0.00 %	-Meta information of messages-up until day-14
	**messages_tp_14**	numeric	7	0.00 %	-Meta information of messages-therapist messages-up until day-14

Label	Variable	Class	# unique values	Missing	Description
	**homeworks_14**	numeric	6	0.00 %	-Number of homework messages sent in-up until day-14
	**messages_len_21**	numeric	341	0.00 %	-Meta information of messages-Length of messages-up until day-21
	**messages_len_tp_21**	numeric	714	0.00 %	-Meta information of messages-Length of messages-therapist messages-up until day-21
	**messages_21**	numeric	8	0.00 %	-Meta information of messages-up until day-21
	**messages_tp_21**	numeric	6	0.00 %	-Meta information of messages-therapist messages-up until day-21
	**homeworks_21**	numeric	5	0.00 %	-Number of homework messages sent in-up until day-21
	**messages_len_28**	numeric	341	0.00 %	-Meta information of messages-Length of messages-up until day-28
	**messages_len_tp_28**	numeric	693	0.00 %	-Meta information of messages-Length of messages-therapist messages-up until day-28
	**messages_28**	numeric	8	0.00 %	-Meta information of messages-up until day-28
	**messages_tp_28**	numeric	5	0.00 %	-Meta information of messages-therapist messages-up until day-28
	**homeworks_28**	numeric	6	0.00 %	-Number of homework messages sent in-up until day-28
	**PDSS-SR-3064_SCREEN_sum**	numeric	106	0.00 %	Anxiety questionnaire, self rated-Timepoint before treatment starts-Sum of the entire measure
	**MADRS-1951_SCREEN_sum**	numeric	121	0.00 %	Depression questionnaire, self rated-Timepoint before treatment starts-Sum of the entire measure
	**LSAS-2241_SCREEN_sum**	numeric	203	0.00 %	Social anxiety questionnaire, self rated-Timepoint before treatment starts-Sum of the entire measure
	**MADRS-1951_SCREEN_DateCompleted_day**	numeric	20	0.00 %	Depression questionnaire, self rated-Timepoint before treatment starts-Cyclic transformation of what day 0-6 during week it was filled in

Label	Variable	Class	# unique values	Missing	Description
	**MADRS-1951_SCREEN_DateCompleted_time**	numeric	836	0.00 %	Depression questionnaire, self rated-Timepoint before treatment starts-Cyclic transformation of what time during day 0-1440 it was filled in
	**PDSS-SR-3064_SCREEN_DateCompleted_day**	numeric	39	0.00 %	Anxiety questionnaire, self rated-Timepoint before treatment starts-Cyclic transformation of what day 0-6 during week it was filled in
	**PDSS-SR-3064_SCREEN_DateCompleted_time**	numeric	816	0.00 %	Anxiety questionnaire, self rated-Timepoint before treatment starts-Cyclic transformation of what time during day 0-1440 it was filled in
	**LSAS-2241_SCREEN_DateCompleted_day**	numeric	21	0.00 %	Social anxiety questionnaire, self rated-Timepoint before treatment starts-Cyclic transformation of what day 0-6 during week it was filled in
	**LSAS-2241_SCREEN_DateCompleted_time**	numeric	832	0.00 %	Social anxiety questionnaire, self rated-Timepoint before treatment starts-Cyclic transformation of what time during day 0-1440 it was filled in
	**outcome**	numeric	535	0.00 %	
	**ncomorbid**	numeric	73	0.00 %	
	**HW-01**	numeric	1576	0.00 %	
	**HW-02**	numeric	1335	0.00 %	
	**HW-03**	numeric	1649	0.00 %	
	**currentwork_proff**	factor	51	0.00 %	Currently in work for trained proffession
	**Marital_1833_gift**	factor	2	0.00 %	Marital status: Married or not
	**Marital_1833_separerad**	factor	2	0.00 %	Marital status: divocered/equivalent
	**Marital_1833_singel**	factor	2	0.00 %	Marital status: single
	**Edu_1843_2**	factor	2	0.00 %	7-9 years education
	**Edu_1843_3**	factor	2	0.00 %	Uncompleted upper secondary school
	**Edu_1843_4**	factor	2	0.00 %	Higher vocational education
	**Edu_1843_5**	factor	2	0.00 %	Completed upper secondary school
	**Edu_1843_6**	factor	2	0.00 %	Uncompleted university degree
	**Edu_1843_7**	factor	2	0.00 %	University degree
	**cscale**	numeric	212	0.00 %	
	**mainsymptom_PRE_sum**	numeric	258	0.00 %	PDSS-SR for panic, MADRS for depression, LSAS for social anxiety-Timepoint just before beginning treatment-Sum of the entire measure

Label	Variable	Class	# unique values	Missing	Description
	**mainsymptom_PRE_duration**	numeric	845	0.00 %	PDSS-SR for panic, MADRS for depression, LSAS for social anxiety-Timepoint just before beginning treatment-Time to fill in measure/questionnaire
	**mainsymptom_PRE_DateCompleted_day**	numeric	139	0.00 %	PDSS-SR for panic, MADRS for depression, LSAS for social anxiety-Timepoint just before beginning treatment-Cyclic transformation of what day 0-6 during week it was filled in
	**mainsymptom_PRE_DateCompleted_time**	numeric	857	0.00 %	PDSS-SR for panic, MADRS for depression, LSAS for social anxiety-Timepoint just before beginning treatment-Cyclic transformation of what time during day 0-1440 it was filled in
	**mainsymptom_WEEK01_sum**	numeric	410	0.00 %	PDSS-SR for panic, MADRS for depression, LSAS for social anxiety-Timepoint after one week in treatment-Sum of the entire measure
	**mainsymptom_WEEK01_duration**	numeric	773	0.00 %	PDSS-SR for panic, MADRS for depression, LSAS for social anxiety-Timepoint after one week in treatment-Time to fill in measure/questionnaire
	**mainsymptom_WEEK01_DateCompleted_day**	numeric	282	0.00 %	PDSS-SR for panic, MADRS for depression, LSAS for social anxiety-Timepoint after one week in treatment-Cyclic transformation of what day 0-6 during week it was filled in
	**mainsymptom_WEEK01_DateCompleted_time**	numeric	1007	0.00 %	PDSS-SR for panic, MADRS for depression, LSAS for social anxiety-Timepoint after one week in treatment-Cyclic transformation of what time during day 0-1440 it was filled in
	**mainsymptom_WEEK02_sum**	numeric	354	0.00 %	PDSS-SR for panic, MADRS for depression, LSAS for social anxiety-Timepoint after two weeks in treatment-Sum of the entire measure
	**mainsymptom_WEEK02_duration**	numeric	708	0.00 %	PDSS-SR for panic, MADRS for depression, LSAS for social anxiety-Timepoint after two weeks in treatment-Time to fill in measure/questionnaire

Label	Variable	Class	# unique values	Missing	Description
	**mainsymptom_WEEK02_DateCompleted_day**	numeric	231	0.00 %	PDSS-SR for panic, MADRS for depression, LSAS for social anxiety-Timepoint after two weeks in treatment-Cyclic transformation of what day 0-6 during week it was filled in
	**mainsymptom_WEEK02_DateCompleted_time**	numeric	965	0.00 %	PDSS-SR for panic, MADRS for depression, LSAS for social anxiety-Timepoint after two weeks in treatment-Cyclic transformation of what time during day 0-1440 it was filled in
	**mainsymptom_WEEK03_sum**	numeric	370	0.00 %	PDSS-SR for panic, MADRS for depression, LSAS for social anxiety-Timepoint after three weeks in treatment-Sum of the entire measure
	**mainsymptom_WEEK03_duration**	numeric	711	0.00 %	PDSS-SR for panic, MADRS for depression, LSAS for social anxiety-Timepoint after three weeks in treatment-Time to fill in measure/questionnaire
	**mainsymptom_WEEK03_DateCompleted_day**	numeric	248	0.00 %	PDSS-SR for panic, MADRS for depression, LSAS for social anxiety-Timepoint after three weeks in treatment-Cyclic transformation of what day 0-6 during week it was filled in
	**mainsymptom_WEEK03_DateCompleted_time**	numeric	964	0.00 %	PDSS-SR for panic, MADRS for depression, LSAS for social anxiety-Timepoint after three weeks in treatment-Cyclic transformation of what time during day 0-1440 it was filled in

Variable list

sex

Feature	Result
Variable type	factor
Number of missing obs.	0 (0 %)
Number of unique values	2
Mode	“0”
Reference category	0

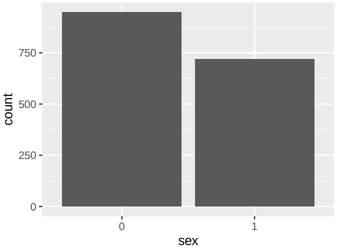

- Observed factor levels: "0", "1".

---

age

Feature	Result
Variable type	numeric
Number of missing obs.	0 (0 %)
Number of unique values	58
Median	-0.46
1st and 3rd quartiles	-0.9; 0.24
Min. and max.	-1.69; 4.25

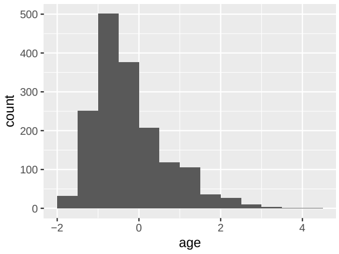

---

messages_len_7

Feature	Result
Variable type	numeric
Number of missing obs.	0 (0 %)
Number of unique values	303
Median	-0.32
1st and 3rd quartiles	-0.32; -0.16
Min. and max.	-0.32; 11.06

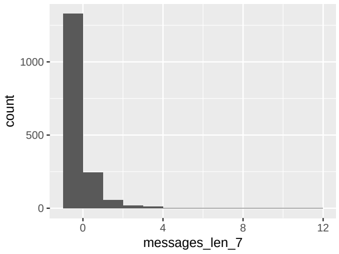

---

messages_len_tp_7

Feature	Result
Variable type	numeric
Number of missing obs.	0 (0 %)
Number of unique values	714
Median	0.04
1st and 3rd quartiles	-0.54; 0.84
Min. and max.	-1.46; 5.25

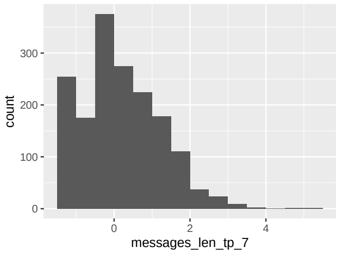

---

messages_7

Feature	Result
Variable type	numeric
Number of missing obs.	0 (0 %)
Number of unique values	8
Median	-0.55
1st and 3rd quartiles	-0.55; 0.58
Min. and max.	-0.55; 8.54

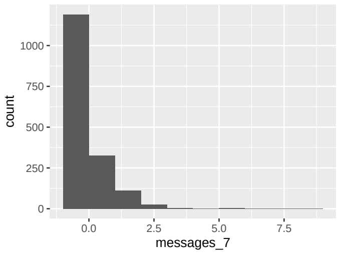

---

messages_tp_7

Feature	Result
Variable type	numeric
Number of missing obs.	0 (0 %)
Number of unique values	7
Median	0.15
1st and 3rd quartiles	-0.91; 0.15
Min. and max.	-1.97; 4.4

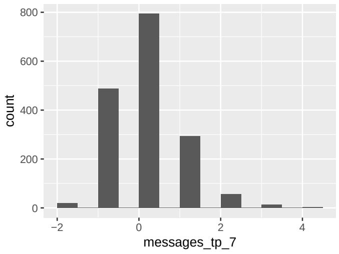

homeworks_7

Feature	Result
Variable type	numeric
Number of missing obs.	0 (0 %)
Number of unique values	7
Median	0.2
1st and 3rd quartiles	-1.03; 0.2
Min. and max.	-1.03; 6.39

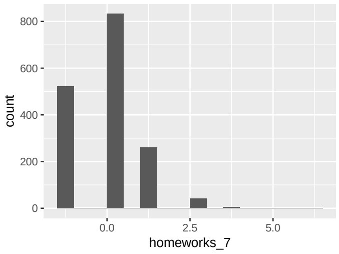

messages_len_14

Feature	Result
Variable type	numeric
Number of missing obs.	0 (0 %)
Number of unique values	339
Median	-0.41
1st and 3rd quartiles	-0.41; -0.05
Min. and max.	-0.41; 12.82

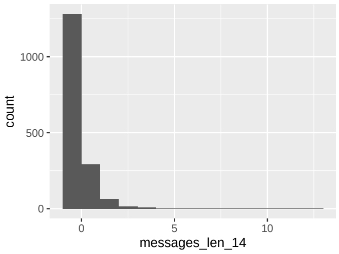

messages_len_tp_14

Feature	Result
Variable type	numeric
Number of missing obs.	0 (0 %)
Number of unique values	751
Median	-0.11
1st and 3rd quartiles	-0.73; 0.59
Min. and max.	-1.11; 5.97

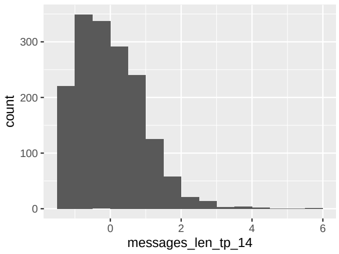

messages_14

Feature	Result
Variable type	numeric
Number of missing obs.	0 (0 %)
Number of unique values	9
Median	-0.7
1st and 3rd quartiles	-0.7; 0.36
Min. and max.	-0.7; 9.9

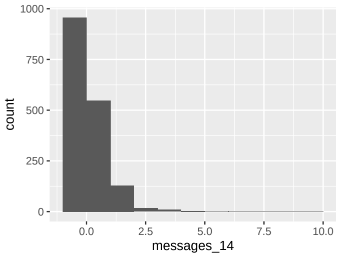

messages_tp_14

Feature	Result
Variable type	numeric
Number of missing obs.	0 (0 %)
Number of unique values	7
Median	-0.35
1st and 3rd quartiles	-0.35; 0.87
Min. and max.	-1.57; 5.75

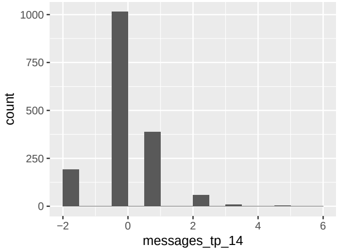

homeworks_14

Feature	Result
Variable type	numeric
Number of missing obs.	0 (0 %)
Number of unique values	6
Median	0.33
1st and 3rd quartiles	-0.98; 0.33
Min. and max.	-0.98; 5.58

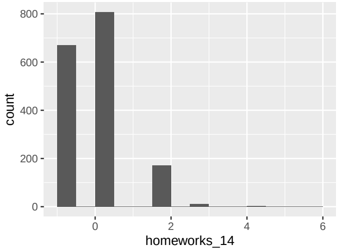

messages_len_21

Feature	Result
Variable type	numeric
Number of missing obs.	0 (0 %)
Number of unique values	341
Median	-0.46
1st and 3rd quartiles	-0.46; -0.02
Min. and max.	-0.46; 13.29

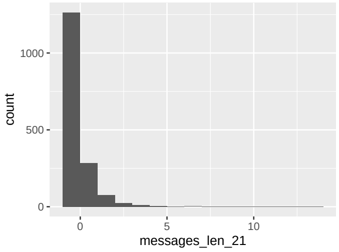

messages_len_tp_21

Feature	Result
Variable type	numeric
Number of missing obs.	0 (0 %)
Number of unique values	714
Median	-0.17
1st and 3rd quartiles	-0.77; 0.59
Min. and max.	-1.01; 4.87

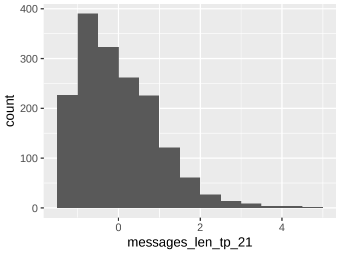

messages_21

Feature	Result
Variable type	numeric
Number of missing obs.	0 (0 %)
Number of unique values	8
Median	-0.74
1st and 3rd quartiles	-0.74; 0.34
Min. and max.	-0.74; 6.83

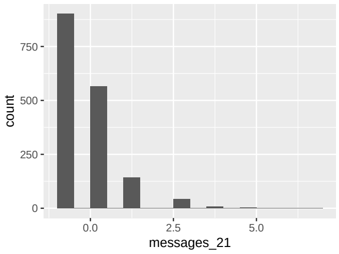

messages_tp_21

Feature	Result
Variable type	numeric
Number of missing obs.	0 (0 %)
Number of unique values	6
Median	-0.32
1st and 3rd quartiles	-0.32; 0.84
Min. and max.	-1.49; 4.33

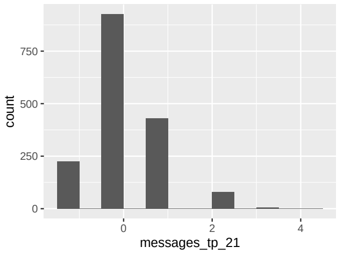

homeworks_21

Feature	Result
Variable type	numeric
Number of missing obs.	0 (0 %)
Number of unique values	5
Median	0.36
1st and 3rd quartiles	-1.01; 0.36
Min. and max.	-1.01; 4.49

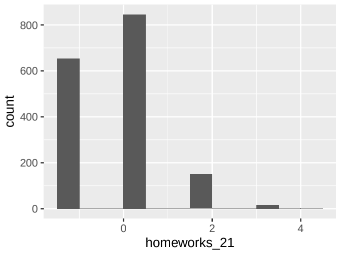

messages_len_28

Feature	Result
Variable type	numeric
Number of missing obs.	0 (0 %)
Number of unique values	341
Median	-0.41
1st and 3rd quartiles	-0.41; 0.02
Min. and max.	-0.41; 10.45

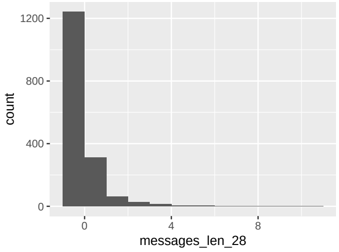

messages_len_tp_28

Feature	Result
Variable type	numeric
Number of missing obs.	0 (0 %)
Number of unique values	693
Median	-0.18
1st and 3rd quartiles	-0.74; 0.54
Min. and max.	-0.94; 9.1

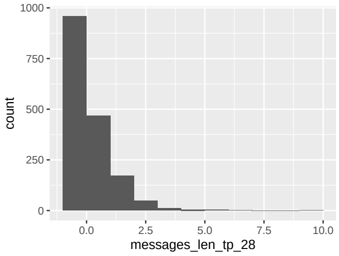

messages_28

Feature	Result
Variable type	numeric
Number of missing obs.	0 (0 %)
Number of unique values	8
Median	-0.72
1st and 3rd quartiles	-0.72; 0.41
Min. and max.	-0.72; 7.2

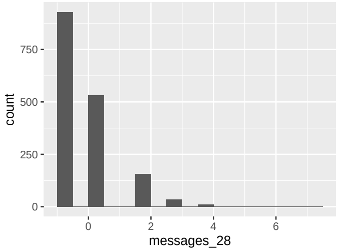

messages_tp_28

Feature	Result
Variable type	numeric
Number of missing obs.	0 (0 %)
Number of unique values	5
Median	-0.24
1st and 3rd quartiles	-0.24; 0.93
Min. and max.	-1.42; 3.28

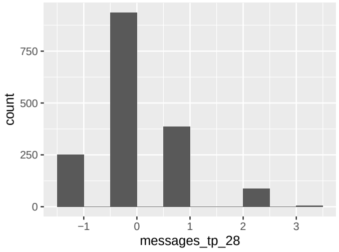

homeworks_28

Feature	Result
Variable type	numeric
Number of missing obs.	0 (0 %)
Number of unique values	6
Median	0.46
1st and 3rd quartiles	-0.91; 0.46
Min. and max.	-0.91; 5.94

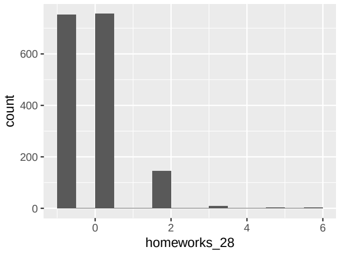

PDSS-SR-3064_SCREEN_sum

Feature	Result
Variable type	numeric
Number of missing obs.	0 (0 %)
Number of unique values	106
Median	-0.19
1st and 3rd quartiles	-0.98; 0.49
Min. and max.	-1.29; 2.8

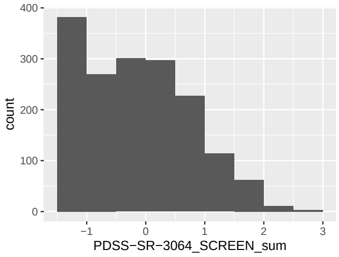

MADRS-1951_SCREEN_sum

Feature	Result
Variable type	numeric
Number of missing obs.	0 (0 %)
Number of unique values	121
Median	-0.45
1st and 3rd quartiles	-1.19; 0.17
Min. and max.	-2.68; 2.28

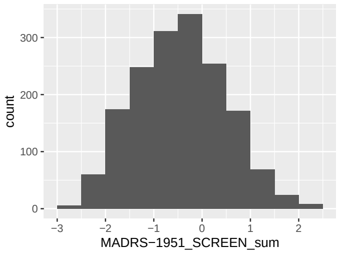

LSAS-2241_SCREEN_sum

Feature	Result
Variable type	numeric
Number of missing obs.	0 (0 %)
Number of unique values	203
Median	0.67
1st and 3rd quartiles	0.12; 1.24
Min. and max.	-1.44; 3.09

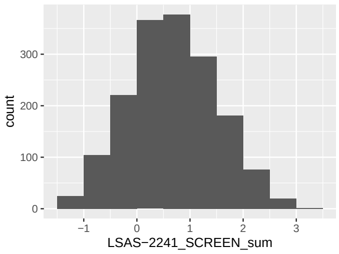

MADRS-1951_SCREEN_DateCompleted_day

Feature	Result
Variable type	numeric
Number of missing obs.	0 (0 %)
Number of unique values	20
Median	0.37
1st and 3rd quartiles	-1; 1
Min. and max.	-1.37; 1.37

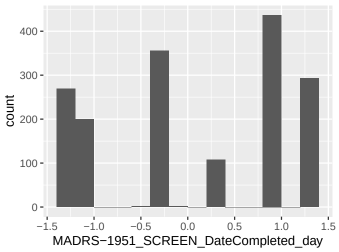

MADRS-1951_SCREEN_DateCompleted_time

Feature	Result
Variable type	numeric
Number of missing obs.	0 (0 %)
Number of unique values	836
Median	-0.61
1st and 3rd quartiles	-1.2; 0.28
Min. and max.	-1.41; 1.41

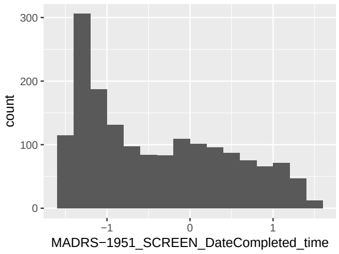

PDSS-SR-3064_SCREEN_DateCompleted_day

Feature	Result
Variable type	numeric
Number of missing obs.	0 (0 %)
Number of unique values	39
Median	0.37
1st and 3rd quartiles	-1; 1
Min. and max.	-1.37; 1.37

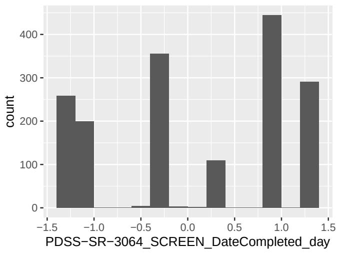

PDSS-SR-3064_SCREEN_DateCompleted_time

Feature	Result
Variable type	numeric
Number of missing obs.	0 (0 %)
Number of unique values	816
Median	-0.58
1st and 3rd quartiles	-1.22; 0.31
Min. and max.	-1.41; 1.41

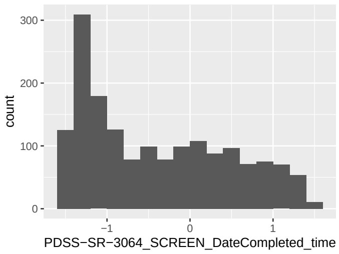

LSAS-2241_SCREEN_DateCompleted_day

Feature	Result
Variable type	numeric
Number of missing obs.	0 (0 %)
Number of unique values	21
Median	-0.16
1st and 3rd quartiles	-1; 1
Min. and max.	-1.37; 1.37

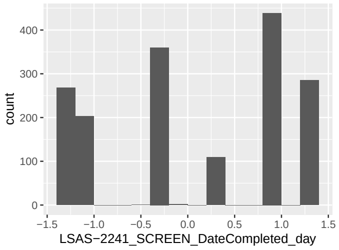

LSAS-2241_SCREEN_DateCompleted_time

Feature	Result
Variable type	numeric
Number of missing obs.	0 (0 %)
Number of unique values	832
Median	-0.55
1st and 3rd quartiles	-1.2; 0.35
Min. and max.	-1.41; 1.41

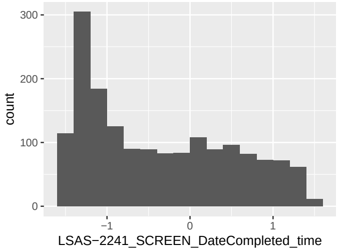

outcome

Feature	Result
Variable type	numeric
Number of missing obs.	0 (0 %)
Number of unique values	535
Median	-0.01
1st and 3rd quartiles	-0.64; 0.62
Min. and max.	-2.05; 3.53

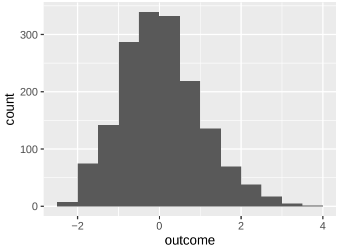

ncomorbid

Feature	Result
Variable type	numeric
Number of missing obs.	0 (0 %)
Number of unique values	73
Median	0
1st and 3rd quartiles	0; 1
Min. and max.	0; 5

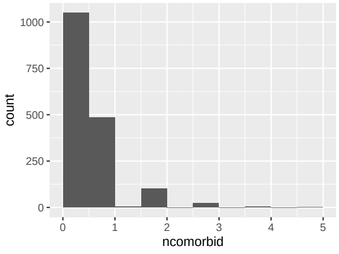

HW-01

Feature	Result
Variable type	numeric
Number of missing obs.	0 (0 %)
Number of unique values	1576
Median	-0.31
1st and 3rd quartiles	-0.81; 0.48
Min. and max.	-1.92; 3.37

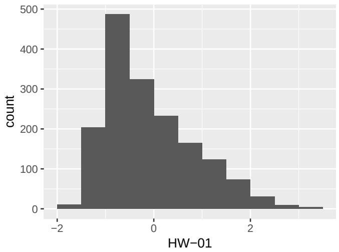

HW-02

Feature	Result
Variable type	numeric
Number of missing obs.	0 (0 %)
Number of unique values	1335
Median	-0.28
1st and 3rd quartiles	-0.54; 0.28
Min. and max.	-1.42; 10.79

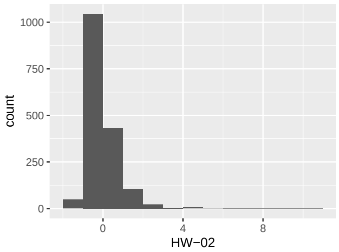

HW-03

Feature	Result
Variable type	numeric
Number of missing obs.	0 (0 %)
Number of unique values	1649
Median	0
1st and 3rd quartiles	-0.22; 0.15
Min. and max.	-4.83; 0.62

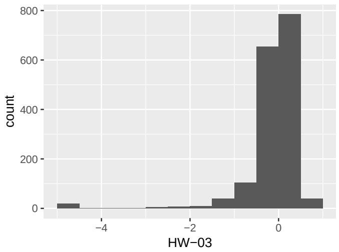

---

currentwork_proff

Feature	Result
Variable type	factor
Number of missing obs.	0 (0 %)
Number of unique values	51
Mode	"1"
Reference category	0

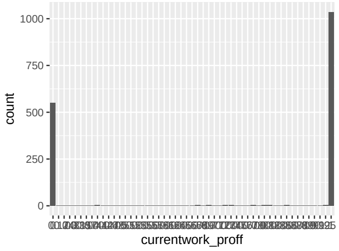

- Observed factor levels: "0", "0.17", "0.24", "0.3", "0.31", "0.35", "0.37", "0.4", "0.41", "0.42", "0.47", "0.48", "0.5", "0.51", "0.52", "0.53", "0.55", "0.56", "0.57", "0.58", "0.61", "0.62", "0.64", "0.65", "0.66", "0.67", "0.68", "0.69", "0.7", "0.71", "0.72", "0.73", "0.74", "0.75", "0.76", "0.77", "0.78", "0.8", "0.81", "0.82", "0.83", "0.85", "0.86", "0.87", "0.88", "0.89", "0.9", "0.91", "0.92", "0.95", "1".

Marital_1833_gift

Feature	Result
Variable type	factor
Number of missing obs.	0 (0 %)
Number of unique values	2
Mode	“1”
Reference category	0

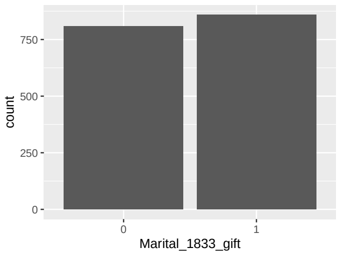

- Observed factor levels: "0", "1".

Marital_1833_separerad

Feature	Result
Variable type	factor
Number of missing obs.	0 (0 %)
Number of unique values	2
Mode	“0”
Reference category	0

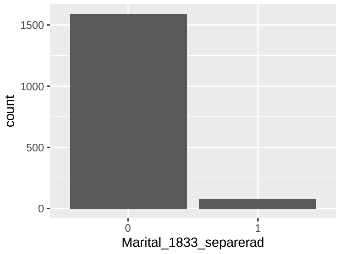

- Observed factor levels: "0", "1".

Marital_1833_singel

Feature	Result
Variable type	factor
Number of missing obs.	0 (0 %)
Number of unique values	2
Mode	“0”
Reference category	0

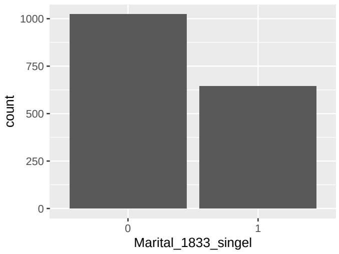

- Observed factor levels: "0", "1".

Edu_1843_2

Feature	Result
Variable type	factor
Number of missing obs.	0 (0 %)
Number of unique values	2
Mode	“0”
Reference category	0

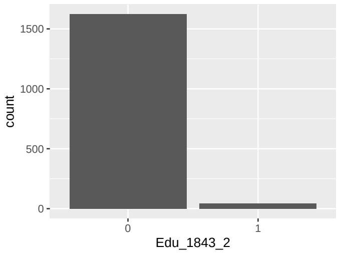

- Observed factor levels: "0", "1".

Edu_1843_3

Feature	Result
Variable type	factor
Number of missing obs.	0 (0 %)
Number of unique values	2
Mode	“0”
Reference category	0

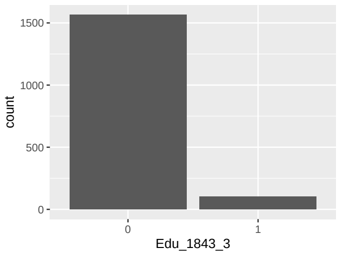

- Observed factor levels: "0", "1".

Edu_1843_4

Feature	Result
Variable type	factor
Number of missing obs.	0 (0 %)
Number of unique values	2
Mode	“0”
Reference category	0

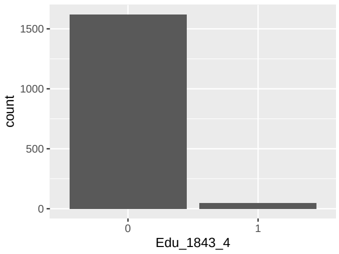

- Observed factor levels: "0", "1".

Edu_1843_5

Feature	Result
Variable type	factor
Number of missing obs.	0 (0 %)
Number of unique values	2
Mode	“0”
Reference category	0

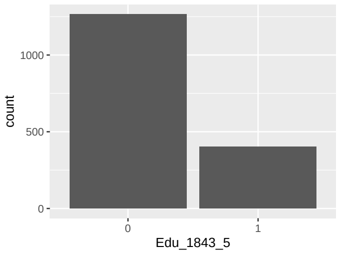

- Observed factor levels: "0", "1".

Edu_1843_6

Feature	Result
Variable type	factor
Number of missing obs.	0 (0 %)
Number of unique values	2
Mode	“0”
Reference category	0

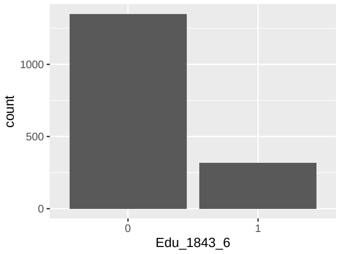

- Observed factor levels: "0", "1".

Edu_1843_7

Feature	Result
Variable type	factor
Number of missing obs.	0 (0 %)
Number of unique values	2
Mode	"0"
Reference category	0

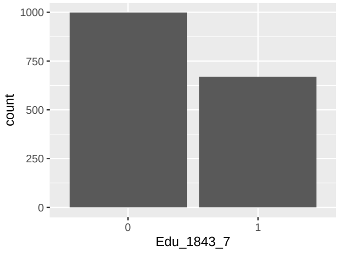

- Observed factor levels: "0", "1".

---

cscale

Feature	Result
Variable type	numeric
Number of missing obs.	0 (0 %)
Number of unique values	212
Median	0.01
1st and 3rd quartiles	-0.46; 0.6
Min. and max.	-4.14; 1.79

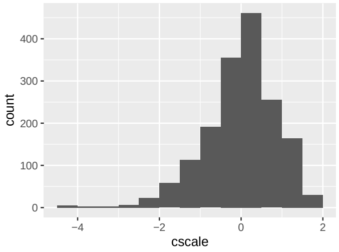

---

mainsymptom_PRE_sum

Feature	Result
Variable type	numeric
Number of missing obs.	0 (0 %)
Number of unique values	258
Median	0
1st and 3rd quartiles	-0.68; 0.63
Min. and max.	-2.7; 3

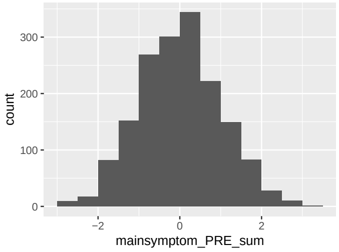

---

mainsymptom_PRE_duration

Feature	Result
Variable type	numeric
Number of missing obs.	0 (0 %)
Number of unique values	845
Median	-0.15
1st and 3rd quartiles	-0.25; 0.02
Min. and max.	-0.44; 33.12

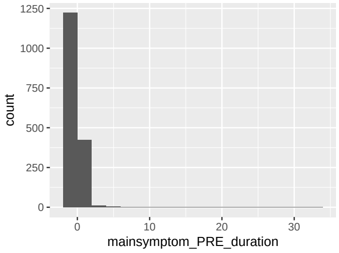

---

mainsymptom_PRE_DateCompleted_day

Feature	Result
Variable type	numeric
Number of missing obs.	0 (0 %)
Number of unique values	139
Median	0.37
1st and 3rd quartiles	-0.37; 1
Min. and max.	-1.37; 1.37

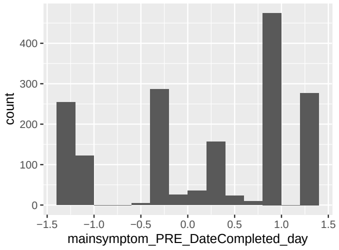

mainsymptom_PRE_DateCompleted_time

Feature	Result
Variable type	numeric
Number of missing obs.	0 (0 %)
Number of unique values	857
Median	-0.58
1st and 3rd quartiles	-1.15; 0.11
Min. and max.	-1.41; 1.41

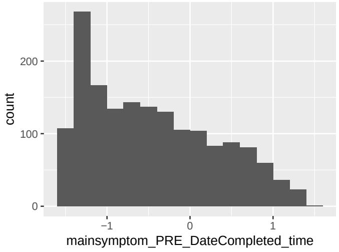

mainsymptom_WEEK01_sum

Feature	Result
Variable type	numeric
Number of missing obs.	0 (0 %)
Number of unique values	410
Median	-0.02
1st and 3rd quartiles	-0.7; 0.62
Min. and max.	-2.84; 3.08

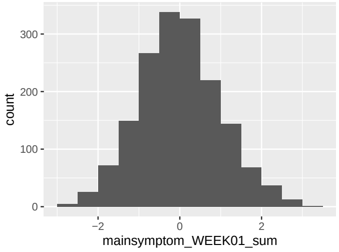

mainsymptom_WEEK01_duration

Feature	Result
Variable type	numeric
Number of missing obs.	0 (0 %)
Number of unique values	773
Median	-0.16
1st and 3rd quartiles	-0.27; 0.04
Min. and max.	-0.62; 20.06

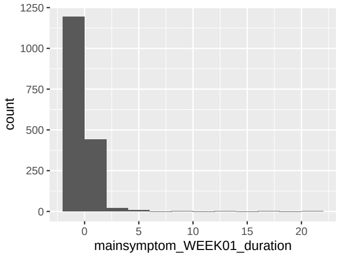

mainsymptom_WEEK01_DateCompleted_day

Feature	Result
Variable type	numeric
Number of missing obs.	0 (0 %)
Number of unique values	282
Median	0.37
1st and 3rd quartiles	-0.37; 1
Min. and max.	-1.37; 1.37

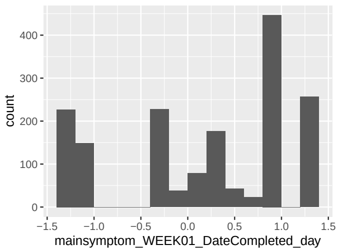

mainsymptom_WEEK01_DateCompleted_time

Feature	Result
Variable type	numeric
Number of missing obs.	0 (0 %)
Number of unique values	1007
Median	-0.57
1st and 3rd quartiles	-1.09; 0.07
Min. and max.	-1.41; 1.41

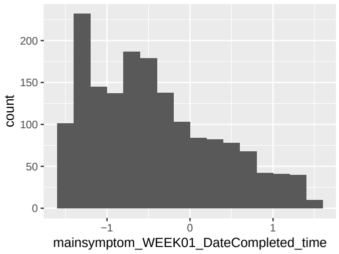

mainsymptom_WEEK02_sum

Feature	Result
Variable type	numeric
Number of missing obs.	0 (0 %)
Number of unique values	354
Median	-0.03
1st and 3rd quartiles	-0.68; 0.66
Min. and max.	-2.75; 3.01

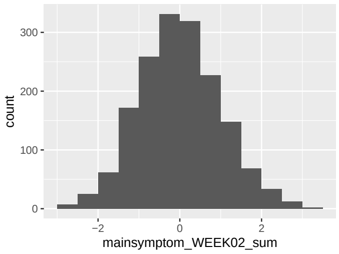

mainsymptom_WEEK02_duration

Feature	Result
Variable type	numeric
Number of missing obs.	0 (0 %)
Number of unique values	708
Median	-0.17
1st and 3rd quartiles	-0.24; -0.03
Min. and max.	-0.46; 11.6

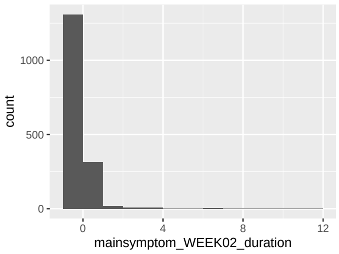

mainsymptom_WEEK02_DateCompleted_day

Feature	Result
Variable type	numeric
Number of missing obs.	0 (0 %)
Number of unique values	231
Median	0.37
1st and 3rd quartiles	-0.37; 1
Min. and max.	-1.37; 1.37

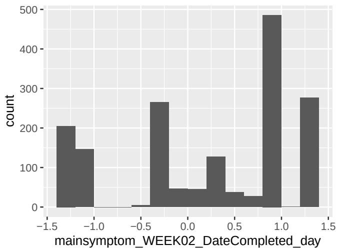

mainsymptom_WEEK02_DateCompleted_time

Feature	Result
Variable type	numeric
Number of missing obs.	0 (0 %)
Number of unique values	965
Median	-0.56
1st and 3rd quartiles	-1.1; 0.07
Min. and max.	-1.41; 1.41

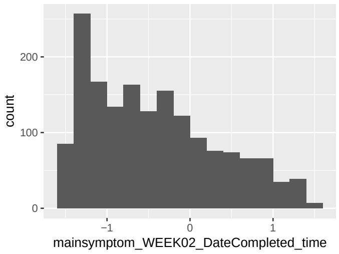

mainsymptom_WEEK03_sum

Feature	Result
Variable type	numeric
Number of missing obs.	0 (0 %)
Number of unique values	370
Median	-0.03
1st and 3rd quartiles	-0.67; 0.7
Min. and max.	-2.64; 3.07

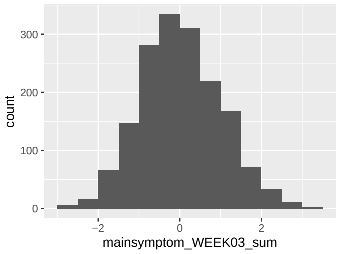

mainsymptom_WEEK03_duration

Feature	Result
Variable type	numeric
Number of missing obs.	0 (0 %)
Number of unique values	711
Median	-0.12
1st and 3rd quartiles	-0.16; -0.05
Min. and max.	-0.28; 28.79

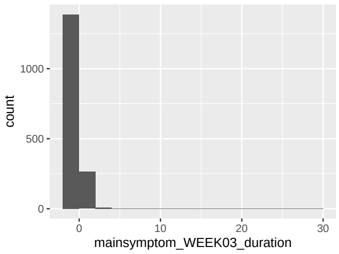

mainsymptom_WEEK03_DateCompleted_day

Feature	Result
Variable type	numeric
Number of missing obs.	0 (0 %)
Number of unique values	248
Median	0.37
1st and 3rd quartiles	-0.37; 1
Min. and max.	-1.37; 1.37

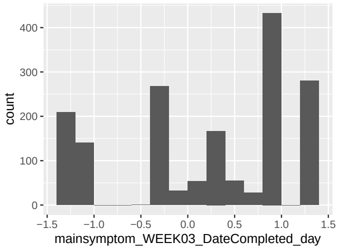

---

mainsymptom_WEEK03_DateCompleted_time

Feature	Result
Variable type	numeric
Number of missing obs.	0 (0 %)
Number of unique values	964
Median	-0.51
1st and 3rd quartiles	-1.1; 0.12
Min. and max.	-1.41; 1.41

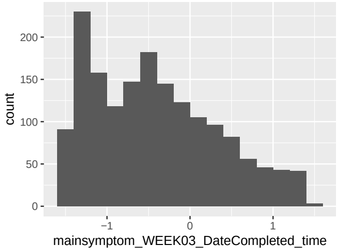

Report generation information:

- Created by: Could not determine from system (username: `nilisa`).
- Report creation time: Mon Jan 09 2023 13:09:05
- Report was run from directory: `/home/nilisa/projects/phd_study1/r`
- dataMaid v1.4.1 [Pkg: 2021-10-08 from CRAN (R 4.2.2)]
- R version 4.2.2 Patched (2022-11-10 r83330).
- Platform: x86_64-pc-linux-gnu (64-bit)(Ubuntu 20.04.5 LTS).
- Function call: `dataMaid::makeDataReport(data = gd, mode = c("summarize", "visualize", "check"), smartNum = FALSE, file = "~/projects/data/study1multiverse/results/graphs_n_figures/codebooks/codebook replace = TRUE, openResult = FALSE, checks = list(character = "showAllFactorLevels", factor = "showAllFactorLevels", labelled = "showAllFactorLevels",   haven_labelled = "showAllFactorLevels", numeric = NULL,   integer = NULL, logical = NULL, Date = NULL), listChecks = FALSE, maxProbVals = Inf, codebook = TRUE, reportTitle = "Handpicked_Social_week04-imput`

Handpicked_Social_week04-naremove_benchmark_test

Autogenerated data summary from dataMaid

2023-01-09 13:16:12

Data report overview

The dataset examined has the following dimensions:

Feature	Result
Number of observations	113
Number of variables	10

Codebook summary table

Label	Variable	Class	# unique values	Missing	Description
	**sex**	factor	2	0.00 %	Sex of patient, 0 = Female, 1=Male
	**age**	numeric	35	0.00 %	
	**PDSS-SR-3064_SCREEN_sum**	numeric	22	0.00 %	Anxiety questionnaire, self rated-Timepoint before treatment starts-Sum of the entire measure
	**MADRS-1951_SCREEN_sum**	numeric	34	0.00 %	Depression questionnaire, self rated-Timepoint before treatment starts-Sum of the entire measure
	**LSAS-2241_SCREEN_sum**	numeric	66	0.00 %	Social anxiety questionnaire, self rated-Timepoint before treatment starts-Sum of the entire measure
	**outcome**	numeric	69	0.00 %	
	**mainsymptom_PRE_sum**	numeric	63	0.00 %	PDSS-SR for panic, MADRS for depression, LSAS for social anxiety-Timepoint just before beginning treatment-Sum of the entire measure
	**mainsymptom_WEEK01_sum**	numeric	70	0.00 %	PDSS-SR for panic, MADRS for depression, LSAS for social anxiety-Timepoint after one week in treatment-Sum of the entire measure
	**mainsymptom_WEEK02_sum**	numeric	67	0.00 %	PDSS-SR for panic, MADRS for depression, LSAS for social anxiety-Timepoint after two weeks in treatment-Sum of the entire measure

Label	Variable	Class	# unique values	Missing	Description
	**mainsymptom_WEEK03_sum**	numeric	66	0.00 %	PDSS-SR for panic, MADRS for depression, LSAS for social anxiety-Timepoint after three weeks in treatment-Sum of the entire measure

Variable list

sex

Feature	Result
Variable type	factor
Number of missing obs.	0 (0 %)
Number of unique values	2
Mode	"0"
Reference category	0

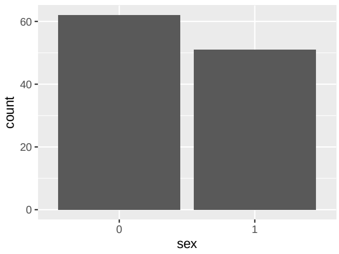

- Observed factor levels: "0", "1".

---

age

Feature	Result
Variable type	numeric
Number of missing obs.	0 (0 %)
Number of unique values	35
Median	-0.46
1st and 3rd quartiles	-0.9; 0.24
Min. and max.	-1.42; 2.51

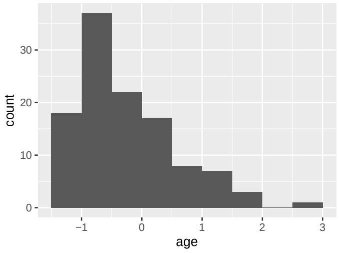

PDSS-SR-3064_SCREEN_sum

Feature	Result
Variable type	numeric
Number of missing obs.	0 (0 %)
Number of unique values	22
Median	-0.35
1st and 3rd quartiles	-0.82; 0.28
Min. and max.	-1.29; 2.49

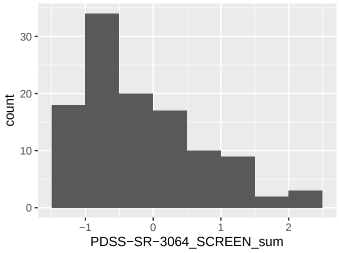

MADRS-1951_SCREEN_sum

Feature	Result
Variable type	numeric
Number of missing obs.	0 (0 %)
Number of unique values	34
Median	-0.45
1st and 3rd quartiles	-1.19; 0.42
Min. and max.	-2.56; 2.03

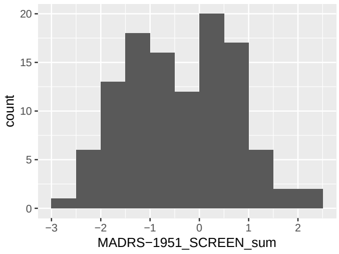

LSAS-2241_SCREEN_sum

Feature	Result
Variable type	numeric
Number of missing obs.	0 (0 %)
Number of unique values	66
Median	0.6
1st and 3rd quartiles	0.12; 1.33
Min. and max.	-1.1; 2.64

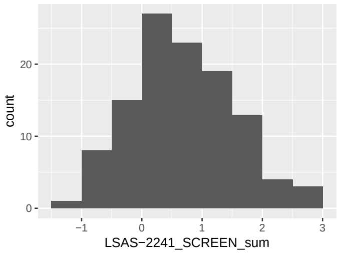

---

outcome

Feature	Result
Variable type	numeric
Number of missing obs.	0 (0 %)
Number of unique values	69
Median	0.03
1st and 3rd quartiles	-0.68; 0.46
Min. and max.	-2.01; 2.79

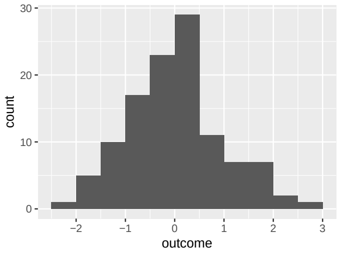

---

mainsymptom_PRE_sum

Feature	Result
Variable type	numeric
Number of missing obs.	0 (0 %)
Number of unique values	63
Median	0
1st and 3rd quartiles	-0.72; 0.85
Min. and max.	-2.45; 2.41

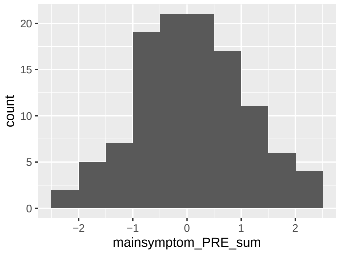

---

mainsymptom_WEEK01_sum

Feature	Result
Variable type	numeric
Number of missing obs.	0 (0 %)
Number of unique values	70
Median	0.04
1st and 3rd quartiles	-0.54; 0.82
Min. and max.	-2.31; 2.46

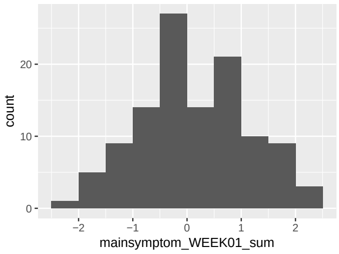

mainsymptom_WEEK02_sum

Feature	Result
Variable type	numeric
Number of missing obs.	0 (0 %)
Number of unique values	67
Median	0.09
1st and 3rd quartiles	-0.64; 0.86
Min. and max.	-2.26; 2.44

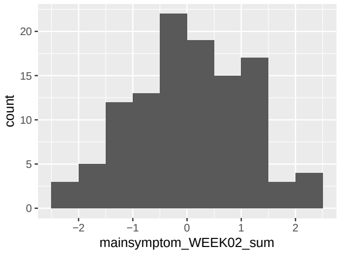

mainsymptom_WEEK03_sum

Feature	Result
Variable type	numeric
Number of missing obs.	0 (0 %)
Number of unique values	66
Median	0.01
1st and 3rd quartiles	-0.63; 0.78
Min. and max.	-2.12; 2.26

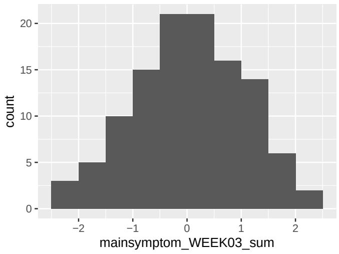

Report generation information:

- Created by: Could not determine from system (username: `nilisa`).
- Report creation time: Mon Jan 09 2023 13:16:12
- Report was run from directory: `/home/nilisa/projects/phd_study1/r`
- dataMaid v1.4.1 [Pkg: 2021-10-08 from CRAN (R 4.2.2)]
- R version 4.2.2 Patched (2022-11-10 r83330).
- Platform: x86_64-pc-linux-gnu (64-bit)(Ubuntu 20.04.5 LTS).
- Function call: `dataMaid::makeDataReport(data = gd, mode = c("summarize", "visualize", "check"), smartNum = FALSE, file = "~/projects/data/study1multiverse/results/graphs_n_figures/codebooks/codebook replace = TRUE, openResult = FALSE, checks = list(character = "showAllFactorLevels", factor = "showAllFactorLevels", labelled = "showAllFactorLevels",   haven_labelled = "showAllFactorLevels", numeric = NULL,   integer = NULL, logical = NULL, Date = NULL), listChecks = FALSE, maxProbVals = Inf, codebook = TRUE, reportTitle = "Handpicked_Social_week04-narem`

Handpicked_Social_week04-naremove_benchmark_train

Autogenerated data summary from dataMaid

2023-01-09 13:09:46

Data report overview

The dataset examined has the following dimensions:

Feature	Result
Number of observations	901
Number of variables	10

Codebook summary table

Label	Variable	Class	# unique values	Missing	Description
	**sex**	factor	2	0.00 %	Sex of patient, 0 = Female, 1=Male
	**age**	numeric	56	0.00 %	
	**PDSS-SR-3064_SCREEN_sum**	numeric	24	0.00 %	Anxiety questionnaire, self rated-Timepoint before treatment starts-Sum of the entire measure
	**MADRS-1951_SCREEN_sum**	numeric	39	0.00 %	Depression questionnaire, self rated-Timepoint before treatment starts-Sum of the entire measure
	**LSAS-2241_SCREEN_sum**	numeric	113	0.00 %	Social anxiety questionnaire, self rated-Timepoint before treatment starts-Sum of the entire measure
	**outcome**	numeric	118	0.00 %	
	**mainsymptom_PRE_sum**	numeric	113	0.00 %	PDSS-SR for panic, MADRS for depression, LSAS for social anxiety-Timepoint just before beginning treatment-Sum of the entire measure
	**mainsymptom_WEEK01_sum**	numeric	117	0.00 %	PDSS-SR for panic, MADRS for depression, LSAS for social anxiety-Timepoint after one week in treatment-Sum of the entire measure
	**mainsymptom_WEEK02_sum**	numeric	122	0.00 %	PDSS-SR for panic, MADRS for depression, LSAS for social anxiety-Timepoint after two weeks in treatment-Sum of the entire measure

Label	Variable	Class	# unique values	Missing	Description
	**mainsymptom_WEEK03_sum**	numeric	120	0.00 %	PDSS-SR for panic, MADRS for depression, LSAS for social anxiety-Timepoint after three weeks in treatment-Sum of the entire measure

Variable list

sex

Feature	Result
Variable type	factor
Number of missing obs.	0 (0 %)
Number of unique values	2
Mode	"0"
Reference category	0

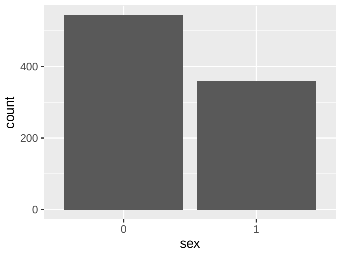

- Observed factor levels: "0", "1".

---

age

Feature	Result
Variable type	numeric
Number of missing obs.	0 (0 %)
Number of unique values	56
Median	-0.46
1st and 3rd quartiles	-0.9; 0.24
Min. and max.	-1.69; 4.25

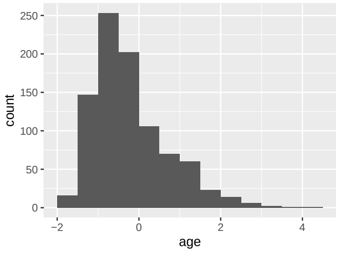

PDSS-SR-3064_SCREEN_sum

Feature	Result
Variable type	numeric
Number of missing obs.	0 (0 %)
Number of unique values	24
Median	-0.19
1st and 3rd quartiles	-0.98; 0.44
Min. and max.	-1.29; 2.8

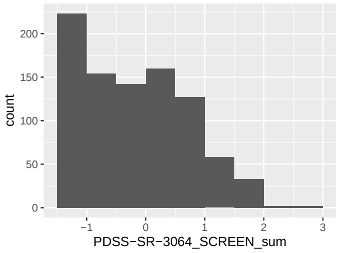

MADRS-1951_SCREEN_sum

Feature	Result
Variable type	numeric
Number of missing obs.	0 (0 %)
Number of unique values	39
Median	-0.57
1st and 3rd quartiles	-1.19; 0.05
Min. and max.	-2.68; 2.28

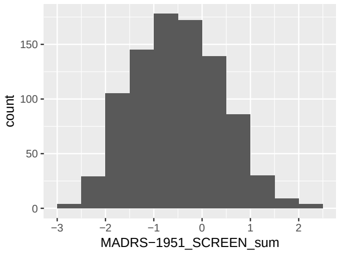

LSAS-2241_SCREEN_sum

Feature	Result
Variable type	numeric
Number of missing obs.	0 (0 %)
Number of unique values	113
Median	0.67
1st and 3rd quartiles	0.08; 1.22
Min. and max.	-1.37; 2.99

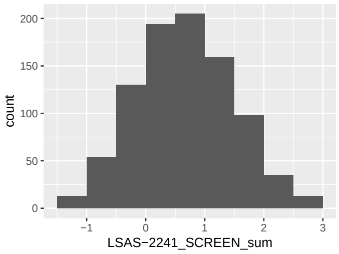

outcome

Feature	Result
Variable type	numeric
Number of missing obs.	0 (0 %)
Number of unique values	118
Median	-0.13
1st and 3rd quartiles	-0.75; 0.54
Min. and max.	-2.05; 3.53

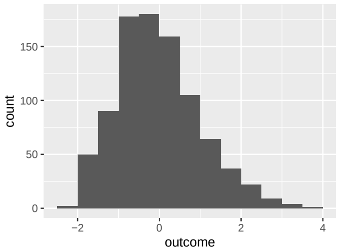

mainsymptom_PRE_sum

Feature	Result
Variable type	numeric
Number of missing obs.	0 (0 %)
Number of unique values	113
Median	-0.04
1st and 3rd quartiles	-0.68; 0.63
Min. and max.	-2.49; 3

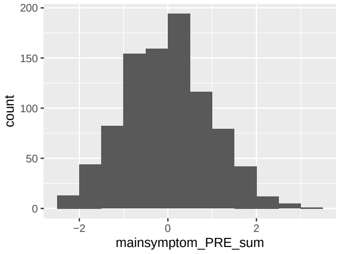

mainsymptom_WEEK01_sum

Feature	Result
Variable type	numeric
Number of missing obs.	0 (0 %)
Number of unique values	117
Median	-0.05
1st and 3rd quartiles	-0.66; 0.57
Min. and max.	-2.84; 2.91

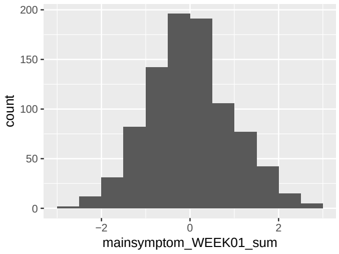

---

mainsymptom_WEEK02_sum

Feature	Result
Variable type	numeric
Number of missing obs.	0 (0 %)
Number of unique values	122
Median	-0.11
1st and 3rd quartiles	-0.72; 0.58
Min. and max.	-2.75; 3.01

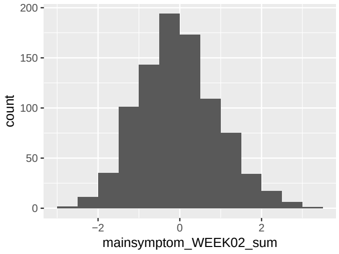

---

mainsymptom_WEEK03_sum

Feature	Result
Variable type	numeric
Number of missing obs.	0 (0 %)
Number of unique values	120
Median	-0.11
1st and 3rd quartiles	-0.71; 0.62
Min. and max.	-2.64; 3.07

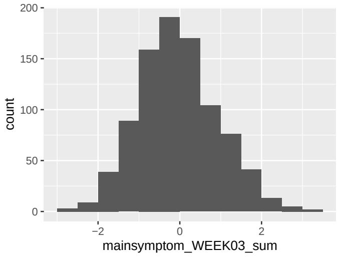

Report generation information:

- Created by: Could not determine from system (username: `nilisa`).
- Report creation time: Mon Jan 09 2023 13:09:46
- Report was run from directory: `/home/nilisa/projects/phd_study1/r`
- dataMaid v1.4.1 [Pkg: 2021-10-08 from CRAN (R 4.2.2)]
- R version 4.2.2 Patched (2022-11-10 r83330).
- Platform: x86_64-pc-linux-gnu (64-bit)(Ubuntu 20.04.5 LTS).
- Function call: `dataMaid::makeDataReport(data = gd, mode = c("summarize", "visualize", "check"), smartNum = FALSE, file = "~/projects/data/study1multiverse/results/graphs_n_figures/codebooks/codebook replace = TRUE, openResult = FALSE, checks = list(character = "showAllFactorLevels", factor = "showAllFactorLevels", labelled = "showAllFactorLevels",   haven_labelled = "showAllFactorLevels", numeric = NULL,   integer = NULL, logical = NULL, Date = NULL), listChecks = FALSE, maxProbVals = Inf, codebook = TRUE, reportTitle = "Handpicked_Social_week04-narem`

Handpicked_Social_week04-naremove_test

Autogenerated data summary from dataMaid

2023-01-09 13:16:20

Data report overview

The dataset examined has the following dimensions:

Feature	Result
Number of observations	108
Number of variables	60

Codebook summary table

Label	Variable	Class	# unique values	Missing	Description
	**sex**	factor	2	0.00 %	Sex of patient, 0 = Female, 1=Male
	**age**	numeric	35	0.00 %	
	**messages_len_7**	numeric	37	0.00 %	-Meta information of messages-Length of messages-up until day-7
	**messages_len_tp_7**	numeric	93	0.00 %	-Meta information of messages-Length of messages-therapist messages-up until day-7
	**messages_7**	numeric	5	0.00 %	-Meta information of messages-up until day-7
	**messages_tp_7**	numeric	4	0.00 %	-Meta information of messages-therapist messages-up until day-7
	**homeworks_7**	numeric	4	0.00 %	-Number of homework messages sent in-up until day-7
	**messages_len_14**	numeric	57	0.00 %	-Meta information of messages-Length of messages-up until day-14
	**messages_len_tp_14**	numeric	95	0.00 %	-Meta information of messages-Length of messages-therapist messages-up until day-14
	**messages_14**	numeric	5	0.00 %	-Meta information of messages-up until day-14
	**messages_tp_14**	numeric	4	0.00 %	-Meta information of messages-therapist messages-up until day-14

Label	Variable	Class	# unique values	Missing	Description
	**homeworks_14**	numeric	4	0.00 %	-Number of homework messages sent in-up until day-14
	**messages_len_21**	numeric	50	0.00 %	-Meta information of messages-Length of messages-up until day-21
	**messages_len_tp_21**	numeric	92	0.00 %	-Meta information of messages-Length of messages-therapist messages-up until day-21
	**messages_21**	numeric	5	0.00 %	-Meta information of messages-up until day-21
	**messages_tp_21**	numeric	5	0.00 %	-Meta information of messages-therapist messages-up until day-21
	**homeworks_21**	numeric	4	0.00 %	-Number of homework messages sent in-up until day-21
	**messages_len_28**	numeric	48	0.00 %	-Meta information of messages-Length of messages-up until day-28
	**messages_len_tp_28**	numeric	97	0.00 %	-Meta information of messages-Length of messages-therapist messages-up until day-28
	**messages_28**	numeric	5	0.00 %	-Meta information of messages-up until day-28
	**messages_tp_28**	numeric	5	0.00 %	-Meta information of messages-therapist messages-up until day-28
	**homeworks_28**	numeric	5	0.00 %	-Number of homework messages sent in-up until day-28
	**PDSS-SR-3064_SCREEN_sum**	numeric	22	0.00 %	Anxiety questionnaire, self rated-Timepoint before treatment starts-Sum of the entire measure
	**MADRS-1951_SCREEN_sum**	numeric	32	0.00 %	Depression questionnaire, self rated-Timepoint before treatment starts-Sum of the entire measure
	**LSAS-2241_SCREEN_sum**	numeric	64	0.00 %	Social anxiety questionnaire, self rated-Timepoint before treatment starts-Sum of the entire measure
	**MADRS-1951_SCREEN_DateCompleted_day**	numeric	7	0.00 %	Depression questionnaire, self rated-Timepoint before treatment starts-Cyclic transformation of what day 0-6 during week it was filled in

Label	Variable	Class	# unique values	Missing	Description
	**MADRS-1951_SCREEN_DateCompleted_time**	numeric	98	0.00 %	Depression questionnaire, self rated-Timepoint before treatment starts-Cyclic transformation of what time during day 0-1440 it was filled in
	**PDSS-SR-3064_SCREEN_DateCompleted_day**	numeric	7	0.00 %	Anxiety questionnaire, self rated-Timepoint before treatment starts-Cyclic transformation of what day 0-6 during week it was filled in
	**PDSS-SR-3064_SCREEN_DateCompleted_time**	numeric	102	0.00 %	Anxiety questionnaire, self rated-Timepoint before treatment starts-Cyclic transformation of what time during day 0-1440 it was filled in
	**LSAS-2241_SCREEN_DateCompleted_day**	numeric	7	0.00 %	Social anxiety questionnaire, self rated-Timepoint before treatment starts-Cyclic transformation of what day 0-6 during week it was filled in
	**LSAS-2241_SCREEN_DateCompleted_time**	numeric	101	0.00 %	Social anxiety questionnaire, self rated-Timepoint before treatment starts-Cyclic transformation of what time during day 0-1440 it was filled in
	**outcome**	numeric	68	0.00 %	
	**ncomorbid**	numeric	5	0.00 %	
	**currentwork_proff**	factor	2	0.00 %	Currently in work for trained proffession
	**Marital_1833_gift**	factor	2	0.00 %	Marital status: Married or not
	**Marital_1833_separerad**	factor	2	0.00 %	Marital status: divocered/equivalent
	**Marital_1833_singel**	factor	2	0.00 %	Marital status: single
	**Edu_1843_2**	factor	2	0.00 %	7-9 years education
	**Edu_1843_3**	factor	2	0.00 %	Uncompleted upper secondary school
	**Edu_1843_4**	factor	2	0.00 %	Higher vocational education
	**Edu_1843_5**	factor	2	0.00 %	Completed upper secondary school
	**Edu_1843_6**	factor	2	0.00 %	Uncompleted university degree
	**Edu_1843_7**	factor	2	0.00 %	University degree
	**cscale**	numeric	30	0.00 %	
	**mainsymptom_PRE_sum**	numeric	61	0.00 %	PDSS-SR for panic, MADRS for depression, LSAS for social anxiety-Timepoint just before beginning treatment-Sum of the entire measure

Label	Variable	Class	# unique values	Missing	Description
	**mainsymptom_PRE_duration**	numeric	97	0.00 %	PDSS-SR for panic, MADRS for depression, LSAS for social anxiety-Timepoint just before beginning treatment-Time to fill in measure/questionnaire
	**mainsymptom_PRE_DateCompleted_day**	numeric	7	0.00 %	PDSS-SR for panic, MADRS for depression, LSAS for social anxiety-Timepoint just before beginning treatment-Cyclic transformation of what day 0-6 during week it was filled in
	**mainsymptom_PRE_DateCompleted_time**	numeric	101	0.00 %	PDSS-SR for panic, MADRS for depression, LSAS for social anxiety-Timepoint just before beginning treatment-Cyclic transformation of what time during day 0-1440 it was filled in
	**mainsymptom_WEEK01_sum**	numeric	68	0.00 %	PDSS-SR for panic, MADRS for depression, LSAS for social anxiety-Timepoint after one week in treatment-Sum of the entire measure
	**mainsymptom_WEEK01_duration**	numeric	99	0.00 %	PDSS-SR for panic, MADRS for depression, LSAS for social anxiety-Timepoint after one week in treatment-Time to fill in measure/questionnaire
	**mainsymptom_WEEK01_DateCompleted_day**	numeric	7	0.00 %	PDSS-SR for panic, MADRS for depression, LSAS for social anxiety-Timepoint after one week in treatment-Cyclic transformation of what day 0-6 during week it was filled in
	**mainsymptom_WEEK01_DateCompleted_time**	numeric	99	0.00 %	PDSS-SR for panic, MADRS for depression, LSAS for social anxiety-Timepoint after one week in treatment-Cyclic transformation of what time during day 0-1440 it was filled in
	**mainsymptom_WEEK02_sum**	numeric	65	0.00 %	PDSS-SR for panic, MADRS for depression, LSAS for social anxiety-Timepoint after two weeks in treatment-Sum of the entire measure
	**mainsymptom_WEEK02_duration**	numeric	88	0.00 %	PDSS-SR for panic, MADRS for depression, LSAS for social anxiety-Timepoint after two weeks in treatment-Time to fill in measure/questionnaire

Label	Variable	Class	# unique values	Missing	Description
	**mainsymptom_WEEK02_DateCompleted_day**	numeric	7	0.00 %	PDSS-SR for panic, MADRS for depression, LSAS for social anxiety-Timepoint after two weeks in treatment-Cyclic transformation of what day 0-6 during week it was filled in
	**mainsymptom_WEEK02_DateCompleted_time**	numeric	103	0.00 %	PDSS-SR for panic, MADRS for depression, LSAS for social anxiety-Timepoint after two weeks in treatment-Cyclic transformation of what time during day 0-1440 it was filled in
	**mainsymptom_WEEK03_sum**	numeric	63	0.00 %	PDSS-SR for panic, MADRS for depression, LSAS for social anxiety-Timepoint after three weeks in treatment-Sum of the entire measure
	**mainsymptom_WEEK03_duration**	numeric	96	0.00 %	PDSS-SR for panic, MADRS for depression, LSAS for social anxiety-Timepoint after three weeks in treatment-Time to fill in measure/questionnaire
	**mainsymptom_WEEK03_DateCompleted_day**	numeric	7	0.00 %	PDSS-SR for panic, MADRS for depression, LSAS for social anxiety-Timepoint after three weeks in treatment-Cyclic transformation of what day 0-6 during week it was filled in
	**mainsymptom_WEEK03_DateCompleted_time**	numeric	103	0.00 %	PDSS-SR for panic, MADRS for depression, LSAS for social anxiety-Timepoint after three weeks in treatment-Cyclic transformation of what time during day 0-1440 it was filled in

Variable list

sex

Feature	Result
Variable type	factor
Number of missing obs.	0 (0 %)
Number of unique values	2
Mode	“0”
Reference category	0

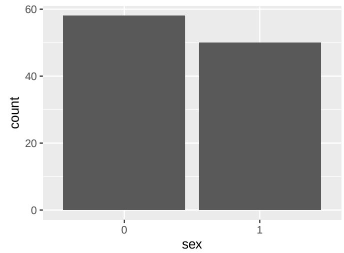

- Observed factor levels: "0", "1".

---

age

Feature	Result
Variable type	numeric
Number of missing obs.	0 (0 %)
Number of unique values	35
Median	-0.42
1st and 3rd quartiles	-0.9; 0.24
Min. and max.	-1.42; 2.51

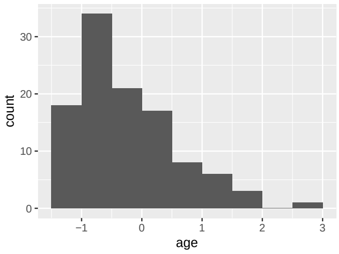

messages_len_7

Feature	Result
Variable type	numeric
Number of missing obs.	0 (0 %)
Number of unique values	37
Median	-0.32
1st and 3rd quartiles	-0.32; -0.01
Min. and max.	-0.32; 5.33

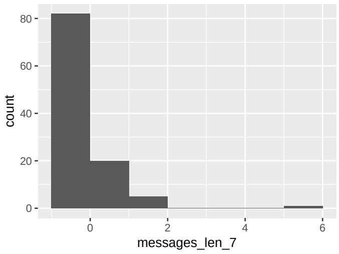

messages_len_tp_7

Feature	Result
Variable type	numeric
Number of missing obs.	0 (0 %)
Number of unique values	93
Median	0.4
1st and 3rd quartiles	-0.35; 0.92
Min. and max.	-1.1; 2.77

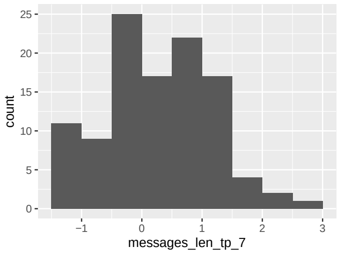

messages_7

Feature	Result
Variable type	numeric
Number of missing obs.	0 (0 %)
Number of unique values	5
Median	-0.55
1st and 3rd quartiles	-0.55; 0.58
Min. and max.	-0.55; 5.13

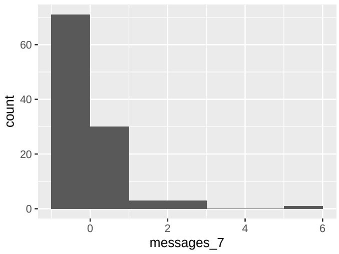

messages_tp_7

Feature	Result
Variable type	numeric
Number of missing obs.	0 (0 %)
Number of unique values	4
Median	0.15
1st and 3rd quartiles	0.15; 1.21
Min. and max.	-0.91; 2.27

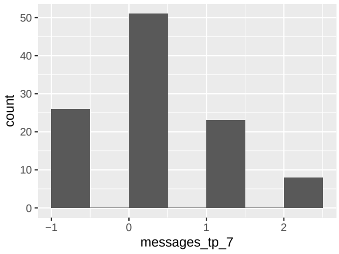

homeworks_7

Feature	Result
Variable type	numeric
Number of missing obs.	0 (0 %)
Number of unique values	4
Median	0.2
1st and 3rd quartiles	0.2; 0.2
Min. and max.	-1.03; 2.68

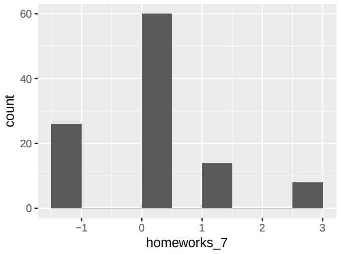

messages_len_14

Feature	Result
Variable type	numeric
Number of missing obs.	0 (0 %)
Number of unique values	57
Median	-0.25
1st and 3rd quartiles	-0.41; 0.37
Min. and max.	-0.41; 7.91

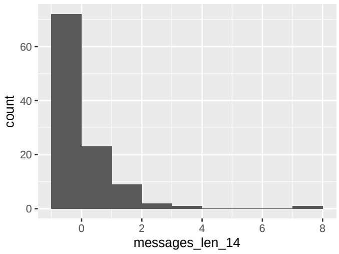

messages_len_tp_14

Feature	Result
Variable type	numeric
Number of missing obs.	0 (0 %)
Number of unique values	95
Median	0.06
1st and 3rd quartiles	-0.41; 0.78
Min. and max.	-1.11; 4.23

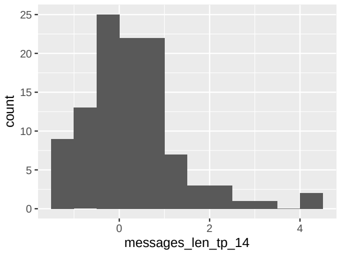

messages_14

Feature	Result
Variable type	numeric
Number of missing obs.	0 (0 %)
Number of unique values	5
Median	0.36
1st and 3rd quartiles	-0.7; 0.36
Min. and max.	-0.7; 3.54

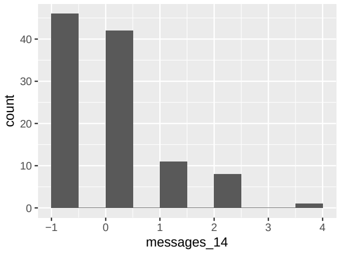

messages_tp_14

Feature	Result
Variable type	numeric
Number of missing obs.	0 (0 %)
Number of unique values	4
Median	-0.35
1st and 3rd quartiles	-0.35; 0.87
Min. and max.	-1.57; 2.09

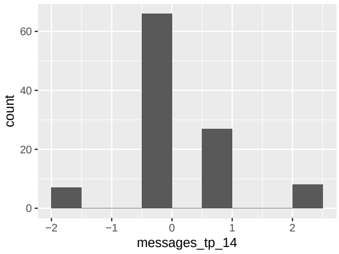

homeworks_14

Feature	Result
Variable type	numeric
Number of missing obs.	0 (0 %)
Number of unique values	4
Median	0.33
1st and 3rd quartiles	-0.98; 0.33
Min. and max.	-0.98; 2.96

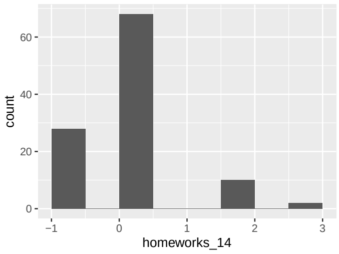

messages_len_21

Feature	Result
Variable type	numeric
Number of missing obs.	0 (0 %)
Number of unique values	50
Median	-0.35
1st and 3rd quartiles	-0.46; 0
Min. and max.	-0.46; 5.36

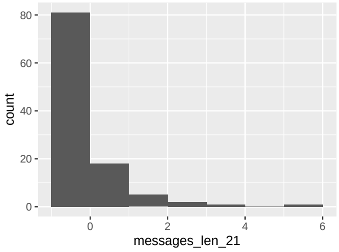

messages_len_tp_21

Feature	Result
Variable type	numeric
Number of missing obs.	0 (0 %)
Number of unique values	92
Median	-0.05
1st and 3rd quartiles	-0.65; 0.53
Min. and max.	-1.01; 7.08

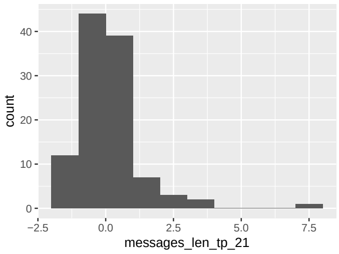

messages_21

Feature	Result
Variable type	numeric
Number of missing obs.	0 (0 %)
Number of unique values	5
Median	0.34
1st and 3rd quartiles	-0.74; 0.34
Min. and max.	-0.74; 4.66

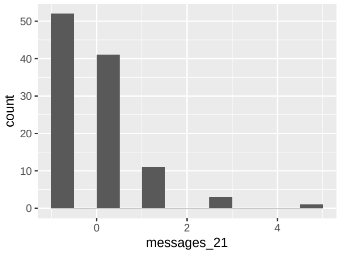

messages_tp_21

Feature	Result
Variable type	numeric
Number of missing obs.	0 (0 %)
Number of unique values	5
Median	-0.32
1st and 3rd quartiles	-0.32; 0.84
Min. and max.	-1.49; 4.33

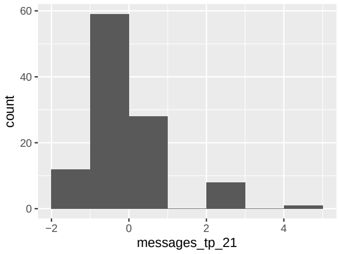

homeworks_21

Feature	Result
Variable type	numeric
Number of missing obs.	0 (0 %)
Number of unique values	4
Median	0.36
1st and 3rd quartiles	-1.01; 0.36
Min. and max.	-1.01; 3.11

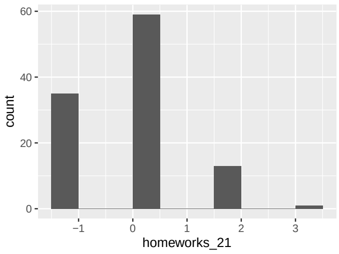

messages_len_28

Feature	Result
Variable type	numeric
Number of missing obs.	0 (0 %)
Number of unique values	48
Median	-0.39
1st and 3rd quartiles	-0.41; 0.02
Min. and max.	-0.41; 3.39

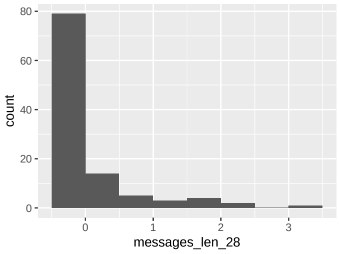

messages_len_tp_28

Feature	Result
Variable type	numeric
Number of missing obs.	0 (0 %)
Number of unique values	97
Median	0.08
1st and 3rd quartiles	-0.34; 0.7
Min. and max.	-0.94; 3.58

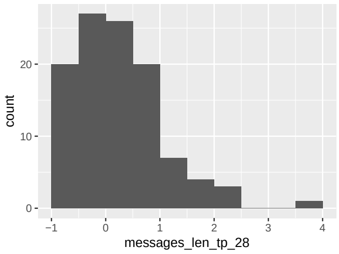

messages_28

Feature	Result
Variable type	numeric
Number of missing obs.	0 (0 %)
Number of unique values	5
Median	-0.15
1st and 3rd quartiles	-0.72; 0.41
Min. and max.	-0.72; 3.8

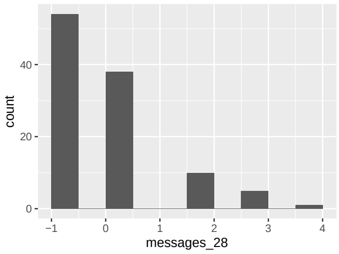

messages_tp_28

Feature	Result
Variable type	numeric
Number of missing obs.	0 (0 %)
Number of unique values	5
Median	-0.24
1st and 3rd quartiles	-0.24; 0.93
Min. and max.	-1.42; 3.28

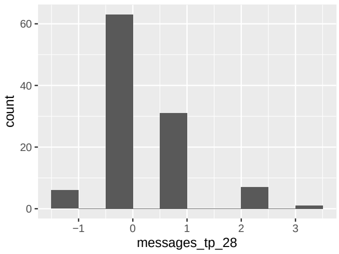

homeworks_28

Feature	Result
Variable type	numeric
Number of missing obs.	0 (0 %)
Number of unique values	5
Median	0.46
1st and 3rd quartiles	-0.91; 0.46
Min. and max.	-0.91; 7.31

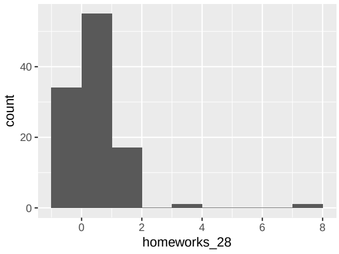

PDSS-SR-3064_SCREEN_sum

Feature	Result
Variable type	numeric
Number of missing obs.	0 (0 %)
Number of unique values	22
Median	-0.35
1st and 3rd quartiles	-0.86; 0.28
Min. and max.	-1.29; 2.49

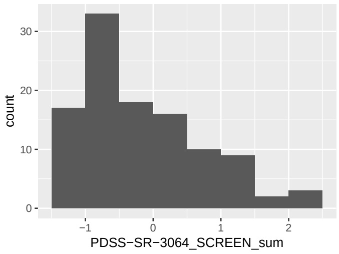

MADRS-1951_SCREEN_sum

Feature	Result
Variable type	numeric
Number of missing obs.	0 (0 %)
Number of unique values	32
Median	-0.45
1st and 3rd quartiles	-1.19; 0.33
Min. and max.	-2.56; 2.03

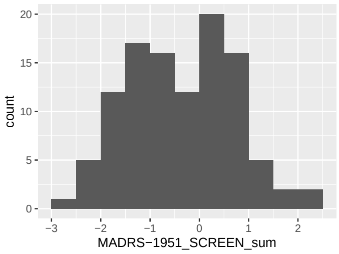

LSAS-2241_SCREEN_sum

Feature	Result
Variable type	numeric
Number of missing obs.	0 (0 %)
Number of unique values	64
Median	0.62
1st and 3rd quartiles	0.14; 1.34
Min. and max.	-1.1; 2.64

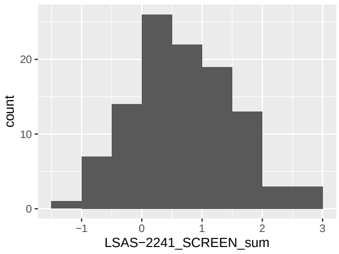

MADRS-1951_SCREEN_DateCompleted_day

Feature	Result
Variable type	numeric
Number of missing obs.	0 (0 %)
Number of unique values	7
Median	1
1st and 3rd quartiles	-1; 1
Min. and max.	-1.37; 1.37

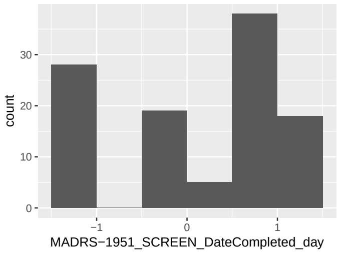

MADRS-1951_SCREEN_DateCompleted_time

Feature	Result
Variable type	numeric
Number of missing obs.	0 (0 %)
Number of unique values	98
Median	-0.57
1st and 3rd quartiles	-1.25; 0.35
Min. and max.	-1.41; 1.41

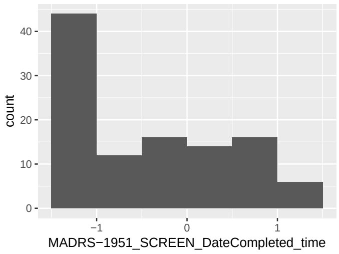

PDSS-SR-3064_SCREEN_DateCompleted_day

Feature	Result
Variable type	numeric
Number of missing obs.	0 (0 %)
Number of unique values	7
Median	1
1st and 3rd quartiles	-1; 1
Min. and max.	-1.37; 1.37

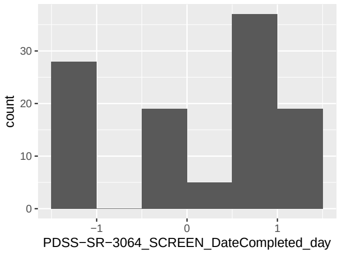

PDSS-SR-3064_SCREEN_DateCompleted_time

Feature	Result
Variable type	numeric
Number of missing obs.	0 (0 %)
Number of unique values	102
Median	-0.54
1st and 3rd quartiles	-1.27; 0.32
Min. and max.	-1.41; 1.41

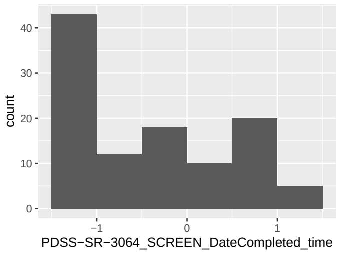

LSAS-2241_SCREEN_DateCompleted_day

Feature	Result
Variable type	numeric
Number of missing obs.	0 (0 %)
Number of unique values	7
Median	1
1st and 3rd quartiles	-0.52; 1
Min. and max.	-1.37; 1.37

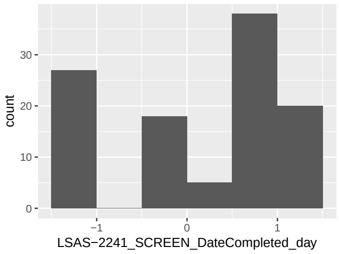

LSAS-2241_SCREEN_DateCompleted_time

Feature	Result
Variable type	numeric
Number of missing obs.	0 (0 %)
Number of unique values	101
Median	-0.54
1st and 3rd quartiles	-1.26; 0.37
Min. and max.	-1.41; 1.41

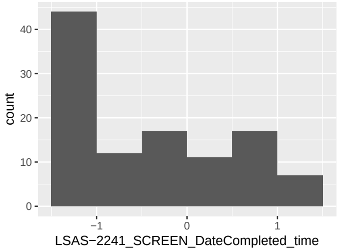

outcome

Feature	Result
Variable type	numeric
Number of missing obs.	0 (0 %)
Number of unique values	68
Median	0.09
1st and 3rd quartiles	-0.62; 0.6
Min. and max.	-2.01; 2.79

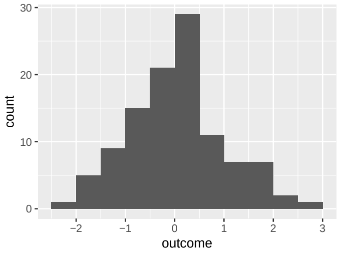

ncomorbid

Feature	Result
Variable type	numeric
Number of missing obs.	0 (0 %)
Number of unique values	5
Median	0
1st and 3rd quartiles	0; 1
Min. and max.	0; 4

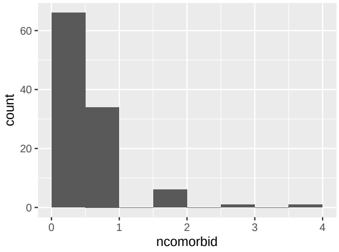

currentwork_proff

Feature	Result
Variable type	factor
Number of missing obs.	0 (0 %)
Number of unique values	2
Mode	“1”
Reference category	0

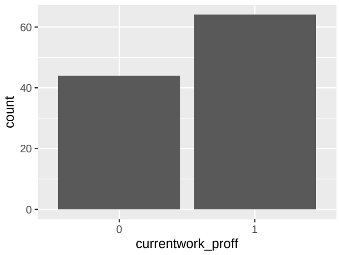

- Observed factor levels: "0", "1".

Marital_1833_gift

Feature	Result
Variable type	factor
Number of missing obs.	0 (0 %)
Number of unique values	2
Mode	“1”
Reference category	0

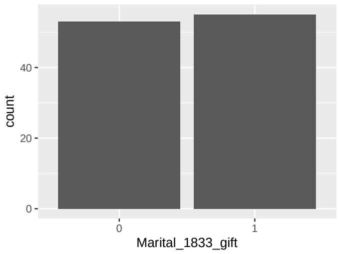

- Observed factor levels: "0", "1".

Marital_1833_separerad

Feature	Result
Variable type	factor
Number of missing obs.	0 (0 %)
Number of unique values	2
Mode	“0”
Reference category	0

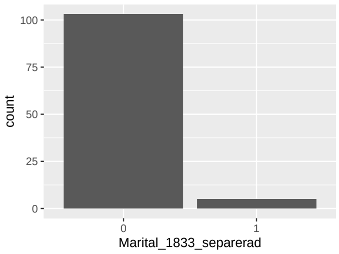

- Observed factor levels: "0", "1".

Marital_1833_singel

Feature	Result
Variable type	factor
Number of missing obs.	0 (0 %)
Number of unique values	2
Mode	“0”
Reference category	0

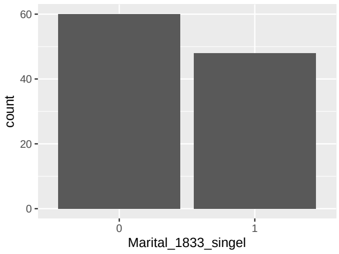

- Observed factor levels: "0", "1".

Edu_1843_2

Feature	Result
Variable type	factor
Number of missing obs.	0 (0 %)
Number of unique values	2
Mode	"0"
Reference category	0

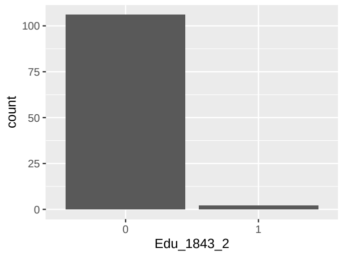

- Observed factor levels: "0", "1".

Edu_1843_3

Feature	Result
Variable type	factor
Number of missing obs.	0 (0 %)
Number of unique values	2
Mode	"0"
Reference category	0

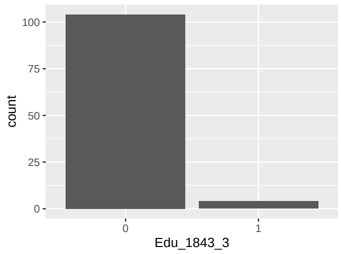

- Observed factor levels: "0", "1".

Edu_1843_4

Feature	Result
Variable type	factor
Number of missing obs.	0 (0 %)
Number of unique values	2
Mode	"0"
Reference category	0

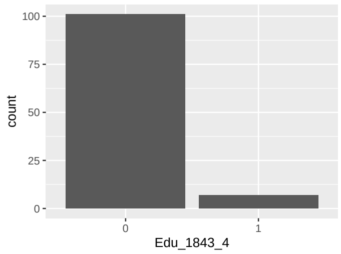

- Observed factor levels: "0", "1".

Edu_1843_5

Feature	Result
Variable type	factor
Number of missing obs.	0 (0 %)
Number of unique values	2
Mode	"0"
Reference category	0

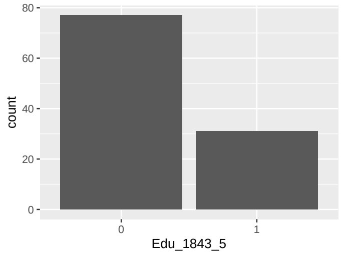

- Observed factor levels: "0", "1".

Edu_1843_6

Feature	Result
Variable type	factor
Number of missing obs.	0 (0 %)
Number of unique values	2
Mode	"0"
Reference category	0

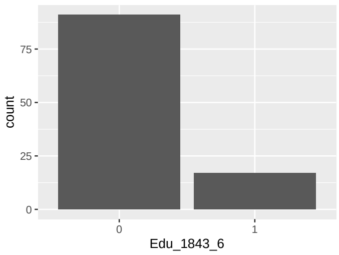

- Observed factor levels: "0", "1".

Edu_1843_7

Feature	Result
Variable type	factor
Number of missing obs.	0 (0 %)
Number of unique values	2
Mode	"0"
Reference category	0

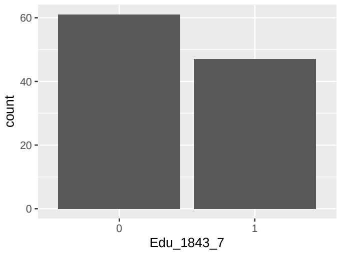

- Observed factor levels: "0", "1".

---

cscale

Feature	Result
Variable type	numeric
Number of missing obs.	0 (0 %)
Number of unique values	30
Median	0.19
1st and 3rd quartiles	-0.26; 0.84
Min. and max.	-4.14; 1.67

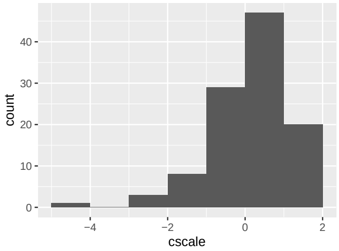

---

mainsymptom_PRE_sum

Feature	Result
Variable type	numeric
Number of missing obs.	0 (0 %)
Number of unique values	61
Median	0.04
1st and 3rd quartiles	-0.68; 0.81
Min. and max.	-2.45; 2.41

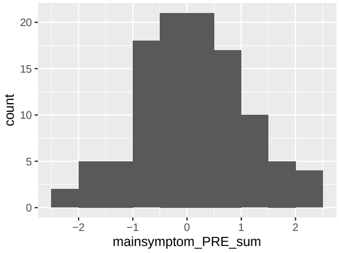

---

mainsymptom_PRE_duration

Feature	Result
Variable type	numeric
Number of missing obs.	0 (0 %)
Number of unique values	97
Median	-0.12
1st and 3rd quartiles	-0.22; -0.01
Min. and max.	-0.38; 4.65

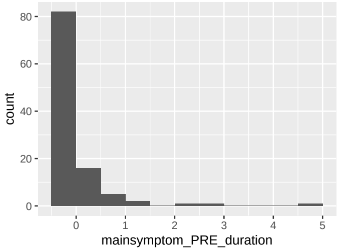

mainsymptom_PRE_DateCompleted_day

Feature	Result
Variable type	numeric
Number of missing obs.	0 (0 %)
Number of unique values	7
Median	1
1st and 3rd quartiles	-0.37; 1.37
Min. and max.	-1.37; 1.37

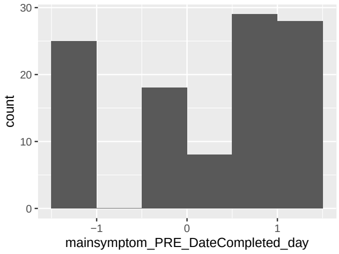

mainsymptom_PRE_DateCompleted_time

Feature	Result
Variable type	numeric
Number of missing obs.	0 (0 %)
Number of unique values	101
Median	-0.74
1st and 3rd quartiles	-1.31; 0.03
Min. and max.	-1.41; 1.33

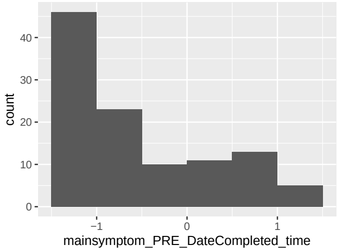

mainsymptom_WEEK01_sum

Feature	Result
Variable type	numeric
Number of missing obs.	0 (0 %)
Number of unique values	68
Median	0.04
1st and 3rd quartiles	-0.51; 0.82
Min. and max.	-2.31; 2.46

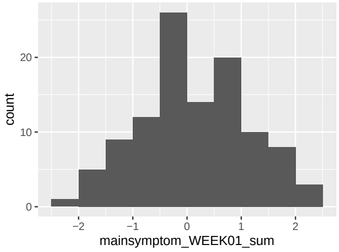

mainsymptom_WEEK01_duration

Feature	Result
Variable type	numeric
Number of missing obs.	0 (0 %)
Number of unique values	99
Median	-0.14
1st and 3rd quartiles	-0.27; 0.07
Min. and max.	-0.46; 3.08

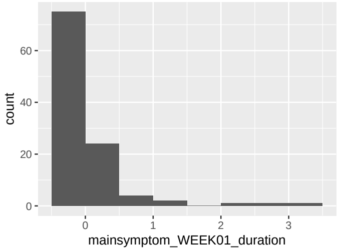

mainsymptom_WEEK01_DateCompleted_day

Feature	Result
Variable type	numeric
Number of missing obs.	0 (0 %)
Number of unique values	7
Median	0
1st and 3rd quartiles	-1; 1
Min. and max.	-1.37; 1.37

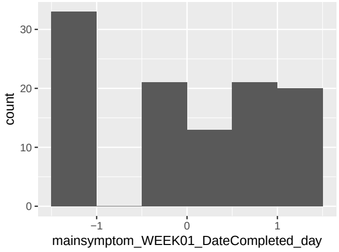

mainsymptom_WEEK01_DateCompleted_time

Feature	Result
Variable type	numeric
Number of missing obs.	0 (0 %)
Number of unique values	99
Median	-0.54
1st and 3rd quartiles	-1.15; 0.32
Min. and max.	-1.41; 1.37

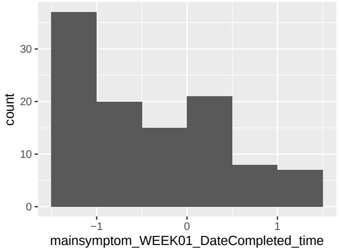

mainsymptom_WEEK02_sum

Feature	Result
Variable type	numeric
Number of missing obs.	0 (0 %)
Number of unique values	65
Median	0.09
1st and 3rd quartiles	-0.58; 0.83
Min. and max.	-2.26; 2.44

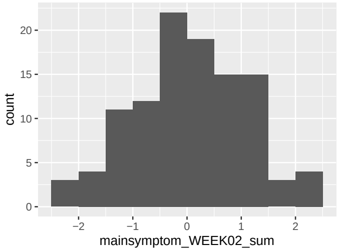

mainsymptom_WEEK02_duration

Feature	Result
Variable type	numeric
Number of missing obs.	0 (0 %)
Number of unique values	88
Median	-0.21
1st and 3rd quartiles	-0.26; -0.1
Min. and max.	-0.4; 12.5

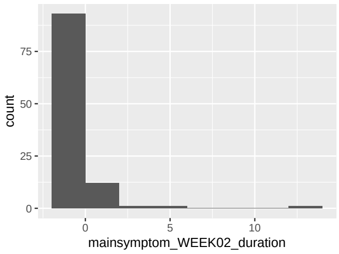

mainsymptom_WEEK02_DateCompleted_day

Feature	Result
Variable type	numeric
Number of missing obs.	0 (0 %)
Number of unique values	7
Median	1
1st and 3rd quartiles	-0.37; 1.37
Min. and max.	-1.37; 1.37

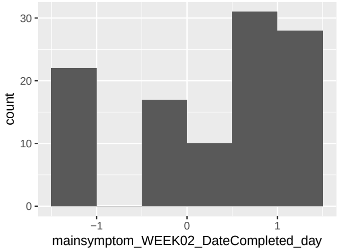

mainsymptom_WEEK02_DateCompleted_time

Feature	Result
Variable type	numeric
Number of missing obs.	0 (0 %)
Number of unique values	103
Median	-0.61
1st and 3rd quartiles	-1.05; 0.21
Min. and max.	-1.41; 1.4

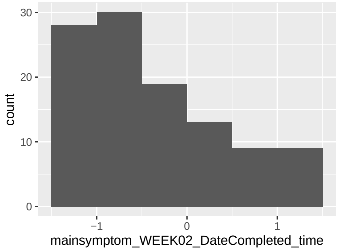

mainsymptom_WEEK03_sum

Feature	Result
Variable type	numeric
Number of missing obs.	0 (0 %)
Number of unique values	63
Median	0.03
1st and 3rd quartiles	-0.55; 0.79
Min. and max.	-2.12; 2.26

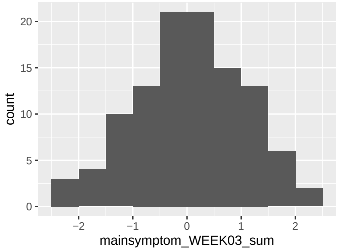

mainsymptom_WEEK03_duration

Feature	Result
Variable type	numeric
Number of missing obs.	0 (0 %)
Number of unique values	96
Median	-0.13
1st and 3rd quartiles	-0.17; -0.07
Min. and max.	-0.23; 6.32

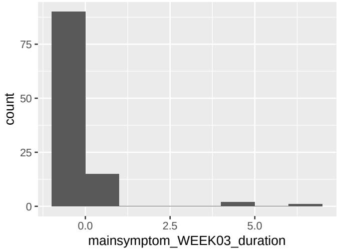

mainsymptom_WEEK03_DateCompleted_day

Feature	Result
Variable type	numeric
Number of missing obs.	0 (0 %)
Number of unique values	7
Median	1
1st and 3rd quartiles	-0.37; 1
Min. and max.	-1.37; 1.37

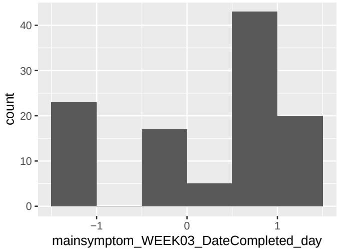

mainsymptom_WEEK03_DateCompleted_time

Feature	Result
Variable type	numeric
Number of missing obs.	0 (0 %)
Number of unique values	103
Median	-0.79
1st and 3rd quartiles	-1.22; -0.14
Min. and max.	-1.41; 1.12

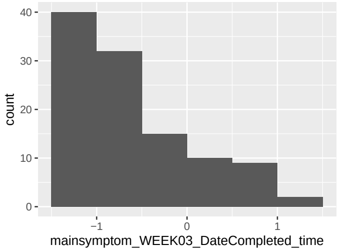

Report generation information:

- Created by: Could not determine from system (username: `nilisa`).
- Report creation time: Mon Jan 09 2023 13:16:20
- Report was run from directory: `/home/nilisa/projects/phd_study1/r`
- dataMaid v1.4.1 [Pkg: 2021-10-08 from CRAN (R 4.2.2)]
- R version 4.2.2 Patched (2022-11-10 r83330).
- Platform: x86_64-pc-linux-gnu (64-bit)(Ubuntu 20.04.5 LTS).
- Function call: `dataMaid::makeDataReport(data = gd, mode = c("summarize", "visualize", "check"), smartNum = FALSE, file = "~/projects/data/study1multiverse/results/graphs_n_figures/codebooks/codebook replace = TRUE, openResult = FALSE, checks = list(character = "showAllFactorLevels", factor = "showAllFactorLevels", labelled = "showAllFactorLevels",   haven_labelled = "showAllFactorLevels", numeric = NULL,   integer = NULL, logical = NULL, Date = NULL), listChecks = FALSE, maxProbVals = Inf, codebook = TRUE, reportTitle = "Handpicked_Social_week04-naren`

Handpicked_Social_week04-naremove_train

Autogenerated data summary from dataMaid

2023-01-09 13:09:54

Data report overview

The dataset examined has the following dimensions:

Feature	Result
Number of observations	879
Number of variables	60

Codebook summary table

Label	Variable	Class	# unique values	Missing	Description
	**sex**	factor	2	0.00 %	Sex of patient, 0 = Female, 1=Male
	**age**	numeric	56	0.00 %	
	**messages_len_7**	numeric	223	0.00 %	-Meta information of messages-Length of messages-up until day-7
	**messages_len_tp_7**	numeric	501	0.00 %	-Meta information of messages-Length of messages-therapist messages-up until day-7
	**messages_7**	numeric	7	0.00 %	-Meta information of messages-up until day-7
	**messages_tp_7**	numeric	6	0.00 %	-Meta information of messages-therapist messages-up until day-7
	**homeworks_7**	numeric	7	0.00 %	-Number of homework messages sent in-up until day-7
	**messages_len_14**	numeric	275	0.00 %	-Meta information of messages-Length of messages-up until day-14
	**messages_len_tp_14**	numeric	552	0.00 %	-Meta information of messages-Length of messages-therapist messages-up until day-14
	**messages_14**	numeric	9	0.00 %	-Meta information of messages-up until day-14
	**messages_tp_14**	numeric	7	0.00 %	-Meta information of messages-therapist messages-up until day-14

Label	Variable	Class	# unique values	Missing	Description
	**homeworks_14**	numeric	5	0.00 %	-Number of homework messages sent in-up until day-14
	**messages_len_21**	numeric	262	0.00 %	-Meta information of messages-Length of messages-up until day-21
	**messages_len_tp_21**	numeric	540	0.00 %	-Meta information of messages-Length of messages-therapist messages-up until day-21
	**messages_21**	numeric	7	0.00 %	-Meta information of messages-up until day-21
	**messages_tp_21**	numeric	5	0.00 %	-Meta information of messages-therapist messages-up until day-21
	**homeworks_21**	numeric	5	0.00 %	-Number of homework messages sent in-up until day-21
	**messages_len_28**	numeric	254	0.00 %	-Meta information of messages-Length of messages-up until day-28
	**messages_len_tp_28**	numeric	530	0.00 %	-Meta information of messages-Length of messages-therapist messages-up until day-28
	**messages_28**	numeric	8	0.00 %	-Meta information of messages-up until day-28
	**messages_tp_28**	numeric	5	0.00 %	-Meta information of messages-therapist messages-up until day-28
	**homeworks_28**	numeric	6	0.00 %	-Number of homework messages sent in-up until day-28
	**PDSS-SR-3064_SCREEN_sum**	numeric	24	0.00 %	Anxiety questionnaire, self rated-Timepoint before treatment starts-Sum of the entire measure
	**MADRS-1951_SCREEN_sum**	numeric	39	0.00 %	Depression questionnaire, self rated-Timepoint before treatment starts-Sum of the entire measure
	**LSAS-2241_SCREEN_sum**	numeric	113	0.00 %	Social anxiety questionnaire, self rated-Timepoint before treatment starts-Sum of the entire measure
	**MADRS-1951_SCREEN_DateCompleted_day**	numeric	7	0.00 %	Depression questionnaire, self rated-Timepoint before treatment starts-Cyclic transformation of what day 0-6 during week it was filled in

Label	Variable	Class	# unique values	Missing	Description
	**MADRS-1951_SCREEN_DateCompleted_time**	numeric	561	0.00 %	Depression questionnaire, self rated-Timepoint before treatment starts-Cyclic transformation of what time during day 0-1440 it was filled in
	**PDSS-SR-3064_SCREEN_DateCompleted_day**	numeric	7	0.00 %	Anxiety questionnaire, self rated-Timepoint before treatment starts-Cyclic transformation of what day 0-6 during week it was filled in
	**PDSS-SR-3064_SCREEN_DateCompleted_time**	numeric	542	0.00 %	Anxiety questionnaire, self rated-Timepoint before treatment starts-Cyclic transformation of what time during day 0-1440 it was filled in
	**LSAS-2241_SCREEN_DateCompleted_day**	numeric	7	0.00 %	Social anxiety questionnaire, self rated-Timepoint before treatment starts-Cyclic transformation of what day 0-6 during week it was filled in
	**LSAS-2241_SCREEN_DateCompleted_time**	numeric	561	0.00 %	Social anxiety questionnaire, self rated-Timepoint before treatment starts-Cyclic transformation of what time during day 0-1440 it was filled in
	**outcome**	numeric	115	0.00 %	
	**ncomorbid**	numeric	5	0.00 %	
	**currentwork_proff**	factor	2	0.00 %	Currently in work for trained proffession
	**Marital_1833_gift**	factor	2	0.00 %	Marital status: Married or not
	**Marital_1833_separerad**	factor	2	0.00 %	Marital status: divocered/equivalent
	**Marital_1833_singel**	factor	2	0.00 %	Marital status: single
	**Edu_1843_2**	factor	2	0.00 %	7-9 years education
	**Edu_1843_3**	factor	2	0.00 %	Uncompleted upper secondary school
	**Edu_1843_4**	factor	2	0.00 %	Higher vocational education
	**Edu_1843_5**	factor	2	0.00 %	Completed upper secondary school
	**Edu_1843_6**	factor	2	0.00 %	Uncompleted university degree
	**Edu_1843_7**	factor	2	0.00 %	University degree
	**cscale**	numeric	43	0.00 %	
	**mainsymptom_PRE_sum**	numeric	112	0.00 %	PDSS-SR for panic, MADRS for depression, LSAS for social anxiety-Timepoint just before beginning treatment-Sum of the entire measure

Label	Variable	Class	# unique values	Missing	Description
	**mainsymptom_PRE_duration**	numeric	522	0.00 %	PDSS-SR for panic, MADRS for depression, LSAS for social anxiety-Timepoint just before beginning treatment-Time to fill in measure/questionnaire
	**mainsymptom_PRE_DateCompleted_day**	numeric	7	0.00 %	PDSS-SR for panic, MADRS for depression, LSAS for social anxiety-Timepoint just before beginning treatment-Cyclic transformation of what day 0-6 during week it was filled in
	**mainsymptom_PRE_DateCompleted_time**	numeric	534	0.00 %	PDSS-SR for panic, MADRS for depression, LSAS for social anxiety-Timepoint just before beginning treatment-Cyclic transformation of what time during day 0-1440 it was filled in
	**mainsymptom_WEEK01_sum**	numeric	116	0.00 %	PDSS-SR for panic, MADRS for depression, LSAS for social anxiety-Timepoint after one week in treatment-Sum of the entire measure
	**mainsymptom_WEEK01_duration**	numeric	402	0.00 %	PDSS-SR for panic, MADRS for depression, LSAS for social anxiety-Timepoint after one week in treatment-Time to fill in measure/questionnaire
	**mainsymptom_WEEK01_DateCompleted_day**	numeric	7	0.00 %	PDSS-SR for panic, MADRS for depression, LSAS for social anxiety-Timepoint after one week in treatment-Cyclic transformation of what day 0-6 during week it was filled in
	**mainsymptom_WEEK01_DateCompleted_time**	numeric	552	0.00 %	PDSS-SR for panic, MADRS for depression, LSAS for social anxiety-Timepoint after one week in treatment-Cyclic transformation of what time during day 0-1440 it was filled in
	**mainsymptom_WEEK02_sum**	numeric	120	0.00 %	PDSS-SR for panic, MADRS for depression, LSAS for social anxiety-Timepoint after two weeks in treatment-Sum of the entire measure
	**mainsymptom_WEEK02_duration**	numeric	407	0.00 %	PDSS-SR for panic, MADRS for depression, LSAS for social anxiety-Timepoint after two weeks in treatment-Time to fill in measure/questionnaire

Label	Variable	Class	# unique values	Missing	Description
	**mainsymptom_WEEK02_DateCompleted_day**	numeric	7	0.00 %	PDSS-SR for panic, MADRS for depression, LSAS for social anxiety-Timepoint after two weeks in treatment-Cyclic transformation of what day 0-6 during week it was filled in
	**mainsymptom_WEEK02_DateCompleted_time**	numeric	569	0.00 %	PDSS-SR for panic, MADRS for depression, LSAS for social anxiety-Timepoint after two weeks in treatment-Cyclic transformation of what time during day 0-1440 it was filled in
	**mainsymptom_WEEK03_sum**	numeric	120	0.00 %	PDSS-SR for panic, MADRS for depression, LSAS for social anxiety-Timepoint after three weeks in treatment-Sum of the entire measure
	**mainsymptom_WEEK03_duration**	numeric	371	0.00 %	PDSS-SR for panic, MADRS for depression, LSAS for social anxiety-Timepoint after three weeks in treatment-Time to fill in measure/questionnaire
	**mainsymptom_WEEK03_DateCompleted_day**	numeric	7	0.00 %	PDSS-SR for panic, MADRS for depression, LSAS for social anxiety-Timepoint after three weeks in treatment-Cyclic transformation of what day 0-6 during week it was filled in
	**mainsymptom_WEEK03_DateCompleted_time**	numeric	548	0.00 %	PDSS-SR for panic, MADRS for depression, LSAS for social anxiety-Timepoint after three weeks in treatment-Cyclic transformation of what time during day 0-1440 it was filled in

Variable list

sex

Feature	Result
Variable type	factor
Number of missing obs.	0 (0 %)
Number of unique values	2
Mode	“0”
Reference category	0

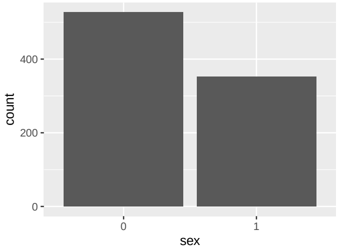

- Observed factor levels: "0", "1".

age

Feature	Result
Variable type	numeric
Number of missing obs.	0 (0 %)
Number of unique values	56
Median	-0.46
1st and 3rd quartiles	-0.9; 0.24
Min. and max.	-1.69; 4.25

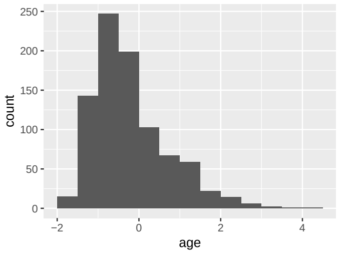

messages_len_7

Feature	Result
Variable type	numeric
Number of missing obs.	0 (0 %)
Number of unique values	223
Median	-0.32
1st and 3rd quartiles	-0.32; 0.01
Min. and max.	-0.32; 11.06

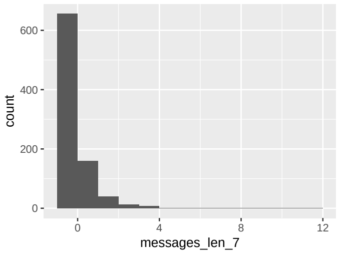

messages_len_tp_7

Feature	Result
Variable type	numeric
Number of missing obs.	0 (0 %)
Number of unique values	501
Median	0.29
1st and 3rd quartiles	-0.29; 1.03
Min. and max.	-1.46; 5.25

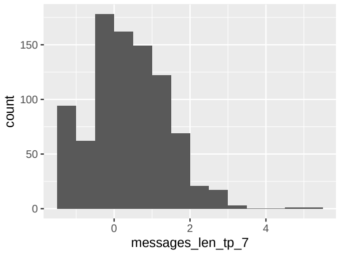

messages_7

Feature	Result
Variable type	numeric
Number of missing obs.	0 (0 %)
Number of unique values	7
Median	-0.55
1st and 3rd quartiles	-0.55; 0.58
Min. and max.	-0.55; 6.27

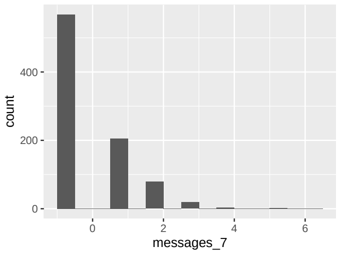

messages_tp_7

Feature	Result
Variable type	numeric
Number of missing obs.	0 (0 %)
Number of unique values	6
Median	0.15
1st and 3rd quartiles	0.15; 1.21
Min. and max.	-1.97; 3.34

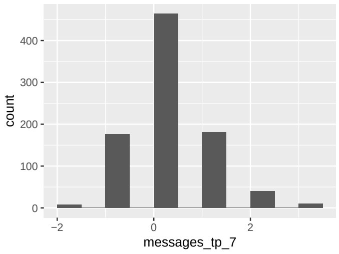

homeworks_7

Feature	Result
Variable type	numeric
Number of missing obs.	0 (0 %)
Number of unique values	7
Median	0.2
1st and 3rd quartiles	0.2; 0.2
Min. and max.	-1.03; 6.39

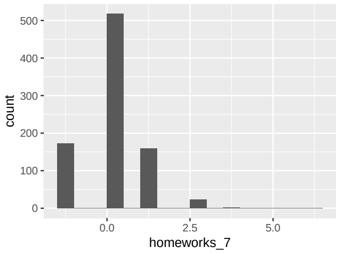

messages_len_14

Feature	Result
Variable type	numeric
Number of missing obs.	0 (0 %)
Number of unique values	275
Median	-0.34
1st and 3rd quartiles	-0.41; 0.09
Min. and max.	-0.41; 12.82

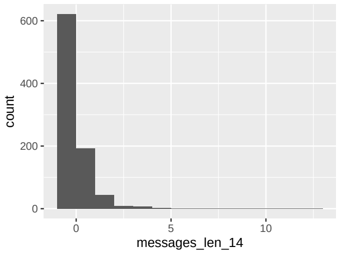

messages_len_tp_14

Feature	Result
Variable type	numeric
Number of missing obs.	0 (0 %)
Number of unique values	552
Median	0.14
1st and 3rd quartiles	-0.54; 0.75
Min. and max.	-1.11; 4.37

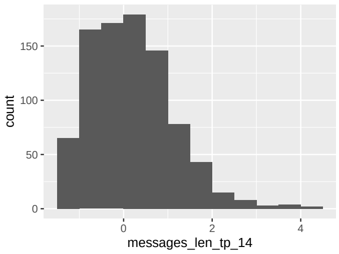

messages_14

Feature	Result
Variable type	numeric
Number of missing obs.	0 (0 %)
Number of unique values	9
Median	0.36
1st and 3rd quartiles	-0.7; 0.36
Min. and max.	-0.7; 9.9

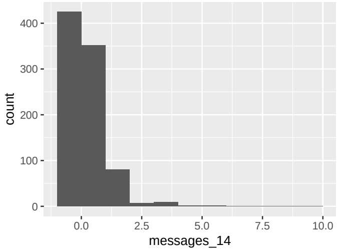

messages_tp_14

Feature	Result
Variable type	numeric
Number of missing obs.	0 (0 %)
Number of unique values	7
Median	-0.35
1st and 3rd quartiles	-0.35; 0.87
Min. and max.	-1.57; 5.75

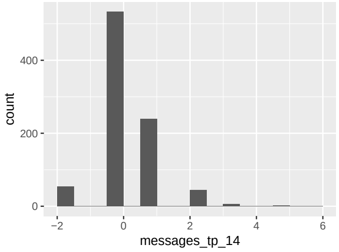

homeworks_14

Feature	Result
Variable type	numeric
Number of missing obs.	0 (0 %)
Number of unique values	5
Median	0.33
1st and 3rd quartiles	-0.98; 0.33
Min. and max.	-0.98; 4.27

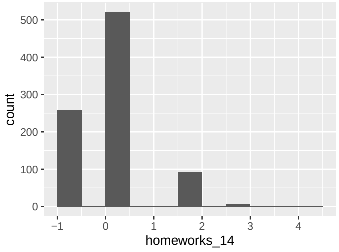

messages_len_21

Feature	Result
Variable type	numeric
Number of missing obs.	0 (0 %)
Number of unique values	262
Median	-0.33
1st and 3rd quartiles	-0.46; 0.12
Min. and max.	-0.46; 13.29

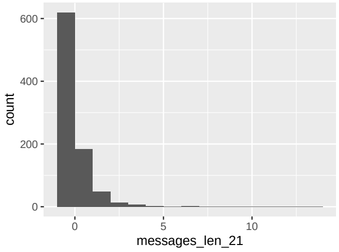

messages_len_tp_21

Feature	Result
Variable type	numeric
Number of missing obs.	0 (0 %)
Number of unique values	540
Median	0.07
1st and 3rd quartiles	-0.53; 0.8
Min. and max.	-1.01; 4.87

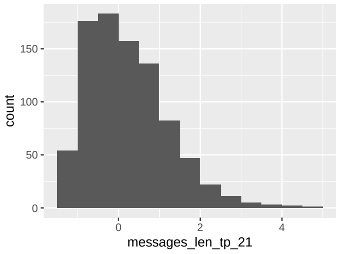

messages_21

Feature	Result
Variable type	numeric
Number of missing obs.	0 (0 %)
Number of unique values	7
Median	0.34
1st and 3rd quartiles	-0.74; 0.34
Min. and max.	-0.74; 6.83

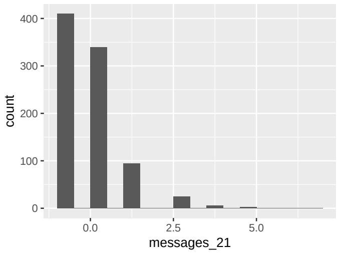

messages_tp_21

Feature	Result
Variable type	numeric
Number of missing obs.	0 (0 %)
Number of unique values	5
Median	-0.32
1st and 3rd quartiles	-0.32; 0.84
Min. and max.	-1.49; 3.17

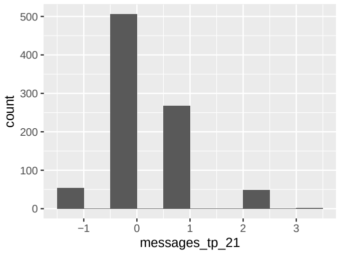

homeworks_21

Feature	Result
Variable type	numeric
Number of missing obs.	0 (0 %)
Number of unique values	5
Median	0.36
1st and 3rd quartiles	-1.01; 0.36
Min. and max.	-1.01; 4.49

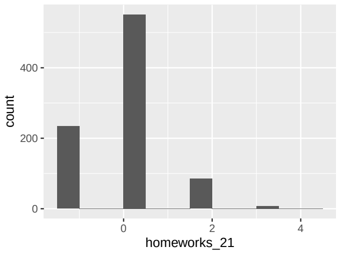

messages_len_28

Feature	Result
Variable type	numeric
Number of missing obs.	0 (0 %)
Number of unique values	254
Median	-0.35
1st and 3rd quartiles	-0.41; 0.12
Min. and max.	-0.41; 10.45

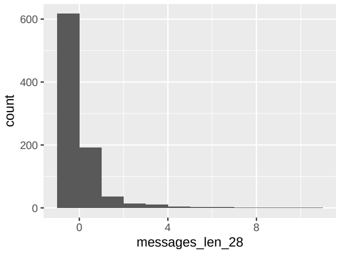

messages_len_tp_28

Feature	Result
Variable type	numeric
Number of missing obs.	0 (0 %)
Number of unique values	530
Median	0.06
1st and 3rd quartiles	-0.49; 0.74
Min. and max.	-0.94; 5.91

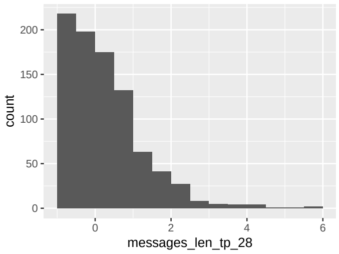

messages_28

Feature	Result
Variable type	numeric
Number of missing obs.	0 (0 %)
Number of unique values	8
Median	0.41
1st and 3rd quartiles	-0.72; 0.41
Min. and max.	-0.72; 7.2

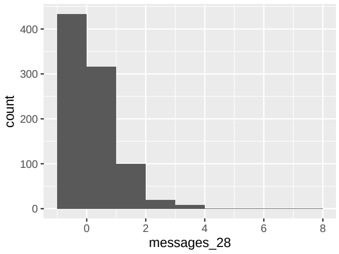

messages_tp_28

Feature	Result
Variable type	numeric
Number of missing obs.	0 (0 %)
Number of unique values	5
Median	-0.24
1st and 3rd quartiles	-0.24; 0.93
Min. and max.	-1.42; 3.28

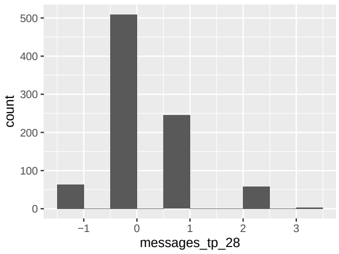

homeworks_28

Feature	Result
Variable type	numeric
Number of missing obs.	0 (0 %)
Number of unique values	6
Median	0.46
1st and 3rd quartiles	-0.91; 0.46
Min. and max.	-0.91; 5.94

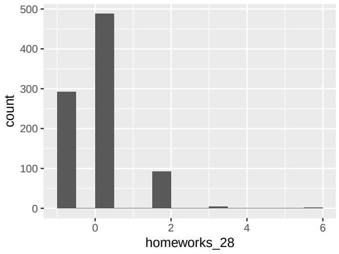

PDSS-SR-3064_SCREEN_sum

Feature	Result
Variable type	numeric
Number of missing obs.	0 (0 %)
Number of unique values	24
Median	-0.19
1st and 3rd quartiles	-0.98; 0.44
Min. and max.	-1.29; 2.8

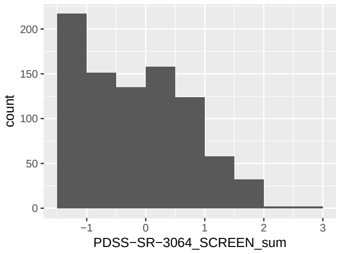

MADRS-1951_SCREEN_sum

Feature	Result
Variable type	numeric
Number of missing obs.	0 (0 %)
Number of unique values	39
Median	-0.57
1st and 3rd quartiles	-1.19; 0.05
Min. and max.	-2.68; 2.28

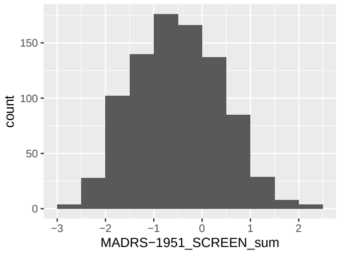

LSAS-2241_SCREEN_sum

Feature	Result
Variable type	numeric
Number of missing obs.	0 (0 %)
Number of unique values	113
Median	0.64
1st and 3rd quartiles	0.08; 1.22
Min. and max.	-1.37; 2.99

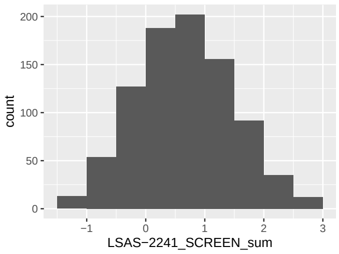

MADRS-1951_SCREEN_DateCompleted_day

Feature	Result
Variable type	numeric
Number of missing obs.	0 (0 %)
Number of unique values	7
Median	0.37
1st and 3rd quartiles	-1; 1
Min. and max.	-1.37; 1.37

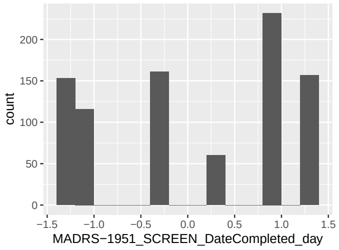

MADRS-1951_SCREEN_DateCompleted_time

Feature	Result
Variable type	numeric
Number of missing obs.	0 (0 %)
Number of unique values	561
Median	-0.65
1st and 3rd quartiles	-1.19; 0.34
Min. and max.	-1.41; 1.41

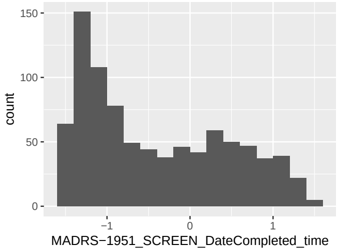

PDSS-SR-3064_SCREEN_DateCompleted_day

Feature	Result
Variable type	numeric
Number of missing obs.	0 (0 %)
Number of unique values	7
Median	0.37
1st and 3rd quartiles	-1; 1
Min. and max.	-1.37; 1.37

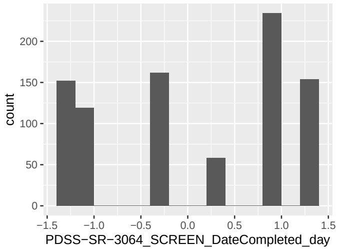

PDSS-SR-3064_SCREEN_DateCompleted_time

Feature	Result
Variable type	numeric
Number of missing obs.	0 (0 %)
Number of unique values	542
Median	-0.59
1st and 3rd quartiles	-1.19; 0.39
Min. and max.	-1.41; 1.41

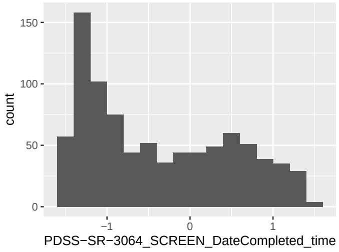

LSAS-2241_SCREEN_DateCompleted_day

Feature	Result
Variable type	numeric
Number of missing obs.	0 (0 %)
Number of unique values	7
Median	0.37
1st and 3rd quartiles	-1; 1
Min. and max.	-1.37; 1.37

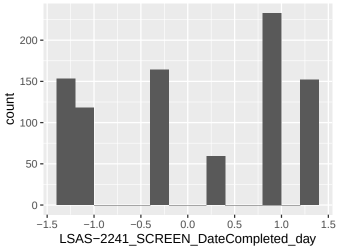

LSAS-2241_SCREEN_DateCompleted_time

Feature	Result
Variable type	numeric
Number of missing obs.	0 (0 %)
Number of unique values	561
Median	-0.58
1st and 3rd quartiles	-1.2; 0.43
Min. and max.	-1.41; 1.41

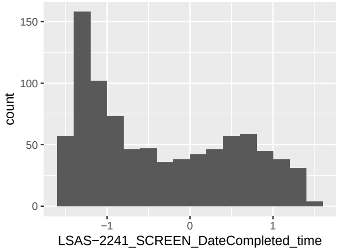

outcome

Feature	Result
Variable type	numeric
Number of missing obs.	0 (0 %)
Number of unique values	115
Median	-0.13
1st and 3rd quartiles	-0.75; 0.54
Min. and max.	-2.05; 3.53

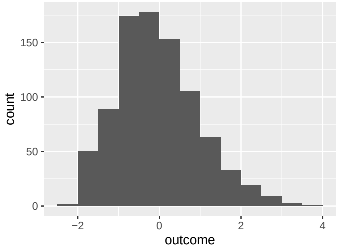

ncomorbid

Feature	Result
Variable type	numeric
Number of missing obs.	0 (0 %)
Number of unique values	5
Median	0
1st and 3rd quartiles	0; 1
Min. and max.	0; 4

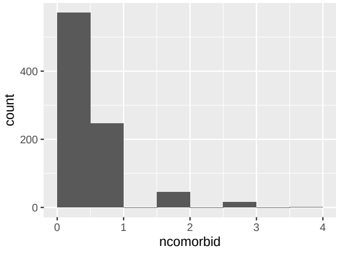

currentwork_proff

Feature	Result
Variable type	factor
Number of missing obs.	0 (0 %)
Number of unique values	2
Mode	“1”
Reference category	0

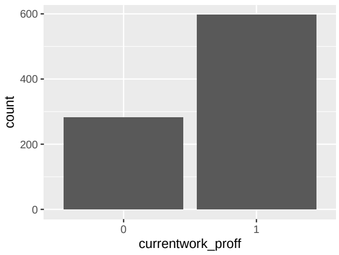

- Observed factor levels: "0", "1".

Marital_1833_gift

Feature	Result
Variable type	factor
Number of missing obs.	0 (0 %)
Number of unique values	2
Mode	“1”
Reference category	0

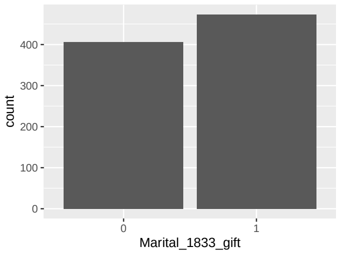

- Observed factor levels: "0", "1".

Marital_1833_separerad

Feature	Result
Variable type	factor
Number of missing obs.	0 (0 %)
Number of unique values	2
Mode	“0”
Reference category	0

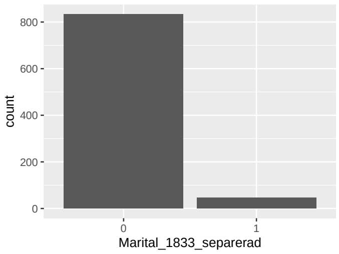

- Observed factor levels: "0", "1".

Marital_1833_singel

Feature	Result
Variable type	factor
Number of missing obs.	0 (0 %)
Number of unique values	2
Mode	“0”
Reference category	0

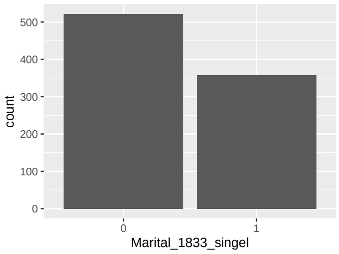

- Observed factor levels: "0", "1".

Edu_1843_2

Feature	Result
Variable type	factor
Number of missing obs.	0 (0 %)
Number of unique values	2
Mode	“0”
Reference category	0

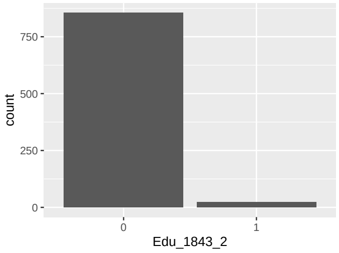

- Observed factor levels: "0", "1".

Edu_1843_3

Feature	Result
Variable type	factor
Number of missing obs.	0 (0 %)
Number of unique values	2
Mode	“0”
Reference category	0

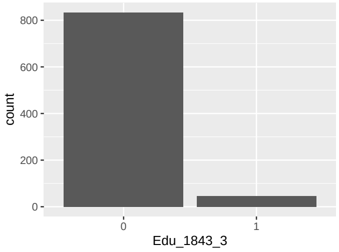

- Observed factor levels: "0", "1".

Edu_1843_4

Feature	Result
Variable type	factor
Number of missing obs.	0 (0 %)
Number of unique values	2
Mode	“0”
Reference category	0

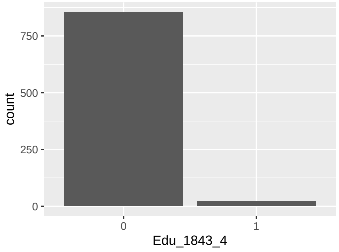

- Observed factor levels: "0", "1".

Edu_1843_5

Feature	Result
Variable type	factor
Number of missing obs.	0 (0 %)
Number of unique values	2
Mode	“0”
Reference category	0

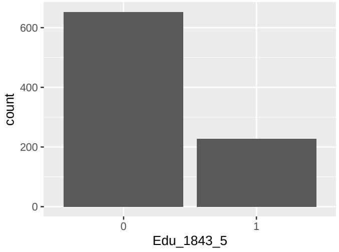

- Observed factor levels: "0", "1".

Edu_1843_6

Feature	Result
Variable type	factor
Number of missing obs.	0 (0 %)
Number of unique values	2
Mode	"0"
Reference category	0

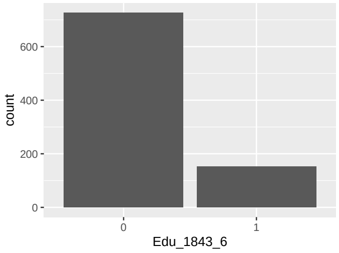

- Observed factor levels: "0", "1".

Edu_1843_7

Feature	Result
Variable type	factor
Number of missing obs.	0 (0 %)
Number of unique values	2
Mode	"0"
Reference category	0

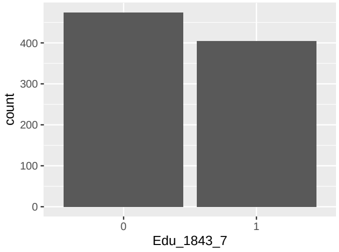

- Observed factor levels: "0", "1".

---

cscale

Feature	Result
Variable type	numeric
Number of missing obs.	0 (0 %)
Number of unique values	43
Median	0.13
1st and 3rd quartiles	-0.46; 0.72
Min. and max.	-3.31; 1.79

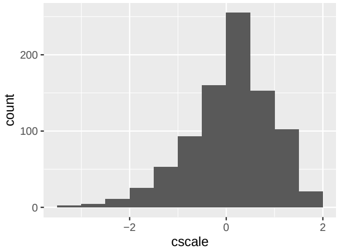

---

mainsymptom_PRE_sum

Feature	Result
Variable type	numeric
Number of missing obs.	0 (0 %)
Number of unique values	112
Median	-0.04
1st and 3rd quartiles	-0.68; 0.59
Min. and max.	-2.49; 3

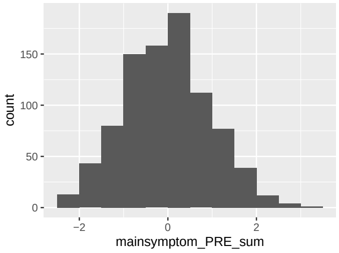

---

mainsymptom_PRE_duration

Feature	Result
Variable type	numeric
Number of missing obs.	0 (0 %)
Number of unique values	522
Median	-0.14
1st and 3rd quartiles	-0.24; 0.01
Min. and max.	-0.44; 8.71

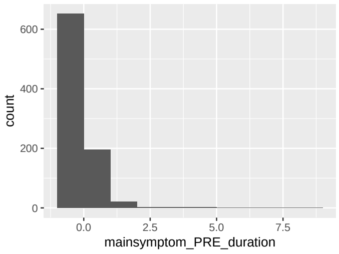

mainsymptom_PRE_DateCompleted_day

Feature	Result
Variable type	numeric
Number of missing obs.	0 (0 %)
Number of unique values	7
Median	0.37
1st and 3rd quartiles	-0.37; 1
Min. and max.	-1.37; 1.37

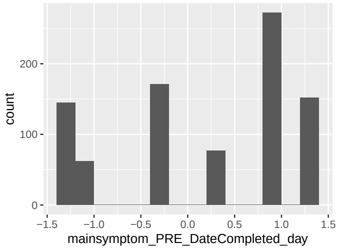

mainsymptom_PRE_DateCompleted_time

Feature	Result
Variable type	numeric
Number of missing obs.	0 (0 %)
Number of unique values	534
Median	-0.61
1st and 3rd quartiles	-1.19; 0.19
Min. and max.	-1.41; 1.41

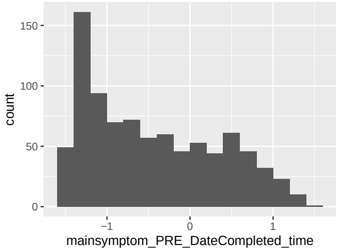

mainsymptom_WEEK01_sum

Feature	Result
Variable type	numeric
Number of missing obs.	0 (0 %)
Number of unique values	116
Median	-0.05
1st and 3rd quartiles	-0.66; 0.57
Min. and max.	-2.84; 2.87

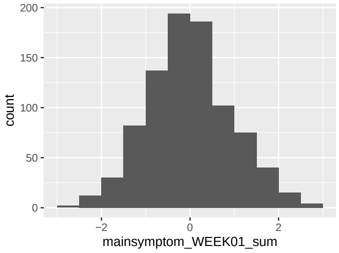

mainsymptom_WEEK01_duration

Feature	Result
Variable type	numeric
Number of missing obs.	0 (0 %)
Number of unique values	402
Median	-0.17
1st and 3rd quartiles	-0.27; 0
Min. and max.	-0.51; 17.57

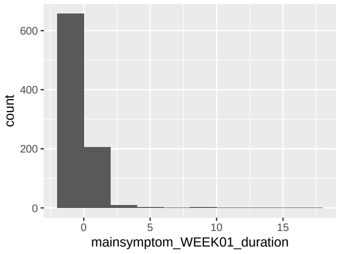

mainsymptom_WEEK01_DateCompleted_day

Feature	Result
Variable type	numeric
Number of missing obs.	0 (0 %)
Number of unique values	7
Median	1
1st and 3rd quartiles	-1; 1
Min. and max.	-1.37; 1.37

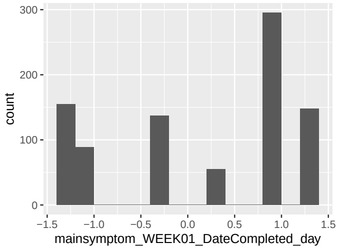

mainsymptom_WEEK01_DateCompleted_time

Feature	Result
Variable type	numeric
Number of missing obs.	0 (0 %)
Number of unique values	552
Median	-0.58
1st and 3rd quartiles	-1.19; 0.18
Min. and max.	-1.41; 1.41

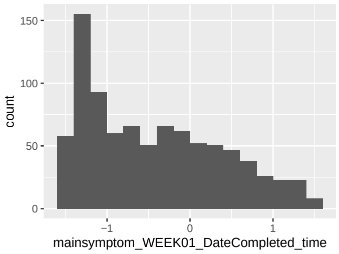

mainsymptom_WEEK02_sum

Feature	Result
Variable type	numeric
Number of missing obs.	0 (0 %)
Number of unique values	120
Median	-0.11
1st and 3rd quartiles	-0.72; 0.58
Min. and max.	-2.75; 2.97

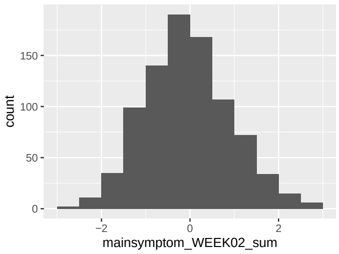

mainsymptom_WEEK02_duration

Feature	Result
Variable type	numeric
Number of missing obs.	0 (0 %)
Number of unique values	407
Median	-0.18
1st and 3rd quartiles	-0.24; -0.05
Min. and max.	-0.46; 11.6

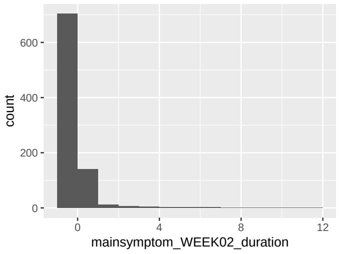

mainsymptom_WEEK02_DateCompleted_day

Feature	Result
Variable type	numeric
Number of missing obs.	0 (0 %)
Number of unique values	7
Median	1
1st and 3rd quartiles	-0.37; 1
Min. and max.	-1.37; 1.37

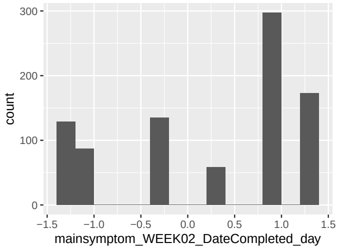

mainsymptom_WEEK02_DateCompleted_time

Feature	Result
Variable type	numeric
Number of missing obs.	0 (0 %)
Number of unique values	569
Median	-0.66
1st and 3rd quartiles	-1.21; 0.11
Min. and max.	-1.41; 1.41

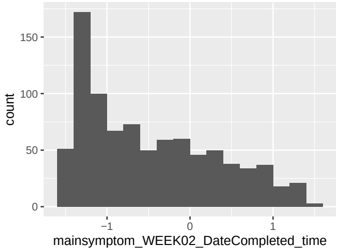

mainsymptom_WEEK03_sum

Feature	Result
Variable type	numeric
Number of missing obs.	0 (0 %)
Number of unique values	120
Median	-0.11
1st and 3rd quartiles	-0.71; 0.62
Min. and max.	-2.64; 3.07

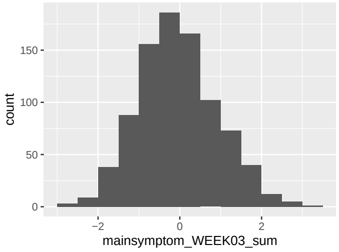

mainsymptom_WEEK03_duration

Feature	Result
Variable type	numeric
Number of missing obs.	0 (0 %)
Number of unique values	371
Median	-0.13
1st and 3rd quartiles	-0.17; -0.07
Min. and max.	-0.24; 8.75

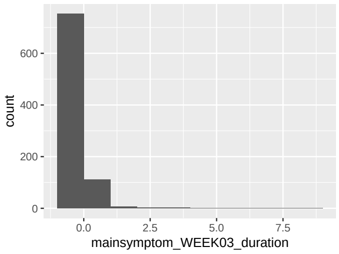

mainsymptom_WEEK03_DateCompleted_day

Feature	Result
Variable type	numeric
Number of missing obs.	0 (0 %)
Number of unique values	7
Median	0.37
1st and 3rd quartiles	-0.37; 1
Min. and max.	-1.37; 1.37

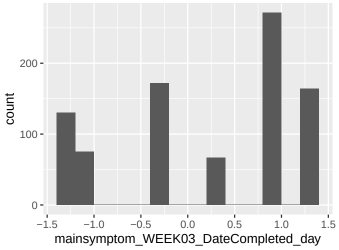

mainsymptom_WEEK03_DateCompleted_time

Feature	Result
Variable type	numeric
Number of missing obs.	0 (0 %)
Number of unique values	548
Median	-0.55
1st and 3rd quartiles	-1.14; 0.16
Min. and max.	-1.41; 1.41

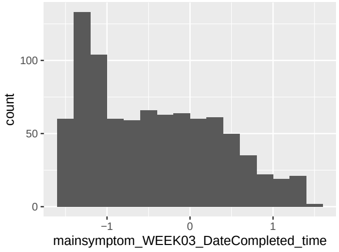

Report generation information:

- Created by: Could not determine from system (username: `nilisa`).
- Report creation time: Mon Jan 09 2023 13:09:55
- Report was run from directory: `/home/nilisa/projects/phd_study1/r`
- dataMaid v1.4.1 [Pkg: 2021-10-08 from CRAN (R 4.2.2)]
- R version 4.2.2 Patched (2022-11-10 r83330).
- Platform: x86_64-pc-linux-gnu (64-bit)(Ubuntu 20.04.5 LTS).
- Function call: `dataMaid::makeDataReport(data = gd, mode = c("summarize", "visualize", "check"), smartNum = FALSE, file = "~/projects/data/study1multiverse/results/graphs_n_figures/codebooks/codebook replace = TRUE, openResult = FALSE, checks = list(character = "showAllFactorLevels", factor = "showAllFactorLevels", labelled = "showAllFactorLevels",   haven_labelled = "showAllFactorLevels", numeric = NULL,   integer = NULL, logical = NULL, Date = NULL), listChecks = FALSE, maxProbVals = Inf, codebook = TRUE, reportTitle = "Handpicked_Social_week04-narem`
